# Supplementary material for: A Non‐Canonical Role of SMAD4 in Regulating 3D Genome Architecture to Inhibit Lung Squamous Cell Carcinoma Development
Source: Adv Sci (Weinh). 2026 May 26;13(47):e75839. doi: 10.1002/advs.75839 (PMC13335900; doi:10.1002/advs.75839)
Supplement: Supplementary file 1 [file ADVS-13-e75839-s001.pdf]

Supporting Information

Qian Tang[1,2], Chen Lian [1], Xinyan Han [1,2], Xuhan Zhang[3,1], Zihan Wang[1,2], Boyu Wang[1],Taoyu Zhu[1,2], Xinrui Lin[1,2], Xiaolei Wang[1,2], Yaping Xu[1,2], Manyu Xiao[1], Zijin Wang[1], Junmin Li[1,2], Silin Chen[1], Yunze Wang[1,2], Yufei Liu[1,2], Songsong Li[1], Zuolin Shen[1,2], Xi Lu[1], Xueqi Han[1], Yilu Zhou[1], Mingyang Xiao[1,2], Jiayi Ran[1,2], Xiaoran Cao[1,2], Xinyi Xu[1,2], Hugo Sámano-Sánchez[1,2], Alfredo Rodríguez[4,5], Lian Wang[6], Shuifang Chen[7,8], Zhanyu Xu[9], Shirong Zhang[10], Nuo Yang[9], Yong Tang[11]*, Jian Liu[1,2,12,13,14,15]**

Supplementary Materials

Supplementary Figure 1
Supplementary Figure 2
Supplementary Table 1. gRNA-*Smad4* vs. gRNA-Control DEGs
Supplementary Table 2. SMAD4 Peak Annotation
Supplementary Table 3. H3K27ac Peak Annotation
Supplementary Table 4. Summary of Primers
Supplementary Table 5. Summary of Guide RNA (gRNA) Sequences
Supplementary Table 6. Summary of siRNA Sequences
Supplementary Table 7. Summary of antisense oligonucleotides (ASO)

Supplementary Figure 1

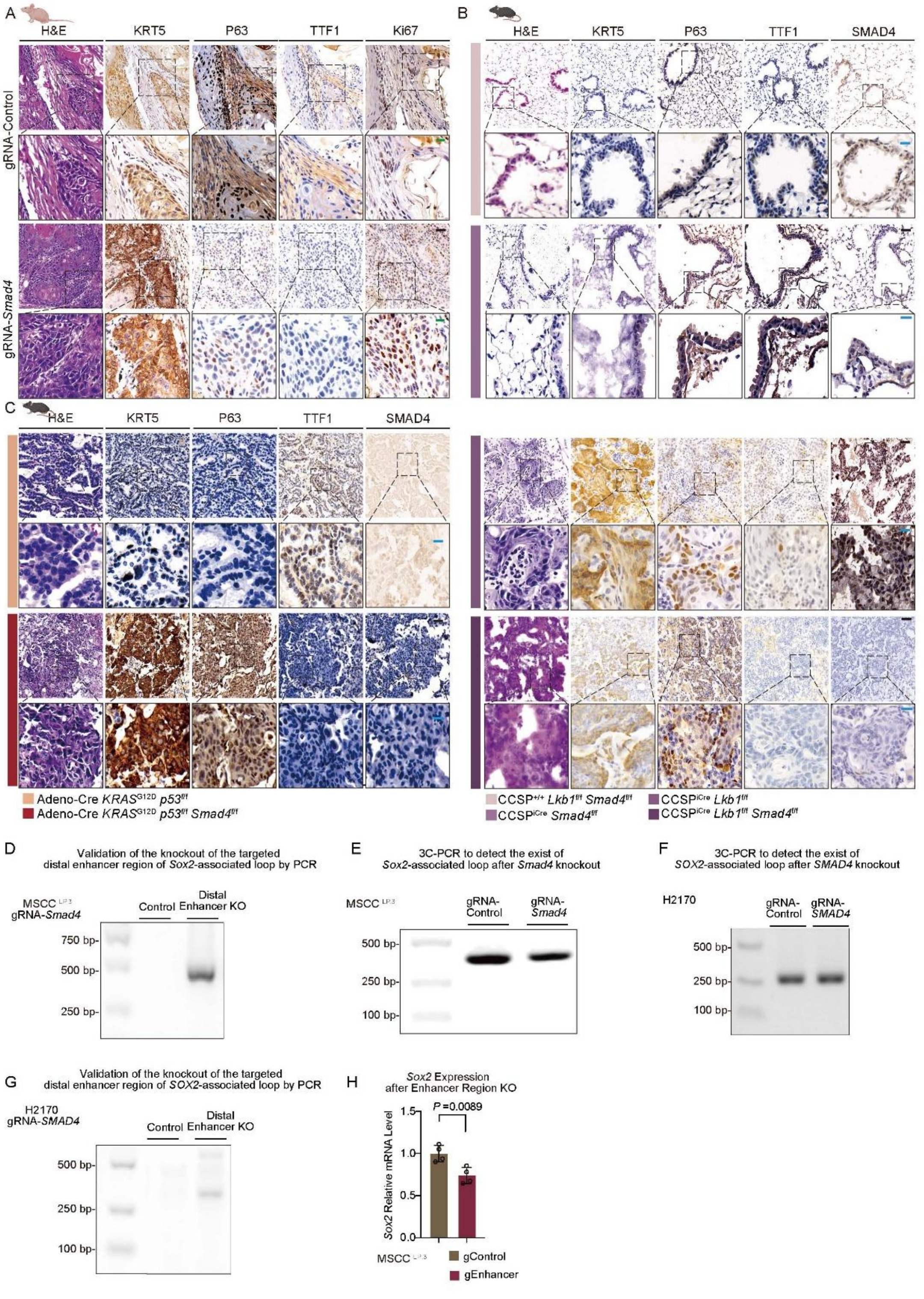

A. H&E staining and IHC staining of LUSC markers KRT5 and TP63, LUAD marker TTF1, and cell proliferation marker Ki67 in MSCC$^{LP.3}$ gRNA-Control and gRNA-*Smad4* xenograft tumors.

B. H&E staining and KRT5, TP63, TTF1 and SMAD4 IHC staining in CCSP$^{+/+}$ *Lkb1*$^{f/f}$ *Smad4*$^{f/f}$, CCSP^{iCre}*Smad4*$^{f/f}$, CCSP^{iCre}*Lkb1*$^{f/f}$, and CCSP^{iCre}*Lkb1*$^{f/f}$*Pten*$^{f/f}$ mice.

C. H&E staining and KRT5, TP63, TTF1 and SMAD4 IHC staining in Adeno-Cre *Kras*^{G12D} *Trp53*$^{f/f}$ and Adeno-Cre *Smad4*$^{f/f}$ *Kras*^{G12D} *Trp53*$^{f/f}$ mice.

D. PCR to validate the knockout of the targeted distal enhancer region of *Sox2*-associated loop in MSCC$^{LP.3}$ gRNA-*Smad4* cells.

E. 3C-PCR to detect the exist of *Sox2*-associated loop before and after *Smad4* knockout in MSCC$^{LP.3}$ cell line.

F. 3C-PCR to detect the exist of *SOX2*-associated loop before and after *SMAD4* knockout in H2170 cell line.

G. PCR to validate the knockout of the targeted distal enhancer region of *SOX2*-associated loop in H2170 gRNA-*SMAD4* cells.

H. RT-qPCR to detect *Sox2* expression followed by the knockout of the enhancer region of *Sox2*-associated loop in MSCC$^{LP.3}$ gRNA-Control cell line.

Supplementary Figure 2

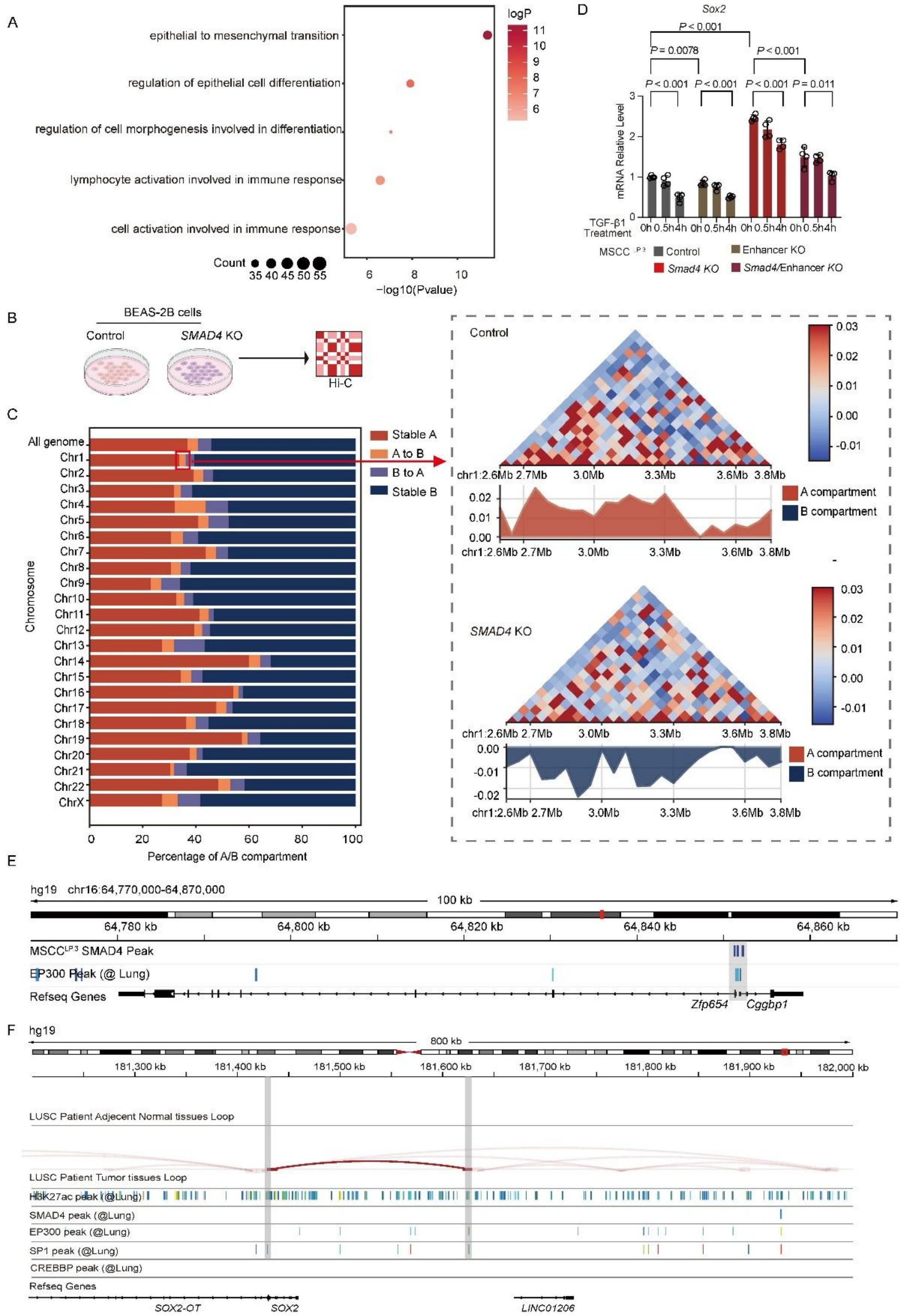

A. GO term pathway enrichment results based on differentially expressed genes between *Smad4* knockout and control groups.

B. qPCR analyses of the regulation of TGF-β on the *Sox2* transcription.

C. Hi-C analysis of *SMAD4* knockout and control BEAS-2B cell lines.

D. Changes in A/B compartments on various chromosomes before and after *SMAD4* knockout in BEAS-2B cell lines.

E. SMAD4 and EP300 binding at the *Zfp654* and *Cggpb1* promoter region in mouse lung cell line.

F. Proteins binding profiling (ChIP-Seq data of lung cell lines in public databases ChIP-Atlas) on the *SOX2*-associated loop anchor regions.

Supplementary Figure 3

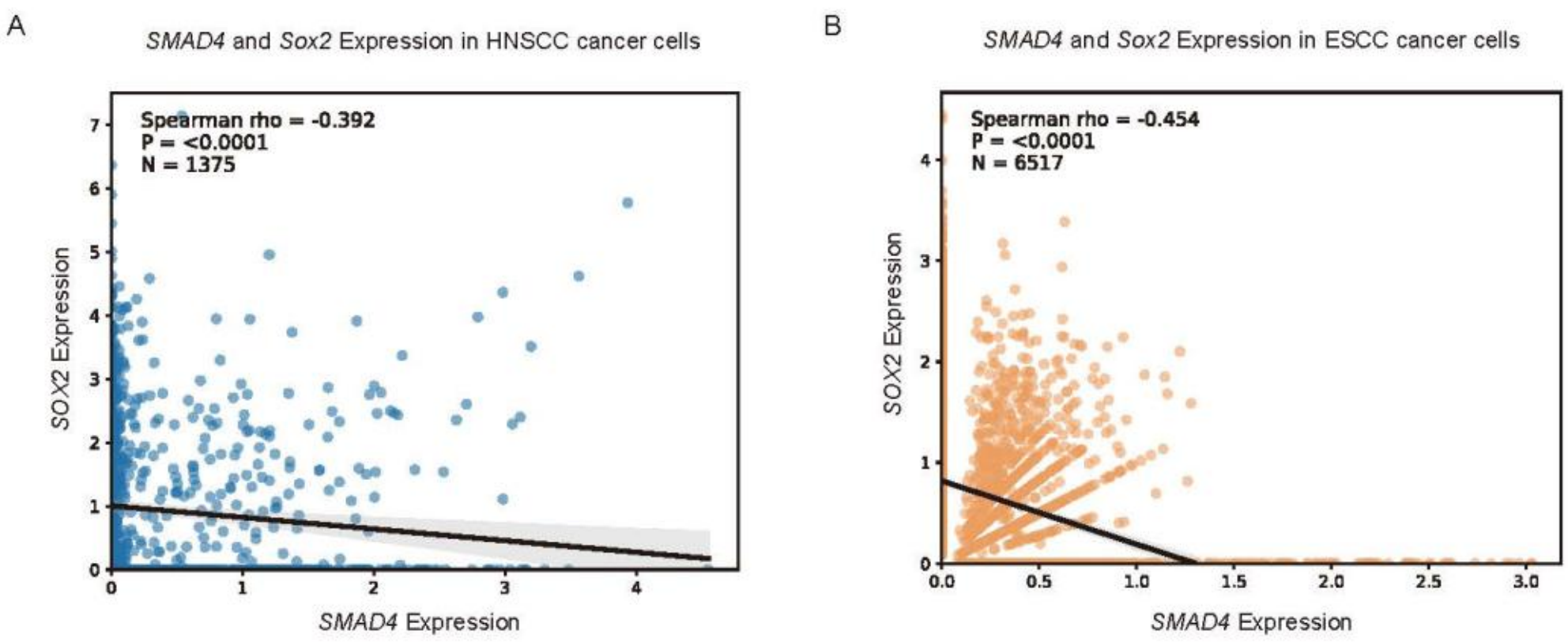

A. Correlation between *SMAD4* and *SOX2* Expression in Tumor Cells in Single-Cell RNA-Seq Data from Human Head and Neck Squamous Cell Carcinoma (HNSCC).

B. Correlation between *SMAD4* and *SOX2* expression in tumor cells in single-cell RNA-Seq Data from Esophageal Squamous Cell Carcinoma (ESCC).

Supplementary Table 1. gRNA-*Smad4* vs. gRNA-Control DEGs

gene_id	log2FoldChange	FDR
Ccl20	1.276047989	4.62E-14
Slc29a2	1.168429943	8.26E-20
Krt15	1.098585554	1.12E-10
Bmp2	0.955949521	3.30E-08
Scd2	0.94391639	4.61E-63
Krt7	0.913203583	3.73E-31
Aldh3a1	0.905833848	1.12E-08
Tnfaip2	0.870119104	1.88E-20
Tmprss11e	0.851693114	7.48E-17
Ces1g	0.844728874	1.12E-10
Akap12	0.797176595	1.32E-15
Prl2a1	0.736194317	5.09E-11
Ly6c1	0.735054685	5.53E-08

Adgrl3	0.730877598	3.96E-05
Fgfbp1	0.719500494	4.95E-13
Stfa3	0.707331967	7.59E-05
Prodh	0.702424165	7.14E-07
Eps8	0.700150182	6.26E-05
Ska1	0.697680801	7.31E-05
Nrg2	0.697385472	1.50E-15
Efemp1	0.683304321	0.000174
Gm22973	0.681324539	0.000108
Hist1h3d	0.679971086	3.13E-14
Krt18	0.67374866	1.28E-11
Hist1h1b	0.661629691	2.46E-27
Ltbp1	0.657874252	1.58E-09
Lhfp	0.649401268	1.92E-18
Hist1h2ae	0.647896656	4.92E-10
Robo2	0.646371234	3.86E-11
Hist1h3h	0.644857447	2.66E-14
Hist1h2af	0.642038226	1.21E-08
Barx2	0.641615691	0.000362
Igf2r	0.640859194	5.50E-18
Depdc1b	0.628455467	1.41E-06
Cdc20	0.623487504	1.29E-22
Hist1h2bj	0.623103543	2.09E-15
Kif22	0.618595471	3.02E-16
Serpine2	0.616285127	1.44E-17
Hist2h2ac	0.61536397	1.04E-12
Nr4a1	0.614678251	0.000213
Slc16a3	0.614163952	5.59E-20
Gm24407	0.612603954	2.80E-05
Hist1h3c	0.610781673	2.30E-15
Hist1h2an	0.609786935	1.56E-06
Fam213a	0.609374153	0.000217
Gap43	0.605243457	3.68E-05
Cdk14	0.601528528	0.00052
Hist1h2bh	0.600623974	3.39E-16
Hist1h2ad	0.600504638	3.38E-08
Hs3st3b1	0.594626737	8.21E-18
Bdnf	0.58796371	0.001558
Psrc1	0.587381685	6.83E-08
Fam111a	0.584364407	2.27E-13
Hist1h4d	0.584262152	7.85E-24
Hist1h2bb	0.579586589	1.38E-22
Hist1h2bn	0.579184885	9.54E-08
Hist1h2be	0.578515061	1.39E-15

Lcn2	0.578453416	0.002033
Tfap4	0.577998682	4.26E-07
Hist1h2bk	0.577459404	4.34E-14
Glyat	0.575172708	0.001919
Hist1h2ag	0.575107875	2.46E-06
Hist1h3b	0.571499825	0.000104
Hist1h2bg	0.570122518	1.05E-17
Lrrc8b	0.56905291	4.57E-06
B4galnt4	0.568551841	9.96E-07
Hist1h2ac	0.568378278	4.03E-11
Hist1h1e	0.566606489	6.19E-20
Pif1	0.56612094	8.61E-08
Iqgap3	0.564341554	1.27E-13
Slc1a5	0.564275893	5.51E-14
Hist1h4b	0.563495824	9.25E-14
Ak3	0.560830803	3.08E-10
Slc7a5	0.559384633	2.26E-10
Evc2	0.558877982	0.003075
Tmem229b	0.557473852	0.001666
Tgm1	0.556942839	0.001552
Ncaph	0.552591528	1.32E-15
Hist1h1d	0.550621563	6.64E-18
Ccnb1	0.549795724	3.45E-12
Hist1h4h	0.542773478	7.48E-17
Pde3b	0.542717509	0.000648
Hist1h3g	0.542680369	4.60E-11
Rfc5	0.542221132	2.90E-15
Hist1h4k	0.541978465	1.65E-15
Nek2	0.541853321	6.00E-15
Slc6a14	0.537116077	0.004796
Hist1h2bp	0.533897395	5.55E-08
Hist1h3i	0.532988898	9.81E-11
Ncapg	0.531957031	4.24E-14
Hist2h2bb	0.531411923	2.67E-05
Car12	0.530677271	6.53E-16
Hist1h1a	0.530027486	4.00E-11
Cenpu	0.529961898	2.48E-05
Ect2	0.528224145	2.28E-11
Hist1h4a	0.52791201	2.21E-13
Lyar	0.527691032	9.00E-10
Trip13	0.527111341	1.62E-08
Bub1b	0.525073573	2.25E-13
Fkbp5	0.524509772	0.00013
Hist1h2bm	0.522194007	1.01E-07

Shmt1	0.522139545	6.32E-08
Cebpa	0.517530198	0.00697
Kif2c	0.516521871	5.52E-11
Il1f6	0.515962199	8.23E-06
Nsl1	0.515882623	4.65E-07
Abcb1b	0.514811539	3.30E-15
Hist1h2bc	0.513177377	7.84E-11
Cks2	0.511461976	1.23E-06
Plk1	0.51085464	6.75E-11
P3h1	0.510379491	0.005592
Cep128	0.508355215	3.51E-05
Hist1h4f	0.508231736	1.04E-14
Chchd4	0.508089074	4.14E-11
Ifrd2	0.50624756	1.84E-09
Slc35f2	0.505704368	0.00817
RP24-112H7.1	0.504180773	0.004862
Dhrs1	0.503564193	9.51E-13
Hirip3	0.502179003	2.82E-06
Cad	0.502165736	9.05E-18
Hist1h3e	0.49998519	7.23E-11
Zwilch	0.49979647	6.18E-10
Hist1h3f	0.499567542	3.22E-11
Cdyl2	0.499263162	0.008696
Esco2	0.498726872	1.26E-06
Hist4h4	0.497423254	1.54E-13
Tacc3	0.497064347	5.56E-17
Anln	0.495610369	5.41E-12
Trp73	0.494864416	0.001211
Apln	0.494523711	0.010365
Arrdc4	0.494354168	5.12E-08
Ccna2	0.494145771	3.57E-05
Rrp1b	0.492711577	7.89E-11
Cenpn	0.492479548	2.09E-05
Zbed5	0.491916128	0.009328
Slc25a48	0.490744666	2.84E-09
Itgb7	0.490059905	8.77E-06
Esr1	0.489456051	0.010582
Diaph3	0.488174014	1.99E-11
Krt19	0.487899979	0.000144
Rrp12	0.48753969	1.23E-14
Hist1h2ab	0.485683369	0.001293
Aurkb	0.485095562	4.05E-09
Dtwd1	0.485062541	7.78E-09
Dut	0.485003268	8.42E-12

Apcdd1	0.484923845	0.010426
Aunip	0.484722796	0.002347
Tmem74	0.483781146	0.01034
Sh3rf2	0.483494153	1.28E-05
Uhrf1	0.482504952	2.74E-12
Dctpp1	0.482298837	1.13E-06
Hist1h4j	0.482162683	1.67E-08
Ndc1	0.480424592	1.34E-10
Krt8	0.480329401	2.66E-13
Hist1h4i	0.478972524	4.28E-10
Snord17	0.478275367	8.34E-05
Tdp1	0.47818147	9.27E-05
Muc15	0.478016629	0.014069
Recql4	0.477170555	5.41E-06
Dlgap5	0.476823062	9.21E-11
Gm22620	0.476379223	0.011796
Slc19a1	0.476138893	2.36E-06
Htr5b	0.475089414	0.01472
Srm	0.474770766	4.08E-10
Gm23130	0.474595159	0.004987
Prc1	0.474266766	2.69E-12
Dgkh	0.472504005	2.14E-05
Hist2h4	0.471772688	7.98E-08
Kif11	0.471037472	2.62E-16
Cmss1	0.470374597	0.000117
Tmem194	0.469931997	0.000109
Gm25395	0.469603228	0.002959
Aspm	0.469203308	3.01E-12
Snora78	0.468882704	0.000185
Lrig1	0.468506035	2.24E-16
Akip1	0.468153172	0.016098
Hist1h2ak	0.468032671	0.00058
Eme1	0.467869999	4.84E-05
Fam72a	0.467829433	0.005995
Cbx6	0.467828459	3.56E-08
Spc24	0.467288483	5.09E-09
Pbk	0.467032963	6.82E-08
Snord93	0.466456998	0.017147
Chtf18	0.465725521	1.37E-08
Card14	0.465665089	0.008029
Cdca2	0.46427333	5.88E-08
Espl1	0.464117122	1.79E-06
Racgap1	0.463285607	1.56E-12
Fanci	0.463125084	2.97E-07

Rad54l	0.462861568	4.11E-05
Hist1h2bl	0.461702028	0.001092
Hist1h4c	0.461257678	2.27E-10
Pla2r1	0.461068692	0.00036
Tcof1	0.460987499	1.21E-08
Mns1	0.460563844	0.007417
Mad2l1	0.46009432	3.87E-07
Gm22422	0.459574477	0.008713
Timm8a1	0.459010159	0.00871
Lgals7	0.45884349	0.014865
RP23-461C4.3	0.4585796	0.001001
Cdk1	0.458322296	7.23E-11
Hmmr	0.457352558	4.87E-12
Wdr12	0.456260826	1.04E-13
H2afx	0.45604946	9.30E-10
Gm25835	0.454383796	0.004033
Atp13a4	0.454145421	0.018069
Ttk	0.453805288	9.81E-11
Rabggta	0.453387348	1.34E-05
Hist2h3b	0.453312366	0.000244
Tgfbr3	0.452891772	0.003241
Aif1l	0.45284105	0.000173
Mis18bp1	0.452739062	9.94E-06
Gdpd2	0.451283769	0.01845
Hist1h1c	0.451274402	8.45E-12
Snord22	0.450665159	0.00087
Ampd1	0.450165768	0.017343
Ccnf	0.449763558	5.37E-08
Gm22068	0.44856128	0.018812
Pdss1	0.448503171	0.000113
Irs1	0.448412027	7.78E-06
Itgb2	0.447900786	0.018173
Ppat	0.4472818	3.25E-11
Pus7	0.446190854	5.27E-11
Rpl7l1	0.445346029	1.98E-18
Aurka	0.445180464	2.67E-12
Ramp3	0.44517528	0.006154
Kif18b	0.443815192	5.39E-07
RP23-110C17.2	0.442941713	0.014231
Mat2a	0.442215544	2.15E-16
Gen1	0.441302374	2.32E-05
Fancb	0.44097926	0.000822
Ung	0.440768489	2.38E-05
Lsm3	0.43910431	3.37E-06

Eri2	0.438121601	1.16E-05
Hist1h2ai	0.437724336	0.013565
Rnu3a	0.437647879	0.024782
Lin28b	0.437500628	0.004129
Hist1h2bf	0.436613832	6.40E-06
Nup37	0.436175577	8.61E-08
Kif4	0.436136474	9.72E-12
Kif20b	0.436018564	4.21E-13
Stom	0.435338081	5.38E-12
RP23-63D8.8	0.434994993	0.020208
Dhfr	0.434965054	3.90E-09
Gart	0.434474938	1.82E-16
Nup85	0.433270641	2.56E-12
Snora21	0.432823153	0.0297
Hdac5	0.432741199	0.015572
Mfsd2a	0.432553867	0.000148
Kntc1	0.432472482	1.74E-10
Gm24265	0.431959806	8.44E-06
Xrcc2	0.431691059	7.23E-06
RP24-160H16.10	0.431384835	0.009854
Exo1	0.431007856	5.17E-08
Gm25813	0.430228981	0.031104
Mphosph9	0.430181732	5.77E-05
Knstrn	0.429954104	3.66E-10
Bora	0.429684438	2.38E-05
Pim3	0.429053641	7.31E-06
Gemin6	0.428424161	0.00028
Sgol1	0.427973654	6.33E-06
Gm25360	0.427951614	0.009021
Suv39h1	0.427174871	9.55E-06
Atf3	0.427162241	0.006266
Snora73b	0.427130126	0.015845
Cenpf	0.426584389	2.93E-11
Siah1b	0.426580619	0.000139
Flot2	0.426495305	0.029437
Cenpi	0.426348164	1.10E-07
Birc5	0.425714809	3.29E-08
Rcc1	0.425628532	2.25E-07
Kifc1	0.425568822	4.82E-05
Cecr2	0.425327312	0.025923
Ankle1	0.423906664	0.00111
Spry1	0.423690879	0.034246
Pdzrn3	0.423676502	2.45E-10
Nup133	0.423562102	7.26E-12

Htra2	0.423314186	6.20E-06
Fignl1	0.422603412	3.71E-09
Plk4	0.422507626	1.49E-08
Mthfd2	0.422163342	3.10E-07
Cdca8	0.42215112	1.41E-07
Ube2c	0.422086018	5.32E-09
Pole	0.421588474	8.08E-09
Fbxo5	0.421444375	2.15E-06
Cenph	0.42135587	7.23E-05
Nav3	0.421146076	2.10E-05
Trmt61a	0.420167562	9.97E-05
Nup107	0.419484818	3.52E-10
Slc39a4	0.419433315	0.031532
Ovol2	0.41905999	0.025053
Glt1d1	0.418639048	0.000327
Dctd	0.417955657	1.31E-05
Mcm7	0.417233541	5.05E-11
Sema3e	0.417053281	8.34E-05
Hist1h2ah	0.416782276	0.02397
Camk1d	0.416736528	0.038159
Nuf2	0.416650518	1.10E-07
Gm24830	0.415550721	0.038839
Top2a	0.414485365	4.25E-11
Nop16	0.414434997	4.15E-08
Pold1	0.414014524	3.79E-07
Rny1	0.413444407	1.13E-05
Dis3	0.41328851	1.52E-09
Kif18a	0.412904121	1.37E-05
Tma16	0.412479374	0.000134
Mir17hg	0.412393594	0.001486
RP23-480B19.17	0.411283776	0.031966
Exosc8	0.411134883	2.88E-07
Traip	0.411128281	2.73E-05
Gsg2	0.410275869	6.87E-05
Grwd1	0.410148271	2.76E-08
Spag5	0.409732015	8.43E-07
Smc4	0.409682542	1.66E-10
RP23-79J21.2	0.40902355	0.031116
Ncapg2	0.408632253	9.22E-10
Fam83d	0.40835068	7.35E-06
Stfa3	0.408267266	0.039633
Rcl1	0.408254308	1.10E-10
Nrp1	0.407826752	4.57E-15
Plekhh1	0.4074827	0.000117

Lsm2	0.40646222	3.83E-06
RP23-300H4.1	0.406089104	0.001286
Serpina3g	0.406010467	0.040753
Cinp	0.40589474	1.01E-07
Kif14	0.405665243	2.32E-05
Man1a	0.40519762	6.51E-06
Txnip	0.405084844	3.82E-05
Cenpe	0.404415864	5.62E-08
Rbm19	0.404413584	8.46E-09
Troap	0.403903239	0.003236
Cyp3a13	0.403726241	0.000718
Cdc25c	0.40353349	3.50E-05
Mrto4	0.403140171	1.90E-07
Twist1	0.402962626	0.045992
Ptpn13	0.402333447	2.08E-20
Gsto1	0.401690217	1.44E-25
RP23-81H23.3	0.401607107	0.032796
Pros1	0.40079998	0.04778
Miip	0.400683403	2.45E-05
Serpinb10	0.400529667	8.07E-13
Snora31	0.400317624	0.048
Nop9	0.399736658	2.08E-08
Timeless	0.39966043	2.03E-07
Pi15	0.399492063	0.044859
Sorbs1	0.398994125	0.00655
Gsta1	0.398022808	0.02931
Ckap2l	0.397776881	1.14E-06
Clec2g	0.397351534	0.045426
Dph2	0.396643926	0.000358
Ptprz1	0.396249145	0.042832
Pla2g4a	0.395419036	5.40E-07
RP23-69P8.2	0.395240004	0.005209
Umps	0.394357086	3.50E-11
Asf1b	0.393946417	0.000189
Kif15	0.393750882	0.000241
Nrm	0.392800166	0.005557
Rrp15	0.391738787	3.69E-08
RP24-201C14.10	0.391199578	0.014516
Kifc5b	0.390917212	0.003046
Tnik	0.390315739	0.003601
Dnph1	0.390251409	0.001845
Fancd2	0.389453119	7.78E-06
Cenpw	0.388977951	0.000409
Incenp	0.387240225	0.004434

Zfp59	0.386677869	0.027656
Ndc80	0.386392538	3.14E-05
Cdca3	0.385316194	1.40E-08
Brip1	0.385015663	3.36E-06
Cyb5r1	0.38496207	1.52E-06
Rpp40	0.384802845	0.001554
Mettl1	0.384631549	0.000144
Slc16a1	0.384351772	6.92E-09
Ddx11	0.384139739	1.10E-06
Lbr	0.384075253	1.61E-12
Eppk1	0.383942735	1.21E-07
Rtel1	0.38387706	4.22E-07
Arhgap11a	0.383705974	1.28E-07
Adk	0.383016784	2.60E-07
Ppid	0.382923708	1.96E-09
Shcbp1	0.382735349	4.41E-06
Gpd2	0.382691678	1.15E-14
Foxg1	0.382672776	0.017721
Tyms	0.382478808	9.70E-08
St3gal6	0.381838055	0.012095
Hist2h2ab	0.381570185	0.023407
Ube2t	0.381301045	0.007748
Nolc1	0.380902506	8.16E-11
Cit	0.379183312	9.04E-05
Cenpq	0.379180416	0.001879
Endou	0.378901619	0.015771
3110082I17Rik	0.378719515	0.003624
Cks1b	0.377984108	2.31E-05
Cdc45	0.377979003	6.51E-07
Dbf4	0.37758975	8.80E-09
Polr1b	0.377451282	3.16E-10
Lmnb1	0.377380906	2.66E-10
Slc5a6	0.37674036	0.004756
Vmn1r43	0.37672585	0.011919
Casc5	0.376569799	2.64E-06
Patz1	0.376240916	0.017992
Metrn	0.376169767	0.023277
Lig1	0.375494173	2.10E-06
Tbl3	0.375042055	1.07E-07
Ddx21	0.375002689	3.05E-15
Nudt11	0.374962541	0.043981
Wnt3a	0.37474663	7.37E-06
Cdt1	0.373600172	5.20E-07
Gm26224	0.373276881	0.011228

Cenpm	0.373013315	0.011136
Tk1	0.372534349	5.96E-05
Nle1	0.37248581	0.00056
Mdm1	0.372328882	0.006394
RP23-313J14.6	0.372138096	0.000762
Mir1949	0.37187963	0.006532
Fen1	0.371855356	1.31E-09
Arhgap19	0.371309809	1.30E-06
Mybl2	0.370882904	0.000121
Hyls1	0.37010545	0.00142
Blm	0.36949868	0.000269
Ddx31	0.369375373	4.17E-08
Haus4	0.369249357	0.000104
Pik3ip1	0.369124932	0.042403
Ppan	0.368651516	1.34E-07
Gpsm2	0.368259174	6.14E-09
Csta1	0.367784324	0.002806
Ttc27	0.367502559	5.57E-06
Tfb2m	0.36688399	0.006872
RP23-139J18.1	0.366831623	0.000511
Wdr43	0.366830681	3.67E-10
Atic	0.365197116	3.02E-12
Gtpbp10	0.365088925	0.000227
Zfp850	0.36490757	0.007352
Plk2	0.364676771	3.28E-13
Gnl3	0.364335912	1.60E-08
Nol8	0.364082219	1.36E-07
Smyd5	0.36353718	1.12E-05
Cdc42ep3	0.363369684	5.47E-05
Grk6	0.362681404	4.95E-10
Hk2	0.362169477	4.99E-09
Ifitm3	0.361788461	1.22E-09
Ruvbl2	0.361765048	3.18E-09
Ckap2	0.361651943	2.96E-07
Lncenc1	0.361482138	1.20E-15
Prmt7	0.361335087	3.06E-07
Nol9	0.36125982	1.80E-09
Polq	0.361199099	0.000126
RP23-413D14.9	0.361176671	0.036325
Gar1	0.361078321	0.003107
Wdr46	0.361044567	2.10E-07
Rad51	0.360645986	0.000372
Ccdc85c	0.360287218	7.43E-05
Ncapd2	0.360161803	9.30E-06

G2e3	0.359474068	1.79E-05
Dkc1	0.359468659	1.09E-06
Foxm1	0.359416277	0.000119
Wdhd1	0.358447842	8.13E-06
Enoph1	0.357950685	1.68E-06
Wdr4	0.357581706	1.31E-05
Fam64a	0.357433787	7.37E-05
Snora68	0.357393341	0.025768
RP23-376B19.2	0.356902391	1.43E-05
Gls2	0.355964585	0.013766
Otud4	0.355487628	1.18E-07
Gm24044	0.354862165	0.041218
Dscc1	0.354859874	0.028013
Mcm2	0.354827934	1.08E-09
Cdca7l	0.354673361	0.00046
Trub1	0.354590885	0.000112
Nsun5	0.353294928	0.001302
Cenpa	0.352251335	2.15E-05
Hist1h3a	0.351825744	0.0004
Cenpp	0.351778246	0.042201
Shmt2	0.35128836	9.44E-09
Bub1	0.350854353	1.90E-06
Sapcd2	0.349094921	0.001358
L3mbtl2	0.348706382	3.96E-05
Cep85	0.348597665	0.0008
Ipmk	0.347764283	9.50E-06
Ercc6l	0.347152076	3.28E-06
Gtse1	0.346712131	7.16E-06
Rrp8	0.346257261	2.75E-06
Wdr73	0.344992352	0.000272
Wdr3	0.344599386	8.99E-08
Rad18	0.344063585	0.000342
Slc2a9	0.343757877	0.042179
Cdca5	0.343274006	0.00037
Epgn	0.342711246	3.34E-08
Cep89	0.342186691	0.001637
Cep55	0.341637276	1.47E-05
Csrp2	0.341474859	0.033875
Ska3	0.341435594	0.003548
Prkg2	0.341318669	2.52E-06
Polr1a	0.34076979	1.83E-09
Zbtb10	0.340420457	0.042401
Hnrnpdl	0.340356614	9.20E-10
Hilpda	0.340204009	0.019669

Rfc4	0.34009948	0.000103
Itpr1	0.339064954	0.002495
Melk	0.338902898	6.71E-05
Zc3h8	0.338705988	0.007187
Sox2	0.338648759	0.000124
Nusap1	0.338415996	7.61E-08
Slc30a4	0.338400231	3.94E-08
Spdl1	0.338340479	7.71E-05
Rrm1	0.338260335	8.13E-12
Npm3	0.338165114	6.83E-08
Cdc7	0.337084447	0.00085
Sacs	0.336869238	0.000922
Fpgs	0.33650483	6.65E-05
Cenpo	0.336228725	0.00853
Mboat1	0.33618362	1.74E-06
Wdr77	0.335930048	2.25E-05
Cep152	0.33586817	9.81E-05
Serpinb2	0.335457553	0.000263
Tmem109	0.335404725	3.42E-05
Pdcd2	0.335282737	0.002424
Nek7	0.334949504	5.16E-07
Rmi2	0.334474212	0.037482
Plk3	0.334403419	0.000113
Mtcl1	0.334356069	1.61E-06
RP23-9L16.5	0.33385995	7.32E-06
Nop56	0.333788457	1.23E-08
Wee1	0.333732603	0.000722
Vars	0.333704534	3.29E-08
Heatr1	0.333494627	5.32E-09
Mcm10	0.333361041	8.09E-06
Xrcc6	0.333292133	2.14E-05
Hjurp	0.332718614	1.63E-08
Parpbp	0.332424528	0.001236
Prmt1	0.332288835	3.93E-06
D030056L22Rik	0.332171765	0.00093
Pwp2	0.331679942	4.86E-08
Rpp14	0.33167441	6.11E-05
Suv39h2	0.331508978	0.003149
Npm1	0.331302137	2.02E-08
Sephs2	0.331192403	3.54E-07
Slfn9	0.330774858	0.000261
Utp15	0.330699805	1.26E-06
Timm9	0.329978488	0.004833
Fam110c	0.329577263	0.000532

Plekho2	0.329186018	0.000309
Apobr	0.329098123	0.020931
Mcm6	0.327912329	1.70E-12
Usp1	0.327478379	6.63E-05
Hnrnpd	0.327443988	3.10E-05
Dpy19l1	0.32742721	9.56E-07
Ddias	0.326722379	9.64E-06
Anp32e	0.32645772	0.000151
Pecr	0.32633265	0.025316
Sgol2a	0.326190288	1.79E-05
Gins3	0.326051292	0.009088
Rnf220	0.326033393	0.000131
Cdkn3	0.325681838	0.00839
Pmf1	0.325643536	0.001915
Pcnt	0.325316575	2.00E-08
Mdc1	0.325271637	0.007072
Qtrt1	0.324923148	0.002529
Ttll4	0.324682053	2.86E-07
Chka	0.324496534	2.96E-07
Zfp472	0.32383271	0.029622
Hspd1	0.323472503	1.75E-10
Cbx3	0.322745922	0.001631
Snhg5	0.322723372	1.53E-06
Rrs1	0.3216548	6.74E-06
Dnmt1	0.320970635	7.27E-08
Far1	0.320647022	1.92E-05
Ddx18	0.320450708	3.43E-06
Foxc1	0.320371323	0.000995
Nrp2	0.319949123	4.80E-06
Dram1	0.319669434	0.000955
Rfc3	0.319626069	0.000225
Mdn1	0.319579784	3.31E-07
Nop14	0.319530008	8.52E-07
Arhgef39	0.319461988	0.021863
Noa1	0.319084338	0.000281
Nup43	0.318898201	9.96E-06
Fjx1	0.318782859	2.94E-05
Rbmxl1	0.318738871	0.001302
Sssca1	0.318632736	0.000182
Dusp6	0.318630727	0.00127
Taf1d	0.318458626	4.14E-05
Dtl	0.318346423	4.75E-06
Chaf1a	0.318149361	8.07E-06
Arhgef1	0.317724202	4.57E-05

Adgrl2	0.317589278	0.01161
Nup205	0.316856916	3.43E-10
Mpp6	0.316085267	5.31E-05
Ppih	0.315938411	0.00748
Ces2e	0.315930456	6.47E-05
RP23-95L9.5	0.315927661	0.045373
Kif23	0.315821114	3.76E-06
Cenpl	0.315716405	0.003806
Nsd2	0.315610069	6.07E-08
Kpna2	0.315541797	9.00E-07
Socs1	0.315368419	0.03102
Pms2	0.315278127	0.003138
Gas2l3	0.315104907	0.000108
Gramd4	0.314963784	0.000151
Mettl13	0.314608803	0.000334
Utp14a	0.31434641	1.86E-06
Telo2	0.314152756	0.000981
Noc4l	0.314068189	8.21E-05
Lin54	0.313809864	7.34E-05
Mcam	0.313800303	6.12E-08
Uck2	0.313544472	3.94E-08
Urb1	0.31330314	9.75E-08
Sdad1	0.313259439	8.04E-08
RP23-185B14.1	0.313226615	0.040563
Ccnb2	0.312258269	1.10E-05
Psat1	0.311498115	2.97E-11
Dnajc9	0.311387122	7.39E-05
Prim1	0.311352189	5.11E-05
Tmpo	0.311334471	4.17E-08
Rfc1	0.311244255	2.87E-09
Vars2	0.310656997	0.000555
Mcm8	0.3105868	0.007687
Slc12a2	0.310028119	1.01E-07
Dsn1	0.309887039	0.001486
Fancm	0.30898157	0.000141
Map3k14	0.3088933	0.008617
Gm25878	0.308492173	0.03186
Gfer	0.308363422	0.000329
Chek2	0.308308106	0.000244
Gpn3	0.308150775	0.000722
Fxn	0.30813873	0.022627
Dgkd	0.307942707	1.39E-05
Slbp	0.307356571	8.62E-06
Paics	0.307190217	7.25E-08

Rhoc	0.30717742	0.040641
Sbsn	0.307153088	1.20E-06
Fam216a	0.307027693	0.000661
Ruvbl1	0.30699062	0.000811
Ddx20	0.30673715	3.90E-05
Rpp38	0.306608749	0.003726
D2Wsu81e	0.306357007	0.000553
Gnb1l	0.306088186	0.015464
Noc2l	0.305952203	1.12E-05
Polr2h	0.305726206	0.000177
Tyw1	0.305669148	0.00033
P2rx7	0.305527863	0.044166
Nob1	0.305248446	0.001509
Galnt18	0.304666995	0.000811
Acer3	0.304264689	1.23E-05
Otud1	0.304234413	0.000778
Gcsh	0.30410999	1.61E-05
Faap24	0.303730465	0.039115
Mnd1	0.303448274	0.028645
Polr3g	0.303445354	4.77E-05
Spata5	0.302842491	0.000181
Ipo5	0.302204595	1.99E-12
Haus6	0.302199585	9.31E-05
Wdr90	0.301943561	0.001779
Rps6ka2	0.301473614	0.016483
Cpsf3	0.301179248	7.61E-05
Nop58	0.30076325	3.52E-07
Sart3	0.300304856	5.48E-07
Ilf3	0.300287269	1.02E-06
Fhl1	0.300232914	0.000195
Sgsm2	-0.300183167	0.003089
Samd5	-0.300413566	0.0002
Sh3pxd2a	-0.300426868	1.28E-13
Il24	-0.300716506	7.36E-05
Aff1	-0.300771993	1.70E-06
Eef2k	-0.30095853	3.83E-06
Baiap2l1	-0.301088615	0.017602
Slc2a1	-0.301593128	2.49E-08
Slc35f6	-0.301794983	1.00E-05
Pthlh	-0.301851933	8.66E-09
Ocrl	-0.302162452	0.001358
Sphk1	-0.302349816	0.036029
Npepps	-0.303042672	7.61E-07
Nhsl1	-0.303478668	0.022513

Mical3	-0.303547553	3.92E-06
Slc44a1	-0.303721106	3.61E-09
Nectin1	-0.304635424	0.000137
Cpt2	-0.304734232	0.017733
Igfbp7	-0.305512097	0.000218
Lynx1	-0.305538043	0.003431
Rab8b	-0.306663892	5.57E-06
Plcd1	-0.30669871	5.57E-06
Ube2j1	-0.306817824	3.30E-07
Slc29a3	-0.30693697	0.020657
Tbc1d2b	-0.307155486	3.62E-09
Fmn1	-0.307579164	0.000704
Creb3	-0.308271299	1.65E-05
Plscr3	-0.30869928	0.000137
Frmd4b	-0.309249145	4.84E-10
Bcl9l	-0.309452699	0.014054
Zfp185	-0.309477071	0.004254
Spint1	-0.309805354	1.35E-06
Fndc3b	-0.310107348	1.09E-07
Dag1	-0.310225749	1.22E-08
Sec11c	-0.310334053	9.50E-05
Tenm4	-0.310609814	0.000252
Atp2c1	-0.31063257	5.17E-08
Nfkbiz	-0.310904295	3.22E-06
RP23-128C4.4	-0.311267834	0.026035
Tmprss13	-0.311586389	0.005813
Ttll5	-0.312349296	0.00012
Tmem50b	-0.312495828	0.001587
Bmp2k	-0.312893606	8.94E-07
Lgr4	-0.313262134	2.58E-06
Rnase4	-0.313509692	1.12E-05
Zfp9	-0.313731922	0.00562
Vamp7	-0.314319713	0.041294
mt-Rnr1	-0.315116739	1.06E-06
Aplp2	-0.315683193	2.47E-09
Zdhhc9	-0.316022418	0.009938
Chsy1	-0.316415978	2.60E-07
Cxcl5	-0.316472265	2.55E-06
Tapbpl	-0.317250244	0.002293
Laptm4a	-0.317727549	8.93E-10
Tacc2	-0.317956116	0.000118
Slc35a2	-0.318011239	0.003062
Pxk	-0.318092802	0.000185
Nog	-0.318254721	0.007994

Ell2	-0.318718863	7.71E-05
Klhdc1	-0.318808826	0.047222
Prl2c2	-0.318991518	6.30E-05
Trp53bp2	-0.319065091	0.004033
Reep5	-0.319290584	4.57E-09
Pard6g	-0.319697344	2.19E-08
Ube2h	-0.319850725	8.81E-05
Ttc39c	-0.320059482	7.23E-07
Fgd6	-0.320511675	0.000118
Runx1	-0.320872695	2.89E-06
Pcdhgb6	-0.321362565	0.007366
Atp6v1e1	-0.321799518	1.36E-08
Gpr68	-0.322434437	0.042401
Tubb2a	-0.322926135	3.22E-07
Mrc2	-0.323120879	0.042278
Zhx3	-0.32325563	0.010102
Fabp5	-0.323612981	9.86E-09
Gpr160	-0.323859311	0.044656
Elavl2	-0.324540401	0.045827
Fgfrl1	-0.32460253	0.033705
Fzd2	-0.325794601	0.000467
Slc16a9	-0.326084025	0.001632
Klhl24	-0.326574417	0.000792
Krt6a	-0.326599242	3.01E-12
Map7d1	-0.327048883	3.45E-09
Lxn	-0.327304404	0.000436
Tiparp	-0.327320684	3.30E-07
Sqrdl	-0.327428232	7.36E-05
Las1l	-0.32766078	0.001705
Lonrf1	-0.328569018	0.009055
Grip1	-0.328859442	7.40E-06
Atp6v1g1	-0.32892776	1.51E-07
Ppfibp2	-0.329031413	0.005274
Ahnak2	-0.329177992	0.000868
Ugcg	-0.329246418	4.84E-06
Capn2	-0.329506787	1.59E-14
Hmox1	-0.329895305	3.54E-05
Cd59a	-0.330199762	0.000328
Lgals9	-0.330238822	0.004034
Synrg	-0.330978048	0.0008
Srl	-0.331300949	0.005712
Fam214a	-0.331308087	0.010423
Map4	-0.331933498	5.02E-09
Tmcc1	-0.332094921	0.000308

Mfsd9	-0.332251665	0.013829
Map6	-0.332418075	6.54E-07
Nlgn2	-0.332717531	0.041645
Dsg2	-0.332786265	2.18E-06
St5	-0.332932203	2.37E-07
Aak1	-0.333132024	0.000718
Plec	-0.333581656	3.16E-09
Ak1	-0.334002457	9.18E-08
Bag3	-0.334757934	6.90E-06
Natd1	-0.334891596	0.007423
Atxn1	-0.335646982	4.96E-10
Sec23a	-0.335977012	1.06E-07
Lamp2	-0.337331463	1.38E-09
Shb	-0.33885984	1.56E-06
Sh3bgrl2	-0.339071397	2.44E-08
Prrc1	-0.339325379	0.000253
Slitrk6	-0.340333613	7.14E-06
Samd4b	-0.340457679	0.002697
Megf9	-0.340672632	3.14E-05
Ptpn23	-0.341460058	0.006026
Tnfaip3	-0.341684334	7.20E-11
Gramd2	-0.341988825	6.25E-05
Ptgfrn	-0.34259666	8.37E-16
Pex11g	-0.34261801	0.027427
Jup	-0.34346749	4.67E-13
Cpeb2	-0.344089704	0.000221
Fam210b	-0.344552748	0.000905
Lrp1	-0.345200891	1.30E-11
Itpripl2	-0.345316909	4.74E-16
P4ha2	-0.345516775	1.31E-05
Zfp182	-0.345744657	0.014656
Itgb5	-0.345898251	1.88E-11
Slit3	-0.345946675	0.011346
Blnk	-0.346152644	1.34E-07
Itga6	-0.34633415	4.65E-16
S100a9	-0.346585835	0.000275
Pcdhb22	-0.347613277	0.017986
Rap1gap2	-0.348093633	0.001431
Samd9l	-0.348566308	0.000191
Sh3rf1	-0.348750959	2.69E-05
Cpeb3	-0.349024573	0.038777
Duoxa1	-0.349118366	1.84E-07
Rnf152	-0.349629243	0.022043
Il1rn	-0.350128221	0.001153

Dnmbp	-0.35077974	1.24E-06
Adrb2	-0.35188931	5.53E-06
Atp10d	-0.351914224	2.72E-13
Ngfrap1	-0.352201844	5.66E-07
Fam101b	-0.352384766	5.91E-10
Atp6ap2	-0.352544137	3.08E-08
Msx1	-0.353036872	0.012706
Mvb12b	-0.353150649	0.001544
Sema4c	-0.353212763	6.47E-05
Fgd4	-0.353494986	0.00697
Pcyt1b	-0.354693386	0.001081
Foxj2	-0.354966763	0.004784
Plpp5	-0.355186654	0.000847
Lrrc32	-0.356689499	3.14E-06
Il4ra	-0.35681881	4.69E-10
Klhl18	-0.357359598	0.000555
Wdtc1	-0.357438499	7.61E-05
Calcoco1	-0.357728259	0.023903
Nucb2	-0.358676288	3.28E-05
Abca2	-0.359463367	0.000143
Kdm5b	-0.359896745	4.45E-11
Fam43a	-0.360321426	0.000282
Gpd1l	-0.36066851	0.001758
Map1lc3b	-0.360803592	9.64E-06
Rnf122	-0.362445908	0.001277
Dnajb9	-0.362469399	0.024158
Wnt5a	-0.362553676	7.23E-11
8-Mar	-0.362886741	9.76E-09
Gne	-0.363094033	0.03195
Aph1b	-0.363460416	0.000862
Pfkfb3	-0.3641724	2.03E-07
Dlg5	-0.364181545	1.20E-06
Garem1	-0.365078361	0.004434
Lhx6	-0.365088317	0.016599
Wnk1	-0.365317444	1.34E-09
Klhl7	-0.365334019	1.03E-05
RP23-73N8.4	-0.366151819	0.042728
Pik3r3	-0.36706068	0.000455
Unc13d	-0.367345672	0.000765
Hk1	-0.368066734	1.80E-09
Pitpnm3	-0.368477233	0.000164
Cobl	-0.369934083	4.63E-05
Thnsl1	-0.370821678	0.007423
Ndrg1	-0.371171344	2.00E-07

Camsap3	-0.371347401	0.00288
Trim29	-0.373159034	2.89E-09
Ampd3	-0.373656237	9.27E-10
Ispd	-0.373869851	0.044874
Ptpru	-0.374175436	9.88E-07
Evpl	-0.374450577	8.70E-09
Sh3tc2	-0.374626021	1.34E-05
Wdpcp	-0.374710113	0.012429
Trnp1	-0.374787672	0.010255
Sh3kbp1	-0.375180915	2.31E-14
L3mbtl3	-0.375742806	4.15E-08
Dsg3	-0.376249575	1.93E-17
Serinc2	-0.377137268	3.64E-09
Ap3m2	-0.377689028	0.038551
Tuft1	-0.378107926	7.89E-11
Phlda1	-0.378350358	0.001616
Cpt1a	-0.378930535	7.78E-08
Wipi1	-0.37893474	1.85E-09
Loxl4	-0.379495399	1.82E-05
Cd24a	-0.379513509	1.51E-15
Ifi203	-0.380349629	0.026202
Nt5dc2	-0.381027502	2.57E-09
Gata3	-0.381238886	0.027317
Dmxl1	-0.381406182	1.70E-08
Fyco1	-0.381922591	0.023268
Nipal2	-0.382579599	0.00057
Rab27b	-0.383459536	6.53E-06
Afap1l2	-0.383510378	4.11E-09
Abr	-0.384399083	5.01E-13
Smpd1	-0.384845943	6.49E-05
Fam214b	-0.384879206	6.47E-08
RP24-180L12.6	-0.385161699	0.018752
Cited2	-0.385567353	0.002496
Aes	-0.385861537	7.45E-06
Metrnl	-0.386036155	0.000358
Cldn4	-0.386195031	2.18E-09
Gatm	-0.386631741	8.42E-05
Ulk2	-0.38698906	0.000138
Tspan13	-0.387093368	0.020481
Apol9a	-0.387412077	0.037081
Wisp1	-0.387631614	3.66E-06
Hsdl2	-0.387844497	8.34E-07
Erp44	-0.388107146	7.15E-10
Strbp	-0.389363197	4.17E-12

Tinagl1	-0.389364742	2.24E-10
Ctns	-0.389445801	0.012347
Sox4	-0.389641283	1.93E-18
Trim12c	-0.390465201	0.001095
Kank1	-0.390573046	6.70E-13
Pcdhb7	-0.39151339	0.041428
Ykt6	-0.391878618	2.14E-14
Gas6	-0.39260883	0.002345
Zfp937	-0.393256274	0.008378
Aqp3	-0.393849751	2.41E-07
Ttyh3	-0.394634186	6.33E-07
Dhrs9	-0.394771466	0.006403
Gadd45a	-0.395591618	4.98E-09
C1s1	-0.39601358	0.00158
Dhrs3	-0.396162234	0.030829
Dok2	-0.396560712	0.035714
Alg2	-0.397265693	0.007773
Rnf144b	-0.39742531	5.53E-06
Shpk	-0.399523469	0.048495
Tcf7l2	-0.399794409	0.044967
Sparc	-0.40016722	3.17E-17
Gba2	-0.40051655	0.001558
Trim30a	-0.401322645	0.04754
Rtn4rl1	-0.40189747	0.034401
RP24-528H20.5	-0.403424002	0.038454
Herc3	-0.403508137	0.001632
Pde4dip	-0.403621756	1.61E-08
Naaa	-0.403759552	1.90E-05
Kifc3	-0.404214741	1.10E-05
Mrgpre	-0.404624309	0.034785
Zbp1	-0.404810903	0.043504
Adarb1	-0.405377951	7.02E-12
Pold4	-0.405694742	0.000138
Ret	-0.406006575	0.036027
Amica1	-0.406161447	0.043847
Etl4	-0.407208341	5.02E-09
Orai2	-0.407718254	9.79E-08
Pnpla1	-0.408025733	0.043326
Mcub	-0.408807183	0.0033
Snrk	-0.409103587	2.59E-09
Clic4	-0.409718581	3.93E-20
Bmpr2	-0.410011256	7.41E-10
RP24-90D21.3	-0.410021255	1.74E-06
Bmp1	-0.410860481	1.99E-18

Jag1	-0.411791592	1.28E-20
Cadm4	-0.412666829	3.17E-07
Shank2	-0.413149707	0.039778
Col4a4	-0.413245808	0.039529
Adrb1	-0.413686226	0.000123
Pld1	-0.414142802	0.00039
Gm26888	-0.414279966	0.037191
Fam46a	-0.41491679	0.005001
Dnpep	-0.415516167	5.09E-10
Numbl	-0.41623545	0.006979
Lamc2	-0.416580987	3.77E-16
Bcl3	-0.417271579	0.02416
Neu3	-0.417470248	0.000191
Pard3b	-0.41757403	0.019017
Tlr2	-0.417842437	3.50E-06
Ror2	-0.418206937	0.013013
Rbms3	-0.418861112	0.00725
Saa1	-0.419696534	0.029485
Micall1	-0.420703381	1.19E-15
Cyp2u1	-0.420853133	0.030028
Ddr1	-0.421106375	4.00E-08
Plpp3	-0.422217345	0.001328
Kcnab2	-0.423138416	0.022113
Chst2	-0.423256558	4.04E-08
Zmiz2	-0.423965313	0.005115
Fbxl20	-0.424114716	2.15E-05
Ndfip1	-0.424427477	1.61E-10
Dlc1	-0.424750567	5.28E-05
Plekha2	-0.424902543	3.34E-12
Clca3a2	-0.425581222	0.026631
Map2k6	-0.426368438	0.00206
Ugt1a7c	-0.427117333	0.002614
Casq2	-0.427133508	1.00E-10
Pim1	-0.428049681	0.000555
Kif26a	-0.428480128	9.49E-11
Sprr2h	-0.428639547	0.026191
Tns4	-0.42969601	3.53E-20
Rcan3	-0.429790116	7.80E-06
Dstyk	-0.430893263	3.58E-10
Pcdhb17	-0.431167136	9.50E-05
Dclk1	-0.431286838	7.54E-05
Spsb1	-0.432505812	0.000447
Tceal9	-0.433339382	1.91E-10
Gab3	-0.434299259	0.026354

Papss2	-0.436182814	1.36E-10
Nacc2	-0.43619259	4.84E-10
Krt6b	-0.437068287	8.99E-20
Itgb4	-0.437246544	3.80E-22
Ikbip	-0.437564872	3.30E-06
Kdelr3	-0.438805263	3.41E-06
Cux1	-0.43907873	2.90E-18
Cbx4	-0.439272348	1.08E-09
Plxnd1	-0.440898068	1.17E-06
Spire1	-0.441445275	1.96E-09
RP23-49E21.2	-0.441490199	0.025538
Prag1	-0.441906444	4.28E-08
Foxp1	-0.441971217	1.10E-14
Mxi1	-0.442164693	3.90E-09
Phldb2	-0.442748356	1.62E-22
Ric3	-0.445821526	0.023339
Ccdc68	-0.446878716	0.009735
Zbtb4	-0.446937701	1.30E-06
Ppbp	-0.447288406	1.23E-16
Neurl1a	-0.447907228	2.23E-07
Pdcd4	-0.448582594	0.000664
Mgll	-0.448906113	0.00132
Nhs	-0.449813271	0.001112
Pof1b	-0.451193059	3.92E-07
Hpgds	-0.45185428	0.000955
RP23-230O9.4	-0.452489874	0.012691
Ccser1	-0.454050829	0.008081
Arhgef3	-0.455841954	8.19E-08
Mroh3	-0.456083535	0.008713
Dsc3	-0.456888845	1.34E-28
Mfsd6	-0.457778014	1.80E-13
Rab11fip2	-0.45966426	1.77E-11
Sash1	-0.459799745	4.83E-13
Hoxb9	-0.459864984	0.003039
Nfat5	-0.460140866	9.64E-06
Rap1gap	-0.461406289	0.011511
Creb3l2	-0.464998522	1.87E-07
RP24-332N10.3	-0.465168022	0.008044
Ppp1r12b	-0.465316249	2.50E-07
Ggt5	-0.465327217	0.016846
Prl8a1	-0.466807997	5.78E-12
Mgat4a	-0.466904166	1.02E-07
Orm1	-0.468255771	0.015146
Ttll7	-0.469126124	1.32E-07

Flrt2	-0.470745701	7.43E-21
Smoc2	-0.470767213	0.014799
Fam20a	-0.47102843	2.97E-07
Chdh	-0.473238655	0.01545
Gprc5a	-0.473422553	3.63E-08
Kctd11	-0.474374692	1.66E-08
Kcnh1	-0.474873847	2.03E-07
Sh3tc1	-0.476123508	3.22E-06
Id2	-0.479814576	1.19E-10
Palmd	-0.480922489	0.001419
Itpkb	-0.483051252	5.91E-10
Nav1	-0.484375062	7.05E-11
Dlx1	-0.485390278	0.003254
Sulf1	-0.485955918	3.54E-16
Nebl	-0.486213546	8.08E-06
RP23-44N5.4	-0.486755281	0.008687
Rora	-0.488226852	0.004588
Maged2	-0.489391679	9.47E-14
Lamb3	-0.490070353	1.07E-26
Raph1	-0.490168485	1.84E-06
Mtss1	-0.491577333	5.43E-10
Grhl3	-0.49217342	0.000235
Ripk3	-0.492911843	8.23E-06
Shroom1	-0.492960105	9.84E-06
Mmp9	-0.49554733	0.009055
Fam167a	-0.496142259	5.46E-24
RP24-263P17.1	-0.496158139	0.009386
Wnt11	-0.49620413	0.006107
Fxyd4	-0.498254127	0.008884
Cst6	-0.49827448	1.26E-06
RP23-442L7.4	-0.501574029	0.000101
Fam114a1	-0.502931828	1.22E-09
Vwa7	-0.503080112	0.00033
Pkd1l3	-0.503248087	0.008988
Prl2c3	-0.504096028	3.76E-06
RP23-133N21.3	-0.505037267	0.005221
S100a8	-0.5052839	1.72E-08
Myl7	-0.506224399	0.004585
Narf	-0.511311801	3.76E-09
Celf2	-0.512503494	0.001568
Dok4	-0.513042134	6.41E-06
Dsg1b	-0.514029902	1.39E-08
Wnt9a	-0.514210595	1.68E-15
Fam83c	-0.515345583	0.006299

Sostdc1	-0.516299481	0.00132
Ap5b1	-0.516767461	3.60E-06
Spon2	-0.518023575	7.57E-06
Lce1g	-0.518931491	0.006452
Vdr	-0.5191204	2.05E-11
Tgm2	-0.522503528	9.20E-10
Mras	-0.522971433	0.000458
Dusp1	-0.524212298	3.16E-07
Arid3a	-0.525057404	2.56E-06
Cobll1	-0.52594396	2.77E-09
Col5a1	-0.526916153	0.003772
Klhdc8a	-0.526959712	1.81E-05
Spata13	-0.527531275	3.83E-05
Serpinb1a	-0.530979055	7.30E-05
Grhl1	-0.531497656	7.16E-11
Itgb6	-0.531642991	6.15E-28
Ccng2	-0.532997056	1.42E-05
Skil	-0.53444084	1.22E-27
Trps1	-0.534736404	0.000534
Epb41l3	-0.536917326	3.11E-07
Gse1	-0.536924885	2.02E-06
Dsg1a	-0.537438214	0.000136
Ifi27l2b	-0.537933319	0.004735
Scube3	-0.538154551	0.000372
Tgm3	-0.540427038	3.90E-06
Pkp2	-0.541077947	2.26E-15
Col5a2	-0.541165573	0.001711
Sox9	-0.544357405	4.61E-07
Ids	-0.546243183	2.83E-07
Bhlhe41	-0.547347926	0.000243
Adam10	-0.548638962	1.01E-22
Atp2c2	-0.552066355	6.18E-10
Pcdh1	-0.552573193	0.0002
Paqr9	-0.553072616	0.003464
Phyhipl	-0.553527506	2.17E-07
Cpeb4	-0.554465603	3.12E-10
Mcf2l	-0.555129142	0.001197
Pcdhb19	-0.555638026	0.001234
Parm1	-0.55565045	9.81E-11
Gck	-0.559681229	0.002185
Acta1	-0.559745274	0.000981
Ctsf	-0.55997888	0.002882
Atp9a	-0.563767409	1.46E-15
Epb41l1	-0.564623494	3.64E-11

Lama3	-0.565852211	2.23E-37
Stab1	-0.571225711	7.03E-31
Ifi202b	-0.572568924	1.23E-16
Soat1	-0.576438305	1.93E-37
Hr	-0.576534645	7.85E-24
Megf6	-0.576761395	7.83E-15
Olfm2	-0.577385917	0.000703
Hephl1	-0.577405198	0.000783
Gpr35	-0.577628749	4.72E-05
CAAA01093181.2	-0.57914973	1.83E-05
Pla2g4e	-0.57953598	0.001235
Cmah	-0.579642079	2.17E-06
Bnip3	-0.581035156	7.65E-17
Nuak1	-0.581470766	8.58E-30
Vegfa	-0.581765118	1.50E-12
Adgrf4	-0.581773825	3.50E-09
Gadd45g	-0.583334536	3.04E-08
C3ar1	-0.583705609	0.001667
Ctsw	-0.588226475	0.00138
Inhba	-0.590256807	7.28E-08
Gnpda1	-0.593499862	5.38E-15
Sorcs2	-0.594185499	1.19E-14
Vgll3	-0.594463933	1.52E-17
Cyth1	-0.594627947	9.94E-28
Akap6	-0.595480221	0.000365
Vldlr	-0.596616925	1.24E-17
Ypel5	-0.5993129	2.90E-15
Peg13	-0.599956634	4.84E-09
Ncam1	-0.60103124	9.11E-26
Mxd4	-0.601041645	0.000159
Cpa4	-0.601496642	4.22E-06
Dlx3	-0.602400377	0.001027
Adgrg1	-0.603445113	3.25E-13
Sptbn2	-0.605414255	1.66E-20
Ndrg4	-0.606791231	3.36E-07
Ctla2a	-0.608429483	0.000651
Stmn2	-0.609305747	0.000976
Adamts15	-0.614571574	1.09E-09
Ccbe1	-0.617641954	0.000846
Rab6b	-0.618276567	0.000266
Fam19a5	-0.618476908	0.000651
Id3	-0.621224116	4.09E-18
Slc7a8	-0.621918938	1.98E-08
Tmem140	-0.623301125	4.79E-07

Id1	-0.624976818	3.80E-30
Socs2	-0.628994325	7.85E-08
Hook1	-0.634131824	9.01E-09
Zfp703	-0.635225164	9.16E-06
Mylip	-0.635231884	8.07E-06
Myh14	-0.635680539	1.62E-06
Ahr	-0.637293109	3.05E-17
Pvrl4	-0.637588967	1.38E-22
Pmepa1	-0.638570255	7.43E-41
Gsta3	-0.640542276	3.95E-16
Camkk1	-0.6436207	6.11E-08
Bhlhe40	-0.644564381	6.75E-21
Zmiz1	-0.647565279	7.67E-07
Pcdhb16	-0.648140777	3.57E-13
Mapre2	-0.649225583	1.49E-17
Unc13b	-0.650399418	9.38E-06
Pkdcc	-0.65153292	1.77E-07
Adra1b	-0.656536554	0.000329
Dsc2	-0.659395429	4.45E-23
Trp53inp2	-0.663795407	8.38E-15
Arrb1	-0.664517063	1.24E-07
Fstl1	-0.664748233	1.74E-30
Pxylp1	-0.667658063	1.44E-06
Optn	-0.668891936	3.62E-19
Krt16	-0.670371033	4.42E-30
Slc4a4	-0.672403368	2.47E-05
Apol9b	-0.676429061	0.000178
Trabd2b	-0.676707038	0.000202
Dusp10	-0.67713394	1.22E-29
Timp1	-0.678062257	2.44E-20
Kcnk7	-0.681080807	6.42E-06
Slc43a2	-0.685376182	3.05E-12
Fbln2	-0.690930535	2.12E-55
Tmcc3	-0.692193558	7.55E-32
Ralgps1	-0.69340894	3.46E-06
Rusc2	-0.694657265	9.92E-18
Havcr2	-0.697358832	3.30E-08
Vegfc	-0.702109164	1.87E-11
Sesn3	-0.704577959	4.83E-18
Fam63b	-0.704959764	5.46E-24
Cpn1	-0.705483625	9.25E-06
Thsd1	-0.7058591	3.23E-09
Slc30a1	-0.707501134	2.31E-11
Apol7a	-0.708123319	2.08E-07

Kif26b	-0.712502177	3.93E-12
Sdc3	-0.722871261	7.54E-22
Serpine1	-0.727692039	1.38E-22
Tbx2	-0.731140369	2.26E-06
Mill1	-0.733707422	1.73E-05
Emx2	-0.737026984	1.10E-09
Nat8l	-0.740436791	1.59E-31
Snai3	-0.750964565	1.37E-05
Atp6v0a1	-0.75864267	3.80E-22
Hoxc13	-0.758812866	4.31E-10
Fgf1	-0.761850961	1.81E-05
Ldlrad4	-0.762430315	8.16E-18
Smad6	-0.762442356	1.44E-13
Gjb2	-0.770055033	1.41E-14
Ifitm10	-0.785144549	3.64E-07
Hs3st3a1	-0.796115816	5.38E-12
Ifih1	-0.801810623	7.91E-19
Smad7	-0.802576664	2.05E-35
Tppp	-0.814009151	3.03E-06
Itga2	-0.825252386	4.57E-29
Foxq1	-0.825718957	9.67E-11
Adam12	-0.829636886	1.36E-25
Cd200	-0.834432781	2.12E-14
Ly6d	-0.837834385	4.07E-33
Serpinb8	-0.86234898	5.11E-24
Adcy9	-0.86996471	3.52E-28
Ovol1	-0.872847094	4.38E-18
Slco2a1	-0.875688447	2.53E-09
Maf	-0.898359486	4.79E-30
Pdgfb	-0.905142338	3.22E-12
Tgfbi	-0.905650145	3.30E-51
Prl8a9	-0.915471662	1.48E-81
Bpifc	-0.95851444	1.88E-11
Has2	-0.98448221	2.34E-12
Moxd1	-0.986933432	3.63E-37
Cyp1b1	-1.008340665	2.07E-18
Col5a3	-1.011356556	5.20E-09
Myo7a	-1.014096311	5.40E-15
RP23-121N17.2	-1.022357923	2.58E-19
Slc24a3	-1.063068649	2.23E-30
Smad4	-1.094934585	2.65E-69
Scnn1g	-1.142133194	2.50E-11
Clec2e	-1.185569663	3.21E-21
Prr9	-1.261706538	2.47E-17

Ccl7	-1.304052356	2.19E-21
Nckap5	-1.351112351	8.80E-38
Lgr6	-1.814441152	1.35E-28

Supplementary Table 2. SMAD4 Peak Annotation

Chromsome	start	end	annotation
chr1	4246133	4246341	Distal Intergenic
chr1	4246495	4249452	Distal Intergenic
chr1	7376716	7376953	Intron
chr1	7377063	7379112	Intron
chr1	9627081	9627314	Distal Intergenic
chr1	12738435	12738849	Intron
chr1	16582814	16583686	Intron
chr1	20711841	20712944	Distal Intergenic
chr1	21622778	21623149	Intron
chr1	23562514	23564486	Distal Intergenic
chr1	25161594	25162301	Intron
chr1	35713317	35714046	Distal Intergenic
chr1	36080482	36080837	Distal Intergenic
chr1	36255807	36256006	Intron
chr1	36836819	36837680	Promoter
chr1	37080804	37081969	Distal Intergenic
chr1	37577567	37577982	Intron
chr1	37580272	37581070	Intron
chr1	38895142	38895393	Distal Intergenic
chr1	39812571	39813000	Distal Intergenic
chr1	39813037	39813493	Distal Intergenic
chr1	41043970	41044536	Distal Intergenic
chr1	42754334	42754639	Promoter
chr1	42754944	42755159	Promoter
chr1	42900900	42901299	Distal Intergenic
chr1	43496484	43497171	Distal Intergenic
chr1	46132797	46133000	Intron
chr1	55245076	55246457	Intron
chr1	58168824	58169144	Promoter
chr1	59820305	59820536	Promoter
chr1	64368128	64368389	Distal Intergenic
chr1	70659010	70659415	Intron
chr1	73621506	73621868	Distal Intergenic
chr1	74030713	74030981	Intron
chr1	75457258	75458178	Intron
chr1	75458234	75459162	Intron
chr1	76726575	76726960	Distal Intergenic

chr1	78854856	78855423	Distal Intergenic
chr1	84274080	84274920	Intron
chr1	84321030	84321415	Intron
chr1	92341268	92341589	Distal Intergenic
chr1	92341809	92345349	Distal Intergenic
chr1	98467118	98467317	Distal Intergenic
chr1	102076721	102076928	Intron
chr1	104524683	104524895	Distal Intergenic
chr1	105554706	105554957	Distal Intergenic
chr1	107212948	107213540	Intron
chr1	120651971	120652225	Distal Intergenic
chr1	122526154	122526698	Distal Intergenic
chr1	123380209	123381635	Intron
chr1	125199204	125199403	Distal Intergenic
chr1	125199759	125200653	Distal Intergenic
chr1	125200737	125200953	Distal Intergenic
chr1	125933818	125934140	Intron
chr1	126393264	126393662	Distal Intergenic
chr1	127250690	127251086	Distal Intergenic
chr1	131207591	131207838	Intron
chr1	131207882	131208586	Intron
chr1	131752989	131753371	Intron
chr1	134240279	134240580	Intron
chr1	135491231	135492493	Distal Intergenic
chr1	137100115	137100314	Distal Intergenic
chr1	137100512	137100738	Distal Intergenic
chr1	138233265	138233557	Intron
chr1	138234860	138235150	Intron
chr1	145207298	145207497	Distal Intergenic
chr1	156879427	156879726	Distal Intergenic
chr1	162210056	162210522	Intron
chr1	162402542	162403410	Intron
chr1	166529666	166529920	Distal Intergenic
chr1	168423367	168424247	Intron
chr1	169270428	169270632	Distal Intergenic
chr1	173769391	173769606	Promoter
chr1	179371853	179372315	Promoter
chr1	181816326	181816920	Distal Intergenic
chr1	182280828	182281824	Intron
chr1	183673106	183673982	Intron
chr1	186681483	186681782	Distal Intergenic
chr1	187497622	187498140	Distal Intergenic
chr1	189011019	189011304	Intron
chr1	189227677	189227888	Distal Intergenic

chr1	189566463	189567055	Intron
chr1	190391659	190391983	Intron
chr1	190617002	190618638	Intron
chr1	191548724	191549204	Distal Intergenic
chr1	192307877	192308456	Distal Intergenic
chr1	192579790	192580800	Distal Intergenic
chr1	192695875	192696839	Intron
chr1	192944815	192945031	Distal Intergenic
chr1	194488694	194492753	Intron
chr1	194493590	194493839	Intron
chr1	194495838	194496069	Intron
chr1	194496114	194496342	Intron
chr1	194573123	194574080	Intron
chr1	194574405	194574930	Intron
chr1	194575287	194575486	Intron
chr10	5080268	5080794	Distal Intergenic
chr10	5728902	5729316	Intron
chr10	8681882	8682137	Distal Intergenic
chr10	11072356	11072867	Intron
chr10	13094778	13094990	Intron
chr10	13139988	13140422	Intron
chr10	18429342	18429613	Intron
chr10	19333186	19334249	Distal Intergenic
chr10	20765924	20766285	Intron
chr10	24660419	24661002	Distal Intergenic
chr10	26582412	26582611	Intron
chr10	34071017	34071345	Intron
chr10	36637700	36638064	Distal Intergenic
chr10	40944240	40946324	Intron
chr10	60427304	60427551	Distal Intergenic
chr10	60836629	60836837	Intron
chr10	61051718	61052197	Distal Intergenic
chr10	64088273	64088687	Intron
chr10	64124592	64124885	Intron
chr10	67166735	67167028	Distal Intergenic
chr10	69686126	69686445	Distal Intergenic
chr10	72634432	72634728	Intron
chr10	77071641	77071931	Intron
chr10	79819758	79820201	Promoter
chr10	79823324	79826052	Exon
chr10	79826842	79827323	Exon
chr10	80869511	80869771	Intron
chr10	82574181	82574520	Intron
chr10	83662255	83662761	Distal Intergenic

chr10	83860932	83861294	Intron
chr10	86930534	86930778	Distal Intergenic
chr10	86962070	86962593	Distal Intergenic
chr10	94036250	94036950	Intron
chr10	94458355	94458659	Distal Intergenic
chr10	97009518	97010562	Distal Intergenic
chr10	98632851	98634226	Intron
chr10	100400128	100400605	Distal Intergenic
chr10	104403035	104403457	Distal Intergenic
chr10	106840473	106840938	Intron
chr10	106874701	106875097	Distal Intergenic
chr10	109625439	109626210	Distal Intergenic
chr10	114931306	114931853	Intron
chr10	115555961	115556754	Intron
chr10	115749042	115749422	Intron
chr10	116190104	116190978	Distal Intergenic
chr10	116226454	116226814	Intron
chr10	116314917	116317015	Intron
chr10	118388695	118389650	Distal Intergenic
chr10	118389686	118389885	Distal Intergenic
chr10	118591740	118592400	Distal Intergenic
chr10	120437370	120437716	Distal Intergenic
chr10	122484243	122485253	Intron
chr10	125343542	125343940	Distal Intergenic
chr10	125356306	125356732	Distal Intergenic
chr10	125532080	125532487	Distal Intergenic
chr10	125599704	125600002	Distal Intergenic
chr11	3046896	3047101	Intron
chr11	3474829	3475066	Distal Intergenic
chr11	4921138	4921458	Intron
chr11	6565255	6565454	Intron
chr11	7005969	7006432	Intron
chr11	7399153	7399742	Distal Intergenic
chr11	19489431	19490749	Distal Intergenic
chr11	24169587	24170262	Distal Intergenic
chr11	24170605	24170849	Distal Intergenic
chr11	26083656	26083867	Intron
chr11	31972482	31972811	Distal Intergenic
chr11	32448513	32448891	Intron
chr11	35817936	35818555	Downstream
chr11	35842691	35842936	Intron
chr11	44105603	44107644	Distal Intergenic
chr11	45330842	45331716	Distal Intergenic
chr11	50232832	50233054	Intron

chr11	50647845	50648061	Intron
chr11	52527238	52527870	Distal Intergenic
chr11	53067029	53067622	Distal Intergenic
chr11	57285216	57286531	Distal Intergenic
chr11	58207212	58207499	Distal Intergenic
chr11	68296230	68296651	Intron
chr11	70423196	70423530	Exon
chr11	77463247	77463912	Distal Intergenic
chr11	78945117	78945459	Intron
chr11	79695739	79696157	Distal Intergenic
chr11	83368404	83369841	Distal Intergenic
chr11	86201935	86202134	Distal Intergenic
chr11	86287458	86287784	Distal Intergenic
chr11	88942905	88943418	Intron
chr11	91439251	91439677	Promoter
chr11	97810679	97810938	Intron
chr11	99122950	99123857	Distal Intergenic
chr11	99268939	99269222	Distal Intergenic
chr11	102875060	102875983	Intron
chr11	104106408	104106664	Intron
chr11	104888866	104889190	Distal Intergenic
chr11	104948880	104949316	Intron
chr11	107519612	107519818	Intron
chr11	108123563	108123852	Intron
chr11	108124061	108124260	Intron
chr11	108872983	108873329	Distal Intergenic
chr11	109847216	109847416	Intron
chr11	109847795	109848355	Intron
chr11	110314851	110315334	Intron
chr11	111742343	111743410	Distal Intergenic
chr11	111743451	111743679	Distal Intergenic
chr11	113741149	113741685	Intron
chr11	114253817	114254063	Distal Intergenic
chr11	118052364	118052864	Intron
chr11	118796844	118797263	Distal Intergenic
chr11	119177134	119177862	Intron
chr11	119489900	119490360	Intron
chr11	119720472	119720980	Intron
chr12	3320886	3321515	Distal Intergenic
chr12	4110845	4112322	Distal Intergenic
chr12	4292179	4293011	Intron
chr12	4536822	4538116	Distal Intergenic
chr12	12722791	12722990	Distal Intergenic
chr12	12724455	12724660	Distal Intergenic

chr12	14665985	14666879	Distal Intergenic
chr12	18136385	18136690	Distal Intergenic
chr12	18140466	18140984	Distal Intergenic
chr12	19178730	19179638	Distal Intergenic
chr12	25173052	25173699	Distal Intergenic
chr12	35578986	35579400	Intron
chr12	54027695	54033510	Intron
chr12	57163120	57163408	Distal Intergenic
chr12	63278293	63278503	Distal Intergenic
chr12	70755068	70755323	Intron
chr12	78269503	78270270	Distal Intergenic
chr12	79451900	79452115	Intron
chr12	84297056	84297595	Distal Intergenic
chr12	89420941	89421195	Promoter
chr12	92493430	92493629	Intron
chr12	92740705	92741259	Intron
chr12	100883366	100883585	Distal Intergenic
chr12	104542378	104542577	Distal Intergenic
chr12	107197512	107197959	Distal Intergenic
chr12	109284211	109285130	Distal Intergenic
chr12	109285173	109285394	Distal Intergenic
chr12	110032024	110032308	Promoter
chr12	111095284	111095549	Distal Intergenic
chr12	111257645	111257844	Intron
chr12	111484198	111484488	Intron
chr12	114174409	114174753	Intron
chr12	114175010	114175509	Intron
chr12	114499767	114500091	Intron
chr13	3963446	3964770	Distal Intergenic
chr13	5346202	5346781	Distal Intergenic
chr13	9833540	9833758	Distal Intergenic
chr13	12051846	12052592	Intron
chr13	14649864	14650185	Distal Intergenic
chr13	27863546	27863818	Downstream
chr13	35130109	35131008	Distal Intergenic
chr13	37726846	37727418	Distal Intergenic
chr13	38429731	38430960	Distal Intergenic
chr13	40765804	40766012	Distal Intergenic
chr13	42190048	42190670	Intron
chr13	44964953	44965380	Intron
chr13	45885192	45885695	Intron
chr13	48370330	48371420	Distal Intergenic
chr13	48529272	48529485	Distal Intergenic
chr13	52752597	52752804	Distal Intergenic

chr13	53832617	53833199	Distal Intergenic
chr13	53837139	53838423	Distal Intergenic
chr13	56332087	56332548	Distal Intergenic
chr13	58080778	58081019	Distal Intergenic
chr13	60198250	60198948	Distal Intergenic
chr13	64596590	64597334	Intron
chr13	69673304	69673586	Promoter
chr13	70631358	70632479	Distal Intergenic
chr13	72931753	72932320	Distal Intergenic
chr13	74665814	74668431	Distal Intergenic
chr13	75353129	75353845	Distal Intergenic
chr13	83181027	83181390	Distal Intergenic
chr13	90516622	90517613	Distal Intergenic
chr13	93699488	93699691	Distal Intergenic
chr13	97960480	97960700	Intron
chr13	100560885	100561150	Distal Intergenic
chr13	105012564	105012778	Intron
chr13	108679396	108679662	Promoter
chr13	109503753	109504285	Intron
chr13	111435405	111436395	Distal Intergenic
chr13	112888463	112889055	Distal Intergenic
chr13	113368277	113368612	Intron
chr13	114521938	114522311	Distal Intergenic
chr14	19877142	19877346	Distal Intergenic
chr14	19877460	19877821	Distal Intergenic
chr14	19877993	19878290	Distal Intergenic
chr14	19878383	19878981	Distal Intergenic
chr14	19879307	19879925	Distal Intergenic
chr14	19880657	19880887	Distal Intergenic
chr14	19880993	19881580	Distal Intergenic
chr14	19881888	19884248	Distal Intergenic
chr14	19884367	19889384	Distal Intergenic
chr14	19889527	19890402	Distal Intergenic
chr14	19890524	19891693	Distal Intergenic
chr14	23764573	23764784	Intron
chr14	25836134	25836334	Distal Intergenic
chr14	27700796	27701034	Intron
chr14	30664481	30664692	Distal Intergenic
chr14	47492729	47493114	Distal Intergenic
chr14	51795014	51795311	Distal Intergenic
chr14	51795441	51795644	Distal Intergenic
chr14	51886686	51886979	Distal Intergenic
chr14	57312720	57312964	Intron
chr14	65109695	65110324	Distal Intergenic

chr14	70331913	70332146	Intron
chr14	72748463	72748759	Distal Intergenic
chr14	75789466	75789725	Distal Intergenic
chr14	75789811	75790010	Distal Intergenic
chr14	86921244	86922607	Distal Intergenic
chr14	88292341	88292602	Distal Intergenic
chr14	99866950	99867627	Distal Intergenic
chr14	103863347	103864010	Intron
chr14	105114979	105115247	Intron
chr14	115607250	115607663	Intron
chr14	118869324	118869749	Distal Intergenic
chr15	5711120	5711356	Distal Intergenic
chr15	6477872	6478628	Distal Intergenic
chr15	6478793	6479465	Distal Intergenic
chr15	6479503	6479712	Distal Intergenic
chr15	6479836	6480962	Distal Intergenic
chr15	6481078	6481777	Distal Intergenic
chr15	6481843	6482159	Distal Intergenic
chr15	6482195	6482876	Distal Intergenic
chr15	6482934	6483617	Distal Intergenic
chr15	6822713	6823231	Intron
chr15	7256989	7257208	Intron
chr15	7693210	7693455	Distal Intergenic
chr15	10509587	10510060	Intron
chr15	10510301	10511760	Intron
chr15	13727492	13727902	Distal Intergenic
chr15	13728054	13728266	Distal Intergenic
chr15	13728566	13728814	Distal Intergenic
chr15	20574659	20575512	Intron
chr15	31017414	31017684	Distal Intergenic
chr15	38806129	38806374	Distal Intergenic
chr15	38806430	38806787	Distal Intergenic
chr15	39581196	39581556	Intron
chr15	47371465	47371664	Distal Intergenic
chr15	55633031	55633258	Intron
chr15	64870510	64870791	Distal Intergenic
chr15	66135297	66135927	Distal Intergenic
chr15	72352984	72354800	Intron
chr15	74170304	74170508	Distal Intergenic
chr15	74170682	74171273	Distal Intergenic
chr15	74916995	74917372	Distal Intergenic
chr15	75563641	75564374	Distal Intergenic
chr15	76831457	76833693	Distal Intergenic
chr15	83567590	83569670	Distal Intergenic

chr15	83572559	83573050	Distal Intergenic
chr15	83867791	83871472	Intron
chr15	86025212	86025681	Distal Intergenic
chr15	86234248	86234463	Intron
chr15	86253199	86253527	Intron
chr15	86583395	86584563	Distal Intergenic
chr15	89919550	89920064	Distal Intergenic
chr15	93842848	93843884	Distal Intergenic
chr15	96548526	96548807	Distal Intergenic
chr15	98220757	98220962	Distal Intergenic
chr15	98330941	98332140	Distal Intergenic
chr15	100584942	100585255	Distal Intergenic
chr15	100697216	100698126	Distal Intergenic
chr15	101299687	101300432	Intron
chr15	101314562	101315114	Intron
chr16	5246396	5247075	Intron
chr16	9464191	9464450	Distal Intergenic
chr16	9464791	9466789	Distal Intergenic
chr16	11144018	11144404	Intron
chr16	11656258	11657419	Exon
chr16	17222381	17222588	Distal Intergenic
chr16	17560388	17560676	Promoter
chr16	17972863	17973330	Distal Intergenic
chr16	18225354	18225557	Intron
chr16	18533189	18533514	Intron
chr16	21487698	21487949	Intron
chr16	22830876	22831114	Intron
chr16	24351437	24352248	Distal Intergenic
chr16	26496733	26499781	Distal Intergenic
chr16	26992935	26994310	Distal Intergenic
chr16	30717547	30718052	Distal Intergenic
chr16	30718116	30718324	Distal Intergenic
chr16	30718573	30718899	Distal Intergenic
chr16	31026165	31027170	Intron
chr16	31162392	31163867	Intron
chr16	32211426	32212537	Distal Intergenic
chr16	33018547	33018806	Intron
chr16	35580733	35581297	Intron
chr16	35981705	35981992	Exon
chr16	45352533	45353413	Distal Intergenic
chr16	45840381	45840580	Intron
chr16	55852037	55852642	Intron
chr16	55854447	55855413	Intron
chr16	56230603	56230852	Intron

chr16	57256410	57256811	Distal Intergenic
chr16	57391290	57391809	Intron
chr16	60947345	60948340	Distal Intergenic
chr16	62833022	62833299	Intron
chr16	64851188	64851397	Promoter
chr16	64851493	64851766	Promoter
chr16	64852130	64852366	Promoter
chr16	65034288	65035426	Intron
chr16	72644429	72645027	Distal Intergenic
chr16	78252723	78253506	Intron
chr16	78660552	78660761	Distal Intergenic
chr16	78660851	78661218	Distal Intergenic
chr16	80491400	80491793	Distal Intergenic
chr16	86000514	86001505	Distal Intergenic
chr16	86263636	86263835	Distal Intergenic
chr16	86820998	86821470	Distal Intergenic
chr16	87165374	87165730	Distal Intergenic
chr16	87543664	87544692	Distal Intergenic
chr16	88762870	88763201	Distal Intergenic
chr16	90178068	90178268	Distal Intergenic
chr16	90913629	90914109	Distal Intergenic
chr16	91438178	91439404	Distal Intergenic
chr16	91439455	91439685	Distal Intergenic
chr16	92250683	92251792	Distal Intergenic
chr16	92323332	92324604	Distal Intergenic
chr16	92655503	92655928	Intron
chr16	92807057	92807344	Intron
chr16	92954834	92955776	Distal Intergenic
chr16	93110075	93110316	Distal Intergenic
chr16	93772185	93772960	Intron
chr16	93773171	93773396	Intron
chr16	93913334	93913699	Distal Intergenic
chr16	94017187	94017425	Distal Intergenic
chr16	94953822	94954079	Distal Intergenic
chr16	94959817	94960038	Distal Intergenic
chr16	94960101	94960473	Distal Intergenic
chr16	95959364	95960010	Distal Intergenic
chr16	96676254	96676633	Intron
chr16	97124847	97125199	Intron
chr17	3059596	3060111	Distal Intergenic
chr17	3060166	3060862	Distal Intergenic
chr17	3061122	3061321	Downstream
chr17	3061650	3062141	Downstream
chr17	3070003	3070206	Intron

chr17	3070344	3073873	Intron
chr17	3074004	3074594	Intron
chr17	5241033	5241232	Intron
chr17	5863896	5867255	Intron
chr17	5879519	5884978	Intron
chr17	10139933	10140132	Distal Intergenic
chr17	10918674	10919179	Intron
chr17	12482261	12483400	Intron
chr17	12829073	12829378	Distal Intergenic
chr17	13498714	13499273	Intron
chr17	13743462	13746166	Intron
chr17	16047782	16047981	Distal Intergenic
chr17	16154152	16154415	Distal Intergenic
chr17	24132294	24132493	Intron
chr17	24479619	24480080	Intron
chr17	25484556	25485442	Distal Intergenic
chr17	27573430	27573733	Intron
chr17	28405166	28405389	Intron
chr17	28495634	28495962	Promoter
chr17	30220335	30220726	Intron
chr17	30822013	30822421	Intron
chr17	36368215	36368524	Distal Intergenic
chr17	38087232	38087442	Downstream
chr17	39980003	39985754	Promoter
chr17	43440990	43441408	Intron
chr17	46237065	46237916	Distal Intergenic
chr17	46472223	46472426	Distal Intergenic
chr17	46616337	46616590	Intron
chr17	50056138	50056567	Distal Intergenic
chr17	50060572	50060857	Distal Intergenic
chr17	50064345	50064913	Distal Intergenic
chr17	50064966	50066171	Distal Intergenic
chr17	50066212	50066713	Distal Intergenic
chr17	50484979	50485424	Distal Intergenic
chr17	50668259	50669375	Intron
chr17	50669431	50669658	Intron
chr17	56794503	56794788	Exon
chr17	63848621	63849088	Promoter
chr17	64184199	64184867	Distal Intergenic
chr17	67574888	67575089	Intron
chr17	68939805	68940812	Intron
chr17	69905748	69905966	Distal Intergenic
chr17	71643413	71643730	Intron
chr17	73703619	73703931	Intron

chr17	75522774	75523499	Intron
chr17	81226807	81227226	Distal Intergenic
chr17	85346380	85346579	Intron
chr17	87296656	87296873	Distal Intergenic
chr17	87411767	87413651	Intron
chr17	87696449	87696648	Intron
chr17	87899638	87899841	Distal Intergenic
chr17	87899913	87900177	Distal Intergenic
chr17	88701590	88701933	Intron
chr17	89148283	89148898	Intron
chr17_random	323668	326663	Distal Intergenic
chr17_random	339011	339978	Distal Intergenic
chr18	3661334	3661685	Distal Intergenic
chr18	4502923	4504993	Distal Intergenic
chr18	5788138	5790995	Distal Intergenic
chr18	6621020	6621904	Intron
chr18	6787571	6787963	Intron
chr18	7608412	7609737	Promoter
chr18	10548269	10548604	Intron
chr18	11636868	11637785	Distal Intergenic
chr18	12050742	12051790	Intron
chr18	12878602	12879073	Intron
chr18	14080236	14080892	Intron
chr18	20747611	20748293	Intron
chr18	28243445	28243757	Distal Intergenic
chr18	30588483	30589029	Distal Intergenic
chr18	37942419	37942871	Intron
chr18	41301131	41301462	Distal Intergenic
chr18	42744421	42745143	Distal Intergenic
chr18	47578003	47578318	Distal Intergenic
chr18	54858619	54858967	Distal Intergenic
chr18	55025645	55025881	Distal Intergenic
chr18	60798392	60798650	Distal Intergenic
chr18	62468618	62469266	Distal Intergenic
chr18	67286364	67286658	Intron
chr18	68132506	68132732	Intron
chr18	68851650	68851947	Distal Intergenic
chr18	72066685	72067163	Intron
chr18	76522184	76522947	Distal Intergenic
chr18	76840394	76841569	Distal Intergenic
chr18	76843440	76843642	Distal Intergenic
chr18	77769574	77769796	Intron
chr18	80529555	80530239	Intron
chr18	81361081	81361435	Distal Intergenic

chr18	81600792	81601341	Distal Intergenic
chr18	81865113	81866078	Distal Intergenic
chr18	82137905	82138104	Distal Intergenic
chr18	82841434	82841940	Intron
chr18	83710464	83711748	Distal Intergenic
chr18	85867357	85867646	Distal Intergenic
chr19	4306479	4306708	Promoter
chr19	5241429	5241892	Intron
chr19	6558628	6558918	Distal Intergenic
chr19	10582454	10584474	Intron
chr19	10746685	10747876	Intron
chr19	11874599	11875330	Intron
chr19	12091591	12091938	Distal Intergenic
chr19	17119948	17120337	Intron
chr19	24932108	24932927	Intron
chr19	25780817	25781691	Distal Intergenic
chr19	29291228	29291498	Distal Intergenic
chr19	29395024	29395286	Downstream
chr19	29552191	29552395	Intron
chr19	35741006	35741205	Distal Intergenic
chr19	35963523	35963740	Intron
chr19	38784777	38785223	Intron
chr19	39064787	39066240	Distal Intergenic
chr19	42339832	42340400	Intron
chr19	42773453	42773814	Distal Intergenic
chr19	42773937	42774439	Distal Intergenic
chr19	46095432	46095950	Distal Intergenic
chr19	47282939	47283216	Intron
chr19	47283274	47283854	Intron
chr19	47708405	47709324	Intron
chr19	48125981	48126323	Distal Intergenic
chr19	54093203	54094089	Intron
chr19	56294289	56294492	Distal Intergenic
chr19	56922084	56922791	Intron
chr19	58004305	58004665	Intron
chr19	58358014	58358694	Intron
chr19	61340160	61340422	Distal Intergenic
chr1_random	1033047	1033329	Intron
chr2	3069508	3069935	Intron
chr2	3881799	3885591	Distal Intergenic
chr2	4442775	4443540	Intron
chr2	5004351	5004761	Distal Intergenic
chr2	5300122	5300422	Intron
chr2	6117991	6118287	Intron

chr2	8631081	8631312	Distal Intergenic
chr2	9062161	9062527	Distal Intergenic
chr2	9719135	9719859	Distal Intergenic
chr2	20821481	20821788	Intron
chr2	22600369	22600625	Distal Intergenic
chr2	24674169	24674403	Intron
chr2	25952075	25952600	Intron
chr2	26919346	26919545	Intron
chr2	26919585	26921107	Intron
chr2	28040518	28041001	Distal Intergenic
chr2	28227484	28227700	Distal Intergenic
chr2	28262860	28263069	Distal Intergenic
chr2	28458107	28458909	Distal Intergenic
chr2	29071003	29072248	Intron
chr2	29072462	29072853	Intron
chr2	29427098	29427297	Distal Intergenic
chr2	29449039	29449872	Distal Intergenic
chr2	31221247	31221490	Distal Intergenic
chr2	38510765	38511967	Distal Intergenic
chr2	44245727	44246365	Distal Intergenic
chr2	44246772	44247020	Distal Intergenic
chr2	45275575	45276879	Distal Intergenic
chr2	45785701	45786593	Distal Intergenic
chr2	49463287	49464202	Distal Intergenic
chr2	49651010	49652251	Intron
chr2	50214498	50214912	Intron
chr2	52887913	52888228	Intron
chr2	53868242	53868700	Distal Intergenic
chr2	54567653	54568051	Intron
chr2	58278367	58278885	Distal Intergenic
chr2	59200562	59200761	Distal Intergenic
chr2	59200840	59201091	Distal Intergenic
chr2	66250049	66250462	Intron
chr2	69211671	69213156	Distal Intergenic
chr2	70244333	70245113	Intron
chr2	76876932	76877889	Exon
chr2	79019472	79021361	Distal Intergenic
chr2	93367857	93368553	Distal Intergenic
chr2	94396476	94398141	Distal Intergenic
chr2	98506877	98507304	Distal Intergenic
chr2	102743138	102744257	Distal Intergenic
chr2	102979719	102980534	Distal Intergenic
chr2	105015001	105015279	Distal Intergenic
chr2	105098010	105098870	Intron

chr2	105098909	105099201	Intron
chr2	112758052	112758313	Intron
chr2	117032975	117033208	Distal Intergenic
chr2	122243566	122243974	Distal Intergenic
chr2	124683455	124683679	Distal Intergenic
chr2	124687603	124687946	Distal Intergenic
chr2	126902864	126903279	Intron
chr2	127280553	127281057	Downstream
chr2	127339651	127339850	Intron
chr2	127489324	127490600	Distal Intergenic
chr2	128737746	128738048	Distal Intergenic
chr2	131567889	131568342	Distal Intergenic
chr2	131990290	131990588	Distal Intergenic
chr2	143629794	143630172	Intron
chr2	143819795	143820425	Intron
chr2	148174513	148175021	Distal Intergenic
chr2	150725303	150725883	Intron
chr2	152849118	152849441	Distal Intergenic
chr2	152919844	152922149	Distal Intergenic
chr2	152922196	152926160	Distal Intergenic
chr2	153661562	153661761	Distal Intergenic
chr2	155205045	155205285	Distal Intergenic
chr2	155477984	155478455	Intron
chr2	158045246	158045554	Intron
chr2	158057563	158057961	Distal Intergenic
chr2	160366766	160367775	Distal Intergenic
chr2	160430996	160432519	Distal Intergenic
chr2	161387561	161388698	Intron
chr2	164489183	164489709	Intron
chr2	164530283	164531113	Distal Intergenic
chr2	167115352	167116465	Distal Intergenic
chr2	167514522	167514772	Promoter
chr2	167744418	167745014	Distal Intergenic
chr2	167745144	167745546	Distal Intergenic
chr2	167745594	167745856	Distal Intergenic
chr2	168187324	168187773	Distal Intergenic
chr2	168235880	168236283	Distal Intergenic
chr2	168422532	168422959	Intron
chr2	169016116	169016356	Distal Intergenic
chr2	169248086	169248306	Distal Intergenic
chr2	169392388	169393476	Distal Intergenic
chr2	170584588	170584865	Intron
chr2	171640211	171641473	Distal Intergenic
chr2	171724689	171725075	Distal Intergenic

chr2	172027442	172027664	Distal Intergenic
chr2	172153263	172153520	Distal Intergenic
chr2	172420452	172421232	Distal Intergenic
chr2	172421470	172421669	Distal Intergenic
chr2	172578925	172579204	Distal Intergenic
chr2	172579277	172580130	Distal Intergenic
chr2	172580172	172580372	Distal Intergenic
chr2	172626719	172626930	Distal Intergenic
chr2	172627799	172628498	Distal Intergenic
chr2	172924080	172926596	Intron
chr2	172953115	172953728	Intron
chr2	172953766	172954113	Intron
chr2	173083440	173083639	Intron
chr2	173100634	173100868	Intron
chr2	173100904	173101227	Promoter
chr2	173219539	173219816	Distal Intergenic
chr2	178497690	178498308	Distal Intergenic
chr2	178737495	178738428	Distal Intergenic
chr2	179642303	179642608	Distal Intergenic
chr2	179711462	179711898	Promoter
chr2	181665342	181665675	Distal Intergenic
chr3	5860388	5860796	Distal Intergenic
chr3	9501623	9502137	Intron
chr3	14109889	14110307	Intron
chr3	26623908	26624600	Intron
chr3	27278494	27278858	Distal Intergenic
chr3	30977928	30978548	Distal Intergenic
chr3	30993239	30994402	Promoter
chr3	30994474	30994681	Promoter
chr3	31110553	31111125	Intron
chr3	31585143	31585342	Distal Intergenic
chr3	48189233	48189956	Distal Intergenic
chr3	48190034	48190494	Distal Intergenic
chr3	50617673	50618155	Distal Intergenic
chr3	51574866	51575454	Intron
chr3	53305404	53306294	Intron
chr3	53324589	53324791	Intron
chr3	53487107	53487394	Distal Intergenic
chr3	54400245	54400722	Distal Intergenic
chr3	58055630	58057394	Distal Intergenic
chr3	58278156	58279062	Distal Intergenic
chr3	59848977	59849965	Distal Intergenic
chr3	60539979	60540287	Distal Intergenic
chr3	61963071	61963311	Distal Intergenic

chr3	63975878	63976094	Distal Intergenic
chr3	64218195	64218546	Distal Intergenic
chr3	66497642	66497937	Distal Intergenic
chr3	68550260	68550573	Distal Intergenic
chr3	69819042	69819403	Intron
chr3	87554297	87554892	Intron
chr3	93008659	93008932	Intron
chr3	93017243	93017749	Exon
chr3	93018250	93018475	Exon
chr3	96042975	96043386	Promoter
chr3	97266093	97266609	Distal Intergenic
chr3	97755300	97756113	Intron
chr3	98967165	98967442	Intron
chr3	108349429	108349952	Distal Intergenic
chr3	116762269	116762604	Distal Intergenic
chr3	120074747	120074963	Distal Intergenic
chr3	120885377	120885797	Distal Intergenic
chr3	120952719	120952930	Intron
chr3	132868307	132868596	Intron
chr3	133014603	133015611	Intron
chr3	133015752	133016446	Intron
chr3	135239930	135240841	Distal Intergenic
chr3	142128097	142128405	Distal Intergenic
chr3	144261652	144261995	Distal Intergenic
chr3	144712250	144712738	Distal Intergenic
chr3	145015083	145015473	Intron
chr3	145617222	145617524	Distal Intergenic
chr3	146222946	146223388	Distal Intergenic
chr3	148715473	148715834	Distal Intergenic
chr3	150426958	150427220	Distal Intergenic
chr3	151792063	151792411	Intron
chr3	152312647	152312951	Intron
chr3	152508188	152508502	Intron
chr3	153287103	153288120	Intron
chr3	154271686	154272403	Intron
chr3	154272625	154273013	Intron
chr4	3154863	3155990	Distal Intergenic
chr4	3348692	3348928	Distal Intergenic
chr4	3348967	3350758	Distal Intergenic
chr4	10844506	10845353	Distal Intergenic
chr4	12285028	12285846	Distal Intergenic
chr4	16010415	16010696	Distal Intergenic
chr4	16010887	16011776	Distal Intergenic
chr4	20118996	20119199	Intron

chr4	32366935	32367235	Intron
chr4	36780589	36781379	Intron
chr4	40303178	40304050	Distal Intergenic
chr4	40481270	40481939	Distal Intergenic
chr4	54113785	54114271	Distal Intergenic
chr4	56703625	56704366	Distal Intergenic
chr4	56705796	56706428	Distal Intergenic
chr4	56855619	56855818	Intron
chr4	58587322	58587970	Distal Intergenic
chr4	58588229	58588474	Distal Intergenic
chr4	60093765	60094475	Intron
chr4	60163228	60163789	Intron
chr4	60445480	60445917	Intron
chr4	60900114	60900316	Intron
chr4	61108383	61108822	Intron
chr4	61264467	61264884	Intron
chr4	61453657	61454317	Distal Intergenic
chr4	61914333	61914694	Distal Intergenic
chr4	62920347	62920546	Intron
chr4	80975436	80976436	Intron
chr4	104552858	104553688	Distal Intergenic
chr4	106781089	106782256	Distal Intergenic
chr4	118076268	118077278	Intron
chr4	118228153	118229463	Intron
chr4	118229634	118229959	Intron
chr4	119513830	119514664	Intron
chr4	119892853	119893199	Distal Intergenic
chr4	122132473	122132672	Distal Intergenic
chr4	124056066	124056342	Distal Intergenic
chr4	128063281	128063484	Intron
chr4	128430368	128430584	Distal Intergenic
chr4	128542311	128543002	Distal Intergenic
chr4	128721091	128721406	Distal Intergenic
chr4	130551172	130551752	Distal Intergenic
chr4	130683554	130683753	Distal Intergenic
chr4	131844972	131845197	Intron
chr4	132498083	132499082	Distal Intergenic
chr4	133309163	133309405	Promoter
chr4	135364615	135364899	Exon
chr4	136929688	136929938	Distal Intergenic
chr4	136930520	136931108	Distal Intergenic
chr4	139831644	139831843	Distal Intergenic
chr4	139831892	139832100	Distal Intergenic
chr4	141544105	141544305	Intron

chr4	142157576	142158279	Distal Intergenic
chr4	142158431	142158649	Distal Intergenic
chr4	142225366	142225694	Intron
chr4	146777742	146777941	Intron
chr4	147761622	147761863	Distal Intergenic
chr4	148996952	148997261	Intron
chr4	150018962	150019290	Intron
chr4	150905933	150906176	Intron
chr4	151662894	151663856	Intron
chr4	151817716	151818592	Intron
chr4	152045140	152045667	Distal Intergenic
chr4	152492726	152492969	Distal Intergenic
chr4	152493005	152493204	Distal Intergenic
chr4	152494187	152494579	Distal Intergenic
chr4	152882710	152882984	Distal Intergenic
chr4	152883346	152883556	Distal Intergenic
chr4	153440631	153440839	Intron
chr4	154427085	154427298	Distal Intergenic
chr4	155375642	155375887	Intron
chr5	9381303	9381563	Intron
chr5	13089854	13090830	Distal Intergenic
chr5	20774226	20774466	Intron
chr5	20774528	20774836	Intron
chr5	21412483	21412920	Intron
chr5	22001289	22001548	Intron
chr5	23318070	23318269	Intron
chr5	23318371	23318576	Intron
chr5	23318717	23319058	Intron
chr5	24161953	24163273	Distal Intergenic
chr5	24221785	24222109	Intron
chr5	25321803	25322113	Intron
chr5	25322211	25322442	Intron
chr5	25322899	25324256	Intron
chr5	27079893	27080103	Distal Intergenic
chr5	28051245	28051718	Intron
chr5	28052372	28052790	3' UTR
chr5	28423709	28424008	Distal Intergenic
chr5	28424161	28426693	Distal Intergenic
chr5	30796501	30797088	Intron
chr5	36301660	36302539	Intron
chr5	36623676	36624116	Intron
chr5	39742037	39742299	Distal Intergenic
chr5	43625949	43626148	Promoter
chr5	44084986	44085241	Intron

chr5	50566048	50566399	Distal Intergenic
chr5	63243318	63243613	Intron
chr5	64735168	64735747	Intron
chr5	65435679	65436611	Distal Intergenic
chr5	67818485	67818766	Promoter
chr5	75560157	75560377	Intron
chr5	77187050	77187746	Distal Intergenic
chr5	86855222	86856208	Intron
chr5	91402205	91402488	Intron
chr5	92223279	92223960	Distal Intergenic
chr5	96830579	96832012	Intron
chr5	101639006	101639780	Distal Intergenic
chr5	101845056	101845374	Distal Intergenic
chr5	101845810	101846532	Distal Intergenic
chr5	102685482	102685863	Intron
chr5	104759722	104761314	Intron
chr5	107237204	107237640	Distal Intergenic
chr5	107815378	107815917	3' UTR
chr5	111007090	111007362	Intron
chr5	111869765	111870259	Intron
chr5	111987710	111988522	Distal Intergenic
chr5	112004520	112005510	Distal Intergenic
chr5	112121697	112122224	Distal Intergenic
chr5	113025004	113025526	Distal Intergenic
chr5	113199443	113200349	Intron
chr5	113451140	113451506	Distal Intergenic
chr5	113601928	113602148	Distal Intergenic
chr5	116555674	116555893	Distal Intergenic
chr5	116612913	116613531	Exon
chr5	116649550	116649751	Intron
chr5	117476469	117476812	Distal Intergenic
chr5	118739894	118740148	Distal Intergenic
chr5	118767038	118767528	Distal Intergenic
chr5	118773675	118774541	Distal Intergenic
chr5	119441695	119444450	Distal Intergenic
chr5	119856945	119857283	Distal Intergenic
chr5	120060206	120060557	Intron
chr5	120060685	120060918	Intron
chr5	120221428	120221636	Distal Intergenic
chr5	121086630	121087408	Distal Intergenic
chr5	125258451	125258656	Intron
chr5	125258724	125259090	Intron
chr5	127607662	127608136	Distal Intergenic
chr5	130984003	130984296	Intron

chr5	131041610	131041809	Intron
chr5	132960971	132961170	Intron
chr5	133427438	133427880	Distal Intergenic
chr5	133898677	133899898	Distal Intergenic
chr5	134435117	134435850	Distal Intergenic
chr5	134474527	134475160	Distal Intergenic
chr5	136531541	136531771	Distal Intergenic
chr5	137042480	137042679	Promoter
chr5	137043052	137043457	Promoter
chr5	137347665	137348025	Intron
chr5	139579904	139580906	Intron
chr5	140415370	140415637	Intron
chr5	141433443	141434333	Intron
chr5	141450854	141452314	Intron
chr5	145153565	145153773	Intron
chr5	147072575	147072923	Intron
chr5	147179714	147180148	Distal Intergenic
chr5	148771054	148771335	Intron
chr5	150283611	150284236	Distal Intergenic
chr5	151776440	151776771	Intron
chr6	3150795	3151622	Distal Intergenic
chr6	4942933	4943262	Intron
chr6	5306485	5307227	Distal Intergenic
chr6	7156808	7157057	Distal Intergenic
chr6	7960792	7961363	Intron
chr6	12664640	12665643	Intron
chr6	12665981	12666277	Intron
chr6	14370215	14370498	Distal Intergenic
chr6	17442653	17443723	Intron
chr6	21159160	21159359	Distal Intergenic
chr6	22069606	22069950	Intron
chr6	29697690	29699139	Intron
chr6	31789435	31789647	Distal Intergenic
chr6	44742951	44743173	Distal Intergenic
chr6	48161607	48161925	Intron
chr6	49186459	49186667	Distal Intergenic
chr6	49287659	49288463	Intron
chr6	51234214	51234413	Distal Intergenic
chr6	52999406	52999618	Intron
chr6	53304019	53304401	Intron
chr6	53383290	53383595	Intron
chr6	61898744	61899149	Intron
chr6	65797943	65798142	Intron
chr6	65798209	65798423	Intron

chr6	65798540	65798766	Intron
chr6	81926764	81927871	Distal Intergenic
chr6	82425512	82425796	Intron
chr6	83480079	83480471	Intron
chr6	86590311	86590901	Distal Intergenic
chr6	95158041	95158274	Distal Intergenic
chr6	95158322	95159009	Distal Intergenic
chr6	96960146	96960724	Intron
chr6	99642414	99642668	Promoter
chr6	99832973	99833406	Distal Intergenic
chr6	99903691	99903922	Distal Intergenic
chr6	100237022	100237519	Promoter
chr6	103264452	103264860	Distal Intergenic
chr6	104183580	104183874	Distal Intergenic
chr6	105111414	105111695	Distal Intergenic
chr6	114291028	114292674	Distal Intergenic
chr6	122474590	122474789	Intron
chr6	122792329	122792674	Intron
chr6	124940017	124940257	Distal Intergenic
chr6	125055387	125055899	Exon
chr6	125254644	125254907	Downstream
chr6	125255057	125255308	Downstream
chr6	125256124	125256408	Downstream
chr6	125967464	125968029	Intron
chr6	125968066	125968355	Intron
chr6	126700569	126701254	Distal Intergenic
chr6	126781782	126782290	Intron
chr6	127209842	127210053	Distal Intergenic
chr6	128802344	128802645	Intron
chr6	134344825	134345196	Intron
chr6	134516796	134517550	Promoter
chr6	134519942	134521027	Distal Intergenic
chr6	134521537	134521760	Distal Intergenic
chr6	134522570	134522769	Distal Intergenic
chr6	135287689	135288064	Distal Intergenic
chr6	136951520	136951765	Distal Intergenic
chr6	137791957	137792249	Distal Intergenic
chr6	138033281	138033571	Distal Intergenic
chr6	138033898	138035071	Distal Intergenic
chr6	139701393	139701592	Intron
chr6	140763924	140764461	Distal Intergenic
chr6	140930141	140930363	Intron
chr6	142143081	142143280	Distal Intergenic
chr6	142345338	142348628	Intron

chr6	142719271	142719698	Exon
chr6	143005397	143006868	Intron
chr6	143199470	143199677	Intron
chr6	143345801	143346606	Distal Intergenic
chr6	145117057	145118138	3' UTR
chr6	145306879	145307163	Distal Intergenic
chr6	145622437	145622905	Distal Intergenic
chr6	145881298	145881526	Distal Intergenic
chr6	145968083	145968604	Distal Intergenic
chr6	147863748	147864350	Distal Intergenic
chr7	6811030	6811296	Intron
chr7	7230526	7231059	Promoter
chr7	20257444	20258165	Intron
chr7	25533520	25534170	Intron
chr7	28205687	28206008	Intron
chr7	28881001	28883596	Intron
chr7	29884124	29884455	Intron
chr7	29884502	29884905	Intron
chr7	51876493	51876848	Intron
chr7	53266441	53267046	Intron
chr7	54915555	54915825	Distal Intergenic
chr7	61901759	61902251	Distal Intergenic
chr7	71610578	71641007	Distal Intergenic
chr7	79898759	79899003	Distal Intergenic
chr7	86962897	86963151	Distal Intergenic
chr7	86973493	86975317	Intron
chr7	89919542	89920545	Intron
chr7	91378236	91378820	Distal Intergenic
chr7	99971331	99971648	Intron
chr7	104592068	104592787	Distal Intergenic
chr7	104944696	104944902	Intron
chr7	107090875	107091530	Exon
chr7	108174628	108175006	Distal Intergenic
chr7	112641213	112642223	Distal Intergenic
chr7	114322642	114322946	Intron
chr7	114323175	114323640	Intron
chr7	120316415	120316726	Distal Intergenic
chr7	120615428	120616054	Distal Intergenic
chr7	125618003	125618289	Distal Intergenic
chr7	127189437	127189636	Distal Intergenic
chr7	128648593	128648795	Intron
chr7	129088133	129089189	Intron
chr7	129694401	129694600	Intron
chr7	131388771	131389450	Distal Intergenic

chr7	133302033	133302423	Intron
chr7	133394404	133394773	Distal Intergenic
chr7	134905111	134905854	Intron
chr7	135226185	135226480	Intron
chr7	135342581	135343046	Intron
chr7	135343097	135343519	Intron
chr7	137004594	137004953	Distal Intergenic
chr7	137005089	137005733	Distal Intergenic
chr7	137942505	137942764	Intron
chr7	137977871	137978134	Distal Intergenic
chr7	137994760	137995011	Distal Intergenic
chr7	139249183	139249389	3' UTR
chr7	139249438	139250466	Exon
chr7	141001843	141004753	Intron
chr7	146688530	146688775	Intron
chr7	148691749	148694982	Intron
chr8	10692142	10692433	Distal Intergenic
chr8	11594976	11595176	Distal Intergenic
chr8	11674823	11675212	Intron
chr8	13070151	13070373	Intron
chr8	13557112	13557332	Intron
chr8	13686800	13687052	Distal Intergenic
chr8	13966923	13967904	Intron
chr8	15519802	15520092	Distal Intergenic
chr8	22044394	22044639	Distal Intergenic
chr8	23946506	23946962	Distal Intergenic
chr8	24363753	24364036	Intron
chr8	24918448	24918647	Intron
chr8	33932668	33933049	Distal Intergenic
chr8	33933109	33933308	Distal Intergenic
chr8	36044098	36044489	Distal Intergenic
chr8	42455155	42455566	Intron
chr8	46468706	46469266	Distal Intergenic
chr8	55181041	55181242	Intron
chr8	61933691	61933974	Distal Intergenic
chr8	66077241	66077988	Distal Intergenic
chr8	81040777	81041142	Intron
chr8	81276659	81277103	Distal Intergenic
chr8	81350719	81351020	Distal Intergenic
chr8	84742829	84743033	Distal Intergenic
chr8	86647068	86649561	Promoter
chr8	90712548	90713421	Distal Intergenic
chr8	93617558	93617813	Intron
chr8	94205615	94205940	Distal Intergenic

chr8	97914331	97914732	Distal Intergenic
chr8	112979721	112980280	Intron
chr8	116532450	116532649	Distal Intergenic
chr8	118071837	118072196	Distal Intergenic
chr8	119607086	119607285	Promoter
chr8	119607503	119607769	Distal Intergenic
chr8	122195863	122196120	Intron
chr8	122216352	122216676	Distal Intergenic
chr8	122523289	122524236	Intron
chr8	124345965	124347127	Intron
chr8	124347325	124348355	Intron
chr8	124348668	124350085	Intron
chr8	124350147	124350958	Intron
chr8	126124567	126124961	Distal Intergenic
chr8	126142447	126142712	Distal Intergenic
chr8	126147428	126147756	Distal Intergenic
chr8	126163841	126165069	Distal Intergenic
chr8	126165480	126165773	Distal Intergenic
chr8	126708796	126709189	Distal Intergenic
chr8	127908003	127908323	Distal Intergenic
chr8	128651523	128651901	Distal Intergenic
chr8	129433850	129434220	Distal Intergenic
chr8_random	162830	163029	Promoter
chr8_random	304327	304751	Distal Intergenic
chr9	3000191	3000849	Distal Intergenic
chr9	3009553	3009768	Distal Intergenic
chr9	3013016	3013219	Distal Intergenic
chr9	3014154	3014434	Distal Intergenic
chr9	3024257	3024464	Distal Intergenic
chr9	3032428	3032627	Distal Intergenic
chr9	3258676	3259089	Distal Intergenic
chr9	13923734	13924096	Distal Intergenic
chr9	14744537	14745378	Distal Intergenic
chr9	15424251	15424662	Exon
chr9	20759267	20759587	Intron
chr9	22024582	22024907	Distal Intergenic
chr9	22249205	22250054	Downstream
chr9	22414865	22416128	Intron
chr9	22516063	22517078	Intron
chr9	26274795	26275151	Distal Intergenic
chr9	29291435	29291889	Intron
chr9	30399588	30399891	Distal Intergenic
chr9	31539220	31539434	Distal Intergenic
chr9	32094323	32094584	Distal Intergenic

chr9	32115191	32116875	Distal Intergenic
chr9	34404195	34404505	Intron
chr9	40986505	40987405	Distal Intergenic
chr9	42603332	42603532	Intron
chr9	45339683	45340059	Intron
chr9	45518847	45519570	Intron
chr9	46823907	46824280	Distal Intergenic
chr9	48015607	48016301	Intron
chr9	48198704	48199022	Promoter
chr9	48470004	48470496	Intron
chr9	50394567	50395055	Distal Intergenic
chr9	56071384	56071740	Intron
chr9	56619766	56620052	Distal Intergenic
chr9	56620225	56620424	Distal Intergenic
chr9	58224476	58225448	Distal Intergenic
chr9	61102006	61102663	Distal Intergenic
chr9	61438580	61438809	Distal Intergenic
chr9	62516383	62516725	Distal Intergenic
chr9	67287980	67289077	Intron
chr9	67289114	67289505	Intron
chr9	68947359	68947955	Intron
chr9	70470745	70471419	Intron
chr9	70804196	70804923	Distal Intergenic
chr9	70835792	70836271	Distal Intergenic
chr9	73097391	73097653	Distal Intergenic
chr9	73097819	73098034	Distal Intergenic
chr9	77500403	77501043	Intron
chr9	85064784	85065887	Distal Intergenic
chr9	88147861	88148107	Distal Intergenic
chr9	94263246	94264193	Distal Intergenic
chr9	99889491	99889933	Distal Intergenic
chr9	101312283	101312620	Distal Intergenic
chr9	105473859	105474262	Distal Intergenic
chr9	112378095	112378332	Distal Intergenic
chr9	112936153	112936760	Distal Intergenic
chr9	115853976	115854281	Intron
chr9	116746515	116747615	Intron
chr9	117144178	117144519	Intron
chr9	117997210	117999177	Intron
chr9	117999321	118002046	Intron
chr9	120950732	120951272	Intron
chr9	121788640	121788839	Distal Intergenic
chr9	122813426	122815467	Distal Intergenic
chr9	123370962	123371245	3' UTR

chrX	6589946	6592899	Intron
chrX	35309657	35309914	Distal Intergenic
chrX	35309961	35310445	Distal Intergenic
chrX	35310512	35315747	Distal Intergenic
chrX	35315958	35316473	Distal Intergenic
chrX	35316544	35316756	Distal Intergenic
chrX	35316816	35324757	Distal Intergenic
chrX	35324980	35325808	Distal Intergenic
chrX	35332039	35332481	Distal Intergenic
chrX	35332595	35332853	Distal Intergenic
chrX	35333460	35333969	Distal Intergenic
chrX	80828573	80849202	Intron
chrX	108419663	108419874	Distal Intergenic
chrX	108472161	108472493	Distal Intergenic
chrX	115912685	115912915	Distal Intergenic
chrX	147089030	147089436	Promoter
chrX	153695973	153696183	Intron
chrX	153696734	153696958	Intron
chrX	153698304	153698503	Intron
chrX	166394537	166394784	Intron
chrX	166394930	166395152	Intron
chrX	166421281	166421649	Intron
chrX	166424842	166425092	Exon
chrX	166425194	166426888	Promoter

Supplementary Table 3. H3K27ac Peak Annotation

chromosome	start	end	annotation
chr1	3659367	3660513	Exon
chr1	4775184	4775563	Intron
chr1	4847458	4849049	5' UTR
chr1	5005189	5005818	Distal Intergenic
chr1	5072993	5073908	Distal Intergenic
chr1	5077133	5077339	Distal Intergenic
chr1	6178409	6178742	Intron
chr1	6178794	6179363	Intron
chr1	6204043	6204986	Promoter
chr1	6269533	6269835	Promoter
chr1	6270005	6270954	3' UTR
chr1	6279205	6281129	3' UTR
chr1	6301364	6302242	Distal Intergenic
chr1	6302465	6302805	Distal Intergenic
chr1	6477028	6477346	3' UTR
chr1	6489133	6489343	Promoter

chr1	6918222	6918783	Intron
chr1	6918888	6919326	Intron
chr1	6929209	6930310	Intron
chr1	7079333	7079726	Intron
chr1	7079821	7080124	Intron
chr1	7115175	7116832	Intron
chr1	7189911	7190409	Intron
chr1	9122221	9122502	Intron
chr1	9122608	9124315	Intron
chr1	9143384	9144326	Intron
chr1	9535487	9535720	Distal Intergenic
chr1	9536047	9536822	Distal Intergenic
chr1	9621398	9621663	Intron
chr1	9628433	9629652	Intron
chr1	9737554	9737768	Intron
chr1	9737820	9738552	Intron
chr1	9744715	9745192	Intron
chr1	9745233	9746399	Intron
chr1	9788511	9790619	3' UTR
chr1	9931302	9932054	Exon
chr1	9932414	9934450	Intron
chr1	9973002	9973703	Distal Intergenic
chr1	10027325	10029226	Intron
chr1	10029399	10029615	Intron
chr1	10221493	10223262	Intron
chr1	10243560	10244887	Distal Intergenic
chr1	10534743	10535457	Promoter
chr1	10560360	10561915	Intron
chr1	10582633	10583839	Intron
chr1	10626595	10628238	Intron
chr1	10862575	10863000	Distal Intergenic
chr1	10870162	10870491	Distal Intergenic
chr1	12610119	12614376	Distal Intergenic
chr1	12766559	12767760	Distal Intergenic
chr1	13206347	13206983	Distal Intergenic
chr1	13294446	13294645	Distal Intergenic
chr1	13295090	13295794	Distal Intergenic
chr1	13362106	13362535	Intron
chr1	13362667	13363077	Intron
chr1	13363454	13363662	Intron
chr1	13568926	13570376	Distal Intergenic
chr1	13605552	13606695	Downstream
chr1	13629408	13629734	Distal Intergenic
chr1	13629802	13630254	Exon

chr1	13633282	13633507	Exon
chr1	13649192	13651816	Distal Intergenic
chr1	13863984	13864205	Distal Intergenic
chr1	13991673	13991945	Distal Intergenic
chr1	14263801	14264113	Distal Intergenic
chr1	14474066	14475019	Distal Intergenic
chr1	15709446	15710698	3' UTR
chr1	15774287	15775657	Distal Intergenic
chr1	15781180	15781565	Distal Intergenic
chr1	15781613	15782179	Distal Intergenic
chr1	15795202	15795600	Intron
chr1	15795740	15796746	Intron
chr1	15988053	15988832	Exon
chr1	16093615	16095130	Exon
chr1	16119515	16120994	Distal Intergenic
chr1	16162003	16162289	Exon
chr1	16162403	16162684	Exon
chr1	16218173	16218833	Intron
chr1	16409666	16409874	Distal Intergenic
chr1	16608069	16608268	Intron
chr1	16608310	16608774	Intron
chr1	16609002	16609207	Intron
chr1	16646084	16647491	Intron
chr1	16651064	16652216	Intron
chr1	16655025	16655480	Intron
chr1	16655814	16656375	Intron
chr1	16665736	16665940	Intron
chr1	16678046	16678717	Promoter
chr1	16678936	16679253	Promoter
chr1	16686547	16686747	Distal Intergenic
chr1	16879911	16880325	Distal Intergenic
chr1	16997917	16998228	Distal Intergenic
chr1	16998360	16999141	Distal Intergenic
chr1	17478299	17481534	Distal Intergenic
chr1	17594485	17594740	Intron
chr1	17594941	17596302	Intron
chr1	18120233	18120482	Intron
chr1	19764054	19765253	Intron
chr1	19784499	19784793	Intron
chr1	19941881	19942091	Intron
chr1	19946790	19947492	Intron
chr1	20861225	20861439	Distal Intergenic
chr1	20992336	20992881	Exon
chr1	20998390	20999449	Exon

chr1	21216230	21217988	Intron
chr1	21224898	21226213	Exon
chr1	21229975	21231368	Intron
chr1	23196709	23197508	Intron
chr1	23262609	23262808	Distal Intergenic
chr1	23274676	23274891	Distal Intergenic
chr1	23276657	23276898	Distal Intergenic
chr1	23277401	23279446	Distal Intergenic
chr1	23919452	23920612	Intron
chr1	23920667	23920944	Intron
chr1	23924079	23925296	Intron
chr1	23925576	23926683	Intron
chr1	23927688	23928100	Intron
chr1	24109331	24109923	Intron
chr1	24113896	24115697	3' UTR
chr1	24685568	24685856	Intron
chr1	24686060	24686294	Intron
chr1	26340634	26340833	Distal Intergenic
chr1	26355245	26355461	Intron
chr1	26446026	26446419	Intron
chr1	26451374	26451659	3' UTR
chr1	26451868	26453870	3' UTR
chr1	28898267	28898552	Exon
chr1	30239990	30242119	Distal Intergenic
chr1	30271784	30272092	Distal Intergenic
chr1	30272159	30272602	Distal Intergenic
chr1	30930586	30933222	Distal Intergenic
chr1	30960346	30960765	Distal Intergenic
chr1	31005299	31005990	Distal Intergenic
chr1	31006341	31006543	Distal Intergenic
chr1	31006704	31007697	Distal Intergenic
chr1	31070754	31071235	Distal Intergenic
chr1	31152753	31153682	Distal Intergenic
chr1	31834992	31835823	Intron
chr1	33582563	33583046	Intron
chr1	33586695	33587147	Distal Intergenic
chr1	33607853	33608227	Exon
chr1	33813506	33814062	Intron
chr1	33814276	33814536	Promoter
chr1	33814775	33815040	Promoter
chr1	33870700	33871401	Intron
chr1	34130367	34130566	Intron
chr1	34130952	34131266	Intron
chr1	34131343	34131770	Intron

chr1	34166219	34167281	Exon
chr1	34169040	34169258	Intron
chr1	34177463	34178803	Intron
chr1	34178883	34179098	Intron
chr1	34179239	34179440	Intron
chr1	34182584	34183005	Intron
chr1	34184149	34185240	Intron
chr1	34196703	34197023	Intron
chr1	34197145	34198488	Intron
chr1	34198563	34200669	Intron
chr1	34204413	34205899	Exon
chr1	34206817	34208317	Intron
chr1	34215362	34219733	Intron
chr1	34220163	34220372	Intron
chr1	34220574	34221180	Intron
chr1	34221224	34221670	Intron
chr1	34240781	34241475	Intron
chr1	34241633	34243486	Intron
chr1	34699650	34699991	Distal Intergenic
chr1	34734533	34735626	Distal Intergenic
chr1	34757674	34758460	Distal Intergenic
chr1	34758524	34758759	Distal Intergenic
chr1	34768711	34769184	Distal Intergenic
chr1	34769235	34769968	Distal Intergenic
chr1	34771068	34771283	Distal Intergenic
chr1	34772890	34774443	Distal Intergenic
chr1	34776285	34780423	Distal Intergenic
chr1	34855952	34856425	Distal Intergenic
chr1	34898605	34898916	Distal Intergenic
chr1	34899062	34899338	Distal Intergenic
chr1	34900131	34900331	Distal Intergenic
chr1	34906784	34906984	Distal Intergenic
chr1	34907043	34909679	Distal Intergenic
chr1	35719600	35720185	Distal Intergenic
chr1	35970263	35971217	Intron
chr1	36073778	36074004	Intron
chr1	36116257	36118427	Distal Intergenic
chr1	36126639	36126922	Distal Intergenic
chr1	36145051	36145370	Distal Intergenic
chr1	36146469	36147503	Distal Intergenic
chr1	36152334	36153097	Distal Intergenic
chr1	36173359	36174290	Distal Intergenic
chr1	36180534	36181012	3' UTR
chr1	36216995	36217692	Exon

chr1	36300843	36301500	Exon
chr1	36301765	36302060	Intron
chr1	36302297	36302542	Intron
chr1	36366089	36366288	Intron
chr1	36382458	36383027	Intron
chr1	36383108	36383427	Exon
chr1	36425363	36425625	Intron
chr1	36490109	36490420	Intron
chr1	36626600	36627160	Intron
chr1	36630431	36631158	Intron
chr1	36637671	36638230	Promoter
chr1	36640455	36641358	5' UTR
chr1	36704064	36705002	Promoter
chr1	36748338	36748767	Intron
chr1	36887679	36889193	Exon
chr1	36889282	36889558	Intron
chr1	36915614	36916922	Promoter
chr1	36960799	36961951	Distal Intergenic
chr1	36971954	36972806	Distal Intergenic
chr1	36976559	36977938	Distal Intergenic
chr1	36979846	36981020	Distal Intergenic
chr1	36994093	36994340	Distal Intergenic
chr1	36994409	36996020	Distal Intergenic
chr1	36996268	36997061	Distal Intergenic
chr1	37005614	37007660	Distal Intergenic
chr1	37022188	37022867	Distal Intergenic
chr1	37145757	37146526	Distal Intergenic
chr1	37272251	37273697	Intron
chr1	37486311	37486900	Intron
chr1	37486987	37487625	Intron
chr1	37643140	37643594	Distal Intergenic
chr1	37651999	37652943	Distal Intergenic
chr1	37928605	37929045	Intron
chr1	37929321	37929843	Intron
chr1	37946639	37947248	Promoter
chr1	38028473	38029086	Intron
chr1	38053476	38055936	Intron
chr1	38086521	38088309	Intron
chr1	38095178	38096336	5' UTR
chr1	38159737	38161006	Intron
chr1	38361196	38361754	Intron
chr1	38375297	38376099	Intron
chr1	38376143	38378761	Intron
chr1	38438324	38439190	Intron

chr1	38450001	38452942	Exon
chr1	38481313	38481574	Intron
chr1	38494333	38494729	Distal Intergenic
chr1	38604354	38606256	Distal Intergenic
chr1	38752807	38753122	Distal Intergenic
chr1	39028684	39030843	Distal Intergenic
chr1	39044145	39044442	Distal Intergenic
chr1	39044946	39045449	Distal Intergenic
chr1	39312846	39314109	Intron
chr1	39424197	39424396	Distal Intergenic
chr1	39424772	39424986	Distal Intergenic
chr1	39425026	39425294	Distal Intergenic
chr1	39425447	39426097	Distal Intergenic
chr1	39691700	39692052	Intron
chr1	39713215	39714930	Intron
chr1	39815953	39816529	Exon
chr1	39816708	39817152	Intron
chr1	39817820	39819219	Exon
chr1	39945870	39946913	Exon
chr1	39952021	39952316	3' UTR
chr1	39957121	39957602	Promoter
chr1	39957765	39958172	Promoter
chr1	39958215	39959992	Promoter
chr1	39963369	39964136	Intron
chr1	39988387	39989905	Exon
chr1	39997813	39998992	Intron
chr1	40024396	40026511	Promoter
chr1	40228668	40228890	Exon
chr1	40230996	40231496	Intron
chr1	40328281	40329820	Intron
chr1	40335457	40337079	Intron
chr1	40411966	40412734	Distal Intergenic
chr1	40415197	40415400	Distal Intergenic
chr1	40437969	40438189	Distal Intergenic
chr1	40441813	40442685	Distal Intergenic
chr1	40450318	40450888	Distal Intergenic
chr1	40501152	40502292	Distal Intergenic
chr1	40502340	40502588	Distal Intergenic
chr1	40599091	40600040	Distal Intergenic
chr1	40643567	40643766	Intron
chr1	42908282	42908481	Intron
chr1	43155060	43155761	Intron
chr1	43155797	43156091	Intron
chr1	43198369	43199635	3' UTR

chr1	43200578	43201018	3' UTR
chr1	43201219	43205763	Promoter
chr1	43205968	43206430	Promoter
chr1	43206637	43207000	Promoter
chr1	43208007	43208896	Distal Intergenic
chr1	43208945	43209215	Downstream
chr1	43214949	43215158	Intron
chr1	43215363	43215592	Intron
chr1	43216720	43217215	Intron
chr1	43217985	43218201	Exon
chr1	43219969	43220523	Intron
chr1	43220726	43221200	Exon
chr1	43223768	43226228	3' UTR
chr1	43226483	43226762	3' UTR
chr1	43259512	43260620	Exon
chr1	43280838	43281503	Intron
chr1	43359857	43360093	Distal Intergenic
chr1	43468926	43469137	Distal Intergenic
chr1	43469634	43469896	Distal Intergenic
chr1	43469977	43470749	Distal Intergenic
chr1	43470789	43471079	Distal Intergenic
chr1	43528112	43529272	Distal Intergenic
chr1	43529332	43529606	Distal Intergenic
chr1	43553283	43554040	Distal Intergenic
chr1	43560527	43561167	Distal Intergenic
chr1	43841236	43842637	Distal Intergenic
chr1	43876084	43876474	Intron
chr1	43990243	43990733	Distal Intergenic
chr1	43990779	43991885	Distal Intergenic
chr1	44025844	44026574	Intron
chr1	44053680	44054356	Intron
chr1	44175505	44176470	Intron
chr1	44204589	44205806	Intron
chr1	45480652	45480938	Exon
chr1	45786486	45787688	Distal Intergenic
chr1	45852004	45852232	Intron
chr1	45852269	45852469	Intron
chr1	46906795	46907714	Exon
chr1	46908275	46909184	Exon
chr1	46910142	46910387	Intron
chr1	46910430	46911165	Exon
chr1	46911428	46911733	Distal Intergenic
chr1	47019882	47020189	Intron
chr1	47020276	47020976	Intron

chr1	51122652	51122881	Intron
chr1	51458849	51459268	Distal Intergenic
chr1	51532821	51536039	Distal Intergenic
chr1	51559434	51561631	Distal Intergenic
chr1	51561710	51564739	Distal Intergenic
chr1	51565144	51566951	Promoter
chr1	51582677	51583299	Intron
chr1	51586499	51587922	Intron
chr1	51700732	51701389	Promoter
chr1	51757720	51758914	Intron
chr1	51804156	51805245	Intron
chr1	51872479	51873465	Intron
chr1	51885992	51886682	Intron
chr1	51905482	51907057	Exon
chr1	51907121	51907364	Intron
chr1	51908793	51914222	Exon
chr1	52169985	52170670	Intron
chr1	52288654	52288890	Intron
chr1	52311262	52311962	Intron
chr1	52339305	52339896	Intron
chr1	52556462	52556879	Distal Intergenic
chr1	52557611	52559013	Distal Intergenic
chr1	52635266	52635672	Intron
chr1	52654734	52655412	Intron
chr1	52779212	52780067	Intron
chr1	52780694	52780929	Intron
chr1	52781024	52781477	Intron
chr1	52855919	52857075	Intron
chr1	52859320	52859574	Exon
chr1	53841241	53841493	Distal Intergenic
chr1	53894751	53896031	Distal Intergenic
chr1	53901758	53902565	Downstream
chr1	53902608	53903689	Downstream
chr1	54770303	54770775	Intron
chr1	55083227	55084477	Intron
chr1	55108241	55109447	Promoter
chr1	55109679	55109910	Intron
chr1	55143136	55143433	Intron
chr1	55143948	55144696	Exon
chr1	55146267	55146552	Intron
chr1	55187751	55187975	Intron
chr1	55188123	55188808	Exon
chr1	55189219	55189466	Intron
chr1	55250154	55250381	3' UTR

chr1	55252499	55254180	Exon
chr1	55257981	55259431	Intron
chr1	56433235	56433492	Distal Intergenic
chr1	56738029	56738540	Distal Intergenic
chr1	56914532	56915747	Distal Intergenic
chr1	57028937	57030125	Intron
chr1	57462791	57464079	3' UTR
chr1	58045660	58046633	Intron
chr1	58052930	58053297	Intron
chr1	58086094	58088189	Intron
chr1	58360737	58361679	Intron
chr1	58416034	58416958	Intron
chr1	58423208	58424635	Intron
chr1	58448710	58448945	Intron
chr1	58449983	58450200	Intron
chr1	58450413	58451050	Intron
chr1	58479843	58480506	Intron
chr1	58480652	58480888	Intron
chr1	58480943	58481205	Intron
chr1	58502038	58503131	Intron
chr1	58643223	58643639	Intron
chr1	58708137	58710594	Intron
chr1	58715001	58715583	Promoter
chr1	58719554	58719902	Intron
chr1	58720120	58720962	Intron
chr1	58767316	58769618	Intron
chr1	58769813	58771104	Intron
chr1	58798742	58800226	Intron
chr1	58817688	58817903	Intron
chr1	58818101	58819090	Intron
chr1	58851835	58853307	Intron
chr1	58853671	58853993	Intron
chr1	58854039	58854244	Intron
chr1	58912836	58913068	Intron
chr1	58913244	58914952	Intron
chr1	58916443	58917197	Intron
chr1	59219286	59220127	Distal Intergenic
chr1	59248008	59248326	Exon
chr1	59294385	59294614	Intron
chr1	59485145	59485362	Distal Intergenic
chr1	59485430	59486926	Distal Intergenic
chr1	59487533	59487916	Distal Intergenic
chr1	59623307	59624669	Distal Intergenic
chr1	59636681	59637527	Distal Intergenic

chr1	59726682	59727965	Distal Intergenic
chr1	59741778	59742675	Distal Intergenic
chr1	59796025	59797594	Intron
chr1	59819669	59820198	Intron
chr1	59820315	59821084	Intron
chr1	59821138	59821740	Intron
chr1	59821858	59822376	Intron
chr1	59852110	59852419	Intron
chr1	59936988	59938093	Intron
chr1	59939044	59940154	Intron
chr1	60057351	60057682	Intron
chr1	60154326	60155010	Intron
chr1	60237354	60237817	Distal Intergenic
chr1	60601241	60602820	Distal Intergenic
chr1	60622564	60622829	Distal Intergenic
chr1	60665770	60667275	Distal Intergenic
chr1	60753227	60753688	Distal Intergenic
chr1	61024531	61025520	Distal Intergenic
chr1	61036244	61036942	Distal Intergenic
chr1	61151586	61152986	Intron
chr1	61541835	61542280	Promoter
chr1	61542322	61542932	Promoter
chr1	62131085	62133171	Distal Intergenic
chr1	62444899	62445101	Intron
chr1	62445209	62445468	Intron
chr1	62767418	62767645	Intron
chr1	62767900	62768974	Intron
chr1	62809938	62811205	Distal Intergenic
chr1	62811949	62812417	Distal Intergenic
chr1	62812575	62812909	Distal Intergenic
chr1	62813501	62815442	Distal Intergenic
chr1	62818442	62818727	Distal Intergenic
chr1	62840784	62841781	Distal Intergenic
chr1	62841911	62842156	Distal Intergenic
chr1	62869528	62870478	Distal Intergenic
chr1	62870539	62870877	Distal Intergenic
chr1	62954634	62955214	Exon
chr1	62955335	62955549	Intron
chr1	62968370	62968689	Intron
chr1	63159336	63161865	Distal Intergenic
chr1	63181569	63181785	Distal Intergenic
chr1	63222791	63224541	Distal Intergenic
chr1	63742912	63743257	Intron
chr1	63743293	63743592	Intron

chr1	64023616	64024088	Intron
chr1	64027424	64027715	Exon
chr1	64027986	64029115	Intron
chr1	64051323	64051796	Distal Intergenic
chr1	64051989	64052618	Distal Intergenic
chr1	64128486	64129004	Distal Intergenic
chr1	64165364	64166398	Distal Intergenic
chr1	64166543	64168007	Distal Intergenic
chr1	64168408	64169336	Distal Intergenic
chr1	64255354	64258633	Intron
chr1	64272250	64273645	Intron
chr1	64298125	64299803	Intron
chr1	64351769	64352368	Intron
chr1	64480125	64481054	Intron
chr1	64481194	64481486	Intron
chr1	64579512	64579802	Intron
chr1	64580192	64580860	Intron
chr1	64585716	64585954	Intron
chr1	64593236	64593533	Intron
chr1	64647578	64648773	Distal Intergenic
chr1	64661603	64663673	Distal Intergenic
chr1	64663787	64664074	Distal Intergenic
chr1	64802027	64802240	Distal Intergenic
chr1	64802339	64803760	Distal Intergenic
chr1	64841960	64844200	Distal Intergenic
chr1	64855046	64855323	Distal Intergenic
chr1	64855473	64855805	Distal Intergenic
chr1	64860397	64861633	Distal Intergenic
chr1	64987800	64989463	Intron
chr1	64991480	64992220	Intron
chr1	65164548	65166032	Distal Intergenic
chr1	65225313	65225684	Intron
chr1	65233279	65233599	Intron
chr1	65436458	65436942	Distal Intergenic
chr1	65878900	65880011	3' UTR
chr1	65978279	65980293	Intron
chr1	65984429	65984924	Intron
chr1	66108679	66109725	Distal Intergenic
chr1	66186861	66187318	Distal Intergenic
chr1	66191296	66191994	Distal Intergenic
chr1	66327113	66327387	Intron
chr1	66444273	66445599	Intron
chr1	66747470	66747720	Intron
chr1	66747911	66748360	Intron

chr1	66834680	66836076	Intron
chr1	66861193	66861467	Distal Intergenic
chr1	66862145	66863478	Distal Intergenic
chr1	66892038	66893958	Distal Intergenic
chr1	67083365	67085162	Intron
chr1	67085202	67085428	Intron
chr1	67088537	67089216	Intron
chr1	67090630	67092555	Exon
chr1	67095340	67095826	Intron
chr1	67496954	67498547	Intron
chr1	68826464	68827065	Distal Intergenic
chr1	69331959	69333643	Distal Intergenic
chr1	69413884	69414250	Distal Intergenic
chr1	69414501	69414922	Distal Intergenic
chr1	69426329	69426718	Distal Intergenic
chr1	69443601	69448965	Distal Intergenic
chr1	69479481	69481522	Distal Intergenic
chr1	69487064	69487707	Distal Intergenic
chr1	69499879	69500159	Distal Intergenic
chr1	69504048	69504406	Distal Intergenic
chr1	69506758	69507045	Distal Intergenic
chr1	69507143	69508437	Distal Intergenic
chr1	69513595	69514108	Distal Intergenic
chr1	69514273	69516431	Distal Intergenic
chr1	69554188	69555682	Distal Intergenic
chr1	69701505	69702953	Distal Intergenic
chr1	69703904	69704189	Distal Intergenic
chr1	69704373	69705506	Distal Intergenic
chr1	69732905	69733505	Distal Intergenic
chr1	69734407	69734711	Distal Intergenic
chr1	70124991	70125591	Intron
chr1	70181590	70181833	Intron
chr1	70182142	70182759	Intron
chr1	70965114	70965328	Distal Intergenic
chr1	71046523	71047317	Distal Intergenic
chr1	71049940	71050217	Distal Intergenic
chr1	71050433	71051898	Distal Intergenic
chr1	71142075	71142479	Distal Intergenic
chr1	71143149	71143366	Distal Intergenic
chr1	71148730	71149017	Distal Intergenic
chr1	71149109	71149530	Distal Intergenic
chr1	71149663	71150130	Distal Intergenic
chr1	71151722	71153281	Distal Intergenic
chr1	71381810	71382344	Intron

chr1	71385197	71386492	Intron
chr1	71416524	71416723	Intron
chr1	71416821	71417048	Intron
chr1	71442390	71445252	Intron
chr1	71459353	71460939	Intron
chr1	71461107	71461472	Intron
chr1	71461517	71461980	Intron
chr1	71504207	71508590	Intron
chr1	71524905	71526049	Intron
chr1	71603628	71603867	Intron
chr1	71604158	71604390	Intron
chr1	71713333	71713584	Distal Intergenic
chr1	71713790	71714933	Distal Intergenic
chr1	71737890	71738281	Distal Intergenic
chr1	71738398	71738718	Distal Intergenic
chr1	71801598	71802674	Distal Intergenic
chr1	71927010	71927263	Intron
chr1	71927311	71927638	Intron
chr1	71940419	71940775	Intron
chr1	72116902	72117403	Intron
chr1	72232722	72233033	Intron
chr1	72239593	72239851	Intron
chr1	72240068	72241223	Intron
chr1	72248548	72250555	Intron
chr1	72251692	72252000	Intron
chr1	72257426	72258607	Intron
chr1	72273090	72273852	Intron
chr1	72284020	72285274	Intron
chr1	72289478	72290434	Intron
chr1	72290541	72291691	Intron
chr1	72301806	72302415	Promoter
chr1	72353626	72353898	Intron
chr1	72630118	72630357	Intron
chr1	72758157	72758872	Distal Intergenic
chr1	72779153	72781180	Distal Intergenic
chr1	72782009	72783637	Distal Intergenic
chr1	72785420	72786209	Distal Intergenic
chr1	72794071	72794910	Distal Intergenic
chr1	72795015	72795840	Distal Intergenic
chr1	72799639	72800660	Distal Intergenic
chr1	72801630	72802879	Distal Intergenic
chr1	72824403	72828455	Distal Intergenic
chr1	72828715	72829683	Distal Intergenic
chr1	72829856	72830982	Distal Intergenic

chr1	72840181	72840380	Distal Intergenic
chr1	72867102	72868354	Distal Intergenic
chr1	72871921	72872863	Distal Intergenic
chr1	72874724	72874923	Distal Intergenic
chr1	72894567	72894778	Distal Intergenic
chr1	73517922	73518831	Distal Intergenic
chr1	73550240	73551809	Distal Intergenic
chr1	73553314	73553990	Distal Intergenic
chr1	73554161	73554905	Distal Intergenic
chr1	74002082	74002517	Distal Intergenic
chr1	74055458	74055739	Distal Intergenic
chr1	74195535	74195957	Distal Intergenic
chr1	74196090	74196302	Distal Intergenic
chr1	74283137	74286558	Distal Intergenic
chr1	74286652	74287208	Distal Intergenic
chr1	74294746	74294987	Distal Intergenic
chr1	74295228	74295795	Distal Intergenic
chr1	74330983	74331336	Distal Intergenic
chr1	74341559	74346313	Distal Intergenic
chr1	74346812	74347397	Distal Intergenic
chr1	74347538	74347766	Distal Intergenic
chr1	74348990	74349667	Distal Intergenic
chr1	74349862	74350713	Distal Intergenic
chr1	74350751	74351174	Distal Intergenic
chr1	74433266	74433889	Distal Intergenic
chr1	74443219	74443685	Distal Intergenic
chr1	74443848	74444389	Distal Intergenic
chr1	74552607	74554050	Intron
chr1	74634383	74634658	Intron
chr1	74647422	74647954	Intron
chr1	74772682	74773092	Intron
chr1	74773182	74773764	Intron
chr1	74838585	74841982	Intron
chr1	74842077	74842405	Intron
chr1	74850306	74853637	Intron
chr1	75121200	75121753	Intron
chr1	75138413	75138690	Promoter
chr1	75163867	75165165	Distal Intergenic
chr1	75189055	75189493	Intron
chr1	75207179	75207588	Intron
chr1	75210807	75211345	Intron
chr1	75211399	75212202	Intron
chr1	75214843	75215839	Intron
chr1	75226961	75229230	Intron

chr1	75231412	75231846	3' UTR
chr1	75232480	75232894	Distal Intergenic
chr1	75233006	75233348	Distal Intergenic
chr1	75233430	75233750	Distal Intergenic
chr1	75244877	75245098	Distal Intergenic
chr1	75245229	75245449	Distal Intergenic
chr1	75289307	75290343	Distal Intergenic
chr1	75313448	75313849	Distal Intergenic
chr1	75432532	75432733	Distal Intergenic
chr1	75517716	75518297	Distal Intergenic
chr1	75584489	75585407	Distal Intergenic
chr1	75585508	75585872	Distal Intergenic
chr1	76020896	76022101	Intron
chr1	76163774	76164221	Intron
chr1	76293111	76293647	Intron
chr1	76638137	76638394	Intron
chr1	76641631	76641851	Intron
chr1	76641917	76643467	Intron
chr1	76648259	76648554	Intron
chr1	76660593	76660796	Intron
chr1	76714197	76714647	Intron
chr1	76714752	76715016	Intron
chr1	76715057	76715986	Intron
chr1	76831643	76831867	Intron
chr1	76869525	76871398	Intron
chr1	76876712	76877559	Intron
chr1	76923554	76924990	Intron
chr1	77004015	77005120	Intron
chr1	77077469	77078046	Intron
chr1	77457081	77460094	Intron
chr1	77461705	77461914	Intron
chr1	77462036	77462281	Intron
chr1	77462377	77462634	Intron
chr1	77463988	77464467	Intron
chr1	77467206	77468369	Intron
chr1	77691640	77691878	Distal Intergenic
chr1	77692088	77692545	Distal Intergenic
chr1	77735556	77736129	Distal Intergenic
chr1	77736168	77736446	Distal Intergenic
chr1	77753923	77755285	Intron
chr1	77836900	77837884	Intron
chr1	77842060	77843490	Intron
chr1	78485835	78486143	Distal Intergenic
chr1	78529313	78529665	Intron

chr1	78533957	78534306	Intron
chr1	78534396	78535277	Intron
chr1	78587905	78588619	Intron
chr1	78588842	78589181	Intron
chr1	78589617	78589821	Intron
chr1	78640551	78641534	Distal Intergenic
chr1	78652724	78653884	Distal Intergenic
chr1	78654415	78654828	Distal Intergenic
chr1	78654904	78656247	Distal Intergenic
chr1	78677138	78677529	Distal Intergenic
chr1	79140902	79141375	Distal Intergenic
chr1	79173512	79173997	Distal Intergenic
chr1	79577749	79578080	Distal Intergenic
chr1	79687827	79688548	Distal Intergenic
chr1	79742343	79743252	Distal Intergenic
chr1	79757245	79757444	Distal Intergenic
chr1	79826688	79827120	Distal Intergenic
chr1	79827158	79827363	Distal Intergenic
chr1	80022015	80022499	Distal Intergenic
chr1	80217081	80218321	Distal Intergenic
chr1	80335899	80336686	Distal Intergenic
chr1	81363027	81364252	Distal Intergenic
chr1	81365654	81366124	Distal Intergenic
chr1	81620621	81620845	Distal Intergenic
chr1	81620898	81621158	Distal Intergenic
chr1	81626879	81627316	Distal Intergenic
chr1	81667335	81668532	Distal Intergenic
chr1	81673586	81675146	Distal Intergenic
chr1	81755024	81757157	Distal Intergenic
chr1	81910207	81910421	Intron
chr1	82028787	82029131	Intron
chr1	82152594	82153308	Intron
chr1	82313199	82313416	Intron
chr1	82368544	82368971	Intron
chr1	82425881	82426131	Intron
chr1	82593633	82596198	Distal Intergenic
chr1	82596252	82596556	Distal Intergenic
chr1	82721497	82721746	Distal Intergenic
chr1	82789503	82790356	Distal Intergenic
chr1	82791543	82793069	Distal Intergenic
chr1	82828187	82828579	Distal Intergenic
chr1	82836060	82836300	Distal Intergenic
chr1	82875709	82875932	Distal Intergenic
chr1	82905092	82905907	Distal Intergenic

chr1	82943440	82944791	Distal Intergenic
chr1	82951418	82951799	Distal Intergenic
chr1	84403198	84403935	Exon
chr1	84769234	84769775	Promoter
chr1	84834611	84835441	Intron
chr1	84836249	84837359	Intron
chr1	84877297	84879013	5' UTR
chr1	84935584	84937314	Distal Intergenic
chr1	84945382	84946913	Promoter
chr1	87690524	87691542	Distal Intergenic
chr1	87791765	87794049	Promoter
chr1	87794839	87795218	Promoter
chr1	87795330	87795669	Intron
chr1	87796558	87796829	Promoter
chr1	87796998	87797352	Promoter
chr1	87961000	87962153	Distal Intergenic
chr1	88042708	88043526	Distal Intergenic
chr1	88044121	88044324	Distal Intergenic
chr1	88046799	88048941	Distal Intergenic
chr1	88049010	88049226	Distal Intergenic
chr1	88050451	88051309	Distal Intergenic
chr1	88140257	88140805	Distal Intergenic
chr1	88255598	88256067	Distal Intergenic
chr1	88256163	88256781	Distal Intergenic
chr1	88272372	88273608	Distal Intergenic
chr1	88331157	88331685	Distal Intergenic
chr1	88334093	88337218	Distal Intergenic
chr1	88366902	88369704	Distal Intergenic
chr1	88412317	88412893	Distal Intergenic
chr1	88418849	88419735	Distal Intergenic
chr1	88424303	88424557	Distal Intergenic
chr1	88424761	88425047	Distal Intergenic
chr1	88425233	88425546	Distal Intergenic
chr1	88427658	88427880	Distal Intergenic
chr1	88428214	88429083	Distal Intergenic
chr1	88518826	88520763	Distal Intergenic
chr1	88576398	88576650	Distal Intergenic
chr1	88576745	88577154	Distal Intergenic
chr1	88920877	88921181	Distal Intergenic
chr1	88921218	88921570	Distal Intergenic
chr1	88925528	88925933	Distal Intergenic
chr1	88932252	88932675	Distal Intergenic
chr1	88933715	88935766	Distal Intergenic
chr1	88936522	88937839	Distal Intergenic

chr1	89077227	89078070	Intron
chr1	89110234	89110466	Intron
chr1	89111057	89111470	Intron
chr1	89149364	89149651	Promoter
chr1	89151892	89152095	Intron
chr1	89224026	89224448	Intron
chr1	89306671	89307922	Distal Intergenic
chr1	89308746	89309559	Distal Intergenic
chr1	89391271	89391482	Distal Intergenic
chr1	89445547	89445770	3' UTR
chr1	89446925	89448251	3' UTR
chr1	89455846	89456148	Intron
chr1	89456198	89457195	Intron
chr1	89778230	89778768	Distal Intergenic
chr1	89867084	89867513	Distal Intergenic
chr1	89990879	89991931	Promoter
chr1	89993521	89995130	Intron
chr1	89998919	90000654	Intron
chr1	90005547	90005950	Intron
chr1	90025701	90025970	Intron
chr1	90026238	90026563	Intron
chr1	90029946	90032618	Intron
chr1	90036903	90037780	Intron
chr1	90049478	90050898	Exon
chr1	90134307	90134646	Intron
chr1	90158818	90159506	Intron
chr1	90173645	90174254	Intron
chr1	90257236	90259351	Distal Intergenic
chr1	90276785	90278403	Distal Intergenic
chr1	90318882	90320164	Intron
chr1	90329675	90330240	Intron
chr1	90333400	90334995	Intron
chr1	90339722	90340207	Intron
chr1	90370412	90372845	Intron
chr1	90402494	90402806	Distal Intergenic
chr1	90404299	90405839	Distal Intergenic
chr1	90421780	90422404	Distal Intergenic
chr1	90422546	90423779	Distal Intergenic
chr1	90430117	90431800	Distal Intergenic
chr1	90432902	90434466	Distal Intergenic
chr1	90439211	90440693	Distal Intergenic
chr1	90453066	90453521	Exon
chr1	90483474	90484497	Exon
chr1	90535809	90536626	Distal Intergenic

chr1	90576819	90577595	Distal Intergenic
chr1	90595636	90597164	Distal Intergenic
chr1	90597231	90598204	Distal Intergenic
chr1	90598309	90598601	Distal Intergenic
chr1	90598953	90599161	Distal Intergenic
chr1	90765611	90766813	Distal Intergenic
chr1	90813666	90814020	Distal Intergenic
chr1	90814394	90814600	Distal Intergenic
chr1	90911722	90912911	Distal Intergenic
chr1	90915980	90917158	Distal Intergenic
chr1	90932892	90933291	Distal Intergenic
chr1	90965262	90966712	Distal Intergenic
chr1	90967003	90967202	Distal Intergenic
chr1	90997826	90998182	Distal Intergenic
chr1	90998264	90999228	Distal Intergenic
chr1	91017868	91021134	Distal Intergenic
chr1	91027814	91028617	Distal Intergenic
chr1	91029874	91030114	Distal Intergenic
chr1	91370707	91372007	Distal Intergenic
chr1	91409859	91411356	Intron
chr1	91471335	91471682	Intron
chr1	91473434	91473797	Intron
chr1	91499454	91499690	Distal Intergenic
chr1	91544301	91545281	Distal Intergenic
chr1	91552498	91554338	Distal Intergenic
chr1	91582741	91584192	Distal Intergenic
chr1	91593011	91593359	Distal Intergenic
chr1	91593418	91594453	Distal Intergenic
chr1	91597003	91597202	Distal Intergenic
chr1	91597482	91598087	Distal Intergenic
chr1	91626566	91627508	Distal Intergenic
chr1	91701393	91702229	Distal Intergenic
chr1	91947547	91948089	Distal Intergenic
chr1	91993857	91994618	Distal Intergenic
chr1	91996500	91997304	Distal Intergenic
chr1	92060347	92061698	Distal Intergenic
chr1	92079949	92081063	Distal Intergenic
chr1	92081323	92081699	Distal Intergenic
chr1	92082366	92083227	Distal Intergenic
chr1	92100581	92102667	Distal Intergenic
chr1	92103155	92103370	Distal Intergenic
chr1	92118011	92118847	Distal Intergenic
chr1	92120609	92124407	Distal Intergenic
chr1	92126995	92127580	Distal Intergenic

chr1	92130567	92131303	Distal Intergenic
chr1	92131369	92131653	Distal Intergenic
chr1	92131699	92131957	Distal Intergenic
chr1	92155078	92156196	Intron
chr1	92312593	92314044	Intron
chr1	92336328	92338114	Intron
chr1	92574077	92574655	Intron
chr1	92919889	92920616	Distal Intergenic
chr1	92920747	92921090	Distal Intergenic
chr1	92950017	92951076	Promoter
chr1	92978051	92978865	3' UTR
chr1	92991410	92991925	Intron
chr1	92991988	92994198	Intron
chr1	93023427	93023827	Intron
chr1	93144384	93145762	Intron
chr1	93146401	93146874	Intron
chr1	93147267	93147885	Intron
chr1	93159552	93159992	Promoter
chr1	93160080	93160285	Promoter
chr1	93168729	93169449	Exon
chr1	93174837	93176159	Intron
chr1	93294958	93295495	Distal Intergenic
chr1	93296856	93298473	Promoter
chr1	93386725	93387097	Intron
chr1	93537138	93537738	Distal Intergenic
chr1	93695848	93697569	Intron
chr1	93956052	93956713	Intron
chr1	93982329	93985322	Intron
chr1	93995806	93996679	Exon
chr1	94002755	94003033	Intron
chr1	94003204	94003690	Intron
chr1	94008046	94008493	Intron
chr1	94008671	94008871	Promoter
chr1	94009232	94009458	Promoter
chr1	94015951	94016682	Exon
chr1	94026019	94027600	3' UTR
chr1	94027695	94028452	3' UTR
chr1	94028505	94028704	Intron
chr1	94040535	94042416	Exon
chr1	94058548	94059709	Intron
chr1	94062794	94063556	Intron
chr1	94063776	94064740	Exon
chr1	94075919	94076199	Intron
chr1	94077225	94077758	Intron

chr1	94077850	94078162	Intron
chr1	94078226	94078675	Intron
chr1	94078895	94081087	Promoter
chr1	94369808	94370354	Exon
chr1	94579573	94580538	Intron
chr1	94641834	94642036	Intron
chr1	94695920	94696148	Intron
chr1	94696485	94697241	5' UTR
chr1	94712200	94712643	Intron
chr1	94799920	94801441	Distal Intergenic
chr1	94808177	94808387	Distal Intergenic
chr1	94827088	94827599	Distal Intergenic
chr1	94830417	94831383	Distal Intergenic
chr1	94868650	94869451	Distal Intergenic
chr1	94875216	94875635	Distal Intergenic
chr1	95026950	95027381	Distal Intergenic
chr1	95027491	95028081	Distal Intergenic
chr1	95056819	95057563	Distal Intergenic
chr1	95084890	95085180	Distal Intergenic
chr1	95093490	95095062	Distal Intergenic
chr1	95196078	95197330	Intron
chr1	95341129	95342717	Intron
chr1	95374162	95374416	Intron
chr1	95374454	95374976	Intron
chr1	95375133	95375434	Intron
chr1	95375512	95376812	Intron
chr1	95425377	95427275	Exon
chr1	95576830	95577176	Intron
chr1	95601604	95603061	Intron
chr1	95645142	95645556	Intron
chr1	95650343	95650579	Intron
chr1	95721839	95722092	Distal Intergenic
chr1	96045894	96046939	Distal Intergenic
chr1	97364776	97365080	Distal Intergenic
chr1	99666899	99667154	Distal Intergenic
chr1	99807225	99808488	Distal Intergenic
chr1	99860604	99861016	Distal Intergenic
chr1	102045693	102047687	Distal Intergenic
chr1	102067596	102069409	Distal Intergenic
chr1	102069640	102070311	Distal Intergenic
chr1	103029753	103031568	Distal Intergenic
chr1	103689359	103690374	Distal Intergenic
chr1	107186441	107188808	Distal Intergenic
chr1	107249753	107250378	Distal Intergenic

chr1	107250448	107250782	Distal Intergenic
chr1	107327261	107327734	Distal Intergenic
chr1	107328357	107328945	Distal Intergenic
chr1	107339075	107339323	Distal Intergenic
chr1	107383390	107384548	Distal Intergenic
chr1	107559432	107560285	Distal Intergenic
chr1	107762286	107762558	Intron
chr1	107762596	107764008	Intron
chr1	107888775	107889887	Intron
chr1	107889976	107890202	Intron
chr1	107954876	107955472	Intron
chr1	108059138	108059376	Distal Intergenic
chr1	108107501	108108327	Distal Intergenic
chr1	108137500	108139110	Exon
chr1	108164397	108165282	Intron
chr1	108303568	108304633	Intron
chr1	108304684	108305107	Intron
chr1	108357420	108358629	Intron
chr1	108498967	108499290	Intron
chr1	108656402	108657003	Distal Intergenic
chr1	108661794	108663718	Distal Intergenic
chr1	108692168	108693891	Intron
chr1	108718510	108719446	Intron
chr1	108719506	108719875	Intron
chr1	108753608	108754040	Distal Intergenic
chr1	108754104	108755330	Distal Intergenic
chr1	108756642	108760396	Distal Intergenic
chr1	108761520	108762824	Distal Intergenic
chr1	109399310	109399509	Promoter
chr1	109401810	109403701	Promoter
chr1	109424724	109428211	5' UTR
chr1	109434814	109436153	Intron
chr1	109486589	109487882	Exon
chr1	109487921	109488173	Intron
chr1	109942971	109943180	3' UTR
chr1	113761047	113761275	Distal Intergenic
chr1	116337958	116339501	Distal Intergenic
chr1	116339541	116339825	Distal Intergenic
chr1	120207376	120208017	Distal Intergenic
chr1	120265011	120266089	5' UTR
chr1	120276896	120277236	Intron
chr1	120283697	120285900	Exon
chr1	120286524	120286835	3' UTR
chr1	120321040	120321769	Distal Intergenic

chr1	120321853	120322080	Distal Intergenic
chr1	120355054	120355263	Promoter
chr1	120355393	120356050	Distal Intergenic
chr1	120435374	120435640	Downstream
chr1	120704114	120704814	Distal Intergenic
chr1	120721428	120722968	Distal Intergenic
chr1	120748554	120749095	Distal Intergenic
chr1	120769886	120771066	Distal Intergenic
chr1	120784156	120784364	Distal Intergenic
chr1	120849592	120850022	Intron
chr1	120872866	120873532	Distal Intergenic
chr1	120961582	120961952	Distal Intergenic
chr1	121021336	121021756	Distal Intergenic
chr1	121021794	121022583	Distal Intergenic
chr1	121063713	121064049	Distal Intergenic
chr1	121064105	121064500	Distal Intergenic
chr1	121121426	121121868	Intron
chr1	121196892	121197282	Distal Intergenic
chr1	121400348	121400560	Distal Intergenic
chr1	121400600	121400861	Distal Intergenic
chr1	121433155	121435298	Distal Intergenic
chr1	121979570	121980062	Distal Intergenic
chr1	121980166	121980807	Distal Intergenic
chr1	122016667	122017372	Distal Intergenic
chr1	122032626	122032962	Distal Intergenic
chr1	122033015	122033345	Distal Intergenic
chr1	122636444	122636824	Distal Intergenic
chr1	122640546	122640759	Distal Intergenic
chr1	122653252	122655957	Distal Intergenic
chr1	122880350	122880796	Distal Intergenic
chr1	122931600	122931915	Distal Intergenic
chr1	123257317	123257709	Distal Intergenic
chr1	123273723	123274490	Distal Intergenic
chr1	123274535	123274880	Distal Intergenic
chr1	123454876	123455212	Distal Intergenic
chr1	123550428	123550777	Distal Intergenic
chr1	125743573	125743960	Distal Intergenic
chr1	125886712	125886993	Distal Intergenic
chr1	126383804	126385919	Distal Intergenic
chr1	127330431	127332094	Distal Intergenic
chr1	127346058	127348028	Distal Intergenic
chr1	127378799	127379282	Distal Intergenic
chr1	127379588	127380277	Distal Intergenic
chr1	127422316	127422528	Distal Intergenic

chr1	127422564	127423146	Distal Intergenic
chr1	127446350	127447274	Distal Intergenic
chr1	127456586	127456916	Distal Intergenic
chr1	127464386	127465273	Distal Intergenic
chr1	127597727	127598254	Distal Intergenic
chr1	127598418	127599060	Distal Intergenic
chr1	128489428	128490462	Distal Intergenic
chr1	128498730	128499261	Distal Intergenic
chr1	128505292	128506137	Distal Intergenic
chr1	128514990	128516668	Distal Intergenic
chr1	128555031	128555947	Distal Intergenic
chr1	128567114	128567655	Distal Intergenic
chr1	128575882	128577030	Distal Intergenic
chr1	128580117	128581624	Distal Intergenic
chr1	128634014	128634224	Distal Intergenic
chr1	128713776	128714929	Distal Intergenic
chr1	128897701	128898934	Distal Intergenic
chr1	128899002	128899759	Distal Intergenic
chr1	129115751	129116035	Distal Intergenic
chr1	129116082	129116688	Distal Intergenic
chr1	129129739	129130182	Distal Intergenic
chr1	129153856	129154238	Distal Intergenic
chr1	129670806	129671576	Distal Intergenic
chr1	129757212	129757428	Distal Intergenic
chr1	129879626	129879842	Distal Intergenic
chr1	129880024	129880708	Distal Intergenic
chr1	129887932	129888572	Distal Intergenic
chr1	130140925	130141841	Distal Intergenic
chr1	130253966	130254637	Distal Intergenic
chr1	131547992	131548432	Distal Intergenic
chr1	132587389	132587613	Distal Intergenic
chr1	132612715	132613776	Distal Intergenic
chr1	132614134	132614451	Distal Intergenic
chr1	132628270	132628538	Distal Intergenic
chr1	132629225	132629494	Distal Intergenic
chr1	132629548	132632104	Distal Intergenic
chr1	132637644	132637880	Distal Intergenic
chr1	132643526	132644124	Distal Intergenic
chr1	132666292	132668654	Distal Intergenic
chr1	132699468	132700065	Distal Intergenic
chr1	132700178	132700692	Distal Intergenic
chr1	132776287	132777868	Distal Intergenic
chr1	132777965	132778323	Distal Intergenic
chr1	132782807	132784056	Distal Intergenic

chr1	132788670	132790036	Distal Intergenic
chr1	132814791	132816052	Distal Intergenic
chr1	132838981	132841376	Distal Intergenic
chr1	132843591	132844879	Distal Intergenic
chr1	132906836	132907690	Distal Intergenic
chr1	132960682	132962037	Distal Intergenic
chr1	132993530	132993782	Distal Intergenic
chr1	133020171	133020542	Distal Intergenic
chr1	133020711	133021204	Distal Intergenic
chr1	133022677	133023363	Distal Intergenic
chr1	133031969	133032241	Distal Intergenic
chr1	133035003	133036417	Distal Intergenic
chr1	133077144	133077441	Distal Intergenic
chr1	133081454	133081752	Distal Intergenic
chr1	133117700	133118976	Distal Intergenic
chr1	133134712	133136991	Distal Intergenic
chr1	133137042	133137333	Distal Intergenic
chr1	133141981	133142277	Distal Intergenic
chr1	133175614	133177147	Distal Intergenic
chr1	133190169	133191122	Distal Intergenic
chr1	133229796	133230225	Distal Intergenic
chr1	133397754	133399984	Distal Intergenic
chr1	133401807	133402364	Distal Intergenic
chr1	133402502	133404257	Distal Intergenic
chr1	133405393	133405771	Distal Intergenic
chr1	133410211	133410628	Distal Intergenic
chr1	133422950	133423400	Distal Intergenic
chr1	133586101	133588245	Distal Intergenic
chr1	133807081	133807697	Distal Intergenic
chr1	133890265	133890561	Distal Intergenic
chr1	133898580	133899093	Distal Intergenic
chr1	133901209	133901408	Distal Intergenic
chr1	133903824	133904110	Distal Intergenic
chr1	133904219	133905233	Distal Intergenic
chr1	133907058	133907419	Distal Intergenic
chr1	134035352	134035553	Distal Intergenic
chr1	134035820	134036030	Distal Intergenic
chr1	134046923	134048858	Distal Intergenic
chr1	134092035	134092483	Distal Intergenic
chr1	134092962	134093186	Distal Intergenic
chr1	134151593	134151844	Distal Intergenic
chr1	134151914	134152221	Distal Intergenic
chr1	134161001	134162310	Distal Intergenic
chr1	134177057	134177878	Distal Intergenic

chr1	134183436	134184171	Distal Intergenic
chr1	134187769	134189583	Distal Intergenic
chr1	134191466	134192862	Distal Intergenic
chr1	134212663	134212887	Distal Intergenic
chr1	134213463	134216211	Distal Intergenic
chr1	134231400	134232732	Distal Intergenic
chr1	134243038	134243721	Distal Intergenic
chr1	134249607	134251376	Distal Intergenic
chr1	134314187	134314471	Distal Intergenic
chr1	134314525	134314811	Distal Intergenic
chr1	134327864	134330438	Distal Intergenic
chr1	134462363	134465906	Distal Intergenic
chr1	134467428	134467802	Distal Intergenic
chr1	134467913	134468112	Distal Intergenic
chr1	134915384	134915592	Distal Intergenic
chr1	134915649	134918027	Distal Intergenic
chr1	134920836	134921207	Distal Intergenic
chr1	134921483	134922352	Distal Intergenic
chr1	135027246	135029120	Distal Intergenic
chr1	135029267	135029492	Distal Intergenic
chr1	135029868	135030067	Distal Intergenic
chr1	135128169	135128522	Distal Intergenic
chr1	135128620	135129090	Distal Intergenic
chr1	135173405	135173742	Distal Intergenic
chr1	135173896	135174149	Distal Intergenic
chr1	135373497	135373927	Distal Intergenic
chr1	135506228	135506455	Distal Intergenic
chr1	135506677	135506899	Distal Intergenic
chr1	135557049	135558165	Distal Intergenic
chr1	135590482	135593283	Distal Intergenic
chr1	135673990	135674465	Distal Intergenic
chr1	135674554	135674793	Distal Intergenic
chr1	135674844	135675965	Distal Intergenic
chr1	135751966	135752729	Distal Intergenic
chr1	135861344	135862627	Distal Intergenic
chr1	135863983	135865208	Distal Intergenic
chr1	135962354	135962753	Distal Intergenic
chr1	135975980	135976263	Distal Intergenic
chr1	135995444	135998583	Distal Intergenic
chr1	136010010	136011837	Distal Intergenic
chr1	136302494	136302781	Distal Intergenic
chr1	136311244	136311519	Distal Intergenic
chr1	136311623	136311884	Distal Intergenic
chr1	136312604	136313004	Distal Intergenic

chr1	136314396	136314672	Distal Intergenic
chr1	136314709	136315211	Distal Intergenic
chr1	136315255	136317027	Distal Intergenic
chr1	136317178	136317583	Distal Intergenic
chr1	136477228	136477726	Distal Intergenic
chr1	136480014	136480882	Distal Intergenic
chr1	136699919	136701465	Distal Intergenic
chr1	137043616	137044073	Distal Intergenic
chr1	137079772	137079971	Distal Intergenic
chr1	137080017	137080225	Distal Intergenic
chr1	137080319	137081137	Distal Intergenic
chr1	137155164	137155401	Distal Intergenic
chr1	137155537	137155825	Distal Intergenic
chr1	137156913	137158131	Distal Intergenic
chr1	137164485	137165520	Distal Intergenic
chr1	137179636	137179852	Distal Intergenic
chr1	137179902	137180249	Distal Intergenic
chr1	137209770	137210141	Distal Intergenic
chr1	137255268	137255806	Distal Intergenic
chr1	137307452	137308198	Distal Intergenic
chr1	137363225	137364215	Distal Intergenic
chr1	137373709	137374967	Distal Intergenic
chr1	137375012	137377850	Distal Intergenic
chr1	137386470	137389813	Distal Intergenic
chr1	137390770	137391246	Distal Intergenic
chr1	137391367	137393053	Distal Intergenic
chr1	137405902	137406737	Distal Intergenic
chr1	137406778	137407492	Distal Intergenic
chr1	137431525	137433315	Distal Intergenic
chr1	137565303	137566025	Distal Intergenic
chr1	137611048	137611584	Distal Intergenic
chr1	137611625	137612350	Distal Intergenic
chr1	137627009	137627475	Distal Intergenic
chr1	137627620	137628975	Distal Intergenic
chr1	137651135	137651356	Distal Intergenic
chr1	137651479	137653269	Distal Intergenic
chr1	137655728	137657791	Distal Intergenic
chr1	137660347	137661011	Distal Intergenic
chr1	137661691	137662624	Distal Intergenic
chr1	137662686	137668974	Distal Intergenic
chr1	137688673	137689644	Distal Intergenic
chr1	137689902	137691341	Distal Intergenic
chr1	137709221	137709476	Distal Intergenic
chr1	137715534	137717060	Distal Intergenic

chr1	137717260	137718669	Distal Intergenic
chr1	137743240	137743536	Distal Intergenic
chr1	137748702	137749894	Distal Intergenic
chr1	137753191	137754846	Distal Intergenic
chr1	137758179	137761000	Distal Intergenic
chr1	137764480	137767103	Distal Intergenic
chr1	137771119	137771356	Distal Intergenic
chr1	137775198	137775427	Distal Intergenic
chr1	137779484	137781407	Distal Intergenic
chr1	137786966	137787178	Distal Intergenic
chr1	137787375	137788320	Distal Intergenic
chr1	137788464	137791599	Distal Intergenic
chr1	137791741	137793475	Distal Intergenic
chr1	137794542	137794760	Distal Intergenic
chr1	137794808	137801056	Distal Intergenic
chr1	137801321	137801643	Distal Intergenic
chr1	137801940	137802198	Distal Intergenic
chr1	137802288	137804552	Distal Intergenic
chr1	137804736	137804976	Distal Intergenic
chr1	137805952	137806163	Distal Intergenic
chr1	137806379	137806736	Distal Intergenic
chr1	137806858	137807386	Distal Intergenic
chr1	137809496	137810064	Distal Intergenic
chr1	137810289	137811330	Distal Intergenic
chr1	137811489	137814734	Distal Intergenic
chr1	137814888	137815934	Distal Intergenic
chr1	137821754	137823053	Distal Intergenic
chr1	137826326	137827256	Distal Intergenic
chr1	137829631	137830991	Distal Intergenic
chr1	137834587	137835440	Distal Intergenic
chr1	137837418	137838534	Distal Intergenic
chr1	137838693	137839769	Distal Intergenic
chr1	137840554	137840823	Distal Intergenic
chr1	138022668	138024635	Distal Intergenic
chr1	138026186	138026674	Distal Intergenic
chr1	138031721	138032153	Distal Intergenic
chr1	138033440	138034299	Distal Intergenic
chr1	138036246	138036445	Distal Intergenic
chr1	138036731	138037823	Distal Intergenic
chr1	138056572	138058110	Distal Intergenic
chr1	138061303	138061509	Distal Intergenic
chr1	138120320	138121453	Distal Intergenic
chr1	138124974	138127697	Distal Intergenic
chr1	138224385	138224606	Distal Intergenic

chr1	138224740	138227435	Distal Intergenic
chr1	138240311	138240759	Distal Intergenic
chr1	138241235	138241434	Distal Intergenic
chr1	138241589	138242702	Distal Intergenic
chr1	138252041	138252832	Distal Intergenic
chr1	138296411	138296761	Distal Intergenic
chr1	138297803	138298433	Distal Intergenic
chr1	138304471	138307795	Distal Intergenic
chr1	138311490	138311727	Distal Intergenic
chr1	138324052	138324411	Distal Intergenic
chr1	138492285	138493318	Distal Intergenic
chr1	138582287	138582518	Distal Intergenic
chr1	138592014	138592405	Distal Intergenic
chr1	138592556	138592880	Distal Intergenic
chr1	138593040	138593275	Distal Intergenic
chr1	138976047	138976266	Distal Intergenic
chr1	140225657	140225946	Distal Intergenic
chr1	140244384	140246220	Distal Intergenic
chr1	140246346	140246545	Distal Intergenic
chr1	140249228	140249748	Distal Intergenic
chr1	140250132	140250366	Distal Intergenic
chr1	140252436	140253806	Distal Intergenic
chr1	140391570	140392467	Distal Intergenic
chr1	140403441	140403815	Distal Intergenic
chr1	140407385	140408818	Distal Intergenic
chr1	140411957	140412744	Distal Intergenic
chr1	140432614	140434215	Distal Intergenic
chr1	140467677	140468477	Distal Intergenic
chr1	140514454	140514670	Distal Intergenic
chr1	140515266	140515575	Distal Intergenic
chr1	140553089	140553298	Distal Intergenic
chr1	141350644	141351072	Distal Intergenic
chr1	143141332	143141594	Intron
chr1	145383919	145384620	Intron
chr1	145389268	145389839	Intron
chr1	145549429	145549851	Promoter
chr1	145788805	145789062	Promoter
chr1	147804841	147805158	Intron
chr1	148204113	148204849	Intron
chr1	149649113	149650555	Exon
chr1	149898756	149899757	Promoter
chr1	149932381	149932623	Intron
chr1	150640840	150641121	Intron
chr1	151908215	151908896	Distal Intergenic

chr1	151911989	151912191	Distal Intergenic
chr1	151944886	151945205	Distal Intergenic
chr1	151945378	151947221	Distal Intergenic
chr1	151962208	151963158	Intron
chr1	151977411	151979685	Intron
chr1	151997949	152000583	Intron
chr1	152011139	152012736	Intron
chr1	152016599	152018136	Distal Intergenic
chr1	152044570	152044856	Distal Intergenic
chr1	152065652	152066064	Distal Intergenic
chr1	152098450	152099327	Intron
chr1	152240404	152240779	Distal Intergenic
chr1	152240852	152241143	Distal Intergenic
chr1	153019068	153019338	Distal Intergenic
chr1	153027894	153028227	Downstream
chr1	153028302	153028654	3' UTR
chr1	153073400	153076467	Intron
chr1	153136257	153137044	Distal Intergenic
chr1	153192188	153192421	Distal Intergenic
chr1	153192675	153192894	Distal Intergenic
chr1	153192967	153193368	Distal Intergenic
chr1	153194285	153194535	Distal Intergenic
chr1	153275664	153276960	Exon
chr1	153800275	153800794	Exon
chr1	153800885	153802076	Intron
chr1	153809813	153810019	Intron
chr1	153891695	153892206	Intron
chr1	153900231	153900918	Downstream
chr1	153908120	153908370	Intron
chr1	153908531	153909913	Exon
chr1	153909997	153911375	Exon
chr1	154973330	154974778	Promoter
chr1	154975143	154975378	Promoter
chr1	154975566	154978783	Promoter
chr1	154979485	154979697	Exon
chr1	154981452	154981916	Intron
chr1	155021801	155023513	Promoter
chr1	155024539	155026137	Promoter
chr1	155027265	155028374	5' UTR
chr1	155028485	155028964	5' UTR
chr1	155029651	155030325	Exon
chr1	155032014	155033216	Exon
chr1	155033304	155033614	Exon
chr1	155034015	155034454	3' UTR

chr1	155045340	155045733	Intron
chr1	155046308	155046724	Intron
chr1	155047411	155051997	Promoter
chr1	155052062	155055120	Promoter
chr1	155147277	155147476	Promoter
chr1	155180287	155181021	Exon
chr1	155389606	155390946	Intron
chr1	155412664	155413044	Intron
chr1	155413146	155413433	Intron
chr1	155419470	155420440	Intron
chr1	155432490	155432698	Intron
chr1	155432837	155433329	Intron
chr1	155912124	155913040	Promoter
chr1	156897821	156898294	Intron
chr1	156899370	156900538	Exon
chr1	156903507	156903786	Downstream
chr1	156919043	156919301	Promoter
chr1	156919753	156921934	Promoter
chr1	156921987	156922348	Intron
chr1	156944679	156945686	Intron
chr1	156985936	156986542	Intron
chr1	157062115	157065135	3' UTR
chr1	157787956	157789475	Promoter
chr1	157882567	157882843	Distal Intergenic
chr1	157882936	157883293	Distal Intergenic
chr1	157883341	157885135	Distal Intergenic
chr1	157887774	157888941	Distal Intergenic
chr1	158256401	158257386	Distal Intergenic
chr1	158407472	158407744	Distal Intergenic
chr1	158437420	158437660	Distal Intergenic
chr1	158520381	158521021	Distal Intergenic
chr1	158523198	158524123	Distal Intergenic
chr1	158528322	158529530	Downstream
chr1	158544782	158548486	Downstream
chr1	158548557	158548876	Promoter
chr1	158549911	158550129	Promoter
chr1	158590166	158590577	Exon
chr1	158597629	158598164	Intron
chr1	158604358	158605370	Exon
chr1	158607089	158609046	Exon
chr1	158609236	158610924	Exon
chr1	158844317	158844529	Distal Intergenic
chr1	158844976	158845720	Distal Intergenic
chr1	159162461	159163366	Exon

chr1	159178460	159178744	Distal Intergenic
chr1	159185939	159186149	Distal Intergenic
chr1	159186560	159187245	Distal Intergenic
chr1	159206080	159207033	Distal Intergenic
chr1	159250248	159251723	Distal Intergenic
chr1	159273040	159274697	Exon
chr1	159309450	159311764	Distal Intergenic
chr1	159335787	159336001	Intron
chr1	159370310	159370859	Intron
chr1	159389661	159390214	Intron
chr1	161182947	161183424	Exon
chr1	161185382	161185667	Promoter
chr1	161186257	161186494	Intron
chr1	161186530	161186763	Intron
chr1	161186806	161187099	Intron
chr1	161970953	161972134	Intron
chr1	161974231	161974641	Intron
chr1	161989998	161990210	Intron
chr1	161990310	161990827	Intron
chr1	162104864	162105420	Intron
chr1	162134341	162134940	Intron
chr1	162141192	162141391	Intron
chr1	162141468	162142304	Intron
chr1	162143269	162143708	Intron
chr1	162193092	162194923	Intron
chr1	162205170	162205923	Intron
chr1	162299904	162301591	Intron
chr1	162450914	162451150	Distal Intergenic
chr1	162451206	162451517	Distal Intergenic
chr1	162451566	162451867	Distal Intergenic
chr1	162525995	162528014	Distal Intergenic
chr1	162837037	162837315	Intron
chr1	162964314	162964513	Distal Intergenic
chr1	162965344	162968943	Distal Intergenic
chr1	162969074	162970840	Distal Intergenic
chr1	162971167	162971403	Distal Intergenic
chr1	163001123	163001969	Distal Intergenic
chr1	163061790	163062012	Distal Intergenic
chr1	163174367	163175181	Intron
chr1	163178537	163178922	Intron
chr1	163179070	163179575	Intron
chr1	163179752	163180200	Intron
chr1	163180334	163180824	Intron
chr1	163180910	163181137	Intron

chr1	163198255	163198507	Intron
chr1	163412159	163412726	Distal Intergenic
chr1	163537302	163537996	Distal Intergenic
chr1	163805420	163806497	Distal Intergenic
chr1	163825701	163827203	Distal Intergenic
chr1	163836988	163838851	Distal Intergenic
chr1	163843249	163844446	Distal Intergenic
chr1	163844662	163844861	Distal Intergenic
chr1	163845909	163846131	Distal Intergenic
chr1	164056216	164056453	Distal Intergenic
chr1	164094808	164095039	Distal Intergenic
chr1	164095161	164095457	Distal Intergenic
chr1	164172936	164174504	Distal Intergenic
chr1	164225037	164226859	Distal Intergenic
chr1	164227137	164227389	Distal Intergenic
chr1	164489772	164491056	Distal Intergenic
chr1	164669648	164670040	Intron
chr1	164670095	164670371	Intron
chr1	164670414	164670674	Intron
chr1	165043727	165044075	Distal Intergenic
chr1	165044281	165045252	Distal Intergenic
chr1	165182313	165182983	Promoter
chr1	165183019	165183843	Promoter
chr1	165203061	165203940	Intron
chr1	165204724	165205082	Intron
chr1	165215179	165215794	Intron
chr1	165310148	165310905	Intron
chr1	165311033	165311368	Intron
chr1	165312252	165313214	Intron
chr1	165313306	165313976	Intron
chr1	165333872	165334106	Distal Intergenic
chr1	165491109	165492986	Exon
chr1	166179577	166181047	Distal Intergenic
chr1	166224397	166224813	Distal Intergenic
chr1	166224865	166225087	Distal Intergenic
chr1	166237179	166237613	Distal Intergenic
chr1	166817870	166819336	Exon
chr1	166923553	166923823	Intron
chr1	167010351	167010668	Distal Intergenic
chr1	167220970	167222497	Intron
chr1	167231913	167234190	Intron
chr1	167516075	167516824	Intron
chr1	167572776	167573144	Distal Intergenic
chr1	167915793	167916723	Intron

chr1	167916775	167917021	Intron
chr1	168898581	168898780	Distal Intergenic
chr1	168926879	168927341	Distal Intergenic
chr1	168949914	168950531	Distal Intergenic
chr1	169113675	169114496	Intron
chr1	169172777	169173136	Intron
chr1	169173182	169173530	Intron
chr1	169198662	169200265	Exon
chr1	169200944	169201310	Intron
chr1	169219012	169219248	Intron
chr1	170208553	170209553	Distal Intergenic
chr1	170253325	170255024	Promoter
chr1	170258176	170259520	Distal Intergenic
chr1	170264579	170266423	Distal Intergenic
chr1	170266624	170267215	Distal Intergenic
chr1	170267265	170268762	Distal Intergenic
chr1	170268811	170269246	Distal Intergenic
chr1	170292147	170293468	Distal Intergenic
chr1	170321009	170321272	Distal Intergenic
chr1	170321391	170322969	Distal Intergenic
chr1	170356140	170358494	Distal Intergenic
chr1	170358557	170361971	Distal Intergenic
chr1	170362643	170362867	Distal Intergenic
chr1	170363868	170364067	Distal Intergenic
chr1	170364236	170365903	Distal Intergenic
chr1	170431947	170432999	Intron
chr1	170989683	170989955	Intron
chr1	171488499	171488759	Intron
chr1	171899596	171900334	Intron
chr1	172118857	172119309	Intron
chr1	172145191	172145508	Intron
chr1	172700838	172701052	Distal Intergenic
chr1	172974297	172974597	Distal Intergenic
chr1	172975293	172975759	Distal Intergenic
chr1	172976221	172977037	Distal Intergenic
chr1	173177006	173177219	Promoter
chr1	173200810	173201340	Downstream
chr1	173224850	173225158	Intron
chr1	173258563	173259490	Intron
chr1	173305481	173309085	Intron
chr1	173314011	173314685	Intron
chr1	173314739	173315033	Intron
chr1	173315076	173315343	Intron
chr1	173315400	173315750	Intron

chr1	173367660	173370337	Intron
chr1	173370648	173370849	Intron
chr1	173371102	173371675	Intron
chr1	173372032	173372361	Intron
chr1	173375111	173375520	Intron
chr1	173399239	173401776	Intron
chr1	173403049	173404632	Intron
chr1	173408057	173408843	Intron
chr1	173408941	173409238	Intron
chr1	173433313	173433538	Intron
chr1	173433611	173433877	Intron
chr1	173948911	173949342	Intron
chr1	173949565	173950083	Exon
chr1	174012290	174012868	Distal Intergenic
chr1	174012919	174013340	Distal Intergenic
chr1	174057242	174057441	Distal Intergenic
chr1	174078036	174079222	Distal Intergenic
chr1	174121278	174121851	Distal Intergenic
chr1	174172580	174172883	Intron
chr1	174219892	174220209	Promoter
chr1	174240487	174241752	5' UTR
chr1	174242605	174242805	Intron
chr1	174341354	174342231	Intron
chr1	174353301	174354020	Intron
chr1	174371893	174372103	Intron
chr1	174372281	174372758	Intron
chr1	174429832	174432427	Intron
chr1	174432524	174433434	Intron
chr1	174433471	174433923	Intron
chr1	174487697	174487910	Intron
chr1	174488054	174488513	Intron
chr1	174488577	174488961	Intron
chr1	174495660	174495907	Intron
chr1	174495991	174496229	Intron
chr1	174496496	174496736	Intron
chr1	174609574	174611614	Intron
chr1	175696510	175697949	Intron
chr1	175776425	175777135	Distal Intergenic
chr1	175809904	175810337	Distal Intergenic
chr1	175842274	175842571	Distal Intergenic
chr1	175842631	175844076	Distal Intergenic
chr1	175872411	175872646	Distal Intergenic
chr1	175912662	175913707	Downstream
chr1	178757984	178758427	Intron

chr1	178762784	178762983	Intron
chr1	179316137	179316467	Intron
chr1	179648832	179649996	Intron
chr1	179678257	179678521	Distal Intergenic
chr1	179724933	179725308	Intron
chr1	179725399	179725614	Intron
chr1	179725806	179726132	Intron
chr1	180227733	180229178	Intron
chr1	180248185	180248405	Distal Intergenic
chr1	180248445	180250644	Distal Intergenic
chr1	180251060	180251301	Distal Intergenic
chr1	180265762	180266978	Intron
chr1	180267442	180268494	Intron
chr1	180335857	180336950	Intron
chr1	180971546	180973618	Exon
chr1	181733400	181733608	Intron
chr1	181778185	181778706	Distal Intergenic
chr1	181900204	181900797	Distal Intergenic
chr1	181944670	181944869	Distal Intergenic
chr1	181944920	181946589	Distal Intergenic
chr1	182242681	182243014	Distal Intergenic
chr1	182258805	182259927	Distal Intergenic
chr1	182843953	182844181	Exon
chr1	182871712	182873859	Exon
chr1	182873971	182875408	Exon
chr1	182885674	182886557	Intron
chr1	182896523	182896760	Intron
chr1	182896827	182898011	Intron
chr1	182945295	182947264	Distal Intergenic
chr1	183056827	183057065	Intron
chr1	183074480	183074717	Intron
chr1	183161271	183161470	Intron
chr1	183890067	183890302	Intron
chr1	183890419	183890709	Intron
chr1	183915914	183916135	Promoter
chr1	183916195	183916563	Promoter
chr1	183927590	183927876	Intron
chr1	183943415	183943683	Intron
chr1	183943753	183944270	Exon
chr1	183944381	183949724	5' UTR
chr1	183950782	183952830	Intron
chr1	183982686	183984124	Intron
chr1	183987442	183988872	Intron
chr1	183988933	183989630	Intron

chr1	183989727	183990137	Intron
chr1	183996442	183999530	Intron
chr1	184004120	184005496	Intron
chr1	184017722	184023556	Promoter
chr1	184024125	184024918	Intron
chr1	184024965	184025486	Intron
chr1	184025629	184025858	Intron
chr1	184119568	184120519	Distal Intergenic
chr1	184445310	184445664	Intron
chr1	184445713	184447367	5' UTR
chr1	184458304	184458840	Intron
chr1	184472162	184472428	Intron
chr1	184472475	184472748	Intron
chr1	184472789	184473149	Intron
chr1	184473306	184473513	Intron
chr1	184522347	184522596	Intron
chr1	184874398	184875633	Intron
chr1	184886167	184886471	Intron
chr1	185493597	185493930	Distal Intergenic
chr1	185602667	185602866	Distal Intergenic
chr1	185688710	185689822	Distal Intergenic
chr1	185728973	185729389	Intron
chr1	185729447	185730075	Intron
chr1	185737664	185738630	Intron
chr1	185740504	185740703	Intron
chr1	185741305	185741856	Intron
chr1	185744866	185745073	Intron
chr1	185745278	185745999	Intron
chr1	185754211	185754877	Intron
chr1	185766415	185766799	Intron
chr1	185768587	185769630	Intron
chr1	185787474	185788055	Intron
chr1	185858264	185858463	Intron
chr1	185875412	185875823	Intron
chr1	185876091	185876381	Intron
chr1	185881047	185881456	Intron
chr1	185900255	185900518	Intron
chr1	185900819	185901424	Intron
chr1	185907736	185909196	Intron
chr1	185990761	185990992	Intron
chr1	186631933	186633327	Distal Intergenic
chr1	186646970	186647279	Intron
chr1	186976503	186977155	Distal Intergenic
chr1	188285434	188285745	Distal Intergenic

chr1	189039131	189039340	Distal Intergenic
chr1	190743493	190743916	Intron
chr1	190743955	190744650	Intron
chr1	190744791	190745004	Intron
chr1	191025485	191025956	Distal Intergenic
chr1	191027784	191029589	Distal Intergenic
chr1	191038225	191039959	Distal Intergenic
chr1	191183223	191184402	Distal Intergenic
chr1	191551544	191551781	Distal Intergenic
chr1	191578690	191579741	Distal Intergenic
chr1	191638228	191638919	Distal Intergenic
chr1	193925090	193925355	Distal Intergenic
chr1	194237541	194238144	Distal Intergenic
chr1	194268410	194269513	Distal Intergenic
chr1	194271300	194274431	Distal Intergenic
chr1	194274660	194274859	Distal Intergenic
chr1	194276064	194276310	Distal Intergenic
chr1	194571583	194571782	Distal Intergenic
chr1	194605037	194605860	Distal Intergenic
chr1	194612143	194612541	Distal Intergenic
chr1	194633106	194633737	Distal Intergenic
chr1	194654950	194655756	Distal Intergenic
chr1	194657679	194657891	Distal Intergenic
chr1	194956597	194958137	Distal Intergenic
chr1	194965480	194968965	Distal Intergenic
chr1	194975952	194976311	Distal Intergenic
chr1	194976759	194978013	Distal Intergenic
chr1	194978214	194978484	Distal Intergenic
chr1	194978846	194979058	Distal Intergenic
chr1	194979419	194980601	Distal Intergenic
chr1	194981088	194981651	Distal Intergenic
chr1	195124154	195124455	Distal Intergenic
chr1	195125992	195127138	Distal Intergenic
chr1	195127271	195128481	Distal Intergenic
chr1	195224503	195227194	Distal Intergenic
chr1	195330909	195331303	Distal Intergenic
chr1	195331937	195333443	Distal Intergenic
chr1	195333498	195340310	Distal Intergenic
chr1	195340379	195341176	Distal Intergenic
chr1	195341256	195341456	Distal Intergenic
chr1	195342158	195349726	Distal Intergenic
chr1	195352477	195353480	Distal Intergenic
chr1	195371747	195371946	Distal Intergenic
chr1	195372240	195374242	Distal Intergenic

chr1	195374506	195377832	Distal Intergenic
chr1	195378098	195378328	Distal Intergenic
chr1	195379966	195381297	Distal Intergenic
chr1	195381396	195381618	Distal Intergenic
chr1	195387658	195389450	Distal Intergenic
chr1	195389643	195391713	Distal Intergenic
chr1	195392540	195396112	Distal Intergenic
chr1	195400515	195401859	Distal Intergenic
chr1	195414361	195414899	Distal Intergenic
chr1	195415104	195415324	Distal Intergenic
chr1	196478399	196478600	Intron
chr1	196501758	196502008	Intron
chr1	196502171	196503057	Intron
chr1	196503284	196503580	Intron
chr1	196591035	196591301	Distal Intergenic
chr1	196955962	196956374	Intron
chr1	196956486	196957733	Intron
chr10	3086226	3086462	Distal Intergenic
chr10	3087084	3088500	Distal Intergenic
chr10	3133814	3134201	Intron
chr10	3156566	3157629	Intron
chr10	3159794	3160726	5' UTR
chr10	3165652	3166104	Intron
chr10	3501202	3502355	Intron
chr10	3502436	3502644	Intron
chr10	3503019	3504714	Intron
chr10	3521369	3522069	Intron
chr10	4522274	4522840	Distal Intergenic
chr10	4767718	4768254	Distal Intergenic
chr10	5057317	5057560	Intron
chr10	5137767	5138390	Intron
chr10	5235430	5235880	Distal Intergenic
chr10	5259996	5261138	3' UTR
chr10	5437503	5438173	Intron
chr10	5724745	5726142	Promoter
chr10	5733763	5735140	Exon
chr10	5735360	5735599	Intron
chr10	5913141	5913412	Intron
chr10	5913532	5913912	Intron
chr10	5951033	5951273	Exon
chr10	6224862	6225835	Intron
chr10	6238151	6239515	Intron
chr10	6370776	6371133	Exon
chr10	6371181	6371474	Exon

chr10	6372169	6372682	Intron
chr10	6372780	6373394	Exon
chr10	6403970	6405544	Distal Intergenic
chr10	6411264	6412474	Distal Intergenic
chr10	6481955	6483500	Intron
chr10	6483680	6484427	Exon
chr10	6509903	6512293	Intron
chr10	6879650	6880642	Exon
chr10	6961113	6963744	Distal Intergenic
chr10	6985714	6985951	Distal Intergenic
chr10	7108807	7109678	Distal Intergenic
chr10	7383173	7383741	Intron
chr10	7400181	7401995	Intron
chr10	7445936	7446767	Intron
chr10	7496253	7497645	Distal Intergenic
chr10	7507292	7508001	Distal Intergenic
chr10	7512054	7513266	Distal Intergenic
chr10	7581388	7581588	Distal Intergenic
chr10	7632149	7632362	Intron
chr10	7674487	7674693	Intron
chr10	7675014	7675299	Intron
chr10	7675392	7676052	Intron
chr10	7676143	7676940	Intron
chr10	7996164	7997903	Intron
chr10	8060868	8061398	Distal Intergenic
chr10	8125278	8125495	Distal Intergenic
chr10	8211614	8212022	Distal Intergenic
chr10	8225585	8226378	Distal Intergenic
chr10	8467367	8467633	Distal Intergenic
chr10	8481784	8482443	Distal Intergenic
chr10	8487679	8489583	Distal Intergenic
chr10	8491721	8491920	Distal Intergenic
chr10	8531093	8532222	Distal Intergenic
chr10	8532422	8532704	Distal Intergenic
chr10	8593150	8593999	Distal Intergenic
chr10	8605902	8606115	Distal Intergenic
chr10	8606186	8606435	Distal Intergenic
chr10	8609681	8609942	Distal Intergenic
chr10	8609981	8610929	Distal Intergenic
chr10	8698108	8701254	Exon
chr10	8991937	8992409	Distal Intergenic
chr10	9002367	9002599	Distal Intergenic
chr10	9215978	9216584	Distal Intergenic
chr10	9216746	9217225	Distal Intergenic

chr10	9248609	9250000	Distal Intergenic
chr10	9250062	9250295	Distal Intergenic
chr10	9264379	9265197	Distal Intergenic
chr10	9318701	9318984	Distal Intergenic
chr10	9319163	9320350	Distal Intergenic
chr10	9350097	9351467	Distal Intergenic
chr10	9360205	9360413	Distal Intergenic
chr10	9360473	9360721	Distal Intergenic
chr10	9601553	9601977	Distal Intergenic
chr10	9621076	9622170	Distal Intergenic
chr10	9779289	9779605	Distal Intergenic
chr10	9971652	9972474	Distal Intergenic
chr10	9972592	9973029	Distal Intergenic
chr10	10117658	10118103	Distal Intergenic
chr10	10263988	10264454	Distal Intergenic
chr10	10264835	10265927	Distal Intergenic
chr10	10274443	10276207	Distal Intergenic
chr10	10276258	10277117	Distal Intergenic
chr10	10277214	10277812	Distal Intergenic
chr10	10917452	10917851	Distal Intergenic
chr10	10917888	10918216	Distal Intergenic
chr10	11000233	11000956	Distal Intergenic
chr10	11001567	11002500	Distal Intergenic
chr10	12324700	12325206	Distal Intergenic
chr10	12414541	12415054	Intron
chr10	12445915	12446145	Intron
chr10	12446191	12446598	Intron
chr10	12507484	12508518	Intron
chr10	12530346	12530629	Intron
chr10	12530834	12531249	Intron
chr10	12547124	12547580	Intron
chr10	12586773	12588081	Intron
chr10	12588140	12588968	Intron
chr10	12594625	12595200	Intron
chr10	12596021	12596352	Intron
chr10	12598169	12598573	Intron
chr10	12598642	12599165	Intron
chr10	12599508	12599720	Intron
chr10	12599788	12600479	Intron
chr10	12612804	12613743	Intron
chr10	12631941	12632651	Intron
chr10	12642623	12642822	Intron
chr10	12683043	12683625	Intron
chr10	12727843	12728124	Intron

chr10	12860069	12860275	Intron
chr10	12860443	12860676	Intron
chr10	12912360	12912994	Distal Intergenic
chr10	13089352	13090377	Intron
chr10	13186487	13187045	Distal Intergenic
chr10	13272571	13272901	Intron
chr10	13273029	13273634	Intron
chr10	13296328	13297979	Distal Intergenic
chr10	13315042	13315242	Distal Intergenic
chr10	13315445	13316580	Distal Intergenic
chr10	13379562	13380093	Promoter
chr10	13383783	13384115	Intron
chr10	13403441	13404654	Distal Intergenic
chr10	13408014	13409053	Distal Intergenic
chr10	13495248	13495480	Intron
chr10	13512176	13512576	Intron
chr10	13512780	13513068	Intron
chr10	13552195	13553089	Distal Intergenic
chr10	13589076	13589614	Distal Intergenic
chr10	13685682	13685908	3' UTR
chr10	13686490	13686823	3' UTR
chr10	13737486	13737924	Intron
chr10	14027426	14027742	Intron
chr10	14031805	14032304	Intron
chr10	14032722	14034677	Intron
chr10	14104437	14107612	Intron
chr10	14247380	14248278	Intron
chr10	14382065	14382323	Intron
chr10	14384422	14385415	Intron
chr10	14424516	14425320	Intron
chr10	16227694	16228007	Distal Intergenic
chr10	16264640	16265909	Distal Intergenic
chr10	16272704	16273286	Distal Intergenic
chr10	16598032	16598598	Distal Intergenic
chr10	16731342	16733923	Intron
chr10	16734009	16734263	Intron
chr10	16734439	16734871	Intron
chr10	16754938	16755188	Intron
chr10	16971998	16973294	Intron
chr10	17018308	17018931	Intron
chr10	17067150	17068216	Intron
chr10	17068417	17068683	Intron
chr10	17268994	17269918	Promoter
chr10	17304111	17306233	Distal Intergenic

chr10	17443097	17443416	Intron
chr10	17471570	17473568	Intron
chr10	17478885	17481327	Intron
chr10	17491909	17492463	Intron
chr10	17496195	17497179	Promoter
chr10	17588802	17589882	Distal Intergenic
chr10	17649915	17651476	Intron
chr10	17651523	17651761	Intron
chr10	17725135	17726036	Intron
chr10	17728272	17728504	Intron
chr10	17741664	17742361	5' UTR
chr10	17742594	17743247	Intron
chr10	17828604	17831673	Intron
chr10	18025001	18025297	Intron
chr10	18043089	18044328	Intron
chr10	18049089	18049994	Intron
chr10	18070198	18071418	Intron
chr10	18158546	18158840	Intron
chr10	18183037	18183318	Exon
chr10	18190949	18192360	Exon
chr10	18200373	18201180	Distal Intergenic
chr10	18564933	18567905	Intron
chr10	18567982	18571126	Intron
chr10	18571712	18572254	Intron
chr10	18578748	18581665	Intron
chr10	18582890	18583899	Intron
chr10	18595393	18596851	Intron
chr10	18604150	18604380	Intron
chr10	18707217	18707494	Intron
chr10	18707554	18708069	Intron
chr10	18728098	18730826	Intron
chr10	18731530	18731729	Intron
chr10	18731770	18732938	Intron
chr10	18733178	18733494	Intron
chr10	18746414	18746733	Intron
chr10	18746815	18747014	Intron
chr10	18747617	18748954	Intron
chr10	18753993	18755827	Intron
chr10	18763588	18763805	Intron
chr10	18765353	18767393	Intron
chr10	18784808	18785906	Intron
chr10	18801041	18803630	Exon
chr10	18811102	18812288	Intron
chr10	18838592	18838953	Intron

chr10	18887702	18888067	Intron
chr10	18896292	18896665	Intron
chr10	18896742	18897860	Intron
chr10	18902766	18903086	Intron
chr10	19183556	19185574	Distal Intergenic
chr10	19217802	19218882	Distal Intergenic
chr10	19219251	19219506	Distal Intergenic
chr10	19797084	19797606	Intron
chr10	19810665	19811606	Intron
chr10	19823992	19824211	Intron
chr10	19824872	19825145	Intron
chr10	19869362	19869629	Intron
chr10	19869785	19870098	Intron
chr10	19870154	19870399	Intron
chr10	19878896	19879733	Intron
chr10	19889073	19890511	Intron
chr10	19893674	19894414	Intron
chr10	19898340	19899427	Distal Intergenic
chr10	20031374	20032538	Distal Intergenic
chr10	20032823	20033428	Distal Intergenic
chr10	20287835	20290135	Intron
chr10	20290318	20290568	Intron
chr10	20290611	20292645	Exon
chr10	20377244	20378334	Intron
chr10	20839029	20839230	Downstream
chr10	20892813	20893793	Distal Intergenic
chr10	21015791	21016595	Distal Intergenic
chr10	21018891	21019116	Distal Intergenic
chr10	21019516	21022120	Distal Intergenic
chr10	21024124	21024416	Distal Intergenic
chr10	21024463	21024757	Distal Intergenic
chr10	21053730	21056364	Distal Intergenic
chr10	21062354	21063076	Distal Intergenic
chr10	21118817	21119188	Intron
chr10	21493369	21493595	Distal Intergenic
chr10	21509775	21511065	Distal Intergenic
chr10	21548259	21550077	Distal Intergenic
chr10	21552930	21554547	Distal Intergenic
chr10	21558636	21560130	Distal Intergenic
chr10	21565941	21568226	Distal Intergenic
chr10	21714154	21714353	Distal Intergenic
chr10	21714516	21715063	Distal Intergenic
chr10	22415377	22415663	Distal Intergenic
chr10	22416853	22417053	Distal Intergenic

chr10	22418054	22418770	Distal Intergenic
chr10	22451064	22451863	Distal Intergenic
chr10	22876810	22877067	Intron
chr10	22896429	22898079	Exon
chr10	22904782	22905994	Intron
chr10	23502957	23505773	Distal Intergenic
chr10	23506125	23506808	Distal Intergenic
chr10	23506863	23508582	Distal Intergenic
chr10	23929386	23931466	Distal Intergenic
chr10	24002107	24002406	Intron
chr10	24002447	24002659	Intron
chr10	24165521	24166765	Intron
chr10	24506976	24507175	Intron
chr10	24507260	24507877	Intron
chr10	24602353	24604458	Intron
chr10	24604621	24604857	Intron
chr10	24692570	24693806	Intron
chr10	24704353	24705120	Intron
chr10	25145894	25147117	Exon
chr10	25152947	25153659	Intron
chr10	25508495	25508748	Intron
chr10	25680938	25681401	Intron
chr10	25968998	25970047	Intron
chr10	25970208	25970431	Intron
chr10	26077951	26079900	Distal Intergenic
chr10	26093536	26093745	Distal Intergenic
chr10	26284091	26284970	Intron
chr10	26387770	26388215	Intron
chr10	26388315	26388833	Intron
chr10	26398682	26398989	Intron
chr10	26399190	26399547	Intron
chr10	26482196	26482635	Exon
chr10	26492306	26492512	Intron
chr10	26615440	26615987	Distal Intergenic
chr10	26715884	26716176	Distal Intergenic
chr10	27373887	27374182	Intron
chr10	27374343	27374581	Intron
chr10	27702846	27706131	Promoter
chr10	27706193	27709570	Distal Intergenic
chr10	27795024	27796166	Intron
chr10	27806890	27807918	Intron
chr10	28023587	28024787	Exon
chr10	28863252	28863932	Intron
chr10	28864093	28864292	Intron

chr10	29427497	29427726	Distal Intergenic
chr10	29427766	29429014	Distal Intergenic
chr10	29429234	29429654	Distal Intergenic
chr10	29917917	29918313	Intron
chr10	29919943	29920294	Intron
chr10	30320211	30320733	Intron
chr10	30327960	30328372	Intron
chr10	30337797	30338145	Intron
chr10	30338325	30338951	Intron
chr10	31252115	31255507	Intron
chr10	31257345	31259197	Intron
chr10	31260127	31261086	Intron
chr10	31272674	31274072	5' UTR
chr10	31277208	31277431	Intron
chr10	31327141	31327973	Distal Intergenic
chr10	31328125	31328378	Distal Intergenic
chr10	31328433	31328714	Distal Intergenic
chr10	31328894	31329097	Distal Intergenic
chr10	31587475	31588323	Distal Intergenic
chr10	32703967	32706018	Distal Intergenic
chr10	33738608	33739225	Distal Intergenic
chr10	33906178	33907411	Distal Intergenic
chr10	33914354	33914559	Distal Intergenic
chr10	33916162	33918208	Distal Intergenic
chr10	33918903	33919102	Distal Intergenic
chr10	33926135	33927661	Distal Intergenic
chr10	33927997	33928274	Distal Intergenic
chr10	33928399	33928663	Distal Intergenic
chr10	33948998	33949881	Distal Intergenic
chr10	33949965	33950202	Distal Intergenic
chr10	34202987	34204625	Distal Intergenic
chr10	34226784	34228800	Distal Intergenic
chr10	34252134	34253165	Distal Intergenic
chr10	34316017	34316375	Distal Intergenic
chr10	34316450	34317006	Distal Intergenic
chr10	34338238	34338793	Distal Intergenic
chr10	36421764	36423683	Distal Intergenic
chr10	36694119	36694745	Distal Intergenic
chr10	36856995	36858797	Distal Intergenic
chr10	36858836	36860092	Distal Intergenic
chr10	36860174	36860793	Distal Intergenic
chr10	36861304	36861659	Distal Intergenic
chr10	37050288	37050965	Distal Intergenic
chr10	37054225	37057891	Distal Intergenic

chr10	37080328	37082005	Distal Intergenic
chr10	37087068	37088325	Distal Intergenic
chr10	37176784	37177167	Distal Intergenic
chr10	37177220	37177628	Distal Intergenic
chr10	37320137	37320835	Distal Intergenic
chr10	37322816	37323299	Distal Intergenic
chr10	37333767	37333979	Distal Intergenic
chr10	37523160	37523370	Distal Intergenic
chr10	37966644	37966856	Distal Intergenic
chr10	37967019	37967225	Distal Intergenic
chr10	38714178	38714919	Distal Intergenic
chr10	39135407	39135749	Distal Intergenic
chr10	39290313	39290724	Distal Intergenic
chr10	39290761	39291198	Distal Intergenic
chr10	39292435	39293446	Distal Intergenic
chr10	39332042	39334274	Distal Intergenic
chr10	39966400	39966626	Distal Intergenic
chr10	39976686	39979476	Distal Intergenic
chr10	39981662	39984452	Distal Intergenic
chr10	39987757	39989158	Distal Intergenic
chr10	40018187	40018766	Distal Intergenic
chr10	40018975	40019190	Distal Intergenic
chr10	40020071	40020372	Distal Intergenic
chr10	40020423	40020920	Distal Intergenic
chr10	40021077	40021688	Distal Intergenic
chr10	40021743	40022711	Distal Intergenic
chr10	40036692	40037288	Distal Intergenic
chr10	40057589	40059476	Distal Intergenic
chr10	40067566	40068566	Distal Intergenic
chr10	40090248	40092855	Distal Intergenic
chr10	40099244	40099483	Distal Intergenic
chr10	40136647	40137741	Distal Intergenic
chr10	40162005	40162508	Distal Intergenic
chr10	40162907	40164029	Distal Intergenic
chr10	40230177	40232087	Distal Intergenic
chr10	40241370	40241704	Distal Intergenic
chr10	40602136	40603147	Distal Intergenic
chr10	40622892	40623176	Distal Intergenic
chr10	41238322	41238880	Distal Intergenic
chr10	41239288	41240302	Distal Intergenic
chr10	41314740	41314952	Distal Intergenic
chr10	41450369	41451797	Distal Intergenic
chr10	41600780	41602090	Distal Intergenic
chr10	41612980	41613779	Distal Intergenic

chr10	41753384	41753846	Distal Intergenic
chr10	41802606	41803065	Distal Intergenic
chr10	41806312	41806772	Distal Intergenic
chr10	41821196	41821412	Distal Intergenic
chr10	41823176	41824672	Distal Intergenic
chr10	41847668	41848096	Distal Intergenic
chr10	41848137	41848686	Distal Intergenic
chr10	41850165	41850713	Distal Intergenic
chr10	41939339	41939635	Distal Intergenic
chr10	41941844	41942129	Distal Intergenic
chr10	41942173	41942509	Distal Intergenic
chr10	41967647	41967864	Distal Intergenic
chr10	41993987	41994653	Distal Intergenic
chr10	41995180	41995468	Distal Intergenic
chr10	41996170	41996514	Distal Intergenic
chr10	42194344	42195183	Distal Intergenic
chr10	42195275	42195532	Distal Intergenic
chr10	42197745	42198429	Distal Intergenic
chr10	42221954	42223190	Distal Intergenic
chr10	42478605	42479201	Distal Intergenic
chr10	42479333	42480061	Distal Intergenic
chr10	42480780	42481152	Distal Intergenic
chr10	42481372	42481911	Distal Intergenic
chr10	42482112	42482904	Distal Intergenic
chr10	42500941	42501606	Distal Intergenic
chr10	42584619	42584945	Distal Intergenic
chr10	42936842	42938218	Intron
chr10	42940325	42942009	Intron
chr10	43298951	43301137	Intron
chr10	43372970	43373566	Distal Intergenic
chr10	43377363	43378126	Distal Intergenic
chr10	43404275	43404498	Distal Intergenic
chr10	43409179	43410104	Distal Intergenic
chr10	43639940	43641625	Intron
chr10	43715741	43716429	Intron
chr10	43718216	43718442	Intron
chr10	43722580	43723545	Intron
chr10	43723610	43726840	Promoter
chr10	43739748	43739966	Intron
chr10	43740006	43740545	Intron
chr10	43763351	43764240	Promoter
chr10	43798609	43799292	Distal Intergenic
chr10	43831600	43831954	Distal Intergenic
chr10	43857681	43858235	Distal Intergenic

chr10	43858286	43858485	Distal Intergenic
chr10	43987693	43987892	Distal Intergenic
chr10	44139651	44140443	Promoter
chr10	44284631	44285059	Promoter
chr10	44285151	44285611	Promoter
chr10	44435211	44437476	Promoter
chr10	44443191	44443486	Intron
chr10	44475037	44475249	Distal Intergenic
chr10	44652426	44652697	Distal Intergenic
chr10	44723000	44723369	Distal Intergenic
chr10	44786365	44786585	Distal Intergenic
chr10	44787197	44790025	Promoter
chr10	44989381	44989893	Distal Intergenic
chr10	45117390	45118645	Distal Intergenic
chr10	45301534	45302976	Distal Intergenic
chr10	45355408	45355638	Intron
chr10	45355744	45357042	Intron
chr10	45357542	45361490	Intron
chr10	45371116	45371315	Intron
chr10	46504809	46506418	Distal Intergenic
chr10	47786071	47787734	Distal Intergenic
chr10	50311604	50311840	3' UTR
chr10	50311994	50312419	Intron
chr10	50312702	50313189	Intron
chr10	51290956	51291796	Exon
chr10	51997329	51997888	Intron
chr10	51997943	51998211	Intron
chr10	52102144	52102423	Intron
chr10	52137978	52139495	Intron
chr10	53114599	53115516	Intron
chr10	53316785	53317051	Intron
chr10	53317513	53317719	Intron
chr10	53317885	53318139	Intron
chr10	53318235	53318807	Intron
chr10	54070482	54070870	Intron
chr10	55882043	55883106	Intron
chr10	56096562	56096958	Intron
chr10	56097075	56100040	Intron
chr10	56100193	56100480	Intron
chr10	56100546	56100932	Intron
chr10	56145161	56145399	Intron
chr10	56188910	56189109	Intron
chr10	56205849	56207628	Intron
chr10	56259722	56259972	Intron

chr10	56260142	56260399	Intron
chr10	56260435	56260660	Intron
chr10	56279579	56281010	Intron
chr10	56302081	56302280	Intron
chr10	56329474	56332420	Intron
chr10	56501760	56504371	Intron
chr10	56664389	56664588	Intron
chr10	56664701	56665319	Intron
chr10	56713989	56715748	Intron
chr10	57251337	57251714	Intron
chr10	57251893	57252542	Intron
chr10	57784003	57784476	Distal Intergenic
chr10	57784785	57786005	Distal Intergenic
chr10	57786343	57787281	Distal Intergenic
chr10	57793231	57795569	Distal Intergenic
chr10	57795674	57797008	Distal Intergenic
chr10	57808005	57809644	Distal Intergenic
chr10	57822779	57823055	Distal Intergenic
chr10	57823234	57823464	Distal Intergenic
chr10	57823506	57823754	Distal Intergenic
chr10	57827371	57828065	Distal Intergenic
chr10	57833022	57833442	Distal Intergenic
chr10	57833804	57834615	Distal Intergenic
chr10	57909618	57910206	Distal Intergenic
chr10	57959374	57960868	Distal Intergenic
chr10	58071638	58072661	Distal Intergenic
chr10	58076523	58077992	Distal Intergenic
chr10	58078947	58079365	Distal Intergenic
chr10	58079403	58080418	Distal Intergenic
chr10	58080617	58082104	Distal Intergenic
chr10	58113907	58115603	Downstream
chr10	58684882	58685081	Distal Intergenic
chr10	58685905	58686700	Distal Intergenic
chr10	58703696	58704730	Distal Intergenic
chr10	58706427	58707705	Distal Intergenic
chr10	58786554	58786980	Distal Intergenic
chr10	59040497	59042193	Distal Intergenic
chr10	59342514	59342883	Distal Intergenic
chr10	59361790	59364868	Distal Intergenic
chr10	59370112	59371639	Distal Intergenic
chr10	59371748	59372532	Distal Intergenic
chr10	59405977	59406646	Distal Intergenic
chr10	59410406	59413240	Distal Intergenic
chr10	59413287	59414050	Distal Intergenic

chr10	59414145	59414575	Distal Intergenic
chr10	59466103	59466361	Distal Intergenic
chr10	59471619	59471829	Distal Intergenic
chr10	59472046	59472706	Distal Intergenic
chr10	59541560	59543236	Distal Intergenic
chr10	59672291	59673528	Distal Intergenic
chr10	59692959	59694294	Distal Intergenic
chr10	59817062	59818417	Distal Intergenic
chr10	59818606	59818805	Distal Intergenic
chr10	60267866	60268095	Distal Intergenic
chr10	60268182	60268740	Distal Intergenic
chr10	60593476	60593882	Distal Intergenic
chr10	60596084	60597027	Distal Intergenic
chr10	60608609	60608827	Distal Intergenic
chr10	60609548	60610294	Distal Intergenic
chr10	60613070	60613628	Distal Intergenic
chr10	60780291	60780945	Distal Intergenic
chr10	60914308	60914634	Distal Intergenic
chr10	61032669	61033005	Intron
chr10	61044846	61046083	Intron
chr10	61051586	61052250	Intron
chr10	61090091	61090293	Intron
chr10	61111334	61112148	Promoter
chr10	61124586	61124939	Distal Intergenic
chr10	61132264	61133114	Distal Intergenic
chr10	61133152	61133399	Distal Intergenic
chr10	61143081	61143319	Distal Intergenic
chr10	61762477	61763643	Distal Intergenic
chr10	61803096	61803339	Intron
chr10	61911927	61913341	Intron
chr10	61933390	61934283	Intron
chr10	61948608	61949308	Intron
chr10	62063923	62064125	Intron
chr10	62064288	62065240	Intron
chr10	62113226	62113457	Intron
chr10	62113575	62113907	Intron
chr10	62254369	62254606	Intron
chr10	62255238	62255542	Intron
chr10	62485158	62486579	Intron
chr10	62487069	62487770	Intron
chr10	62640225	62640792	Intron
chr10	62706224	62706425	Intron
chr10	62706852	62707246	Intron
chr10	63304162	63304474	Distal Intergenic

chr10	63306642	63308586	Distal Intergenic
chr10	63486336	63486654	Intron
chr10	63538271	63540160	Distal Intergenic
chr10	64298694	64300237	Intron
chr10	65802808	65803653	Distal Intergenic
chr10	65816100	65818815	Distal Intergenic
chr10	66312457	66313324	Distal Intergenic
chr10	66326893	66327728	Distal Intergenic
chr10	66383316	66384482	Distal Intergenic
chr10	66438412	66438650	Distal Intergenic
chr10	66465520	66466735	Distal Intergenic
chr10	66472937	66474104	Distal Intergenic
chr10	66559881	66562259	Distal Intergenic
chr10	66569276	66570252	Distal Intergenic
chr10	66617907	66620818	Distal Intergenic
chr10	66647811	66650601	Distal Intergenic
chr10	66681137	66682518	Distal Intergenic
chr10	67308417	67309614	Distal Intergenic
chr10	67334696	67335857	Distal Intergenic
chr10	67361677	67362404	Distal Intergenic
chr10	67446990	67447200	Distal Intergenic
chr10	67470834	67471590	Distal Intergenic
chr10	67502327	67502550	Distal Intergenic
chr10	67502713	67503259	Distal Intergenic
chr10	67565689	67568208	Distal Intergenic
chr10	67599499	67599970	Distal Intergenic
chr10	67618473	67621297	Distal Intergenic
chr10	67628635	67629738	Distal Intergenic
chr10	67709334	67710200	Intron
chr10	67710366	67713093	Intron
chr10	67735493	67737115	Intron
chr10	67738016	67740383	Intron
chr10	67740485	67741096	Intron
chr10	67741686	67742135	Intron
chr10	67743969	67744178	Intron
chr10	67817932	67819271	Intron
chr10	67909297	67909879	Intron
chr10	68163773	68164828	Intron
chr10	68168431	68169405	Intron
chr10	68354897	68356562	Intron
chr10	68360248	68360515	Intron
chr10	68369849	68370601	Intron
chr10	68452962	68453507	Intron
chr10	68453639	68454047	Intron

chr10	68648054	68649432	Intron
chr10	68814950	68815692	Intron
chr10	68815802	68816022	Intron
chr10	68970402	68970637	Intron
chr10	69239785	69240289	Intron
chr10	69334771	69336126	Intron
chr10	69425008	69425518	Promoter
chr10	69557289	69559300	Intron
chr10	69559347	69559721	Intron
chr10	69725049	69728605	3' UTR
chr10	69729117	69729594	Intron
chr10	70057193	70057524	Promoter
chr10	70057675	70058916	Promoter
chr10	70059027	70059328	Intron
chr10	70160744	70161682	5' UTR
chr10	70261278	70263017	Intron
chr10	70729273	70729503	Intron
chr10	71330319	71330522	Downstream
chr10	72096397	72096660	Intron
chr10	72096820	72097868	Intron
chr10	72116528	72118361	Intron
chr10	72126472	72126784	Intron
chr10	72701206	72701406	Distal Intergenic
chr10	74000871	74002041	Distal Intergenic
chr10	74125826	74126060	Intron
chr10	74143052	74143277	Intron
chr10	74533172	74533371	Intron
chr10	74547999	74548815	Intron
chr10	74555896	74556634	Intron
chr10	74566289	74567493	Intron
chr10	74567627	74568966	Intron
chr10	74575372	74575586	Intron
chr10	74592320	74592684	Intron
chr10	74684789	74686237	Intron
chr10	74980291	74980692	Intron
chr10	74980746	74981083	Intron
chr10	75011022	75011251	Intron
chr10	75321700	75322032	Intron
chr10	75322578	75322863	Intron
chr10	75323097	75323451	Intron
chr10	75335191	75336666	Promoter
chr10	75341502	75342313	Intron
chr10	75343590	75344595	Intron
chr10	75361406	75361848	Distal Intergenic

chr10	75384005	75384314	Distal Intergenic
chr10	75384606	75384819	Distal Intergenic
chr10	75494800	75495170	Exon
chr10	75495483	75496170	Distal Intergenic
chr10	75725594	75726139	Distal Intergenic
chr10	75751160	75751368	Distal Intergenic
chr10	75799478	75799990	Intron
chr10	75808337	75808748	Intron
chr10	75820028	75821957	Intron
chr10	75999474	76000890	Intron
chr10	76004731	76006158	Intron
chr10	76336033	76337377	Intron
chr10	76337416	76337769	Intron
chr10	76365395	76365987	Intron
chr10	76390094	76390294	Intron
chr10	76393819	76394050	Intron
chr10	76394097	76394456	Intron
chr10	76546875	76547726	Distal Intergenic
chr10	76552274	76553413	Distal Intergenic
chr10	76574314	76574758	Distal Intergenic
chr10	76584044	76584711	Distal Intergenic
chr10	76588636	76589100	Intron
chr10	76604889	76605088	Intron
chr10	76614276	76615292	Intron
chr10	76615904	76617362	Intron
chr10	76624538	76625504	Intron
chr10	76626844	76627058	Intron
chr10	76628000	76628897	Intron
chr10	76629996	76630939	Intron
chr10	76663930	76665530	Intron
chr10	76669218	76669811	Intron
chr10	76673064	76674904	Intron
chr10	76700276	76700644	Intron
chr10	76700793	76701120	Intron
chr10	76714759	76715293	Intron
chr10	76741484	76742260	Exon
chr10	76841886	76842417	Distal Intergenic
chr10	76842486	76845757	Distal Intergenic
chr10	76849306	76851663	Downstream
chr10	76860664	76861561	Intron
chr10	76869218	76869804	Promoter
chr10	76873850	76874902	Intron
chr10	76893438	76893654	Intron
chr10	76928721	76929370	Intron

chr10	76972154	76972505	Intron
chr10	76972557	76973038	Intron
chr10	77032413	77033852	Distal Intergenic
chr10	77044808	77045007	Distal Intergenic
chr10	77045053	77045291	Distal Intergenic
chr10	77046265	77046522	Distal Intergenic
chr10	77085707	77085995	Intron
chr10	77100311	77100632	Intron
chr10	77100835	77101426	Intron
chr10	77171652	77172546	Distal Intergenic
chr10	77436818	77437857	Distal Intergenic
chr10	77438605	77439186	Distal Intergenic
chr10	77439358	77439729	Distal Intergenic
chr10	77471719	77472122	Distal Intergenic
chr10	77472158	77472483	Distal Intergenic
chr10	77624998	77626152	Intron
chr10	77633787	77634144	Intron
chr10	77646004	77646238	Intron
chr10	77646824	77647023	Intron
chr10	77647274	77647772	Intron
chr10	77680728	77681200	Intron
chr10	77681364	77681640	Intron
chr10	77883509	77884591	Intron
chr10	77888486	77889218	Intron
chr10	77891072	77891483	Intron
chr10	77891557	77892239	Intron
chr10	78027950	78028197	Intron
chr10	78037455	78037684	Intron
chr10	78037745	78038416	Intron
chr10	79145328	79145731	Intron
chr10	79145771	79146427	Intron
chr10	79146589	79146856	Intron
chr10	79147051	79147250	Intron
chr10	79167238	79167824	Intron
chr10	79169576	79169974	Intron
chr10	79170281	79171077	Intron
chr10	79315634	79317286	Intron
chr10	79317375	79317584	Exon
chr10	79318375	79318574	Exon
chr10	79319403	79319845	Exon
chr10	79320914	79321201	Intron
chr10	79371443	79371709	Intron
chr10	79447704	79448050	Distal Intergenic
chr10	79448304	79448714	Distal Intergenic

chr10	79487092	79487315	Distal Intergenic
chr10	79516690	79516925	Distal Intergenic
chr10	79533997	79534261	Distal Intergenic
chr10	79535420	79536049	Distal Intergenic
chr10	79538166	79538640	Distal Intergenic
chr10	79563631	79563842	Intron
chr10	79564100	79564608	Intron
chr10	79565222	79568141	Exon
chr10	79610564	79611017	Intron
chr10	79611684	79612077	Intron
chr10	79612457	79612667	Promoter
chr10	79613209	79614407	Promoter
chr10	79614800	79615384	Intron
chr10	79620958	79622543	Intron
chr10	79622816	79623542	Intron
chr10	79623587	79624763	Intron
chr10	79624862	79625122	Intron
chr10	79625919	79627150	5' UTR
chr10	79627285	79628153	3' UTR
chr10	79628329	79628528	3' UTR
chr10	79635629	79635896	Intron
chr10	79712030	79712444	Distal Intergenic
chr10	79713972	79715433	Distal Intergenic
chr10	79723982	79724625	Distal Intergenic
chr10	79799296	79799496	Intron
chr10	79807163	79808548	Intron
chr10	79848625	79849335	Distal Intergenic
chr10	79898272	79898665	Distal Intergenic
chr10	80024055	80024527	Intron
chr10	80065991	80066221	Intron
chr10	80086161	80086523	Intron
chr10	80126215	80126562	Intron
chr10	80126604	80127772	Exon
chr10	80138052	80138293	Intron
chr10	80139455	80141444	Exon
chr10	80563795	80564482	Distal Intergenic
chr10	80568023	80568596	Distal Intergenic
chr10	80599435	80599678	Distal Intergenic
chr10	80600631	80601515	Distal Intergenic
chr10	80604153	80605167	Distal Intergenic
chr10	80605224	80606147	Distal Intergenic
chr10	80608127	80610261	Distal Intergenic
chr10	80698041	80699047	Distal Intergenic
chr10	80783664	80783910	Intron

chr10	80847169	80847409	Intron
chr10	80985792	80988012	Intron
chr10	80988130	80988615	Intron
chr10	81089898	81090756	Distal Intergenic
chr10	81640861	81641086	Distal Intergenic
chr10	81816956	81817449	Intron
chr10	81967640	81968668	Promoter
chr10	82092208	82092448	Distal Intergenic
chr10	82092961	82093468	Downstream
chr10	82225712	82225936	Intron
chr10	82258097	82258432	Intron
chr10	82261002	82262091	Intron
chr10	82262298	82263237	Intron
chr10	82306785	82307228	Intron
chr10	82307306	82307606	Intron
chr10	82321057	82324247	Intron
chr10	82438770	82438992	Distal Intergenic
chr10	82451934	82454176	Distal Intergenic
chr10	82455383	82455610	Distal Intergenic
chr10	82455744	82456080	Distal Intergenic
chr10	82516906	82517537	Distal Intergenic
chr10	82518666	82519750	Distal Intergenic
chr10	82614923	82615860	Distal Intergenic
chr10	82640868	82641157	Distal Intergenic
chr10	83358351	83359033	Distal Intergenic
chr10	83360181	83360726	Distal Intergenic
chr10	83361006	83361350	Distal Intergenic
chr10	83371294	83371779	Distal Intergenic
chr10	83371894	83372206	Distal Intergenic
chr10	83431319	83432140	Distal Intergenic
chr10	83432183	83434317	Distal Intergenic
chr10	83447532	83447911	Distal Intergenic
chr10	83448117	83448474	Distal Intergenic
chr10	83466113	83466583	Distal Intergenic
chr10	83486134	83487445	Distal Intergenic
chr10	83519230	83519439	Distal Intergenic
chr10	83528846	83529780	Distal Intergenic
chr10	83542338	83544234	Distal Intergenic
chr10	83563288	83564804	Distal Intergenic
chr10	83577609	83578226	Distal Intergenic
chr10	83584466	83585251	Distal Intergenic
chr10	83656664	83657703	Intron
chr10	83697255	83697661	Intron
chr10	83697733	83697932	Intron

chr10	83701512	83701899	Intron
chr10	83855495	83855912	Intron
chr10	83903751	83904418	Intron
chr10	83994856	83996297	Intron
chr10	84010448	84010686	Intron
chr10	84010931	84011404	Intron
chr10	84039379	84039591	Intron
chr10	84084658	84085532	Intron
chr10	84152442	84152783	Intron
chr10	84817696	84818421	Distal Intergenic
chr10	84829211	84830379	Distal Intergenic
chr10	84834269	84835703	Distal Intergenic
chr10	84851876	84852618	Distal Intergenic
chr10	84852718	84853765	Distal Intergenic
chr10	84883647	84883930	Distal Intergenic
chr10	84914160	84914905	Distal Intergenic
chr10	84931961	84932164	Distal Intergenic
chr10	84944660	84945369	Distal Intergenic
chr10	84948020	84949834	Distal Intergenic
chr10	84957378	84958411	Distal Intergenic
chr10	84978238	84978964	Distal Intergenic
chr10	84985101	84985462	Distal Intergenic
chr10	84989352	84989631	Distal Intergenic
chr10	84990395	84990654	Distal Intergenic
chr10	85040001	85041681	Distal Intergenic
chr10	85188122	85188781	Distal Intergenic
chr10	85435655	85435987	Distal Intergenic
chr10	85451594	85452855	Distal Intergenic
chr10	85473289	85475048	Distal Intergenic
chr10	85485010	85485473	Distal Intergenic
chr10	85558485	85559369	Distal Intergenic
chr10	85559445	85559644	Distal Intergenic
chr10	85563246	85563481	Distal Intergenic
chr10	85633378	85634400	Distal Intergenic
chr10	85635841	85636899	Distal Intergenic
chr10	85660972	85661896	Distal Intergenic
chr10	85748796	85749559	Distal Intergenic
chr10	85753191	85753416	Distal Intergenic
chr10	85801361	85804132	Distal Intergenic
chr10	85913664	85914564	Distal Intergenic
chr10	86074814	86076091	Distal Intergenic
chr10	86167931	86168130	Intron
chr10	86168575	86169585	Intron
chr10	87268466	87268747	Distal Intergenic

chr10	87268930	87269168	Distal Intergenic
chr10	87343011	87343428	Intron
chr10	87609696	87610090	Intron
chr10	87612818	87613550	Intron
chr10	87663951	87664268	Intron
chr10	87808797	87809728	Intron
chr10	87868386	87868611	Intron
chr10	87868722	87868930	Intron
chr10	87977065	87977404	Intron
chr10	88190796	88191032	Distal Intergenic
chr10	88288745	88289204	Distal Intergenic
chr10	88289327	88289526	Distal Intergenic
chr10	88433861	88434300	Intron
chr10	88905397	88905838	Intron
chr10	88906166	88906389	Intron
chr10	88989577	88990204	Intron
chr10	89013736	89015365	Intron
chr10	89062044	89062634	Intron
chr10	89067065	89068056	Promoter
chr10	89208291	89208582	Distal Intergenic
chr10	89208655	89209251	Distal Intergenic
chr10	89531618	89531842	Intron
chr10	89655878	89656741	Intron
chr10	89843054	89843566	Distal Intergenic
chr10	90555145	90555691	Distal Intergenic
chr10	90585348	90586192	Exon
chr10	90586454	90586822	Intron
chr10	90586899	90587718	Intron
chr10	90619263	90619764	Distal Intergenic
chr10	90619806	90620017	Distal Intergenic
chr10	90620616	90622263	Distal Intergenic
chr10	90629760	90630715	Distal Intergenic
chr10	90630872	90631489	Distal Intergenic
chr10	90631572	90631809	Distal Intergenic
chr10	90632014	90632601	Distal Intergenic
chr10	90633021	90633271	Distal Intergenic
chr10	90634763	90635051	Distal Intergenic
chr10	90643201	90644503	Promoter
chr10	90644962	90646731	Promoter
chr10	90696623	90698550	Exon
chr10	90703727	90704195	Intron
chr10	90706660	90707165	5' UTR
chr10	92394875	92395784	Distal Intergenic
chr10	92405580	92406012	Distal Intergenic

chr10	92532057	92532404	Intron
chr10	92557708	92558324	Intron
chr10	92570525	92570748	Intron
chr10	92600951	92601644	Intron
chr10	92623634	92624598	Distal Intergenic
chr10	92624635	92625403	Distal Intergenic
chr10	92648723	92649445	Intron
chr10	92650265	92652255	Intron
chr10	92723895	92725174	Distal Intergenic
chr10	92725229	92725581	Distal Intergenic
chr10	92730673	92732291	Distal Intergenic
chr10	92733342	92736659	Distal Intergenic
chr10	92750310	92751482	Distal Intergenic
chr10	92768126	92772075	Distal Intergenic
chr10	92772128	92773388	Distal Intergenic
chr10	92779430	92780021	Distal Intergenic
chr10	92805291	92807249	Promoter
chr10	92819503	92821054	Intron
chr10	92884618	92886716	Distal Intergenic
chr10	92887999	92888594	Distal Intergenic
chr10	92971452	92973663	Intron
chr10	93104321	93106247	Intron
chr10	93110544	93111819	Exon
chr10	93123526	93124255	Intron
chr10	93124295	93124766	Intron
chr10	93139578	93139812	Intron
chr10	93139929	93140128	Intron
chr10	93245998	93246229	Intron
chr10	93351627	93352801	Intron
chr10	93395985	93396872	Distal Intergenic
chr10	93510710	93511334	Distal Intergenic
chr10	93521990	93522847	Distal Intergenic
chr10	93544576	93545313	Distal Intergenic
chr10	93545357	93545640	Distal Intergenic
chr10	93579324	93581610	Exon
chr10	93611049	93611410	Exon
chr10	93637199	93638233	Distal Intergenic
chr10	93661305	93661727	Distal Intergenic
chr10	93843331	93844183	Intron
chr10	93979650	93980115	Intron
chr10	93987400	93988302	Intron
chr10	94007573	94009478	Intron
chr10	94013473	94014454	Intron
chr10	94014571	94014845	Intron

chr10	94015180	94015408	Intron
chr10	94016579	94019455	Intron
chr10	94020848	94021133	Intron
chr10	94023620	94024580	Intron
chr10	94032485	94032885	Intron
chr10	94033044	94033317	Intron
chr10	94033546	94033839	Intron
chr10	94151925	94152203	Distal Intergenic
chr10	94675948	94676469	Intron
chr10	94744629	94744925	Intron
chr10	94902663	94902901	Distal Intergenic
chr10	94920239	94921517	Distal Intergenic
chr10	94921714	94921938	Distal Intergenic
chr10	94970533	94972332	Distal Intergenic
chr10	94977699	94979089	Distal Intergenic
chr10	94986116	94986338	Distal Intergenic
chr10	94986681	94988674	Distal Intergenic
chr10	94988799	94989047	Distal Intergenic
chr10	94989172	94990247	Distal Intergenic
chr10	95093627	95093866	Exon
chr10	95094471	95094787	Intron
chr10	95324724	95326112	Promoter
chr10	95326674	95327462	Promoter
chr10	95346345	95347212	Exon
chr10	95583559	95585311	Distal Intergenic
chr10	95693989	95694290	Intron
chr10	95878183	95879681	Intron
chr10	96079692	96079957	Intron
chr10	96080216	96080792	Intron
chr10	96080828	96081058	Intron
chr10	96090667	96091774	Downstream
chr10	96095351	96095888	Intron
chr10	96160308	96160517	Distal Intergenic
chr10	96160556	96160902	Distal Intergenic
chr10	96184710	96184909	Intron
chr10	96184966	96185191	Intron
chr10	96776013	96776256	Distal Intergenic
chr10	97795048	97795404	Intron
chr10	97795547	97795784	Intron
chr10	97971428	97971953	Intron
chr10	98406688	98406969	Intron
chr10	98413905	98414359	Intron
chr10	98416918	98417295	Intron
chr10	98417342	98417628	Intron

chr10	98436459	98437606	Intron
chr10	98525163	98526063	Distal Intergenic
chr10	98544277	98544788	Distal Intergenic
chr10	98635967	98636166	Intron
chr10	98699722	98700473	Intron
chr10	98704101	98704866	Intron
chr10	98704914	98705398	Intron
chr10	98705577	98705808	Intron
chr10	98705964	98706563	Intron
chr10	98724177	98725641	3' UTR
chr10	98725889	98726207	Intron
chr10	98771426	98771762	Intron
chr10	98772013	98772449	Intron
chr10	98853720	98853953	Intron
chr10	98854048	98854921	Intron
chr10	98855444	98855653	Intron
chr10	98858528	98860150	Intron
chr10	99372616	99372815	Intron
chr10	99372861	99373257	Intron
chr10	99495138	99495570	Distal Intergenic
chr10	99923311	99924264	Intron
chr10	99946760	99949957	Intron
chr10	100029971	100030363	Distal Intergenic
chr10	102122103	102122341	3' UTR
chr10	102122470	102122969	3' UTR
chr10	104960448	104961066	Intron
chr10	106212125	106212399	Intron
chr10	106212567	106213523	Intron
chr10	107095182	107095537	Distal Intergenic
chr10	107095610	107096631	Distal Intergenic
chr10	107266108	107266369	Distal Intergenic
chr10	107429495	107429801	Distal Intergenic
chr10	107599404	107599645	Distal Intergenic
chr10	107607569	107607998	Distal Intergenic
chr10	107609698	107610623	Distal Intergenic
chr10	107621692	107623892	Distal Intergenic
chr10	107638419	107638778	Distal Intergenic
chr10	107730761	107731824	Distal Intergenic
chr10	107734518	107734979	Distal Intergenic
chr10	107735244	107735902	Distal Intergenic
chr10	107744761	107745852	Distal Intergenic
chr10	107766998	107768931	Distal Intergenic
chr10	107769443	107769670	Distal Intergenic
chr10	107774899	107775263	Distal Intergenic

chr10	107812782	107813202	Distal Intergenic
chr10	107813518	107813717	Distal Intergenic
chr10	107813912	107814111	Distal Intergenic
chr10	107814155	107816250	Distal Intergenic
chr10	109255019	109255524	Distal Intergenic
chr10	109340463	109341420	Distal Intergenic
chr10	109760607	109761333	Distal Intergenic
chr10	109761548	109761851	Distal Intergenic
chr10	110671038	110671468	Distal Intergenic
chr10	110943706	110944289	Distal Intergenic
chr10	110944761	110945244	Distal Intergenic
chr10	110945423	110945900	Distal Intergenic
chr10	110946146	110946780	Distal Intergenic
chr10	110952255	110954513	Distal Intergenic
chr10	110976780	110977034	Distal Intergenic
chr10	110980385	110981258	Distal Intergenic
chr10	110981346	110981547	Distal Intergenic
chr10	110981656	110981869	Distal Intergenic
chr10	111006311	111006703	Distal Intergenic
chr10	111007225	111008599	Distal Intergenic
chr10	111012678	111014239	Distal Intergenic
chr10	111019976	111020261	Distal Intergenic
chr10	111020371	111020748	Distal Intergenic
chr10	111020876	111021759	Distal Intergenic
chr10	111021802	111022140	Distal Intergenic
chr10	111055076	111057663	Distal Intergenic
chr10	111057949	111061967	Distal Intergenic
chr10	111072608	111074996	Distal Intergenic
chr10	111090982	111094193	Distal Intergenic
chr10	111109432	111110476	Distal Intergenic
chr10	111121819	111122603	Distal Intergenic
chr10	111128622	111129146	Distal Intergenic
chr10	111150534	111150767	Distal Intergenic
chr10	111151682	111152705	Distal Intergenic
chr10	111279809	111280033	Distal Intergenic
chr10	111280113	111280724	Distal Intergenic
chr10	111432699	111433898	Distal Intergenic
chr10	111918771	111919140	Distal Intergenic
chr10	112365513	112366015	Distal Intergenic
chr10	114752891	114753144	Intron
chr10	114768672	114770076	Intron
chr10	114785973	114787013	Intron
chr10	114791603	114792058	Intron
chr10	114799604	114800167	Promoter

chr10	114821370	114821609	Intron
chr10	114821990	114822238	Intron
chr10	114822448	114822726	Intron
chr10	114822826	114823263	Intron
chr10	115675790	115676217	Exon
chr10	115703355	115704920	Distal Intergenic
chr10	115704974	115705600	Distal Intergenic
chr10	115705726	115705955	Distal Intergenic
chr10	115965606	115966748	Exon
chr10	115973372	115974683	Exon
chr10	116017041	116018087	Intron
chr10	116018457	116018829	Intron
chr10	116130455	116131535	Intron
chr10	116143503	116144545	Intron
chr10	116144587	116144821	Intron
chr10	116162384	116162622	Intron
chr10	116170090	116171598	Distal Intergenic
chr10	116185572	116187508	Distal Intergenic
chr10	116203800	116204100	Exon
chr10	116205787	116206041	Intron
chr10	116206165	116206959	Intron
chr10	116208010	116209383	Intron
chr10	116433052	116433270	Intron
chr10	116471804	116472841	Intron
chr10	116500381	116500847	Intron
chr10	116501029	116501239	Intron
chr10	116812751	116812991	Distal Intergenic
chr10	117146014	117147386	Intron
chr10	117147543	117147743	Intron
chr10	117147971	117148190	Intron
chr10	117148255	117149103	Intron
chr10	117154569	117155229	Intron
chr10	117158445	117158697	Intron
chr10	117158733	117159258	Intron
chr10	117281724	117282192	Intron
chr10	118246546	118247022	Distal Intergenic
chr10	118425001	118425764	3' UTR
chr10	118425802	118426050	Intron
chr10	118428650	118428926	Promoter
chr10	118437985	118438946	Intron
chr10	118441128	118441344	Exon
chr10	118676933	118677139	Intron
chr10	118796173	118796780	Intron
chr10	118805169	118806409	Intron

chr10	118881648	118882444	Intron
chr10	118883676	118884437	Intron
chr10	118891309	118891521	3' UTR
chr10	119247296	119248684	Exon
chr10	119313957	119314384	Distal Intergenic
chr10	119641676	119642201	Distal Intergenic
chr10	119646377	119646636	Distal Intergenic
chr10	119663952	119664262	Distal Intergenic
chr10	119664619	119664997	Distal Intergenic
chr10	119745746	119746072	Distal Intergenic
chr10	119746340	119746777	Distal Intergenic
chr10	119746829	119747143	Distal Intergenic
chr10	119911720	119912018	Intron
chr10	120153968	120154663	Distal Intergenic
chr10	121024136	121024353	Intron
chr10	121062798	121062997	Intron
chr10	121063156	121063355	Intron
chr10	121340825	121341446	5' UTR
chr10	122244388	122244638	Intron
chr10	122247841	122248739	Intron
chr10	122299062	122299438	Intron
chr10	122299493	122299805	Intron
chr10	122306858	122307236	Intron
chr10	122307287	122308157	Intron
chr10	122308205	122308873	Intron
chr10	122328665	122328955	Intron
chr10	122330024	122330480	Intron
chr10	122330633	122331260	Intron
chr10	122331537	122331832	Intron
chr10	122421256	122421522	Distal Intergenic
chr10	122421646	122422494	Distal Intergenic
chr10	122423422	122424017	Distal Intergenic
chr10	122431814	122432159	Distal Intergenic
chr10	122442035	122442425	Distal Intergenic
chr10	126540176	126540665	Distal Intergenic
chr10	126563633	126563884	Distal Intergenic
chr10	126581178	126581965	Distal Intergenic
chr10	126726470	126727273	Intron
chr10	126867880	126868250	Distal Intergenic
chr10	127000106	127000557	Distal Intergenic
chr10	127027659	127028934	Distal Intergenic
chr10	127029426	127029947	Distal Intergenic
chr10	127030064	127030347	Distal Intergenic
chr10	127033693	127034133	Distal Intergenic

chr10	127034423	127034707	Distal Intergenic
chr10	127058379	127058735	Distal Intergenic
chr10	127072493	127073661	Distal Intergenic
chr10	127079224	127081279	Distal Intergenic
chr10	127104011	127104254	Distal Intergenic
chr10	127104435	127105113	Distal Intergenic
chr10	127105311	127105918	Distal Intergenic
chr10	127341518	127342349	Intron
chr10	127359150	127360968	Intron
chr10	127361086	127363081	Intron
chr10	127364672	127364977	Intron
chr10	127472420	127472821	Distal Intergenic
chr10	127473123	127473372	Distal Intergenic
chr10	127473488	127474341	Downstream
chr10	127496199	127497091	Promoter
chr10	127520331	127520565	Intron
chr10	127527821	127529807	Exon
chr10	127529870	127530387	3' UTR
chr10	127531298	127531725	3' UTR
chr10	127541185	127542012	5' UTR
chr10	127563348	127563598	Intron
chr10	127613067	127613515	Intron
chr10	127613596	127613852	Intron
chr10	127622538	127622825	Intron
chr10	127734933	127735423	Intron
chr10	127740425	127740645	Intron
chr10	127759513	127759885	Intron
chr10	127775359	127775852	Intron
chr10	127781727	127782658	Exon
chr10	127847614	127847850	Intron
chr10	127983617	127984384	Intron
chr10	127985789	127986192	Intron
chr10	128063356	128063825	Intron
chr10	128178951	128180460	Intron
chr10	128184594	128184794	Intron
chr10	128184868	128185235	Intron
chr10	128259064	128259268	Intron
chr10	128358608	128359490	Promoter
chr10	128359642	128360080	Promoter
chr11	3023678	3024358	Exon
chr11	3051313	3051530	Exon
chr11	3052313	3053140	Exon
chr11	3053859	3054274	Intron
chr11	3056938	3058767	Intron

chr11	3166276	3166621	Intron
chr11	3232608	3232807	Distal Intergenic
chr11	3234662	3235849	Distal Intergenic
chr11	3236827	3237026	Downstream
chr11	3237312	3238077	Downstream
chr11	3238165	3238607	Promoter
chr11	3238682	3239384	Promoter
chr11	3248708	3249463	3' UTR
chr11	3249510	3249714	Exon
chr11	3265487	3265820	Distal Intergenic
chr11	3265864	3268237	Distal Intergenic
chr11	3268687	3272279	Distal Intergenic
chr11	3272365	3272666	Distal Intergenic
chr11	3273648	3275451	Distal Intergenic
chr11	3308388	3309022	Distal Intergenic
chr11	3337294	3338647	Distal Intergenic
chr11	3342959	3343644	Distal Intergenic
chr11	3351244	3351483	Distal Intergenic
chr11	3351604	3352975	Distal Intergenic
chr11	3438088	3439398	Distal Intergenic
chr11	3459594	3459987	Distal Intergenic
chr11	3460145	3460582	Distal Intergenic
chr11	3467286	3467643	Distal Intergenic
chr11	3467898	3468473	Distal Intergenic
chr11	3515523	3516060	Distal Intergenic
chr11	3516172	3516457	Distal Intergenic
chr11	3547887	3548086	Distal Intergenic
chr11	3548435	3549156	Distal Intergenic
chr11	3568298	3569815	Distal Intergenic
chr11	3571037	3571713	Distal Intergenic
chr11	3574629	3575059	Distal Intergenic
chr11	3732311	3732773	Intron
chr11	3793793	3795662	Exon
chr11	3804381	3806403	Intron
chr11	3808843	3809937	Intron
chr11	3811132	3811603	Intron
chr11	3811978	3812634	Intron
chr11	3813021	3814856	Intron
chr11	3863542	3864744	Distal Intergenic
chr11	3907581	3907982	Intron
chr11	4002119	4002468	Intron
chr11	4060115	4061096	Intron
chr11	4087035	4087730	Intron
chr11	4087938	4089895	Intron

chr11	4089941	4091992	Exon
chr11	4094852	4096530	Exon
chr11	4097554	4097897	Intron
chr11	4097941	4100163	Intron
chr11	4115779	4118389	Promoter
chr11	4118552	4118833	Intron
chr11	4160682	4160972	Intron
chr11	4161222	4163256	Intron
chr11	4163484	4167652	Intron
chr11	4167711	4168000	Intron
chr11	4198837	4199709	Intron
chr11	4199748	4199997	Intron
chr11	4202109	4204135	Intron
chr11	4207326	4209991	Promoter
chr11	4244909	4245127	Distal Intergenic
chr11	4301235	4302069	Distal Intergenic
chr11	4389204	4390899	Promoter
chr11	4478128	4479397	Distal Intergenic
chr11	4493852	4494478	Distal Intergenic
chr11	4494565	4494806	Distal Intergenic
chr11	4505622	4507797	Distal Intergenic
chr11	4537069	4537550	Distal Intergenic
chr11	4537699	4538724	Distal Intergenic
chr11	4561201	4561447	Distal Intergenic
chr11	4561570	4561788	Distal Intergenic
chr11	4603988	4604618	Distal Intergenic
chr11	4629433	4630082	Promoter
chr11	4738288	4738487	Distal Intergenic
chr11	4743659	4743980	Distal Intergenic
chr11	4748844	4749567	Distal Intergenic
chr11	4749615	4750234	Distal Intergenic
chr11	4760009	4762134	Distal Intergenic
chr11	4762354	4762653	Distal Intergenic
chr11	4795070	4795796	Distal Intergenic
chr11	4799344	4800471	Distal Intergenic
chr11	4909548	4910377	Distal Intergenic
chr11	4922611	4922923	Distal Intergenic
chr11	4937526	4938373	Promoter
chr11	4942849	4943513	Downstream
chr11	4964420	4964817	Downstream
chr11	4965049	4965745	Downstream
chr11	4997065	4997338	Distal Intergenic
chr11	4997617	5001987	Distal Intergenic
chr11	5140212	5140411	Distal Intergenic

chr11	5144460	5145353	Exon
chr11	5145400	5145602	Intron
chr11	5161794	5163739	Distal Intergenic
chr11	5171305	5172503	Downstream
chr11	5195142	5195564	Distal Intergenic
chr11	5255080	5255884	Promoter
chr11	5298525	5298788	Intron
chr11	5327281	5327499	Intron
chr11	5332315	5332735	Intron
chr11	5332839	5333360	Intron
chr11	5334078	5334477	Intron
chr11	5334538	5334745	Intron
chr11	5420936	5422203	Intron
chr11	5441431	5441685	Intron
chr11	5441947	5442216	Intron
chr11	5637144	5637767	Intron
chr11	5641352	5641594	Promoter
chr11	5661937	5662136	Intron
chr11	5662180	5662379	Intron
chr11	5687978	5688485	Intron
chr11	5688530	5689141	Exon
chr11	5697896	5698348	Intron
chr11	5734946	5735781	Intron
chr11	5736549	5738017	Intron
chr11	5796110	5796395	Intron
chr11	5855783	5856701	Intron
chr11	6068591	6070300	Distal Intergenic
chr11	6099982	6100662	Distal Intergenic
chr11	6167510	6168236	Distal Intergenic
chr11	6191919	6192291	Promoter
chr11	6192493	6192768	Promoter
chr11	6246655	6247412	Intron
chr11	6277304	6277593	Distal Intergenic
chr11	6314289	6316049	Distal Intergenic
chr11	6316452	6316859	Distal Intergenic
chr11	6316925	6317648	Distal Intergenic
chr11	6374257	6375693	Distal Intergenic
chr11	6376148	6376587	Distal Intergenic
chr11	6426049	6428756	Promoter
chr11	6446054	6446724	Distal Intergenic
chr11	6446929	6447145	Distal Intergenic
chr11	6447274	6447658	Distal Intergenic
chr11	6486788	6487131	5' UTR
chr11	6487340	6487776	Intron

chr11	6525075	6525373	Intron
chr11	6525856	6526263	Intron
chr11	6594865	6598472	Distal Intergenic
chr11	7111087	7111381	Promoter
chr11	7111675	7112519	3' UTR
chr11	7138030	7138601	Distal Intergenic
chr11	7304484	7305523	Intron
chr11	7322780	7325423	Exon
chr11	7351979	7352380	Intron
chr11	7353937	7354156	Intron
chr11	7356414	7356764	Intron
chr11	7357503	7357708	Intron
chr11	7426376	7426768	Intron
chr11	7461458	7462771	Exon
chr11	7502191	7504676	Intron
chr11	7504716	7505210	Intron
chr11	7515349	7515840	Intron
chr11	7583301	7583571	Intron
chr11	7583736	7583994	Intron
chr11	7605181	7606102	Intron
chr11	7679787	7681928	Distal Intergenic
chr11	7693348	7696239	Promoter
chr11	7797570	7798459	Distal Intergenic
chr11	7798728	7799273	Distal Intergenic
chr11	7799784	7801270	Distal Intergenic
chr11	7895494	7896320	Intron
chr11	8343967	8344754	Distal Intergenic
chr11	8344985	8345200	Distal Intergenic
chr11	8351886	8352489	Distal Intergenic
chr11	8363478	8363823	Distal Intergenic
chr11	8365777	8365982	Distal Intergenic
chr11	8366116	8369212	Distal Intergenic
chr11	8423395	8423651	Intron
chr11	8423852	8424193	Intron
chr11	8443940	8445452	Intron
chr11	8469414	8469948	Intron
chr11	8470015	8470334	Intron
chr11	8470424	8470927	Intron
chr11	8479629	8480058	Intron
chr11	8480126	8480531	Intron
chr11	8482750	8485173	5' UTR
chr11	8489065	8490338	Intron
chr11	8542128	8542374	Intron
chr11	8563958	8564638	Intron

chr11	8567969	8568595	Intron
chr11	8574359	8576112	Intron
chr11	8578051	8578291	Intron
chr11	8578348	8579073	Intron
chr11	8584856	8586636	Intron
chr11	8720852	8722254	Exon
chr11	8730443	8730741	Intron
chr11	8910921	8911407	Intron
chr11	8939321	8939592	3' UTR
chr11	9456389	9457824	Exon
chr11	9458013	9458235	Intron
chr11	12270922	12271140	Intron
chr11	12284693	12285074	3' UTR
chr11	12310842	12311324	Intron
chr11	12311391	12311683	Intron
chr11	12320633	12322536	Intron
chr11	12335006	12335429	Intron
chr11	12341891	12343246	Intron
chr11	12370229	12371416	Exon
chr11	12768116	12768862	Intron
chr11	12770844	12771080	Intron
chr11	12771884	12772375	Intron
chr11	12772535	12773167	Intron
chr11	16407319	16408104	Intron
chr11	16491090	16492352	Intron
chr11	16492389	16492625	Intron
chr11	16583244	16584190	Intron
chr11	16635004	16635696	Intron
chr11	16635796	16636184	Intron
chr11	16637319	16637546	Intron
chr11	16652242	16653798	Intron
chr11	16689704	16691752	Intron
chr11	16695731	16695950	Intron
chr11	16700497	16701210	Intron
chr11	16704088	16704679	Intron
chr11	16709670	16710372	Intron
chr11	16710491	16710868	Intron
chr11	16729059	16729258	Intron
chr11	16729301	16731380	Intron
chr11	16731594	16731797	Intron
chr11	16732599	16733453	Intron
chr11	16744027	16744960	Intron
chr11	16749138	16749877	Intron
chr11	16750605	16751893	Intron

chr11	16757185	16757824	Intron
chr11	16850768	16850980	Intron
chr11	17059503	17059791	Distal Intergenic
chr11	17059837	17060685	Distal Intergenic
chr11	17072021	17073116	Promoter
chr11	17076520	17077138	Distal Intergenic
chr11	17111613	17111891	Intron
chr11	17149394	17149958	Intron
chr11	17157610	17158141	Exon
chr11	17229620	17229876	Promoter
chr11	17286510	17287392	Intron
chr11	18798099	18798863	Intron
chr11	18799013	18799437	Intron
chr11	18804791	18806050	Intron
chr11	18818752	18821150	Distal Intergenic
chr11	18853148	18854392	Distal Intergenic
chr11	18854600	18854846	Distal Intergenic
chr11	18857024	18857331	Distal Intergenic
chr11	18876124	18876573	Distal Intergenic
chr11	18918549	18918748	Distal Intergenic
chr11	18919064	18919869	Distal Intergenic
chr11	18919908	18920451	Distal Intergenic
chr11	19566483	19567420	Intron
chr11	19637161	19638027	Intron
chr11	19639969	19640327	Intron
chr11	19640368	19641356	Intron
chr11	19729323	19730763	Intron
chr11	19734199	19734934	Promoter
chr11	19735038	19737175	Promoter
chr11	19738781	19739434	Intron
chr11	19742718	19743970	Intron
chr11	19747423	19750262	Intron
chr11	19750383	19751146	Intron
chr11	19761457	19761656	Intron
chr11	19769127	19770935	Intron
chr11	19773393	19774610	Intron
chr11	19776883	19778126	Intron
chr11	19819694	19820722	Intron
chr11	19824310	19825003	Intron
chr11	19825111	19825354	Intron
chr11	19825504	19826013	Intron
chr11	19832563	19833445	Intron
chr11	19854891	19855329	Intron
chr11	19855556	19855901	Intron

chr11	19857993	19861602	Intron
chr11	19869160	19870612	Intron
chr11	19878707	19879942	Intron
chr11	19892315	19892549	Intron
chr11	19892766	19893489	Intron
chr11	20007147	20009168	Intron
chr11	20011747	20012525	Intron
chr11	20012604	20013806	Intron
chr11	20101247	20101548	Intron
chr11	20101587	20102879	Exon
chr11	20136900	20138214	Intron
chr11	20232908	20234383	Distal Intergenic
chr11	20247349	20249007	Distal Intergenic
chr11	20257438	20258370	Distal Intergenic
chr11	20269831	20271613	Distal Intergenic
chr11	20272446	20272734	Distal Intergenic
chr11	20272837	20273226	Distal Intergenic
chr11	20377329	20378136	Distal Intergenic
chr11	20442067	20442436	Intron
chr11	20443260	20443460	Intron
chr11	20443537	20443878	Intron
chr11	20470774	20472582	Intron
chr11	20486359	20486953	Intron
chr11	20494242	20495990	Intron
chr11	20530812	20531463	3' UTR
chr11	20531506	20531805	Distal Intergenic
chr11	20532116	20533398	Distal Intergenic
chr11	20547534	20547938	Distal Intergenic
chr11	20640626	20640925	Intron
chr11	20641415	20641634	Intron
chr11	20641689	20642609	Intron
chr11	20786360	20787236	Intron
chr11	20796396	20796710	Intron
chr11	20982874	20983351	Intron
chr11	20991268	20991563	Intron
chr11	20991805	20992006	Intron
chr11	21138289	21139180	Intron
chr11	21139254	21140526	Intron
chr11	21141361	21141634	Intron
chr11	21270038	21270468	Intron
chr11	21270716	21270973	Intron
chr11	21298262	21300366	Intron
chr11	21481129	21482227	Intron
chr11	21482715	21484439	Intron

chr11	21588238	21589030	Intron
chr11	21851077	21852603	Distal Intergenic
chr11	21901320	21901714	Distal Intergenic
chr11	21901818	21902378	Distal Intergenic
chr11	21902431	21904123	Distal Intergenic
chr11	21904226	21905923	Distal Intergenic
chr11	21908291	21908951	Distal Intergenic
chr11	22147284	22147911	Distal Intergenic
chr11	22360095	22361730	Promoter
chr11	22424393	22425055	Distal Intergenic
chr11	22430270	22430568	Distal Intergenic
chr11	22511673	22511946	Distal Intergenic
chr11	22521706	22523024	Distal Intergenic
chr11	22528657	22530370	Distal Intergenic
chr11	22619701	22619905	Distal Intergenic
chr11	22633048	22633699	Distal Intergenic
chr11	22711562	22711831	Intron
chr11	22743128	22744809	Intron
chr11	22751042	22751320	Intron
chr11	22751362	22752160	Intron
chr11	22754943	22755149	Intron
chr11	22756306	22757256	Intron
chr11	22757311	22758782	Intron
chr11	22759275	22759691	Exon
chr11	22865142	22865851	Downstream
chr11	22882056	22882284	Promoter
chr11	22890280	22890541	Distal Intergenic
chr11	22890728	22891593	Distal Intergenic
chr11	22968574	22968805	Distal Intergenic
chr11	23155031	23155504	Distal Intergenic
chr11	23156161	23156568	Distal Intergenic
chr11	23156746	23157055	Distal Intergenic
chr11	23157119	23157791	Distal Intergenic
chr11	23205500	23205801	Distal Intergenic
chr11	23205902	23206270	Distal Intergenic
chr11	23206830	23207865	Distal Intergenic
chr11	23208046	23208975	Distal Intergenic
chr11	23564623	23564899	Distal Intergenic
chr11	23565036	23567009	Distal Intergenic
chr11	23612270	23613187	Distal Intergenic
chr11	23623287	23623658	Distal Intergenic
chr11	23623708	23627499	Distal Intergenic
chr11	23646223	23647443	Distal Intergenic
chr11	23669174	23670972	Distal Intergenic

chr11	24090050	24090422	Distal Intergenic
chr11	24242998	24243352	Distal Intergenic
chr11	24271997	24272356	Distal Intergenic
chr11	24272402	24272713	Distal Intergenic
chr11	24272988	24273236	Distal Intergenic
chr11	26492813	26494395	Intron
chr11	26499367	26500651	Intron
chr11	27229982	27231631	Distal Intergenic
chr11	27243733	27244658	Distal Intergenic
chr11	27571660	27572269	Intron
chr11	28712067	28712335	Distal Intergenic
chr11	28712395	28713521	Distal Intergenic
chr11	28733158	28734545	Distal Intergenic
chr11	28780911	28782239	Distal Intergenic
chr11	28790224	28790500	Distal Intergenic
chr11	28806066	28806272	Distal Intergenic
chr11	28806312	28806716	Distal Intergenic
chr11	29030759	29030962	Distal Intergenic
chr11	29072538	29073671	Distal Intergenic
chr11	29073734	29073933	Distal Intergenic
chr11	29414650	29414896	Distal Intergenic
chr11	29446441	29447555	Distal Intergenic
chr11	29447619	29448796	Distal Intergenic
chr11	29579578	29579781	Distal Intergenic
chr11	29579869	29580401	Distal Intergenic
chr11	29592292	29592698	Distal Intergenic
chr11	29592955	29593188	Distal Intergenic
chr11	29593582	29593952	Distal Intergenic
chr11	29594032	29594690	Distal Intergenic
chr11	29739955	29740226	Distal Intergenic
chr11	30017931	30019072	Distal Intergenic
chr11	30058211	30059241	Distal Intergenic
chr11	30082880	30083249	Distal Intergenic
chr11	30083317	30083523	Distal Intergenic
chr11	30096026	30097323	Distal Intergenic
chr11	30097554	30099160	Distal Intergenic
chr11	30109492	30109925	Distal Intergenic
chr11	30111221	30111721	Distal Intergenic
chr11	30510591	30512136	Intron
chr11	30672246	30672593	Distal Intergenic
chr11	30673097	30673410	Distal Intergenic
chr11	30853461	30853882	Intron
chr11	30854364	30854582	Intron
chr11	30885890	30886944	3' UTR

chr11	31423952	31424886	Intron
chr11	31570405	31570903	Intron
chr11	31571296	31571517	Intron
chr11	32118485	32119307	Promoter
chr11	32120830	32122228	Exon
chr11	32150903	32151543	Distal Intergenic
chr11	32162891	32163269	Distal Intergenic
chr11	32167097	32167947	Distal Intergenic
chr11	32212483	32215431	Distal Intergenic
chr11	32236358	32236947	Distal Intergenic
chr11	32245446	32247100	Distal Intergenic
chr11	32247409	32247809	Distal Intergenic
chr11	32247912	32250152	Distal Intergenic
chr11	32335371	32336153	Distal Intergenic
chr11	32487153	32487797	Distal Intergenic
chr11	32505105	32505898	Distal Intergenic
chr11	32534957	32535406	Distal Intergenic
chr11	32542512	32543349	Distal Intergenic
chr11	32543390	32544170	Distal Intergenic
chr11	32561625	32562377	Distal Intergenic
chr11	32566834	32568702	Distal Intergenic
chr11	32605010	32605209	Promoter
chr11	32665904	32666412	Intron
chr11	33048296	33048623	Intron
chr11	33061431	33061940	Promoter
chr11	33062070	33062792	Promoter
chr11	34715470	34715680	Distal Intergenic
chr11	34716020	34716258	Distal Intergenic
chr11	34879835	34880144	Distal Intergenic
chr11	34969069	34971207	Exon
chr11	35075467	35075715	Distal Intergenic
chr11	35076060	35076343	Distal Intergenic
chr11	35076490	35076818	Distal Intergenic
chr11	35130481	35132296	Distal Intergenic
chr11	35144409	35145916	Distal Intergenic
chr11	35228856	35230117	5' UTR
chr11	35286845	35288097	3' UTR
chr11	35481486	35481949	Intron
chr11	35520486	35521156	Intron
chr11	35582275	35583213	Distal Intergenic
chr11	35583293	35584355	Distal Intergenic
chr11	35584427	35584698	Distal Intergenic
chr11	35647251	35647750	Distal Intergenic
chr11	35651017	35652094	Distal Intergenic

chr11	35652147	35652996	Distal Intergenic
chr11	35658033	35658250	Distal Intergenic
chr11	35691549	35691947	Intron
chr11	35692164	35692363	Intron
chr11	35739006	35739709	Intron
chr11	35754248	35755201	Intron
chr11	35756377	35756623	Intron
chr11	35764444	35764696	Intron
chr11	35768639	35770094	Intron
chr11	35783962	35785681	Intron
chr11	35792661	35793534	Intron
chr11	35796079	35796682	Intron
chr11	37909481	37909685	Distal Intergenic
chr11	37931447	37931830	Distal Intergenic
chr11	40505617	40507376	Intron
chr11	40507539	40508185	Intron
chr11	40546492	40546914	Intron
chr11	40547453	40548263	Intron
chr11	40554790	40555791	Intron
chr11	40567402	40569569	Intron
chr11	40603331	40603543	Intron
chr11	40603776	40604197	Intron
chr11	42199786	42200797	Distal Intergenic
chr11	42752907	42753192	Distal Intergenic
chr11	42836375	42836980	Distal Intergenic
chr11	42845717	42845916	Distal Intergenic
chr11	43239585	43240479	Distal Intergenic
chr11	43253187	43253449	Distal Intergenic
chr11	43706035	43707205	Intron
chr11	43732922	43733121	Intron
chr11	43733184	43734142	Intron
chr11	43740292	43740753	Intron
chr11	43746887	43747092	Intron
chr11	43952421	43953599	Distal Intergenic
chr11	44136286	44137452	Intron
chr11	44333037	44335166	Distal Intergenic
chr11	44347990	44349396	Distal Intergenic
chr11	44988499	44988714	Distal Intergenic
chr11	45618675	45619340	Distal Intergenic
chr11	45665939	45666449	Downstream
chr11	45689985	45691365	Distal Intergenic
chr11	45973672	45973871	Intron
chr11	45973973	45975092	Exon
chr11	45978222	45978612	Intron

chr11	45979213	45979503	Intron
chr11	45979613	45980090	Intron
chr11	45990375	45991326	Intron
chr11	46038399	46039700	Intron
chr11	46134623	46135161	Intron
chr11	46250499	46250702	Distal Intergenic
chr11	46250893	46251094	Distal Intergenic
chr11	46275322	46275604	Distal Intergenic
chr11	47910553	47912238	Distal Intergenic
chr11	48612944	48615400	Distal Intergenic
chr11	48631172	48631745	Distal Intergenic
chr11	48632823	48634617	Distal Intergenic
chr11	48651344	48653290	Distal Intergenic
chr11	48668670	48668889	Distal Intergenic
chr11	48787596	48788891	Distal Intergenic
chr11	48859596	48860279	Distal Intergenic
chr11	48860334	48860596	Distal Intergenic
chr11	49016237	49018174	Distal Intergenic
chr11	49059590	49062968	Distal Intergenic
chr11	49063092	49064450	Distal Intergenic
chr11	49280772	49281315	Distal Intergenic
chr11	49345179	49345531	Distal Intergenic
chr11	49346243	49346745	Distal Intergenic
chr11	49526082	49526313	Distal Intergenic
chr11	49526491	49526874	Distal Intergenic
chr11	49580153	49580715	Promoter
chr11	50023275	50027395	Distal Intergenic
chr11	50038074	50038772	Distal Intergenic
chr11	50065805	50066227	Distal Intergenic
chr11	50088593	50088798	Distal Intergenic
chr11	50088931	50089661	Distal Intergenic
chr11	50104722	50105418	Distal Intergenic
chr11	50105455	50105808	Distal Intergenic
chr11	50105999	50106820	Distal Intergenic
chr11	50126316	50126634	Distal Intergenic
chr11	50126724	50126968	Distal Intergenic
chr11	50127565	50127868	Distal Intergenic
chr11	50138029	50139221	Distal Intergenic
chr11	50139359	50139806	Distal Intergenic
chr11	50139916	50140169	Distal Intergenic
chr11	50191206	50191883	Distal Intergenic
chr11	50254978	50255735	Intron
chr11	50487595	50488008	Distal Intergenic
chr11	51076656	51076873	Distal Intergenic

chr11	51076966	51077326	Distal Intergenic
chr11	51077521	51078444	Distal Intergenic
chr11	51242973	51243200	Distal Intergenic
chr11	51243241	51243559	Distal Intergenic
chr11	51243616	51244392	Distal Intergenic
chr11	51244432	51245411	Distal Intergenic
chr11	51397288	51398001	Distal Intergenic
chr11	51398224	51398693	Distal Intergenic
chr11	51419307	51419553	Distal Intergenic
chr11	51419808	51420039	Distal Intergenic
chr11	51420440	51420712	Distal Intergenic
chr11	51428665	51429600	Distal Intergenic
chr11	51433436	51433746	Distal Intergenic
chr11	51433808	51434041	Distal Intergenic
chr11	51438491	51438754	Distal Intergenic
chr11	51501770	51502019	Distal Intergenic
chr11	51502378	51502795	Distal Intergenic
chr11	51502857	51503065	Distal Intergenic
chr11	51614286	51614559	Distal Intergenic
chr11	51614821	51615241	Distal Intergenic
chr11	51668650	51668912	Distal Intergenic
chr11	51669318	51669530	Distal Intergenic
chr11	51679458	51680054	Distal Intergenic
chr11	51687556	51688010	Distal Intergenic
chr11	51688046	51688876	Distal Intergenic
chr11	51728084	51728355	Distal Intergenic
chr11	51729158	51730549	Distal Intergenic
chr11	51735733	51736713	Distal Intergenic
chr11	51781610	51781867	Distal Intergenic
chr11	51813446	51813851	Distal Intergenic
chr11	51816912	51817625	Distal Intergenic
chr11	51817778	51818510	Distal Intergenic
chr11	51848436	51849728	Distal Intergenic
chr11	51859922	51860443	Distal Intergenic
chr11	51885054	51885416	Distal Intergenic
chr11	51891172	51892164	Distal Intergenic
chr11	51893175	51894553	Distal Intergenic
chr11	51912402	51913565	Distal Intergenic
chr11	51937860	51939251	Distal Intergenic
chr11	51962125	51962556	Distal Intergenic
chr11	52045592	52045827	Distal Intergenic
chr11	52045899	52046102	Distal Intergenic
chr11	52146459	52147822	Distal Intergenic
chr11	52149368	52149650	Distal Intergenic

chr11	52150904	52151997	Distal Intergenic
chr11	52174396	52175372	Distal Intergenic
chr11	52209815	52210120	Distal Intergenic
chr11	52210283	52210546	Distal Intergenic
chr11	52261701	52263153	Distal Intergenic
chr11	53112622	53113844	Distal Intergenic
chr11	53114022	53114658	Distal Intergenic
chr11	53144354	53145402	Distal Intergenic
chr11	53145578	53145803	Distal Intergenic
chr11	53164525	53165796	Distal Intergenic
chr11	53243811	53244348	Distal Intergenic
chr11	53273054	53274484	Distal Intergenic
chr11	53296832	53297039	Distal Intergenic
chr11	53297136	53297460	Distal Intergenic
chr11	53333646	53334318	Distal Intergenic
chr11	53520227	53520746	Distal Intergenic
chr11	53572244	53573492	Distal Intergenic
chr11	53574930	53575174	Distal Intergenic
chr11	53575210	53576546	Distal Intergenic
chr11	53576584	53576800	Distal Intergenic
chr11	53582157	53583154	Distal Intergenic
chr11	53593147	53593412	Distal Intergenic
chr11	53608366	53609159	Distal Intergenic
chr11	53609326	53610957	Distal Intergenic
chr11	53616244	53618293	Distal Intergenic
chr11	53627077	53627279	Distal Intergenic
chr11	53831465	53831851	Distal Intergenic
chr11	53831964	53833276	Distal Intergenic
chr11	54252248	54252485	Distal Intergenic
chr11	54265977	54266188	Distal Intergenic
chr11	54305353	54306560	Distal Intergenic
chr11	54336400	54336961	Distal Intergenic
chr11	54542686	54543426	Distal Intergenic
chr11	54561995	54562788	Distal Intergenic
chr11	54600179	54600503	Distal Intergenic
chr11	54673182	54673722	Distal Intergenic
chr11	54674126	54674694	Distal Intergenic
chr11	54769192	54769391	Distal Intergenic
chr11	54941650	54942245	Distal Intergenic
chr11	55093530	55094415	Distal Intergenic
chr11	55102977	55104344	Distal Intergenic
chr11	55126877	55127093	Distal Intergenic
chr11	55127250	55128748	Distal Intergenic
chr11	55128965	55130403	Distal Intergenic

chr11	55140339	55142624	Distal Intergenic
chr11	55178543	55178785	Distal Intergenic
chr11	55214566	55214779	Distal Intergenic
chr11	55217978	55220550	Distal Intergenic
chr11	55229476	55229850	Distal Intergenic
chr11	55230270	55230592	Distal Intergenic
chr11	55230757	55233870	Distal Intergenic
chr11	55237501	55237774	Distal Intergenic
chr11	55241309	55242550	Distal Intergenic
chr11	55259910	55260234	Distal Intergenic
chr11	55266625	55267968	Distal Intergenic
chr11	55268763	55270157	Distal Intergenic
chr11	55270213	55271166	Distal Intergenic
chr11	55273949	55274222	Distal Intergenic
chr11	55281845	55282708	Distal Intergenic
chr11	55314438	55314840	Distal Intergenic
chr11	55314930	55315259	Distal Intergenic
chr11	55316034	55318565	Distal Intergenic
chr11	55318622	55319624	Distal Intergenic
chr11	55405637	55405843	Promoter
chr11	55408293	55409712	Distal Intergenic
chr11	55447079	55447823	Distal Intergenic
chr11	55557628	55558711	Distal Intergenic
chr11	55560337	55562042	Promoter
chr11	55780165	55780548	Distal Intergenic
chr11	57331430	57331878	Intron
chr11	57532962	57533280	Intron
chr11	57712790	57713046	Distal Intergenic
chr11	57724954	57725234	Distal Intergenic
chr11	57726752	57728520	Distal Intergenic
chr11	57775673	57776252	Distal Intergenic
chr11	57821852	57822056	Intron
chr11	57822961	57824255	5' UTR
chr11	57828676	57830501	Intron
chr11	57834275	57835571	Intron
chr11	57837342	57838930	Intron
chr11	57854451	57855565	Intron
chr11	57856499	57856754	Intron
chr11	57881501	57882989	Intron
chr11	57917593	57917931	Intron
chr11	58050210	58051374	Distal Intergenic
chr11	58057087	58057915	Distal Intergenic
chr11	58062810	58064251	Distal Intergenic
chr11	58143860	58144347	Distal Intergenic

chr11	58147646	58148206	Distal Intergenic
chr11	58792189	58794543	Intron
chr11	59031689	59033162	Distal Intergenic
chr11	59039371	59041746	Distal Intergenic
chr11	59091903	59092189	Distal Intergenic
chr11	59092274	59092910	Distal Intergenic
chr11	59093076	59093314	Distal Intergenic
chr11	59095258	59097155	Distal Intergenic
chr11	59097199	59098445	Distal Intergenic
chr11	59137149	59138101	Distal Intergenic
chr11	59198316	59198582	Distal Intergenic
chr11	59215795	59217466	Distal Intergenic
chr11	59222877	59223076	Downstream
chr11	59223128	59228853	Promoter
chr11	59233030	59235878	Distal Intergenic
chr11	59245022	59245260	Promoter
chr11	59263399	59264138	Distal Intergenic
chr11	59269989	59270335	Promoter
chr11	59270457	59270664	Promoter
chr11	59271247	59274193	Promoter
chr11	59285205	59285980	Distal Intergenic
chr11	59319519	59319739	Distal Intergenic
chr11	59413826	59415701	Exon
chr11	59447423	59448355	Distal Intergenic
chr11	59448471	59449915	Distal Intergenic
chr11	59450207	59450882	Distal Intergenic
chr11	59468157	59470001	Distal Intergenic
chr11	59476580	59476945	Distal Intergenic
chr11	59476997	59477236	Distal Intergenic
chr11	59477286	59477549	Downstream
chr11	59496034	59496787	Distal Intergenic
chr11	59531200	59531426	Intron
chr11	59544407	59544867	Intron
chr11	59545024	59546696	Intron
chr11	59556566	59557793	Intron
chr11	59623344	59624121	Exon
chr11	59903894	59904254	Distal Intergenic
chr11	59919078	59920468	Distal Intergenic
chr11	59921853	59922655	Distal Intergenic
chr11	59926978	59927368	Distal Intergenic
chr11	59927420	59927704	Distal Intergenic
chr11	59927820	59928021	Distal Intergenic
chr11	59928081	59928823	Distal Intergenic
chr11	59928866	59929287	Distal Intergenic

chr11	60033180	60034082	Distal Intergenic
chr11	60034377	60034602	Distal Intergenic
chr11	60170575	60170794	Intron
chr11	60179941	60180140	Intron
chr11	60350313	60350703	Distal Intergenic
chr11	60350746	60350976	Distal Intergenic
chr11	60351516	60351715	Distal Intergenic
chr11	60521040	60521679	Distal Intergenic
chr11	60540164	60540383	Intron
chr11	60540610	60541332	Exon
chr11	60541460	60541663	Intron
chr11	60589999	60591006	Distal Intergenic
chr11	60591104	60591379	Distal Intergenic
chr11	60644202	60644409	Distal Intergenic
chr11	60677195	60678277	Distal Intergenic
chr11	60691714	60691929	Promoter
chr11	60691986	60692325	Promoter
chr11	60692433	60692801	Promoter
chr11	60744423	60745396	Intron
chr11	60745699	60745968	Intron
chr11	60746018	60746312	Intron
chr11	60747069	60747268	Intron
chr11	60748768	60749035	Intron
chr11	60749073	60749433	Intron
chr11	60754442	60755106	Intron
chr11	61013299	61013787	Exon
chr11	61080750	61081043	Exon
chr11	61087059	61088087	Intron
chr11	61092205	61092598	Intron
chr11	61451030	61452045	Intron
chr11	61455253	61455923	Intron
chr11	61457008	61457287	Intron
chr11	61457572	61458761	Intron
chr11	61460670	61462129	Intron
chr11	61488789	61490737	Exon
chr11	61497646	61497845	Intron
chr11	61497894	61502206	Exon
chr11	61502263	61502860	Exon
chr11	61503009	61503349	Exon
chr11	61504095	61504338	Intron
chr11	61506059	61506819	Intron
chr11	61506876	61508267	Exon
chr11	61510436	61510792	Intron
chr11	61512088	61512502	3' UTR

chr11	61512630	61513632	3' UTR
chr11	61519518	61520246	Promoter
chr11	61521699	61522619	Promoter
chr11	61836175	61836429	Distal Intergenic
chr11	61836465	61837336	Distal Intergenic
chr11	61888583	61889334	Distal Intergenic
chr11	61890501	61890764	Promoter
chr11	61891246	61891834	Promoter
chr11	61903295	61903636	Intron
chr11	61954299	61954820	Distal Intergenic
chr11	61957530	61957945	Promoter
chr11	61958094	61958350	Promoter
chr11	61958718	61959159	Intron
chr11	61959215	61959422	Intron
chr11	61961294	61961553	Distal Intergenic
chr11	61998433	61999138	Distal Intergenic
chr11	62057559	62057802	Distal Intergenic
chr11	62063117	62063483	Downstream
chr11	62063536	62065002	3' UTR
chr11	62081300	62081618	Distal Intergenic
chr11	62081713	62083276	Distal Intergenic
chr11	62122833	62123279	Intron
chr11	62270186	62270419	Intron
chr11	62270580	62270879	Intron
chr11	62270920	62271823	Intron
chr11	62362922	62364565	Exon
chr11	62364727	62365677	5' UTR
chr11	62416308	62418344	Exon
chr11	62418762	62419016	Intron
chr11	62627438	62628340	Intron
chr11	62633431	62635638	Intron
chr11	62638626	62639811	Exon
chr11	62912622	62913502	Promoter
chr11	62928282	62928910	Intron
chr11	62949644	62950237	Intron
chr11	63575864	63576065	Distal Intergenic
chr11	63576335	63577807	Distal Intergenic
chr11	63660533	63660904	Intron
chr11	63671849	63673513	Exon
chr11	63676248	63676647	Exon
chr11	63704985	63705502	Promoter
chr11	63723113	63724318	3' UTR
chr11	63892994	63893443	Intron
chr11	64049793	64050172	Intron

chr11	64195673	64195872	Distal Intergenic
chr11	64196031	64196695	Distal Intergenic
chr11	64282233	64282472	Distal Intergenic
chr11	64282575	64282877	Distal Intergenic
chr11	64285513	64287240	Distal Intergenic
chr11	64314069	64314329	Distal Intergenic
chr11	64315773	64317176	Distal Intergenic
chr11	64457277	64458189	Promoter
chr11	64750380	64751001	Distal Intergenic
chr11	64759956	64760322	Intron
chr11	65601543	65601744	Promoter
chr11	65619952	65620386	Exon
chr11	65631506	65631717	Promoter
chr11	66865692	66866100	Distal Intergenic
chr11	67037802	67038203	Intron
chr11	67376762	67377002	Promoter
chr11	67903591	67904009	Distal Intergenic
chr11	67905638	67906331	Distal Intergenic
chr11	68665741	68666533	Intron
chr11	68666735	68668033	5' UTR
chr11	68673169	68674818	Exon
chr11	68685184	68687181	Exon
chr11	68715097	68715756	Distal Intergenic
chr11	68715988	68716422	Distal Intergenic
chr11	68759821	68760618	Distal Intergenic
chr11	68780587	68780867	Promoter
chr11	68780931	68781418	Promoter
chr11	68828948	68830700	Exon
chr11	68852940	68853743	Exon
chr11	68858739	68859006	Distal Intergenic
chr11	68859134	68859781	Distal Intergenic
chr11	68873245	68873510	Distal Intergenic
chr11	68886689	68887107	Distal Intergenic
chr11	68887143	68887587	Distal Intergenic
chr11	68908918	68909393	Distal Intergenic
chr11	68910234	68910493	Distal Intergenic
chr11	68910596	68910828	Distal Intergenic
chr11	68910882	68911130	Distal Intergenic
chr11	68913118	68913488	Distal Intergenic
chr11	68938824	68939294	Distal Intergenic
chr11	69178990	69181180	Distal Intergenic
chr11	69196143	69196633	Distal Intergenic
chr11	69208731	69209212	Distal Intergenic
chr11	69222136	69224960	Distal Intergenic

chr11	69225014	69225567	Distal Intergenic
chr11	69227200	69228224	Distal Intergenic
chr11	69228389	69229580	Exon
chr11	69229942	69230169	Exon
chr11	69231260	69231638	Exon
chr11	69231738	69232102	Distal Intergenic
chr11	69392459	69392907	Distal Intergenic
chr11	69393029	69394621	Distal Intergenic
chr11	69394753	69395253	Distal Intergenic
chr11	69468143	69468500	3' UTR
chr11	69468574	69469007	3' UTR
chr11	69469105	69470897	3' UTR
chr11	69475899	69476105	Intron
chr11	69479458	69479708	Intron
chr11	69479839	69480042	Intron
chr11	69484956	69485235	Intron
chr11	69485289	69485527	Intron
chr11	69485618	69486494	3' UTR
chr11	69495036	69495957	Distal Intergenic
chr11	69571871	69572249	Distal Intergenic
chr11	69572286	69572529	Distal Intergenic
chr11	69572615	69573095	Distal Intergenic
chr11	69573139	69573421	Distal Intergenic
chr11	69618021	69618397	Distal Intergenic
chr11	69659207	69661018	Distal Intergenic
chr11	69691483	69695694	Distal Intergenic
chr11	69696507	69697925	Distal Intergenic
chr11	69727234	69727471	Distal Intergenic
chr11	69732896	69734461	Distal Intergenic
chr11	69734612	69734830	Distal Intergenic
chr11	69735326	69735921	Distal Intergenic
chr11	69774479	69775751	Distal Intergenic
chr11	69804118	69804486	Distal Intergenic
chr11	69804738	69805711	Distal Intergenic
chr11	69809403	69810251	Distal Intergenic
chr11	69810306	69810680	Distal Intergenic
chr11	69810885	69811114	Distal Intergenic
chr11	70002474	70003801	Exon
chr11	70005662	70006149	Intron
chr11	70006193	70007969	Exon
chr11	70008037	70008322	Intron
chr11	70010947	70012487	Exon
chr11	70023774	70025054	Intron
chr11	70033341	70034023	Exon

chr11	70034092	70034617	3' UTR
chr11	70051714	70052097	Intron
chr11	70052429	70052654	3' UTR
chr11	70072808	70073185	Distal Intergenic
chr11	70101598	70102214	Distal Intergenic
chr11	70104713	70105022	Distal Intergenic
chr11	70106872	70107236	Distal Intergenic
chr11	70265452	70265684	Promoter
chr11	70265740	70266856	Promoter
chr11	70271786	70273870	Intron
chr11	70280875	70281688	3' UTR
chr11	70281957	70282199	3' UTR
chr11	70338844	70339282	Intron
chr11	70375211	70376129	Intron
chr11	70386225	70386607	Intron
chr11	70387073	70387311	Intron
chr11	70387493	70387775	Intron
chr11	70389975	70394200	Intron
chr11	70396564	70398298	Intron
chr11	70398595	70400915	Intron
chr11	70460785	70461232	Intron
chr11	70464814	70465452	Intron
chr11	70465627	70466185	Intron
chr11	70467188	70468150	Intron
chr11	70468776	70469243	Intron
chr11	70481743	70482234	Intron
chr11	70482503	70482790	Intron
chr11	70514342	70514632	Intron
chr11	70515110	70515316	Intron
chr11	70528867	70529148	Intron
chr11	70529290	70529542	Intron
chr11	70648913	70649399	Intron
chr11	70833150	70833572	Intron
chr11	71855459	71855730	Distal Intergenic
chr11	71895727	71897959	Distal Intergenic
chr11	71898088	71898287	Distal Intergenic
chr11	71903789	71904450	Promoter
chr11	71923684	71925057	Distal Intergenic
chr11	71925257	71925625	Distal Intergenic
chr11	71925693	71926732	Distal Intergenic
chr11	71945543	71946523	Exon
chr11	71956481	71958201	Distal Intergenic
chr11	71966646	71967541	Distal Intergenic
chr11	71976735	71977171	Distal Intergenic

chr11	71977458	71977805	Distal Intergenic
chr11	72009730	72011051	Intron
chr11	72020085	72023317	Promoter
chr11	72248754	72249222	Distal Intergenic
chr11	72254624	72255393	Distal Intergenic
chr11	72309642	72309850	Intron
chr11	72314670	72314869	Intron
chr11	72314907	72315121	Intron
chr11	72315241	72315707	Intron
chr11	72420749	72420972	Exon
chr11	72421692	72422166	Exon
chr11	72503495	72504024	Promoter
chr11	72509771	72510857	Distal Intergenic
chr11	72565290	72565633	Intron
chr11	72604991	72607963	Intron
chr11	72608114	72609571	Intron
chr11	72655908	72656469	Intron
chr11	72656630	72656929	Intron
chr11	72657019	72657299	Intron
chr11	72659996	72662384	Intron
chr11	72835232	72835431	Intron
chr11	72861367	72861851	Distal Intergenic
chr11	72989737	72990154	Intron
chr11	72990289	72990543	Intron
chr11	72990713	72992528	Intron
chr11	73079659	73080156	3' UTR
chr11	73080279	73080917	3' UTR
chr11	73139953	73140370	Intron
chr11	74295039	74295340	Distal Intergenic
chr11	74296845	74297513	Distal Intergenic
chr11	74369375	74369775	Distal Intergenic
chr11	74370274	74370478	Distal Intergenic
chr11	74370634	74371531	Distal Intergenic
chr11	74455719	74455970	Distal Intergenic
chr11	74456069	74456455	Distal Intergenic
chr11	74484425	74484827	Intron
chr11	74485012	74485243	Intron
chr11	74486245	74487616	Intron
chr11	74517365	74517965	Intron
chr11	74536730	74537531	Intron
chr11	74537652	74538299	Intron
chr11	74538348	74538569	Intron
chr11	74576764	74577916	Intron
chr11	74643409	74643695	Intron

chr11	74645475	74645695	Intron
chr11	74645971	74646283	Intron
chr11	74684743	74685216	Intron
chr11	74687549	74688325	3' UTR
chr11	74711618	74711974	Intron
chr11	74724291	74724542	Distal Intergenic
chr11	74859134	74859488	Intron
chr11	74866421	74867069	Intron
chr11	74870061	74871022	Promoter
chr11	74873987	74874553	Intron
chr11	75201376	75201849	Promoter
chr11	75202909	75203129	Intron
chr11	75215906	75216105	Intron
chr11	75216226	75217897	Exon
chr11	75273163	75274984	Promoter
chr11	75275123	75275384	Intron
chr11	75275508	75277051	Intron
chr11	75300364	75300891	Intron
chr11	75364885	75365785	Intron
chr11	75377269	75378410	Intron
chr11	75406019	75406311	Distal Intergenic
chr11	75433946	75434266	Intron
chr11	75434619	75435092	Intron
chr11	75444041	75444340	Distal Intergenic
chr11	75465058	75465885	Distal Intergenic
chr11	75466897	75467449	Downstream
chr11	75468561	75469775	Exon
chr11	75491292	75492211	Intron
chr11	75492311	75493465	Intron
chr11	75493503	75493900	Intron
chr11	75546486	75547477	Intron
chr11	75549403	75550754	Promoter
chr11	75673898	75675620	Intron
chr11	75793473	75795053	Intron
chr11	75873327	75874172	Distal Intergenic
chr11	75934223	75934457	Distal Intergenic
chr11	75934560	75934769	Distal Intergenic
chr11	75944052	75944698	Distal Intergenic
chr11	75948707	75949886	Distal Intergenic
chr11	76022101	76023824	Distal Intergenic
chr11	76029962	76031042	Distal Intergenic
chr11	76049198	76050093	Distal Intergenic
chr11	76078702	76079021	Intron
chr11	76079203	76079492	Intron

chr11	76097166	76098282	Intron
chr11	76098388	76100398	Intron
chr11	76100453	76101090	Intron
chr11	76113713	76115320	Exon
chr11	76115594	76116594	Intron
chr11	76125436	76127218	Exon
chr11	76128432	76128689	Distal Intergenic
chr11	76132804	76133417	Distal Intergenic
chr11	76133463	76134041	Distal Intergenic
chr11	76144597	76146945	Distal Intergenic
chr11	76187733	76189922	Intron
chr11	76211060	76211318	Intron
chr11	76211378	76211974	Intron
chr11	76220428	76220869	Intron
chr11	76278579	76278792	Distal Intergenic
chr11	76282872	76284173	Distal Intergenic
chr11	76286005	76286623	Distal Intergenic
chr11	76289777	76291657	Distal Intergenic
chr11	76291871	76294561	Distal Intergenic
chr11	76306871	76307112	Distal Intergenic
chr11	76307237	76307742	Distal Intergenic
chr11	76307789	76308786	Distal Intergenic
chr11	76385212	76385411	Distal Intergenic
chr11	76715027	76716000	Intron
chr11	76716171	76716990	Intron
chr11	76815965	76816362	Intron
chr11	76817979	76819450	Intron
chr11	76891200	76892594	Promoter
chr11	76973785	76974464	Intron
chr11	77041981	77043442	Intron
chr11	77047977	77049057	Exon
chr11	77278152	77279212	Distal Intergenic
chr11	77279283	77280519	Distal Intergenic
chr11	77307513	77307727	Intron
chr11	77420146	77420789	Intron
chr11	77421538	77421760	Intron
chr11	77499300	77499703	Intron
chr11	77499956	77501593	Intron
chr11	77501661	77501997	Intron
chr11	77577095	77577894	Intron
chr11	77598862	77600120	Intron
chr11	77605535	77605743	Intron
chr11	77606949	77607330	Intron
chr11	77649990	77651801	Intron

chr11	77796971	77797605	Distal Intergenic
chr11	77977870	77978365	Intron
chr11	77979106	77980045	Intron
chr11	77990027	77990816	Intron
chr11	77991167	77992024	5' UTR
chr11	77992570	77993646	Intron
chr11	77995323	77995522	Intron
chr11	77995714	77996000	Intron
chr11	77996179	77997396	Intron
chr11	78059002	78060168	Intron
chr11	78060255	78060702	Intron
chr11	78196326	78196629	Intron
chr11	78260659	78260943	Intron
chr11	78279184	78279935	5' UTR
chr11	78325203	78325440	Distal Intergenic
chr11	78325730	78325938	Distal Intergenic
chr11	78348485	78349302	Distal Intergenic
chr11	78804738	78804971	Intron
chr11	78815874	78816253	Intron
chr11	79027748	79028364	Intron
chr11	79066275	79067649	Intron
chr11	79067927	79068184	Intron
chr11	79226944	79227550	Distal Intergenic
chr11	79296998	79298872	Distal Intergenic
chr11	79308689	79308907	Distal Intergenic
chr11	79517599	79517808	Distal Intergenic
chr11	79552815	79553059	Distal Intergenic
chr11	79685232	79685698	Distal Intergenic
chr11	79721776	79722910	Distal Intergenic
chr11	79775522	79775877	Distal Intergenic
chr11	79807105	79807502	Distal Intergenic
chr11	79807726	79807925	Distal Intergenic
chr11	80196470	80196695	Distal Intergenic
chr11	80196755	80197839	Distal Intergenic
chr11	80198217	80198682	Distal Intergenic
chr11	80226753	80226985	Distal Intergenic
chr11	80242129	80242806	Distal Intergenic
chr11	80253424	80253658	Distal Intergenic
chr11	80328918	80329235	Distal Intergenic
chr11	80346214	80346467	Distal Intergenic
chr11	80367238	80368204	Distal Intergenic
chr11	80588760	80589000	Distal Intergenic
chr11	80592726	80592973	Distal Intergenic
chr11	80597803	80598269	Distal Intergenic

chr11	81722082	81722385	Intron
chr11	81722541	81722929	Intron
chr11	81820507	81820840	Intron
chr11	81825174	81825614	Intron
chr11	81828429	81829486	Intron
chr11	81843581	81844483	Intron
chr11	81846197	81847604	Intron
chr11	81887207	81887486	Intron
chr11	81929487	81929776	Intron
chr11	81952729	81953047	Intron
chr11	81953084	81953557	Intron
chr11	81982533	81983794	Intron
chr11	81984908	81985816	Intron
chr11	82577943	82578142	Intron
chr11	82653379	82654820	Intron
chr11	82659542	82660029	Intron
chr11	82852946	82853153	Distal Intergenic
chr11	82853311	82853518	Distal Intergenic
chr11	82853564	82854044	Distal Intergenic
chr11	82857210	82857921	Distal Intergenic
chr11	83104915	83105137	Intron
chr11	83112044	83112447	Intron
chr11	83112529	83112761	Intron
chr11	83115337	83115559	Intron
chr11	83115690	83117316	Intron
chr11	83268241	83269955	Intron
chr11	83270349	83270548	Intron
chr11	83270875	83272457	Intron
chr11	83282020	83282908	Intron
chr11	83285668	83286236	Intron
chr11	83286924	83287234	Intron
chr11	83377393	83378020	Intron
chr11	83411055	83412900	Intron
chr11	83419461	83420814	Intron
chr11	83429916	83430501	Intron
chr11	83705189	83705479	Intron
chr11	83733899	83734268	Intron
chr11	83751985	83752403	Intron
chr11	83755687	83755905	Intron
chr11	83856550	83856906	Intron
chr11	83860844	83861064	Intron
chr11	83876355	83877040	Intron
chr11	83880988	83881507	Intron
chr11	83882109	83883120	Intron

chr11	83893786	83894170	Intron
chr11	83914420	83916498	Intron
chr11	83992567	83992949	Intron
chr11	83993799	83994109	Intron
chr11	83994241	83994502	Intron
chr11	84627314	84628032	Intron
chr11	84632077	84632320	Intron
chr11	84682912	84683416	Intron
chr11	84684220	84684579	Intron
chr11	84684640	84685002	Intron
chr11	84693132	84693400	Intron
chr11	84693520	84695635	Intron
chr11	84695770	84695974	Intron
chr11	84729994	84730920	Intron
chr11	84953779	84954253	Intron
chr11	84970718	84971279	Intron
chr11	85047838	85048088	Intron
chr11	85087057	85089337	Intron
chr11	85090652	85091514	Intron
chr11	85125384	85125784	Intron
chr11	85180751	85182223	Intron
chr11	85192315	85192815	Intron
chr11	85205251	85205500	Intron
chr11	85205701	85206014	Intron
chr11	85206159	85206358	Intron
chr11	85603176	85603925	Intron
chr11	85605919	85606452	Exon
chr11	85625986	85626411	Intron
chr11	85631654	85632699	Distal Intergenic
chr11	85944482	85945551	Distal Intergenic
chr11	86014970	86015265	Promoter
chr11	86137586	86138323	Distal Intergenic
chr11	86138369	86138568	Distal Intergenic
chr11	86169782	86171012	Intron
chr11	86171416	86171778	Intron
chr11	86205425	86206326	Intron
chr11	86206365	86206664	Intron
chr11	86320170	86322096	Intron
chr11	86358067	86358282	Intron
chr11	86390725	86393094	Distal Intergenic
chr11	86393130	86402330	Distal Intergenic
chr11	86402636	86402854	Distal Intergenic
chr11	86402965	86403297	Distal Intergenic
chr11	86403450	86405705	Distal Intergenic

chr11	86405760	86408369	Distal Intergenic
chr11	86413155	86416039	Distal Intergenic
chr11	86437120	86439282	Distal Intergenic
chr11	86441558	86442047	Distal Intergenic
chr11	86447789	86449680	Distal Intergenic
chr11	86459334	86466777	Distal Intergenic
chr11	86497538	86497766	Distal Intergenic
chr11	86570505	86571019	Intron
chr11	86571412	86572450	Intron
chr11	86573241	86573489	Intron
chr11	86573530	86574034	Intron
chr11	86923107	86923458	Intron
chr11	87123496	87124005	Distal Intergenic
chr11	87235817	87236328	Distal Intergenic
chr11	87236979	87237222	Distal Intergenic
chr11	87262315	87262778	Distal Intergenic
chr11	87275853	87276158	Distal Intergenic
chr11	87284974	87285176	Distal Intergenic
chr11	87404939	87405143	Distal Intergenic
chr11	87405917	87407010	Distal Intergenic
chr11	87492200	87492430	Distal Intergenic
chr11	87539009	87539337	Distal Intergenic
chr11	87539541	87539823	Distal Intergenic
chr11	87860900	87861102	Intron
chr11	87861207	87861839	Intron
chr11	87917886	87918943	Distal Intergenic
chr11	87919485	87919684	Distal Intergenic
chr11	87932043	87932263	Distal Intergenic
chr11	87932360	87932816	Distal Intergenic
chr11	87936332	87937879	Distal Intergenic
chr11	87964806	87965297	Distal Intergenic
chr11	87967134	87967457	Distal Intergenic
chr11	87967701	87968352	Distal Intergenic
chr11	87968794	87969143	Distal Intergenic
chr11	87974647	87974846	Distal Intergenic
chr11	87978055	87979367	Distal Intergenic
chr11	87985069	87986101	Distal Intergenic
chr11	87986160	87986827	Distal Intergenic
chr11	87987114	87987368	Distal Intergenic
chr11	88281269	88281468	Intron
chr11	88308994	88309965	Intron
chr11	88383956	88384752	Intron
chr11	88799194	88799631	Promoter
chr11	88857519	88858469	Distal Intergenic

chr11	88861458	88861809	Distal Intergenic
chr11	88884919	88885278	Distal Intergenic
chr11	88885589	88885925	Distal Intergenic
chr11	89048396	89049644	Distal Intergenic
chr11	89067575	89068432	Intron
chr11	89100889	89101182	Intron
chr11	89293080	89294032	Intron
chr11	89294185	89294765	Intron
chr11	89400953	89401485	Intron
chr11	90201495	90202927	Distal Intergenic
chr11	90211098	90211997	Distal Intergenic
chr11	90212750	90215285	Distal Intergenic
chr11	90215949	90217725	Distal Intergenic
chr11	90502739	90502963	Intron
chr11	90526199	90526959	Intron
chr11	90540056	90540419	Intron
chr11	92356589	92356799	Intron
chr11	92867347	92867918	Distal Intergenic
chr11	93529294	93530066	Exon
chr11	93801693	93802683	Intron
chr11	93816088	93816360	Intron
chr11	93816465	93816787	Intron
chr11	93816910	93817215	Intron
chr11	93817405	93819001	Intron
chr11	93828465	93829598	Intron
chr11	93848253	93850804	Intron
chr11	93855396	93857169	Intron
chr11	94002958	94003607	Distal Intergenic
chr11	94088789	94089027	Distal Intergenic
chr11	94089085	94089298	Distal Intergenic
chr11	94135849	94137555	Distal Intergenic
chr11	94183077	94183279	Intron
chr11	94231342	94232447	Promoter
chr11	94277685	94277952	Promoter
chr11	94316552	94316802	Exon
chr11	94317098	94318508	Intron
chr11	94318640	94318856	Exon
chr11	94359074	94360920	Distal Intergenic
chr11	94491106	94492161	Distal Intergenic
chr11	94819129	94819763	Distal Intergenic
chr11	94849069	94849400	Intron
chr11	94849442	94852165	Intron
chr11	94852207	94852493	Intron
chr11	94852749	94853000	Intron

chr11	94853916	94854124	Intron
chr11	94930530	94935997	Intron
chr11	94936060	94938011	Intron
chr11	94938111	94938613	Intron
chr11	94938803	94940277	Intron
chr11	94946162	94948664	Intron
chr11	94954267	94955683	Intron
chr11	94957414	94960146	Intron
chr11	94960227	94960435	Intron
chr11	94970376	94970670	Distal Intergenic
chr11	94998098	94998700	Distal Intergenic
chr11	95014709	95015226	Distal Intergenic
chr11	95134811	95136389	Distal Intergenic
chr11	95161115	95161795	Distal Intergenic
chr11	95171159	95171544	Distal Intergenic
chr11	95171651	95171925	Distal Intergenic
chr11	95245996	95246418	Distal Intergenic
chr11	95254354	95254574	Distal Intergenic
chr11	95254621	95255027	Distal Intergenic
chr11	95263751	95265265	Distal Intergenic
chr11	95276128	95276561	Distal Intergenic
chr11	95277091	95277648	Distal Intergenic
chr11	95294118	95294872	Distal Intergenic
chr11	95411005	95411236	Distal Intergenic
chr11	95411295	95412581	Distal Intergenic
chr11	95437784	95437983	Distal Intergenic
chr11	95638320	95639808	Intron
chr11	95661554	95661896	Distal Intergenic
chr11	95662049	95662424	Distal Intergenic
chr11	95679913	95681335	Distal Intergenic
chr11	95843212	95843475	Intron
chr11	95843566	95844886	Intron
chr11	95849564	95850430	Intron
chr11	95865740	95866287	Intron
chr11	95866771	95867242	Intron
chr11	95879357	95879607	Intron
chr11	95925864	95926190	Intron
chr11	96112869	96113085	Intron
chr11	96113497	96114528	Intron
chr11	96120907	96121106	Promoter
chr11	96121303	96121744	Promoter
chr11	96320100	96320482	Distal Intergenic
chr11	96608684	96609509	Distal Intergenic
chr11	96610131	96611214	Distal Intergenic

chr11	96651382	96651632	Distal Intergenic
chr11	96683849	96685265	Distal Intergenic
chr11	96685483	96686956	Distal Intergenic
chr11	96690215	96690574	Distal Intergenic
chr11	96691532	96691808	Distal Intergenic
chr11	96831034	96831900	Distal Intergenic
chr11	96833710	96835105	Distal Intergenic
chr11	96835678	96835914	Distal Intergenic
chr11	96836106	96837042	Distal Intergenic
chr11	96837168	96837459	Distal Intergenic
chr11	96837511	96838451	Distal Intergenic
chr11	96838768	96839003	Distal Intergenic
chr11	96841218	96841554	Distal Intergenic
chr11	96880459	96880724	Distal Intergenic
chr11	96880770	96881144	Distal Intergenic
chr11	97049863	97050098	Distal Intergenic
chr11	97050147	97050411	Distal Intergenic
chr11	97053257	97053668	Distal Intergenic
chr11	97068478	97068973	Distal Intergenic
chr11	97140726	97141175	Distal Intergenic
chr11	97163888	97164097	Distal Intergenic
chr11	97170091	97171630	Distal Intergenic
chr11	97177252	97177576	Distal Intergenic
chr11	97183241	97183579	Distal Intergenic
chr11	97233252	97234205	Distal Intergenic
chr11	97280716	97280935	Distal Intergenic
chr11	97281132	97281349	Distal Intergenic
chr11	97288937	97289616	Distal Intergenic
chr11	97289893	97290185	Distal Intergenic
chr11	97295904	97297958	Distal Intergenic
chr11	97299533	97299984	Distal Intergenic
chr11	97301150	97301349	Distal Intergenic
chr11	97301906	97303782	Distal Intergenic
chr11	97310464	97311884	Distal Intergenic
chr11	97332164	97333335	Distal Intergenic
chr11	97561135	97561496	Distal Intergenic
chr11	97561666	97561918	Distal Intergenic
chr11	97564885	97565084	Distal Intergenic
chr11	97610117	97610319	Distal Intergenic
chr11	97627593	97628182	Distal Intergenic
chr11	97641443	97641760	Distal Intergenic
chr11	97642046	97642357	Distal Intergenic
chr11	97642812	97643011	Distal Intergenic
chr11	97643357	97643640	Distal Intergenic

chr11	97661311	97661918	Distal Intergenic
chr11	97661961	97663164	Distal Intergenic
chr11	97665188	97665392	Distal Intergenic
chr11	97669158	97669519	Distal Intergenic
chr11	97670529	97670738	Distal Intergenic
chr11	97670832	97672454	Distal Intergenic
chr11	97672566	97673231	Distal Intergenic
chr11	97689007	97689360	Distal Intergenic
chr11	97810930	97811167	Distal Intergenic
chr11	97811283	97812121	Distal Intergenic
chr11	97823601	97823909	Distal Intergenic
chr11	97824041	97824382	Distal Intergenic
chr11	97849756	97850117	Distal Intergenic
chr11	97887751	97888502	Distal Intergenic
chr11	97888574	97888859	Distal Intergenic
chr11	97918715	97919484	Distal Intergenic
chr11	97923158	97923357	Distal Intergenic
chr11	97923568	97923878	Distal Intergenic
chr11	98064082	98064507	Distal Intergenic
chr11	98065434	98065668	Distal Intergenic
chr11	98280110	98280309	Distal Intergenic
chr11	98280541	98280921	Distal Intergenic
chr11	98449068	98449743	Distal Intergenic
chr11	98570061	98570304	Distal Intergenic
chr11	98570706	98570920	Distal Intergenic
chr11	98633790	98634316	Distal Intergenic
chr11	98634450	98635122	Distal Intergenic
chr11	98635458	98635663	Distal Intergenic
chr11	98635773	98636247	Distal Intergenic
chr11	98637065	98637544	Distal Intergenic
chr11	98655599	98656255	Distal Intergenic
chr11	98656365	98656584	Distal Intergenic
chr11	98656745	98657447	Distal Intergenic
chr11	98657552	98657797	Distal Intergenic
chr11	98697328	98698013	Distal Intergenic
chr11	98699607	98700660	Distal Intergenic
chr11	98793976	98794191	Distal Intergenic
chr11	98794440	98794709	Distal Intergenic
chr11	98797200	98797597	Distal Intergenic
chr11	98797770	98798815	Distal Intergenic
chr11	98885020	98885512	Distal Intergenic
chr11	98937810	98938876	Intron
chr11	98939045	98939268	Intron
chr11	98941934	98950740	Intron

chr11	98950909	98951185	Intron
chr11	98956222	98956894	Intron
chr11	98959676	98960679	Intron
chr11	98965743	98966931	Intron
chr11	98966986	98971584	Intron
chr11	98971653	98972007	Intron
chr11	99060956	99061538	Intron
chr11	99090812	99091439	Intron
chr11	99091615	99092462	Intron
chr11	99130138	99131140	Intron
chr11	99333586	99333858	Intron
chr11	99361380	99361866	Intron
chr11	99377788	99379246	Intron
chr11	99419427	99420025	Intron
chr11	99420097	99420481	Intron
chr11	99442535	99442994	Intron
chr11	99917194	99917963	Intron
chr11	99927347	99927572	Intron
chr11	99927609	99929022	Intron
chr11	99929073	99929483	Intron
chr11	99974657	99975530	Intron
chr11	99975586	99976006	Intron
chr11	100062808	100064708	Promoter
chr11	100065387	100065714	Intron
chr11	100066062	100071605	Intron
chr11	100109667	100109879	Intron
chr11	100109925	100111354	Intron
chr11	100113915	100114229	Intron
chr11	100114323	100115957	Intron
chr11	100116129	100117375	Intron
chr11	100118621	100119002	Intron
chr11	100119208	100122422	Intron
chr11	100122674	100123729	Intron
chr11	100139004	100140880	Intron
chr11	100142961	100143419	Intron
chr11	100144091	100144546	Intron
chr11	100144738	100153008	Intron
chr11	100153061	100153391	Intron
chr11	100159805	100160614	Intron
chr11	100165989	100167434	Intron
chr11	100181274	100181679	Intron
chr11	100247823	100251133	Distal Intergenic
chr11	100251862	100252084	Distal Intergenic
chr11	100252663	100252862	Distal Intergenic

chr11	100254306	100255617	Distal Intergenic
chr11	100255825	100258807	Distal Intergenic
chr11	100259522	100260030	Distal Intergenic
chr11	100267295	100269106	Distal Intergenic
chr11	100269212	100270062	Distal Intergenic
chr11	100272083	100275035	Distal Intergenic
chr11	100450339	100451361	Distal Intergenic
chr11	100452139	100452579	Distal Intergenic
chr11	100452770	100453058	Distal Intergenic
chr11	100480688	100480918	Distal Intergenic
chr11	100484227	100484525	Distal Intergenic
chr11	100579587	100580068	Intron
chr11	100580136	100581203	Intron
chr11	100581413	100584709	Intron
chr11	100598240	100599032	Intron
chr11	100599134	100599507	Intron
chr11	100792940	100793772	Intron
chr11	100823209	100825415	Intron
chr11	100826006	100827275	Intron
chr11	100827325	100827652	Intron
chr11	100830921	100831410	Promoter
chr11	100831498	100831714	Promoter
chr11	100870438	100870651	Distal Intergenic
chr11	100943938	100944303	Intron
chr11	100963498	100965944	Intron
chr11	100972663	100972909	Intron
chr11	100999923	101000332	Promoter
chr11	101033496	101033899	Intron
chr11	101034169	101034404	Intron
chr11	101114130	101114943	Distal Intergenic
chr11	101114999	101116216	Distal Intergenic
chr11	101118925	101119240	Distal Intergenic
chr11	101139048	101139281	Distal Intergenic
chr11	101139382	101140130	Distal Intergenic
chr11	101163223	101163542	Distal Intergenic
chr11	101309170	101309449	Distal Intergenic
chr11	101309524	101310369	Distal Intergenic
chr11	101488502	101489038	Distal Intergenic
chr11	101489079	101489827	Distal Intergenic
chr11	101548608	101549984	Distal Intergenic
chr11	101648073	101648272	Distal Intergenic
chr11	102046157	102046356	Intron
chr11	102106786	102106985	Distal Intergenic
chr11	102146216	102146461	Distal Intergenic

chr11	102156516	102157678	Distal Intergenic
chr11	102158564	102159046	Distal Intergenic
chr11	102178668	102178949	Distal Intergenic
chr11	102179402	102180477	Distal Intergenic
chr11	102244409	102244922	Intron
chr11	102249089	102249314	3' UTR
chr11	102270053	102270318	Intron
chr11	102286323	102286664	Intron
chr11	102327615	102328871	Distal Intergenic
chr11	102397414	102398632	3' UTR
chr11	102416811	102417117	Distal Intergenic
chr11	102505152	102506163	Distal Intergenic
chr11	102517407	102518062	Distal Intergenic
chr11	102742119	102742472	Exon
chr11	102785797	102786408	Distal Intergenic
chr11	102786554	102786979	Distal Intergenic
chr11	102820008	102820441	Intron
chr11	102857591	102858251	Distal Intergenic
chr11	102864537	102865233	Distal Intergenic
chr11	102889364	102889575	Distal Intergenic
chr11	102912317	102914125	Distal Intergenic
chr11	102955298	102955653	Intron
chr11	102955719	102956285	Intron
chr11	102956346	102956741	Intron
chr11	102957930	102959662	Intron
chr11	102961581	102961991	Promoter
chr11	102962373	102962665	Promoter
chr11	102963030	102963500	Promoter
chr11	102976376	102977344	Distal Intergenic
chr11	102993611	102994046	Exon
chr11	103098468	103098667	Intron
chr11	103107394	103108651	Intron
chr11	103123783	103124693	Exon
chr11	103131266	103131465	Intron
chr11	103131648	103131878	Intron
chr11	103160204	103160890	Intron
chr11	103173129	103173982	Exon
chr11	103195792	103197319	Intron
chr11	103201576	103202732	Intron
chr11	103222987	103223228	Intron
chr11	103223345	103223578	Intron
chr11	103223629	103225453	Intron
chr11	103239469	103240137	Intron
chr11	103242954	103244408	Intron

chr11	103257835	103258721	Intron
chr11	103545597	103545847	Distal Intergenic
chr11	103546019	103547263	Distal Intergenic
chr11	103550254	103550586	Distal Intergenic
chr11	103551052	103551284	Distal Intergenic
chr11	103558451	103559825	Distal Intergenic
chr11	104149832	104150802	Distal Intergenic
chr11	104150842	104151543	Distal Intergenic
chr11	104164808	104165044	Distal Intergenic
chr11	104239189	104239452	Distal Intergenic
chr11	104302037	104302704	Distal Intergenic
chr11	104302776	104303015	Distal Intergenic
chr11	104303827	104305146	Distal Intergenic
chr11	104411384	104411621	Distal Intergenic
chr11	104534779	104535602	Distal Intergenic
chr11	104553298	104554003	Distal Intergenic
chr11	105021163	105021362	Distal Intergenic
chr11	105042941	105043268	Distal Intergenic
chr11	105043320	105044865	Distal Intergenic
chr11	105191359	105192788	Distal Intergenic
chr11	105301117	105301485	Distal Intergenic
chr11	105328647	105329130	Distal Intergenic
chr11	105329180	105329382	Distal Intergenic
chr11	105654048	105654263	Intron
chr11	105684331	105685094	Intron
chr11	105744899	105745124	Intron
chr11	105962770	105963138	Intron
chr11	106054467	106054836	Distal Intergenic
chr11	106055147	106055408	Distal Intergenic
chr11	106078252	106078934	Distal Intergenic
chr11	106116765	106117960	Distal Intergenic
chr11	106133279	106133538	Exon
chr11	106277282	106278485	Distal Intergenic
chr11	106331349	106332531	Distal Intergenic
chr11	106345017	106345812	Distal Intergenic
chr11	106362128	106363917	Distal Intergenic
chr11	106510315	106510572	Distal Intergenic
chr11	106519252	106519995	Distal Intergenic
chr11	106526532	106528490	Distal Intergenic
chr11	106532272	106532699	Distal Intergenic
chr11	106535766	106536770	Distal Intergenic
chr11	106574205	106576237	Intron
chr11	106583786	106584408	Intron
chr11	106641045	106641502	Intron

chr11	106648537	106648827	Intron
chr11	106649577	106649786	Intron
chr11	106650292	106651129	Intron
chr11	106758084	106758334	Intron
chr11	106860028	106860312	Intron
chr11	106860520	106860825	Intron
chr11	106873454	106873963	Intron
chr11	106993775	106994012	Distal Intergenic
chr11	106999170	106999690	Distal Intergenic
chr11	107009842	107010051	Distal Intergenic
chr11	107010163	107011917	Distal Intergenic
chr11	107018101	107018678	Distal Intergenic
chr11	107018928	107019212	Distal Intergenic
chr11	107050234	107050464	Distal Intergenic
chr11	107220206	107220567	Intron
chr11	107278199	107279019	Intron
chr11	107289258	107290138	Intron
chr11	107309965	107311357	Intron
chr11	107319528	107320085	Intron
chr11	107331511	107331947	Distal Intergenic
chr11	107340836	107341387	Distal Intergenic
chr11	107880769	107882214	Intron
chr11	107982431	107982643	Distal Intergenic
chr11	108076907	108077159	Intron
chr11	108078117	108078867	Intron
chr11	108129236	108129476	Intron
chr11	108144057	108144802	Intron
chr11	108209467	108209829	Intron
chr11	108226726	108227204	Exon
chr11	108613782	108614045	Intron
chr11	108614340	108615079	Intron
chr11	108618981	108619252	Intron
chr11	108619294	108619821	Intron
chr11	108758949	108760244	Intron
chr11	109216922	109217916	Distal Intergenic
chr11	109221557	109221791	Distal Intergenic
chr11	109221885	109223850	Distal Intergenic
chr11	109470275	109470576	Distal Intergenic
chr11	109471110	109471593	Distal Intergenic
chr11	109471699	109472034	Distal Intergenic
chr11	109522815	109523014	Distal Intergenic
chr11	109523505	109523817	Distal Intergenic
chr11	109530789	109531342	Distal Intergenic
chr11	109531413	109533181	Distal Intergenic

chr11	109533219	109533741	Distal Intergenic
chr11	109559238	109560526	Distal Intergenic
chr11	109678290	109678491	Distal Intergenic
chr11	109721475	109721940	Distal Intergenic
chr11	110240559	110241660	Intron
chr11	110300212	110300411	Promoter
chr11	110309983	110312125	Intron
chr11	110333206	110333484	3' UTR
chr11	110333565	110333971	3' UTR
chr11	110640357	110641272	Distal Intergenic
chr11	110643178	110643719	Distal Intergenic
chr11	110643823	110644034	Distal Intergenic
chr11	112027126	112027692	Intron
chr11	112060510	112061715	Intron
chr11	112112884	112113128	Intron
chr11	112113282	112115212	Intron
chr11	112163072	112164544	Distal Intergenic
chr11	112164597	112164821	Distal Intergenic
chr11	112331959	112332272	Distal Intergenic
chr11	112481815	112482903	Distal Intergenic
chr11	112492650	112493371	Distal Intergenic
chr11	112522287	112522583	Distal Intergenic
chr11	112522678	112523022	Distal Intergenic
chr11	112523126	112523686	Distal Intergenic
chr11	112849262	112849609	Intron
chr11	112918029	112918528	Intron
chr11	112921797	112923023	Intron
chr11	112924171	112925152	Intron
chr11	112935489	112936677	Intron
chr11	112961729	112965483	Intron
chr11	112965806	112967841	Intron
chr11	112994203	112994440	Intron
chr11	113057642	113057854	Intron
chr11	113057954	113058337	Intron
chr11	113059582	113060353	Intron
chr11	113060414	113061447	Intron
chr11	113070597	113070946	Intron
chr11	113398142	113398988	Distal Intergenic
chr11	113612058	113612390	Intron
chr11	113612516	113613119	Intron
chr11	113616273	113617843	Intron
chr11	113628192	113628494	Exon
chr11	114529927	114530170	Distal Intergenic
chr11	114530838	114531073	Distal Intergenic

chr11	115033921	115035678	Distal Intergenic
chr11	115375319	115375718	Promoter
chr11	115425560	115425763	Distal Intergenic
chr11	115608260	115608544	Distal Intergenic
chr11	115637202	115637457	Distal Intergenic
chr11	115637720	115638050	Distal Intergenic
chr11	115638132	115639084	Distal Intergenic
chr11	115639153	115639548	Distal Intergenic
chr11	115688508	115688724	Distal Intergenic
chr11	115730459	115730889	Distal Intergenic
chr11	115794938	115795148	Distal Intergenic
chr11	115805432	115808068	Distal Intergenic
chr11	115815572	115817192	Distal Intergenic
chr11	115817285	115819440	Distal Intergenic
chr11	115830444	115833158	Distal Intergenic
chr11	115833239	115833519	Distal Intergenic
chr11	115835096	115836308	Distal Intergenic
chr11	115836714	115838324	Distal Intergenic
chr11	115885200	115885467	Distal Intergenic
chr11	115892079	115892440	Distal Intergenic
chr11	116028584	116028848	Distal Intergenic
chr11	116028924	116029200	Distal Intergenic
chr11	116029242	116029489	Distal Intergenic
chr11	116116208	116116609	Distal Intergenic
chr11	116117226	116118341	Distal Intergenic
chr11	116267502	116267936	Distal Intergenic
chr11	116383122	116383718	Distal Intergenic
chr11	116482318	116482686	Distal Intergenic
chr11	116482735	116483013	Distal Intergenic
chr11	116483317	116483606	Distal Intergenic
chr11	116703906	116704168	Downstream
chr11	116704311	116704612	Downstream
chr11	116714245	116714560	3' UTR
chr11	116714625	116714839	3' UTR
chr11	116942577	116943048	Intron
chr11	116943880	116944079	Intron
chr11	117061158	117061515	Exon
chr11	117063112	117064105	Exon
chr11	117064166	117065089	Exon
chr11	117065630	117067571	3' UTR
chr11	117068841	117069545	Promoter
chr11	117117217	117117521	Intron
chr11	117120617	117120949	Intron
chr11	117120994	117122296	Intron

chr11	117124856	117125120	Intron
chr11	117125171	117126021	Intron
chr11	117126161	117126378	Intron
chr11	117126419	117127102	Intron
chr11	117185082	117185438	Intron
chr11	117185571	117185938	Intron
chr11	117195277	117195476	Intron
chr11	117195517	117196161	Intron
chr11	117429514	117430863	Intron
chr11	117568390	117568921	Intron
chr11	117642144	117643383	Intron
chr11	117649749	117649948	Intron
chr11	117650232	117650481	Intron
chr11	117651748	117652002	Intron
chr11	117652111	117652633	Intron
chr11	117652750	117652963	Intron
chr11	117672101	117672338	Intron
chr11	117685594	117686109	Intron
chr11	117686531	117687954	Promoter
chr11	117704645	117705367	Intron
chr11	117706323	117706564	Intron
chr11	117709509	117709836	Exon
chr11	117710045	117710356	Exon
chr11	117710640	117711051	Exon
chr11	117803053	117803508	Distal Intergenic
chr11	117819073	117819517	Distal Intergenic
chr11	117819587	117820022	Distal Intergenic
chr11	117848743	117849013	Distal Intergenic
chr11	117851372	117851602	Distal Intergenic
chr11	117855243	117855886	Distal Intergenic
chr11	117868354	117868947	Intron
chr11	117869222	117869755	Exon
chr11	117871057	117871583	3' UTR
chr11	117873378	117873662	Distal Intergenic
chr11	117873774	117874776	Distal Intergenic
chr11	117890608	117890861	Intron
chr11	118014270	118015481	Promoter
chr11	118017647	118019358	Intron
chr11	118019454	118019714	Intron
chr11	118021565	118022576	Intron
chr11	118035932	118037166	3' UTR
chr11	118048777	118049021	Distal Intergenic
chr11	118049107	118049307	Distal Intergenic
chr11	118073953	118074233	Promoter

chr11	118074272	118074484	Promoter
chr11	118076839	118078628	Intron
chr11	118208016	118209030	Downstream
chr11	118712874	118713631	Distal Intergenic
chr11	118966572	118967709	Promoter
chr11	118968713	118969404	3' UTR
chr11	118989313	118989687	Distal Intergenic
chr11	119011625	119012328	Distal Intergenic
chr11	119012765	119013754	Distal Intergenic
chr11	119046110	119046454	Intron
chr11	119254688	119254900	Intron
chr11	119263382	119264830	Intron
chr11	119264896	119265128	Intron
chr11	119546984	119547736	Intron
chr11	119556953	119557938	Intron
chr11	119679141	119679397	Distal Intergenic
chr11	119718021	119718511	Distal Intergenic
chr11	119794678	119795501	Distal Intergenic
chr11	119798205	119799302	Distal Intergenic
chr11	119829646	119830913	Distal Intergenic
chr11	119836716	119838113	Distal Intergenic
chr11	119850059	119850556	Distal Intergenic
chr11	120114380	120114579	Intron
chr11	120210166	120211276	Intron
chr11	120211469	120211725	Intron
chr11	120368496	120370407	Distal Intergenic
chr11	120371276	120371603	Distal Intergenic
chr11	120414325	120414534	Intron
chr11	120416105	120416400	Intron
chr11	120416445	120416692	Intron
chr11	120435191	120435560	Promoter
chr11	120435702	120435901	Promoter
chr11	120491306	120492270	Intron
chr11	120492419	120492634	Intron
chr11	120493387	120493647	Intron
chr11	120493754	120494171	Intron
chr11	120534689	120534895	Intron
chr11	120559036	120559459	Intron
chr11	120559517	120559798	Intron
chr11	120565414	120565681	Intron
chr11	120569197	120569896	Intron
chr11	120593375	120593871	Intron
chr11	120594184	120594726	Intron
chr11	120684614	120684975	Intron

chr11	120807525	120807747	Intron
chr11	120807796	120808007	Intron
chr11	120845460	120846712	Intron
chr11	120846921	120847233	Intron
chr11	120852285	120852613	Intron
chr11	121098029	121098248	Distal Intergenic
chr11	121376858	121378533	Intron
chr11	121380797	121381190	Intron
chr11	121381398	121381936	Intron
chr11	121405019	121405419	Intron
chr11	121405516	121405920	Intron
chr11	121405961	121407145	Intron
chr11	121458523	121458952	Exon
chr11	121462660	121463329	Intron
chr11	121507529	121507853	Distal Intergenic
chr11	121507910	121508761	Distal Intergenic
chr12	3082039	3082857	Intron
chr12	3174126	3174331	Distal Intergenic
chr12	3174382	3174608	Distal Intergenic
chr12	3284745	3285300	Intron
chr12	3308618	3309014	Intron
chr12	3310153	3310963	5' UTR
chr12	3319376	3319811	Intron
chr12	3329553	3330404	Intron
chr12	3348946	3351143	Intron
chr12	3351991	3353029	Intron
chr12	3426088	3426692	Distal Intergenic
chr12	3426775	3427106	Distal Intergenic
chr12	3427176	3428031	Exon
chr12	3537313	3538768	Intron
chr12	3582648	3582854	Intron
chr12	3719408	3719891	Intron
chr12	3746137	3746412	Intron
chr12	4233442	4234718	Distal Intergenic
chr12	4292533	4292755	Distal Intergenic
chr12	4357022	4357539	Distal Intergenic
chr12	4595556	4595859	Downstream
chr12	4596968	4598278	3' UTR
chr12	4598827	4599505	3' UTR
chr12	4599615	4600006	Exon
chr12	4600170	4600740	Exon
chr12	4606129	4606635	Intron
chr12	4613368	4613941	Intron
chr12	4824054	4824253	Distal Intergenic

chr12	4824318	4825884	Distal Intergenic
chr12	4825925	4826854	Distal Intergenic
chr12	4924174	4925547	Intron
chr12	4981351	4981600	Distal Intergenic
chr12	5352909	5353128	Distal Intergenic
chr12	5353250	5354066	Distal Intergenic
chr12	5356284	5357896	Distal Intergenic
chr12	5357936	5358177	Distal Intergenic
chr12	8077481	8078525	Exon
chr12	8082410	8082687	Exon
chr12	8082746	8083021	Intron
chr12	8083104	8083434	Exon
chr12	8214964	8215346	Intron
chr12	8369274	8369591	Distal Intergenic
chr12	8371151	8371755	Downstream
chr12	8378611	8380004	Promoter
chr12	8385401	8385890	Intron
chr12	8385947	8386399	Intron
chr12	8386519	8387429	Exon
chr12	8393070	8394708	Promoter
chr12	8394905	8396890	Promoter
chr12	8494912	8495131	Distal Intergenic
chr12	8506555	8506832	Downstream
chr12	8516434	8518777	Intron
chr12	8526226	8526593	Intron
chr12	8526750	8527479	Intron
chr12	8527521	8528557	Intron
chr12	8528609	8528975	Intron
chr12	8559023	8559278	Distal Intergenic
chr12	8680638	8680837	Distal Intergenic
chr12	8681444	8682748	Distal Intergenic
chr12	8767336	8768720	Distal Intergenic
chr12	8778810	8791841	Distal Intergenic
chr12	8791962	8792642	Distal Intergenic
chr12	8793445	8794013	Distal Intergenic
chr12	8803815	8805346	Exon
chr12	8805916	8807188	Exon
chr12	8812179	8813156	Intron
chr12	8813750	8814192	Intron
chr12	8814405	8814618	Promoter
chr12	8815834	8820092	Promoter
chr12	8824592	8824946	Distal Intergenic
chr12	8825106	8825376	Distal Intergenic
chr12	8828744	8829022	Distal Intergenic

chr12	8831859	8832242	Distal Intergenic
chr12	8832511	8832723	Distal Intergenic
chr12	8833416	8834660	Promoter
chr12	8861096	8861733	Intron
chr12	8864957	8867386	5' UTR
chr12	8867547	8868492	Intron
chr12	8871268	8871553	Intron
chr12	8871593	8871842	Intron
chr12	8871884	8872267	Intron
chr12	8880425	8883191	Intron
chr12	8891396	8892744	Intron
chr12	8903656	8904066	Intron
chr12	8922839	8923711	Intron
chr12	8927720	8930486	3' UTR
chr12	8930548	8930824	Intron
chr12	8934630	8935069	Intron
chr12	8935493	8935797	Exon
chr12	8976571	8976962	Intron
chr12	8981444	8981817	Intron
chr12	9072836	9073367	Intron
chr12	9118533	9120113	Distal Intergenic
chr12	9236587	9236993	Intron
chr12	9238815	9239566	Intron
chr12	9303274	9303517	Exon
chr12	9303685	9304079	Intron
chr12	9304116	9304381	Exon
chr12	9305273	9305502	Exon
chr12	9305665	9306495	Exon
chr12	9310383	9311784	Exon
chr12	9694658	9696932	Distal Intergenic
chr12	9862539	9862874	Distal Intergenic
chr12	10397595	10398306	Distal Intergenic
chr12	11271902	11272766	Intron
chr12	11273438	11274193	Intron
chr12	11394215	11399749	Intron
chr12	12549431	12549786	Intron
chr12	12895264	12895652	Intron
chr12	12988276	12988475	Distal Intergenic
chr12	12988644	12989327	Distal Intergenic
chr12	12989474	12990430	Distal Intergenic
chr12	12990648	12991229	Distal Intergenic
chr12	13083209	13084414	Distal Intergenic
chr12	13099373	13100074	Intron
chr12	13182633	13184083	Intron

chr12	14075632	14075999	Intron
chr12	14076208	14076542	Intron
chr12	14216390	14216620	Distal Intergenic
chr12	14216678	14217320	Distal Intergenic
chr12	14219516	14220089	Distal Intergenic
chr12	14293136	14293602	Distal Intergenic
chr12	14293654	14293853	Distal Intergenic
chr12	14307970	14309911	Distal Intergenic
chr12	15839409	15839623	Intron
chr12	15839739	15839968	Intron
chr12	16673891	16674149	Distal Intergenic
chr12	16674209	16674531	Distal Intergenic
chr12	16840986	16841689	Distal Intergenic
chr12	16841749	16842216	Distal Intergenic
chr12	16847648	16848446	Distal Intergenic
chr12	16852183	16852499	Distal Intergenic
chr12	16860199	16860580	Distal Intergenic
chr12	16901758	16903386	Distal Intergenic
chr12	16926831	16927756	Distal Intergenic
chr12	17006024	17006290	Distal Intergenic
chr12	17006979	17007224	Distal Intergenic
chr12	17046215	17046622	Distal Intergenic
chr12	17046888	17047559	Distal Intergenic
chr12	17227237	17228311	Distal Intergenic
chr12	17273767	17273970	Distal Intergenic
chr12	17306367	17307424	Distal Intergenic
chr12	17309951	17310722	Distal Intergenic
chr12	17310901	17311155	Distal Intergenic
chr12	17354624	17354893	Distal Intergenic
chr12	17354983	17355714	Distal Intergenic
chr12	17394121	17394906	Distal Intergenic
chr12	17397069	17397462	Distal Intergenic
chr12	17426470	17428110	Distal Intergenic
chr12	17516899	17518364	Distal Intergenic
chr12	17547390	17547658	Distal Intergenic
chr12	17549133	17550215	Distal Intergenic
chr12	17559115	17559771	Distal Intergenic
chr12	17700619	17701433	Distal Intergenic
chr12	17701472	17701798	Distal Intergenic
chr12	17727907	17728521	Distal Intergenic
chr12	17735867	17736790	Distal Intergenic
chr12	17739541	17739970	Distal Intergenic
chr12	17768647	17768846	Distal Intergenic
chr12	17768885	17770231	Distal Intergenic

chr12	21172283	21172701	Exon
chr12	21182279	21182637	Intron
chr12	21232824	21233605	Intron
chr12	21378779	21380144	Intron
chr12	21422491	21422897	Exon
chr12	21422943	21423451	Intron
chr12	21430626	21431574	Intron
chr12	22077763	22077963	3' UTR
chr12	25183409	25183643	Distal Intergenic
chr12	25202626	25203896	Distal Intergenic
chr12	25210179	25210585	Intron
chr12	25220278	25223608	5' UTR
chr12	25235389	25236081	Intron
chr12	25237420	25238092	Intron
chr12	25277648	25277894	Intron
chr12	25278018	25278232	Intron
chr12	25278578	25278777	Intron
chr12	25284491	25285582	Intron
chr12	25391898	25393203	Intron
chr12	25393300	25394993	Intron
chr12	25408407	25408820	Distal Intergenic
chr12	25408866	25409351	Distal Intergenic
chr12	25561605	25562826	Distal Intergenic
chr12	25647950	25649579	3' UTR
chr12	25651549	25652003	Intron
chr12	25690507	25690717	Intron
chr12	25697462	25698010	Intron
chr12	25700295	25700592	Intron
chr12	25779737	25779936	Intron
chr12	25780151	25780482	Intron
chr12	25781080	25781364	Intron
chr12	25795138	25796237	Intron
chr12	26112534	26114479	Promoter
chr12	26859803	26860043	Intron
chr12	28174769	28175708	Distal Intergenic
chr12	28176913	28177737	Distal Intergenic
chr12	28683412	28685257	Intron
chr12	28685321	28685612	Intron
chr12	28685649	28685932	Intron
chr12	28686933	28687223	Intron
chr12	29320166	29321095	Intron
chr12	29412083	29412451	Intron
chr12	29482406	29483143	Intron
chr12	29489288	29490644	Downstream

chr12	30434319	30434685	Distal Intergenic
chr12	30677149	30677482	Distal Intergenic
chr12	30677703	30677978	Distal Intergenic
chr12	30688437	30688754	Distal Intergenic
chr12	31596206	31596405	Intron
chr12	31596572	31596771	Intron
chr12	31743941	31745101	Promoter
chr12	31745204	31745810	Intron
chr12	31745914	31746331	Intron
chr12	31746761	31747404	Exon
chr12	31753979	31754818	Exon
chr12	31758955	31759381	Intron
chr12	31760044	31760368	Intron
chr12	31760906	31761107	Intron
chr12	31762986	31763499	Intron
chr12	31768045	31769011	Exon
chr12	31870132	31870489	Intron
chr12	31871471	31872098	Intron
chr12	31955830	31956478	Distal Intergenic
chr12	31973571	31974380	Distal Intergenic
chr12	31976929	31977196	Distal Intergenic
chr12	31977249	31977778	Distal Intergenic
chr12	32036058	32036257	Distal Intergenic
chr12	32100924	32101216	Distal Intergenic
chr12	32101319	32101546	Distal Intergenic
chr12	32198481	32198937	Distal Intergenic
chr12	32248653	32249481	Distal Intergenic
chr12	32339226	32339645	Intron
chr12	32532985	32534368	Distal Intergenic
chr12	32634089	32634308	Distal Intergenic
chr12	32658583	32658976	Intron
chr12	32676954	32677292	Intron
chr12	32681561	32681839	Intron
chr12	32742088	32742504	Intron
chr12	33119478	33120523	Distal Intergenic
chr12	33306187	33306463	Distal Intergenic
chr12	33306580	33307764	Distal Intergenic
chr12	33307852	33308264	Distal Intergenic
chr12	33308652	33309115	Distal Intergenic
chr12	33504425	33504881	Distal Intergenic
chr12	33639397	33639807	Distal Intergenic
chr12	33679504	33679771	Distal Intergenic
chr12	33680143	33680409	Distal Intergenic
chr12	33704779	33706276	Distal Intergenic

chr12	33857430	33858155	Distal Intergenic
chr12	33858558	33859011	Distal Intergenic
chr12	33866625	33866837	Distal Intergenic
chr12	33970490	33970689	Distal Intergenic
chr12	34114631	34114865	Distal Intergenic
chr12	34724782	34725953	Distal Intergenic
chr12	34760766	34760967	Distal Intergenic
chr12	35731878	35732298	Distal Intergenic
chr12	35893889	35894629	Distal Intergenic
chr12	35894714	35895521	Distal Intergenic
chr12	35902097	35902484	Distal Intergenic
chr12	36218889	36219088	Distal Intergenic
chr12	36247460	36248310	Distal Intergenic
chr12	36337186	36337385	Distal Intergenic
chr12	36340971	36341237	Distal Intergenic
chr12	36341396	36341603	Distal Intergenic
chr12	36355578	36357865	Distal Intergenic
chr12	36387105	36388179	Distal Intergenic
chr12	36388358	36388633	Distal Intergenic
chr12	36397337	36397833	Distal Intergenic
chr12	36418920	36419223	Distal Intergenic
chr12	36860741	36861070	Distal Intergenic
chr12	36883762	36884132	Distal Intergenic
chr12	36884171	36884562	Distal Intergenic
chr12	36942086	36942320	Distal Intergenic
chr12	36942398	36944206	Distal Intergenic
chr12	36989009	36989223	Distal Intergenic
chr12	36993720	36995591	Distal Intergenic
chr12	37016954	37017218	Distal Intergenic
chr12	37107959	37108337	Distal Intergenic
chr12	37108487	37108768	Distal Intergenic
chr12	37186685	37187391	Distal Intergenic
chr12	40556829	40558927	Distal Intergenic
chr12	40558981	40559259	Distal Intergenic
chr12	40608816	40609577	Distal Intergenic
chr12	40646839	40647270	Intron
chr12	40657042	40657863	Exon
chr12	40948560	40949116	Intron
chr12	40960555	40960773	Intron
chr12	41310833	41312210	Intron
chr12	41312299	41312502	Exon
chr12	41614383	41614879	Intron
chr12	41615193	41616177	Intron
chr12	41625128	41625900	Intron

chr12	41634437	41636566	Intron
chr12	41806288	41808006	Intron
chr12	41808384	41808592	Intron
chr12	41810686	41812745	Intron
chr12	41812786	41813353	Intron
chr12	41816143	41816414	Intron
chr12	41816875	41817273	Intron
chr12	43050221	43051347	Distal Intergenic
chr12	46148322	46148638	Intron
chr12	46160905	46161286	Intron
chr12	46161349	46161947	Intron
chr12	46162226	46162614	Intron
chr12	46641486	46641800	Intron
chr12	49055415	49055776	Intron
chr12	49120877	49121339	Promoter
chr12	50146988	50147333	Intron
chr12	50577924	50578225	Intron
chr12	50613402	50614446	Intron
chr12	51070377	51070588	Intron
chr12	51070809	51071008	Intron
chr12	52384722	52385208	Intron
chr12	52448820	52449877	Promoter
chr12	52478555	52479092	Intron
chr12	52791419	52792689	Intron
chr12	52792982	52793227	Intron
chr12	52793308	52793572	Intron
chr12	52793615	52793833	Exon
chr12	52793913	52794148	Exon
chr12	52794289	52796577	Exon
chr12	52804777	52805228	Distal Intergenic
chr12	52822574	52823838	Intron
chr12	52834948	52835156	Distal Intergenic
chr12	52835210	52837382	Distal Intergenic
chr12	52929197	52930338	Distal Intergenic
chr12	53604149	53604554	3' UTR
chr12	53605605	53606253	Exon
chr12	53630290	53631268	Distal Intergenic
chr12	53705036	53705235	Intron
chr12	53705275	53705509	Intron
chr12	53705836	53706044	Intron
chr12	53726388	53726996	Intron
chr12	53736768	53737004	Distal Intergenic
chr12	53737172	53737775	Distal Intergenic
chr12	53793198	53793748	Intron

chr12	53801458	53801657	Intron
chr12	53801714	53802564	Intron
chr12	53802692	53803895	Exon
chr12	53882423	53882626	Intron
chr12	53883116	53884984	Intron
chr12	53935323	53937735	3' UTR
chr12	53937789	53938026	Intron
chr12	54051650	54051962	Distal Intergenic
chr12	54247946	54248242	Distal Intergenic
chr12	54989490	54990002	Distal Intergenic
chr12	55049649	55050582	Distal Intergenic
chr12	55288675	55289709	Distal Intergenic
chr12	55292855	55293101	Distal Intergenic
chr12	55293147	55293736	Distal Intergenic
chr12	55301728	55302222	Distal Intergenic
chr12	55302452	55302876	Distal Intergenic
chr12	55342840	55343130	3' UTR
chr12	55387601	55388980	Distal Intergenic
chr12	55699113	55699406	Distal Intergenic
chr12	55720243	55722592	Distal Intergenic
chr12	55741116	55742101	Distal Intergenic
chr12	55796281	55797794	Distal Intergenic
chr12	55811974	55812188	Distal Intergenic
chr12	55896401	55897387	Distal Intergenic
chr12	55963066	55963418	Distal Intergenic
chr12	56041730	56042483	Distal Intergenic
chr12	56086666	56087545	Exon
chr12	56087637	56087860	Exon
chr12	56096285	56096552	Promoter
chr12	56096934	56097258	Promoter
chr12	56108809	56109033	Promoter
chr12	56181503	56181711	Intron
chr12	56256350	56256734	Distal Intergenic
chr12	56331727	56332028	5' UTR
chr12	56403785	56404162	Distal Intergenic
chr12	56499251	56500217	Intron
chr12	56500758	56501045	Exon
chr12	56563523	56564343	Exon
chr12	56564968	56565745	Exon
chr12	56565976	56568630	Exon
chr12	56572660	56572948	Exon
chr12	56579678	56579916	Intron
chr12	56590022	56590309	Distal Intergenic
chr12	56591650	56591877	Distal Intergenic

chr12	56592045	56592364	Distal Intergenic
chr12	57419502	57420557	Downstream
chr12	57426613	57427756	Intron
chr12	57458657	57459302	Intron
chr12	57880205	57881569	Promoter
chr12	57895569	57896294	Intron
chr12	57904979	57905894	Promoter
chr12	60112272	60112563	Intron
chr12	60112599	60112814	Intron
chr12	60113178	60114347	Intron
chr12	60161856	60162428	5' UTR
chr12	60162479	60162678	Intron
chr12	60168077	60168307	Intron
chr12	60177422	60179576	3' UTR
chr12	66066294	66066924	Distal Intergenic
chr12	66067177	66067552	Distal Intergenic
chr12	66071609	66071981	Distal Intergenic
chr12	66076915	66077611	Distal Intergenic
chr12	66137502	66137758	Distal Intergenic
chr12	66138038	66138516	Distal Intergenic
chr12	66174735	66174972	Intron
chr12	66273039	66273440	Intron
chr12	70259489	70260155	Distal Intergenic
chr12	70336565	70336940	Intron
chr12	70340699	70341063	Intron
chr12	70462581	70463418	Distal Intergenic
chr12	70463496	70463790	Distal Intergenic
chr12	70464954	70465231	Distal Intergenic
chr12	70472485	70472874	Distal Intergenic
chr12	70473107	70473404	Distal Intergenic
chr12	70473879	70474925	Distal Intergenic
chr12	70523584	70524488	Distal Intergenic
chr12	70537695	70538714	Distal Intergenic
chr12	70547494	70547892	Distal Intergenic
chr12	70548186	70548748	Distal Intergenic
chr12	70548863	70549095	Distal Intergenic
chr12	70603162	70603514	Distal Intergenic
chr12	70603558	70603757	Distal Intergenic
chr12	70638096	70640493	Promoter
chr12	70844764	70845833	Distal Intergenic
chr12	70925656	70926772	Exon
chr12	70935176	70935788	Intron
chr12	70979794	70981547	Exon
chr12	70981619	70982132	Intron

chr12	70982197	70982481	Intron
chr12	70982580	70983261	Intron
chr12	70985912	70986712	3' UTR
chr12	70989733	70989977	Exon
chr12	70992868	70993815	Intron
chr12	71087459	71087902	Intron
chr12	71098457	71100849	Intron
chr12	71645485	71646637	Intron
chr12	71655116	71657631	Intron
chr12	71657669	71657941	Intron
chr12	71678673	71679534	Intron
chr12	71690477	71690704	Intron
chr12	71691174	71692176	Intron
chr12	71701783	71702136	Intron
chr12	71717388	71717780	Intron
chr12	71724814	71727665	Intron
chr12	71727763	71730907	Intron
chr12	71748080	71752217	Intron
chr12	71752287	71753254	Intron
chr12	71754171	71754414	Intron
chr12	71760396	71762105	Intron
chr12	71771319	71775506	Intron
chr12	71795775	71797265	Intron
chr12	71806791	71809356	Intron
chr12	71812692	71814299	Intron
chr12	71821787	71822381	Intron
chr12	71840415	71841806	Intron
chr12	71925738	71926012	Intron
chr12	71926054	71926253	Intron
chr12	71926913	71927636	Intron
chr12	71931173	71931402	Intron
chr12	71931578	71932244	Intron
chr12	71936032	71936391	Intron
chr12	71991795	71992833	Distal Intergenic
chr12	72001878	72002115	Downstream
chr12	72008121	72009101	Exon
chr12	72009143	72009579	Intron
chr12	72009891	72010091	Intron
chr12	72010925	72011228	Intron
chr12	72038557	72038869	Exon
chr12	72068959	72069449	Intron
chr12	72069520	72069906	Intron
chr12	72075959	72076461	Distal Intergenic
chr12	72971727	72971996	Intron

chr12	72985981	72986306	Intron
chr12	72988567	72991210	Intron
chr12	73084025	73084360	Distal Intergenic
chr12	73084648	73084998	Distal Intergenic
chr12	73103778	73104524	Distal Intergenic
chr12	73639517	73639921	Distal Intergenic
chr12	73845844	73846051	Distal Intergenic
chr12	73861449	73862013	Distal Intergenic
chr12	74248002	74248478	Distal Intergenic
chr12	74248559	74248788	Distal Intergenic
chr12	74329980	74331000	Distal Intergenic
chr12	74331074	74331291	Distal Intergenic
chr12	74331365	74331644	Distal Intergenic
chr12	74395533	74397081	Distal Intergenic
chr12	74717117	74718996	Distal Intergenic
chr12	74819171	74820007	Distal Intergenic
chr12	74826400	74827193	Distal Intergenic
chr12	74827353	74827642	Distal Intergenic
chr12	74840280	74840500	Distal Intergenic
chr12	74840554	74841210	Distal Intergenic
chr12	74847261	74847460	Distal Intergenic
chr12	74855066	74856733	Distal Intergenic
chr12	74856847	74857920	Distal Intergenic
chr12	74865778	74866052	Distal Intergenic
chr12	74879345	74880990	Distal Intergenic
chr12	74891290	74892395	Distal Intergenic
chr12	74900234	74901169	Distal Intergenic
chr12	74907812	74908011	Distal Intergenic
chr12	74908239	74909735	Distal Intergenic
chr12	74911802	74912004	Distal Intergenic
chr12	74915591	74916635	Distal Intergenic
chr12	74939254	74939597	Distal Intergenic
chr12	74943422	74943621	Distal Intergenic
chr12	74981326	74981791	Distal Intergenic
chr12	74995890	74997832	Distal Intergenic
chr12	74997893	74998191	Distal Intergenic
chr12	75008311	75008663	Distal Intergenic
chr12	75009007	75009276	Distal Intergenic
chr12	75009706	75011287	Distal Intergenic
chr12	75011476	75012158	Distal Intergenic
chr12	75194016	75194698	Distal Intergenic
chr12	75194846	75196201	Distal Intergenic
chr12	75625083	75625697	Distal Intergenic
chr12	75925522	75926420	Distal Intergenic

chr12	75926604	75926817	Distal Intergenic
chr12	76629758	76630387	Distal Intergenic
chr12	76644876	76647867	Distal Intergenic
chr12	76655952	76656631	Distal Intergenic
chr12	76656669	76657025	Distal Intergenic
chr12	76658791	76659655	Distal Intergenic
chr12	76664236	76665435	Distal Intergenic
chr12	76665531	76665809	Distal Intergenic
chr12	76697475	76697770	Distal Intergenic
chr12	76770748	76773201	Exon
chr12	76781259	76782778	Intron
chr12	76803131	76804598	Exon
chr12	76927490	76929491	5' UTR
chr12	76967839	76968426	Distal Intergenic
chr12	76980812	76981067	Distal Intergenic
chr12	77115619	77115818	Distal Intergenic
chr12	77116551	77116906	Distal Intergenic
chr12	77143623	77144303	Distal Intergenic
chr12	77187108	77188193	Intron
chr12	77356860	77357173	Distal Intergenic
chr12	77471185	77471653	Distal Intergenic
chr12	77559834	77560205	Distal Intergenic
chr12	77560247	77560631	Distal Intergenic
chr12	77580862	77581414	Distal Intergenic
chr12	77604171	77604838	Distal Intergenic
chr12	77631934	77632326	Distal Intergenic
chr12	77632711	77632984	Distal Intergenic
chr12	77633227	77637208	Distal Intergenic
chr12	77637280	77638869	Distal Intergenic
chr12	77641421	77642501	Distal Intergenic
chr12	77648631	77648844	Distal Intergenic
chr12	77648946	77649178	Distal Intergenic
chr12	77652984	77653951	Distal Intergenic
chr12	77656001	77656403	Distal Intergenic
chr12	77657168	77657382	Distal Intergenic
chr12	77658398	77659955	Distal Intergenic
chr12	77694744	77695177	Distal Intergenic
chr12	77695229	77695467	Distal Intergenic
chr12	77895310	77895771	Distal Intergenic
chr12	77895831	77896980	Distal Intergenic
chr12	77910605	77910865	Distal Intergenic
chr12	77919103	77919460	Distal Intergenic
chr12	78120889	78121145	Distal Intergenic
chr12	78186213	78186424	Distal Intergenic

chr12	79071265	79071503	Distal Intergenic
chr12	79741375	79741848	Intron
chr12	79742054	79742297	Intron
chr12	79868959	79869296	Distal Intergenic
chr12	79869346	79869904	Distal Intergenic
chr12	79870136	79870563	Distal Intergenic
chr12	79942532	79942749	Distal Intergenic
chr12	79943207	79944194	Distal Intergenic
chr12	79961927	79962470	Distal Intergenic
chr12	79962736	79963169	Distal Intergenic
chr12	80008886	80009128	Intron
chr12	80011391	80013015	Intron
chr12	80053003	80053884	Intron
chr12	80148588	80148800	Distal Intergenic
chr12	80269007	80270098	Intron
chr12	80272907	80273114	Intron
chr12	80291702	80291910	Intron
chr12	80292476	80292853	Intron
chr12	80330761	80331170	Distal Intergenic
chr12	80348695	80350231	Distal Intergenic
chr12	80355191	80356292	Distal Intergenic
chr12	80431499	80431783	Distal Intergenic
chr12	80448865	80450307	Distal Intergenic
chr12	80452489	80453800	Distal Intergenic
chr12	80453840	80454272	Distal Intergenic
chr12	80483973	80485307	Distal Intergenic
chr12	80521464	80522507	Distal Intergenic
chr12	80534028	80536744	Distal Intergenic
chr12	80566692	80567938	Distal Intergenic
chr12	80572152	80573673	Distal Intergenic
chr12	80589820	80590308	Distal Intergenic
chr12	80596204	80596508	Distal Intergenic
chr12	80599007	80600038	Distal Intergenic
chr12	80602499	80603694	Promoter
chr12	80603796	80606101	Promoter
chr12	80606672	80606929	Intron
chr12	80607035	80609292	Intron
chr12	80615288	80616329	Exon
chr12	80624565	80624978	Intron
chr12	80625262	80627823	Exon
chr12	80628167	80628604	Intron
chr12	80646517	80647409	Exon
chr12	80652252	80654499	Intron
chr12	80654566	80654850	Intron

chr12	80656041	80656688	Intron
chr12	80660330	80660730	Exon
chr12	80660819	80661031	Intron
chr12	80684218	80685102	Intron
chr12	80698763	80699942	Exon
chr12	80699980	80700856	Intron
chr12	80701021	80702999	Intron
chr12	80703340	80703623	Intron
chr12	80711717	80713140	Exon
chr12	80749812	80751014	Promoter
chr12	80965397	80966461	Intron
chr12	81091304	81092373	Distal Intergenic
chr12	81135874	81136639	Distal Intergenic
chr12	81198781	81199258	Intron
chr12	81199400	81199600	Intron
chr12	81205026	81205307	Exon
chr12	81212963	81213524	Intron
chr12	81214630	81214851	Intron
chr12	81215169	81215796	Intron
chr12	81216069	81216784	Intron
chr12	81216828	81217213	Intron
chr12	81217331	81217730	Intron
chr12	81321003	81321922	Intron
chr12	81324134	81325225	Intron
chr12	81325322	81325589	Intron
chr12	81325652	81325989	Intron
chr12	81329066	81337689	Promoter
chr12	81341303	81341786	Distal Intergenic
chr12	81344817	81346489	Distal Intergenic
chr12	81352149	81352856	Distal Intergenic
chr12	81356736	81357115	Distal Intergenic
chr12	81357459	81359342	Distal Intergenic
chr12	81360109	81360833	Distal Intergenic
chr12	81361533	81362188	Distal Intergenic
chr12	81444971	81448162	Distal Intergenic
chr12	81459523	81459922	Distal Intergenic
chr12	81466731	81467701	Distal Intergenic
chr12	81468960	81469612	Distal Intergenic
chr12	81475006	81476464	Intron
chr12	81535875	81536668	Intron
chr12	81744667	81745289	Intron
chr12	81891599	81891827	Intron
chr12	81892009	81892645	Intron
chr12	81892718	81893539	Intron

chr12	81944532	81944849	Intron
chr12	81945000	81946031	Intron
chr12	81954251	81955603	Intron
chr12	82037970	82039228	Intron
chr12	82046509	82046715	Intron
chr12	82257294	82257596	Distal Intergenic
chr12	82257683	82258117	Distal Intergenic
chr12	82302338	82302537	Distal Intergenic
chr12	82336643	82336884	Distal Intergenic
chr12	82585699	82586107	Distal Intergenic
chr12	82633679	82633887	Distal Intergenic
chr12	82798607	82800367	Intron
chr12	82824152	82824918	Exon
chr12	82824960	82825160	Intron
chr12	82848744	82851747	Exon
chr12	82863436	82863984	Intron
chr12	82880619	82881084	Distal Intergenic
chr12	82881246	82881519	Distal Intergenic
chr12	82882334	82882868	Distal Intergenic
chr12	82941844	82944084	Distal Intergenic
chr12	82944163	82944983	Distal Intergenic
chr12	83072652	83073164	Distal Intergenic
chr12	83255257	83256335	Intron
chr12	84866621	84867943	Distal Intergenic
chr12	84972829	84973558	Distal Intergenic
chr12	84973663	84974575	Distal Intergenic
chr12	85028791	85029367	Distal Intergenic
chr12	85029434	85030292	Distal Intergenic
chr12	85062443	85062691	Distal Intergenic
chr12	85175989	85176483	Distal Intergenic
chr12	85215453	85215810	Distal Intergenic
chr12	85515757	85516012	Intron
chr12	85516507	85517237	Intron
chr12	85517641	85518247	Exon
chr12	85518787	85519103	Intron
chr12	85519274	85519591	Intron
chr12	85521580	85521878	Exon
chr12	85526473	85527158	Intron
chr12	85533520	85533722	Intron
chr12	85535456	85535868	Intron
chr12	85542679	85545232	Intron
chr12	85557901	85559619	Intron
chr12	85559854	85560465	Intron
chr12	85563281	85563795	Intron

chr12	85564086	85564323	Intron
chr12	85573657	85575270	Intron
chr12	85576528	85577927	Intron
chr12	85725523	85726469	Distal Intergenic
chr12	85736054	85736361	Distal Intergenic
chr12	85736529	85736738	Distal Intergenic
chr12	85955533	85956266	Distal Intergenic
chr12	85972853	85973215	Distal Intergenic
chr12	86108478	86108752	Distal Intergenic
chr12	86187660	86187913	Distal Intergenic
chr12	86208348	86208572	Intron
chr12	86391939	86392250	Intron
chr12	86574284	86574494	Intron
chr12	86679278	86679839	Intron
chr12	86702387	86702643	Intron
chr12	86703310	86704068	Intron
chr12	86714600	86716374	Intron
chr12	86758761	86759477	Intron
chr12	86759842	86760055	Intron
chr12	86761705	86761930	Intron
chr12	86792529	86793504	Intron
chr12	86910538	86910737	Intron
chr12	86910842	86911411	Intron
chr12	87165122	87165325	Intron
chr12	87165692	87165909	Intron
chr12	87210509	87210742	Intron
chr12	87210816	87211188	Intron
chr12	87300109	87301866	Distal Intergenic
chr12	87302270	87302480	Distal Intergenic
chr12	87302530	87302762	Distal Intergenic
chr12	87310360	87311578	Distal Intergenic
chr12	87312556	87313806	Distal Intergenic
chr12	87314209	87314421	Distal Intergenic
chr12	87341165	87341455	Distal Intergenic
chr12	87341574	87341831	Distal Intergenic
chr12	87352201	87353518	Distal Intergenic
chr12	87423759	87424517	Distal Intergenic
chr12	87424585	87427254	Distal Intergenic
chr12	87483016	87483908	Distal Intergenic
chr12	87582302	87582779	Distal Intergenic
chr12	88116548	88117294	Distal Intergenic
chr12	88128055	88128636	Distal Intergenic
chr12	88136375	88136936	Distal Intergenic
chr12	88137307	88137562	Distal Intergenic

chr12	88139848	88140917	Distal Intergenic
chr12	88140986	88143251	Distal Intergenic
chr12	88143331	88143627	Distal Intergenic
chr12	88164780	88166040	Distal Intergenic
chr12	88166218	88166685	Distal Intergenic
chr12	88172811	88176041	Downstream
chr12	88177411	88177799	Promoter
chr12	88208273	88209261	Distal Intergenic
chr12	88213297	88213566	Distal Intergenic
chr12	88224941	88225222	Distal Intergenic
chr12	88230053	88230336	Distal Intergenic
chr12	88488710	88488984	Intron
chr12	88607368	88607595	Distal Intergenic
chr12	88607928	88608199	Distal Intergenic
chr12	88775353	88776928	Distal Intergenic
chr12	88783889	88784089	Distal Intergenic
chr12	88784445	88784936	Distal Intergenic
chr12	88812456	88812662	Distal Intergenic
chr12	88812808	88813134	Distal Intergenic
chr12	89108904	89109561	Distal Intergenic
chr12	89453124	89453535	Distal Intergenic
chr12	89453595	89455215	Distal Intergenic
chr12	89515962	89516578	Distal Intergenic
chr12	92020330	92020548	Distal Intergenic
chr12	92143322	92143561	Distal Intergenic
chr12	92159460	92159896	Distal Intergenic
chr12	92159977	92160176	Distal Intergenic
chr12	92826703	92827414	Intron
chr12	92828092	92828413	Intron
chr12	92924260	92924554	Distal Intergenic
chr12	92924686	92925191	Distal Intergenic
chr12	92931529	92932474	Distal Intergenic
chr12	92984036	92984765	Distal Intergenic
chr12	92986329	92986748	Distal Intergenic
chr12	93013161	93013974	Distal Intergenic
chr12	96614516	96615889	Intron
chr12	96685547	96686209	Intron
chr12	96707965	96708298	Intron
chr12	96729251	96731066	Intron
chr12	96776791	96777118	Intron
chr12	96798745	96800201	Distal Intergenic
chr12	96801762	96802123	Distal Intergenic
chr12	96837244	96839504	Distal Intergenic
chr12	96930810	96931015	Intron

chr12	96931231	96933241	Exon
chr12	96933535	96933789	Intron
chr12	99520668	99522279	Intron
chr12	99951791	99952126	Intron
chr12	99975150	99975433	Intron
chr12	100232657	100233193	Intron
chr12	100233229	100233465	Intron
chr12	100233638	100233967	Intron
chr12	100234069	100235088	Intron
chr12	100262789	100263005	Intron
chr12	100302321	100303011	Intron
chr12	100568806	100569005	Distal Intergenic
chr12	100573885	100576200	Distal Intergenic
chr12	100594554	100595963	Promoter
chr12	100599235	100599536	5' UTR
chr12	100599665	100599936	Intron
chr12	100615059	100616309	Exon
chr12	101217777	101218725	Intron
chr12	101448988	101449330	Intron
chr12	101518357	101518649	Intron
chr12	101759784	101760096	Intron
chr12	101943117	101943387	Distal Intergenic
chr12	101994898	101995097	Intron
chr12	102016551	102016911	Intron
chr12	102017322	102017803	Intron
chr12	102219191	102220400	Intron
chr12	102240837	102241739	Distal Intergenic
chr12	102321268	102321653	Distal Intergenic
chr12	103151156	103151594	Distal Intergenic
chr12	103161416	103162419	Distal Intergenic
chr12	103163441	103163789	Distal Intergenic
chr12	103213515	103214995	Exon
chr12	103963621	103964009	Distal Intergenic
chr12	103964214	103964645	Distal Intergenic
chr12	103982004	103982635	Promoter
chr12	104115447	104115860	Intron
chr12	104519418	104519674	Intron
chr12	104519832	104520366	Exon
chr12	104618382	104618710	Exon
chr12	104618787	104619244	Intron
chr12	104803131	104803508	Distal Intergenic
chr12	105241032	105241547	Intron
chr12	105302771	105303659	Exon
chr12	105361483	105361829	Distal Intergenic

chr12	105362126	105362401	Distal Intergenic
chr12	105362531	105363213	Distal Intergenic
chr12	105433437	105433636	Exon
chr12	105450215	105450416	Intron
chr12	105485170	105485893	Distal Intergenic
chr12	105485993	105486353	Distal Intergenic
chr12	105500390	105501601	Promoter
chr12	105509285	105509870	5' UTR
chr12	105654258	105654621	Distal Intergenic
chr12	106237108	106237560	Distal Intergenic
chr12	106811245	106813149	Intron
chr12	107022963	107023176	Intron
chr12	107081353	107082527	Intron
chr12	107806588	107806908	Intron
chr12	107806998	107807458	Intron
chr12	108001604	108001817	Intron
chr12	108001879	108002787	Intron
chr12	108003140	108003392	Intron
chr12	108117312	108117767	Distal Intergenic
chr12	108118250	108118469	Distal Intergenic
chr12	108855020	108855607	Distal Intergenic
chr12	108883827	108884093	Distal Intergenic
chr12	108990717	108990994	Promoter
chr12	108992893	108994843	Promoter
chr12	109045454	109046616	Exon
chr12	109094278	109094792	Intron
chr12	109095095	109095632	5' UTR
chr12	109189447	109189921	Intron
chr12	109691954	109692594	Exon
chr12	109917576	109917775	Intron
chr12	109987053	109989409	Downstream
chr12	110152166	110152691	Promoter
chr12	110152745	110153114	Promoter
chr12	110864902	110865245	Distal Intergenic
chr12	111724020	111724312	Intron
chr12	111749547	111749934	Exon
chr12	111750203	111751371	Intron
chr12	111751531	111751779	Intron
chr12	111770852	111772356	Exon
chr12	111790460	111792016	Distal Intergenic
chr12	111793634	111793925	Distal Intergenic
chr12	111794014	111794214	Distal Intergenic
chr12	111794271	111794919	Distal Intergenic
chr12	112036155	112036354	Promoter

chr12	112424990	112425375	Intron
chr12	112476539	112477088	Exon
chr12	112587659	112587954	Exon
chr12	112588065	112588303	Intron
chr12	112591203	112591696	3' UTR
chr12	112709589	112710271	Intron
chr12	112775748	112776109	Intron
chr12	112790912	112792142	Intron
chr12	112794811	112795236	Intron
chr12	112798092	112798404	Intron
chr12	112803686	112803889	Intron
chr12	112806749	112807708	Intron
chr12	112813409	112813608	Intron
chr12	112936191	112937044	Intron
chr12	112942820	112943888	3' UTR
chr12	112991277	112991916	Distal Intergenic
chr12	112996088	112996813	Distal Intergenic
chr12	112998308	112998936	Distal Intergenic
chr12	113001149	113001441	Distal Intergenic
chr12	113388168	113388455	Intron
chr12	113420135	113420415	Intron
chr12	113420754	113421030	Intron
chr12	113421186	113421440	Intron
chr12	113421612	113421861	Intron
chr12	113422513	113422802	Intron
chr12	113532741	113533250	Exon
chr12	113533482	113533796	Intron
chr12	113533962	113534266	Intron
chr12	113578033	113578656	Distal Intergenic
chr12	113816791	113817000	Intron
chr12	113817317	113817724	Intron
chr12	113817935	113818215	Intron
chr12	113832725	113834516	Intron
chr12	113854985	113855221	Distal Intergenic
chr12	113912616	113913017	Distal Intergenic
chr12	113913287	113913519	Distal Intergenic
chr12	114032212	114032800	Distal Intergenic
chr12	114032856	114034425	Distal Intergenic
chr12	114035399	114035788	Distal Intergenic
chr12	114036423	114036647	Distal Intergenic
chr12	114060556	114062980	Distal Intergenic
chr12	114066859	114067573	Distal Intergenic
chr12	114089347	114090730	Distal Intergenic
chr12	114114845	114115375	Distal Intergenic

chr12	114116100	114116512	Distal Intergenic
chr12	114116555	114116934	Distal Intergenic
chr12	114118829	114119181	Distal Intergenic
chr12	114119412	114119770	Distal Intergenic
chr12	114165789	114166070	Distal Intergenic
chr12	114178524	114180084	Distal Intergenic
chr12	114261191	114261390	Intron
chr12	114415330	114415559	Distal Intergenic
chr12	114416034	114416267	Distal Intergenic
chr12	117518933	117519300	Intron
chr12	117531904	117532833	Intron
chr12	117712615	117713747	Intron
chr12	118669007	118669990	Intron
chr12	118680614	118681211	Intron
chr12	118681870	118682100	Intron
chr12	118687098	118687618	Intron
chr12	118771627	118771851	Intron
chr12	120052834	120053245	Intron
chr12	120144933	120145188	Intron
chr12	120145229	120145603	Intron
chr12	120145702	120145959	Intron
chr12	120664455	120664731	Promoter
chr12	120679547	120679753	Intron
chr13	3094316	3094515	Distal Intergenic
chr13	3537412	3538395	Distal Intergenic
chr13	3609200	3609768	Distal Intergenic
chr13	3745611	3746237	Distal Intergenic
chr13	3778294	3778570	Distal Intergenic
chr13	3817602	3818371	Distal Intergenic
chr13	3820876	3821766	Distal Intergenic
chr13	3830953	3831232	Distal Intergenic
chr13	3831443	3831692	Distal Intergenic
chr13	3861734	3862586	Distal Intergenic
chr13	3864726	3865330	Distal Intergenic
chr13	3866216	3866714	Distal Intergenic
chr13	3876038	3877611	Distal Intergenic
chr13	3886101	3886747	Distal Intergenic
chr13	3891033	3891258	Distal Intergenic
chr13	3891529	3891762	Distal Intergenic
chr13	3891808	3892765	Distal Intergenic
chr13	5301638	5302116	Distal Intergenic
chr13	5314188	5314655	Distal Intergenic
chr13	5423275	5423659	Distal Intergenic
chr13	5443417	5445148	Distal Intergenic

chr13	5723129	5723410	Distal Intergenic
chr13	5723453	5723697	Distal Intergenic
chr13	5802476	5804008	Distal Intergenic
chr13	5824733	5826141	Distal Intergenic
chr13	5836288	5837082	Distal Intergenic
chr13	5839755	5841460	Distal Intergenic
chr13	5843145	5845231	Distal Intergenic
chr13	5858171	5859332	Distal Intergenic
chr13	5859418	5860474	Distal Intergenic
chr13	5860724	5863254	Distal Intergenic
chr13	5863339	5864454	Distal Intergenic
chr13	5864528	5866319	Distal Intergenic
chr13	5868541	5870201	Distal Intergenic
chr13	5870402	5871177	Distal Intergenic
chr13	5885665	5886793	Distal Intergenic
chr13	5891293	5893489	Distal Intergenic
chr13	5912233	5912659	Distal Intergenic
chr13	6164650	6165253	Distal Intergenic
chr13	6190935	6191189	Distal Intergenic
chr13	6191302	6193335	Distal Intergenic
chr13	6203922	6204121	Distal Intergenic
chr13	6612983	6614274	Distal Intergenic
chr13	6623601	6624036	Distal Intergenic
chr13	6628185	6628529	Distal Intergenic
chr13	6630788	6631137	Distal Intergenic
chr13	6646544	6646875	Distal Intergenic
chr13	6658419	6658623	Distal Intergenic
chr13	8828582	8828984	Distal Intergenic
chr13	8870244	8870726	Distal Intergenic
chr13	8883993	8885451	Distal Intergenic
chr13	8885517	8885841	Distal Intergenic
chr13	8885935	8886154	Distal Intergenic
chr13	8886253	8886472	Distal Intergenic
chr13	8994522	8995555	Distal Intergenic
chr13	9093775	9094187	Distal Intergenic
chr13	9700051	9700826	Distal Intergenic
chr13	9748828	9749470	Distal Intergenic
chr13	9762400	9763680	Distal Intergenic
chr13	9772285	9772990	Distal Intergenic
chr13	11641365	11642023	Distal Intergenic
chr13	11642392	11642616	Distal Intergenic
chr13	12384574	12385114	Distal Intergenic
chr13	12385261	12385830	Distal Intergenic
chr13	12385877	12386352	Distal Intergenic

chr13	12552672	12553546	Distal Intergenic
chr13	12577864	12578739	Distal Intergenic
chr13	12599299	12599667	Distal Intergenic
chr13	12612135	12612373	Distal Intergenic
chr13	12612605	12612824	Distal Intergenic
chr13	12795904	12796344	Distal Intergenic
chr13	12798563	12798823	Distal Intergenic
chr13	12892325	12892865	Distal Intergenic
chr13	12892932	12893546	Distal Intergenic
chr13	12894031	12895476	Distal Intergenic
chr13	12900507	12902760	Distal Intergenic
chr13	12904775	12907116	Distal Intergenic
chr13	12910119	12911410	Distal Intergenic
chr13	13097318	13098793	Distal Intergenic
chr13	13099602	13100548	Distal Intergenic
chr13	13106105	13106478	Distal Intergenic
chr13	13106639	13107095	Distal Intergenic
chr13	13116921	13118957	Distal Intergenic
chr13	13122466	13123217	Distal Intergenic
chr13	13166698	13167345	Distal Intergenic
chr13	13168425	13168974	Distal Intergenic
chr13	13255470	13256510	Distal Intergenic
chr13	13260018	13262619	Distal Intergenic
chr13	13265629	13266176	Distal Intergenic
chr13	13272340	13272890	Distal Intergenic
chr13	13273900	13274628	Distal Intergenic
chr13	13533514	13533713	Distal Intergenic
chr13	13665097	13665383	Distal Intergenic
chr13	13840540	13841006	Distal Intergenic
chr13	14060443	14061848	Distal Intergenic
chr13	14126577	14126832	Distal Intergenic
chr13	14131236	14131530	Distal Intergenic
chr13	14131624	14132911	Distal Intergenic
chr13	14152158	14152674	Distal Intergenic
chr13	14154670	14155415	Distal Intergenic
chr13	14155980	14156439	Distal Intergenic
chr13	14705491	14706253	Distal Intergenic
chr13	14722223	14722436	Distal Intergenic
chr13	14722621	14722954	Distal Intergenic
chr13	15263607	15263876	Distal Intergenic
chr13	15275812	15276864	Distal Intergenic
chr13	15513201	15514114	Distal Intergenic
chr13	15518587	15519299	Distal Intergenic
chr13	15602654	15602893	Distal Intergenic

chr13	15603391	15604364	Distal Intergenic
chr13	15604821	15606447	Distal Intergenic
chr13	15629514	15630098	Distal Intergenic
chr13	15752253	15752758	Distal Intergenic
chr13	15895759	15895958	Distal Intergenic
chr13	15896076	15896448	Distal Intergenic
chr13	15898214	15899671	Distal Intergenic
chr13	16557356	16558456	Distal Intergenic
chr13	16578534	16579238	Distal Intergenic
chr13	16589885	16590439	Distal Intergenic
chr13	16641846	16642581	Distal Intergenic
chr13	16733407	16734604	Distal Intergenic
chr13	16749305	16749736	Distal Intergenic
chr13	16750896	16752338	Distal Intergenic
chr13	16761224	16762780	Distal Intergenic
chr13	16827592	16828606	Distal Intergenic
chr13	16829151	16832251	Distal Intergenic
chr13	16852905	16853908	Distal Intergenic
chr13	17549298	17550497	Distal Intergenic
chr13	17584169	17587244	Distal Intergenic
chr13	17603118	17604007	Distal Intergenic
chr13	17695250	17697196	Distal Intergenic
chr13	17698310	17698663	Distal Intergenic
chr13	17728447	17728978	Distal Intergenic
chr13	17781602	17782046	Distal Intergenic
chr13	17785723	17786474	Distal Intergenic
chr13	17786915	17787192	Distal Intergenic
chr13	17787231	17787509	Distal Intergenic
chr13	17787610	17788079	Distal Intergenic
chr13	17797066	17797492	Distal Intergenic
chr13	17895032	17895697	Distal Intergenic
chr13	17896161	17896382	Distal Intergenic
chr13	17992265	17993899	Distal Intergenic
chr13	18035040	18035246	Distal Intergenic
chr13	18035294	18035768	Distal Intergenic
chr13	19486705	19487140	Intron
chr13	21271253	21272035	Distal Intergenic
chr13	21494437	21494648	Distal Intergenic
chr13	21507601	21507930	Distal Intergenic
chr13	21507993	21508207	Distal Intergenic
chr13	21508698	21509933	Distal Intergenic
chr13	21544840	21545292	Downstream
chr13	21598235	21599037	Intron
chr13	21599176	21600658	Intron

chr13	21600856	21601056	Intron
chr13	21807710	21808119	Distal Intergenic
chr13	21808276	21808680	Distal Intergenic
chr13	21808933	21809416	Distal Intergenic
chr13	21809537	21811050	Distal Intergenic
chr13	21812967	21814593	Distal Intergenic
chr13	21826246	21826821	Distal Intergenic
chr13	21827041	21827642	Distal Intergenic
chr13	21841008	21841599	Distal Intergenic
chr13	21841688	21842909	Distal Intergenic
chr13	21843965	21844822	Distal Intergenic
chr13	21845715	21847234	Distal Intergenic
chr13	21870303	21872758	Promoter
chr13	21873784	21875450	Intron
chr13	21877523	21879266	Intron
chr13	21879338	21880136	Intron
chr13	21900513	21902191	Intron
chr13	21902501	21904126	Intron
chr13	21921077	21921276	Intron
chr13	21922719	21923120	Intron
chr13	21923330	21925395	Exon
chr13	21925533	21927086	Intron
chr13	22125564	22126048	Intron
chr13	22126677	22126926	Intron
chr13	22127027	22127383	Intron
chr13	22127940	22128749	Intron
chr13	22131580	22134682	Intron
chr13	22135505	22135756	Intron
chr13	22135794	22136161	Intron
chr13	23460515	23461076	Distal Intergenic
chr13	23536694	23537395	Distal Intergenic
chr13	23537453	23537751	Distal Intergenic
chr13	23597929	23598141	Distal Intergenic
chr13	23622324	23622818	Distal Intergenic
chr13	23623016	23625438	Distal Intergenic
chr13	23625895	23628523	Distal Intergenic
chr13	23633921	23634121	Distal Intergenic
chr13	23634202	23636051	Distal Intergenic
chr13	23636307	23637835	Distal Intergenic
chr13	23641587	23641822	Distal Intergenic
chr13	23642359	23644173	Distal Intergenic
chr13	23646602	23649121	Distal Intergenic
chr13	23652773	23654221	Distal Intergenic
chr13	23654893	23655211	Distal Intergenic

chr13	23655390	23655648	Distal Intergenic
chr13	23661472	23661687	Distal Intergenic
chr13	23661992	23663040	Distal Intergenic
chr13	23663148	23666333	Distal Intergenic
chr13	23666386	23667022	Distal Intergenic
chr13	23667134	23668628	Distal Intergenic
chr13	23668676	23669185	Distal Intergenic
chr13	23672960	23675383	Distal Intergenic
chr13	23675566	23678082	Distal Intergenic
chr13	23678300	23678660	Distal Intergenic
chr13	23712622	23713976	Distal Intergenic
chr13	23751240	23751505	Distal Intergenic
chr13	23751757	23752375	Distal Intergenic
chr13	23774145	23774723	Intron
chr13	23774815	23775507	Intron
chr13	23775568	23775884	Intron
chr13	23775927	23777720	Intron
chr13	23789483	23790227	Intron
chr13	23830191	23832706	Intron
chr13	23835952	23838494	Intron
chr13	23838647	23843037	Intron
chr13	23843173	23845736	Intron
chr13	23845778	23845991	Intron
chr13	23848348	23848734	Intron
chr13	23848803	23850101	Intron
chr13	23852076	23853029	Intron
chr13	24188251	24188558	Intron
chr13	24195569	24196955	Intron
chr13	24197060	24198013	Intron
chr13	24466650	24468286	Exon
chr13	24489016	24489617	Intron
chr13	24612871	24613083	Intron
chr13	24613171	24613801	Intron
chr13	24613841	24614421	Intron
chr13	24628913	24629303	Intron
chr13	24656622	24658692	Intron
chr13	24766007	24766501	Intron
chr13	24770753	24771856	Intron
chr13	24892741	24893702	Exon
chr13	24893756	24894002	Intron
chr13	24894046	24894367	Intron
chr13	25163028	25163232	Intron
chr13	25840662	25840932	Intron
chr13	27032094	27032448	Distal Intergenic

chr13	27095812	27096534	Distal Intergenic
chr13	27119075	27119422	Distal Intergenic
chr13	27204654	27206261	Intron
chr13	27240429	27241141	Intron
chr13	27241376	27241604	Intron
chr13	27674210	27674523	Intron
chr13	27677911	27678330	Intron
chr13	27678456	27678726	Intron
chr13	27752133	27752774	Distal Intergenic
chr13	27810804	27811527	Distal Intergenic
chr13	27816617	27817906	Distal Intergenic
chr13	27820867	27823937	Distal Intergenic
chr13	27825434	27827470	Promoter
chr13	27890644	27891605	Distal Intergenic
chr13	27891643	27892020	Distal Intergenic
chr13	28548787	28550366	Downstream
chr13	28721183	28721941	Intron
chr13	28810887	28811307	Intron
chr13	28829546	28830105	Intron
chr13	28976067	28976385	Intron
chr13	29008877	29010284	Intron
chr13	29042129	29042707	Intron
chr13	29042757	29043488	Intron
chr13	29043772	29045318	Intron
chr13	29593172	29596226	Distal Intergenic
chr13	29596416	29597126	Distal Intergenic
chr13	29719287	29720672	Intron
chr13	30075373	30075741	Intron
chr13	30076650	30076849	Intron
chr13	30076892	30077541	3' UTR
chr13	30200362	30200572	Distal Intergenic
chr13	30200979	30201596	Distal Intergenic
chr13	30241007	30241496	Distal Intergenic
chr13	30253554	30253939	Distal Intergenic
chr13	30285744	30286093	Distal Intergenic
chr13	30286203	30287167	Distal Intergenic
chr13	31023690	31024183	Distal Intergenic
chr13	31064574	31064773	Intron
chr13	31064944	31065781	Intron
chr13	31343400	31344413	Distal Intergenic
chr13	31419498	31419915	Distal Intergenic
chr13	31422668	31423777	Distal Intergenic
chr13	31423971	31424603	Distal Intergenic
chr13	31426526	31427162	Distal Intergenic

chr13	31433332	31433556	Distal Intergenic
chr13	31497357	31497636	Intron
chr13	31499951	31500546	Intron
chr13	31500747	31501024	Intron
chr13	31659147	31660043	Distal Intergenic
chr13	31669309	31669709	Distal Intergenic
chr13	31669926	31670487	Distal Intergenic
chr13	31899181	31899567	Intron
chr13	31900661	31901215	Intron
chr13	31901316	31901984	Intron
chr13	31902196	31902395	Intron
chr13	31955195	31956420	Distal Intergenic
chr13	31968707	31968948	Distal Intergenic
chr13	32226335	32227369	Distal Intergenic
chr13	32570602	32571597	Distal Intergenic
chr13	32683526	32684311	Intron
chr13	32752127	32752396	5' UTR
chr13	32752498	32752697	5' UTR
chr13	32924067	32925210	Intron
chr13	32937895	32938379	Intron
chr13	32976462	32976802	3' UTR
chr13	32995361	32995711	Intron
chr13	33004498	33005465	Downstream
chr13	33005507	33005709	Downstream
chr13	33015898	33016133	Intron
chr13	33016284	33017683	Exon
chr13	33018030	33020823	5' UTR
chr13	33030872	33031883	Intron
chr13	33055586	33057074	Promoter
chr13	33057112	33057546	Intron
chr13	33095250	33097290	5' UTR
chr13	33118476	33118944	Distal Intergenic
chr13	33119174	33119446	Distal Intergenic
chr13	33119515	33119757	Distal Intergenic
chr13	33119824	33121624	Distal Intergenic
chr13	33142488	33143220	Distal Intergenic
chr13	33143757	33144083	Distal Intergenic
chr13	33158620	33158936	Distal Intergenic
chr13	33158994	33160190	Promoter
chr13	33169767	33170004	Intron
chr13	33170055	33171677	Intron
chr13	33181354	33181666	Intron
chr13	33181813	33182872	Intron
chr13	33182932	33183441	Intron

chr13	34026725	34027409	Intron
chr13	34027474	34027824	Intron
chr13	34027959	34028365	Intron
chr13	34094206	34094459	Intron
chr13	34171946	34172325	Intron
chr13	34187942	34189032	Intron
chr13	34201378	34202206	Intron
chr13	34203590	34203801	Intron
chr13	34203890	34205813	Intron
chr13	34221750	34222354	Intron
chr13	34254345	34254844	Distal Intergenic
chr13	34281309	34284829	Distal Intergenic
chr13	34284896	34285126	Distal Intergenic
chr13	34285203	34285408	Distal Intergenic
chr13	34301557	34302585	Distal Intergenic
chr13	34304042	34304888	Distal Intergenic
chr13	34304994	34305454	Distal Intergenic
chr13	34418606	34419031	Intron
chr13	34424344	34424774	Intron
chr13	34424832	34425088	Intron
chr13	34435068	34435336	Intron
chr13	34435387	34435726	Intron
chr13	34461648	34462365	Intron
chr13	34462538	34464423	Intron
chr13	34489935	34492481	Intron
chr13	34493690	34495105	Intron
chr13	34632236	34632488	Distal Intergenic
chr13	34742699	34742909	Distal Intergenic
chr13	34743059	34743935	Distal Intergenic
chr13	34744339	34744639	Distal Intergenic
chr13	34744860	34745333	Distal Intergenic
chr13	34757784	34758320	Distal Intergenic
chr13	34767871	34769695	Distal Intergenic
chr13	34774830	34775033	Distal Intergenic
chr13	34775342	34776636	Distal Intergenic
chr13	34781591	34782543	Distal Intergenic
chr13	34788653	34789552	Distal Intergenic
chr13	34807033	34807550	Distal Intergenic
chr13	34807618	34807979	Distal Intergenic
chr13	34818109	34818367	Distal Intergenic
chr13	34966842	34967614	Distal Intergenic
chr13	34967697	34968042	Distal Intergenic
chr13	35375677	35376056	Distal Intergenic
chr13	35376101	35376563	Distal Intergenic

chr13	35604223	35604689	Intron
chr13	35925869	35926279	Intron
chr13	36625943	36626900	Intron
chr13	36702421	36702885	Intron
chr13	37382419	37383177	Distal Intergenic
chr13	37545070	37546817	Exon
chr13	37875275	37877026	Distal Intergenic
chr13	37919355	37919681	Distal Intergenic
chr13	37920093	37920297	Distal Intergenic
chr13	37922577	37922933	Distal Intergenic
chr13	37938597	37939897	Distal Intergenic
chr13	37957979	37959033	Distal Intergenic
chr13	37959091	37959665	Distal Intergenic
chr13	38000104	38001563	Distal Intergenic
chr13	38001675	38002005	Distal Intergenic
chr13	38002092	38003304	Distal Intergenic
chr13	38015907	38016226	Distal Intergenic
chr13	38162283	38162920	Intron
chr13	38163066	38163361	Intron
chr13	38171582	38174292	Promoter
chr13	38174367	38175700	Intron
chr13	38187790	38189446	Distal Intergenic
chr13	38190294	38191156	Distal Intergenic
chr13	38210319	38210912	3' UTR
chr13	38214955	38215183	Intron
chr13	38215268	38216460	Intron
chr13	38218879	38219899	Intron
chr13	38236600	38237873	Exon
chr13	38242251	38245744	Intron
chr13	38245785	38255851	5' UTR
chr13	38296827	38297384	Intron
chr13	38302229	38303451	Intron
chr13	38303488	38303703	Intron
chr13	38441202	38442897	Intron
chr13	38806743	38806972	Distal Intergenic
chr13	38807008	38807392	Distal Intergenic
chr13	38868141	38868779	Distal Intergenic
chr13	38885065	38885313	Distal Intergenic
chr13	38885351	38885801	Distal Intergenic
chr13	38907215	38907860	Distal Intergenic
chr13	38907898	38908128	Distal Intergenic
chr13	38908216	38908422	Distal Intergenic
chr13	39703314	39703834	Distal Intergenic
chr13	39999331	39999574	Intron

chr13	40018454	40019083	Intron
chr13	40019497	40020810	Intron
chr13	40023974	40024792	Intron
chr13	40779367	40779660	Intron
chr13	40813810	40815737	Distal Intergenic
chr13	40818822	40820115	Distal Intergenic
chr13	40820169	40820370	Distal Intergenic
chr13	40820465	40821118	Distal Intergenic
chr13	40821430	40822015	Distal Intergenic
chr13	40822149	40826247	Distal Intergenic
chr13	40827023	40828957	Distal Intergenic
chr13	40944295	40944757	Intron
chr13	41011979	41012966	Intron
chr13	41078347	41078589	Intron
chr13	41081212	41081946	Intron
chr13	41096136	41096827	Intron
chr13	41111671	41112035	Intron
chr13	41419367	41419797	Intron
chr13	41419852	41420456	Intron
chr13	41426253	41429606	Exon
chr13	41430163	41430711	Intron
chr13	41434317	41434807	Intron
chr13	41443674	41443899	Intron
chr13	41444784	41445078	Intron
chr13	41445445	41445817	Intron
chr13	41571802	41572469	Intron
chr13	42328677	42329857	Intron
chr13	42423947	42424316	Intron
chr13	42573152	42574220	Distal Intergenic
chr13	42650102	42650316	Intron
chr13	42650525	42650773	Intron
chr13	42650833	42651114	Intron
chr13	42653278	42654661	Intron
chr13	43243867	43245190	Distal Intergenic
chr13	43249946	43250152	Distal Intergenic
chr13	43268159	43268495	Distal Intergenic
chr13	43288774	43289231	Distal Intergenic
chr13	43330556	43330777	Distal Intergenic
chr13	43388263	43388674	Distal Intergenic
chr13	43574453	43574749	Distal Intergenic
chr13	44182684	44182897	Intron
chr13	44628373	44628607	Distal Intergenic
chr13	44726607	44727494	Intron
chr13	44755134	44755821	Distal Intergenic

chr13	44755976	44756232	Distal Intergenic
chr13	44798659	44799690	Distal Intergenic
chr13	44826174	44826556	Distal Intergenic
chr13	44827585	44827875	Distal Intergenic
chr13	44828117	44828571	Distal Intergenic
chr13	44829066	44829704	Distal Intergenic
chr13	44914667	44915145	Distal Intergenic
chr13	44941237	44941473	Distal Intergenic
chr13	45034324	45036467	Intron
chr13	45064446	45064657	Intron
chr13	45370777	45371169	Downstream
chr13	45371636	45371893	Downstream
chr13	45373153	45373768	Exon
chr13	45401425	45401681	Distal Intergenic
chr13	45401789	45402224	Distal Intergenic
chr13	45497200	45498865	Distal Intergenic
chr13	45665084	45666052	Distal Intergenic
chr13	45752861	45754310	Intron
chr13	45779680	45781294	Intron
chr13	45781387	45781774	Exon
chr13	45790269	45790972	Intron
chr13	45814511	45814710	Intron
chr13	45814779	45815088	Intron
chr13	45846568	45847046	Intron
chr13	45897549	45899285	Distal Intergenic
chr13	45923128	45923407	Intron
chr13	45992564	45994432	Promoter
chr13	46001833	46002236	Distal Intergenic
chr13	46002358	46003652	Distal Intergenic
chr13	46006922	46007583	Distal Intergenic
chr13	46017576	46019126	Distal Intergenic
chr13	46019170	46020992	Distal Intergenic
chr13	46026624	46027035	Distal Intergenic
chr13	46060832	46061727	Intron
chr13	46062055	46062330	Intron
chr13	46137903	46138179	Exon
chr13	46138248	46141049	Intron
chr13	46142354	46142759	Exon
chr13	46203179	46204595	Distal Intergenic
chr13	46204727	46205288	Distal Intergenic
chr13	46322628	46323343	Distal Intergenic
chr13	46377123	46378693	Intron
chr13	46379140	46379438	Intron
chr13	46379537	46381080	Intron

chr13	46811669	46812211	Intron
chr13	46820732	46821199	Exon
chr13	46821248	46823359	Intron
chr13	46935015	46938821	Exon
chr13	47023466	47024661	Distal Intergenic
chr13	47025341	47026478	Distal Intergenic
chr13	47050116	47052111	Distal Intergenic
chr13	47054127	47055471	Distal Intergenic
chr13	47059658	47064310	Distal Intergenic
chr13	47263411	47267179	Exon
chr13	47275463	47275903	Intron
chr13	47289883	47290523	Intron
chr13	47311685	47312228	Intron
chr13	47312379	47312994	Intron
chr13	47340038	47340400	Distal Intergenic
chr13	47340470	47340778	Distal Intergenic
chr13	47368275	47368474	Intron
chr13	47376487	47376962	Distal Intergenic
chr13	47489729	47490485	Distal Intergenic
chr13	47581910	47582109	Distal Intergenic
chr13	48112281	48112527	Distal Intergenic
chr13	48205192	48205532	Distal Intergenic
chr13	48205651	48206492	Distal Intergenic
chr13	48611045	48611366	Promoter
chr13	48661123	48663826	Intron
chr13	48666628	48666980	Intron
chr13	48710141	48710374	Distal Intergenic
chr13	48965007	48965450	Intron
chr13	49028234	49028468	Intron
chr13	49028529	49028885	Intron
chr13	49054953	49055326	3' UTR
chr13	49055578	49058883	3' UTR
chr13	49061097	49062987	Downstream
chr13	49063830	49064206	3' UTR
chr13	49279146	49279756	Intron
chr13	49279794	49280003	Promoter
chr13	49282214	49282657	3' UTR
chr13	49389323	49389604	Distal Intergenic
chr13	49389654	49390031	Distal Intergenic
chr13	49393665	49395257	Distal Intergenic
chr13	49395312	49395521	Distal Intergenic
chr13	49395867	49396469	Distal Intergenic
chr13	49402736	49403077	Distal Intergenic
chr13	49403322	49403537	Distal Intergenic

chr13	49434513	49435013	Distal Intergenic
chr13	49437752	49438492	Distal Intergenic
chr13	49441219	49441515	Distal Intergenic
chr13	49457354	49458579	Distal Intergenic
chr13	49459398	49460975	Distal Intergenic
chr13	49461037	49461495	Distal Intergenic
chr13	49461875	49464212	Distal Intergenic
chr13	49466756	49467034	Distal Intergenic
chr13	49467078	49467668	Distal Intergenic
chr13	49467869	49468449	Distal Intergenic
chr13	49491760	49492058	Distal Intergenic
chr13	49492175	49492544	Distal Intergenic
chr13	49497743	49498142	Distal Intergenic
chr13	49645210	49645499	Intron
chr13	49679130	49679869	Intron
chr13	49748481	49748869	Exon
chr13	49777905	49778105	Intron
chr13	50538142	50538468	Distal Intergenic
chr13	51052687	51054919	Intron
chr13	51055787	51056756	Intron
chr13	51106109	51106590	Intron
chr13	51106658	51106964	Intron
chr13	51107044	51107270	Intron
chr13	51109969	51110230	Intron
chr13	51134368	51134587	Intron
chr13	51134625	51135340	Intron
chr13	51135896	51138002	Intron
chr13	51157632	51159026	Intron
chr13	51185167	51185652	Intron
chr13	51185734	51186084	Intron
chr13	51196835	51197681	Intron
chr13	51208077	51212094	Distal Intergenic
chr13	51223310	51223560	Distal Intergenic
chr13	51396531	51397627	3' UTR
chr13	51423902	51424917	Exon
chr13	51427973	51429593	Distal Intergenic
chr13	51495115	51495594	Intron
chr13	51495670	51496345	Intron
chr13	51740667	51741091	Intron
chr13	51741229	51741653	Intron
chr13	51746616	51746826	Promoter
chr13	51770653	51771256	Distal Intergenic
chr13	51779988	51781417	Distal Intergenic
chr13	51834065	51834275	Intron

chr13	51851645	51851892	Intron
chr13	51857771	51858097	3' UTR
chr13	51858135	51859245	3' UTR
chr13	51943364	51943590	Exon
chr13	52060070	52060276	Distal Intergenic
chr13	52064476	52064839	Distal Intergenic
chr13	52070963	52071527	Distal Intergenic
chr13	52284926	52285749	Intron
chr13	52331198	52331456	Intron
chr13	52337149	52337902	Distal Intergenic
chr13	52401834	52403047	Intron
chr13	52550140	52551134	Intron
chr13	52573411	52574053	Intron
chr13	52577114	52577323	Intron
chr13	52577381	52577729	Intron
chr13	52621646	52622024	Distal Intergenic
chr13	52636747	52638393	Downstream
chr13	52683256	52683590	Intron
chr13	52754072	52754274	Intron
chr13	52754397	52755746	Exon
chr13	52770229	52772376	Intron
chr13	52775321	52775573	Intron
chr13	52975221	52975434	Intron
chr13	53104068	53105029	Intron
chr13	53113067	53113438	Intron
chr13	53113513	53113838	Intron
chr13	53113875	53114566	Intron
chr13	53114635	53115011	Intron
chr13	53325921	53326720	Distal Intergenic
chr13	53434288	53434948	Distal Intergenic
chr13	53435125	53435437	Distal Intergenic
chr13	53813073	53813829	Distal Intergenic
chr13	54196457	54197237	Distal Intergenic
chr13	54635276	54635475	Distal Intergenic
chr13	54691800	54692022	Distal Intergenic
chr13	54706564	54707586	Exon
chr13	54707821	54709139	Distal Intergenic
chr13	54709497	54711033	Distal Intergenic
chr13	54729995	54731238	Distal Intergenic
chr13	54787823	54788898	Distal Intergenic
chr13	55290702	55291654	Distal Intergenic
chr13	55420228	55421482	Distal Intergenic
chr13	55431425	55431624	Distal Intergenic
chr13	55463609	55464202	Distal Intergenic

chr13	55501319	55501660	Distal Intergenic
chr13	55505293	55506400	Distal Intergenic
chr13	55611408	55614478	Distal Intergenic
chr13	55705944	55706143	Distal Intergenic
chr13	55724399	55724598	Distal Intergenic
chr13	55793701	55794579	Distal Intergenic
chr13	55828924	55829193	Distal Intergenic
chr13	55927492	55928256	Distal Intergenic
chr13	55928360	55931705	Distal Intergenic
chr13	55932141	55932581	Distal Intergenic
chr13	55933364	55933563	Distal Intergenic
chr13	55933737	55934042	Distal Intergenic
chr13	55994231	55994850	Distal Intergenic
chr13	55996580	55997415	Distal Intergenic
chr13	56038135	56038334	Distal Intergenic
chr13	56038509	56038798	Distal Intergenic
chr13	56039190	56039443	Distal Intergenic
chr13	56061426	56062144	Distal Intergenic
chr13	56062342	56062621	Distal Intergenic
chr13	56062709	56063351	Distal Intergenic
chr13	56063535	56064057	Distal Intergenic
chr13	56077123	56079068	Distal Intergenic
chr13	56093914	56094476	Distal Intergenic
chr13	56105405	56106419	Distal Intergenic
chr13	56106803	56108455	Distal Intergenic
chr13	56129180	56129379	Distal Intergenic
chr13	56129528	56129780	Distal Intergenic
chr13	56425482	56425725	Distal Intergenic
chr13	56505812	56507644	Distal Intergenic
chr13	56531735	56532109	Distal Intergenic
chr13	56532158	56532557	Distal Intergenic
chr13	56539013	56539320	Distal Intergenic
chr13	56539374	56539602	Distal Intergenic
chr13	56542185	56543009	Distal Intergenic
chr13	56544017	56545700	Distal Intergenic
chr13	56683477	56683779	Distal Intergenic
chr13	56684248	56684552	Distal Intergenic
chr13	56687011	56688054	Distal Intergenic
chr13	56690001	56690900	Distal Intergenic
chr13	56695355	56695573	Distal Intergenic
chr13	56699881	56700667	Distal Intergenic
chr13	56701101	56701327	Distal Intergenic
chr13	56701463	56701777	Distal Intergenic
chr13	56701830	56704049	Distal Intergenic

chr13	56709049	56709269	Distal Intergenic
chr13	56709373	56709790	Distal Intergenic
chr13	56710155	56711008	Distal Intergenic
chr13	56711225	56711468	Distal Intergenic
chr13	56714387	56715877	Distal Intergenic
chr13	56804409	56804647	Distal Intergenic
chr13	56813351	56813636	Distal Intergenic
chr13	57959946	57960154	Distal Intergenic
chr13	58228036	58228292	Intron
chr13	58229238	58229870	Intron
chr13	58230361	58231603	Intron
chr13	58315987	58316336	Distal Intergenic
chr13	58502030	58502799	Distal Intergenic
chr13	58503029	58503341	Distal Intergenic
chr13	58504032	58504246	Distal Intergenic
chr13	58504397	58504618	Distal Intergenic
chr13	58621887	58622139	Distal Intergenic
chr13	58635719	58636785	Distal Intergenic
chr13	59725362	59726330	Distal Intergenic
chr13	59747931	59748877	Distal Intergenic
chr13	59871641	59871857	Distal Intergenic
chr13	59925878	59926091	Distal Intergenic
chr13	59958080	59958378	Distal Intergenic
chr13	59977149	59977722	Distal Intergenic
chr13	60120505	60121768	Distal Intergenic
chr13	60145727	60146744	Distal Intergenic
chr13	60146829	60147046	Distal Intergenic
chr13	60148450	60150387	Distal Intergenic
chr13	60152408	60152752	Distal Intergenic
chr13	60411192	60411392	Intron
chr13	60528758	60529036	Intron
chr13	60529277	60529695	Intron
chr13	60719630	60720231	Promoter
chr13	60759490	60760794	Distal Intergenic
chr13	60761371	60762849	Distal Intergenic
chr13	60763425	60764077	Distal Intergenic
chr13	60998926	60999447	Intron
chr13	63147409	63147823	Distal Intergenic
chr13	63147926	63148417	Distal Intergenic
chr13	63166784	63168905	Distal Intergenic
chr13	63171884	63172225	Distal Intergenic
chr13	63172593	63176418	Distal Intergenic
chr13	63188893	63191939	Distal Intergenic
chr13	63193521	63195524	Distal Intergenic

chr13	63218883	63219748	Distal Intergenic
chr13	63220946	63222944	Distal Intergenic
chr13	63257438	63257797	Distal Intergenic
chr13	63287866	63289359	Distal Intergenic
chr13	63294523	63294722	Distal Intergenic
chr13	63318086	63318299	Distal Intergenic
chr13	63318410	63318612	Distal Intergenic
chr13	63341511	63343105	Distal Intergenic
chr13	63343143	63344052	Distal Intergenic
chr13	63344169	63344411	Distal Intergenic
chr13	63344466	63344843	Distal Intergenic
chr13	63353460	63355316	Distal Intergenic
chr13	63356056	63356481	Distal Intergenic
chr13	63367316	63367898	Distal Intergenic
chr13	63375428	63376302	Distal Intergenic
chr13	63379379	63380198	Distal Intergenic
chr13	63380270	63380575	Distal Intergenic
chr13	63380784	63382124	Distal Intergenic
chr13	63382314	63382513	Distal Intergenic
chr13	63405605	63406921	Distal Intergenic
chr13	63407104	63407679	Distal Intergenic
chr13	63665151	63666217	Distal Intergenic
chr13	63675909	63676111	Distal Intergenic
chr13	63677130	63677393	Distal Intergenic
chr13	63677593	63678299	Distal Intergenic
chr13	63773775	63774148	Distal Intergenic
chr13	63783811	63784382	Distal Intergenic
chr13	64255470	64255822	Distal Intergenic
chr13	64256061	64256279	Distal Intergenic
chr13	64357869	64360093	Distal Intergenic
chr13	64408988	64409507	Intron
chr13	64409699	64409921	Intron
chr13	64432850	64434445	Distal Intergenic
chr13	64438572	64440204	Distal Intergenic
chr13	64471277	64471580	Distal Intergenic
chr13	64471781	64472128	Distal Intergenic
chr13	64478825	64479068	Distal Intergenic
chr13	64489752	64490585	Distal Intergenic
chr13	64501920	64503702	Distal Intergenic
chr13	64522747	64523134	Distal Intergenic
chr13	64523229	64524132	Distal Intergenic
chr13	65213860	65214517	Distal Intergenic
chr13	67005644	67006019	Intron
chr13	67033582	67033868	Intron

chr13	67034022	67034404	Intron
chr13	67209315	67209566	Intron
chr13	67461146	67461360	Intron
chr13	67653534	67654039	Intron
chr13	67885058	67885266	Distal Intergenic
chr13	67885318	67885727	Distal Intergenic
chr13	70861933	70863911	Distal Intergenic
chr13	70883505	70883769	Distal Intergenic
chr13	70932106	70932305	Distal Intergenic
chr13	70932598	70932910	Distal Intergenic
chr13	70932998	70933210	Distal Intergenic
chr13	70933315	70933834	Distal Intergenic
chr13	71785501	71787897	Distal Intergenic
chr13	71789238	71789597	Distal Intergenic
chr13	71796328	71796527	Distal Intergenic
chr13	71948782	71949430	Distal Intergenic
chr13	71949637	71949869	Distal Intergenic
chr13	72100069	72100367	Intron
chr13	72335088	72337053	Intron
chr13	73511213	73512060	Intron
chr13	73512100	73512657	Intron
chr13	73567371	73567953	Intron
chr13	73568338	73568555	Intron
chr13	73568686	73570562	Intron
chr13	73643255	73643504	Intron
chr13	74163007	74163972	Distal Intergenic
chr13	74307415	74307634	Intron
chr13	74438657	74440042	Intron
chr13	74454291	74454772	Intron
chr13	74776694	74777693	Distal Intergenic
chr13	74902802	74903011	Distal Intergenic
chr13	74904823	74905120	Distal Intergenic
chr13	74905185	74905468	Distal Intergenic
chr13	74906263	74907548	Distal Intergenic
chr13	74907618	74910808	Distal Intergenic
chr13	74944707	74946469	Distal Intergenic
chr13	74959139	74959893	Distal Intergenic
chr13	75597971	75598765	Distal Intergenic
chr13	75680826	75681083	Distal Intergenic
chr13	75846083	75846314	Distal Intergenic
chr13	75846353	75846570	Distal Intergenic
chr13	75889670	75890248	Promoter
chr13	75891015	75891240	Promoter
chr13	75891292	75892481	Promoter

chr13	75926340	75927535	Intron
chr13	75935000	75935349	Intron
chr13	75935557	75936593	Exon
chr13	75962553	75962958	Intron
chr13	76155472	76155943	Intron
chr13	76239020	76239988	Intron
chr13	76260062	76261494	Intron
chr13	81024574	81024792	Distal Intergenic
chr13	81025069	81025338	Distal Intergenic
chr13	81025491	81025690	Distal Intergenic
chr13	81235387	81235616	Distal Intergenic
chr13	81315280	81315623	Distal Intergenic
chr13	81315815	81316136	Distal Intergenic
chr13	81316562	81317324	Distal Intergenic
chr13	81818459	81818772	Distal Intergenic
chr13	81822419	81822680	Distal Intergenic
chr13	81822993	81824318	Distal Intergenic
chr13	82121203	82121473	Distal Intergenic
chr13	82121601	82121800	Distal Intergenic
chr13	85427658	85428069	Distal Intergenic
chr13	85428555	85428786	Distal Intergenic
chr13	86185686	86186279	Distal Intergenic
chr13	90356311	90356806	Distal Intergenic
chr13	90714935	90715135	Intron
chr13	90936265	90937628	Distal Intergenic
chr13	91062546	91063001	Distal Intergenic
chr13	91116727	91117307	Distal Intergenic
chr13	91120205	91122445	Distal Intergenic
chr13	91177597	91181632	Exon
chr13	91303751	91304900	Distal Intergenic
chr13	91304990	91306323	Distal Intergenic
chr13	91317313	91319284	Distal Intergenic
chr13	91345746	91346279	Distal Intergenic
chr13	91356575	91357094	Distal Intergenic
chr13	91359242	91360087	Distal Intergenic
chr13	91363360	91363585	Distal Intergenic
chr13	91363767	91364687	Distal Intergenic
chr13	91445315	91446758	Distal Intergenic
chr13	91447478	91448326	Distal Intergenic
chr13	91465919	91467376	Distal Intergenic
chr13	91489066	91489586	Distal Intergenic
chr13	91506345	91506695	Distal Intergenic
chr13	91789037	91789512	Intron
chr13	91789713	91789937	Intron

chr13	91941029	91941952	Distal Intergenic
chr13	91947030	91947229	Distal Intergenic
chr13	91947289	91947517	Distal Intergenic
chr13	92842377	92842727	Intron
chr13	93445259	93445886	Intron
chr13	93446073	93446948	Intron
chr13	93961006	93961408	Intron
chr13	93961563	93962116	Intron
chr13	94192970	94193615	Intron
chr13	94254359	94254712	Intron
chr13	94262406	94262792	Intron
chr13	94263371	94263780	Intron
chr13	94267003	94267300	Intron
chr13	94267611	94267898	Intron
chr13	94762092	94763161	Intron
chr13	94767690	94767929	Intron
chr13	94768198	94768804	Intron
chr13	95199547	95199777	Distal Intergenic
chr13	95587623	95588402	Exon
chr13	95597757	95598281	Distal Intergenic
chr13	95598367	95600830	Distal Intergenic
chr13	96254666	96255009	Intron
chr13	96258783	96259595	Intron
chr13	96284762	96285124	Intron
chr13	96285177	96285410	Intron
chr13	96410514	96411794	Intron
chr13	96413730	96414172	Intron
chr13	96560685	96562424	Intron
chr13	96564052	96564766	Intron
chr13	96780852	96781552	Intron
chr13	96785211	96785707	Intron
chr13	96785911	96786132	Intron
chr13	97257406	97257788	Intron
chr13	97280567	97281579	Intron
chr13	97312259	97312557	Intron
chr13	97344064	97344272	Intron
chr13	97344367	97344597	Intron
chr13	97439484	97439747	Intron
chr13	97439892	97440838	Intron
chr13	97441262	97441710	Intron
chr13	97450534	97451180	Intron
chr13	97451247	97451471	Intron
chr13	97907963	97908162	Intron
chr13	97971130	97971467	Intron

chr13	98018277	98018509	Intron
chr13	98018827	98020165	Intron
chr13	98252599	98252848	Distal Intergenic
chr13	98253302	98253755	Distal Intergenic
chr13	98254320	98256187	Distal Intergenic
chr13	98257925	98258129	Distal Intergenic
chr13	98267055	98267285	Distal Intergenic
chr13	98267841	98268078	Distal Intergenic
chr13	98612342	98612555	Intron
chr13	98697946	98698359	Distal Intergenic
chr13	98698488	98698687	Distal Intergenic
chr13	98783545	98784130	Distal Intergenic
chr13	98784251	98785441	Distal Intergenic
chr13	98785509	98786051	Distal Intergenic
chr13	98786100	98786393	Distal Intergenic
chr13	98797134	98798267	Intron
chr13	98808593	98808921	Intron
chr13	98808963	98809360	Intron
chr13	98891566	98892863	Intron
chr13	98902835	98903108	Intron
chr13	99086360	99086623	Intron
chr13	99086670	99086945	Intron
chr13	99204420	99204678	Intron
chr13	99204757	99205315	Intron
chr13	99393004	99393272	Intron
chr13	99402275	99402746	Intron
chr13	99404932	99405217	Promoter
chr13	99449570	99450287	Exon
chr13	99638329	99638668	Intron
chr13	99638763	99639013	Intron
chr13	99663915	99664493	Intron
chr13	99695243	99695457	Intron
chr13	99940487	99940748	Intron
chr13	99945235	99945506	Intron
chr13	100008662	100008873	Promoter
chr13	100077788	100078019	Distal Intergenic
chr13	100078651	100078874	Distal Intergenic
chr13	100101509	100101955	Distal Intergenic
chr13	100148478	100148920	Intron
chr13	100154030	100154386	Promoter
chr13	100154472	100155472	Promoter
chr13	100161340	100161832	Intron
chr13	100161881	100162125	Intron
chr13	100212720	100213595	Intron

chr13	100217280	100218061	Distal Intergenic
chr13	100219142	100220532	Distal Intergenic
chr13	100229147	100229694	Distal Intergenic
chr13	100245561	100245793	Distal Intergenic
chr13	100362677	100362928	Intron
chr13	100531320	100531909	Intron
chr13	100708828	100709389	Distal Intergenic
chr13	100717611	100717972	Distal Intergenic
chr13	100733666	100733901	Distal Intergenic
chr13	100873401	100873736	Intron
chr13	101286508	101286794	Intron
chr13	101421099	101421416	Intron
chr13	101500260	101500872	Intron
chr13	103342768	103343879	Exon
chr13	103352556	103352767	Intron
chr13	103354119	103354648	Intron
chr13	103364370	103364677	Distal Intergenic
chr13	103638781	103639338	Distal Intergenic
chr13	103670215	103670446	Distal Intergenic
chr13	103670558	103671071	Distal Intergenic
chr13	103678913	103679639	Distal Intergenic
chr13	103685975	103686498	Distal Intergenic
chr13	103687764	103688346	Distal Intergenic
chr13	103688409	103689416	Distal Intergenic
chr13	103689479	103690185	Distal Intergenic
chr13	103690744	103690948	Distal Intergenic
chr13	103695392	103696571	3' UTR
chr13	103748618	103749029	Distal Intergenic
chr13	104176866	104177163	Distal Intergenic
chr13	104177361	104177659	Distal Intergenic
chr13	104564057	104564286	Distal Intergenic
chr13	104709631	104710087	Distal Intergenic
chr13	104710303	104710713	Distal Intergenic
chr13	104768098	104769022	Distal Intergenic
chr13	104831166	104831840	Distal Intergenic
chr13	104864885	104865238	Distal Intergenic
chr13	105310413	105311762	Distal Intergenic
chr13	105606574	105607209	Distal Intergenic
chr13	108601454	108603098	Distal Intergenic
chr13	108603289	108605284	Distal Intergenic
chr13	108617184	108617454	Distal Intergenic
chr13	108617789	108619140	Distal Intergenic
chr13	108620025	108621438	Distal Intergenic
chr13	108623843	108624042	Distal Intergenic

chr13	108656623	108656888	Distal Intergenic
chr13	108833339	108833776	Distal Intergenic
chr13	108833860	108834566	Distal Intergenic
chr13	108834840	108835104	Distal Intergenic
chr13	108835207	108835532	Distal Intergenic
chr13	108835779	108836335	Distal Intergenic
chr13	109127759	109128080	Distal Intergenic
chr13	110033125	110033327	Distal Intergenic
chr13	110050194	110051576	Distal Intergenic
chr13	110058805	110059635	Distal Intergenic
chr13	110068589	110069020	Distal Intergenic
chr13	110069089	110069804	Distal Intergenic
chr13	110609388	110610974	Distal Intergenic
chr13	110611543	110612921	Distal Intergenic
chr13	110613146	110614388	Distal Intergenic
chr13	110688143	110688797	Distal Intergenic
chr13	111008687	111008939	Intron
chr13	111009032	111009281	Intron
chr13	111185260	111185744	Intron
chr13	111331797	111331996	Intron
chr13	111514427	111516403	Exon
chr13	111698061	111698417	Distal Intergenic
chr13	111704004	111704328	Distal Intergenic
chr13	111704455	111704673	Distal Intergenic
chr13	111715436	111716059	Distal Intergenic
chr13	111753610	111754081	Distal Intergenic
chr13	111756736	111758390	Distal Intergenic
chr13	112279841	112280091	Distal Intergenic
chr13	112817799	112818842	Distal Intergenic
chr13	112845159	112845374	Distal Intergenic
chr13	112861195	112862356	Distal Intergenic
chr13	113172991	113173509	Exon
chr13	113548300	113548851	Distal Intergenic
chr13	113560693	113560924	Intron
chr13	113561003	113561241	Intron
chr13	113954391	113955025	Intron
chr13	114048899	114049168	Distal Intergenic
chr13	114134424	114135231	Exon
chr13	114141147	114141670	Intron
chr13	114285324	114286687	Exon
chr13	114391534	114391735	Intron
chr13	114635008	114635216	Distal Intergenic
chr13	115419755	115420208	Distal Intergenic
chr13	115420371	115420599	Distal Intergenic

chr13	115425488	115426548	Distal Intergenic
chr13	115428750	115430822	Distal Intergenic
chr13	115433443	115433645	Distal Intergenic
chr13	115433837	115434631	Distal Intergenic
chr13	115469505	115469720	Distal Intergenic
chr13	115525044	115525379	Distal Intergenic
chr13	115525613	115526804	Distal Intergenic
chr13	115720733	115720941	Distal Intergenic
chr13	115735302	115736961	Distal Intergenic
chr13	117617750	117617964	Distal Intergenic
chr13	117739045	117740529	Distal Intergenic
chr14	3271936	3272306	Distal Intergenic
chr14	3279989	3280385	Distal Intergenic
chr14	3627463	3627812	Distal Intergenic
chr14	3858840	3859146	Distal Intergenic
chr14	3866766	3867066	Distal Intergenic
chr14	4706341	4706651	Distal Intergenic
chr14	5051181	5051512	Distal Intergenic
chr14	7166690	7166931	Distal Intergenic
chr14	7490510	7490995	Distal Intergenic
chr14	8640784	8641587	Distal Intergenic
chr14	8641711	8642664	Distal Intergenic
chr14	8642858	8643070	Distal Intergenic
chr14	8645119	8645340	Distal Intergenic
chr14	8650575	8650821	Distal Intergenic
chr14	8651067	8651606	Distal Intergenic
chr14	8651643	8652349	Distal Intergenic
chr14	8658763	8659529	Distal Intergenic
chr14	8678558	8679560	Distal Intergenic
chr14	8679648	8685294	Distal Intergenic
chr14	8686033	8686757	Distal Intergenic
chr14	8693306	8693662	Distal Intergenic
chr14	8693848	8694688	Distal Intergenic
chr14	8756931	8757377	Distal Intergenic
chr14	8784726	8787861	Distal Intergenic
chr14	8788059	8788571	Distal Intergenic
chr14	8788952	8789480	Distal Intergenic
chr14	8793780	8794438	Distal Intergenic
chr14	8794482	8795048	Distal Intergenic
chr14	8795117	8795339	Distal Intergenic
chr14	8831036	8832088	Distal Intergenic
chr14	8913090	8913289	Distal Intergenic
chr14	8931055	8932810	Distal Intergenic
chr14	8934298	8934532	Distal Intergenic

chr14	8941525	8942063	Distal Intergenic
chr14	8943464	8944757	Distal Intergenic
chr14	8957278	8960014	Distal Intergenic
chr14	8960293	8960798	Distal Intergenic
chr14	9126631	9127324	Distal Intergenic
chr14	9189610	9189809	Distal Intergenic
chr14	9274953	9275687	Distal Intergenic
chr14	9442678	9443070	Distal Intergenic
chr14	10055759	10056738	Distal Intergenic
chr14	10057411	10057628	Distal Intergenic
chr14	10111020	10111235	Distal Intergenic
chr14	10113774	10114736	Distal Intergenic
chr14	10117063	10117302	Distal Intergenic
chr14	10117688	10118135	Distal Intergenic
chr14	10152073	10152811	Distal Intergenic
chr14	10264645	10265711	Distal Intergenic
chr14	10378754	10380648	Distal Intergenic
chr14	10380701	10381094	Distal Intergenic
chr14	10960934	10961391	Distal Intergenic
chr14	10961497	10961918	Distal Intergenic
chr14	12386307	12386665	Distal Intergenic
chr14	12448311	12449055	Distal Intergenic
chr14	12516090	12516404	Distal Intergenic
chr14	12643452	12643899	Distal Intergenic
chr14	12898197	12898773	Distal Intergenic
chr14	12898989	12899720	Distal Intergenic
chr14	13116745	13117510	Distal Intergenic
chr14	13488888	13489311	Distal Intergenic
chr14	14249025	14249927	Distal Intergenic
chr14	14249986	14250185	Distal Intergenic
chr14	14793167	14793386	Distal Intergenic
chr14	15323303	15323582	Distal Intergenic
chr14	15350545	15352737	Distal Intergenic
chr14	15465541	15465740	Distal Intergenic
chr14	16264665	16265565	Distal Intergenic
chr14	17075900	17076648	Distal Intergenic
chr14	17139966	17140529	Distal Intergenic
chr14	17140668	17140957	Distal Intergenic
chr14	17141006	17141406	Distal Intergenic
chr14	17141451	17141763	Distal Intergenic
chr14	17141941	17142569	Distal Intergenic
chr14	17197671	17198072	Distal Intergenic
chr14	17198269	17198763	Distal Intergenic
chr14	17198825	17199099	Distal Intergenic

chr14	17201295	17202211	Distal Intergenic
chr14	17316426	17316695	Distal Intergenic
chr14	17316743	17317112	Distal Intergenic
chr14	17345559	17346066	Distal Intergenic
chr14	17427125	17427356	Distal Intergenic
chr14	17430522	17430721	Distal Intergenic
chr14	17896015	17896467	Distal Intergenic
chr14	19057814	19058024	Distal Intergenic
chr14	19066075	19067299	Distal Intergenic
chr14	19071413	19071612	Distal Intergenic
chr14	19103020	19103257	Distal Intergenic
chr14	19103311	19103569	Distal Intergenic
chr14	19104091	19104330	Distal Intergenic
chr14	19397092	19398345	Distal Intergenic
chr14	19606306	19607473	Exon
chr14	19680594	19681094	Promoter
chr14	20305862	20306555	Distal Intergenic
chr14	20787408	20788246	Intron
chr14	20902145	20902344	Intron
chr14	20902522	20902739	Intron
chr14	21167034	21167329	Intron
chr14	21167417	21167980	5' UTR
chr14	21296786	21299488	Distal Intergenic
chr14	21322115	21322684	Distal Intergenic
chr14	21364700	21365543	Distal Intergenic
chr14	21365581	21365811	Distal Intergenic
chr14	21428067	21429974	Distal Intergenic
chr14	21450058	21450830	Distal Intergenic
chr14	21460231	21460506	3' UTR
chr14	21492877	21493176	Promoter
chr14	21493502	21494013	Promoter
chr14	21494062	21494301	Promoter
chr14	21521330	21521671	Intron
chr14	21522042	21522750	Intron
chr14	21623746	21624066	Promoter
chr14	21624240	21626358	Promoter
chr14	21646707	21647111	Distal Intergenic
chr14	21647186	21647989	Distal Intergenic
chr14	21648211	21648463	Distal Intergenic
chr14	21651051	21651501	Distal Intergenic
chr14	21652880	21653320	Distal Intergenic
chr14	21655957	21656297	Distal Intergenic
chr14	21663803	21667657	Downstream
chr14	21669431	21670361	Exon

chr14	21722326	21722536	Intron
chr14	21749199	21749616	Distal Intergenic
chr14	21757374	21758057	Intron
chr14	21788543	21788849	Promoter
chr14	21788901	21789818	Promoter
chr14	21791813	21793458	Promoter
chr14	21884859	21885573	Intron
chr14	21905252	21907967	Promoter
chr14	21954422	21954858	Promoter
chr14	22170562	22170877	Distal Intergenic
chr14	22366427	22367553	Intron
chr14	22567184	22567393	Intron
chr14	22567506	22567914	Intron
chr14	22570237	22570637	Intron
chr14	22649996	22650414	Intron
chr14	22650465	22651338	Intron
chr14	22884958	22885396	Intron
chr14	23468661	23469995	Promoter
chr14	23470323	23470584	Promoter
chr14	24576084	24577649	Intron
chr14	24577753	24577953	Intron
chr14	25001638	25002037	Distal Intergenic
chr14	25002073	25002798	Distal Intergenic
chr14	25012121	25014460	Distal Intergenic
chr14	25025252	25025805	Distal Intergenic
chr14	25025971	25027251	Distal Intergenic
chr14	25032111	25032414	Distal Intergenic
chr14	25032505	25033342	Distal Intergenic
chr14	25042209	25042434	Downstream
chr14	25047067	25048487	Distal Intergenic
chr14	25063332	25063553	Distal Intergenic
chr14	25063624	25063990	Distal Intergenic
chr14	25064055	25064718	Distal Intergenic
chr14	25091544	25092290	Distal Intergenic
chr14	25092480	25092735	Distal Intergenic
chr14	25130613	25131037	Distal Intergenic
chr14	25288022	25289015	Promoter
chr14	25309895	25310103	Intron
chr14	25851060	25851692	Distal Intergenic
chr14	25957321	25957553	Distal Intergenic
chr14	26006350	26006831	Distal Intergenic
chr14	26182273	26182974	Distal Intergenic
chr14	26184767	26185007	Distal Intergenic
chr14	26185043	26185416	Distal Intergenic

chr14	26185494	26185779	Distal Intergenic
chr14	26197432	26198947	Distal Intergenic
chr14	26200226	26200656	Distal Intergenic
chr14	26233895	26235173	Distal Intergenic
chr14	26278740	26279826	Distal Intergenic
chr14	26280024	26280375	Distal Intergenic
chr14	26280899	26281238	Distal Intergenic
chr14	26283866	26284226	Distal Intergenic
chr14	26284300	26284536	Distal Intergenic
chr14	26293502	26297179	Distal Intergenic
chr14	26306246	26306818	Distal Intergenic
chr14	26346290	26346712	Distal Intergenic
chr14	26346963	26347380	Distal Intergenic
chr14	26353590	26353852	Distal Intergenic
chr14	26405723	26409397	Distal Intergenic
chr14	26418699	26420741	Distal Intergenic
chr14	26425849	26426166	Distal Intergenic
chr14	26446658	26447812	Distal Intergenic
chr14	26449380	26449590	Distal Intergenic
chr14	26449759	26450107	Distal Intergenic
chr14	26455502	26456768	Distal Intergenic
chr14	26520199	26520752	Distal Intergenic
chr14	26520869	26521127	Distal Intergenic
chr14	26662051	26662458	Distal Intergenic
chr14	26681210	26681886	Distal Intergenic
chr14	26746798	26747112	Distal Intergenic
chr14	26886492	26886745	Distal Intergenic
chr14	27326806	27327349	Distal Intergenic
chr14	27344340	27344574	Distal Intergenic
chr14	27352730	27352984	Distal Intergenic
chr14	27353050	27353490	Distal Intergenic
chr14	27354126	27354645	Distal Intergenic
chr14	27360134	27361013	Distal Intergenic
chr14	27398747	27398946	Distal Intergenic
chr14	27399440	27399668	Distal Intergenic
chr14	27457144	27457544	Distal Intergenic
chr14	27457704	27459008	Distal Intergenic
chr14	27488641	27489631	Distal Intergenic
chr14	27882732	27883544	Intron
chr14	28062463	28062821	Intron
chr14	28062858	28063124	Intron
chr14	28112989	28113331	Intron
chr14	28114291	28114531	Intron
chr14	28115024	28115390	Intron

chr14	28154311	28155005	Distal Intergenic
chr14	28160228	28160853	Distal Intergenic
chr14	28163880	28165311	Distal Intergenic
chr14	28188976	28190025	Distal Intergenic
chr14	28242030	28242229	Distal Intergenic
chr14	28242368	28243027	Distal Intergenic
chr14	28747161	28747382	Distal Intergenic
chr14	29182231	29184843	Distal Intergenic
chr14	29243778	29243992	Intron
chr14	29319019	29319474	Distal Intergenic
chr14	29319604	29319950	Distal Intergenic
chr14	29320056	29320349	Distal Intergenic
chr14	29320648	29321122	Distal Intergenic
chr14	29338365	29338893	Distal Intergenic
chr14	29355465	29356021	Distal Intergenic
chr14	29357585	29358041	Distal Intergenic
chr14	29358299	29358603	Distal Intergenic
chr14	29358853	29359327	Distal Intergenic
chr14	29375658	29377249	Distal Intergenic
chr14	29377363	29377580	Distal Intergenic
chr14	29621444	29621645	Distal Intergenic
chr14	29885712	29886401	Distal Intergenic
chr14	29893153	29895183	Downstream
chr14	29895411	29896060	Downstream
chr14	30082050	30082291	Intron
chr14	30095697	30097446	Exon
chr14	30309996	30310303	Intron
chr14	30310418	30310664	Intron
chr14	30781509	30782191	Distal Intergenic
chr14	31292827	31293182	Distal Intergenic
chr14	31316345	31317276	Distal Intergenic
chr14	31344055	31344254	Promoter
chr14	31344657	31346426	Promoter
chr14	31346504	31346752	Intron
chr14	31362038	31362589	Intron
chr14	31374463	31375422	Exon
chr14	31406429	31406953	Intron
chr14	31407032	31407296	Intron
chr14	31409240	31409748	Intron
chr14	31434084	31436178	Intron
chr14	31436241	31436458	Intron
chr14	31436602	31437585	Intron
chr14	31437786	31438744	Intron
chr14	31438828	31439036	Intron

chr14	31439226	31439454	Intron
chr14	31787190	31787716	Exon
chr14	31813970	31814529	Exon
chr14	31831786	31832243	Intron
chr14	31952871	31953321	5' UTR
chr14	31967054	31967383	Distal Intergenic
chr14	31973005	31974382	Distal Intergenic
chr14	31978104	31980242	Distal Intergenic
chr14	32011016	32011659	Distal Intergenic
chr14	32011695	32012109	Distal Intergenic
chr14	32018324	32019488	Distal Intergenic
chr14	32217162	32218189	Intron
chr14	32261111	32261581	Intron
chr14	32261969	32262180	Intron
chr14	32308044	32309213	Intron
chr14	32475023	32475689	Distal Intergenic
chr14	32475788	32476159	Distal Intergenic
chr14	32482220	32482508	Distal Intergenic
chr14	32482709	32483849	Distal Intergenic
chr14	32594664	32595193	Intron
chr14	32757904	32758122	Distal Intergenic
chr14	32758351	32758550	Distal Intergenic
chr14	32758690	32759391	Distal Intergenic
chr14	32759429	32759749	Distal Intergenic
chr14	32898201	32898436	Intron
chr14	32899686	32899885	Intron
chr14	32976952	32978972	Intron
chr14	33014313	33015285	Exon
chr14	33306266	33306488	Distal Intergenic
chr14	34163951	34164167	Intron
chr14	34164242	34166060	Intron
chr14	34537113	34538560	Distal Intergenic
chr14	34637192	34638824	Distal Intergenic
chr14	34643020	34643535	Distal Intergenic
chr14	34898297	34901407	Downstream
chr14	35110295	35110532	Distal Intergenic
chr14	35124633	35124851	Distal Intergenic
chr14	35175838	35177333	Downstream
chr14	35186889	35188320	Distal Intergenic
chr14	35188640	35190519	Distal Intergenic
chr14	35191754	35192566	Distal Intergenic
chr14	35280785	35280984	Intron
chr14	35314820	35315139	Intron
chr14	35316003	35318139	Intron

chr14	35341040	35341246	Intron
chr14	35361512	35362664	Distal Intergenic
chr14	35481641	35481990	Intron
chr14	35487226	35488315	Exon
chr14	37512079	37512730	Intron
chr14	37512782	37513180	Intron
chr14	37698173	37698627	Intron
chr14	37698828	37699344	Intron
chr14	37745958	37746199	Intron
chr14	37948279	37948506	Intron
chr14	41716100	41716494	Distal Intergenic
chr14	41760713	41764278	Distal Intergenic
chr14	41768981	41770371	Distal Intergenic
chr14	41808765	41810151	Distal Intergenic
chr14	41844501	41844918	Distal Intergenic
chr14	44448973	44449474	Distal Intergenic
chr14	45660444	45660757	Intron
chr14	45810343	45810881	Distal Intergenic
chr14	45810919	45811342	Distal Intergenic
chr14	45949522	45949999	Distal Intergenic
chr14	46062401	46062871	Distal Intergenic
chr14	46098758	46099097	Distal Intergenic
chr14	46111971	46112492	Distal Intergenic
chr14	46791860	46792691	Intron
chr14	46792750	46793081	Intron
chr14	47136432	47136631	Distal Intergenic
chr14	47137242	47137462	Distal Intergenic
chr14	47256266	47256465	Distal Intergenic
chr14	47407372	47407587	Intron
chr14	47410115	47410810	Intron
chr14	47420204	47421474	Intron
chr14	47585565	47586062	Intron
chr14	47586166	47586418	Intron
chr14	47641704	47642653	Intron
chr14	47896648	47897269	Intron
chr14	47917862	47918264	Intron
chr14	47918436	47918858	Intron
chr14	47970664	47971239	Intron
chr14	47973525	47974060	Intron
chr14	47994016	47994215	Intron
chr14	48138526	48138739	Intron
chr14	48152217	48153438	Distal Intergenic
chr14	48235402	48236193	Exon
chr14	48244195	48245959	Intron

chr14	48253655	48254647	Intron
chr14	48771979	48772231	Distal Intergenic
chr14	49685068	49685715	Distal Intergenic
chr14	49686143	49687056	Distal Intergenic
chr14	49692433	49694445	Distal Intergenic
chr14	49694505	49694704	Distal Intergenic
chr14	51543625	51543993	Intron
chr14	51544077	51544365	Intron
chr14	51550844	51552050	Intron
chr14	51552550	51553011	Intron
chr14	51563625	51563919	Distal Intergenic
chr14	51564172	51564381	Distal Intergenic
chr14	51564465	51566896	Distal Intergenic
chr14	51576894	51578160	Distal Intergenic
chr14	51710450	51710649	5' UTR
chr14	51710866	51712762	5' UTR
chr14	51713108	51714394	Exon
chr14	51720554	51721069	Exon
chr14	51818747	51819084	Intron
chr14	51819271	51820286	Intron
chr14	52477766	52477966	Intron
chr14	52485018	52487139	Exon
chr14	52490558	52490821	Intron
chr14	52552104	52552893	Distal Intergenic
chr14	52579942	52580169	Distal Intergenic
chr14	52633359	52633772	Distal Intergenic
chr14	52634496	52636104	Distal Intergenic
chr14	52639079	52639370	Distal Intergenic
chr14	52639541	52639785	Distal Intergenic
chr14	52639842	52640481	Distal Intergenic
chr14	52640525	52640880	Distal Intergenic
chr14	52642834	52643206	Distal Intergenic
chr14	52723365	52723688	Distal Intergenic
chr14	52816238	52816689	Distal Intergenic
chr14	52816730	52817557	Distal Intergenic
chr14	52899187	52899438	Exon
chr14	52924228	52925007	Intron
chr14	54841854	54843010	Distal Intergenic
chr14	55049949	55050557	Intron
chr14	55050967	55054573	Intron
chr14	55082572	55082919	Intron
chr14	55111136	55112041	Intron
chr14	55191023	55191491	Intron
chr14	55191543	55191849	Intron

chr14	55192067	55194217	Intron
chr14	55194261	55194606	Intron
chr14	55194958	55195205	Intron
chr14	55195297	55196081	Intron
chr14	55196310	55196650	Intron
chr14	55236321	55237075	Exon
chr14	55304915	55305696	Distal Intergenic
chr14	55349052	55349531	Intron
chr14	55349605	55350013	Intron
chr14	55385661	55385986	Distal Intergenic
chr14	55386137	55388464	Distal Intergenic
chr14	55501822	55502166	Intron
chr14	55502359	55502734	Intron
chr14	56209441	56209794	Distal Intergenic
chr14	56209841	56210449	Distal Intergenic
chr14	56254573	56254773	Intron
chr14	56261721	56262665	Promoter
chr14	56290227	56290489	Distal Intergenic
chr14	56290529	56291172	Distal Intergenic
chr14	56291234	56291509	Distal Intergenic
chr14	56333624	56334868	Distal Intergenic
chr14	56342807	56343023	Distal Intergenic
chr14	56343063	56343296	Distal Intergenic
chr14	56343606	56343824	Distal Intergenic
chr14	56348910	56349119	Distal Intergenic
chr14	56381967	56382166	Distal Intergenic
chr14	56383810	56385023	Distal Intergenic
chr14	56386342	56387794	Distal Intergenic
chr14	57431141	57431671	Distal Intergenic
chr14	57494034	57494815	Distal Intergenic
chr14	57504543	57504777	Distal Intergenic
chr14	57506016	57506483	Distal Intergenic
chr14	57506606	57506892	Distal Intergenic
chr14	57724709	57724997	Intron
chr14	57757001	57757556	Distal Intergenic
chr14	57757806	57758298	Distal Intergenic
chr14	57779775	57779983	Distal Intergenic
chr14	57910117	57910876	Distal Intergenic
chr14	57914276	57914758	Distal Intergenic
chr14	57915217	57915490	Distal Intergenic
chr14	57916148	57916735	Distal Intergenic
chr14	57916995	57917214	Distal Intergenic
chr14	58185263	58185479	Intron
chr14	58283063	58283614	Intron

chr14	58284075	58284311	Intron
chr14	58324188	58324690	Intron
chr14	58325954	58327235	Intron
chr14	58327350	58327631	Intron
chr14	58364540	58364924	Distal Intergenic
chr14	58469047	58470183	Downstream
chr14	58501839	58502094	Intron
chr14	58513137	58513443	Intron
chr14	59406062	59406345	Distal Intergenic
chr14	59406383	59406975	Distal Intergenic
chr14	59407022	59407542	Distal Intergenic
chr14	60870444	60870692	Distal Intergenic
chr14	61344915	61345979	Intron
chr14	61943474	61943815	Promoter
chr14	61973360	61973608	Intron
chr14	61973829	61974292	Intron
chr14	62040019	62040246	Intron
chr14	62128341	62129490	Distal Intergenic
chr14	62129526	62129726	Distal Intergenic
chr14	62214851	62218184	Promoter
chr14	62218670	62218924	Distal Intergenic
chr14	62220976	62221200	Distal Intergenic
chr14	62221355	62221767	Distal Intergenic
chr14	62221854	62222112	Distal Intergenic
chr14	62264303	62264559	Distal Intergenic
chr14	62265672	62266873	Distal Intergenic
chr14	62267375	62267685	Distal Intergenic
chr14	63335823	63336448	Intron
chr14	63379019	63380010	Intron
chr14	63443995	63444278	Intron
chr14	63444516	63445454	Intron
chr14	63559349	63559781	Intron
chr14	63741558	63741784	Intron
chr14	63742014	63742628	Intron
chr14	63750225	63750463	Intron
chr14	63750616	63751668	Intron
chr14	63767320	63768154	Distal Intergenic
chr14	64022389	64022649	Distal Intergenic
chr14	64046130	64046385	Distal Intergenic
chr14	64046433	64046834	Distal Intergenic
chr14	64065678	64067537	5' UTR
chr14	64069555	64073893	Intron
chr14	64535703	64536686	Intron
chr14	64596906	64597307	Exon

chr14	64597345	64598246	Intron
chr14	64598314	64599377	Exon
chr14	64601544	64601992	Intron
chr14	64616684	64617011	Intron
chr14	64880056	64880998	Intron
chr14	64882610	64883552	Intron
chr14	64968569	64969242	Intron
chr14	65059008	65059411	Distal Intergenic
chr14	65059465	65059771	Distal Intergenic
chr14	65059983	65060397	Distal Intergenic
chr14	65578337	65578865	Distal Intergenic
chr14	65601282	65601682	Distal Intergenic
chr14	65716178	65716548	Distal Intergenic
chr14	65719237	65720580	Distal Intergenic
chr14	65731628	65732086	Distal Intergenic
chr14	65732247	65732855	Distal Intergenic
chr14	66157395	66157603	Intron
chr14	66158386	66158641	Intron
chr14	66486072	66487167	Distal Intergenic
chr14	66646921	66647194	Distal Intergenic
chr14	66723871	66725341	Distal Intergenic
chr14	66849500	66849699	Distal Intergenic
chr14	66875975	66878539	Distal Intergenic
chr14	66885224	66885585	Distal Intergenic
chr14	67595453	67596089	Intron
chr14	67596161	67596454	Intron
chr14	67665658	67666444	Exon
chr14	67666492	67666753	Exon
chr14	67669500	67669727	Intron
chr14	67716004	67716384	Intron
chr14	67716538	67717226	Intron
chr14	68334675	68335188	Intron
chr14	68335258	68335469	Intron
chr14	68495503	68495883	Intron
chr14	68496119	68496449	Intron
chr14	68541626	68542107	Intron
chr14	68638195	68638579	Intron
chr14	68653069	68653300	Intron
chr14	68653533	68653907	Intron
chr14	69264690	69264927	Distal Intergenic
chr14	69885090	69886453	Intron
chr14	70212295	70212564	Intron
chr14	70212662	70212940	Intron
chr14	70213024	70213461	Intron

chr14	70215547	70215985	Intron
chr14	70216051	70216267	Intron
chr14	70586958	70587159	Intron
chr14	70853775	70854366	Intron
chr14	70897479	70898747	Distal Intergenic
chr14	70900393	70901506	Distal Intergenic
chr14	70903861	70904060	Distal Intergenic
chr14	70915931	70918468	Promoter
chr14	70954848	70955094	Distal Intergenic
chr14	70974454	70975419	Distal Intergenic
chr14	70975470	70975683	Distal Intergenic
chr14	70976460	70979002	Distal Intergenic
chr14	70979898	70980397	Distal Intergenic
chr14	70983701	70986137	Downstream
chr14	70999160	70999421	Intron
chr14	71166837	71167185	Distal Intergenic
chr14	71172349	71173374	Distal Intergenic
chr14	71364860	71366421	Distal Intergenic
chr14	71482444	71482686	Intron
chr14	71630176	71630376	Distal Intergenic
chr14	72195609	72196829	Exon
chr14	73076051	73076665	Exon
chr14	73125143	73125673	Intron
chr14	73183991	73184235	Intron
chr14	73184291	73184835	Intron
chr14	73234716	73235112	Intron
chr14	73235253	73235557	Intron
chr14	73286622	73286979	Intron
chr14	73323643	73324123	Intron
chr14	73324756	73325142	Intron
chr14	73510042	73511244	Distal Intergenic
chr14	73569474	73570993	Exon
chr14	73580731	73580964	Intron
chr14	73633790	73636012	Intron
chr14	73637075	73639271	Exon
chr14	73639307	73639685	Intron
chr14	73653466	73653852	Exon
chr14	73768611	73772370	Intron
chr14	73782892	73783100	Exon
chr14	73783531	73785089	Intron
chr14	73785224	73786110	Intron
chr14	73787866	73789039	Intron
chr14	73794350	73794658	Intron
chr14	73796854	73797245	Intron

chr14	73802098	73802741	Intron
chr14	73809546	73810057	Intron
chr14	73810139	73810438	Intron
chr14	73845097	73846300	Intron
chr14	73848208	73850538	Intron
chr14	73850796	73851422	Intron
chr14	73860292	73860678	Intron
chr14	73883518	73884146	Intron
chr14	73885854	73886535	Intron
chr14	73896200	73896451	Intron
chr14	73907510	73908016	Intron
chr14	74107465	74108146	Distal Intergenic
chr14	74288132	74288509	Distal Intergenic
chr14	74302332	74302531	Distal Intergenic
chr14	74418029	74418237	Promoter
chr14	74498963	74499209	Promoter
chr14	74663798	74664512	Intron
chr14	74727468	74727814	3' UTR
chr14	75132043	75133862	Intron
chr14	75136417	75137144	Exon
chr14	75184245	75184605	Intron
chr14	75187325	75187578	Intron
chr14	75187701	75188010	Intron
chr14	75222325	75223897	Distal Intergenic
chr14	75280474	75281570	Intron
chr14	75282737	75283344	Exon
chr14	75283501	75285054	Exon
chr14	75310073	75310886	Distal Intergenic
chr14	75345253	75346949	Distal Intergenic
chr14	75396127	75396919	Distal Intergenic
chr14	75445162	75445503	Distal Intergenic
chr14	75653688	75653942	Distal Intergenic
chr14	76233815	76235013	Intron
chr14	76244055	76244456	Intron
chr14	76337318	76338404	Intron
chr14	76338558	76339489	Intron
chr14	76349895	76350784	Intron
chr14	76354108	76355123	Intron
chr14	76410100	76410675	Intron
chr14	76483768	76484429	Intron
chr14	76499497	76500880	Intron
chr14	76510895	76511158	Intron
chr14	76678246	76678913	Distal Intergenic
chr14	76679117	76679507	Distal Intergenic

chr14	76878425	76879527	Intron
chr14	76904667	76905395	Intron
chr14	76905440	76905683	Intron
chr14	76934734	76935335	Intron
chr14	77052528	77052727	Distal Intergenic
chr14	77064460	77065323	Distal Intergenic
chr14	77065521	77065957	Distal Intergenic
chr14	77141781	77142783	Distal Intergenic
chr14	77155160	77156085	Distal Intergenic
chr14	77161017	77162211	Distal Intergenic
chr14	77187048	77187288	Distal Intergenic
chr14	77346635	77347476	Distal Intergenic
chr14	77351147	77351750	Distal Intergenic
chr14	77436837	77438174	Distal Intergenic
chr14	78419993	78420550	Distal Intergenic
chr14	79335184	79335785	Intron
chr14	79734015	79734745	Intron
chr14	79735032	79736588	Intron
chr14	79789424	79790038	Intron
chr14	79881576	79882009	Intron
chr14	79903192	79903832	Intron
chr14	79903918	79904163	Intron
chr14	79915085	79916774	Intron
chr14	79916855	79917304	Intron
chr14	79926259	79928727	Intron
chr14	79933353	79933634	Exon
chr14	79934108	79934484	Intron
chr14	79934548	79935341	Intron
chr14	79949069	79950060	Intron
chr14	79951674	79952094	Intron
chr14	79986839	79987038	Intron
chr14	80883874	80884247	Intron
chr14	80884337	80884539	Intron
chr14	82275560	82276155	Distal Intergenic
chr14	87451321	87451533	Distal Intergenic
chr14	87540056	87540902	Distal Intergenic
chr14	90622426	90623002	Intron
chr14	92248528	92249078	3' UTR
chr14	98870738	98871985	Distal Intergenic
chr14	98881886	98882717	Distal Intergenic
chr14	98882835	98883087	Distal Intergenic
chr14	99436909	99437294	Distal Intergenic
chr14	99573599	99574198	Distal Intergenic
chr14	99698834	99699275	Intron

chr14	99699403	99700109	Intron
chr14	99700211	99700763	Intron
chr14	99723924	99724524	Exon
chr14	99772877	99773076	Distal Intergenic
chr14	99848480	99849324	Distal Intergenic
chr14	99865036	99865858	Exon
chr14	99947641	99947943	Promoter
chr14	99947980	99948217	Promoter
chr14	99977738	99978547	3' UTR
chr14	99978713	99978999	3' UTR
chr14	99979168	99979674	3' UTR
chr14	100023630	100024969	Intron
chr14	100030171	100032594	Intron
chr14	101274713	101274962	Distal Intergenic
chr14	102269828	102270032	Intron
chr14	105666813	105668151	Distal Intergenic
chr14	105727371	105728688	Intron
chr14	105740730	105740988	Intron
chr14	105768720	105769739	Intron
chr14	105776672	105777057	Intron
chr14	106561625	106561948	Exon
chr14	110925971	110927438	Distal Intergenic
chr14	111103084	111103354	Distal Intergenic
chr14	111103430	111103823	Distal Intergenic
chr14	111569764	111570171	Distal Intergenic
chr14	111570211	111570575	Distal Intergenic
chr14	116700646	116700881	Distal Intergenic
chr14	117328065	117328285	Distal Intergenic
chr14	118785196	118785576	Distal Intergenic
chr14	118792579	118793628	Distal Intergenic
chr14	118901579	118902351	Distal Intergenic
chr14	118965013	118965741	Distal Intergenic
chr14	118965862	118966175	Distal Intergenic
chr14	120675461	120677239	Distal Intergenic
chr14	120677709	120677929	Distal Intergenic
chr14	120691051	120692433	Distal Intergenic
chr14	120705512	120705871	Distal Intergenic
chr14	120705977	120706213	Distal Intergenic
chr14	120706394	120707696	Distal Intergenic
chr14	120717420	120718041	Distal Intergenic
chr14	120720114	120721490	Distal Intergenic
chr14	120721563	120723091	Distal Intergenic
chr14	120723324	120723536	Distal Intergenic
chr14	120740413	120742248	Distal Intergenic

chr14	120771865	120773026	Distal Intergenic
chr14	120773113	120773346	Distal Intergenic
chr14	121245121	121245852	Distal Intergenic
chr14	121324874	121325085	Distal Intergenic
chr14	121755664	121756148	Distal Intergenic
chr14	122010600	122011536	Distal Intergenic
chr14	122108996	122109394	Distal Intergenic
chr14	122109528	122109730	Distal Intergenic
chr14	122109914	122110911	Distal Intergenic
chr14	122111373	122112121	Distal Intergenic
chr14	122112337	122113393	Distal Intergenic
chr14	122197369	122197603	Distal Intergenic
chr14	122221251	122221897	Distal Intergenic
chr14	122221952	122222567	Distal Intergenic
chr14	122223885	122224094	Distal Intergenic
chr14	122224367	122225557	Distal Intergenic
chr14	122243608	122243920	Distal Intergenic
chr14	122504393	122504605	Distal Intergenic
chr14	122505726	122505974	Distal Intergenic
chr14	122582902	122583378	Distal Intergenic
chr14	124061419	124061827	Distal Intergenic
chr14	124061896	124062148	Distal Intergenic
chr15	3087090	3087417	Distal Intergenic
chr15	3929011	3929216	Distal Intergenic
chr15	3929522	3929945	Distal Intergenic
chr15	3950196	3950436	Distal Intergenic
chr15	3972403	3972745	Distal Intergenic
chr15	3973379	3974715	Distal Intergenic
chr15	4117894	4118160	Distal Intergenic
chr15	4118227	4118473	Distal Intergenic
chr15	4118620	4119850	Distal Intergenic
chr15	5065587	5066318	Distal Intergenic
chr15	5066619	5067480	Distal Intergenic
chr15	5067672	5067925	Distal Intergenic
chr15	5135488	5135692	Distal Intergenic
chr15	5144510	5144949	Distal Intergenic
chr15	5145874	5146706	Distal Intergenic
chr15	5148742	5148947	Distal Intergenic
chr15	5408838	5409850	Distal Intergenic
chr15	5409897	5410366	Distal Intergenic
chr15	5411183	5412700	Distal Intergenic
chr15	5487213	5487528	Distal Intergenic
chr15	5489167	5489779	Distal Intergenic
chr15	6009242	6009441	Distal Intergenic

chr15	6183507	6184174	Distal Intergenic
chr15	6263356	6264394	Distal Intergenic
chr15	6334023	6334869	Distal Intergenic
chr15	6442636	6442872	Distal Intergenic
chr15	6514990	6515470	Distal Intergenic
chr15	6524164	6525193	Distal Intergenic
chr15	6675568	6675857	Distal Intergenic
chr15	6675997	6676411	Distal Intergenic
chr15	6823729	6824003	Distal Intergenic
chr15	6824146	6825069	Distal Intergenic
chr15	6876925	6878613	Distal Intergenic
chr15	6901022	6901223	Distal Intergenic
chr15	6945574	6945784	Distal Intergenic
chr15	7547043	7547253	Distal Intergenic
chr15	7547445	7548196	Distal Intergenic
chr15	7548248	7548601	Distal Intergenic
chr15	7628687	7629504	Distal Intergenic
chr15	7630648	7632008	Distal Intergenic
chr15	7641263	7641477	Distal Intergenic
chr15	7642738	7644116	Distal Intergenic
chr15	7644179	7644450	Distal Intergenic
chr15	7666006	7666355	Distal Intergenic
chr15	7700232	7700563	Distal Intergenic
chr15	7761124	7761769	Distal Intergenic
chr15	7788419	7789496	Distal Intergenic
chr15	7812413	7813505	Distal Intergenic
chr15	7827649	7828038	Distal Intergenic
chr15	7881641	7882453	Distal Intergenic
chr15	7912063	7912299	Distal Intergenic
chr15	7912408	7913904	Distal Intergenic
chr15	8059287	8059858	Distal Intergenic
chr15	8139651	8140665	Distal Intergenic
chr15	8140746	8140945	Distal Intergenic
chr15	8391916	8392182	Distal Intergenic
chr15	8392375	8394455	Distal Intergenic
chr15	8395516	8396619	Distal Intergenic
chr15	8396666	8397063	Distal Intergenic
chr15	8617802	8618048	Distal Intergenic
chr15	8618161	8618479	Distal Intergenic
chr15	8735526	8735725	Distal Intergenic
chr15	8944677	8945315	Distal Intergenic
chr15	8945415	8945958	Distal Intergenic
chr15	9165080	9166078	Distal Intergenic
chr15	10414572	10415692	Distal Intergenic

chr15	10415743	10416025	Distal Intergenic
chr15	10598641	10598867	Distal Intergenic
chr15	10600107	10600719	Distal Intergenic
chr15	10615165	10615786	Distal Intergenic
chr15	10620623	10620904	Distal Intergenic
chr15	10620941	10621229	Distal Intergenic
chr15	10747733	10748996	Distal Intergenic
chr15	10752646	10753482	Distal Intergenic
chr15	10794056	10795410	Distal Intergenic
chr15	10940914	10941660	Distal Intergenic
chr15	10947660	10947913	Distal Intergenic
chr15	10948945	10949181	Distal Intergenic
chr15	11013880	11015305	Distal Intergenic
chr15	11088467	11089460	Distal Intergenic
chr15	11383561	11385531	Distal Intergenic
chr15	11386010	11386397	Distal Intergenic
chr15	11386906	11387842	Distal Intergenic
chr15	11626142	11626870	Distal Intergenic
chr15	11629558	11630106	Distal Intergenic
chr15	11925879	11926099	Distal Intergenic
chr15	12047105	12047505	Distal Intergenic
chr15	12047653	12048028	Distal Intergenic
chr15	12048400	12048619	Distal Intergenic
chr15	12063523	12063799	Distal Intergenic
chr15	12064060	12064893	Distal Intergenic
chr15	12065118	12065352	Distal Intergenic
chr15	12134112	12134629	Distal Intergenic
chr15	12151322	12152349	Distal Intergenic
chr15	12251846	12252162	Distal Intergenic
chr15	12252230	12252490	Distal Intergenic
chr15	12503214	12503771	Distal Intergenic
chr15	13296138	13296768	Distal Intergenic
chr15	13297063	13298763	Distal Intergenic
chr15	19551897	19552683	Distal Intergenic
chr15	20365957	20366231	Distal Intergenic
chr15	22662430	22663467	Intron
chr15	25278370	25278746	3' UTR
chr15	25298586	25299797	Promoter
chr15	25303210	25303873	Promoter
chr15	25325863	25327500	Promoter
chr15	25342086	25342303	Promoter
chr15	25342389	25342647	Promoter
chr15	25343123	25343352	Promoter
chr15	25420641	25421175	Promoter

chr15	25424246	25424878	Promoter
chr15	25552294	25555604	Intron
chr15	25555953	25556195	Intron
chr15	25567378	25567612	Intron
chr15	25567676	25568334	Intron
chr15	25581935	25583574	3' UTR
chr15	25792588	25792823	Distal Intergenic
chr15	25914061	25914281	Distal Intergenic
chr15	26283178	26284006	Intron
chr15	26304032	26304878	Distal Intergenic
chr15	27347969	27348216	Intron
chr15	27348326	27349217	Intron
chr15	27563636	27564468	Intron
chr15	27731668	27733099	Intron
chr15	27853121	27853525	Distal Intergenic
chr15	27853833	27854234	Distal Intergenic
chr15	27855192	27857523	Distal Intergenic
chr15	27897666	27898761	Distal Intergenic
chr15	27913540	27913833	Distal Intergenic
chr15	27914034	27914509	Distal Intergenic
chr15	27921647	27923989	Distal Intergenic
chr15	27924071	27924432	Distal Intergenic
chr15	27926761	27928220	Distal Intergenic
chr15	27933908	27935153	Distal Intergenic
chr15	27954821	27955115	Distal Intergenic
chr15	27989399	27990146	Distal Intergenic
chr15	28010575	28010863	Intron
chr15	28011176	28011769	Intron
chr15	28061919	28063861	Intron
chr15	28088681	28089062	Intron
chr15	28109503	28110017	Intron
chr15	28110135	28110398	Intron
chr15	29329929	29330182	Intron
chr15	30275054	30276678	Distal Intergenic
chr15	30312870	30313328	Distal Intergenic
chr15	30329452	30331221	Distal Intergenic
chr15	31021871	31022305	Intron
chr15	31114499	31114988	5' UTR
chr15	31116446	31117861	Exon
chr15	31117907	31118779	Intron
chr15	31152239	31152495	Distal Intergenic
chr15	31153725	31153971	Distal Intergenic
chr15	31154901	31155167	Distal Intergenic
chr15	31227433	31227895	Intron

chr15	31241387	31241779	Promoter
chr15	31241834	31242172	Promoter
chr15	31261697	31262130	Intron
chr15	31530883	31531242	Distal Intergenic
chr15	31531425	31532049	Distal Intergenic
chr15	32171099	32171423	Distal Intergenic
chr15	32249931	32251526	Distal Intergenic
chr15	32251610	32253225	Distal Intergenic
chr15	32310299	32310708	Distal Intergenic
chr15	32331436	32332020	Intron
chr15	32332096	32332327	Intron
chr15	32846327	32846844	Intron
chr15	32850068	32850460	Intron
chr15	32850592	32851146	Intron
chr15	32929749	32930162	3' UTR
chr15	33573368	33573876	Distal Intergenic
chr15	33574074	33574385	Distal Intergenic
chr15	33836334	33837645	Intron
chr15	33845333	33845567	Intron
chr15	33845667	33846247	Intron
chr15	33881184	33882154	Intron
chr15	34012453	34013835	Intron
chr15	34039552	34040598	Exon
chr15	34076253	34077805	Intron
chr15	34168497	34168705	Intron
chr15	34182495	34182857	Intron
chr15	34183883	34184664	Intron
chr15	34262201	34262737	Intron
chr15	34342091	34344109	Intron
chr15	34371533	34373755	Downstream
chr15	34425067	34425473	Distal Intergenic
chr15	34608068	34608269	Intron
chr15	34608638	34609176	Intron
chr15	34950187	34950395	Distal Intergenic
chr15	34950482	34951138	Distal Intergenic
chr15	35239424	35239705	Intron
chr15	35248977	35249194	Intron
chr15	35301019	35301594	Distal Intergenic
chr15	35994392	35995896	Intron
chr15	36211849	36212881	Distal Intergenic
chr15	36251992	36253727	Distal Intergenic
chr15	36254110	36254866	Distal Intergenic
chr15	36303754	36304350	Distal Intergenic
chr15	36304469	36304971	Distal Intergenic

chr15	36535946	36537865	Distal Intergenic
chr15	36538021	36538539	Distal Intergenic
chr15	36549258	36549487	Distal Intergenic
chr15	36550946	36551161	Distal Intergenic
chr15	36551382	36551581	Distal Intergenic
chr15	36586016	36587895	Distal Intergenic
chr15	36672438	36673188	Distal Intergenic
chr15	36689182	36690106	Distal Intergenic
chr15	36691304	36693180	Distal Intergenic
chr15	36697279	36697520	Distal Intergenic
chr15	36716367	36716640	Distal Intergenic
chr15	36716925	36719284	Distal Intergenic
chr15	36721278	36722391	Distal Intergenic
chr15	36722513	36723043	Distal Intergenic
chr15	36723118	36723357	Distal Intergenic
chr15	36723886	36724517	Distal Intergenic
chr15	36771709	36774268	Distal Intergenic
chr15	36781784	36782200	Distal Intergenic
chr15	36784442	36786215	Distal Intergenic
chr15	36857027	36858015	Distal Intergenic
chr15	36868095	36868361	Distal Intergenic
chr15	36935995	36936673	5' UTR
chr15	36937250	36937477	5' UTR
chr15	36960812	36961673	Intron
chr15	37089385	37089676	Intron
chr15	37122243	37123519	Distal Intergenic
chr15	37126841	37127491	Distal Intergenic
chr15	37127595	37131760	Distal Intergenic
chr15	37162759	37164975	Intron
chr15	37193515	37193733	Intron
chr15	37193779	37194012	Intron
chr15	37194188	37194884	Intron
chr15	37196145	37196490	Intron
chr15	37196527	37198718	Intron
chr15	37204966	37205365	Intron
chr15	37210164	37212142	Intron
chr15	37235445	37235699	Intron
chr15	37242886	37243092	Intron
chr15	37243180	37243471	Intron
chr15	37406860	37407090	Distal Intergenic
chr15	37425514	37426176	Distal Intergenic
chr15	37536802	37537113	Distal Intergenic
chr15	37537183	37537402	Distal Intergenic
chr15	37890643	37890851	Distal Intergenic

chr15	38106028	38106334	Distal Intergenic
chr15	38253902	38254110	Intron
chr15	38256206	38256517	Intron
chr15	38314858	38315216	Intron
chr15	38320678	38322454	Intron
chr15	38381977	38382353	Distal Intergenic
chr15	38382393	38385288	Distal Intergenic
chr15	38385902	38387998	Distal Intergenic
chr15	38388118	38388389	Distal Intergenic
chr15	38399968	38400406	Distal Intergenic
chr15	38447633	38447947	Intron
chr15	38502086	38502727	Intron
chr15	38515213	38515632	Intron
chr15	38620630	38621916	Intron
chr15	38639027	38640948	Intron
chr15	38651427	38652152	Distal Intergenic
chr15	38655229	38656185	Distal Intergenic
chr15	38658082	38658291	Distal Intergenic
chr15	38754604	38754809	Intron
chr15	38763459	38764921	Intron
chr15	38781044	38783621	3' UTR
chr15	38787752	38788829	Intron
chr15	38804515	38806039	Exon
chr15	38809724	38811565	Promoter
chr15	38838194	38838881	Intron
chr15	38944444	38944989	Distal Intergenic
chr15	40195266	40197526	Intron
chr15	40197694	40197917	Intron
chr15	40487645	40487875	Intron
chr15	40785370	40788195	Distal Intergenic
chr15	40788320	40790376	Distal Intergenic
chr15	40800911	40802144	Distal Intergenic
chr15	40859403	40861327	Promoter
chr15	41558996	41559269	Intron
chr15	41566694	41568164	Intron
chr15	41573248	41573740	3' UTR
chr15	43113655	43114097	Intron
chr15	43638824	43639127	Intron
chr15	43696894	43697632	3' UTR
chr15	44258426	44258686	Intron
chr15	44258818	44259164	Intron
chr15	44259272	44259478	Intron
chr15	44259969	44260182	Intron
chr15	44530523	44530765	Distal Intergenic

chr15	49237942	49238192	Intron
chr15	50003907	50004106	Distal Intergenic
chr15	50535518	50535970	Intron
chr15	50773979	50774240	Exon
chr15	50774291	50774658	Intron
chr15	50804090	50804764	Intron
chr15	50806315	50806537	Intron
chr15	50806707	50807053	Intron
chr15	51297147	51299415	3' UTR
chr15	51452762	51453140	Distal Intergenic
chr15	51453181	51453559	Distal Intergenic
chr15	51511115	51511836	Promoter
chr15	51513640	51514340	3' UTR
chr15	51562319	51563118	Intron
chr15	51635992	51636257	Intron
chr15	51636486	51636865	Intron
chr15	51638024	51638231	Intron
chr15	51638272	51638602	Intron
chr15	51684091	51684357	Intron
chr15	51684406	51686502	Intron
chr15	51686542	51686763	Intron
chr15	51696354	51697072	3' UTR
chr15	51708924	51709176	Distal Intergenic
chr15	51709390	51709609	Distal Intergenic
chr15	51817206	51818033	Intron
chr15	51821796	51822863	Intron
chr15	51823069	51823268	Intron
chr15	52935054	52936548	Intron
chr15	52944269	52944884	Promoter
chr15	53001693	53003865	Distal Intergenic
chr15	53027823	53029017	Distal Intergenic
chr15	53053981	53055013	Intron
chr15	53089787	53090034	Distal Intergenic
chr15	53090323	53091204	Distal Intergenic
chr15	53218083	53219271	Distal Intergenic
chr15	53222263	53223808	Distal Intergenic
chr15	53298819	53299164	Distal Intergenic
chr15	53507620	53507822	Distal Intergenic
chr15	53520476	53521242	Distal Intergenic
chr15	53521422	53521700	Distal Intergenic
chr15	53728673	53733864	Distal Intergenic
chr15	53764592	53765064	Distal Intergenic
chr15	53837408	53837641	Intron
chr15	53838115	53838325	Intron

chr15	54966093	54967306	Distal Intergenic
chr15	55017850	55018912	Distal Intergenic
chr15	55147063	55147491	Distal Intergenic
chr15	55165085	55165373	Distal Intergenic
chr15	55388537	55389353	Distal Intergenic
chr15	55413101	55413852	Distal Intergenic
chr15	55658147	55658364	Intron
chr15	55849040	55849244	Intron
chr15	56385534	56385733	3' UTR
chr15	56517030	56517469	Intron
chr15	56552831	56553327	Distal Intergenic
chr15	56971848	56972047	Intron
chr15	57399312	57399723	Intron
chr15	57399795	57400220	Intron
chr15	57443753	57444028	Intron
chr15	57444069	57444412	Intron
chr15	57447674	57449829	Intron
chr15	57477519	57478452	Intron
chr15	57479792	57480200	Intron
chr15	57480299	57480535	Intron
chr15	57481262	57483138	Intron
chr15	57485842	57486461	Intron
chr15	57491194	57492304	Intron
chr15	57493798	57494031	Intron
chr15	57592998	57593197	Promoter
chr15	57593294	57593769	Promoter
chr15	57615801	57616903	Distal Intergenic
chr15	57722986	57723658	Intron
chr15	57738214	57739172	Intron
chr15	57807945	57808190	Intron
chr15	57849282	57849883	Distal Intergenic
chr15	57995948	57997115	Intron
chr15	58025947	58026146	Distal Intergenic
chr15	58087602	58088169	Distal Intergenic
chr15	58088390	58089563	Distal Intergenic
chr15	58090051	58091342	Distal Intergenic
chr15	58092053	58092836	Distal Intergenic
chr15	58132484	58132762	Distal Intergenic
chr15	58133192	58133830	Distal Intergenic
chr15	58156716	58157785	Distal Intergenic
chr15	58163034	58163503	Distal Intergenic
chr15	58189451	58190293	Distal Intergenic
chr15	58194936	58195711	Distal Intergenic
chr15	58198849	58199481	Distal Intergenic

chr15	58209810	58213552	Distal Intergenic
chr15	58213645	58214510	Distal Intergenic
chr15	58214724	58214925	Distal Intergenic
chr15	58244301	58244963	Downstream
chr15	58248282	58248506	Intron
chr15	58648681	58648880	Distal Intergenic
chr15	58649732	58650882	Distal Intergenic
chr15	58745643	58745889	Intron
chr15	58758993	58760085	Intron
chr15	58763872	58765809	Intron
chr15	58829300	58829709	Intron
chr15	58912127	58912551	Intron
chr15	58922117	58922639	Intron
chr15	58982932	58983445	3' UTR
chr15	58990015	58990445	Intron
chr15	58990583	58991333	Intron
chr15	58999759	59002090	Intron
chr15	59015794	59015998	Intron
chr15	59016155	59017046	Intron
chr15	59017140	59017408	Intron
chr15	59017463	59017662	Intron
chr15	59017792	59018011	Intron
chr15	59018063	59018304	Intron
chr15	59018352	59018553	Intron
chr15	59029177	59029669	Intron
chr15	59033365	59034508	Intron
chr15	59043618	59044821	Distal Intergenic
chr15	59053934	59054156	Distal Intergenic
chr15	59054237	59056108	Distal Intergenic
chr15	59145561	59146298	Intron
chr15	59146353	59148541	3' UTR
chr15	59217464	59219861	Intron
chr15	59240761	59241350	Distal Intergenic
chr15	59243733	59244018	Distal Intergenic
chr15	59279663	59280715	Promoter
chr15	59309006	59309388	Intron
chr15	59309568	59309833	Intron
chr15	59312268	59313397	Intron
chr15	59381343	59381778	Intron
chr15	59386616	59387859	3' UTR
chr15	59428087	59428798	3' UTR
chr15	59429121	59429862	3' UTR
chr15	59429981	59431673	Exon
chr15	59431727	59432067	Intron

chr15	59557666	59558064	Intron
chr15	59590882	59591361	Intron
chr15	59591526	59592128	Intron
chr15	59654267	59654914	Intron
chr15	59932956	59933325	Intron
chr15	59933559	59935094	Exon
chr15	59935294	59936133	Intron
chr15	59936271	59936630	Intron
chr15	59941889	59944655	5' UTR
chr15	59957837	59958052	Intron
chr15	59958259	59958464	Intron
chr15	60936751	60938016	Intron
chr15	60940470	60942038	Intron
chr15	60946387	60946790	Intron
chr15	60955802	60956093	Intron
chr15	60956374	60957841	Intron
chr15	60958186	60960015	Intron
chr15	60960179	60960777	Intron
chr15	60969810	60971600	Exon
chr15	60989374	60990557	Intron
chr15	60996863	60997567	Intron
chr15	61038422	61040302	Intron
chr15	61072365	61074265	Intron
chr15	61079028	61079227	Intron
chr15	61125383	61127696	Intron
chr15	61128180	61132438	Intron
chr15	61283298	61283534	Intron
chr15	61283690	61287095	Intron
chr15	61338201	61338778	Intron
chr15	61339988	61340466	Intron
chr15	61363242	61366323	Intron
chr15	61366451	61368015	Intron
chr15	61439530	61442234	Intron
chr15	61449929	61451308	Intron
chr15	61479070	61479363	Intron
chr15	61479415	61479632	Intron
chr15	61516806	61517063	Intron
chr15	61517117	61517361	Intron
chr15	61517436	61517791	Intron
chr15	61743552	61744591	Distal Intergenic
chr15	61744998	61745799	Distal Intergenic
chr15	61816465	61816770	Distal Intergenic
chr15	61816811	61817057	Distal Intergenic
chr15	61817336	61818391	Distal Intergenic

chr15	61823158	61823646	Distal Intergenic
chr15	61830607	61830984	Distal Intergenic
chr15	61869918	61870525	Distal Intergenic
chr15	61870808	61871191	Distal Intergenic
chr15	61875089	61875401	Distal Intergenic
chr15	61910314	61911043	Distal Intergenic
chr15	61911138	61912131	Distal Intergenic
chr15	61914747	61915527	Distal Intergenic
chr15	61923450	61924913	Distal Intergenic
chr15	61950408	61950976	Distal Intergenic
chr15	61970357	61971082	Distal Intergenic
chr15	61993215	61994387	Distal Intergenic
chr15	62446414	62446687	Distal Intergenic
chr15	63172171	63172395	Distal Intergenic
chr15	63229413	63229631	Distal Intergenic
chr15	63819909	63821355	5' UTR
chr15	63821425	63821688	Intron
chr15	63828635	63828894	Promoter
chr15	63829050	63829273	Promoter
chr15	63836704	63838982	Intron
chr15	63844903	63845941	5' UTR
chr15	63850178	63851029	5' UTR
chr15	63863434	63863644	Intron
chr15	63864254	63865388	Intron
chr15	63890639	63891077	Intron
chr15	63983834	63984903	Exon
chr15	64016392	64016964	Intron
chr15	64162392	64162591	Distal Intergenic
chr15	64162836	64163035	Distal Intergenic
chr15	64213004	64214031	Intron
chr15	64214533	64215702	Intron
chr15	64308960	64309312	Intron
chr15	65768094	65768346	Intron
chr15	66371933	66375585	Intron
chr15	66675837	66676040	Distal Intergenic
chr15	66720519	66721415	Intron
chr15	66722368	66723059	Intron
chr15	66763148	66764036	Intron
chr15	66978677	66979270	Distal Intergenic
chr15	67126594	67126793	Distal Intergenic
chr15	67612414	67612625	Intron
chr15	67991970	67992176	Intron
chr15	67992278	67992501	Intron
chr15	67992559	67992886	Intron

chr15	67997206	67997800	Intron
chr15	72485475	72486401	Intron
chr15	72489849	72491719	Promoter
chr15	72491806	72492359	5' UTR
chr15	72492508	72493136	5' UTR
chr15	72493330	72493782	Intron
chr15	72498635	72498907	Intron
chr15	72921339	72921694	Distal Intergenic
chr15	72977947	72978823	Promoter
chr15	72980243	72980653	Intron
chr15	72980909	72982096	Intron
chr15	72993182	72993887	Intron
chr15	73014475	73015013	Promoter
chr15	73065039	73066281	Intron
chr15	73066553	73066861	Promoter
chr15	73072904	73074456	Promoter
chr15	73074633	73074916	Promoter
chr15	73075030	73075229	Promoter
chr15	73075578	73075845	Promoter
chr15	73076079	73078140	Promoter
chr15	73098595	73099086	Distal Intergenic
chr15	73253754	73254146	Distal Intergenic
chr15	73579081	73580335	Intron
chr15	74131728	74131948	Distal Intergenic
chr15	74132005	74132217	Distal Intergenic
chr15	74419518	74420317	3' UTR
chr15	74452111	74452310	Distal Intergenic
chr15	74452472	74454404	Distal Intergenic
chr15	74591501	74592381	Intron
chr15	74594584	74597072	Intron
chr15	74614090	74616115	Intron
chr15	74721939	74722767	Intron
chr15	74751356	74752445	5' UTR
chr15	74760776	74764313	Intron
chr15	74764442	74766045	Intron
chr15	74784786	74785977	Distal Intergenic
chr15	74786051	74786370	Distal Intergenic
chr15	74822578	74822829	Distal Intergenic
chr15	74822891	74823093	Distal Intergenic
chr15	74825796	74826910	Distal Intergenic
chr15	74827002	74830195	Distal Intergenic
chr15	74830246	74830894	Distal Intergenic
chr15	74831014	74831256	Distal Intergenic
chr15	74879076	74879532	Intron

chr15	74879589	74881414	Intron
chr15	74893904	74894249	Exon
chr15	75100233	75101719	Distal Intergenic
chr15	75156453	75157626	Intron
chr15	75159217	75161769	Intron
chr15	75200034	75200495	Promoter
chr15	75502351	75502960	Intron
chr15	75627925	75628714	Promoter
chr15	75681341	75681708	Intron
chr15	75753896	75754195	Distal Intergenic
chr15	75759825	75760024	3' UTR
chr15	75819421	75819701	Exon
chr15	75829894	75830991	Intron
chr15	75831039	75831818	Intron
chr15	75837745	75838001	Intron
chr15	75838484	75839923	Intron
chr15	75840065	75840332	Intron
chr15	75843679	75844749	Intron
chr15	75845050	75845272	Intron
chr15	75911699	75912052	Intron
chr15	75987408	75987642	Intron
chr15	75987816	75988017	Intron
chr15	75988130	75989743	Intron
chr15	75989963	75990264	Intron
chr15	76022597	76023300	Exon
chr15	76023422	76024161	Intron
chr15	76024828	76026718	Intron
chr15	76027033	76027241	Intron
chr15	76027999	76028247	Intron
chr15	76028383	76028629	Intron
chr15	76028799	76030316	Promoter
chr15	76034371	76035798	Distal Intergenic
chr15	76037187	76038369	Distal Intergenic
chr15	76149812	76150012	Intron
chr15	76157405	76157649	Intron
chr15	76157862	76158074	Intron
chr15	76291524	76292098	Intron
chr15	76307868	76308286	Distal Intergenic
chr15	76524952	76525866	Intron
chr15	76554648	76554940	Intron
chr15	76707276	76708162	Intron
chr15	76709788	76710115	Intron
chr15	76734570	76734849	Intron
chr15	76779078	76779324	Intron

chr15	76865870	76868433	Exon
chr15	76886190	76886389	Intron
chr15	76889255	76889666	Intron
chr15	76907301	76907701	Intron
chr15	76980170	76985319	Intron
chr15	77022816	77023527	Intron
chr15	77036109	77037108	Intron
chr15	77049969	77050995	Intron
chr15	77227971	77229630	Exon
chr15	77571115	77572173	Intron
chr15	77572228	77572627	Intron
chr15	77629470	77632365	Intron
chr15	77632413	77632641	Intron
chr15	77633010	77633319	Intron
chr15	77633443	77634715	Intron
chr15	77634950	77635187	Intron
chr15	77637121	77637962	Intron
chr15	77645633	77648379	Intron
chr15	77648522	77650109	Intron
chr15	77650701	77654749	Intron
chr15	77654828	77655778	Intron
chr15	77655843	77656120	Intron
chr15	77656214	77656555	Intron
chr15	77658437	77658645	Intron
chr15	77659879	77662175	Intron
chr15	77662304	77665755	Intron
chr15	77665810	77672010	Intron
chr15	77707126	77707599	Intron
chr15	77707635	77707978	Intron
chr15	77718965	77719305	Intron
chr15	77720147	77720500	Intron
chr15	77720614	77720813	Intron
chr15	77743562	77744182	Intron
chr15	77752247	77752851	Intron
chr15	77800870	77801224	Distal Intergenic
chr15	77801316	77801892	Distal Intergenic
chr15	78280722	78280965	Distal Intergenic
chr15	78637845	78638233	Intron
chr15	78638300	78639106	Intron
chr15	78658300	78658527	Distal Intergenic
chr15	78658593	78659548	Distal Intergenic
chr15	78673356	78677006	Distal Intergenic
chr15	78705979	78706185	Distal Intergenic
chr15	78706386	78706637	Distal Intergenic

chr15	78706758	78707589	Distal Intergenic
chr15	78730833	78731877	Promoter
chr15	78927573	78928996	5' UTR
chr15	78936549	78936756	Intron
chr15	78937115	78937367	Intron
chr15	78937425	78939403	Intron
chr15	78939770	78941160	Intron
chr15	79122823	79123805	Distal Intergenic
chr15	79128732	79130992	Distal Intergenic
chr15	79153977	79154316	Distal Intergenic
chr15	79157505	79158634	Distal Intergenic
chr15	79158745	79159044	Distal Intergenic
chr15	79160328	79160783	Distal Intergenic
chr15	79160908	79161139	Distal Intergenic
chr15	79489290	79489553	Intron
chr15	79595572	79595841	Distal Intergenic
chr15	79722760	79723789	Distal Intergenic
chr15	79730020	79730951	Intron
chr15	79884201	79885046	Distal Intergenic
chr15	79891778	79892137	Distal Intergenic
chr15	79892558	79892867	Distal Intergenic
chr15	79893565	79893830	Distal Intergenic
chr15	79906024	79907371	Distal Intergenic
chr15	79909987	79910242	Distal Intergenic
chr15	79910407	79910627	Distal Intergenic
chr15	79911179	79911397	Distal Intergenic
chr15	79911557	79914351	Distal Intergenic
chr15	79963672	79964029	Distal Intergenic
chr15	80084731	80085496	Distal Intergenic
chr15	80085571	80085836	Distal Intergenic
chr15	80085956	80087004	Distal Intergenic
chr15	80087566	80087887	Distal Intergenic
chr15	80088130	80088995	Distal Intergenic
chr15	80180610	80181616	Exon
chr15	80479453	80480289	Distal Intergenic
chr15	80483235	80484725	Distal Intergenic
chr15	80502350	80502624	Distal Intergenic
chr15	80502870	80504361	Distal Intergenic
chr15	80504417	80505095	Distal Intergenic
chr15	80505188	80511288	Distal Intergenic
chr15	80511489	80511778	Distal Intergenic
chr15	80511963	80512170	Distal Intergenic
chr15	80532601	80532800	Distal Intergenic
chr15	80532940	80534103	Distal Intergenic

chr15	80628496	80628958	Intron
chr15	80629028	80629294	Intron
chr15	80629662	80629972	Intron
chr15	80630062	80630261	Intron
chr15	80643051	80643492	Distal Intergenic
chr15	80679647	80680933	Distal Intergenic
chr15	80685764	80686353	Distal Intergenic
chr15	80691044	80691301	Distal Intergenic
chr15	80691583	80691793	Distal Intergenic
chr15	80691897	80692338	Distal Intergenic
chr15	80728994	80729258	Intron
chr15	80872115	80872854	Exon
chr15	80898119	80902561	Distal Intergenic
chr15	80904283	80905198	Distal Intergenic
chr15	80909768	80909967	Distal Intergenic
chr15	80916136	80916564	Distal Intergenic
chr15	80916645	80918522	Distal Intergenic
chr15	80967824	80968239	Distal Intergenic
chr15	80988740	80990831	Intron
chr15	80993931	80999691	Intron
chr15	81021537	81023997	Intron
chr15	81077847	81078550	Intron
chr15	81094897	81095254	Intron
chr15	81183805	81184317	Intron
chr15	81186946	81187878	Exon
chr15	81190629	81191093	Intron
chr15	81230562	81230761	Intron
chr15	81348349	81349603	Distal Intergenic
chr15	81352858	81353146	Distal Intergenic
chr15	81415891	81416541	Intron
chr15	81416649	81417193	Intron
chr15	81559911	81560306	Intron
chr15	81640756	81641290	Intron
chr15	81688791	81690414	Distal Intergenic
chr15	81790281	81790927	Distal Intergenic
chr15	81976398	81977371	Distal Intergenic
chr15	81977926	81979506	Distal Intergenic
chr15	81981919	81982221	Distal Intergenic
chr15	81982588	81983117	Distal Intergenic
chr15	81983158	81983357	Distal Intergenic
chr15	81989655	81989979	Distal Intergenic
chr15	81990193	81990681	Distal Intergenic
chr15	81990881	81991367	Distal Intergenic
chr15	81991403	81991997	Distal Intergenic

chr15	81994693	81995425	Distal Intergenic
chr15	81995668	81997211	Distal Intergenic
chr15	82551838	82552079	Intron
chr15	82552457	82552656	Intron
chr15	82622051	82622415	Exon
chr15	82731708	82732052	Distal Intergenic
chr15	82732225	82732441	Distal Intergenic
chr15	82770647	82771597	Intron
chr15	82972932	82974162	Exon
chr15	82979149	82979396	Intron
chr15	82980041	82980511	Exon
chr15	82987522	82988510	Intron
chr15	82995617	82995844	Intron
chr15	82997432	82997703	Intron
chr15	83122586	83122905	Intron
chr15	83122955	83124221	Intron
chr15	83168598	83168797	Intron
chr15	83168838	83169327	Intron
chr15	83239009	83239289	Intron
chr15	83271231	83272177	Intron
chr15	83297797	83298224	Promoter
chr15	83393098	83393604	Downstream
chr15	83394311	83394705	3' UTR
chr15	83394801	83396630	5' UTR
chr15	83404718	83406337	Intron
chr15	83424389	83424793	Promoter
chr15	84022341	84022540	Distal Intergenic
chr15	84330639	84331233	Intron
chr15	84754636	84755400	Intron
chr15	85062309	85062900	Distal Intergenic
chr15	85063269	85064314	Distal Intergenic
chr15	85064367	85064643	Distal Intergenic
chr15	85112505	85112914	Promoter
chr15	85127572	85129904	Distal Intergenic
chr15	85177357	85177606	Exon
chr15	85177659	85178324	Intron
chr15	85254429	85255243	Intron
chr15	85309262	85309511	Intron
chr15	85384031	85384271	Exon
chr15	85386440	85387056	Intron
chr15	85465246	85465445	Intron
chr15	85475601	85476787	Exon
chr15	85477313	85477582	Intron
chr15	85494839	85501003	Intron

chr15	85502012	85504982	3' UTR
chr15	85505033	85505617	Intron
chr15	85505803	85506228	Intron
chr15	85506506	85507127	Intron
chr15	85507222	85508142	Intron
chr15	85508298	85509793	Intron
chr15	85509850	85510716	Intron
chr15	85510955	85511254	Intron
chr15	85514206	85515725	Intron
chr15	85515802	85516131	Intron
chr15	85516172	85516417	Intron
chr15	85516697	85517621	Intron
chr15	85724623	85724862	Distal Intergenic
chr15	85725076	85725296	Distal Intergenic
chr15	85766317	85766568	Intron
chr15	85796670	85797248	Distal Intergenic
chr15	85800128	85800614	Distal Intergenic
chr15	85800714	85801191	Distal Intergenic
chr15	85821856	85822117	Distal Intergenic
chr15	85822346	85822819	Distal Intergenic
chr15	85834489	85835951	Distal Intergenic
chr15	85838064	85838700	Distal Intergenic
chr15	85839078	85839747	Distal Intergenic
chr15	85846174	85847656	Distal Intergenic
chr15	85865017	85865369	Distal Intergenic
chr15	85924718	85925281	Promoter
chr15	85925328	85925618	Intron
chr15	85942855	85943312	Intron
chr15	85943358	85944152	Intron
chr15	85944229	85944453	Intron
chr15	85997228	85998087	Intron
chr15	86016724	86017048	Intron
chr15	86017286	86017790	Intron
chr15	87677791	87678827	Distal Intergenic
chr15	88563511	88563951	Intron
chr15	89000205	89000472	Downstream
chr15	89005249	89005467	3' UTR
chr15	89005547	89006401	3' UTR
chr15	89007257	89011192	Promoter
chr15	89011278	89011539	Promoter
chr15	89019532	89020409	Exon
chr15	89186604	89187568	Intron
chr15	89198124	89198361	Intron
chr15	89203596	89204134	Distal Intergenic

chr15	89260123	89260339	Distal Intergenic
chr15	90232075	90232965	Intron
chr15	90261944	90263585	Promoter
chr15	90342656	90343778	Exon
chr15	90437428	90437737	Promoter
chr15	90437871	90440619	Promoter
chr15	90446609	90447163	Exon
chr15	90486718	90487664	Distal Intergenic
chr15	90548839	90549741	Intron
chr15	90549802	90550056	Intron
chr15	90746228	90747490	Intron
chr15	90858956	90859642	Distal Intergenic
chr15	90868883	90869159	Distal Intergenic
chr15	90869250	90869450	Distal Intergenic
chr15	91506699	91507304	Downstream
chr15	91533448	91533959	Intron
chr15	92755647	92756817	Distal Intergenic
chr15	92836110	92836315	Distal Intergenic
chr15	92836585	92837000	Distal Intergenic
chr15	93017251	93018763	Intron
chr15	93019012	93019319	Intron
chr15	93020033	93020334	Intron
chr15	93020690	93020921	Intron
chr15	93106109	93106323	Distal Intergenic
chr15	93228370	93228584	Intron
chr15	93564208	93565484	3' UTR
chr15	94403826	94406211	Intron
chr15	94419309	94419545	Intron
chr15	95201303	95201819	Distal Intergenic
chr15	95633477	95636222	Distal Intergenic
chr15	95653500	95654869	Distal Intergenic
chr15	95689556	95689919	Distal Intergenic
chr15	96117981	96118404	Distal Intergenic
chr15	96118811	96119423	Distal Intergenic
chr15	96138855	96139159	Distal Intergenic
chr15	96290169	96290796	Distal Intergenic
chr15	96452091	96452456	Distal Intergenic
chr15	96528310	96528516	Distal Intergenic
chr15	96535038	96535299	Distal Intergenic
chr15	96535398	96535619	Distal Intergenic
chr15	96586457	96586891	Distal Intergenic
chr15	96586950	96587552	Distal Intergenic
chr15	96587597	96587911	Distal Intergenic
chr15	96600211	96601542	Distal Intergenic

chr15	96601622	96602046	Distal Intergenic
chr15	96652464	96653232	Distal Intergenic
chr15	96660073	96661919	Distal Intergenic
chr15	96662357	96662757	Distal Intergenic
chr15	96662804	96663471	Distal Intergenic
chr15	96673154	96673877	Distal Intergenic
chr15	96673925	96676204	Distal Intergenic
chr15	96707735	96708959	Distal Intergenic
chr15	96709009	96709698	Distal Intergenic
chr15	96872568	96872767	Intron
chr15	97574361	97574738	Distal Intergenic
chr15	97574790	97575076	Distal Intergenic
chr15	97586355	97586625	Distal Intergenic
chr15	97603480	97604142	Distal Intergenic
chr15	97604213	97605058	Distal Intergenic
chr15	97605300	97606509	Distal Intergenic
chr15	97654962	97655182	Distal Intergenic
chr15	97662146	97662345	Distal Intergenic
chr15	97667325	97668574	Distal Intergenic
chr15	97701408	97703158	Distal Intergenic
chr15	97705334	97706667	Distal Intergenic
chr15	97811881	97812088	Distal Intergenic
chr15	97812137	97812971	Distal Intergenic
chr15	97922965	97923764	Distal Intergenic
chr15	97923807	97924021	Distal Intergenic
chr15	98049512	98049717	Distal Intergenic
chr15	98049941	98050340	Distal Intergenic
chr15	98356356	98356595	Intron
chr15	98356637	98357720	Intron
chr15	98362895	98364294	Intron
chr15	98397597	98398161	Intron
chr15	98398221	98398725	Intron
chr15	98492442	98492707	Intron
chr15	98492880	98493340	Intron
chr15	98495224	98495429	Intron
chr15	98501333	98501563	Intron
chr15	98503029	98503801	Promoter
chr15	98574710	98575446	Distal Intergenic
chr15	98765656	98765902	Distal Intergenic
chr15	98815282	98816718	Distal Intergenic
chr15	98823944	98824372	Distal Intergenic
chr15	98859753	98860534	Distal Intergenic
chr15	98860790	98861468	Distal Intergenic
chr15	98861704	98864043	Distal Intergenic

chr15	98877245	98877541	Distal Intergenic
chr15	99082463	99082766	Distal Intergenic
chr15	99223396	99224881	Intron
chr15	99226822	99227073	Intron
chr15	99228029	99228568	Intron
chr15	99628238	99628437	Distal Intergenic
chr15	99628601	99628832	Distal Intergenic
chr15	99628908	99629470	Distal Intergenic
chr15	99629608	99630129	Distal Intergenic
chr15	99647728	99648404	Intron
chr15	99648522	99651122	Intron
chr15	99651230	99651849	Intron
chr15	99651953	99652263	Intron
chr15	99803429	99803689	Intron
chr15	99869644	99870072	Intron
chr15	99871561	99871823	Intron
chr15	99872014	99873652	Intron
chr15	99881169	99882126	Intron
chr15	99890325	99891324	Intron
chr15	99912306	99912631	Intron
chr15	99912689	99912954	Intron
chr15	99913339	99914008	Intron
chr15	99917890	99919768	Intron
chr15	99919818	99920623	Intron
chr15	99926986	99927970	3' UTR
chr15	99940210	99940748	Distal Intergenic
chr15	99949411	99950338	Distal Intergenic
chr15	100050771	100051179	Distal Intergenic
chr15	100051273	100052275	Distal Intergenic
chr15	100052322	100052565	Distal Intergenic
chr15	100057754	100057993	Distal Intergenic
chr15	100058819	100059229	Distal Intergenic
chr15	100081494	100082282	Distal Intergenic
chr15	100100069	100101402	Distal Intergenic
chr15	100129646	100129940	Intron
chr15	100155422	100155860	Intron
chr15	100156035	100156548	Intron
chr15	100251833	100252037	Intron
chr15	100550127	100550687	Intron
chr15	100900364	100903895	Distal Intergenic
chr15	101034776	101034982	Intron
chr15	101051159	101051469	Intron
chr15	101159534	101164285	Intron
chr15	101165484	101166117	Intron

chr15	101166362	101169322	Intron
chr15	101169679	101169963	Exon
chr15	101202753	101204070	Distal Intergenic
chr15	101204257	101204481	Distal Intergenic
chr15	101284888	101285094	Distal Intergenic
chr15	101519552	101519893	Intron
chr15	101523088	101524410	Exon
chr15	101524509	101524750	Intron
chr15	101524872	101526927	Intron
chr15	101527341	101528558	Intron
chr15	101535808	101536511	Intron
chr15	101536556	101536811	Intron
chr15	101538228	101538533	Intron
chr15	101541120	101542134	Intron
chr15	101542186	101545119	Intron
chr15	101545293	101546658	Intron
chr15	101546762	101549333	Exon
chr15	101600129	101603161	Exon
chr15	101804383	101807855	Distal Intergenic
chr15	101808061	101808329	Downstream
chr15	101810268	101811234	3' UTR
chr15	101811472	101812276	3' UTR
chr15	101846121	101846461	Intron
chr15	101846505	101847213	Intron
chr15	101876176	101876605	Intron
chr15	101905016	101905254	3' UTR
chr15	102065614	102066240	Distal Intergenic
chr15	102074417	102074806	Distal Intergenic
chr15	102076252	102076767	Distal Intergenic
chr15	102092872	102093563	Distal Intergenic
chr15	102156599	102156820	Distal Intergenic
chr15	102180170	102181194	Intron
chr15	102232600	102233029	Intron
chr15	102235322	102236255	Intron
chr15	102236746	102236957	Intron
chr15	102237387	102237899	Intron
chr15	102348743	102349340	Distal Intergenic
chr15	102632556	102632843	Distal Intergenic
chr15	102666777	102668852	Distal Intergenic
chr15	102668892	102669134	Distal Intergenic
chr15	102669439	102670301	Distal Intergenic
chr15	102958456	102958677	Distal Intergenic
chr15	102961467	102961703	Distal Intergenic
chr15	103045006	103046253	Distal Intergenic

chr15	103051047	103051984	Distal Intergenic
chr15	103070885	103071247	Distal Intergenic
chr15	103176819	103177018	Distal Intergenic
chr16	3743451	3743924	Intron
chr16	3744095	3745092	Intron
chr16	3819577	3820680	Exon
chr16	3884338	3884577	Intron
chr16	4077718	4078188	Intron
chr16	4447705	4448921	Intron
chr16	4464739	4466768	Promoter
chr16	4469205	4470555	Distal Intergenic
chr16	4473017	4473236	Distal Intergenic
chr16	4473297	4473545	Distal Intergenic
chr16	4477039	4479154	Intron
chr16	4558045	4558671	Exon
chr16	4586858	4588121	Promoter
chr16	4624257	4624962	Exon
chr16	4683440	4684025	Intron
chr16	4776982	4778197	Promoter
chr16	4878170	4879814	Intron
chr16	4879889	4880100	Intron
chr16	4880254	4880463	Intron
chr16	4880581	4881454	Intron
chr16	4913110	4913538	Intron
chr16	4959271	4959730	Intron
chr16	4959801	4960103	Intron
chr16	5049343	5050377	Intron
chr16	5135108	5137040	3' UTR
chr16	6640886	6641088	Intron
chr16	7148030	7148357	Intron
chr16	7951032	7951268	Distal Intergenic
chr16	7951354	7951858	Distal Intergenic
chr16	8636810	8638616	Distal Intergenic
chr16	8665971	8668442	Distal Intergenic
chr16	8671158	8671357	Distal Intergenic
chr16	8671538	8671900	Distal Intergenic
chr16	8790596	8791497	Intron
chr16	8811102	8811434	Intron
chr16	8829188	8829692	5' UTR
chr16	8830220	8831703	Intron
chr16	8848561	8849799	Intron
chr16	8849930	8851268	Intron
chr16	8853775	8853977	Intron
chr16	8861986	8863182	Exon

chr16	8864059	8864535	Intron
chr16	9038368	9039446	Intron
chr16	9259238	9259641	Distal Intergenic
chr16	10286399	10287466	Distal Intergenic
chr16	10330716	10331400	Distal Intergenic
chr16	10347328	10347597	Distal Intergenic
chr16	10347740	10348171	Distal Intergenic
chr16	10365519	10367761	Distal Intergenic
chr16	10373317	10375217	Distal Intergenic
chr16	10558465	10558887	Intron
chr16	10640680	10641027	Intron
chr16	10754367	10754708	Intron
chr16	10754904	10755154	Intron
chr16	10782093	10782934	Intron
chr16	10785695	10786829	Intron
chr16	10836861	10837131	Promoter
chr16	10837274	10839607	Promoter
chr16	10857166	10858111	Exon
chr16	10872858	10873188	Intron
chr16	10975579	10977694	Intron
chr16	10991339	10993232	Exon
chr16	11007657	11009664	Exon
chr16	11015813	11017040	Exon
chr16	11017464	11022865	3' UTR
chr16	11049837	11050895	Intron
chr16	11057353	11058324	Intron
chr16	11058395	11059733	Intron
chr16	11062773	11063859	Exon
chr16	11085772	11086071	Intron
chr16	11133831	11134428	Intron
chr16	11134846	11135482	Intron
chr16	11172895	11173262	Intron
chr16	11173391	11173590	Intron
chr16	11173716	11176049	Intron
chr16	11176540	11177018	Intron
chr16	11193011	11194333	Intron
chr16	11202456	11203400	Intron
chr16	11203475	11204406	Intron
chr16	11252836	11254445	Intron
chr16	11254519	11255196	Intron
chr16	11263530	11263762	Intron
chr16	11286169	11286481	Distal Intergenic
chr16	11501093	11502244	Distal Intergenic
chr16	11505884	11506181	Distal Intergenic

chr16	11511395	11511754	Distal Intergenic
chr16	11511895	11512432	Distal Intergenic
chr16	11776332	11776795	Intron
chr16	11778270	11779177	Intron
chr16	11867890	11869767	Exon
chr16	11908596	11909805	Distal Intergenic
chr16	12742072	12743948	Distal Intergenic
chr16	13119107	13119565	Intron
chr16	13159846	13160350	Intron
chr16	13160481	13161716	Intron
chr16	13240160	13241077	Intron
chr16	13323313	13324441	Intron
chr16	13350824	13352141	Distal Intergenic
chr16	13366263	13366481	Distal Intergenic
chr16	13366555	13367218	Distal Intergenic
chr16	13367304	13367792	Distal Intergenic
chr16	13370936	13372654	Distal Intergenic
chr16	13381894	13382125	Distal Intergenic
chr16	13667858	13668229	Distal Intergenic
chr16	13672028	13672234	Distal Intergenic
chr16	13780157	13781331	Distal Intergenic
chr16	13819714	13819982	Distal Intergenic
chr16	13825832	13827397	Distal Intergenic
chr16	13872369	13872568	Distal Intergenic
chr16	13903302	13903613	Distal Intergenic
chr16	13968919	13969175	Distal Intergenic
chr16	14159006	14159296	Distal Intergenic
chr16	14159637	14159924	Distal Intergenic
chr16	14162696	14163322	Distal Intergenic
chr16	14163392	14163705	Distal Intergenic
chr16	14163775	14164028	Distal Intergenic
chr16	14177618	14177897	Intron
chr16	14178153	14179331	Intron
chr16	14316819	14317049	Intron
chr16	14317115	14317332	Intron
chr16	14317581	14318275	Intron
chr16	14319513	14320423	Intron
chr16	14350368	14353871	Exon
chr16	14361196	14362776	Distal Intergenic
chr16	14363177	14363376	Distal Intergenic
chr16	14369465	14370924	Distal Intergenic
chr16	14371532	14373976	Distal Intergenic
chr16	14375755	14378649	Distal Intergenic
chr16	14378786	14381643	Exon

chr16	14416630	14417972	Distal Intergenic
chr16	14704952	14705831	Intron
chr16	14705937	14706408	Intron
chr16	14706603	14707698	Intron
chr16	14709261	14709460	Intron
chr16	14799911	14800821	Distal Intergenic
chr16	14883978	14886574	Distal Intergenic
chr16	14926894	14929215	Promoter
chr16	14929848	14930116	Intron
chr16	14930155	14932269	Exon
chr16	14932315	14934027	Exon
chr16	14935736	14938875	Exon
chr16	14939093	14941651	Exon
chr16	14941786	14942172	Intron
chr16	14942972	14944225	Intron
chr16	14983805	14984678	Intron
chr16	15112344	15116220	Exon
chr16	15157684	15158062	Intron
chr16	15250823	15251222	Distal Intergenic
chr16	15251349	15252883	Distal Intergenic
chr16	15354665	15355425	Distal Intergenic
chr16	15355578	15358351	Distal Intergenic
chr16	15358633	15358937	Distal Intergenic
chr16	15360606	15360981	Distal Intergenic
chr16	15493259	15494213	Intron
chr16	15541759	15542150	Intron
chr16	15546631	15547622	Intron
chr16	15549942	15551170	Intron
chr16	15551617	15551989	Intron
chr16	15594006	15594223	Intron
chr16	15594371	15594864	Intron
chr16	15595296	15595675	Promoter
chr16	15637253	15637452	Intron
chr16	15641057	15641270	Intron
chr16	15684854	15685808	Downstream
chr16	15709936	15710174	Intron
chr16	15984499	15984956	Distal Intergenic
chr16	15991333	15991756	Distal Intergenic
chr16	15991964	15992942	Distal Intergenic
chr16	16060444	16061214	Intron
chr16	16082558	16083612	Intron
chr16	16215932	16217811	Exon
chr16	16221806	16222289	Intron
chr16	16223852	16225297	Intron

chr16	16302667	16304145	Exon
chr16	16359109	16359460	Intron
chr16	16865950	16867032	Distal Intergenic
chr16	16870385	16870777	Distal Intergenic
chr16	16870966	16871439	Distal Intergenic
chr16	16911891	16913178	Distal Intergenic
chr16	16983505	16983901	Distal Intergenic
chr16	16984225	16984653	Distal Intergenic
chr16	16987804	16989604	Distal Intergenic
chr16	17024298	17024506	Distal Intergenic
chr16	17111073	17111316	Distal Intergenic
chr16	17167575	17167807	Distal Intergenic
chr16	17170039	17170293	Distal Intergenic
chr16	17190753	17191313	Distal Intergenic
chr16	17200473	17202171	3' UTR
chr16	17249133	17249729	Intron
chr16	17451606	17451806	Intron
chr16	17452074	17453218	Intron
chr16	17453342	17453734	Intron
chr16	17456568	17457808	Intron
chr16	17508611	17509670	Intron
chr16	17722023	17723040	Distal Intergenic
chr16	17733076	17733757	Distal Intergenic
chr16	17865563	17866023	Distal Intergenic
chr16	17866110	17866724	Distal Intergenic
chr16	17876498	17876868	Distal Intergenic
chr16	17891163	17891362	Distal Intergenic
chr16	17922067	17922958	Distal Intergenic
chr16	17923434	17923635	Distal Intergenic
chr16	17928313	17929093	Distal Intergenic
chr16	17938306	17939323	Distal Intergenic
chr16	17949122	17950494	Distal Intergenic
chr16	18182848	18183517	Distal Intergenic
chr16	18194603	18195472	Distal Intergenic
chr16	18247811	18248790	Distal Intergenic
chr16	18249091	18249722	Distal Intergenic
chr16	18249766	18250022	Distal Intergenic
chr16	18338536	18338904	Distal Intergenic
chr16	18364026	18364231	Distal Intergenic
chr16	18376386	18377488	Distal Intergenic
chr16	18394855	18395411	Distal Intergenic
chr16	18416138	18416337	Intron
chr16	18416427	18416735	Intron
chr16	18425764	18426064	Intron

chr16	18426213	18426894	Intron
chr16	18482521	18483351	Distal Intergenic
chr16	18484279	18485760	Exon
chr16	18498457	18498801	Distal Intergenic
chr16	18547810	18548194	Intron
chr16	18687076	18687547	Distal Intergenic
chr16	18811536	18812663	Promoter
chr16	18812711	18812987	Promoter
chr16	18836400	18836888	Intron
chr16	18876885	18877288	Intron
chr16	18877489	18877702	Intron
chr16	18877797	18878368	Exon
chr16	18949847	18950900	Distal Intergenic
chr16	19997566	19997934	Distal Intergenic
chr16	20301491	20301715	Distal Intergenic
chr16	20301826	20302385	Distal Intergenic
chr16	20353789	20354051	Intron
chr16	20354255	20354454	Intron
chr16	20428663	20429531	Exon
chr16	20429580	20430024	Exon
chr16	20438878	20439359	Exon
chr16	20445604	20447183	Intron
chr16	20449738	20450095	Intron
chr16	20476899	20477361	Exon
chr16	20496504	20496797	Intron
chr16	20499263	20499574	Exon
chr16	20510988	20512052	Distal Intergenic
chr16	20517137	20517710	Distal Intergenic
chr16	20535032	20535441	Distal Intergenic
chr16	20535656	20538422	Distal Intergenic
chr16	20547241	20547576	Downstream
chr16	20547665	20548517	3' UTR
chr16	20610833	20611184	Distal Intergenic
chr16	20651740	20652165	Exon
chr16	20673245	20673505	Intron
chr16	20677748	20678275	Intron
chr16	20716880	20717569	Distal Intergenic
chr16	20718053	20718526	Distal Intergenic
chr16	20910961	20911621	Promoter
chr16	20931191	20931398	Intron
chr16	20938187	20940359	Distal Intergenic
chr16	20975219	20976518	Exon
chr16	21138455	21139728	Exon
chr16	21139833	21140667	Intron

chr16	21151247	21152185	Exon
chr16	21163305	21163521	Intron
chr16	21207023	21209189	3' UTR
chr16	21209244	21210061	Intron
chr16	21210142	21210912	Exon
chr16	21211113	21211947	Exon
chr16	21225712	21227311	Distal Intergenic
chr16	21255959	21256221	Intron
chr16	21332814	21333672	Distal Intergenic
chr16	21339301	21339579	Distal Intergenic
chr16	21361243	21361537	Promoter
chr16	21361655	21362049	Promoter
chr16	21381264	21383712	Intron
chr16	21401719	21402766	Distal Intergenic
chr16	21402948	21403719	Distal Intergenic
chr16	21818466	21818888	Intron
chr16	21998120	21998407	Intron
chr16	21998490	21998689	Intron
chr16	22009572	22010019	Intron
chr16	22027758	22028057	Intron
chr16	22077297	22077538	Intron
chr16	22125413	22125710	Intron
chr16	22151561	22151833	Exon
chr16	22152044	22152387	Intron
chr16	22153506	22153705	Intron
chr16	22160888	22161260	Exon
chr16	22161405	22161826	Intron
chr16	22162197	22162627	Intron
chr16	22162728	22162944	Intron
chr16	22163605	22164093	Exon
chr16	22201220	22202121	Distal Intergenic
chr16	22263098	22263389	Intron
chr16	22263660	22263859	Intron
chr16	22264065	22266047	Intron
chr16	22266138	22266971	Intron
chr16	22365655	22366733	Intron
chr16	22920077	22920277	Intron
chr16	22920362	22920625	Intron
chr16	23107218	23107421	Intron
chr16	23107540	23109573	Intron
chr16	23109612	23109916	Intron
chr16	23109985	23110402	Intron
chr16	23110472	23111066	Intron
chr16	23127295	23127586	Intron

chr16	23127709	23128068	Intron
chr16	23128175	23128449	Intron
chr16	23266611	23267049	Distal Intergenic
chr16	23272364	23273006	Distal Intergenic
chr16	23988521	23988738	Intron
chr16	23998219	23998479	Intron
chr16	24012359	24013476	Intron
chr16	24037898	24038146	Intron
chr16	24053239	24054716	Intron
chr16	24055364	24056222	Intron
chr16	24066429	24067655	Intron
chr16	24092781	24093307	Intron
chr16	24236306	24237647	Distal Intergenic
chr16	24364462	24365094	Intron
chr16	24392502	24393735	Distal Intergenic
chr16	24393868	24395863	Distal Intergenic
chr16	24401079	24401393	Distal Intergenic
chr16	24427339	24428545	Distal Intergenic
chr16	24430790	24431731	Distal Intergenic
chr16	24433858	24435706	Distal Intergenic
chr16	24443280	24444026	Distal Intergenic
chr16	24474443	24474702	Distal Intergenic
chr16	24474750	24476437	Distal Intergenic
chr16	24495352	24496011	Distal Intergenic
chr16	24496069	24496372	Distal Intergenic
chr16	24497989	24498191	Distal Intergenic
chr16	24498392	24498951	Distal Intergenic
chr16	24566033	24567316	Promoter
chr16	24678527	24679470	Intron
chr16	24695227	24695712	Distal Intergenic
chr16	24696523	24697561	Distal Intergenic
chr16	24732866	24733925	Distal Intergenic
chr16	24737288	24737987	Distal Intergenic
chr16	24910714	24911287	Intron
chr16	24913430	24914367	Intron
chr16	24948561	24949481	Promoter
chr16	24949524	24949817	Intron
chr16	25011336	25011664	Intron
chr16	25147326	25147942	Intron
chr16	25281993	25283576	Distal Intergenic
chr16	25384801	25385902	Distal Intergenic
chr16	25407445	25407797	Distal Intergenic
chr16	25407908	25408142	Distal Intergenic
chr16	25465600	25466636	Distal Intergenic

chr16	25483399	25484037	Distal Intergenic
chr16	25484077	25486150	Distal Intergenic
chr16	25531693	25532486	Distal Intergenic
chr16	25584864	25585159	Distal Intergenic
chr16	25615514	25616136	Distal Intergenic
chr16	25623727	25626157	Distal Intergenic
chr16	25724917	25726361	Intron
chr16	25777757	25778324	Intron
chr16	25778741	25778963	Intron
chr16	25779032	25779260	Intron
chr16	25779388	25780893	Intron
chr16	25801619	25801852	Intron
chr16	25801900	25806283	Intron
chr16	25806748	25807018	Intron
chr16	25807369	25807568	Intron
chr16	25811863	25812687	Intron
chr16	25832522	25832766	Intron
chr16	25833348	25835832	Intron
chr16	25835927	25837051	Intron
chr16	25837855	25839046	Intron
chr16	25839090	25839305	Intron
chr16	25839523	25839851	Intron
chr16	25841867	25845027	Intron
chr16	25845165	25846231	Intron
chr16	25849422	25849664	Intron
chr16	25851629	25852150	Intron
chr16	25853089	25853386	Intron
chr16	25853436	25854203	Intron
chr16	25859160	25859609	Intron
chr16	25859662	25859943	Intron
chr16	25860430	25861761	Intron
chr16	25898627	25901432	Intron
chr16	25901817	25903319	Intron
chr16	25904361	25906796	Intron
chr16	25912227	25913355	Intron
chr16	25933883	25934763	Intron
chr16	25941762	25943474	Intron
chr16	25946785	25947211	Intron
chr16	25947298	25949210	Intron
chr16	25951427	25952227	Intron
chr16	25953048	25954293	Intron
chr16	26041175	26041988	Intron
chr16	26053771	26054062	Intron
chr16	26088567	26088802	Intron

chr16	26146238	26148150	3' UTR
chr16	26211298	26211746	Distal Intergenic
chr16	26248064	26248315	Distal Intergenic
chr16	26248351	26249031	Distal Intergenic
chr16	26430199	26430989	Distal Intergenic
chr16	26444819	26445064	Distal Intergenic
chr16	27365979	27367069	Intron
chr16	27384929	27385132	Distal Intergenic
chr16	27385267	27386165	Distal Intergenic
chr16	27388751	27389017	Distal Intergenic
chr16	27389097	27390090	Distal Intergenic
chr16	28870234	28871491	Intron
chr16	28871877	28872262	Intron
chr16	28878398	28882665	5' UTR
chr16	28882707	28883635	Exon
chr16	28883848	28886040	3' UTR
chr16	28929325	28929757	Intron
chr16	28930086	28930486	Intron
chr16	29169840	29170041	Intron
chr16	29775431	29776720	Intron
chr16	29814490	29815076	Exon
chr16	29815285	29816616	Promoter
chr16	29822593	29824319	Promoter
chr16	29903142	29903624	Intron
chr16	29903675	29904108	Intron
chr16	29909898	29912280	Promoter
chr16	29915218	29915459	Intron
chr16	29915591	29916301	Exon
chr16	29944049	29945388	Intron
chr16	29945570	29946931	Intron
chr16	29977753	29978376	Intron
chr16	30024428	30025746	Promoter
chr16	30033834	30034787	Promoter
chr16	30043161	30043391	Promoter
chr16	30056815	30058759	Intron
chr16	30058970	30060442	Intron
chr16	30060480	30060820	Intron
chr16	30062458	30063425	Promoter
chr16	30064520	30065148	Promoter
chr16	30065363	30067037	Promoter
chr16	30067198	30067584	Intron
chr16	30068264	30068523	Intron
chr16	30068722	30069311	Intron
chr16	30069373	30070284	Intron

chr16	30070783	30071077	Intron
chr16	30133390	30133694	Promoter
chr16	30187672	30188258	Intron
chr16	30332312	30333328	Intron
chr16	30356295	30358017	Distal Intergenic
chr16	30375135	30376075	Intron
chr16	30387257	30387525	Exon
chr16	30387752	30388379	Exon
chr16	30388968	30389233	Promoter
chr16	30439481	30440583	Promoter
chr16	30442058	30443196	Promoter
chr16	30465146	30465345	Distal Intergenic
chr16	30465478	30466564	Distal Intergenic
chr16	30499733	30500001	Intron
chr16	30500258	30500679	Exon
chr16	30502301	30504566	Intron
chr16	30505305	30505926	Exon
chr16	30510816	30514696	Promoter
chr16	30517660	30519511	Exon
chr16	30524477	30526826	Exon
chr16	30527385	30527589	Intron
chr16	30527636	30529260	Exon
chr16	30572359	30572715	Distal Intergenic
chr16	30587136	30588308	Intron
chr16	30597315	30597916	Promoter
chr16	30771094	30773000	Promoter
chr16	30780591	30780995	Exon
chr16	30835522	30836592	Distal Intergenic
chr16	30955999	30957144	Intron
chr16	31066994	31067233	Distal Intergenic
chr16	31185845	31186079	Distal Intergenic
chr16	31189358	31189672	Distal Intergenic
chr16	31191259	31191599	Promoter
chr16	31191637	31193220	Promoter
chr16	31200896	31201154	Exon
chr16	31233953	31234505	Exon
chr16	31255897	31257079	Distal Intergenic
chr16	31274454	31275837	Intron
chr16	31290381	31291291	Intron
chr16	31291378	31291778	Intron
chr16	31407562	31408697	Exon
chr16	31506450	31507641	Intron
chr16	31526803	31527092	Distal Intergenic
chr16	31592100	31593949	Distal Intergenic

chr16	31598145	31599581	Distal Intergenic
chr16	31948464	31949233	Distal Intergenic
chr16	31979363	31980166	Distal Intergenic
chr16	31994137	31994949	Distal Intergenic
chr16	32002804	32004103	Distal Intergenic
chr16	32005547	32006353	Distal Intergenic
chr16	32008663	32008889	Distal Intergenic
chr16	32010100	32010597	Distal Intergenic
chr16	32045981	32046957	Distal Intergenic
chr16	32058907	32059632	Intron
chr16	32068269	32069812	Intron
chr16	32077393	32079412	Exon
chr16	32231611	32232633	Distal Intergenic
chr16	32260901	32261624	Distal Intergenic
chr16	32277030	32278550	Distal Intergenic
chr16	32332474	32332692	Distal Intergenic
chr16	32332735	32333551	Distal Intergenic
chr16	32640285	32640690	Distal Intergenic
chr16	32641656	32642185	Distal Intergenic
chr16	32644861	32646658	Distal Intergenic
chr16	32657013	32657281	Distal Intergenic
chr16	32671885	32673549	Distal Intergenic
chr16	32675969	32677688	Distal Intergenic
chr16	32821967	32823547	Distal Intergenic
chr16	32858148	32860485	Distal Intergenic
chr16	32863471	32863755	Distal Intergenic
chr16	32863821	32864257	Distal Intergenic
chr16	32867538	32868479	Distal Intergenic
chr16	32914468	32915611	Exon
chr16	32915723	32916165	Intron
chr16	33056253	33058291	Intron
chr16	33061612	33062456	Intron
chr16	33129713	33131760	Intron
chr16	33185655	33186046	Intron
chr16	33379882	33381122	Distal Intergenic
chr16	33381524	33382184	Distal Intergenic
chr16	33382278	33382514	Distal Intergenic
chr16	33394440	33394801	Distal Intergenic
chr16	33400009	33400959	Distal Intergenic
chr16	33410972	33411182	Distal Intergenic
chr16	33411276	33411783	Distal Intergenic
chr16	33667477	33667890	Distal Intergenic
chr16	33830127	33831308	Distal Intergenic
chr16	33832429	33832682	Distal Intergenic

chr16	33832735	33834414	Distal Intergenic
chr16	33836103	33837953	Distal Intergenic
chr16	33852962	33853814	Distal Intergenic
chr16	33863138	33863671	Distal Intergenic
chr16	33868999	33874248	Distal Intergenic
chr16	33877504	33879443	Distal Intergenic
chr16	33883634	33884137	Distal Intergenic
chr16	33932937	33935147	Distal Intergenic
chr16	33940900	33941184	Distal Intergenic
chr16	33941232	33941722	Distal Intergenic
chr16	34602930	34603451	Intron
chr16	34603746	34604087	Intron
chr16	35022999	35023760	Distal Intergenic
chr16	35033804	35034293	Distal Intergenic
chr16	35046679	35048443	Distal Intergenic
chr16	35363606	35363906	Distal Intergenic
chr16	35938995	35939596	Distal Intergenic
chr16	36068834	36072946	Distal Intergenic
chr16	36089737	36091885	Distal Intergenic
chr16	36095388	36096211	Distal Intergenic
chr16	36130520	36131767	Distal Intergenic
chr16	36132829	36136359	Distal Intergenic
chr16	36241301	36242057	Distal Intergenic
chr16	36335286	36335841	Distal Intergenic
chr16	36455046	36455447	Distal Intergenic
chr16	36455513	36455730	Distal Intergenic
chr16	36874624	36875081	Distal Intergenic
chr16	36875212	36875416	Distal Intergenic
chr16	37057168	37058738	Distal Intergenic
chr16	37440478	37441053	Distal Intergenic
chr16	37539302	37539521	Distal Intergenic
chr16	37539566	37540585	Distal Intergenic
chr16	37654043	37654427	Distal Intergenic
chr16	37675579	37676667	Distal Intergenic
chr16	37704708	37705583	Distal Intergenic
chr16	37725588	37726252	Distal Intergenic
chr16	37728497	37729139	Distal Intergenic
chr16	37787794	37791731	Distal Intergenic
chr16	37800667	37802182	Distal Intergenic
chr16	37868704	37869381	Distal Intergenic
chr16	37940588	37940812	Distal Intergenic
chr16	38012478	38012933	Distal Intergenic
chr16	38017103	38018764	Distal Intergenic
chr16	38088003	38088406	Distal Intergenic

chr16	38089149	38089548	Distal Intergenic
chr16	38089690	38091106	Distal Intergenic
chr16	38092097	38092347	Distal Intergenic
chr16	38119290	38119650	Distal Intergenic
chr16	38138571	38138774	Distal Intergenic
chr16	38160092	38161027	Distal Intergenic
chr16	38162001	38162206	Distal Intergenic
chr16	38204783	38205948	Distal Intergenic
chr16	38346885	38347984	Distal Intergenic
chr16	38348093	38348487	Distal Intergenic
chr16	38368383	38368656	Distal Intergenic
chr16	38370341	38372428	Distal Intergenic
chr16	38393797	38394946	Distal Intergenic
chr16	38395011	38395666	Distal Intergenic
chr16	38395791	38397033	Distal Intergenic
chr16	38402755	38404859	Distal Intergenic
chr16	38406282	38407329	Distal Intergenic
chr16	38452467	38453436	Distal Intergenic
chr16	38458093	38460614	Distal Intergenic
chr16	38461110	38461406	Distal Intergenic
chr16	38521959	38522789	Distal Intergenic
chr16	38522848	38523443	Distal Intergenic
chr16	38549872	38550108	Distal Intergenic
chr16	38550204	38550408	Distal Intergenic
chr16	38562642	38562869	Distal Intergenic
chr16	38562909	38563181	Distal Intergenic
chr16	38563342	38563569	Distal Intergenic
chr16	42297722	42298410	Distal Intergenic
chr16	42385575	42386093	Distal Intergenic
chr16	42440181	42441655	Distal Intergenic
chr16	42442662	42443502	Distal Intergenic
chr16	42443770	42443985	Distal Intergenic
chr16	42452467	42452791	Distal Intergenic
chr16	42452858	42456025	Distal Intergenic
chr16	42478876	42481241	Distal Intergenic
chr16	42498898	42500330	Distal Intergenic
chr16	42585565	42587532	Distal Intergenic
chr16	42638622	42639012	Distal Intergenic
chr16	42648356	42653298	Distal Intergenic
chr16	42661570	42663251	Distal Intergenic
chr16	42663293	42663816	Distal Intergenic
chr16	42674951	42675211	Distal Intergenic
chr16	42675663	42675915	Distal Intergenic
chr16	42675967	42678632	Distal Intergenic

chr16	42745872	42746800	Distal Intergenic
chr16	42781433	42782969	Distal Intergenic
chr16	42874900	42875271	Distal Intergenic
chr16	43252708	43253696	Distal Intergenic
chr16	43256971	43257387	Distal Intergenic
chr16	43261171	43261685	Distal Intergenic
chr16	43458405	43460090	Distal Intergenic
chr16	43463801	43466216	Distal Intergenic
chr16	43655011	43655211	Distal Intergenic
chr16	43663510	43665504	Distal Intergenic
chr16	43697532	43700340	Distal Intergenic
chr16	43701361	43701560	Distal Intergenic
chr16	43701697	43702411	Distal Intergenic
chr16	43889046	43890482	Distal Intergenic
chr16	44100910	44103018	Distal Intergenic
chr16	44131489	44133643	Distal Intergenic
chr16	44136987	44137186	Distal Intergenic
chr16	44138455	44138655	Distal Intergenic
chr16	44138836	44140538	Distal Intergenic
chr16	44140800	44141149	Distal Intergenic
chr16	44724408	44725663	Distal Intergenic
chr16	44726017	44726326	Distal Intergenic
chr16	44745409	44745765	Distal Intergenic
chr16	44746210	44746422	Distal Intergenic
chr16	45110567	45110952	Distal Intergenic
chr16	45110994	45111327	Distal Intergenic
chr16	45124168	45125056	Distal Intergenic
chr16	45158851	45159722	Distal Intergenic
chr16	45336023	45336732	Distal Intergenic
chr16	45355970	45357129	Distal Intergenic
chr16	45360686	45361005	Distal Intergenic
chr16	45363072	45363344	Distal Intergenic
chr16	45363386	45363729	Distal Intergenic
chr16	45376949	45380315	Distal Intergenic
chr16	45383862	45384365	Distal Intergenic
chr16	45384683	45384882	Distal Intergenic
chr16	45742604	45743081	Distal Intergenic
chr16	45837555	45839226	Distal Intergenic
chr16	45839579	45839940	Distal Intergenic
chr16	45843150	45845541	Distal Intergenic
chr16	45848759	45849837	Distal Intergenic
chr16	45877593	45877824	Distal Intergenic
chr16	45886546	45887829	Distal Intergenic
chr16	45892531	45892931	Distal Intergenic

chr16	45893006	45893711	Distal Intergenic
chr16	45902990	45904408	Distal Intergenic
chr16	45930337	45931272	Distal Intergenic
chr16	45944761	45947204	Distal Intergenic
chr16	45952514	45952857	Distal Intergenic
chr16	48992857	48995340	Distal Intergenic
chr16	49276340	49276574	Distal Intergenic
chr16	49276629	49277033	Distal Intergenic
chr16	49277085	49278339	Distal Intergenic
chr16	49288562	49290327	Distal Intergenic
chr16	49507498	49509735	Distal Intergenic
chr16	49854910	49855651	Promoter
chr16	49856063	49856473	Promoter
chr16	49856513	49859720	Promoter
chr16	49864151	49864437	Intron
chr16	49864473	49864695	Intron
chr16	49904525	49904758	Distal Intergenic
chr16	49905041	49906481	Distal Intergenic
chr16	49914788	49915033	Distal Intergenic
chr16	49915086	49917092	Distal Intergenic
chr16	49923665	49923924	Distal Intergenic
chr16	49935931	49939865	Distal Intergenic
chr16	49951170	49951837	Distal Intergenic
chr16	49951954	49953073	Distal Intergenic
chr16	49969648	49971866	Distal Intergenic
chr16	50009519	50009797	Distal Intergenic
chr16	50188230	50188429	Promoter
chr16	50188659	50188872	Intron
chr16	50288875	50289346	Distal Intergenic
chr16	50289552	50290320	Distal Intergenic
chr16	50302878	50303135	Intron
chr16	50364529	50365131	Intron
chr16	50432272	50432486	Distal Intergenic
chr16	50462183	50462470	Distal Intergenic
chr16	50528356	50529753	Distal Intergenic
chr16	50529797	50530211	Distal Intergenic
chr16	50731995	50732431	Promoter
chr16	50737172	50737839	Intron
chr16	51811060	51811847	Distal Intergenic
chr16	51918246	51918793	Distal Intergenic
chr16	51926746	51927003	Distal Intergenic
chr16	51999737	52000862	Distal Intergenic
chr16	52000906	52001177	Distal Intergenic
chr16	52030935	52031201	Distal Intergenic

chr16	52031343	52031605	Distal Intergenic
chr16	52031671	52032263	Distal Intergenic
chr16	52079080	52080377	Intron
chr16	52134844	52136183	Distal Intergenic
chr16	52179708	52180838	Distal Intergenic
chr16	52196314	52197622	Distal Intergenic
chr16	54041729	54042198	Intron
chr16	54263650	54263902	Distal Intergenic
chr16	55298139	55299594	Distal Intergenic
chr16	55533046	55533396	Intron
chr16	55540218	55541142	3' UTR
chr16	55555121	55556524	Intron
chr16	55556578	55557091	Intron
chr16	55608195	55608559	Exon
chr16	55608616	55609103	Exon
chr16	55636112	55637184	Distal Intergenic
chr16	55770199	55770412	Exon
chr16	55783175	55784896	Exon
chr16	55785079	55785425	Distal Intergenic
chr16	55787198	55789348	Distal Intergenic
chr16	55789593	55790096	Distal Intergenic
chr16	55819808	55822302	Distal Intergenic
chr16	55950916	55951216	Intron
chr16	55966187	55967738	Intron
chr16	55970451	55970783	Intron
chr16	55972448	55972651	Intron
chr16	55973233	55975148	Intron
chr16	56027921	56028131	Distal Intergenic
chr16	56028366	56028595	Distal Intergenic
chr16	56028669	56030192	Distal Intergenic
chr16	56037063	56037716	Distal Intergenic
chr16	56716639	56716972	Promoter
chr16	56717756	56718341	3' UTR
chr16	56852670	56853567	Intron
chr16	56869859	56874438	Exon
chr16	57121806	57122076	Distal Intergenic
chr16	57122130	57122701	Distal Intergenic
chr16	57122934	57123328	Distal Intergenic
chr16	57167535	57167957	Intron
chr16	57226379	57227386	Intron
chr16	57383497	57384332	Distal Intergenic
chr16	57418811	57422885	3' UTR
chr16	57424762	57424987	Distal Intergenic
chr16	57425331	57425533	Distal Intergenic

chr16	57427128	57428849	Distal Intergenic
chr16	57452583	57452886	Distal Intergenic
chr16	57452974	57453469	Distal Intergenic
chr16	57453666	57454975	Distal Intergenic
chr16	57467202	57468211	3' UTR
chr16	57525196	57526480	Distal Intergenic
chr16	57526749	57527766	Distal Intergenic
chr16	57528227	57528612	Distal Intergenic
chr16	57531991	57532926	Distal Intergenic
chr16	57536005	57536273	Distal Intergenic
chr16	57536402	57537398	Distal Intergenic
chr16	57543378	57543664	Downstream
chr16	57545552	57547644	3' UTR
chr16	57548909	57552168	Exon
chr16	57552252	57553680	Exon
chr16	57553787	57554559	Intron
chr16	57554734	57555626	Exon
chr16	57555790	57558699	Intron
chr16	57558972	57560025	Exon
chr16	57560219	57561018	Intron
chr16	57561082	57561457	Intron
chr16	57561877	57567211	5' UTR
chr16	57671156	57671514	Intron
chr16	57671724	57672924	Promoter
chr16	57745862	57746112	Intron
chr16	57746862	57753955	Exon
chr16	57754061	57754330	Intron
chr16	57757417	57757776	Intron
chr16	58352048	58352751	Distal Intergenic
chr16	58409183	58409425	Distal Intergenic
chr16	58553237	58553977	3' UTR
chr16	58586604	58587357	Promoter
chr16	58590898	58592180	Intron
chr16	58669151	58670541	Distal Intergenic
chr16	58670769	58671423	Distal Intergenic
chr16	58671519	58671866	Distal Intergenic
chr16	58701687	58702900	Intron
chr16	58703040	58703526	Intron
chr16	58703662	58704323	Exon
chr16	58728071	58729101	Distal Intergenic
chr16	58745337	58747662	Intron
chr16	59472523	59472722	Distal Intergenic
chr16	59474187	59474592	Distal Intergenic
chr16	59474819	59475623	Distal Intergenic

chr16	59487070	59488343	Distal Intergenic
chr16	59547430	59548776	Distal Intergenic
chr16	59565957	59566997	Distal Intergenic
chr16	59583737	59584047	Distal Intergenic
chr16	59600152	59600560	Distal Intergenic
chr16	62729432	62729705	Distal Intergenic
chr16	62729906	62730747	Distal Intergenic
chr16	62786198	62786507	Distal Intergenic
chr16	62798249	62798617	Distal Intergenic
chr16	62798701	62800237	Distal Intergenic
chr16	62846234	62846784	Distal Intergenic
chr16	64758846	64759250	Distal Intergenic
chr16	64774995	64776252	Distal Intergenic
chr16	64779117	64779763	Distal Intergenic
chr16	64796453	64796739	Distal Intergenic
chr16	64797073	64797409	Distal Intergenic
chr16	64850512	64850926	Distal Intergenic
chr16	64851213	64851616	Distal Intergenic
chr16	64851789	64852041	Distal Intergenic
chr16	64873179	64874534	Distal Intergenic
chr16	65562102	65562471	Intron
chr16	65562707	65562907	Intron
chr16	65814753	65815035	Distal Intergenic
chr16	65815487	65815827	Distal Intergenic
chr16	65816035	65817738	Distal Intergenic
chr16	65817875	65818240	Distal Intergenic
chr16	65818505	65819898	Distal Intergenic
chr16	65822304	65822575	Distal Intergenic
chr16	65868694	65870210	Distal Intergenic
chr16	65870281	65870504	Distal Intergenic
chr16	66921404	66921656	3' UTR
chr16	66921884	66922721	3' UTR
chr16	66922813	66923086	3' UTR
chr16	68205287	68205730	Intron
chr16	69382054	69382281	Intron
chr16	69577016	69578202	Distal Intergenic
chr16	69943788	69944508	3' UTR
chr16	70007071	70007854	Intron
chr16	70307025	70307259	Intron
chr16	70310160	70311752	Exon
chr16	70330722	70331266	Intron
chr16	70492480	70493015	Intron
chr16	70495867	70496649	Intron
chr16	70523813	70525179	Exon

chr16	70525216	70525458	Intron
chr16	71511989	71512236	5' UTR
chr16	72688611	72689272	Distal Intergenic
chr16	73770376	73771582	Distal Intergenic
chr16	73856500	73856761	Distal Intergenic
chr16	74095589	74096270	Distal Intergenic
chr16	74135116	74135433	Distal Intergenic
chr16	74159783	74159987	Distal Intergenic
chr16	74160647	74161208	Distal Intergenic
chr16	74388946	74389330	Intron
chr16	74389451	74390230	Intron
chr16	74400865	74401825	Promoter
chr16	74519284	74519495	Intron
chr16	74526472	74527635	Exon
chr16	74530779	74531042	Intron
chr16	74531923	74532129	Intron
chr16	74609361	74610438	Intron
chr16	74610523	74612836	Intron
chr16	74937293	74937615	Promoter
chr16	75753118	75753326	Distal Intergenic
chr16	75836319	75838109	Distal Intergenic
chr16	75838189	75838388	Distal Intergenic
chr16	76201314	76202356	Distal Intergenic
chr16	76296041	76296556	Distal Intergenic
chr16	76296699	76296913	Distal Intergenic
chr16	76371184	76371695	Intron
chr16	76371745	76372686	Intron
chr16	76373442	76373957	Intron
chr16	76471623	76472194	Intron
chr16	76472251	76472890	Intron
chr16	76499762	76500033	Intron
chr16	76500082	76500323	Intron
chr16	76525698	76526895	Intron
chr16	76539686	76542329	Intron
chr16	76621814	76622174	Distal Intergenic
chr16	76958804	76959052	Distal Intergenic
chr16	76959102	76959337	Distal Intergenic
chr16	76959433	76959735	Distal Intergenic
chr16	77005322	77006073	Distal Intergenic
chr16	77014883	77015112	Distal Intergenic
chr16	77015150	77015423	Distal Intergenic
chr16	77015475	77015689	Distal Intergenic
chr16	78302515	78303199	Intron
chr16	78318143	78318364	Intron

chr16	78423017	78423733	Intron
chr16	78423788	78424102	Intron
chr16	78456271	78457257	Intron
chr16	78473375	78474619	Intron
chr16	78504121	78504469	Intron
chr16	78505005	78505286	Intron
chr16	78531896	78533108	Intron
chr16	78563285	78565141	Intron
chr16	78575069	78576392	Intron
chr16	78576695	78576929	Intron
chr16	78577251	78577739	Intron
chr16	78619010	78619209	Intron
chr16	78619328	78620127	Intron
chr16	78631250	78633227	Intron
chr16	79570329	79571240	Distal Intergenic
chr16	79571309	79571723	Distal Intergenic
chr16	79591026	79591378	Distal Intergenic
chr16	79839528	79840398	Distal Intergenic
chr16	84807329	84807555	Intron
chr16	84834614	84835806	Distal Intergenic
chr16	84835959	84836585	Distal Intergenic
chr16	84838294	84839165	Distal Intergenic
chr16	85134108	85134549	Intron
chr16	85137164	85138546	Intron
chr16	85138607	85138831	Intron
chr16	85141164	85144310	Exon
chr16	85144841	85145363	Promoter
chr16	85147879	85150469	Distal Intergenic
chr16	85152395	85153652	Distal Intergenic
chr16	85158652	85159680	Distal Intergenic
chr16	85171697	85172008	Promoter
chr16	85172105	85172786	Intron
chr16	85172910	85173777	Intron
chr16	85195730	85197799	Distal Intergenic
chr16	85297709	85297921	Distal Intergenic
chr16	85422875	85423308	Distal Intergenic
chr16	85445560	85446864	Distal Intergenic
chr16	85801167	85801523	Distal Intergenic
chr16	85803571	85803797	Distal Intergenic
chr16	85804147	85804350	Distal Intergenic
chr16	86138272	86138539	Distal Intergenic
chr16	86268709	86269277	Distal Intergenic
chr16	86269391	86269645	Distal Intergenic
chr16	86346658	86346987	Distal Intergenic

chr16	86347033	86348932	Distal Intergenic
chr16	86853258	86854154	Distal Intergenic
chr16	87043956	87044398	Distal Intergenic
chr16	87085673	87086423	Distal Intergenic
chr16	87103560	87104141	Distal Intergenic
chr16	87214786	87215360	Intron
chr16	87354369	87354643	Distal Intergenic
chr16	87440634	87441126	3' UTR
chr16	87495545	87495925	Intron
chr16	87599846	87600105	Distal Intergenic
chr16	87625201	87626335	Distal Intergenic
chr16	87656924	87657624	Intron
chr16	87658182	87658484	Intron
chr16	87658539	87659495	Intron
chr16	87659546	87659790	Intron
chr16	87661174	87663940	Intron
chr16	87699246	87700719	Intron
chr16	87712863	87713847	Intron
chr16	87784096	87785275	Promoter
chr16	88478650	88478879	Distal Intergenic
chr16	88505172	88505513	Exon
chr16	89835751	89838114	Exon
chr16	89952677	89953980	Intron
chr16	89954343	89955527	Intron
chr16	89973119	89975827	Exon
chr16	90095004	90096402	Promoter
chr16	90096711	90097048	Promoter
chr16	90097672	90099303	5' UTR
chr16	90126826	90127743	Promoter
chr16	90144867	90145187	Distal Intergenic
chr16	90150842	90151145	Distal Intergenic
chr16	90181650	90181851	Intron
chr16	90210878	90211085	Distal Intergenic
chr16	90284355	90284657	Intron
chr16	90393826	90394045	Distal Intergenic
chr16	90468266	90469551	Distal Intergenic
chr16	90479096	90479321	Distal Intergenic
chr16	90621711	90622947	Distal Intergenic
chr16	90830246	90830520	Distal Intergenic
chr16	90830559	90830917	Distal Intergenic
chr16	90831115	90831438	Distal Intergenic
chr16	90831558	90831916	Distal Intergenic
chr16	90844722	90845864	Distal Intergenic
chr16	90856645	90857785	Distal Intergenic

chr16	90865889	90866226	Distal Intergenic
chr16	90873577	90873778	Distal Intergenic
chr16	90885142	90887076	Distal Intergenic
chr16	91010985	91011354	Distal Intergenic
chr16	91011851	91012074	Distal Intergenic
chr16	91015829	91016320	Distal Intergenic
chr16	91044066	91044287	Distal Intergenic
chr16	91398044	91398946	Distal Intergenic
chr16	91399041	91399246	Distal Intergenic
chr16	91405732	91406070	Distal Intergenic
chr16	91406150	91406639	Distal Intergenic
chr16	91485573	91485772	Distal Intergenic
chr16	91646424	91646739	Distal Intergenic
chr16	91647348	91647582	Distal Intergenic
chr16	91648055	91648368	Distal Intergenic
chr16	91648562	91648888	Distal Intergenic
chr16	92399104	92400399	Distal Intergenic
chr16	92400533	92401431	Distal Intergenic
chr16	92402411	92402838	Distal Intergenic
chr16	92418998	92420374	Distal Intergenic
chr16	92421070	92421269	Distal Intergenic
chr16	92425783	92426325	Distal Intergenic
chr16	92550053	92553814	Distal Intergenic
chr16	92608260	92608861	Distal Intergenic
chr16	92608898	92611561	Distal Intergenic
chr16	92615173	92615372	Distal Intergenic
chr16	92615411	92616680	Distal Intergenic
chr16	92616733	92618187	Distal Intergenic
chr16	92620207	92622757	Distal Intergenic
chr16	92622858	92623213	Distal Intergenic
chr16	92623603	92625285	Distal Intergenic
chr16	92626527	92627756	Distal Intergenic
chr16	92684202	92685933	Distal Intergenic
chr16	92693107	92693321	Distal Intergenic
chr16	92693648	92695020	Distal Intergenic
chr16	92695382	92696958	Distal Intergenic
chr16	92697648	92697953	Distal Intergenic
chr16	92698402	92699017	Distal Intergenic
chr16	92824280	92825100	Distal Intergenic
chr16	92827813	92828054	Distal Intergenic
chr16	92828132	92828700	Distal Intergenic
chr16	92828839	92829247	Distal Intergenic
chr16	92903932	92904287	Distal Intergenic
chr16	92904488	92904796	Distal Intergenic

chr16	92912184	92912730	Distal Intergenic
chr16	93150603	93150812	Distal Intergenic
chr16	93151019	93151218	Distal Intergenic
chr16	93381997	93382196	Distal Intergenic
chr16	93446036	93447300	Distal Intergenic
chr16	93542341	93542677	Distal Intergenic
chr16	93542880	93543215	Distal Intergenic
chr16	94023967	94024396	Distal Intergenic
chr16	94733623	94733861	Distal Intergenic
chr16	94740983	94741262	Distal Intergenic
chr16	94741321	94741596	Distal Intergenic
chr16	94791246	94791668	Distal Intergenic
chr16	94792214	94793344	Distal Intergenic
chr16	94794001	94794200	Distal Intergenic
chr16	94807103	94808800	Distal Intergenic
chr16	94808845	94809126	Distal Intergenic
chr16	94823715	94824860	Distal Intergenic
chr16	94824930	94825168	Distal Intergenic
chr16	94830801	94831278	Distal Intergenic
chr16	95259592	95259846	Distal Intergenic
chr16	95259923	95260787	Distal Intergenic
chr16	95500564	95501150	Distal Intergenic
chr16	95505657	95507750	Distal Intergenic
chr16	95661993	95662256	Distal Intergenic
chr16	95880154	95881589	Distal Intergenic
chr16	95890266	95891045	Distal Intergenic
chr16	95895769	95896744	Distal Intergenic
chr16	95896987	95897585	Distal Intergenic
chr16	95903656	95904055	Distal Intergenic
chr16	95919753	95920587	Distal Intergenic
chr16	95924055	95925627	Distal Intergenic
chr16	95937792	95938141	Distal Intergenic
chr16	95950321	95950976	Distal Intergenic
chr16	95962929	95963696	Distal Intergenic
chr16	95981534	95982195	Distal Intergenic
chr16	95988823	95989140	Distal Intergenic
chr16	96048981	96049638	Distal Intergenic
chr16	96119445	96120633	Distal Intergenic
chr16	96312571	96313920	Distal Intergenic
chr16	96348038	96348447	Distal Intergenic
chr16	97755314	97755551	Distal Intergenic
chr16	97781923	97782942	Distal Intergenic
chr16	97783016	97783231	Distal Intergenic
chr16	97815480	97815713	Distal Intergenic

chr16	97892683	97892928	Distal Intergenic
chr16	97893135	97893404	Distal Intergenic
chr16	97899190	97900056	Distal Intergenic
chr16	97901849	97902300	Distal Intergenic
chr16	97902379	97903409	Distal Intergenic
chr16	97907319	97907518	Distal Intergenic
chr16	97929737	97930572	Distal Intergenic
chr16	97930748	97930966	Distal Intergenic
chr16	97936117	97936331	Distal Intergenic
chr16	97936381	97936783	Distal Intergenic
chr16	97937897	97938133	Distal Intergenic
chr16	97939146	97939660	Distal Intergenic
chr16	97958186	97958507	Distal Intergenic
chr16	97958631	97958910	Distal Intergenic
chr16	97972463	97972667	Distal Intergenic
chr16	97973494	97974415	Distal Intergenic
chr16	97974600	97974926	Distal Intergenic
chr16	97978604	97978849	Distal Intergenic
chr16	97979055	97979704	Distal Intergenic
chr16	97979814	97980063	Distal Intergenic
chr16	97980455	97980717	Distal Intergenic
chr16	97980818	97981865	Distal Intergenic
chr16	97982048	97982415	Distal Intergenic
chr16	97982489	97985008	Distal Intergenic
chr16	97985257	97985512	Distal Intergenic
chr16	97990266	97990632	Distal Intergenic
chr16	98093472	98093734	Distal Intergenic
chr16	98093842	98094632	Distal Intergenic
chr16	98118937	98119370	Distal Intergenic
chr16	98149230	98149556	Distal Intergenic
chr16	98218201	98218661	Distal Intergenic
chr16	98303200	98303600	Distal Intergenic
chr17	3083772	3084140	Distal Intergenic
chr17	3114976	3115175	Distal Intergenic
chr17	3115391	3115823	Distal Intergenic
chr17	3162084	3163563	Distal Intergenic
chr17	3165348	3166265	Distal Intergenic
chr17	3331835	3332254	Distal Intergenic
chr17	3349435	3349859	Exon
chr17	3349981	3350712	Intron
chr17	4993726	4994536	Intron
chr17	4996187	4996552	3' UTR
chr17	4997097	4997325	3' UTR
chr17	5125907	5126227	Intron

chr17	5131649	5132331	Intron
chr17	5132625	5133340	Intron
chr17	5189056	5189955	Intron
chr17	5237774	5238571	Exon
chr17	5238608	5238823	Exon
chr17	5822493	5822695	Intron
chr17	5846367	5846720	Distal Intergenic
chr17	5846810	5847140	Distal Intergenic
chr17	5974712	5976113	Promoter
chr17	5989907	5990134	Intron
chr17	5991162	5991630	Exon
chr17	5991673	5991887	Intron
chr17	5994440	5995188	Intron
chr17	6000862	6001268	Intron
chr17	6001516	6001728	Intron
chr17	6001769	6001984	Intron
chr17	6005879	6006269	Intron
chr17	6008148	6008360	Intron
chr17	6016544	6017175	Intron
chr17	6017687	6018271	Intron
chr17	6107242	6108431	Distal Intergenic
chr17	6117169	6117933	Distal Intergenic
chr17	6127416	6128824	Distal Intergenic
chr17	6140633	6141084	Distal Intergenic
chr17	6141145	6141344	Distal Intergenic
chr17	6153201	6154802	Distal Intergenic
chr17	6172222	6173490	Distal Intergenic
chr17	6174017	6174559	Distal Intergenic
chr17	6201968	6203943	Distal Intergenic
chr17	6955507	6956639	Distal Intergenic
chr17	6959681	6960805	Distal Intergenic
chr17	6961210	6963311	Distal Intergenic
chr17	6982793	6982999	Promoter
chr17	6983443	6984072	Promoter
chr17	6984268	6984547	Promoter
chr17	7012366	7014207	Intron
chr17	7031275	7033320	Distal Intergenic
chr17	7164852	7165072	Intron
chr17	7165589	7165794	Promoter
chr17	8358728	8359090	Intron
chr17	8701421	8702288	Promoter
chr17	8745119	8746016	Intron
chr17	10012992	10015507	Intron
chr17	10247527	10247740	Intron

chr17	10375049	10375889	Intron
chr17	10442293	10442518	Intron
chr17	10510353	10511334	Intron
chr17	11676313	11676827	Intron
chr17	12468433	12468650	Intron
chr17	12468698	12468915	Intron
chr17	12933393	12934148	Distal Intergenic
chr17	12943294	12943900	Distal Intergenic
chr17	12946988	12948520	Distal Intergenic
chr17	12983692	12985071	Distal Intergenic
chr17	13108959	13109786	Distal Intergenic
chr17	13183947	13184162	Distal Intergenic
chr17	13184229	13184494	Distal Intergenic
chr17	13735556	13735884	Distal Intergenic
chr17	13819356	13821274	Distal Intergenic
chr17	13836269	13836525	Distal Intergenic
chr17	13836640	13838003	Distal Intergenic
chr17	13846614	13846885	Distal Intergenic
chr17	13851843	13853323	Distal Intergenic
chr17	13939746	13940089	Intron
chr17	13992388	13992976	Intron
chr17	13993195	13993481	Intron
chr17	13993531	13993926	Intron
chr17	14003807	14004970	Intron
chr17	14057540	14058732	Intron
chr17	14061182	14061891	Intron
chr17	14061986	14062430	Intron
chr17	14062691	14063330	5' UTR
chr17	14309692	14310180	Distal Intergenic
chr17	14324650	14325240	Distal Intergenic
chr17	14330886	14331718	Distal Intergenic
chr17	14473630	14476111	Distal Intergenic
chr17	14476151	14476359	Distal Intergenic
chr17	14476547	14477556	Distal Intergenic
chr17	14561620	14561942	Distal Intergenic
chr17	14623251	14624094	Distal Intergenic
chr17	14650936	14651206	Distal Intergenic
chr17	14651522	14651721	Distal Intergenic
chr17	14890312	14890536	Distal Intergenic
chr17	15110233	15110690	Distal Intergenic
chr17	15110792	15111047	Distal Intergenic
chr17	15114870	15115759	Distal Intergenic
chr17	15116059	15116652	Distal Intergenic
chr17	15126735	15127736	Distal Intergenic

chr17	15127879	15129701	Distal Intergenic
chr17	15141411	15142544	Intron
chr17	15146333	15147083	Intron
chr17	15147276	15147875	Intron
chr17	15157873	15160660	Intron
chr17	15172763	15173842	Distal Intergenic
chr17	15177450	15178506	Distal Intergenic
chr17	15178554	15179248	Distal Intergenic
chr17	15189186	15189468	Distal Intergenic
chr17	15189900	15190340	Distal Intergenic
chr17	15190560	15190872	Distal Intergenic
chr17	15191147	15191824	Distal Intergenic
chr17	15277476	15277877	Distal Intergenic
chr17	15278185	15278386	Distal Intergenic
chr17	15285907	15287200	Distal Intergenic
chr17	15360206	15362028	Intron
chr17	15450206	15450885	5' UTR
chr17	15462562	15465011	Intron
chr17	15493357	15494188	Intron
chr17	15502439	15503705	Promoter
chr17	15512328	15512622	Intron
chr17	15647335	15647864	3' UTR
chr17	15647993	15648274	3' UTR
chr17	15841284	15841526	Distal Intergenic
chr17	15842598	15842917	Distal Intergenic
chr17	15958557	15958805	Intron
chr17	15960701	15961476	Promoter
chr17	15961843	15962227	Promoter
chr17	16205147	16205699	Intron
chr17	16871239	16871438	Intron
chr17	17201248	17201448	Distal Intergenic
chr17	17510870	17511105	Distal Intergenic
chr17	17511267	17511936	Distal Intergenic
chr17	17511984	17512183	Distal Intergenic
chr17	17755403	17756258	Intron
chr17	17760424	17761025	Intron
chr17	17761156	17761659	Exon
chr17	17761722	17762489	Intron
chr17	17928924	17929282	Intron
chr17	17929483	17930225	Exon
chr17	17960446	17963941	Exon
chr17	17963981	17964431	Intron
chr17	17964570	17965617	Exon
chr17	17971259	17972198	3' UTR

chr17	17973396	17973817	Distal Intergenic
chr17	17973856	17976214	Distal Intergenic
chr17	17981979	17983263	Distal Intergenic
chr17	21081623	21083117	Exon
chr17	21145945	21146289	3' UTR
chr17	21146435	21146684	3' UTR
chr17	21586878	21587583	Distal Intergenic
chr17	21609888	21611296	Distal Intergenic
chr17	21612761	21612996	Distal Intergenic
chr17	21615010	21615660	Distal Intergenic
chr17	21652913	21653976	Distal Intergenic
chr17	21654056	21654255	Distal Intergenic
chr17	21668892	21672046	Distal Intergenic
chr17	21672460	21674926	Distal Intergenic
chr17	21694204	21694680	Distal Intergenic
chr17	21882604	21883395	Distal Intergenic
chr17	22099251	22099450	Distal Intergenic
chr17	22099703	22099945	Distal Intergenic
chr17	22362423	22362622	Distal Intergenic
chr17	22451409	22451834	Distal Intergenic
chr17	22498423	22498865	Distal Intergenic
chr17	22876461	22876830	Distal Intergenic
chr17	23680515	23681164	Distal Intergenic
chr17	23683818	23684248	Distal Intergenic
chr17	23687527	23688093	Distal Intergenic
chr17	23794573	23794937	Distal Intergenic
chr17	23794980	23795312	Distal Intergenic
chr17	23810969	23813433	Distal Intergenic
chr17	23813617	23814269	Distal Intergenic
chr17	23817116	23817647	Distal Intergenic
chr17	23831109	23831768	Distal Intergenic
chr17	23831811	23832310	Distal Intergenic
chr17	23863920	23864152	Distal Intergenic
chr17	23940249	23940448	Distal Intergenic
chr17	23940613	23941744	Distal Intergenic
chr17	23965148	23966051	Distal Intergenic
chr17	24061489	24061804	Distal Intergenic
chr17	24061997	24062282	Distal Intergenic
chr17	24116319	24117732	Distal Intergenic
chr17	24134561	24137102	Distal Intergenic
chr17	24137225	24137424	Distal Intergenic
chr17	24152209	24152529	Distal Intergenic
chr17	24152615	24152893	Distal Intergenic
chr17	24171683	24172425	Distal Intergenic

chr17	24210397	24210601	Distal Intergenic
chr17	24283659	24284166	Distal Intergenic
chr17	24284571	24284810	Distal Intergenic
chr17	24289008	24289727	Distal Intergenic
chr17	24305575	24305815	Distal Intergenic
chr17	24306442	24306643	Distal Intergenic
chr17	24357854	24358092	Distal Intergenic
chr17	24380772	24382409	Distal Intergenic
chr17	24387425	24388384	Distal Intergenic
chr17	24388583	24389220	Distal Intergenic
chr17	24551744	24552690	Distal Intergenic
chr17	24615787	24615986	Distal Intergenic
chr17	24664211	24667669	Distal Intergenic
chr17	24667846	24668054	Distal Intergenic
chr17	24804740	24804939	Distal Intergenic
chr17	24805519	24806554	Distal Intergenic
chr17	24807070	24807421	Distal Intergenic
chr17	24808995	24809226	Distal Intergenic
chr17	24832485	24832892	Distal Intergenic
chr17	24856468	24856890	Distal Intergenic
chr17	24857060	24857305	Distal Intergenic
chr17	24888250	24888867	Distal Intergenic
chr17	24954654	24956105	Distal Intergenic
chr17	24976066	24978625	Distal Intergenic
chr17	24985981	24986215	Distal Intergenic
chr17	24986429	24986712	Distal Intergenic
chr17	24986848	24987291	Distal Intergenic
chr17	24992217	24993314	Distal Intergenic
chr17	24993364	24993652	Distal Intergenic
chr17	24996647	24997368	Distal Intergenic
chr17	25031697	25032448	Distal Intergenic
chr17	25032562	25032898	Distal Intergenic
chr17	25033046	25033401	Distal Intergenic
chr17	25066301	25066983	Distal Intergenic
chr17	25085126	25085531	Distal Intergenic
chr17	25087431	25088456	Distal Intergenic
chr17	25359664	25360182	Distal Intergenic
chr17	25408092	25409043	Distal Intergenic
chr17	25409191	25410891	Distal Intergenic
chr17	25893331	25894611	Intron
chr17	26055746	26055979	Distal Intergenic
chr17	26078862	26079190	Distal Intergenic
chr17	26122019	26122342	Intron
chr17	26315376	26315660	Distal Intergenic

chr17	26389619	26390319	Intron
chr17	26390591	26390973	Intron
chr17	26497865	26498431	Intron
chr17	26639413	26640118	Distal Intergenic
chr17	26640337	26641082	Distal Intergenic
chr17	26670638	26671530	Exon
chr17	26674772	26675398	3' UTR
chr17	26686011	26686496	Exon
chr17	26697941	26698394	Promoter
chr17	26741805	26742754	Distal Intergenic
chr17	26742799	26743056	Distal Intergenic
chr17	26757870	26758871	Distal Intergenic
chr17	26767454	26767826	Distal Intergenic
chr17	26768142	26769540	Distal Intergenic
chr17	26813502	26814073	Intron
chr17	27164491	27164698	Intron
chr17	27164780	27165559	Intron
chr17	27175148	27175370	Distal Intergenic
chr17	27175423	27175681	Distal Intergenic
chr17	27175744	27176117	Distal Intergenic
chr17	27180345	27180573	Distal Intergenic
chr17	27185024	27185299	Promoter
chr17	27187353	27188668	Promoter
chr17	27193140	27194105	Distal Intergenic
chr17	27203826	27204160	Downstream
chr17	27206802	27207312	3' UTR
chr17	27210155	27210976	Exon
chr17	27211014	27213424	Exon
chr17	27219239	27220095	Intron
chr17	27220266	27221384	Intron
chr17	27340208	27341034	Distal Intergenic
chr17	27399891	27400227	Downstream
chr17	27724473	27724721	Intron
chr17	27759329	27759584	Intron
chr17	27759788	27759987	Intron
chr17	27771521	27771734	Intron
chr17	27771968	27772210	Intron
chr17	27934143	27938192	5' UTR
chr17	27956256	27956854	3' UTR
chr17	27957104	27957336	3' UTR
chr17	27962684	27963187	Exon
chr17	27963246	27963568	Exon
chr17	28113411	28114002	Intron
chr17	28114167	28114584	Intron

chr17	28159412	28160630	Intron
chr17	28162377	28163390	Intron
chr17	28344398	28345966	Intron
chr17	28360382	28360797	Intron
chr17	28361269	28362362	Exon
chr17	28367601	28367918	Intron
chr17	28368057	28368460	Intron
chr17	28368551	28368780	Intron
chr17	28369667	28374252	Intron
chr17	28374295	28374720	Intron
chr17	28393235	28394520	Intron
chr17	28395478	28395944	Intron
chr17	28395998	28397590	Intron
chr17	28405181	28405464	Exon
chr17	28406011	28407756	Exon
chr17	28450665	28450913	Intron
chr17	28466303	28467410	Intron
chr17	28467526	28467872	Intron
chr17	28468324	28468596	Intron
chr17	28477307	28477873	Intron
chr17	28487459	28487750	Intron
chr17	28487793	28488162	Intron
chr17	28834052	28835573	Intron
chr17	28929981	28930374	Intron
chr17	29062877	29063134	Intron
chr17	29089570	29090012	Intron
chr17	29144464	29144723	Intron
chr17	29144862	29145416	Intron
chr17	29168669	29169034	Intron
chr17	29169315	29170091	Intron
chr17	29170206	29171177	Exon
chr17	29171222	29171492	Intron
chr17	29215432	29216373	Intron
chr17	29218959	29219371	Intron
chr17	29227498	29229562	Exon
chr17	29230032	29232219	3' UTR
chr17	29232758	29234171	Promoter
chr17	29292706	29292973	Distal Intergenic
chr17	29293283	29293482	Distal Intergenic
chr17	29296435	29297826	Promoter
chr17	29317570	29317872	Intron
chr17	29400617	29401146	Distal Intergenic
chr17	29468080	29469158	Intron
chr17	29512515	29513230	Intron

chr17	29513359	29513597	Intron
chr17	29538171	29539385	Intron
chr17	29797761	29798353	Intron
chr17	29861242	29862333	Promoter
chr17	30140983	30141319	Distal Intergenic
chr17	30209561	30209790	Intron
chr17	30209990	30210206	Intron
chr17	30748239	30749283	Distal Intergenic
chr17	31204203	31204402	Promoter
chr17	31210939	31211138	Distal Intergenic
chr17	31412390	31412848	Intron
chr17	31442091	31442532	Intron
chr17	31648628	31648849	Intron
chr17	31794280	31795067	Intron
chr17	31795107	31795496	Intron
chr17	31880832	31881488	Intron
chr17	31883027	31886024	Intron
chr17	31890573	31891147	Intron
chr17	31964911	31965209	Intron
chr17	31967306	31968755	Intron
chr17	31970425	31971650	Intron
chr17	31971699	31972162	Intron
chr17	31978575	31980053	Intron
chr17	32033615	32033882	Intron
chr17	32033979	32034475	Intron
chr17	32140177	32140429	Intron
chr17	32140507	32140723	Intron
chr17	32167492	32168176	Intron
chr17	32228008	32228542	Intron
chr17	32228633	32229125	Intron
chr17	32339600	32340396	Intron
chr17	32420414	32420750	Intron
chr17	32420849	32421092	Intron
chr17	32457275	32457551	Intron
chr17	32457755	32458101	Intron
chr17	32591243	32591503	Distal Intergenic
chr17	32594796	32595289	Distal Intergenic
chr17	32595436	32595635	Distal Intergenic
chr17	32611855	32612106	Promoter
chr17	32637477	32637676	Distal Intergenic
chr17	32923555	32923755	Intron
chr17	32924069	32924609	Intron
chr17	32924840	32925246	Intron
chr17	33226249	33227263	Distal Intergenic

chr17	33766333	33766667	3' UTR
chr17	33821597	33822343	Intron
chr17	33897013	33897304	Distal Intergenic
chr17	33920676	33920875	Intron
chr17	33961480	33961896	Intron
chr17	33961994	33962193	Intron
chr17	34046394	34047336	Intron
chr17	34052863	34053083	3' UTR
chr17	34055804	34057135	Distal Intergenic
chr17	34076989	34077767	Exon
chr17	34088552	34088921	Promoter
chr17	34089632	34089862	Intron
chr17	34090618	34090855	Intron
chr17	34092702	34092977	3' UTR
chr17	34167876	34168140	Intron
chr17	34168393	34168592	Intron
chr17	34256622	34259216	Promoter
chr17	34260049	34260873	Downstream
chr17	34394818	34395284	Intron
chr17	34397334	34398158	Exon
chr17	34405893	34406687	Distal Intergenic
chr17	34741230	34742197	Intron
chr17	34987773	34988006	Distal Intergenic
chr17	35034269	35035317	Distal Intergenic
chr17	35036204	35037153	Distal Intergenic
chr17	35081338	35081563	Distal Intergenic
chr17	35086394	35086634	Distal Intergenic
chr17	35086780	35090019	Distal Intergenic
chr17	35096355	35098865	Distal Intergenic
chr17	35101577	35102216	Distal Intergenic
chr17	35102276	35103220	Distal Intergenic
chr17	35117762	35119053	Distal Intergenic
chr17	35119824	35120023	Distal Intergenic
chr17	35137830	35138389	Distal Intergenic
chr17	35153665	35153970	Distal Intergenic
chr17	35179343	35179583	Distal Intergenic
chr17	35180145	35183186	Distal Intergenic
chr17	35183252	35183558	Distal Intergenic
chr17	35184627	35184863	Distal Intergenic
chr17	35186669	35186920	Distal Intergenic
chr17	35186957	35189241	Distal Intergenic
chr17	35258388	35259132	Distal Intergenic
chr17	35271722	35272359	Distal Intergenic
chr17	35272404	35272781	Distal Intergenic

chr17	35300338	35300681	3' UTR
chr17	35300786	35301766	3' UTR
chr17	35323090	35323312	Intron
chr17	35323682	35323983	Intron
chr17	35335061	35335660	Intron
chr17	35335702	35336603	Intron
chr17	35336640	35337102	Intron
chr17	35337168	35338195	Intron
chr17	35371634	35371958	Intron
chr17	35372237	35372874	Intron
chr17	35378741	35380256	Intron
chr17	35694073	35694447	Intron
chr17	35725551	35725890	Intron
chr17	35725960	35726326	5' UTR
chr17	35727361	35727920	Intron
chr17	35729041	35729412	Intron
chr17	35732549	35732794	Promoter
chr17	35817989	35818188	Intron
chr17	35834418	35835042	Exon
chr17	35835121	35836164	Intron
chr17	35836339	35837389	3' UTR
chr17	35837999	35838296	Distal Intergenic
chr17	35838600	35838819	Distal Intergenic
chr17	35880851	35881460	Intron
chr17	35887020	35887241	Intron
chr17	35887409	35887635	Intron
chr17	35932498	35933185	Intron
chr17	35933883	35934144	Intron
chr17	35944956	35945832	Exon
chr17	35948983	35951081	Intron
chr17	35958068	35958319	Intron
chr17	35959113	35960061	Promoter
chr17	35973941	35974153	3' UTR
chr17	35974209	35974665	3' UTR
chr17	35974839	35976182	Intron
chr17	36002707	36008232	Promoter
chr17	36011399	36011783	Distal Intergenic
chr17	36047140	36047339	3' UTR
chr17	36052504	36052968	Intron
chr17	36053092	36053357	Intron
chr17	36054068	36054489	Intron
chr17	36054574	36056546	Intron
chr17	36106408	36106705	Distal Intergenic
chr17	36131429	36131938	Distal Intergenic

chr17	36137739	36139687	Distal Intergenic
chr17	36160600	36160972	Distal Intergenic
chr17	36179843	36180124	Distal Intergenic
chr17	36408259	36408479	Intron
chr17	36408546	36409201	Intron
chr17	37068907	37069263	Intron
chr17	37088024	37088267	Distal Intergenic
chr17	37088344	37088668	Distal Intergenic
chr17	40939599	40940822	5' UTR
chr17	40943406	40943984	Intron
chr17	41014187	41015920	Distal Intergenic
chr17	41015961	41016198	Distal Intergenic
chr17	41016330	41016613	Distal Intergenic
chr17	41016732	41017943	Distal Intergenic
chr17	41021055	41021434	3' UTR
chr17	41021624	41023059	Distal Intergenic
chr17	42432699	42433196	Exon
chr17	42827471	42829677	3' UTR
chr17	43275246	43275817	Distal Intergenic
chr17	43276015	43276259	Distal Intergenic
chr17	43356492	43356817	Intron
chr17	43356988	43357371	Intron
chr17	43544269	43544484	Intron
chr17	43694878	43695373	Distal Intergenic
chr17	44636723	44638421	Intron
chr17	44638491	44638826	Intron
chr17	44725105	44725330	Intron
chr17	44791829	44792040	Intron
chr17	44826846	44827930	Exon
chr17	44846273	44847639	Exon
chr17	44847792	44848248	Intron
chr17	44904385	44904895	Distal Intergenic
chr17	45202281	45202480	Intron
chr17	45202579	45202874	Intron
chr17	45496570	45496974	Intron
chr17	45709005	45709946	Distal Intergenic
chr17	45709987	45710334	Distal Intergenic
chr17	45821879	45822934	3' UTR
chr17	45831541	45832732	Distal Intergenic
chr17	45883595	45883822	Downstream
chr17	45883873	45884427	Downstream
chr17	45935905	45936108	Distal Intergenic
chr17	46042486	46042729	Distal Intergenic
chr17	46296756	46297101	Intron

chr17	46338856	46339055	Intron
chr17	46384505	46384737	Intron
chr17	46403329	46405508	Intron
chr17	46405866	46406314	Intron
chr17	46406380	46407465	Intron
chr17	46410414	46411872	Intron
chr17	46413978	46414946	Intron
chr17	46414982	46415342	Intron
chr17	46464783	46465188	Intron
chr17	46465279	46465507	Intron
chr17	46690052	46690317	3' UTR
chr17	46692860	46693080	Promoter
chr17	46696639	46696980	Downstream
chr17	46711553	46712077	Distal Intergenic
chr17	46746630	46747458	Distal Intergenic
chr17	46760584	46761998	Distal Intergenic
chr17	46765435	46765825	Distal Intergenic
chr17	46810515	46811230	Distal Intergenic
chr17	46818024	46818409	Distal Intergenic
chr17	46910125	46910480	Intron
chr17	46919395	46920076	Intron
chr17	46920113	46920321	Intron
chr17	46993208	46995409	Exon
chr17	47003646	47004766	3' UTR
chr17	47011111	47011898	Intron
chr17	47012770	47014721	Exon
chr17	47016944	47017370	Intron
chr17	47066054	47066253	Distal Intergenic
chr17	47066570	47066861	Distal Intergenic
chr17	47068796	47070892	Distal Intergenic
chr17	47084639	47085811	Intron
chr17	47146496	47146985	Distal Intergenic
chr17	47202440	47202656	Distal Intergenic
chr17	47278872	47280152	Distal Intergenic
chr17	47280277	47280723	Downstream
chr17	47281328	47282386	Downstream
chr17	47403408	47404937	Intron
chr17	47430697	47432182	Intron
chr17	47433388	47434678	Intron
chr17	47505619	47505970	Distal Intergenic
chr17	47506182	47506402	Distal Intergenic
chr17	47573789	47574554	Intron
chr17	47574793	47575444	Intron
chr17	47575482	47575686	Intron

chr17	47575801	47576217	Intron
chr17	47587418	47588440	Exon
chr17	47621603	47621802	Distal Intergenic
chr17	47748230	47748879	Intron
chr17	47765724	47766130	Distal Intergenic
chr17	47970671	47972065	Distal Intergenic
chr17	47978393	47979752	Distal Intergenic
chr17	48024605	48025806	Distal Intergenic
chr17	48495389	48495772	Distal Intergenic
chr17	48547990	48548189	Intron
chr17	48549450	48550121	Exon
chr17	51317716	51318181	Distal Intergenic
chr17	51318328	51318579	Distal Intergenic
chr17	51792701	51793142	Distal Intergenic
chr17	52895421	52895862	Distal Intergenic
chr17	53617881	53618484	Distal Intergenic
chr17	53618632	53619511	Distal Intergenic
chr17	53622071	53622822	Distal Intergenic
chr17	53828786	53829027	Promoter
chr17	56017520	56017733	Intron
chr17	56018443	56018645	Intron
chr17	56098375	56098765	Distal Intergenic
chr17	56166500	56166775	3' UTR
chr17	56175034	56175233	Distal Intergenic
chr17	56207233	56207662	Distal Intergenic
chr17	56232126	56232331	Promoter
chr17	56405470	56405891	Promoter
chr17	56415962	56416231	Intron
chr17	56416423	56416724	Intron
chr17	56430047	56430637	Promoter
chr17	56499539	56500712	3' UTR
chr17	56604244	56604473	Promoter
chr17	56617513	56617713	Promoter
chr17	56748463	56749385	Intron
chr17	56812200	56812468	Distal Intergenic
chr17	56812721	56813381	Distal Intergenic
chr17	56958399	56958610	Intron
chr17	56958656	56960136	Intron
chr17	56961606	56961928	Intron
chr17	56961996	56962307	Intron
chr17	57016749	57017010	Intron
chr17	57046004	57046838	Intron
chr17	57047015	57047216	Exon
chr17	57150409	57150733	Intron

chr17	57366568	57368259	Distal Intergenic
chr17	57387976	57388201	Distal Intergenic
chr17	57389530	57389807	Distal Intergenic
chr17	59215526	59215728	Intron
chr17	59215805	59216382	Intron
chr17	59216576	59216831	Intron
chr17	59408805	59410020	Intron
chr17	61360777	61360976	Intron
chr17	61420399	61420855	Intron
chr17	61440753	61441215	Intron
chr17	61441408	61441856	Intron
chr17	62635336	62636354	Intron
chr17	62636503	62638578	Intron
chr17	62697898	62698178	Distal Intergenic
chr17	62727962	62728585	Distal Intergenic
chr17	62728669	62728965	Distal Intergenic
chr17	62764038	62764459	Intron
chr17	62854218	62855024	Exon
chr17	62983738	62983960	Distal Intergenic
chr17	62984500	62984759	Distal Intergenic
chr17	62984835	62985060	Distal Intergenic
chr17	63025466	63026629	Intron
chr17	63036447	63036970	Intron
chr17	63037066	63037569	Intron
chr17	63106540	63106739	Distal Intergenic
chr17	63132891	63133258	Promoter
chr17	63133481	63134003	Promoter
chr17	63229264	63229758	Distal Intergenic
chr17	63510982	63511332	Distal Intergenic
chr17	64146243	64146645	Intron
chr17	64147238	64149191	Intron
chr17	64150396	64150640	Intron
chr17	64150738	64151598	Intron
chr17	64298186	64298729	Promoter
chr17	64298780	64299024	Promoter
chr17	64352545	64352979	Intron
chr17	64353015	64353577	Intron
chr17	64949259	64949458	Distal Intergenic
chr17	64994184	64994590	Intron
chr17	65115170	65115566	Intron
chr17	65961801	65962132	Intron
chr17	65963381	65964039	Intron
chr17	66052814	66054104	Distal Intergenic
chr17	66068465	66070601	Distal Intergenic

chr17	66075273	66075541	Distal Intergenic
chr17	66075724	66076667	Distal Intergenic
chr17	66086146	66087990	Distal Intergenic
chr17	66088997	66090918	Distal Intergenic
chr17	66121177	66121408	Intron
chr17	66131965	66132261	Exon
chr17	66133169	66134142	Distal Intergenic
chr17	66163277	66163545	Distal Intergenic
chr17	66182701	66183508	Distal Intergenic
chr17	66254482	66254835	Promoter
chr17	66255003	66256220	Promoter
chr17	66262256	66264067	3' UTR
chr17	66265020	66265282	3' UTR
chr17	66265347	66265991	Exon
chr17	66451030	66451242	Intron
chr17	66451284	66451484	Intron
chr17	66586470	66586829	5' UTR
chr17	66586903	66587263	Intron
chr17	66618514	66618743	Distal Intergenic
chr17	66671426	66671745	Distal Intergenic
chr17	66671819	66673677	Distal Intergenic
chr17	66674029	66674932	Distal Intergenic
chr17	66714554	66714753	Distal Intergenic
chr17	66715301	66715526	Distal Intergenic
chr17	66769200	66769411	Distal Intergenic
chr17	67473303	67473695	Intron
chr17	67524946	67525295	Intron
chr17	67525342	67525557	Intron
chr17	67974541	67974850	Distal Intergenic
chr17	69576803	69577633	Distal Intergenic
chr17	69578521	69579434	Distal Intergenic
chr17	69766254	69766654	Distal Intergenic
chr17	69766762	69767307	Distal Intergenic
chr17	69767590	69767789	Distal Intergenic
chr17	69771162	69771604	Distal Intergenic
chr17	71069471	71069896	Intron
chr17	71075553	71078248	Intron
chr17	71078381	71079677	Intron
chr17	71083434	71084592	Intron
chr17	71085164	71085505	Intron
chr17	71085973	71089006	Promoter
chr17	71090652	71091799	Distal Intergenic
chr17	71095594	71095902	Distal Intergenic
chr17	71095951	71098325	Distal Intergenic

chr17	71102572	71103539	Distal Intergenic
chr17	71104544	71104770	Distal Intergenic
chr17	71104967	71105318	Distal Intergenic
chr17	71185969	71186260	Distal Intergenic
chr17	71186385	71186635	Distal Intergenic
chr17	71196535	71200549	Exon
chr17	71200851	71201095	Intron
chr17	71201509	71202177	Exon
chr17	71202399	71202886	Exon
chr17	71208117	71208439	Intron
chr17	71208481	71208707	Intron
chr17	71237669	71238157	Intron
chr17	71238246	71238448	Exon
chr17	71248565	71249613	Exon
chr17	71257781	71258325	Promoter
chr17	71258865	71259064	Promoter
chr17	71349495	71350000	Intron
chr17	71350038	71351229	Intron
chr17	71351628	71352543	Intron
chr17	71508524	71509164	Intron
chr17	71542215	71542431	Intron
chr17	71542647	71543272	Intron
chr17	71544093	71545151	Intron
chr17	71545235	71545467	Intron
chr17	71876323	71876671	Distal Intergenic
chr17	71965373	71965645	Distal Intergenic
chr17	71966119	71966519	Distal Intergenic
chr17	72001178	72003076	Distal Intergenic
chr17	72091431	72092040	Distal Intergenic
chr17	72092308	72092556	Distal Intergenic
chr17	72129911	72130269	Distal Intergenic
chr17	72347105	72347678	3' UTR
chr17	72491662	72491981	Distal Intergenic
chr17	72648890	72649122	Distal Intergenic
chr17	72688328	72689574	Intron
chr17	72705442	72707102	Intron
chr17	73177033	73178007	Exon
chr17	73306654	73306853	Distal Intergenic
chr17	73306910	73307235	Distal Intergenic
chr17	73307499	73307785	Distal Intergenic
chr17	73307841	73309610	Distal Intergenic
chr17	73315503	73315874	3' UTR
chr17	74167467	74167747	Intron
chr17	74242640	74243875	Distal Intergenic

chr17	74245810	74247454	Distal Intergenic
chr17	74282716	74284877	5' UTR
chr17	74285089	74285290	Intron
chr17	74285726	74287681	5' UTR
chr17	74287718	74287949	Exon
chr17	74290755	74292434	Intron
chr17	74297318	74299966	5' UTR
chr17	74332402	74332650	Intron
chr17	74333070	74333272	Intron
chr17	74338729	74339626	Intron
chr17	74366545	74367393	Distal Intergenic
chr17	74678516	74678755	Intron
chr17	74679245	74679494	Intron
chr17	74682108	74682595	Intron
chr17	74685284	74685670	Promoter
chr17	74687719	74688807	Intron
chr17	74693345	74693712	Intron
chr17	74694027	74694234	Intron
chr17	74695090	74695911	Intron
chr17	74809063	74810027	Distal Intergenic
chr17	74818071	74818976	Distal Intergenic
chr17	74925711	74926026	Intron
chr17	74926083	74927397	Intron
chr17	74928446	74928812	Exon
chr17	74945674	74946078	3' UTR
chr17	75098522	75098949	Intron
chr17	75446392	75446991	Promoter
chr17	75509612	75510665	Distal Intergenic
chr17	75510715	75511182	Distal Intergenic
chr17	75598911	75599327	Distal Intergenic
chr17	75655144	75655359	Distal Intergenic
chr17	75657566	75658530	Distal Intergenic
chr17	75744393	75744683	Distal Intergenic
chr17	75806393	75808017	Distal Intergenic
chr17	76154984	76156658	Promoter
chr17	76336188	76336391	Distal Intergenic
chr17	77993246	77993685	Intron
chr17	77993767	77994021	Intron
chr17	78564567	78564836	Intron
chr17	78564953	78565265	Intron
chr17	78566156	78566383	Intron
chr17	78606031	78606394	Intron
chr17	78606472	78606911	Intron
chr17	78611494	78613332	Intron

chr17	78720252	78720773	Intron
chr17	78720900	78721219	Intron
chr17	78721533	78721991	Intron
chr17	78722161	78722414	Intron
chr17	78787013	78788370	Intron
chr17	78791709	78791915	Intron
chr17	78791974	78792326	Intron
chr17	78801164	78801627	Intron
chr17	78801830	78802061	Intron
chr17	78802254	78802690	Intron
chr17	78802748	78803296	Intron
chr17	79123820	79124231	Intron
chr17	79125966	79126347	Intron
chr17	79126411	79126724	Intron
chr17	79684411	79685247	Exon
chr17	79771524	79772813	3' UTR
chr17	79793911	79794397	Promoter
chr17	79794436	79794791	Distal Intergenic
chr17	79989219	79989617	Promoter
chr17	79998839	79999047	Distal Intergenic
chr17	80145485	80145956	Intron
chr17	80461173	80461421	Distal Intergenic
chr17	81128105	81128310	Distal Intergenic
chr17	81164434	81164772	Distal Intergenic
chr17	81200305	81200523	Distal Intergenic
chr17	81285372	81285814	Distal Intergenic
chr17	81896855	81897713	Distal Intergenic
chr17	83479797	83481035	Distal Intergenic
chr17	83484964	83485830	Distal Intergenic
chr17	83505384	83505874	Distal Intergenic
chr17	83506001	83506495	Distal Intergenic
chr17	83509008	83509550	Distal Intergenic
chr17	83580798	83581642	Distal Intergenic
chr17	84294841	84295260	Distal Intergenic
chr17	84296253	84297730	Distal Intergenic
chr17	84298536	84299547	Distal Intergenic
chr17	84301238	84302447	Distal Intergenic
chr17	84321624	84321823	Distal Intergenic
chr17	84402452	84403745	Distal Intergenic
chr17	84424680	84425360	Distal Intergenic
chr17	84470129	84470574	Distal Intergenic
chr17	84474222	84475033	Distal Intergenic
chr17	84483293	84484028	Distal Intergenic
chr17	84484353	84485822	Distal Intergenic

chr17	84548392	84548892	Distal Intergenic
chr17	84584182	84584388	Distal Intergenic
chr17	84584428	84584922	Distal Intergenic
chr17	84724874	84725937	Distal Intergenic
chr17	84727729	84728592	Distal Intergenic
chr17	85189951	85190150	Distal Intergenic
chr17	85355983	85356215	Distal Intergenic
chr17	85562062	85562409	Distal Intergenic
chr17	85562525	85562868	Distal Intergenic
chr17	86844510	86845124	Distal Intergenic
chr17	86953534	86953757	Distal Intergenic
chr17	86972654	86973313	Distal Intergenic
chr17	86990370	86990719	Distal Intergenic
chr17	86990845	86991413	Distal Intergenic
chr17	87249888	87250173	Distal Intergenic
chr17	87452138	87452358	Distal Intergenic
chr17	87569299	87570760	Distal Intergenic
chr17	87571011	87571210	Distal Intergenic
chr17	87606296	87607068	Distal Intergenic
chr17	87653121	87653426	Distal Intergenic
chr17	87653471	87653918	Distal Intergenic
chr17	87681544	87682140	Distal Intergenic
chr17	87970645	87971033	Distal Intergenic
chr17	88012710	88013543	Distal Intergenic
chr17	88248115	88251704	Distal Intergenic
chr17	88396962	88397555	Distal Intergenic
chr17	88463344	88463623	Distal Intergenic
chr17	88464780	88465101	Distal Intergenic
chr17	88594950	88595196	Distal Intergenic
chr17	88655150	88657032	Distal Intergenic
chr17	88657387	88657993	Distal Intergenic
chr17	88908018	88909249	Distal Intergenic
chr17	88909746	88910728	Distal Intergenic
chr17	88941854	88942904	Distal Intergenic
chr17	89010565	89010907	Distal Intergenic
chr17	89027923	89028649	Distal Intergenic
chr17	89028737	89030283	Distal Intergenic
chr17	89673415	89674735	Distal Intergenic
chr17	89680500	89681087	Distal Intergenic
chr17	89685974	89686385	Distal Intergenic
chr17	91865924	91866599	Distal Intergenic
chr17	95148984	95149252	Distal Intergenic
chr17	95263990	95264221	Distal Intergenic
chr18	3280529	3281275	Distal Intergenic

chr18	3336553	3337346	Distal Intergenic
chr18	3382900	3383171	Distal Intergenic
chr18	3383618	3383868	Distal Intergenic
chr18	3767355	3767897	Intron
chr18	3963550	3964218	Intron
chr18	3964361	3964560	Intron
chr18	4156080	4156666	Intron
chr18	4157785	4158004	Intron
chr18	4310881	4311225	Intron
chr18	4375348	4375780	Intron
chr18	4553190	4553454	Distal Intergenic
chr18	4737639	4738214	Distal Intergenic
chr18	4785245	4786111	Distal Intergenic
chr18	4845472	4846001	Distal Intergenic
chr18	4920938	4921566	Distal Intergenic
chr18	4921613	4922999	Distal Intergenic
chr18	4959548	4960091	Distal Intergenic
chr18	5001977	5002205	Distal Intergenic
chr18	5002269	5002849	Distal Intergenic
chr18	5012816	5013666	Distal Intergenic
chr18	5016223	5017283	Distal Intergenic
chr18	5045821	5047627	Distal Intergenic
chr18	5077531	5079179	Distal Intergenic
chr18	5334601	5334806	Distal Intergenic
chr18	5395835	5396227	Exon
chr18	5396335	5396995	Intron
chr18	5402544	5403340	Intron
chr18	5414377	5415038	Intron
chr18	5945852	5946572	Distal Intergenic
chr18	6119723	6120296	Intron
chr18	6135260	6135571	Intron
chr18	6135718	6135946	Intron
chr18	6136527	6136753	Intron
chr18	6192953	6193185	Intron
chr18	6231100	6232465	Intron
chr18	6235543	6235829	Intron
chr18	6237835	6238855	Exon
chr18	6239440	6239639	Intron
chr18	6240283	6242235	Exon
chr18	6275368	6275964	Intron
chr18	6280816	6282938	Intron
chr18	6285491	6286132	Intron
chr18	6412409	6412651	Intron
chr18	6419580	6419868	Distal Intergenic

chr18	6489567	6489790	Distal Intergenic
chr18	6489981	6490853	Distal Intergenic
chr18	6516269	6516506	Intron
chr18	6726112	6727323	Distal Intergenic
chr18	6727441	6728870	Distal Intergenic
chr18	6760268	6761422	Distal Intergenic
chr18	6765224	6765776	Distal Intergenic
chr18	6959736	6960264	Intron
chr18	7613816	7614394	Intron
chr18	7626068	7626858	Intron
chr18	7649480	7650479	Intron
chr18	7651242	7653054	Intron
chr18	7868177	7868505	Intron
chr18	7868866	7870428	Intron
chr18	8161143	8161470	Intron
chr18	8161507	8161706	Intron
chr18	8693500	8693701	Distal Intergenic
chr18	8694018	8694491	Distal Intergenic
chr18	8719805	8720019	Intron
chr18	8720094	8720586	Exon
chr18	9356764	9356998	Intron
chr18	9357043	9358282	Intron
chr18	9363935	9364194	Intron
chr18	9373971	9375691	Intron
chr18	9375850	9376555	Intron
chr18	9386427	9389931	5' UTR
chr18	9390307	9390517	Intron
chr18	9390652	9391082	Intron
chr18	9394017	9394608	Intron
chr18	9428248	9429804	Distal Intergenic
chr18	9450242	9450487	Distal Intergenic
chr18	9552317	9552757	Intron
chr18	9552864	9553492	Exon
chr18	9734972	9735563	Intron
chr18	9814585	9814823	Intron
chr18	9957767	9958749	3' UTR
chr18	10012995	10013335	Distal Intergenic
chr18	10013479	10014382	Distal Intergenic
chr18	10028934	10029729	Distal Intergenic
chr18	10181953	10182168	Distal Intergenic
chr18	10290062	10290615	Distal Intergenic
chr18	10533087	10533570	5' UTR
chr18	10617897	10618619	Distal Intergenic
chr18	10725588	10725982	Intron

chr18	10726334	10726599	Intron
chr18	10726636	10726867	Intron
chr18	11200321	11200849	Distal Intergenic
chr18	11356174	11356401	Distal Intergenic
chr18	11357063	11357526	Distal Intergenic
chr18	11719601	11721662	Intron
chr18	11742454	11744329	Intron
chr18	11752472	11752734	Promoter
chr18	11754876	11757294	Intron
chr18	11791209	11791716	Intron
chr18	11791774	11793616	Intron
chr18	11815059	11815300	Intron
chr18	11815551	11815807	Intron
chr18	12035306	12035956	Distal Intergenic
chr18	12041588	12042865	Distal Intergenic
chr18	12124687	12125950	Exon
chr18	12136239	12136831	Distal Intergenic
chr18	12136893	12137092	Distal Intergenic
chr18	12225372	12225688	Distal Intergenic
chr18	12286923	12287126	Distal Intergenic
chr18	12287281	12287508	Distal Intergenic
chr18	12287544	12288070	Distal Intergenic
chr18	12288115	12288314	Distal Intergenic
chr18	12288350	12290187	Distal Intergenic
chr18	12413414	12414548	Intron
chr18	12414591	12414802	Intron
chr18	12420051	12421360	Promoter
chr18	12476596	12477308	Intron
chr18	12478579	12479890	Exon
chr18	12549956	12550515	Intron
chr18	12614943	12615628	Intron
chr18	12620704	12621001	Intron
chr18	12621141	12622565	Intron
chr18	12642202	12643775	Intron
chr18	12656228	12656439	Promoter
chr18	12656548	12656949	Promoter
chr18	12660957	12665978	3' UTR
chr18	12669709	12669916	Intron
chr18	12670027	12670818	Intron
chr18	12671030	12671807	Intron
chr18	12769009	12772294	Distal Intergenic
chr18	12785827	12788188	Exon
chr18	12801630	12802163	Exon
chr18	12802507	12804055	Intron

chr18	12806204	12806420	Intron
chr18	12806700	12807590	Intron
chr18	12809468	12810539	Intron
chr18	12811248	12814552	Exon
chr18	12844959	12845690	Intron
chr18	12884057	12884874	Promoter
chr18	12898581	12899711	Distal Intergenic
chr18	13099759	13100012	Intron
chr18	13113894	13114224	Exon
chr18	13114386	13114652	Exon
chr18	14840742	14840986	Intron
chr18	14841039	14841530	Intron
chr18	14931285	14931874	Distal Intergenic
chr18	17449397	17449634	Distal Intergenic
chr18	20117209	20120722	Distal Intergenic
chr18	20130277	20130769	Distal Intergenic
chr18	20151514	20151719	Distal Intergenic
chr18	20151782	20152016	Distal Intergenic
chr18	20152411	20152650	Distal Intergenic
chr18	20156967	20157562	Distal Intergenic
chr18	20157621	20157934	Distal Intergenic
chr18	20158307	20158532	Distal Intergenic
chr18	20158806	20161044	Distal Intergenic
chr18	20164148	20165748	Distal Intergenic
chr18	20185321	20185915	Distal Intergenic
chr18	20192855	20193324	Distal Intergenic
chr18	20193362	20193718	Distal Intergenic
chr18	20264729	20265044	Distal Intergenic
chr18	20265199	20265519	Distal Intergenic
chr18	20300269	20302958	Distal Intergenic
chr18	20304689	20304893	Distal Intergenic
chr18	20307179	20307494	Distal Intergenic
chr18	20307602	20307947	Distal Intergenic
chr18	20315724	20317219	Distal Intergenic
chr18	20345782	20350213	Distal Intergenic
chr18	20386329	20387358	Distal Intergenic
chr18	20533489	20535112	Intron
chr18	20647658	20649118	Distal Intergenic
chr18	20667868	20671124	Distal Intergenic
chr18	20671201	20672111	Distal Intergenic
chr18	20672279	20672494	Distal Intergenic
chr18	20672588	20673053	Distal Intergenic
chr18	20673278	20673725	Distal Intergenic
chr18	20673844	20674955	Distal Intergenic

chr18	20675189	20677095	Distal Intergenic
chr18	20677191	20677507	Distal Intergenic
chr18	20711142	20711910	Distal Intergenic
chr18	20712128	20713676	Promoter
chr18	20717042	20717320	Intron
chr18	20941149	20941404	Intron
chr18	21160435	21161036	Intron
chr18	21310319	21311798	Intron
chr18	23265144	23265343	Distal Intergenic
chr18	23265392	23265594	Distal Intergenic
chr18	23265715	23266016	Distal Intergenic
chr18	23807537	23807747	Intron
chr18	23846712	23847304	Intron
chr18	23941461	23942646	Intron
chr18	24025113	24026201	Distal Intergenic
chr18	24034239	24035700	3' UTR
chr18	24035768	24037428	3' UTR
chr18	24113250	24113616	Intron
chr18	24143488	24143933	Intron
chr18	24178632	24178890	Intron
chr18	24208952	24210879	5' UTR
chr18	24253539	24253777	Distal Intergenic
chr18	24253946	24254369	Distal Intergenic
chr18	24269591	24269790	Exon
chr18	24269878	24271590	Exon
chr18	24274833	24276489	Intron
chr18	24334951	24335296	Distal Intergenic
chr18	24361054	24361635	Distal Intergenic
chr18	24688286	24688609	Intron
chr18	24757309	24759460	Intron
chr18	24760748	24761634	Intron
chr18	24761690	24762317	Intron
chr18	24762424	24762831	Intron
chr18	24878506	24878737	Distal Intergenic
chr18	24878805	24879012	Distal Intergenic
chr18	24880557	24882265	Distal Intergenic
chr18	24900623	24900986	Distal Intergenic
chr18	25019194	25021025	Intron
chr18	25129939	25130369	Intron
chr18	25135442	25135641	Intron
chr18	25139846	25140237	Intron
chr18	25140290	25140589	Intron
chr18	25210669	25210957	Distal Intergenic
chr18	25250394	25250634	Distal Intergenic

chr18	25443286	25443933	Distal Intergenic
chr18	25525493	25526673	Distal Intergenic
chr18	25643769	25645333	Intron
chr18	25669279	25669512	Intron
chr18	25788267	25789630	Distal Intergenic
chr18	25796584	25797116	Distal Intergenic
chr18	25797321	25797956	Distal Intergenic
chr18	25797998	25798369	Distal Intergenic
chr18	30109636	30110537	Distal Intergenic
chr18	30494614	30495176	Distal Intergenic
chr18	31499661	31500059	Intron
chr18	31597187	31599251	Intron
chr18	31919493	31920418	Distal Intergenic
chr18	31948289	31948508	Distal Intergenic
chr18	31948563	31949171	Distal Intergenic
chr18	31963733	31963967	Distal Intergenic
chr18	32211726	32212828	Intron
chr18	32214528	32215305	Intron
chr18	32227684	32228228	Intron
chr18	32312618	32312924	Intron
chr18	32399504	32400181	Intron
chr18	32439846	32440121	Intron
chr18	32495229	32495886	Distal Intergenic
chr18	32502477	32502863	Distal Intergenic
chr18	32502927	32503307	Distal Intergenic
chr18	32506591	32507873	Distal Intergenic
chr18	32537212	32537580	Distal Intergenic
chr18	32779068	32779352	Distal Intergenic
chr18	32793758	32793957	Distal Intergenic
chr18	32794001	32795067	Distal Intergenic
chr18	32798427	32799390	Distal Intergenic
chr18	32800551	32802513	Distal Intergenic
chr18	32803792	32804125	Distal Intergenic
chr18	32804238	32806340	Distal Intergenic
chr18	32806606	32808411	Distal Intergenic
chr18	32808754	32809943	Distal Intergenic
chr18	32996956	32997462	Distal Intergenic
chr18	33372512	33373529	Distal Intergenic
chr18	33373621	33374810	Distal Intergenic
chr18	33954597	33956302	Intron
chr18	33957127	33957384	Intron
chr18	34008954	34010053	Intron
chr18	34028799	34029050	Intron
chr18	34047976	34051718	Intron

chr18	34054658	34055697	Intron
chr18	34067633	34068071	Intron
chr18	34110211	34110584	Intron
chr18	34146954	34147993	Intron
chr18	34380644	34381758	Intron
chr18	34428317	34428526	Intron
chr18	34490241	34490710	Intron
chr18	34531071	34532663	Intron
chr18	34538149	34538469	Promoter
chr18	34538509	34538841	Promoter
chr18	34666623	34668348	Intron
chr18	34783559	34785173	Intron
chr18	34918504	34919351	Intron
chr18	34936860	34937722	Intron
chr18	35057124	35057515	Intron
chr18	35060287	35061872	Intron
chr18	35081496	35081935	Intron
chr18	35089727	35090023	Intron
chr18	35090136	35092443	Intron
chr18	35092665	35092901	Intron
chr18	35107914	35108722	Intron
chr18	35111295	35111623	Intron
chr18	35111801	35112144	Intron
chr18	35112223	35112431	Intron
chr18	35112571	35112786	Intron
chr18	35112884	35113179	Intron
chr18	35113416	35114258	Intron
chr18	35114497	35115242	Intron
chr18	35117676	35118346	Intron
chr18	35118459	35118795	Intron
chr18	35181872	35182742	Distal Intergenic
chr18	35225248	35225706	Distal Intergenic
chr18	35225795	35226060	Distal Intergenic
chr18	35277965	35279863	Distal Intergenic
chr18	35299233	35299493	Distal Intergenic
chr18	35349815	35350160	Distal Intergenic
chr18	35350389	35350911	Distal Intergenic
chr18	35351200	35352402	Distal Intergenic
chr18	35458701	35459719	Distal Intergenic
chr18	35713004	35716387	Distal Intergenic
chr18	35719795	35720287	Distal Intergenic
chr18	35720818	35721066	Distal Intergenic
chr18	35721119	35721489	Distal Intergenic
chr18	35721858	35723918	Distal Intergenic

chr18	35758308	35758684	Distal Intergenic
chr18	35931225	35932365	Distal Intergenic
chr18	36192829	36193554	Distal Intergenic
chr18	36200837	36202701	Distal Intergenic
chr18	36202974	36203471	Distal Intergenic
chr18	36203518	36203727	Distal Intergenic
chr18	36208225	36208482	Distal Intergenic
chr18	36231166	36231409	Distal Intergenic
chr18	36231750	36232050	Distal Intergenic
chr18	36233990	36234431	Distal Intergenic
chr18	36258817	36260157	Distal Intergenic
chr18	36261936	36263279	Distal Intergenic
chr18	36277293	36278078	Distal Intergenic
chr18	36297916	36298989	Distal Intergenic
chr18	36473840	36474060	Distal Intergenic
chr18	36564146	36566151	Distal Intergenic
chr18	36574379	36576807	Distal Intergenic
chr18	36613604	36613838	Distal Intergenic
chr18	36613904	36614127	Exon
chr18	36652134	36653181	Distal Intergenic
chr18	36673765	36674155	Distal Intergenic
chr18	36674389	36674614	Distal Intergenic
chr18	36675741	36676141	Distal Intergenic
chr18	36676860	36677373	Distal Intergenic
chr18	36681452	36681948	Distal Intergenic
chr18	36693817	36694872	Distal Intergenic
chr18	36718926	36719527	Distal Intergenic
chr18	36752295	36752651	Distal Intergenic
chr18	36762233	36763486	Distal Intergenic
chr18	36816504	36816728	Intron
chr18	36828936	36829175	Intron
chr18	36829289	36830406	Intron
chr18	36839260	36839569	Intron
chr18	36868517	36868779	Intron
chr18	36885854	36886494	Intron
chr18	36894047	36895349	Intron
chr18	36910137	36910336	Intron
chr18	36942631	36943294	Intron
chr18	36953518	36953833	Intron
chr18	37803439	37803840	Distal Intergenic
chr18	37803989	37804203	Distal Intergenic
chr18	37966094	37966330	Distal Intergenic
chr18	37985979	37986706	Distal Intergenic
chr18	38007462	38007661	Distal Intergenic

chr18	38008277	38008559	Distal Intergenic
chr18	38008652	38010370	Distal Intergenic
chr18	38035844	38039981	Distal Intergenic
chr18	38040035	38040620	Distal Intergenic
chr18	38043307	38044264	Distal Intergenic
chr18	38083424	38084958	Distal Intergenic
chr18	38085327	38086051	Distal Intergenic
chr18	38087217	38088007	Distal Intergenic
chr18	38093946	38094854	Distal Intergenic
chr18	38123854	38124184	Distal Intergenic
chr18	38239955	38241082	Distal Intergenic
chr18	38304089	38304288	Distal Intergenic
chr18	38365147	38365777	Distal Intergenic
chr18	38409287	38410097	Distal Intergenic
chr18	38410145	38410736	Distal Intergenic
chr18	38411193	38413146	Distal Intergenic
chr18	38414758	38415439	Distal Intergenic
chr18	38415496	38415711	Distal Intergenic
chr18	38418874	38420389	Distal Intergenic
chr18	38433774	38434104	Distal Intergenic
chr18	38434187	38434447	Distal Intergenic
chr18	38456708	38457637	Distal Intergenic
chr18	38507661	38509775	Distal Intergenic
chr18	38543440	38545330	Distal Intergenic
chr18	38566544	38566977	Distal Intergenic
chr18	38578932	38579350	Distal Intergenic
chr18	39040983	39041185	Distal Intergenic
chr18	39041600	39042153	Distal Intergenic
chr18	39471585	39471956	Distal Intergenic
chr18	39478677	39479058	Distal Intergenic
chr18	39479104	39479373	Distal Intergenic
chr18	39533891	39535318	Promoter
chr18	39538158	39538501	Intron
chr18	39540422	39541468	Intron
chr18	39736858	39737132	Distal Intergenic
chr18	39737262	39737542	Distal Intergenic
chr18	39896727	39897827	Intron
chr18	39900947	39901178	Intron
chr18	39959737	39960085	Intron
chr18	40359831	40360242	Intron
chr18	40378813	40379083	Intron
chr18	40642477	40642730	Intron
chr18	42207617	42207965	Distal Intergenic
chr18	42208012	42208247	Distal Intergenic

chr18	42212080	42212447	Distal Intergenic
chr18	42212654	42213232	Distal Intergenic
chr18	42221300	42221770	Distal Intergenic
chr18	42282395	42282997	Intron
chr18	42283189	42284796	Intron
chr18	42421147	42421598	Intron
chr18	42434594	42434861	Intron
chr18	42435012	42435436	Intron
chr18	42435953	42436357	Intron
chr18	42467188	42468533	Intron
chr18	42471571	42472447	Intron
chr18	42493906	42494332	Intron
chr18	42671024	42671938	Distal Intergenic
chr18	42672106	42672561	Distal Intergenic
chr18	42688444	42689904	Distal Intergenic
chr18	42723024	42723265	Distal Intergenic
chr18	42926703	42927498	Intron
chr18	42929769	42931639	Intron
chr18	42952393	42953157	Intron
chr18	42988504	42989138	Intron
chr18	43592549	43594201	Intron
chr18	43595788	43597347	Exon
chr18	43627626	43629866	Intron
chr18	43968424	43968664	Intron
chr18	43976276	43977545	Intron
chr18	43977884	43978700	Intron
chr18	43986232	43986444	Intron
chr18	44006525	44007827	Intron
chr18	44022831	44024136	Intron
chr18	44090515	44091146	Intron
chr18	44121004	44121910	Exon
chr18	44122813	44123022	5' UTR
chr18	44126871	44128391	5' UTR
chr18	44200277	44200555	Intron
chr18	44236007	44236279	Promoter
chr18	44540182	44540424	Intron
chr18	44540528	44541078	Intron
chr18	44554358	44554600	Exon
chr18	44554700	44554907	Exon
chr18	44631697	44633244	Downstream
chr18	44699778	44701266	Intron
chr18	44701817	44703346	Promoter
chr18	44756430	44757837	Exon
chr18	44794821	44795140	Distal Intergenic

chr18	44803320	44803527	Distal Intergenic
chr18	44814260	44815134	Intron
chr18	44815617	44815909	Intron
chr18	44820913	44822740	Intron
chr18	44827507	44829021	Intron
chr18	45420232	45420598	Intron
chr18	45420754	45421048	Intron
chr18	45438131	45439736	Intron
chr18	46439785	46440075	Distal Intergenic
chr18	46440483	46440688	Distal Intergenic
chr18	46440804	46441003	Distal Intergenic
chr18	46484639	46484844	Distal Intergenic
chr18	46540859	46541276	Distal Intergenic
chr18	46549712	46550050	Distal Intergenic
chr18	46683090	46683333	Intron
chr18	46683918	46685269	Intron
chr18	46685955	46686168	Intron
chr18	46701036	46701915	Intron
chr18	46756451	46756964	Intron
chr18	46757409	46759053	Intron
chr18	46901248	46901458	Intron
chr18	47122705	47122964	Distal Intergenic
chr18	47603240	47603584	Intron
chr18	50145958	50146246	Intron
chr18	50146293	50147114	Intron
chr18	50149922	50150149	Intron
chr18	50177454	50178330	Intron
chr18	50182591	50184506	Intron
chr18	50189871	50191710	Intron
chr18	50191748	50192227	Intron
chr18	50192293	50192632	Intron
chr18	50220730	50220935	Intron
chr18	50221099	50223501	Intron
chr18	50223576	50223833	Intron
chr18	50223875	50226172	Intron
chr18	50255558	50256169	Intron
chr18	51733811	51734908	Intron
chr18	53062317	53063825	Intron
chr18	53099895	53100459	Intron
chr18	53357632	53358077	Distal Intergenic
chr18	53397958	53398252	Distal Intergenic
chr18	53409036	53409371	Distal Intergenic
chr18	53409649	53410309	Distal Intergenic
chr18	53505219	53507094	Distal Intergenic

chr18	53576920	53577125	Distal Intergenic
chr18	53850156	53852012	Distal Intergenic
chr18	53904483	53904733	Distal Intergenic
chr18	53961962	53962171	Distal Intergenic
chr18	54021413	54021619	Distal Intergenic
chr18	54021835	54022149	Distal Intergenic
chr18	54022498	54022793	Distal Intergenic
chr18	54063021	54063640	Distal Intergenic
chr18	55019925	55020927	Promoter
chr18	55089222	55089519	Distal Intergenic
chr18	55340072	55340480	Intron
chr18	55355727	55356503	Exon
chr18	55425733	55425981	Intron
chr18	55783608	55784056	Intron
chr18	55806875	55808264	Intron
chr18	56579253	56579547	Intron
chr18	56603114	56603330	Intron
chr18	56637020	56637781	Intron
chr18	56650526	56651085	Intron
chr18	56773594	56775109	Distal Intergenic
chr18	56846717	56849111	Distal Intergenic
chr18	56866777	56867166	Distal Intergenic
chr18	56867488	56867729	Distal Intergenic
chr18	56868681	56869028	Distal Intergenic
chr18	56881925	56882277	Distal Intergenic
chr18	56882337	56884299	Distal Intergenic
chr18	56885370	56887047	Promoter
chr18	56896035	56897467	Intron
chr18	57241683	57242574	Intron
chr18	57244134	57244819	Intron
chr18	57945935	57946509	Distal Intergenic
chr18	57947697	57947972	Distal Intergenic
chr18	57948017	57948539	Distal Intergenic
chr18	57964260	57965738	Distal Intergenic
chr18	57970729	57970994	Distal Intergenic
chr18	57996296	57997932	Distal Intergenic
chr18	58015872	58016242	Distal Intergenic
chr18	58017467	58018281	Distal Intergenic
chr18	58727634	58728138	Distal Intergenic
chr18	60640390	60641577	Intron
chr18	60660058	60663173	Distal Intergenic
chr18	60683146	60683468	Distal Intergenic
chr18	60684951	60685826	Distal Intergenic
chr18	60686301	60687530	Distal Intergenic

chr18	60808238	60809539	Intron
chr18	60828667	60829264	Intron
chr18	60895935	60897242	Intron
chr18	60899813	60901492	Intron
chr18	60905394	60907859	Intron
chr18	60908274	60909126	Intron
chr18	60909794	60910761	Intron
chr18	60910945	60912372	Intron
chr18	60915830	60916664	Intron
chr18	60934015	60934454	Intron
chr18	60934496	60935390	Intron
chr18	60935573	60935854	Intron
chr18	61336005	61336417	Distal Intergenic
chr18	61336546	61336805	Distal Intergenic
chr18	61374563	61374814	Intron
chr18	61375169	61375472	Intron
chr18	61383339	61384650	Exon
chr18	61676610	61676919	Distal Intergenic
chr18	61710264	61710580	Distal Intergenic
chr18	61714272	61714648	Distal Intergenic
chr18	61714904	61717074	Distal Intergenic
chr18	61778826	61779040	Intron
chr18	61785471	61787216	Intron
chr18	61791220	61794372	Intron
chr18	61794629	61794829	Exon
chr18	61795651	61800558	Intron
chr18	61850762	61851022	Intron
chr18	61904107	61906240	Intron
chr18	61934120	61934535	Intron
chr18	61934842	61935189	Intron
chr18	61997109	61997712	Intron
chr18	62101037	62101236	Distal Intergenic
chr18	62111432	62113033	Distal Intergenic
chr18	62175877	62176589	Distal Intergenic
chr18	62176941	62177356	Distal Intergenic
chr18	62191913	62194436	Distal Intergenic
chr18	62260600	62263769	Distal Intergenic
chr18	62281059	62282911	Distal Intergenic
chr18	62328359	62329825	Distal Intergenic
chr18	62338047	62338598	Distal Intergenic
chr18	62340066	62340433	Distal Intergenic
chr18	62362423	62364257	Distal Intergenic
chr18	62708106	62708323	Distal Intergenic
chr18	62708593	62708804	Distal Intergenic

chr18	62744868	62745090	Distal Intergenic
chr18	63065731	63066783	Distal Intergenic
chr18	63153376	63153983	Distal Intergenic
chr18	63200230	63200959	Distal Intergenic
chr18	63202300	63202507	Distal Intergenic
chr18	63560375	63560805	Distal Intergenic
chr18	63622724	63623451	Distal Intergenic
chr18	63850337	63850558	Distal Intergenic
chr18	63850819	63851991	Distal Intergenic
chr18	63852100	63852407	Distal Intergenic
chr18	63990531	63990880	Distal Intergenic
chr18	64041395	64041605	Distal Intergenic
chr18	64641891	64643014	Distal Intergenic
chr18	64672125	64672446	Distal Intergenic
chr18	64675457	64676025	Distal Intergenic
chr18	65098831	65099964	Distal Intergenic
chr18	65104575	65105990	Distal Intergenic
chr18	65210081	65211609	Intron
chr18	65269174	65269817	Intron
chr18	65540746	65542016	Exon
chr18	65725132	65725430	Distal Intergenic
chr18	65725523	65725824	Distal Intergenic
chr18	65739591	65740367	Distal Intergenic
chr18	65740488	65741048	Distal Intergenic
chr18	65757573	65758479	Distal Intergenic
chr18	65762992	65763831	Distal Intergenic
chr18	65765222	65765429	Distal Intergenic
chr18	65766004	65766308	Distal Intergenic
chr18	65766354	65767335	Distal Intergenic
chr18	65775701	65775900	Distal Intergenic
chr18	65785871	65788498	Distal Intergenic
chr18	65829306	65830357	Distal Intergenic
chr18	65857777	65859517	Distal Intergenic
chr18	65864325	65866253	Distal Intergenic
chr18	65897908	65898252	Distal Intergenic
chr18	65898971	65899762	Distal Intergenic
chr18	65954727	65955184	Distal Intergenic
chr18	66618418	66618626	Intron
chr18	66631501	66631881	Intron
chr18	66632258	66632737	Intron
chr18	66637606	66637950	Intron
chr18	67126462	67126712	Intron
chr18	67354012	67355232	Intron
chr18	67458941	67459572	Intron

chr18	67461198	67461404	Intron
chr18	67461669	67461888	Intron
chr18	67469595	67470529	Intron
chr18	67470578	67470786	Intron
chr18	67471773	67472024	Intron
chr18	67477346	67478645	Intron
chr18	67550828	67551033	Intron
chr18	67551257	67551901	Intron
chr18	67709360	67709810	Intron
chr18	67737156	67738268	Intron
chr18	67801293	67801604	Intron
chr18	67865930	67866185	Promoter
chr18	67870600	67871465	5' UTR
chr18	67882916	67883438	Distal Intergenic
chr18	67934697	67935099	Distal Intergenic
chr18	67974947	67975317	Intron
chr18	68105453	68105697	Distal Intergenic
chr18	68142964	68145571	Distal Intergenic
chr18	68145624	68145916	Distal Intergenic
chr18	68216994	68217813	Distal Intergenic
chr18	68217888	68218719	Distal Intergenic
chr18	68243747	68244491	Distal Intergenic
chr18	68278929	68279539	Distal Intergenic
chr18	68314972	68315175	Distal Intergenic
chr18	68316069	68318380	Distal Intergenic
chr18	68319769	68320006	Distal Intergenic
chr18	68320121	68320475	Distal Intergenic
chr18	68349730	68350289	Distal Intergenic
chr18	68351192	68351433	Distal Intergenic
chr18	68460030	68460302	Distal Intergenic
chr18	68947620	68948325	Distal Intergenic
chr18	68948577	68948979	Distal Intergenic
chr18	68988867	68989238	Distal Intergenic
chr18	69504945	69505211	Distal Intergenic
chr18	69505444	69505729	Distal Intergenic
chr18	69814833	69815196	Distal Intergenic
chr18	69973753	69974567	Distal Intergenic
chr18	70085423	70085673	Distal Intergenic
chr18	70152054	70152454	Distal Intergenic
chr18	70182178	70182443	Distal Intergenic
chr18	70182593	70182945	Distal Intergenic
chr18	70403773	70404717	Distal Intergenic
chr18	70412844	70414011	3' UTR
chr18	70690093	70690424	Distal Intergenic

chr18	70690636	70690891	Distal Intergenic
chr18	70728788	70729017	Distal Intergenic
chr18	70760613	70761703	Distal Intergenic
chr18	70803858	70804480	Distal Intergenic
chr18	71293236	71293501	Distal Intergenic
chr18	73462849	73463094	Distal Intergenic
chr18	73463214	73464420	Distal Intergenic
chr18	73731142	73731772	Distal Intergenic
chr18	73732424	73732819	Distal Intergenic
chr18	73732973	73733207	Distal Intergenic
chr18	73733329	73734597	Distal Intergenic
chr18	73734730	73734931	Distal Intergenic
chr18	73876589	73877335	Distal Intergenic
chr18	73877729	73878210	Distal Intergenic
chr18	74507372	74507937	Exon
chr18	75106607	75107052	Distal Intergenic
chr18	75164597	75165400	Distal Intergenic
chr18	75330400	75331843	Distal Intergenic
chr18	75352813	75353810	Distal Intergenic
chr18	75501493	75502381	Distal Intergenic
chr18	75502594	75502847	Distal Intergenic
chr18	75504394	75505161	Distal Intergenic
chr18	75522746	75522976	Distal Intergenic
chr18	75523267	75524593	Distal Intergenic
chr18	75526434	75526800	Distal Intergenic
chr18	75527698	75528645	Distal Intergenic
chr18	75528757	75529066	Distal Intergenic
chr18	75529228	75529735	Distal Intergenic
chr18	75529900	75530254	Distal Intergenic
chr18	75530491	75531881	Distal Intergenic
chr18	75532428	75532747	Distal Intergenic
chr18	75532819	75533933	Distal Intergenic
chr18	75534290	75535382	Distal Intergenic
chr18	75539742	75539986	Distal Intergenic
chr18	75546483	75546785	Distal Intergenic
chr18	75546943	75547168	Distal Intergenic
chr18	75614997	75616186	Distal Intergenic
chr18	75616255	75616482	Distal Intergenic
chr18	75654220	75654891	Distal Intergenic
chr18	75715454	75716162	Distal Intergenic
chr18	75716341	75716585	Distal Intergenic
chr18	76018003	76018448	Distal Intergenic
chr18	76020595	76021645	Distal Intergenic
chr18	76030873	76031103	Distal Intergenic

chr18	76212797	76213656	Distal Intergenic
chr18	76256477	76257479	Distal Intergenic
chr18	76269988	76271409	Distal Intergenic
chr18	76443035	76443259	Distal Intergenic
chr18	76495076	76495861	Distal Intergenic
chr18	77293263	77293935	Distal Intergenic
chr18	77489738	77489981	Intron
chr18	77902454	77903299	Distal Intergenic
chr18	78038315	78038904	Distal Intergenic
chr18	78118606	78119227	Distal Intergenic
chr18	78141439	78141948	Distal Intergenic
chr18	78145557	78145787	Distal Intergenic
chr18	78677129	78677924	Distal Intergenic
chr18	78690448	78690746	Distal Intergenic
chr18	78694701	78694910	Distal Intergenic
chr18	78774226	78774537	Distal Intergenic
chr18	79205202	79205979	Distal Intergenic
chr18	79393246	79393528	Distal Intergenic
chr18	79663635	79664482	Distal Intergenic
chr18	80201983	80203710	Distal Intergenic
chr18	80243829	80245229	Distal Intergenic
chr18	80246770	80247031	Distal Intergenic
chr18	80247208	80249265	Distal Intergenic
chr18	80253448	80254697	Distal Intergenic
chr18	80273177	80274650	Distal Intergenic
chr18	80296996	80297300	Distal Intergenic
chr18	80300125	80300332	Distal Intergenic
chr18	80300623	80300840	Distal Intergenic
chr18	80306721	80309944	Distal Intergenic
chr18	80312301	80313439	Distal Intergenic
chr18	80346953	80347648	Distal Intergenic
chr18	80348038	80348474	Distal Intergenic
chr18	80366125	80367739	Distal Intergenic
chr18	80369586	80369871	Distal Intergenic
chr18	80449152	80450543	Distal Intergenic
chr18	80452008	80452896	Distal Intergenic
chr18	80453063	80453353	Distal Intergenic
chr18	80453934	80454239	Distal Intergenic
chr18	80457615	80460848	Distal Intergenic
chr18	80511795	80512015	Distal Intergenic
chr18	80666963	80667209	Distal Intergenic
chr18	80751372	80751651	Distal Intergenic
chr18	80751800	80752186	Distal Intergenic
chr18	80780475	80780742	Distal Intergenic

chr18	80852615	80852814	Distal Intergenic
chr18	80853329	80853532	Distal Intergenic
chr18	81129530	81129821	Distal Intergenic
chr18	81130056	81130863	Distal Intergenic
chr18	81248089	81248448	Distal Intergenic
chr18	81248646	81248866	Distal Intergenic
chr18	82104303	82104516	Distal Intergenic
chr18	82657817	82659997	Distal Intergenic
chr18	82661810	82662602	Distal Intergenic
chr18	82662800	82664320	Distal Intergenic
chr18	82667717	82667916	Distal Intergenic
chr18	82668144	82668735	Distal Intergenic
chr18	82687294	82687596	Distal Intergenic
chr18	82691292	82692911	Distal Intergenic
chr18	82694040	82694246	Distal Intergenic
chr18	82861368	82861930	Distal Intergenic
chr18	82862364	82862802	Distal Intergenic
chr18	82907185	82907487	Distal Intergenic
chr18	83082812	83083105	Distal Intergenic
chr18	83084609	83084810	Distal Intergenic
chr18	83085702	83086505	Distal Intergenic
chr18	83503303	83503706	Distal Intergenic
chr18	84064368	84064656	Distal Intergenic
chr18	84478746	84479430	Distal Intergenic
chr18	84758904	84759862	Distal Intergenic
chr18	85049222	85050269	Distal Intergenic
chr18	85050378	85050953	Distal Intergenic
chr18	85063395	85063701	Distal Intergenic
chr18	85063738	85063962	Distal Intergenic
chr18	85593934	85594924	Distal Intergenic
chr18	87639611	87639814	Distal Intergenic
chr18	89004551	89004908	Distal Intergenic
chr18	89019932	89020255	Distal Intergenic
chr18	89062557	89063445	Distal Intergenic
chr18	89068642	89069808	Distal Intergenic
chr18	89077287	89078016	Distal Intergenic
chr18	89096863	89097452	Distal Intergenic
chr18	89097624	89098691	Distal Intergenic
chr18	89267206	89267691	Distal Intergenic
chr18	89728959	89729357	Distal Intergenic
chr19	3282612	3283277	Intron
chr19	3324298	3325167	Distal Intergenic
chr19	3333768	3334167	Distal Intergenic
chr19	3353666	3353930	Distal Intergenic

chr19	3519231	3520047	Intron
chr19	3525965	3526817	Exon
chr19	3554985	3555392	Intron
chr19	3574776	3575152	Intron
chr19	3575477	3575702	Exon
chr19	3624452	3626617	Promoter
chr19	3654801	3655069	Intron
chr19	3663627	3663867	Intron
chr19	3664098	3664382	Intron
chr19	3668830	3670112	Intron
chr19	3767947	3768704	Downstream
chr19	3832986	3833185	Intron
chr19	3838856	3840693	Intron
chr19	3852491	3852690	Exon
chr19	3903864	3904965	Intron
chr19	3905229	3906868	Exon
chr19	3907144	3907514	Intron
chr19	3913087	3913366	Intron
chr19	4000315	4001190	Distal Intergenic
chr19	4045479	4047471	3' UTR
chr19	4093628	4094210	Intron
chr19	4099206	4099944	Exon
chr19	4112984	4113605	Intron
chr19	4113984	4114245	Intron
chr19	4125790	4127367	Distal Intergenic
chr19	4134842	4136353	Distal Intergenic
chr19	4148560	4148855	Distal Intergenic
chr19	4192196	4192916	Intron
chr19	4201237	4201611	Intron
chr19	4213594	4214810	Intron
chr19	4214861	4215232	Intron
chr19	4231886	4232110	Intron
chr19	4243983	4245835	Distal Intergenic
chr19	4268528	4269379	3' UTR
chr19	4298828	4299175	Intron
chr19	4299561	4300822	Intron
chr19	4305419	4306064	Promoter
chr19	4326051	4326818	Intron
chr19	4332491	4334653	Exon
chr19	4395038	4396721	Intron
chr19	4396870	4397119	Intron
chr19	4419043	4421747	Intron
chr19	4429884	4431398	Exon
chr19	4437845	4441362	Intron

chr19	4478046	4478263	Intron
chr19	4481727	4482120	Intron
chr19	4614750	4614959	Distal Intergenic
chr19	4615563	4615804	Distal Intergenic
chr19	4625367	4625643	Distal Intergenic
chr19	4625817	4626087	Distal Intergenic
chr19	4665051	4665953	Intron
chr19	4670650	4672890	Promoter
chr19	4686447	4687422	Intron
chr19	4720619	4721052	Intron
chr19	4721223	4721475	Intron
chr19	4729514	4730001	Distal Intergenic
chr19	4741384	4741697	Distal Intergenic
chr19	4741774	4742072	Distal Intergenic
chr19	4755814	4756105	Distal Intergenic
chr19	4793265	4794773	3' UTR
chr19	4811177	4811584	Distal Intergenic
chr19	4811841	4812280	Distal Intergenic
chr19	4858849	4859661	Exon
chr19	4928406	4928623	Intron
chr19	4962053	4962584	3' UTR
chr19	5012829	5013851	Intron
chr19	5015918	5017012	Promoter
chr19	5017117	5019442	Promoter
chr19	5041116	5041760	Exon
chr19	5049658	5052012	Intron
chr19	5057526	5059528	Intron
chr19	5060302	5060649	Intron
chr19	5060696	5061336	Intron
chr19	5061409	5061763	Intron
chr19	5088109	5088308	Intron
chr19	5256890	5257521	Intron
chr19	5294856	5295440	Intron
chr19	5300391	5301036	Intron
chr19	5345292	5346475	Distal Intergenic
chr19	5366706	5367693	Distal Intergenic
chr19	5388533	5389385	Distal Intergenic
chr19	5424333	5424808	Distal Intergenic
chr19	5424998	5425708	Distal Intergenic
chr19	5428075	5431029	Distal Intergenic
chr19	5431688	5433252	Distal Intergenic
chr19	5433464	5434132	Distal Intergenic
chr19	5445157	5445376	Distal Intergenic
chr19	5447677	5449202	Distal Intergenic

chr19	5449373	5450222	Distal Intergenic
chr19	5488747	5489053	Distal Intergenic
chr19	5489218	5489461	Distal Intergenic
chr19	5490577	5491047	Distal Intergenic
chr19	5491215	5491643	Distal Intergenic
chr19	5529575	5530104	Distal Intergenic
chr19	5566836	5567596	Promoter
chr19	5572278	5572946	Distal Intergenic
chr19	5609327	5610535	Exon
chr19	5637495	5637694	Intron
chr19	5637792	5638058	Intron
chr19	5688850	5690216	Promoter
chr19	5703587	5705539	Intron
chr19	5713640	5714311	5' UTR
chr19	5724393	5726300	Exon
chr19	5729111	5729327	Intron
chr19	5729364	5729706	Intron
chr19	5749609	5749822	Intron
chr19	5793205	5793767	Distal Intergenic
chr19	5793908	5794338	Distal Intergenic
chr19	5794399	5795795	Distal Intergenic
chr19	5795949	5796185	Distal Intergenic
chr19	5799159	5805004	Distal Intergenic
chr19	5805055	5805295	Distal Intergenic
chr19	5808813	5809285	Distal Intergenic
chr19	5809456	5811535	Distal Intergenic
chr19	5811581	5812851	Distal Intergenic
chr19	5814443	5816725	Distal Intergenic
chr19	5817291	5818095	Distal Intergenic
chr19	5818168	5818382	Distal Intergenic
chr19	5831357	5832605	5' UTR
chr19	5836447	5836886	Intron
chr19	5837147	5845880	Promoter
chr19	5846110	5846476	5' UTR
chr19	5846621	5850851	Promoter
chr19	5851076	5852470	Promoter
chr19	5877538	5878069	Intron
chr19	5911942	5912807	Distal Intergenic
chr19	5912950	5913349	Promoter
chr19	5944854	5945965	Intron
chr19	5953766	5954417	Intron
chr19	5963927	5964151	Intron
chr19	5964772	5965213	Intron
chr19	5965451	5965832	Intron

chr19	5965997	5967365	Intron
chr19	5991666	5992171	Intron
chr19	6014057	6015970	Intron
chr19	6046112	6046460	Intron
chr19	6057959	6058288	Intron
chr19	6058336	6058976	Intron
chr19	6059751	6059950	Intron
chr19	6060276	6061417	Intron
chr19	6061710	6062409	Intron
chr19	6076647	6077250	Intron
chr19	6084906	6085919	Intron
chr19	6117389	6119653	Distal Intergenic
chr19	6125006	6125709	Distal Intergenic
chr19	6127564	6128278	Distal Intergenic
chr19	6136026	6137228	Promoter
chr19	6235951	6236328	Intron
chr19	6277427	6279059	Promoter
chr19	6295748	6298213	Distal Intergenic
chr19	6305806	6306042	Downstream
chr19	6306170	6306426	Downstream
chr19	6306617	6308566	3' UTR
chr19	6334512	6335202	Promoter
chr19	6363702	6364480	Exon
chr19	6893951	6894609	Intron
chr19	6894876	6895524	Intron
chr19	6895572	6895771	Intron
chr19	6914506	6916605	Exon
chr19	6934858	6936032	Exon
chr19	6951622	6951874	Downstream
chr19	6984081	6985105	5' UTR
chr19	6995352	6995808	Distal Intergenic
chr19	7016376	7017284	Distal Intergenic
chr19	7048959	7049616	Promoter
chr19	7049733	7051096	Promoter
chr19	7054162	7054361	Downstream
chr19	7055119	7055481	Downstream
chr19	7066779	7066994	Distal Intergenic
chr19	7067153	7067356	Distal Intergenic
chr19	7092234	7093113	Distal Intergenic
chr19	7097701	7098584	Distal Intergenic
chr19	7113565	7114501	3' UTR
chr19	7115352	7115691	3' UTR
chr19	7128959	7130114	Exon
chr19	7130427	7130950	Intron

chr19	7165816	7166905	Exon
chr19	7222199	7223881	Intron
chr19	7233220	7235119	Intron
chr19	7235205	7236273	Intron
chr19	7279036	7279272	Intron
chr19	7279355	7281234	Intron
chr19	7291517	7292615	Intron
chr19	7332148	7332443	Distal Intergenic
chr19	7369494	7370384	Distal Intergenic
chr19	7372063	7372655	Distal Intergenic
chr19	7375859	7377679	Distal Intergenic
chr19	7377810	7378036	Distal Intergenic
chr19	7381905	7383664	Distal Intergenic
chr19	7384087	7386949	Distal Intergenic
chr19	7410126	7410766	Distal Intergenic
chr19	7413958	7415066	Distal Intergenic
chr19	7415298	7416356	Distal Intergenic
chr19	7416418	7416949	Distal Intergenic
chr19	7456561	7457504	Intron
chr19	7533327	7534615	Exon
chr19	7557939	7558245	Distal Intergenic
chr19	7566393	7567620	Intron
chr19	7567958	7568206	Intron
chr19	7568550	7569153	Exon
chr19	7569333	7569716	Promoter
chr19	7573223	7573815	3' UTR
chr19	8773477	8774085	Distal Intergenic
chr19	8776520	8777457	Distal Intergenic
chr19	8777561	8777898	Distal Intergenic
chr19	8779446	8782381	Distal Intergenic
chr19	8783878	8788597	Distal Intergenic
chr19	8796744	8801769	Distal Intergenic
chr19	8801805	8802795	Distal Intergenic
chr19	8815645	8815861	Distal Intergenic
chr19	8815979	8816532	Distal Intergenic
chr19	8830827	8831269	Distal Intergenic
chr19	8831554	8832158	Distal Intergenic
chr19	8848128	8849171	Distal Intergenic
chr19	8872414	8872643	Distal Intergenic
chr19	8876493	8876796	Distal Intergenic
chr19	8877008	8878302	Distal Intergenic
chr19	8893027	8893902	Distal Intergenic
chr19	8894188	8894683	Distal Intergenic
chr19	8945729	8945965	Distal Intergenic

chr19	8946036	8947078	Distal Intergenic
chr19	8961178	8961448	Intron
chr19	8961484	8962434	Exon
chr19	8962495	8964123	Intron
chr19	8967514	8967871	Intron
chr19	8972262	8972470	Intron
chr19	8972543	8972822	Intron
chr19	8973092	8973452	Intron
chr19	8994451	8995214	Exon
chr19	8995311	8995607	Intron
chr19	9016151	9019328	Exon
chr19	9027519	9029064	Exon
chr19	9040626	9042963	Intron
chr19	9043122	9043358	Intron
chr19	9051055	9051762	Intron
chr19	9053934	9054458	Exon
chr19	9054507	9059144	Exon
chr19	9062130	9062637	Exon
chr19	9062972	9064451	Exon
chr19	9064499	9065297	Exon
chr19	9065398	9067874	Exon
chr19	9068549	9071154	Exon
chr19	9071224	9073000	Exon
chr19	9107248	9108526	Distal Intergenic
chr19	9133810	9135301	Distal Intergenic
chr19	9135491	9135883	Distal Intergenic
chr19	9140158	9141061	Distal Intergenic
chr19	9147804	9149413	Distal Intergenic
chr19	9180678	9182604	Distal Intergenic
chr19	9972070	9974055	Intron
chr19	9974108	9974937	Intron
chr19	10012789	10013009	Intron
chr19	10013151	10014034	Intron
chr19	10028514	10029600	Intron
chr19	10057208	10057538	Distal Intergenic
chr19	10114555	10115231	Exon
chr19	10115964	10118540	Exon
chr19	10174866	10175105	Intron
chr19	10175235	10176149	Intron
chr19	10176652	10177112	Intron
chr19	10187092	10187474	Distal Intergenic
chr19	10187527	10188060	Distal Intergenic
chr19	10211257	10213198	Promoter
chr19	10232212	10232463	Distal Intergenic

chr19	10237375	10238135	Distal Intergenic
chr19	10269510	10269717	Intron
chr19	10269797	10270101	Intron
chr19	10270168	10272124	Exon
chr19	10277786	10278885	Promoter
chr19	10364050	10365004	Intron
chr19	10599106	10599713	Intron
chr19	10599766	10600163	Exon
chr19	10635472	10637241	Distal Intergenic
chr19	10676544	10680674	Promoter
chr19	10731223	10732313	Intron
chr19	10762945	10763196	Exon
chr19	10763514	10764300	Promoter
chr19	10764407	10764663	Promoter
chr19	10908175	10908400	Exon
chr19	10943762	10945220	3' UTR
chr19	10954586	10954834	Distal Intergenic
chr19	10954884	10955240	Distal Intergenic
chr19	10956333	10956661	Distal Intergenic
chr19	11022948	11023854	Promoter
chr19	11026246	11026931	Intron
chr19	11395569	11395894	Distal Intergenic
chr19	11844909	11845470	Intron
chr19	11884476	11884675	Intron
chr19	11884841	11885676	Intron
chr19	11887170	11888247	Intron
chr19	11892144	11892593	Exon
chr19	11892970	11893499	3' UTR
chr19	12079396	12079626	Intron
chr19	12080059	12080429	Intron
chr19	12084651	12085006	Intron
chr19	12547786	12548289	Intron
chr19	12548552	12548758	Intron
chr19	12549837	12550138	Intron
chr19	12550186	12550505	Intron
chr19	12565500	12566542	Distal Intergenic
chr19	12649322	12649760	Intron
chr19	12707587	12707840	Intron
chr19	12832738	12832952	Promoter
chr19	12833047	12833956	Promoter
chr19	12836919	12838045	Distal Intergenic
chr19	12840684	12840933	Downstream
chr19	12868582	12869178	3' UTR
chr19	12869388	12869632	Distal Intergenic

chr19	12870427	12870655	Distal Intergenic
chr19	12870717	12871412	Downstream
chr19	12871512	12871753	Downstream
chr19	14272114	14272921	Exon
chr19	15998158	15998677	Intron
chr19	16015362	16016954	Distal Intergenic
chr19	16020715	16020914	Downstream
chr19	16021043	16021467	Downstream
chr19	16090235	16091627	Distal Intergenic
chr19	16150026	16150437	Exon
chr19	16160909	16161262	Distal Intergenic
chr19	16161439	16163123	Distal Intergenic
chr19	16241070	16242884	Intron
chr19	16244978	16245411	Promoter
chr19	16256517	16256749	Intron
chr19	16277034	16277887	Intron
chr19	16278810	16280355	Exon
chr19	16284528	16284769	Promoter
chr19	16325177	16325861	Intron
chr19	16344095	16346093	3' UTR
chr19	16346178	16346377	Distal Intergenic
chr19	16346952	16348388	Distal Intergenic
chr19	16360596	16361871	Distal Intergenic
chr19	16400128	16400817	Distal Intergenic
chr19	16446173	16446841	Distal Intergenic
chr19	16460547	16461550	Distal Intergenic
chr19	16483494	16485415	Intron
chr19	16485573	16485779	Intron
chr19	16485822	16486098	Intron
chr19	16492879	16493118	Intron
chr19	16587840	16589312	Downstream
chr19	16718942	16720254	Intron
chr19	17085867	17086081	Exon
chr19	17237908	17238169	Intron
chr19	17330651	17331924	Distal Intergenic
chr19	17335047	17335461	Distal Intergenic
chr19	17337731	17338340	Promoter
chr19	17338395	17338596	Intron
chr19	17435108	17435456	Intron
chr19	17875077	17875483	5' UTR
chr19	18687906	18688189	3' UTR
chr19	18696936	18698812	Promoter
chr19	18704481	18705831	Exon
chr19	18706101	18706302	Intron

chr19	18732750	18733953	Distal Intergenic
chr19	18734292	18734546	Distal Intergenic
chr19	18734760	18736760	Distal Intergenic
chr19	19320765	19322303	Promoter
chr19	20460527	20461413	Intron
chr19	20462367	20465466	Intron
chr19	20465653	20466022	Intron
chr19	20467647	20469264	Intron
chr19	20476964	20477879	Intron
chr19	20480816	20481585	Intron
chr19	21125773	21126753	3' UTR
chr19	21151998	21153163	Distal Intergenic
chr19	21346768	21349624	5' UTR
chr19	21678764	21680700	Exon
chr19	21718098	21718297	Intron
chr19	21718527	21719140	Exon
chr19	21720117	21721068	3' UTR
chr19	21726371	21727548	Intron
chr19	21847945	21848268	Distal Intergenic
chr19	21848310	21848720	Distal Intergenic
chr19	21852868	21853474	Distal Intergenic
chr19	21888568	21888832	Distal Intergenic
chr19	21894328	21895051	Distal Intergenic
chr19	21936897	21938660	Intron
chr19	22109411	22110148	Distal Intergenic
chr19	22110195	22111066	Distal Intergenic
chr19	22990874	22991483	Distal Intergenic
chr19	23254657	23255517	Distal Intergenic
chr19	23403732	23404011	Downstream
chr19	23404049	23404636	Downstream
chr19	23429078	23429608	Intron
chr19	23594801	23597141	Intron
chr19	24150272	24151638	Distal Intergenic
chr19	24162828	24163165	Distal Intergenic
chr19	24164280	24165155	Distal Intergenic
chr19	24240695	24241700	Intron
chr19	24247798	24248443	Exon
chr19	24248890	24250036	Exon
chr19	24267097	24268122	Intron
chr19	24271325	24272197	Intron
chr19	24298672	24298972	Intron
chr19	24299055	24299264	Intron
chr19	24299749	24300324	Intron
chr19	24336129	24336870	Distal Intergenic

chr19	24551834	24552382	Distal Intergenic
chr19	24806606	24807439	Distal Intergenic
chr19	25069131	25069337	Distal Intergenic
chr19	25134217	25135605	Distal Intergenic
chr19	25241216	25242170	Distal Intergenic
chr19	25242331	25242553	Distal Intergenic
chr19	25245011	25248368	Distal Intergenic
chr19	25249030	25249573	Distal Intergenic
chr19	25259907	25266160	Distal Intergenic
chr19	25338137	25339793	Distal Intergenic
chr19	25340927	25343296	Distal Intergenic
chr19	25364819	25365267	Distal Intergenic
chr19	25415630	25417628	Distal Intergenic
chr19	25418952	25419279	Distal Intergenic
chr19	25426829	25427072	Distal Intergenic
chr19	25427194	25427397	Distal Intergenic
chr19	25431257	25432449	Distal Intergenic
chr19	25446886	25449898	Distal Intergenic
chr19	25473960	25475641	Distal Intergenic
chr19	25500490	25502242	Distal Intergenic
chr19	25502437	25503593	Distal Intergenic
chr19	25535938	25536921	Distal Intergenic
chr19	25654851	25655132	Distal Intergenic
chr19	26165012	26165250	Distal Intergenic
chr19	26165306	26166720	Distal Intergenic
chr19	26214843	26215708	Distal Intergenic
chr19	26218123	26218358	Distal Intergenic
chr19	26287586	26288614	Distal Intergenic
chr19	26512962	26513181	Distal Intergenic
chr19	26717341	26718233	Distal Intergenic
chr19	26807644	26808637	Distal Intergenic
chr19	26820648	26823159	Distal Intergenic
chr19	26920021	26920253	Distal Intergenic
chr19	26965593	26966694	Distal Intergenic
chr19	26967569	26967904	Distal Intergenic
chr19	27012956	27013301	Distal Intergenic
chr19	27013499	27014138	Distal Intergenic
chr19	27089921	27091171	Distal Intergenic
chr19	27114556	27114820	Distal Intergenic
chr19	27136587	27136816	Distal Intergenic
chr19	27136853	27137244	Distal Intergenic
chr19	27139941	27140249	Distal Intergenic
chr19	27200567	27201517	Distal Intergenic
chr19	27208831	27209526	Distal Intergenic

chr19	27251107	27252196	Distal Intergenic
chr19	27253386	27256920	Distal Intergenic
chr19	27257665	27258896	Distal Intergenic
chr19	27300963	27302413	Distal Intergenic
chr19	27372223	27373047	Distal Intergenic
chr19	27425926	27426476	Distal Intergenic
chr19	27503705	27503904	Distal Intergenic
chr19	27504140	27504374	Distal Intergenic
chr19	27504472	27504852	Distal Intergenic
chr19	27978692	27978942	Distal Intergenic
chr19	27978997	27979968	Distal Intergenic
chr19	28085887	28086119	Distal Intergenic
chr19	28086380	28086796	Distal Intergenic
chr19	28273030	28274717	Distal Intergenic
chr19	28277899	28278338	Distal Intergenic
chr19	28278402	28278721	Downstream
chr19	28320660	28321499	Distal Intergenic
chr19	28348719	28349138	Distal Intergenic
chr19	28370682	28370992	Distal Intergenic
chr19	28371116	28371364	Distal Intergenic
chr19	28373467	28375461	Distal Intergenic
chr19	28463007	28464616	Distal Intergenic
chr19	28549953	28550335	Distal Intergenic
chr19	28592623	28593004	Distal Intergenic
chr19	28593062	28593438	Distal Intergenic
chr19	28593616	28593954	Distal Intergenic
chr19	28632184	28633785	Distal Intergenic
chr19	28690466	28690733	Distal Intergenic
chr19	28697809	28699632	Distal Intergenic
chr19	28877671	28878369	Distal Intergenic
chr19	29064921	29065333	Intron
chr19	29115904	29116267	Intron
chr19	29116526	29117464	Intron
chr19	29325367	29325827	Distal Intergenic
chr19	29345324	29346599	Distal Intergenic
chr19	29374753	29375583	Distal Intergenic
chr19	29458585	29460198	Exon
chr19	29596964	29597599	Distal Intergenic
chr19	29598232	29598435	Distal Intergenic
chr19	29678453	29679031	Distal Intergenic
chr19	29715830	29716040	Distal Intergenic
chr19	29967005	29967658	Intron
chr19	29968087	29969537	Intron
chr19	30105850	30106437	3' UTR

chr19	30647210	30648141	Distal Intergenic
chr19	30925308	30925676	Intron
chr19	30927635	30929043	Intron
chr19	31193631	31193874	Distal Intergenic
chr19	31924318	31926559	Distal Intergenic
chr19	32140840	32141904	Distal Intergenic
chr19	32142254	32142520	Distal Intergenic
chr19	32142777	32143025	Distal Intergenic
chr19	32143083	32145239	Distal Intergenic
chr19	32145440	32145884	Distal Intergenic
chr19	32162464	32163939	Distal Intergenic
chr19	32181845	32182493	Distal Intergenic
chr19	32182677	32182915	Distal Intergenic
chr19	32247066	32247404	Distal Intergenic
chr19	32254183	32254704	Distal Intergenic
chr19	32256187	32256449	Distal Intergenic
chr19	32256571	32256852	Distal Intergenic
chr19	32264383	32265383	Distal Intergenic
chr19	32271122	32273804	Distal Intergenic
chr19	32284547	32286553	Distal Intergenic
chr19	32338205	32339007	Distal Intergenic
chr19	32341098	32341376	Distal Intergenic
chr19	32341441	32342142	Distal Intergenic
chr19	32346874	32349106	Distal Intergenic
chr19	32349151	32349357	Distal Intergenic
chr19	32359969	32360187	Distal Intergenic
chr19	32365510	32367105	Distal Intergenic
chr19	32378196	32381016	Distal Intergenic
chr19	32390662	32391207	Distal Intergenic
chr19	32391779	32391991	Distal Intergenic
chr19	32420526	32421299	Distal Intergenic
chr19	32463216	32463542	Distal Intergenic
chr19	32468304	32468733	Distal Intergenic
chr19	32591680	32592055	Distal Intergenic
chr19	32592174	32593272	Distal Intergenic
chr19	32598136	32598503	Distal Intergenic
chr19	32598591	32598851	Distal Intergenic
chr19	32747592	32747816	Distal Intergenic
chr19	32747987	32748470	Distal Intergenic
chr19	32748653	32748852	Distal Intergenic
chr19	32750084	32750961	Distal Intergenic
chr19	32786051	32786797	Distal Intergenic
chr19	32786907	32787288	Distal Intergenic
chr19	32830300	32830935	Distal Intergenic

chr19	32831519	32831834	Distal Intergenic
chr19	32832227	32832731	Distal Intergenic
chr19	32833258	32835268	Distal Intergenic
chr19	33048809	33049179	Distal Intergenic
chr19	34096013	34096236	Distal Intergenic
chr19	34096309	34097013	Distal Intergenic
chr19	34116911	34117646	Intron
chr19	34117764	34118102	Intron
chr19	34125774	34127512	Intron
chr19	34132401	34133093	Intron
chr19	34173238	34173461	Intron
chr19	34193001	34195806	Intron
chr19	34238449	34241171	Intron
chr19	34253861	34254080	Intron
chr19	34437486	34438082	Distal Intergenic
chr19	34483351	34483875	Distal Intergenic
chr19	34484097	34484339	Distal Intergenic
chr19	34484710	34485217	Distal Intergenic
chr19	34485499	34485730	Distal Intergenic
chr19	34498647	34498906	Distal Intergenic
chr19	34516008	34517355	Distal Intergenic
chr19	34519660	34520909	Distal Intergenic
chr19	34522458	34522698	Distal Intergenic
chr19	34522899	34524191	Distal Intergenic
chr19	34524862	34525380	Distal Intergenic
chr19	34525427	34525797	Distal Intergenic
chr19	34529010	34530287	Distal Intergenic
chr19	34760540	34760739	Intron
chr19	34934454	34934929	Exon
chr19	34952021	34952955	Intron
chr19	34953382	34953604	Intron
chr19	34953698	34954053	Intron
chr19	34996543	34997472	Distal Intergenic
chr19	35201331	35202711	Distal Intergenic
chr19	36157622	36158375	Promoter
chr19	36203842	36204089	Promoter
chr19	36436830	36437087	Distal Intergenic
chr19	36525317	36526118	3' UTR
chr19	36832237	36832772	Exon
chr19	36907207	36908726	Promoter
chr19	36908794	36910527	Promoter
chr19	36990748	36993343	Distal Intergenic
chr19	36993420	36993619	Distal Intergenic
chr19	36999852	37000502	Downstream

chr19	37000618	37001931	3' UTR
chr19	37002003	37002240	3' UTR
chr19	37027057	37027545	Distal Intergenic
chr19	37174744	37178317	Promoter
chr19	37179503	37180781	Promoter
chr19	37281991	37283645	Distal Intergenic
chr19	37338601	37339329	Intron
chr19	37339452	37339675	Intron
chr19	37407202	37408447	Promoter
chr19	37449870	37451262	Intron
chr19	38030044	38031892	3' UTR
chr19	38033182	38035707	3' UTR
chr19	38035786	38035986	Distal Intergenic
chr19	38040191	38041114	Promoter
chr19	38044496	38045155	Intron
chr19	38059595	38061033	Exon
chr19	38062720	38063172	Intron
chr19	38071306	38071722	Intron
chr19	38089043	38091574	5' UTR
chr19	38091929	38092227	Exon
chr19	38097269	38098135	Intron
chr19	38098801	38099909	Intron
chr19	38099959	38101727	Intron
chr19	38106799	38108301	Distal Intergenic
chr19	38112145	38113735	Distal Intergenic
chr19	38116638	38117881	Distal Intergenic
chr19	38129470	38130063	Intron
chr19	38130387	38130907	Intron
chr19	39005584	39005934	Exon
chr19	39005981	39006278	Intron
chr19	40305689	40306597	Distal Intergenic
chr19	40316251	40316454	3' UTR
chr19	40316619	40317361	Exon
chr19	40321374	40321778	Exon
chr19	40321883	40322720	5' UTR
chr19	40324265	40324479	Promoter
chr19	40324691	40324890	Promoter
chr19	40327651	40333892	Promoter
chr19	40338335	40339340	Distal Intergenic
chr19	40341236	40345823	Distal Intergenic
chr19	40570003	40571046	Distal Intergenic
chr19	40626534	40627352	Distal Intergenic
chr19	40629128	40630296	Distal Intergenic
chr19	40700694	40700980	Intron

chr19	40956152	40956544	Intron
chr19	40968969	40969836	Intron
chr19	41050787	41051045	Intron
chr19	41060823	41061681	3' UTR
chr19	41063974	41065324	Intron
chr19	41067938	41071026	Intron
chr19	41072740	41073142	Intron
chr19	41337790	41338071	Distal Intergenic
chr19	41338167	41338403	Distal Intergenic
chr19	41338545	41338766	Distal Intergenic
chr19	41569703	41569902	Distal Intergenic
chr19	41569938	41570165	Distal Intergenic
chr19	41821489	41822989	Exon
chr19	41840451	41842017	Intron
chr19	41847095	41847718	Intron
chr19	41931849	41932054	Exon
chr19	41970083	41970487	Intron
chr19	41970563	41970891	Intron
chr19	41980391	41982242	Intron
chr19	41985840	41986291	Intron
chr19	41986525	41988908	Intron
chr19	42008048	42008575	Distal Intergenic
chr19	42067492	42067832	Intron
chr19	42068414	42068711	Intron
chr19	42090532	42092770	Exon
chr19	42092876	42093282	3' UTR
chr19	42096129	42097612	Distal Intergenic
chr19	42154198	42154648	Distal Intergenic
chr19	42173526	42177339	3' UTR
chr19	42194676	42195347	Distal Intergenic
chr19	42199609	42206403	Distal Intergenic
chr19	42209772	42210953	Distal Intergenic
chr19	42222327	42223557	Intron
chr19	42226058	42226331	Intron
chr19	42241004	42242395	Distal Intergenic
chr19	42324492	42325290	Distal Intergenic
chr19	42661881	42662182	Distal Intergenic
chr19	42662235	42663059	Distal Intergenic
chr19	42663120	42663343	Distal Intergenic
chr19	42672663	42673262	Distal Intergenic
chr19	42687733	42687968	Distal Intergenic
chr19	42706021	42707127	5' UTR
chr19	42734225	42735069	3' UTR
chr19	42838791	42839060	Intron

chr19	42839101	42839654	Exon
chr19	43573057	43574257	Intron
chr19	43605221	43605550	Intron
chr19	43625390	43626351	Intron
chr19	43743395	43744036	Intron
chr19	43750127	43750528	Intron
chr19	43785459	43786237	Distal Intergenic
chr19	43826857	43827469	Distal Intergenic
chr19	43987408	43987641	Intron
chr19	43987808	43988511	Intron
chr19	43990775	43992051	Exon
chr19	43992091	43992388	Intron
chr19	43995758	43996350	Intron
chr19	43996560	43996959	Intron
chr19	44012616	44014187	Exon
chr19	44043571	44043799	Distal Intergenic
chr19	44044000	44044261	Distal Intergenic
chr19	44126312	44127512	3' UTR
chr19	44170187	44170608	Intron
chr19	44181918	44182276	Distal Intergenic
chr19	44210118	44210317	Distal Intergenic
chr19	44220121	44220639	Promoter
chr19	44322458	44322677	Intron
chr19	44322867	44323402	Intron
chr19	44355339	44356068	Distal Intergenic
chr19	44482444	44482849	Distal Intergenic
chr19	44752370	44753073	Intron
chr19	44753254	44755635	Intron
chr19	44755855	44756143	Intron
chr19	44783238	44783609	Intron
chr19	44783667	44784413	Intron
chr19	44829523	44829813	Downstream
chr19	44856274	44857404	Intron
chr19	44973588	44974256	Distal Intergenic
chr19	45000779	45001737	5' UTR
chr19	45005684	45005888	Distal Intergenic
chr19	45080962	45081566	Distal Intergenic
chr19	45122065	45123145	Intron
chr19	45129958	45130159	Intron
chr19	45132214	45133987	Intron
chr19	45148999	45149369	Intron
chr19	45250779	45251228	Promoter
chr19	45283775	45284949	Exon
chr19	45726509	45726726	Intron

chr19	45729783	45731181	Exon
chr19	45733375	45735001	Intron
chr19	45856762	45857531	Intron
chr19	45857787	45858088	Exon
chr19	45864899	45865316	Promoter
chr19	45911100	45912372	3' UTR
chr19	45933759	45936467	Intron
chr19	46072667	46073049	Intron
chr19	46077068	46077712	Intron
chr19	46099419	46099919	Intron
chr19	46112060	46114062	3' UTR
chr19	46114215	46114414	Intron
chr19	46131039	46131998	Intron
chr19	46150658	46150904	Distal Intergenic
chr19	46378836	46379042	Distal Intergenic
chr19	46379241	46379749	Distal Intergenic
chr19	46379872	46380114	Distal Intergenic
chr19	46426366	46427308	Distal Intergenic
chr19	46464751	46465726	Intron
chr19	46465825	46466069	Intron
chr19	46468629	46468882	Intron
chr19	46469782	46470231	Intron
chr19	46522918	46523204	Promoter
chr19	46553487	46555156	Distal Intergenic
chr19	46557657	46560219	Distal Intergenic
chr19	46561806	46562022	Exon
chr19	46562199	46563276	Exon
chr19	46563421	46565046	Exon
chr19	46574684	46575157	Intron
chr19	46575508	46575721	Intron
chr19	46577385	46577641	Intron
chr19	46577901	46579011	Intron
chr19	46579273	46579535	Intron
chr19	46595895	46596526	Distal Intergenic
chr19	46596674	46596941	Distal Intergenic
chr19	46603663	46604455	Distal Intergenic
chr19	46673586	46674722	Distal Intergenic
chr19	46674802	46675047	Distal Intergenic
chr19	46694708	46695022	Intron
chr19	46695231	46695572	Intron
chr19	46881264	46881619	Intron
chr19	46967139	46967860	Downstream
chr19	47035333	47035583	Intron
chr19	47035970	47036453	Intron

chr19	47059528	47059982	Intron
chr19	47062362	47062586	Intron
chr19	47141735	47142107	Distal Intergenic
chr19	47164153	47164393	Promoter
chr19	47164557	47165743	Promoter
chr19	47211555	47213349	Promoter
chr19	47251430	47251748	Intron
chr19	47251818	47252486	5' UTR
chr19	47252635	47253332	Intron
chr19	47293487	47295796	Distal Intergenic
chr19	47295853	47296122	Distal Intergenic
chr19	47306295	47308080	Distal Intergenic
chr19	47323868	47324119	Distal Intergenic
chr19	47324377	47324841	Distal Intergenic
chr19	47355510	47357533	Distal Intergenic
chr19	47357600	47357799	Distal Intergenic
chr19	47385181	47385389	Distal Intergenic
chr19	47385529	47391199	Distal Intergenic
chr19	47402652	47406660	Distal Intergenic
chr19	47420388	47420782	Distal Intergenic
chr19	47517712	47518254	Distal Intergenic
chr19	47565049	47566170	Downstream
chr19	47566253	47569870	3' UTR
chr19	47574390	47576485	Exon
chr19	47581827	47582111	Intron
chr19	47582561	47585813	Exon
chr19	47589726	47590172	Exon
chr19	47745347	47746551	Distal Intergenic
chr19	47762064	47762524	Intron
chr19	47762750	47767115	Exon
chr19	47771818	47775192	3' UTR
chr19	47781837	47782036	Distal Intergenic
chr19	47782135	47783404	Distal Intergenic
chr19	47784121	47785980	Distal Intergenic
chr19	47800456	47800861	Distal Intergenic
chr19	47803205	47805782	Distal Intergenic
chr19	47909146	47910033	3' UTR
chr19	47918037	47919291	Promoter
chr19	47926612	47927097	Distal Intergenic
chr19	47928220	47931181	Downstream
chr19	47931220	47933287	3' UTR
chr19	47943974	47945007	Exon
chr19	47974607	47976372	Promoter
chr19	47977034	47977901	Downstream

chr19	47983096	47983295	Exon
chr19	47990714	47991711	3' UTR
chr19	47991966	47992279	Intron
chr19	47992420	47994530	Exon
chr19	48002524	48004485	Exon
chr19	48005615	48006865	Exon
chr19	50996014	50996466	Distal Intergenic
chr19	51019476	51019719	Downstream
chr19	51041827	51042352	Intron
chr19	52191837	52192354	Distal Intergenic
chr19	52192648	52192847	Distal Intergenic
chr19	52314095	52314328	Intron
chr19	52707163	52707712	Intron
chr19	53096085	53096461	Distal Intergenic
chr19	53112646	53113085	Downstream
chr19	53113208	53113707	Downstream
chr19	53114613	53116121	3' UTR
chr19	53353972	53354312	Intron
chr19	53354507	53354706	Intron
chr19	53358068	53359306	Intron
chr19	53464129	53465058	Intron
chr19	53465146	53465816	Promoter
chr19	53486396	53487295	Intron
chr19	53495034	53495517	Intron
chr19	53495610	53496493	Promoter
chr19	53523941	53526125	Distal Intergenic
chr19	53605970	53606395	Promoter
chr19	53674643	53674842	Intron
chr19	53806431	53807022	Distal Intergenic
chr19	53969312	53970090	Promoter
chr19	54017968	54018727	Distal Intergenic
chr19	54019384	54019586	Distal Intergenic
chr19	55057084	55057302	Distal Intergenic
chr19	55057441	55057691	Distal Intergenic
chr19	55295726	55296147	3' UTR
chr19	55306416	55307070	Intron
chr19	55327807	55329993	Promoter
chr19	55331685	55333098	Exon
chr19	55341362	55341663	Exon
chr19	55497376	55498879	Exon
chr19	55537795	55538342	Intron
chr19	55616365	55617138	Intron
chr19	55617176	55617788	Intron
chr19	55644632	55646391	Exon

chr19	55786666	55787912	Intron
chr19	55788110	55788534	Intron
chr19	55807192	55807598	Intron
chr19	55869375	55869645	3' UTR
chr19	55869851	55870500	3' UTR
chr19	56070083	56070982	Distal Intergenic
chr19	56112113	56112353	Promoter
chr19	56471795	56472407	Intron
chr19	56525360	56526749	Intron
chr19	56622242	56622618	Intron
chr19	56622670	56623214	Intron
chr19	56623321	56624207	Intron
chr19	56645766	56646420	Distal Intergenic
chr19	56653711	56654738	Intron
chr19	56662493	56663522	Intron
chr19	56674350	56677916	Distal Intergenic
chr19	56778235	56778521	Intron
chr19	56798328	56798543	Intron
chr19	56885462	56885742	Intron
chr19	56960352	56960610	Intron
chr19	56980098	56980638	Intron
chr19	56989182	56989943	Promoter
chr19	57009002	57011854	Distal Intergenic
chr19	57033060	57034546	Intron
chr19	57051584	57053110	Intron
chr19	57055072	57055437	Intron
chr19	57064210	57066043	3' UTR
chr19	57067517	57070506	3' UTR
chr19	57076346	57077600	Intron
chr19	57081137	57082651	5' UTR
chr19	57089096	57090492	3' UTR
chr19	57130046	57130699	Intron
chr19	57181726	57181986	Intron
chr19	57191656	57192736	Distal Intergenic
chr19	57192790	57193532	Distal Intergenic
chr19	57255546	57258308	Intron
chr19	57272457	57273077	Intron
chr19	57312675	57314226	Intron
chr19	57315827	57316519	Intron
chr19	57316605	57316935	Intron
chr19	57347610	57347888	Promoter
chr19	57347949	57348368	Promoter
chr19	57426182	57427476	Distal Intergenic
chr19	57436098	57436773	Distal Intergenic

chr19	57438541	57439526	Distal Intergenic
chr19	57680738	57681075	Distal Intergenic
chr19	57801502	57801895	Intron
chr19	58178959	58179726	Promoter
chr19	58295113	58295887	Intron
chr19	58596217	58598813	3' UTR
chr19	59145837	59146270	Distal Intergenic
chr19	59420013	59420283	Distal Intergenic
chr19	59474766	59475361	Distal Intergenic
chr19	59532986	59533318	Distal Intergenic
chr19	59661767	59662384	Distal Intergenic
chr19	59919528	59923299	Distal Intergenic
chr19	59933260	59933658	Distal Intergenic
chr19	59937436	59938977	Distal Intergenic
chr19	59954580	59956215	Distal Intergenic
chr19	59956314	59956608	Distal Intergenic
chr19	59962424	59963451	Distal Intergenic
chr19	59963751	59964090	Distal Intergenic
chr19	59964521	59965280	Distal Intergenic
chr19	59973085	59974838	Distal Intergenic
chr19	60018835	60019174	Distal Intergenic
chr19	60116321	60118271	Distal Intergenic
chr19	60655790	60656139	Distal Intergenic
chr19	60657109	60657870	Distal Intergenic
chr19	60692294	60692799	Distal Intergenic
chr19	60692960	60693589	Distal Intergenic
chr19	60862265	60862920	Distal Intergenic
chr19	60864734	60865335	Distal Intergenic
chr19	60865520	60866032	Distal Intergenic
chr19	60866172	60866933	Distal Intergenic
chr19	60950051	60950420	Distal Intergenic
chr19	61031794	61032187	Distal Intergenic
chr19	61283138	61283426	Distal Intergenic
chr2	3058856	3059101	Intron
chr2	3134944	3135421	Distal Intergenic
chr2	3154728	3155112	Distal Intergenic
chr2	3156014	3157050	Distal Intergenic
chr2	3248204	3248536	Intron
chr2	3248738	3249371	Intron
chr2	3249522	3249879	Intron
chr2	3370167	3370376	Intron
chr2	3629473	3630634	Distal Intergenic
chr2	3630689	3630938	Distal Intergenic
chr2	3643806	3644444	Intron

chr2	3665410	3667172	Intron
chr2	3670909	3671294	Intron
chr2	3677197	3677412	Intron
chr2	3677460	3678324	Intron
chr2	3702966	3703271	Distal Intergenic
chr2	4018786	4019039	Intron
chr2	4183402	4184071	Distal Intergenic
chr2	4342084	4342550	Distal Intergenic
chr2	4366451	4367343	Distal Intergenic
chr2	4427670	4428059	Distal Intergenic
chr2	4480090	4481002	Distal Intergenic
chr2	4496276	4496587	Distal Intergenic
chr2	4572777	4573147	Distal Intergenic
chr2	4803281	4803634	Distal Intergenic
chr2	4826310	4826552	Distal Intergenic
chr2	5207119	5207638	Distal Intergenic
chr2	5534965	5536075	Distal Intergenic
chr2	5765801	5766752	Distal Intergenic
chr2	5766888	5767087	Distal Intergenic
chr2	5809744	5811267	Distal Intergenic
chr2	5871272	5872129	Distal Intergenic
chr2	5872442	5872777	Distal Intergenic
chr2	5879322	5879533	Distal Intergenic
chr2	5879620	5879831	Distal Intergenic
chr2	5975685	5975897	Distal Intergenic
chr2	6000689	6003397	Distal Intergenic
chr2	6038558	6040244	Distal Intergenic
chr2	6049955	6050678	Distal Intergenic
chr2	6050873	6051113	Distal Intergenic
chr2	6083315	6085571	Intron
chr2	6089122	6089444	Intron
chr2	6089498	6090114	Intron
chr2	6134087	6134371	Distal Intergenic
chr2	6269970	6271511	Distal Intergenic
chr2	6273705	6274073	Distal Intergenic
chr2	6279307	6280585	Distal Intergenic
chr2	6280896	6281195	Distal Intergenic
chr2	6287510	6287831	Distal Intergenic
chr2	6299803	6300233	Distal Intergenic
chr2	6300459	6301495	Distal Intergenic
chr2	6468898	6471281	Distal Intergenic
chr2	6490405	6491685	Distal Intergenic
chr2	6503638	6504140	Distal Intergenic
chr2	6514615	6516467	Distal Intergenic

chr2	7456372	7456636	Distal Intergenic
chr2	8197240	8197480	Intron
chr2	8197521	8198761	Intron
chr2	8702570	8703993	Distal Intergenic
chr2	9062989	9063615	Intron
chr2	9403315	9403760	Intron
chr2	9406956	9407550	Intron
chr2	9408739	9409273	Intron
chr2	9654834	9655454	Intron
chr2	9712902	9714595	Distal Intergenic
chr2	9716985	9717297	Distal Intergenic
chr2	9724743	9724992	3' UTR
chr2	9725059	9725269	3' UTR
chr2	9757028	9757308	Intron
chr2	9788618	9789063	Distal Intergenic
chr2	9800351	9801214	Distal Intergenic
chr2	9877476	9878066	Distal Intergenic
chr2	10001532	10001735	Intron
chr2	10001838	10002056	Intron
chr2	10008665	10009524	Intron
chr2	10502669	10502930	Intron
chr2	11260330	11261200	Exon
chr2	11298965	11299196	Intron
chr2	11316745	11316958	Intron
chr2	11339425	11339637	Intron
chr2	11351003	11351408	Intron
chr2	11379510	11380822	Intron
chr2	11385965	11388544	Intron
chr2	11524109	11524786	Distal Intergenic
chr2	11545332	11545695	Distal Intergenic
chr2	11545747	11545978	Distal Intergenic
chr2	11597790	11598253	Intron
chr2	11626689	11629528	Distal Intergenic
chr2	11629588	11631583	Distal Intergenic
chr2	11975056	11975502	Downstream
chr2	11998981	11999180	Distal Intergenic
chr2	12333821	12334271	Intron
chr2	12340144	12340720	Intron
chr2	12340854	12341064	Intron
chr2	12686317	12686546	Intron
chr2	12687816	12688087	Intron
chr2	12845838	12846220	Distal Intergenic
chr2	13061337	13063283	Distal Intergenic
chr2	13292999	13293746	Distal Intergenic

chr2	13294017	13294569	Distal Intergenic
chr2	13326261	13327748	Distal Intergenic
chr2	13328839	13330019	Distal Intergenic
chr2	13330056	13330261	Distal Intergenic
chr2	13330928	13331976	Distal Intergenic
chr2	13332038	13332499	Distal Intergenic
chr2	13372404	13372914	Distal Intergenic
chr2	13478932	13479421	Distal Intergenic
chr2	13994760	13997261	Distal Intergenic
chr2	14970485	14971237	Distal Intergenic
chr2	14976215	14976486	Distal Intergenic
chr2	14976593	14976799	Distal Intergenic
chr2	14976916	14977726	Distal Intergenic
chr2	14977808	14978063	Distal Intergenic
chr2	14981910	14982748	Distal Intergenic
chr2	15827810	15828030	Distal Intergenic
chr2	16090071	16090832	Distal Intergenic
chr2	16135729	16136093	Distal Intergenic
chr2	16327528	16329638	Distal Intergenic
chr2	16367280	16367673	Distal Intergenic
chr2	17570188	17570395	Distal Intergenic
chr2	17663075	17664643	Distal Intergenic
chr2	17766931	17767437	Intron
chr2	17949289	17950553	Exon
chr2	17977825	17978194	Intron
chr2	17978567	17979554	Intron
chr2	17985335	17986082	Distal Intergenic
chr2	17986202	17986861	Distal Intergenic
chr2	17987144	17987648	Distal Intergenic
chr2	18038132	18038363	Distal Intergenic
chr2	18056225	18056996	Distal Intergenic
chr2	18135663	18136000	Distal Intergenic
chr2	18313720	18313992	Distal Intergenic
chr2	18314034	18314432	Distal Intergenic
chr2	18332978	18334235	Distal Intergenic
chr2	18593563	18593826	Distal Intergenic
chr2	18594096	18595108	Distal Intergenic
chr2	18615689	18616132	Distal Intergenic
chr2	18687380	18689278	Distal Intergenic
chr2	18849718	18850377	Distal Intergenic
chr2	19328406	19329332	Distal Intergenic
chr2	19462326	19462860	Distal Intergenic
chr2	19500263	19502755	Distal Intergenic
chr2	19527274	19527794	Distal Intergenic

chr2	19527830	19528419	Distal Intergenic
chr2	19579584	19579816	Distal Intergenic
chr2	19580398	19580689	Distal Intergenic
chr2	19580740	19581093	Distal Intergenic
chr2	19604797	19605155	Distal Intergenic
chr2	19606987	19607321	Distal Intergenic
chr2	19624655	19624985	Distal Intergenic
chr2	20440979	20443628	Distal Intergenic
chr2	20468206	20468427	Intron
chr2	20468476	20472340	Intron
chr2	20476788	20478042	Promoter
chr2	20480929	20482538	Intron
chr2	20482798	20484489	Exon
chr2	20484657	20485072	Intron
chr2	20518349	20519843	5' UTR
chr2	20519991	20521549	Intron
chr2	20521662	20522765	Intron
chr2	20522870	20523128	Intron
chr2	20538314	20538528	Intron
chr2	20540135	20542466	Intron
chr2	20563195	20563436	Distal Intergenic
chr2	20563599	20565402	Distal Intergenic
chr2	20573621	20577209	Distal Intergenic
chr2	20577549	20577917	Distal Intergenic
chr2	20578087	20578350	Distal Intergenic
chr2	20579856	20580083	Distal Intergenic
chr2	20581748	20585520	Distal Intergenic
chr2	20585601	20585961	Distal Intergenic
chr2	20586113	20586816	Distal Intergenic
chr2	20586987	20588605	Distal Intergenic
chr2	20612229	20613230	Distal Intergenic
chr2	20648615	20650153	3' UTR
chr2	20657315	20659316	Distal Intergenic
chr2	20673742	20676140	Distal Intergenic
chr2	20794720	20795968	Distal Intergenic
chr2	20865073	20865537	Promoter
chr2	20865573	20866104	Promoter
chr2	20870332	20870546	Exon
chr2	20870830	20871173	Exon
chr2	20888517	20891327	Intron
chr2	20891364	20891587	Intron
chr2	20951026	20953195	Intron
chr2	20958902	20959221	Intron
chr2	20975223	20976533	Intron

chr2	20986251	20987259	Intron
chr2	21103958	21105048	Distal Intergenic
chr2	21155841	21156275	Distal Intergenic
chr2	21156331	21159317	Distal Intergenic
chr2	21161496	21161808	Distal Intergenic
chr2	21220966	21221487	Downstream
chr2	21444463	21444712	Distal Intergenic
chr2	21864273	21865758	Distal Intergenic
chr2	22579337	22580110	Distal Intergenic
chr2	22861477	22862448	Distal Intergenic
chr2	22862607	22862870	Distal Intergenic
chr2	22863495	22863743	Distal Intergenic
chr2	22870367	22870889	Distal Intergenic
chr2	22876991	22878134	Distal Intergenic
chr2	22878239	22878463	Distal Intergenic
chr2	22889914	22892469	Distal Intergenic
chr2	22894222	22895818	Distal Intergenic
chr2	22923001	22924761	Distal Intergenic
chr2	22924931	22925191	Distal Intergenic
chr2	23010636	23011489	Distal Intergenic
chr2	23011583	23012433	Distal Intergenic
chr2	23426950	23427179	Distal Intergenic
chr2	23427369	23427568	Distal Intergenic
chr2	23929440	23930513	3' UTR
chr2	24041541	24042219	Intron
chr2	24070280	24070479	Intron
chr2	24123015	24123214	Intron
chr2	24146532	24146732	Intron
chr2	24149626	24151416	Promoter
chr2	24172772	24172971	Intron
chr2	24191856	24192491	Exon
chr2	24197160	24197365	Intron
chr2	24223261	24223941	3' UTR
chr2	24240186	24243074	Exon
chr2	24262908	24264354	Intron
chr2	24294194	24294432	Intron
chr2	24294482	24296277	Intron
chr2	24308383	24309470	Promoter
chr2	24418099	24419406	Distal Intergenic
chr2	24761546	24762013	Distal Intergenic
chr2	24762169	24762694	Distal Intergenic
chr2	24774181	24774386	Distal Intergenic
chr2	24783260	24784520	Distal Intergenic
chr2	24784573	24785176	Distal Intergenic

chr2	24830702	24830938	Intron
chr2	25028108	25028422	Intron
chr2	25031807	25032740	Intron
chr2	25035090	25036008	Intron
chr2	25051214	25053120	Intron
chr2	25053243	25053449	Intron
chr2	25066450	25066906	Intron
chr2	25079189	25080083	Intron
chr2	25080916	25081444	Intron
chr2	25082972	25083962	Intron
chr2	25092883	25094309	Intron
chr2	25095411	25095660	Exon
chr2	25110360	25111185	Intron
chr2	25111636	25111854	Intron
chr2	25117764	25117963	Intron
chr2	25121132	25121910	Intron
chr2	25141278	25143511	Promoter
chr2	25187782	25188074	Intron
chr2	25316556	25316838	Intron
chr2	25356321	25356897	Intron
chr2	25357938	25358172	Intron
chr2	25358316	25358520	Exon
chr2	25389298	25390744	Promoter
chr2	25401861	25402077	Distal Intergenic
chr2	25430022	25430404	Distal Intergenic
chr2	25430499	25430746	Distal Intergenic
chr2	25463363	25463670	Exon
chr2	25463814	25464074	Intron
chr2	25512716	25513325	Intron
chr2	25516941	25517495	Intron
chr2	25831083	25831334	Intron
chr2	25841757	25845080	Intron
chr2	25974454	25974776	Intron
chr2	25988641	25989470	Intron
chr2	25999661	26000380	Intron
chr2	26085665	26085864	Intron
chr2	26086423	26087883	Intron
chr2	26090949	26091224	Intron
chr2	26107987	26108282	Distal Intergenic
chr2	26108531	26108861	Distal Intergenic
chr2	26167517	26167975	Intron
chr2	26206419	26206885	Promoter
chr2	26206939	26207575	Distal Intergenic
chr2	26244964	26245366	Distal Intergenic

chr2	26300278	26300769	Intron
chr2	26301467	26301872	Intron
chr2	26302119	26302323	Intron
chr2	26356208	26359619	3' UTR
chr2	26361466	26364050	Distal Intergenic
chr2	26387108	26387510	Distal Intergenic
chr2	26387557	26387757	Distal Intergenic
chr2	26403569	26404068	Promoter
chr2	26406211	26412987	3' UTR
chr2	26494244	26496520	Exon
chr2	26498944	26499388	Intron
chr2	26757702	26759347	Intron
chr2	26765293	26765803	Intron
chr2	26765965	26766971	Intron
chr2	26769861	26770446	Intron
chr2	26770723	26770929	Intron
chr2	26771042	26772538	Intron
chr2	26788285	26788927	Intron
chr2	26819383	26820497	Intron
chr2	26865816	26866038	Distal Intergenic
chr2	27173715	27174285	Distal Intergenic
chr2	27182467	27182666	Distal Intergenic
chr2	27220311	27220610	Intron
chr2	27272007	27272369	Distal Intergenic
chr2	27319253	27319504	Intron
chr2	27319663	27319987	Exon
chr2	27360259	27362136	Promoter
chr2	27371109	27371324	Promoter
chr2	27408417	27409389	Distal Intergenic
chr2	27409601	27409914	Distal Intergenic
chr2	27410012	27410231	Distal Intergenic
chr2	27486204	27487245	Promoter
chr2	27527541	27527777	Intron
chr2	27531868	27532195	Promoter
chr2	27533355	27533640	Intron
chr2	27534550	27534796	Exon
chr2	27535183	27536614	Exon
chr2	27557262	27560482	Promoter
chr2	27569098	27571960	Intron
chr2	27572121	27572384	Intron
chr2	27572827	27575453	5' UTR
chr2	27575498	27578117	Intron
chr2	27590085	27590486	3' UTR
chr2	27595456	27595856	5' UTR

chr2	27598792	27598991	Exon
chr2	27599083	27600795	3' UTR
chr2	27600903	27602081	Exon
chr2	27613601	27615155	Intron
chr2	28372974	28373461	Intron
chr2	28379178	28379414	Intron
chr2	28379453	28380078	Intron
chr2	28380496	28380695	Intron
chr2	28380757	28381884	Intron
chr2	28384837	28385746	Intron
chr2	28438329	28438762	Intron
chr2	28496517	28496727	Intron
chr2	28695674	28696060	Distal Intergenic
chr2	28696176	28696408	Distal Intergenic
chr2	28696533	28697064	Distal Intergenic
chr2	28915685	28915979	Distal Intergenic
chr2	28916042	28916257	Distal Intergenic
chr2	28920536	28920997	Distal Intergenic
chr2	28921173	28922129	Distal Intergenic
chr2	28944087	28945379	Distal Intergenic
chr2	28956165	28956908	Distal Intergenic
chr2	29090809	29091310	Intron
chr2	29091414	29091644	Intron
chr2	29278011	29278843	Distal Intergenic
chr2	29381582	29382308	Intron
chr2	29386475	29386677	Intron
chr2	29386962	29387235	Intron
chr2	29468966	29469222	Intron
chr2	29474228	29475128	Intron
chr2	29475529	29475875	Intron
chr2	29476040	29476617	Intron
chr2	29480019	29480369	Intron
chr2	29480447	29480686	Intron
chr2	29483533	29483980	Intron
chr2	29484516	29485097	Intron
chr2	29485160	29486863	Intron
chr2	29487644	29489043	Intron
chr2	29526169	29526729	Intron
chr2	29546515	29548054	Intron
chr2	29550397	29552630	Exon
chr2	29561249	29561628	Intron
chr2	29566299	29569348	Intron
chr2	29571681	29573083	Intron
chr2	29584712	29585725	Intron

chr2	29627107	29627899	Intron
chr2	29682136	29682525	Intron
chr2	29682636	29683170	Intron
chr2	29745719	29746137	Intron
chr2	29816136	29817685	Intron
chr2	29819702	29820821	Intron
chr2	29822077	29824868	Intron
chr2	29827462	29828831	Intron
chr2	29917625	29919707	Exon
chr2	29921318	29921537	Intron
chr2	29922109	29922502	Intron
chr2	29922544	29923450	Intron
chr2	29988860	29989068	Intron
chr2	30092280	30093135	Intron
chr2	30093743	30094015	Intron
chr2	30105926	30108019	Intron
chr2	30113094	30115466	Intron
chr2	30121223	30122312	Intron
chr2	30142101	30142360	Intron
chr2	30142636	30142941	Exon
chr2	30178307	30179938	Distal Intergenic
chr2	30180463	30181738	Distal Intergenic
chr2	30206189	30207151	Distal Intergenic
chr2	30240975	30241694	Distal Intergenic
chr2	30295536	30298799	Distal Intergenic
chr2	30298867	30299109	Distal Intergenic
chr2	30299369	30299573	Distal Intergenic
chr2	30300112	30301840	Distal Intergenic
chr2	30318859	30320015	Distal Intergenic
chr2	30325579	30326758	Distal Intergenic
chr2	30326891	30327564	Distal Intergenic
chr2	30327627	30328095	Distal Intergenic
chr2	30403511	30404095	Distal Intergenic
chr2	30555523	30555907	Distal Intergenic
chr2	30562816	30563126	Distal Intergenic
chr2	30563162	30563632	Distal Intergenic
chr2	30563755	30565529	Distal Intergenic
chr2	30567036	30567272	Distal Intergenic
chr2	30567836	30568956	Distal Intergenic
chr2	30574009	30574970	Exon
chr2	30575103	30575398	Exon
chr2	30575520	30575936	Distal Intergenic
chr2	30663508	30663844	Distal Intergenic
chr2	30753033	30753294	Intron

chr2	30753369	30753576	Intron
chr2	30754445	30755506	Intron
chr2	30757397	30758504	Intron
chr2	30773055	30774575	Intron
chr2	30774669	30776322	5' UTR
chr2	30783909	30786223	5' UTR
chr2	30788336	30789696	Intron
chr2	30898030	30899190	Distal Intergenic
chr2	30978870	30979547	Intron
chr2	31007455	31007695	Intron
chr2	31007812	31009104	Intron
chr2	31017931	31021558	Intron
chr2	31023115	31024753	Intron
chr2	31027455	31027870	Intron
chr2	31118970	31120595	Distal Intergenic
chr2	31297789	31298899	Intron
chr2	31309059	31310810	Intron
chr2	31325894	31326699	Intron
chr2	31379784	31380007	Distal Intergenic
chr2	31397456	31397719	3' UTR
chr2	31427980	31428241	5' UTR
chr2	31526286	31526512	Distal Intergenic
chr2	31543818	31544483	Distal Intergenic
chr2	31615217	31616781	Intron
chr2	31625007	31627163	Exon
chr2	31790258	31790672	Intron
chr2	31830070	31830377	Distal Intergenic
chr2	31862310	31863996	Distal Intergenic
chr2	31993220	31993432	Distal Intergenic
chr2	32007149	32008367	Distal Intergenic
chr2	32008414	32008861	Distal Intergenic
chr2	32016055	32016651	Distal Intergenic
chr2	32020200	32021016	Distal Intergenic
chr2	32022519	32023550	Distal Intergenic
chr2	32029137	32031494	Distal Intergenic
chr2	32037139	32037470	Distal Intergenic
chr2	32037515	32039503	Distal Intergenic
chr2	32091483	32091996	Downstream
chr2	32092116	32092433	Downstream
chr2	32180759	32180958	Intron
chr2	32197653	32198389	Intron
chr2	32198519	32198899	Intron
chr2	32218730	32219267	Intron
chr2	32278591	32280734	Distal Intergenic

chr2	32283309	32284374	Distal Intergenic
chr2	32292498	32292761	Intron
chr2	32326259	32326835	Intron
chr2	32426403	32426960	Intron
chr2	32483863	32485321	Intron
chr2	32535330	32538031	Distal Intergenic
chr2	32541187	32542170	Distal Intergenic
chr2	32567431	32568121	Distal Intergenic
chr2	32569758	32569966	Distal Intergenic
chr2	32570436	32571192	Distal Intergenic
chr2	32576085	32576294	Distal Intergenic
chr2	32630839	32631097	Intron
chr2	32631369	32631731	Exon
chr2	32690160	32690555	Exon
chr2	32722590	32722981	Intron
chr2	32723026	32723688	Intron
chr2	32725167	32726744	Exon
chr2	32730519	32736016	Exon
chr2	32736167	32738472	Exon
chr2	32738557	32738955	Intron
chr2	32750025	32750583	Exon
chr2	32751390	32753297	Intron
chr2	32753512	32753832	Intron
chr2	32754402	32754654	Intron
chr2	32757638	32758564	Promoter
chr2	32772944	32773168	Exon
chr2	32817259	32818615	Intron
chr2	32818692	32820598	Exon
chr2	32821140	32821348	Intron
chr2	32821387	32823088	Exon
chr2	32883235	32883521	Intron
chr2	33052161	33052608	Intron
chr2	33060177	33062703	Intron
chr2	33071582	33072877	Intron
chr2	33074787	33077728	Intron
chr2	33148236	33149121	Intron
chr2	33158757	33159131	Intron
chr2	33159328	33159547	Intron
chr2	33169688	33171209	Exon
chr2	33173378	33173589	Intron
chr2	33174451	33176980	Intron
chr2	33195367	33196488	Intron
chr2	33197596	33198701	Intron
chr2	33203480	33204084	Intron

chr2	33204154	33204716	Intron
chr2	33212136	33213533	Intron
chr2	33353648	33354040	Intron
chr2	33354158	33354450	Intron
chr2	34609825	34610177	Distal Intergenic
chr2	34610305	34612136	Distal Intergenic
chr2	34625508	34625754	Distal Intergenic
chr2	34625963	34626224	Distal Intergenic
chr2	34626314	34628018	Distal Intergenic
chr2	34655041	34655240	Distal Intergenic
chr2	34655302	34655779	Distal Intergenic
chr2	34655847	34656100	Distal Intergenic
chr2	34657487	34658600	Distal Intergenic
chr2	34681922	34682189	Distal Intergenic
chr2	34688401	34688863	Distal Intergenic
chr2	34987196	34988303	Distal Intergenic
chr2	35049567	35049969	Distal Intergenic
chr2	35055286	35055869	Distal Intergenic
chr2	35056793	35056992	Distal Intergenic
chr2	35059298	35059925	Distal Intergenic
chr2	35085725	35086001	Distal Intergenic
chr2	35121066	35122802	Distal Intergenic
chr2	35122891	35126550	Distal Intergenic
chr2	35127266	35127778	Distal Intergenic
chr2	35165335	35166098	Distal Intergenic
chr2	35170949	35171292	Distal Intergenic
chr2	35189451	35189650	Distal Intergenic
chr2	35197877	35198745	Distal Intergenic
chr2	35201418	35202829	Distal Intergenic
chr2	35253766	35254070	Distal Intergenic
chr2	35279348	35280446	Distal Intergenic
chr2	35289348	35289633	Distal Intergenic
chr2	35300234	35303588	Distal Intergenic
chr2	35316252	35318562	Distal Intergenic
chr2	35333736	35334150	Distal Intergenic
chr2	35342673	35342925	Distal Intergenic
chr2	35345403	35346411	Distal Intergenic
chr2	35357115	35358032	Distal Intergenic
chr2	35363287	35363530	Distal Intergenic
chr2	35517141	35517904	Distal Intergenic
chr2	35518073	35518272	Distal Intergenic
chr2	35546890	35547823	Distal Intergenic
chr2	35551942	35552420	Distal Intergenic
chr2	35558394	35559302	Distal Intergenic

chr2	35570018	35570374	Distal Intergenic
chr2	35894393	35895970	Distal Intergenic
chr2	35991702	35992135	Distal Intergenic
chr2	35992185	35992432	Distal Intergenic
chr2	36073044	36073392	Distal Intergenic
chr2	36077357	36078974	Distal Intergenic
chr2	36081475	36083352	Distal Intergenic
chr2	36089624	36092159	Distal Intergenic
chr2	36094114	36094321	Distal Intergenic
chr2	36094412	36094938	Distal Intergenic
chr2	36095671	36096211	Distal Intergenic
chr2	36130580	36132145	Distal Intergenic
chr2	37214593	37215089	Intron
chr2	37277484	37278511	Intron
chr2	37278619	37278897	Intron
chr2	37285983	37287330	Exon
chr2	37298006	37298625	Intron
chr2	37298800	37299617	Exon
chr2	37307249	37307599	Intron
chr2	37307803	37308095	Intron
chr2	37514129	37514474	Intron
chr2	37564632	37565042	Distal Intergenic
chr2	37565088	37567147	Distal Intergenic
chr2	37567328	37567565	Distal Intergenic
chr2	37858544	37858772	Distal Intergenic
chr2	38367988	38368225	Intron
chr2	38476747	38477701	Distal Intergenic
chr2	38498657	38499439	Distal Intergenic
chr2	38595262	38595576	Intron
chr2	38598295	38598494	Intron
chr2	38640690	38640927	Distal Intergenic
chr2	38721637	38723262	Distal Intergenic
chr2	38730318	38730517	Distal Intergenic
chr2	38781381	38781592	Distal Intergenic
chr2	38781669	38781913	Distal Intergenic
chr2	38782031	38782285	Distal Intergenic
chr2	38805855	38806445	Intron
chr2	38829124	38830361	Promoter
chr2	38842020	38842601	Distal Intergenic
chr2	38842732	38844330	Distal Intergenic
chr2	38859564	38861809	Distal Intergenic
chr2	38862829	38863545	Distal Intergenic
chr2	38863839	38864290	Distal Intergenic
chr2	38920694	38920910	Intron

chr2	38921173	38921531	Intron
chr2	39081116	39082023	Promoter
chr2	42828458	42828799	Intron
chr2	44674258	44674727	Intron
chr2	44776086	44776412	Promoter
chr2	44782043	44782407	Intron
chr2	44800965	44801473	Intron
chr2	44808110	44808967	Intron
chr2	44811683	44813243	Intron
chr2	45309603	45311189	Distal Intergenic
chr2	45311333	45311664	Distal Intergenic
chr2	45632434	45633164	Intron
chr2	46086141	46087070	Intron
chr2	46110324	46111413	Intron
chr2	46180719	46181629	Intron
chr2	46298599	46299603	Intron
chr2	46346607	46347061	Intron
chr2	46368540	46368739	Intron
chr2	47733502	47734017	Intron
chr2	47932715	47933753	Distal Intergenic
chr2	48295051	48295271	Distal Intergenic
chr2	48296617	48296824	Distal Intergenic
chr2	48804372	48804716	Intron
chr2	48804971	48805304	Intron
chr2	49664846	49665341	Distal Intergenic
chr2	49666613	49668202	Distal Intergenic
chr2	49669075	49669795	Distal Intergenic
chr2	49672894	49673095	Distal Intergenic
chr2	49764322	49767558	Distal Intergenic
chr2	49767611	49768091	Distal Intergenic
chr2	49986154	49986386	Distal Intergenic
chr2	50151243	50151575	Intron
chr2	50151611	50152131	Intron
chr2	50221063	50221361	Intron
chr2	50223451	50224556	Intron
chr2	50232016	50232804	Intron
chr2	50317470	50317736	Intron
chr2	50317782	50318819	Exon
chr2	50318932	50319369	Intron
chr2	50409066	50410392	Intron
chr2	50519632	50520085	Intron
chr2	50523636	50523989	Intron
chr2	50542267	50545509	Intron
chr2	50545559	50547133	Intron

chr2	50647577	50649911	Intron
chr2	50680331	50681911	Intron
chr2	50741294	50741758	Intron
chr2	50742023	50742287	Intron
chr2	50818837	50820292	Intron
chr2	50870313	50871464	Intron
chr2	50917354	50917582	Intron
chr2	50978808	50979134	Intron
chr2	50979187	50979420	Intron
chr2	50993049	50993459	Intron
chr2	50994252	50994511	Intron
chr2	50994672	50995438	Intron
chr2	50995490	51000798	Intron
chr2	51001066	51004659	Intron
chr2	51026435	51026697	Intron
chr2	51026836	51027567	Intron
chr2	51029444	51031133	Intron
chr2	51032507	51032989	Intron
chr2	51033099	51033584	Intron
chr2	51034524	51035518	Intron
chr2	51082721	51084067	Intron
chr2	51134126	51135466	Intron
chr2	51160255	51160845	Intron
chr2	51160922	51161136	Intron
chr2	51274235	51274605	Distal Intergenic
chr2	51294564	51294786	Distal Intergenic
chr2	51294974	51295282	Distal Intergenic
chr2	51790174	51790409	Distal Intergenic
chr2	52600741	52601431	Distal Intergenic
chr2	52728139	52728558	Distal Intergenic
chr2	52732627	52733155	Distal Intergenic
chr2	52742636	52742965	Distal Intergenic
chr2	52743628	52745160	Distal Intergenic
chr2	52745677	52747868	Distal Intergenic
chr2	52749750	52750935	Distal Intergenic
chr2	52797262	52797463	Distal Intergenic
chr2	52797565	52797922	Distal Intergenic
chr2	52801868	52802067	Distal Intergenic
chr2	52802245	52803294	Distal Intergenic
chr2	52810878	52811872	Distal Intergenic
chr2	52813828	52815378	Distal Intergenic
chr2	52887837	52888068	Distal Intergenic
chr2	52888194	52888393	Distal Intergenic
chr2	53049334	53050270	Distal Intergenic

chr2	53050797	53051059	Distal Intergenic
chr2	53051486	53051888	Distal Intergenic
chr2	55001848	55002047	Intron
chr2	55002342	55002719	Intron
chr2	55057095	55059803	Intron
chr2	55059947	55060188	Intron
chr2	55069143	55070206	Intron
chr2	55070252	55070660	Intron
chr2	55105573	55105936	Intron
chr2	55106072	55106522	Intron
chr2	56969291	56969891	Distal Intergenic
chr2	57082229	57082917	Distal Intergenic
chr2	57082961	57083369	Distal Intergenic
chr2	57083626	57084558	Distal Intergenic
chr2	57090068	57091525	Distal Intergenic
chr2	57091684	57092119	Distal Intergenic
chr2	57112994	57113731	Distal Intergenic
chr2	57113932	57114247	Distal Intergenic
chr2	57115680	57116070	Distal Intergenic
chr2	57116398	57116980	Distal Intergenic
chr2	57206120	57206632	Distal Intergenic
chr2	58333050	58333636	Intron
chr2	58333824	58334094	Intron
chr2	58334266	58334631	Intron
chr2	58335555	58335881	Intron
chr2	58335990	58336208	Intron
chr2	58336323	58338816	Intron
chr2	58338856	58339781	Intron
chr2	58349446	58349789	Intron
chr2	58357868	58359084	Exon
chr2	58382341	58382540	Intron
chr2	58394585	58395248	Intron
chr2	58398737	58400136	Intron
chr2	58400338	58400605	Intron
chr2	58400839	58402525	Intron
chr2	58414289	58414579	Intron
chr2	58524061	58524694	Distal Intergenic
chr2	58524864	58525653	Distal Intergenic
chr2	58682424	58683130	Distal Intergenic
chr2	58898031	58898230	Intron
chr2	59009213	59009736	Intron
chr2	59040979	59041645	Intron
chr2	59041721	59041920	Intron
chr2	59177707	59178659	Intron

chr2	59189469	59189732	Intron
chr2	59276394	59276618	Intron
chr2	59285410	59286826	Exon
chr2	59322660	59323219	Distal Intergenic
chr2	59323268	59323660	Distal Intergenic
chr2	59425461	59426222	Distal Intergenic
chr2	59450596	59450829	Distal Intergenic
chr2	59455538	59456034	Distal Intergenic
chr2	59507897	59509032	Distal Intergenic
chr2	59514124	59515578	Distal Intergenic
chr2	59517479	59518274	Distal Intergenic
chr2	59607051	59607568	Distal Intergenic
chr2	59607682	59608050	Distal Intergenic
chr2	59613480	59614752	Distal Intergenic
chr2	59616727	59618588	Distal Intergenic
chr2	59638625	59638872	Distal Intergenic
chr2	59658632	59658854	Distal Intergenic
chr2	59661817	59662460	Distal Intergenic
chr2	59734429	59734865	Distal Intergenic
chr2	59836070	59836333	Distal Intergenic
chr2	59932087	59932483	Distal Intergenic
chr2	59962129	59963028	Distal Intergenic
chr2	59963203	59963990	Distal Intergenic
chr2	60009673	60010018	Distal Intergenic
chr2	60010056	60010382	Distal Intergenic
chr2	60048264	60049392	Distal Intergenic
chr2	60076775	60077954	Distal Intergenic
chr2	60376855	60378066	Distal Intergenic
chr2	60381955	60383598	Distal Intergenic
chr2	60387843	60388455	Distal Intergenic
chr2	60471019	60472436	Distal Intergenic
chr2	60472878	60473655	Distal Intergenic
chr2	60475365	60479725	Distal Intergenic
chr2	60509917	60510116	Distal Intergenic
chr2	60510159	60512239	Distal Intergenic
chr2	60512325	60512587	Distal Intergenic
chr2	60527000	60528338	Distal Intergenic
chr2	60529609	60534642	Distal Intergenic
chr2	60535870	60536359	Distal Intergenic
chr2	60537942	60539703	Distal Intergenic
chr2	60549035	60550267	Distal Intergenic
chr2	60645349	60646913	Distal Intergenic
chr2	60648251	60649051	Distal Intergenic
chr2	60649471	60649681	Distal Intergenic

chr2	60663848	60666016	Distal Intergenic
chr2	60666754	60668183	Distal Intergenic
chr2	60668831	60669200	Distal Intergenic
chr2	60692541	60694915	Intron
chr2	60694951	60695451	Intron
chr2	60695510	60695828	Intron
chr2	60702383	60704429	Intron
chr2	60707990	60708302	Intron
chr2	60708399	60709884	Intron
chr2	60710272	60710575	Intron
chr2	60715331	60719105	Intron
chr2	60719790	60720327	Intron
chr2	60739281	60739510	Intron
chr2	60739617	60740816	Intron
chr2	60800543	60800945	Distal Intergenic
chr2	60902443	60902794	Distal Intergenic
chr2	61105648	61106167	Intron
chr2	61106511	61108028	Promoter
chr2	61146907	61148512	Exon
chr2	61431207	61432654	Promoter
chr2	61549811	61551047	Intron
chr2	61579380	61579579	Intron
chr2	64877832	64878508	Intron
chr2	64979227	64979798	Distal Intergenic
chr2	64985721	64985949	Distal Intergenic
chr2	65041835	65042074	Distal Intergenic
chr2	65045865	65046281	Distal Intergenic
chr2	65059613	65061805	Distal Intergenic
chr2	65065122	65066202	Distal Intergenic
chr2	65074685	65075878	Exon
chr2	65076213	65076436	Intron
chr2	65077206	65077513	Intron
chr2	65103158	65103387	Distal Intergenic
chr2	65104972	65107569	Distal Intergenic
chr2	65248033	65248410	3' UTR
chr2	65961974	65962181	Intron
chr2	66018583	66018871	Intron
chr2	68060219	68060418	Distal Intergenic
chr2	68060482	68061075	Distal Intergenic
chr2	68098289	68099031	Distal Intergenic
chr2	68141961	68143989	Distal Intergenic
chr2	68700127	68700935	Intron
chr2	68716823	68717777	Exon
chr2	68748669	68749565	Intron

chr2	68753693	68754188	Intron
chr2	68754637	68755716	Intron
chr2	68762667	68765472	5' UTR
chr2	68772880	68774699	Intron
chr2	68818051	68818598	Intron
chr2	68819095	68819294	Intron
chr2	68820499	68823122	Intron
chr2	68823486	68824019	Intron
chr2	68824168	68827296	Intron
chr2	68832304	68834012	Intron
chr2	68894683	68895558	Distal Intergenic
chr2	68911254	68913173	Intron
chr2	68914113	68916196	Intron
chr2	69160792	69161377	Distal Intergenic
chr2	69238841	69239854	Promoter
chr2	69484832	69485132	Distal Intergenic
chr2	69560888	69561264	Intron
chr2	69561372	69561831	Exon
chr2	69627925	69628140	Intron
chr2	69628196	69628483	Intron
chr2	69698973	69699208	3' UTR
chr2	69699565	69700676	3' UTR
chr2	70499762	70500243	Intron
chr2	70649724	70649935	Distal Intergenic
chr2	70893798	70893999	Intron
chr2	71049778	71050448	Distal Intergenic
chr2	71069583	71070366	Distal Intergenic
chr2	71380300	71380761	Distal Intergenic
chr2	71427319	71427941	Intron
chr2	71431839	71433614	Intron
chr2	71435972	71437066	Intron
chr2	71460583	71460792	Distal Intergenic
chr2	71460866	71461122	Distal Intergenic
chr2	71461208	71461621	Distal Intergenic
chr2	71464403	71464961	Distal Intergenic
chr2	71477865	71479483	Distal Intergenic
chr2	71481798	71482157	Distal Intergenic
chr2	71482386	71483015	Distal Intergenic
chr2	71489239	71491831	Distal Intergenic
chr2	71527975	71529250	Intron
chr2	71576261	71578420	Promoter
chr2	71609815	71610040	Intron
chr2	71625723	71628698	5' UTR
chr2	71628770	71629142	5' UTR

chr2	71638236	71638506	Intron
chr2	71638596	71641109	Intron
chr2	71641161	71641402	Intron
chr2	71642041	71644736	Promoter
chr2	71652149	71652387	Intron
chr2	72079560	72079954	Distal Intergenic
chr2	72079994	72080225	Distal Intergenic
chr2	72091111	72091350	Distal Intergenic
chr2	72118704	72119360	Distal Intergenic
chr2	72123206	72127911	Distal Intergenic
chr2	72127958	72128297	Distal Intergenic
chr2	72134972	72136643	Distal Intergenic
chr2	72146200	72146465	Distal Intergenic
chr2	72146629	72148657	Distal Intergenic
chr2	72168479	72169464	Distal Intergenic
chr2	72188492	72188733	Distal Intergenic
chr2	72188988	72190649	Distal Intergenic
chr2	72282162	72282495	Distal Intergenic
chr2	72282554	72282783	Distal Intergenic
chr2	72282941	72285491	Distal Intergenic
chr2	72313808	72314154	Distal Intergenic
chr2	72314352	72314611	Distal Intergenic
chr2	72314827	72315511	Distal Intergenic
chr2	72570859	72574068	Intron
chr2	72574360	72575418	Intron
chr2	72719823	72720469	Intron
chr2	72746651	72747791	Intron
chr2	72818368	72820710	Intron
chr2	72833220	72835729	Intron
chr2	72843674	72844145	Intron
chr2	72861861	72862071	Intron
chr2	72873597	72874502	Intron
chr2	72879064	72880269	Intron
chr2	73051856	73052055	Intron
chr2	73150335	73150686	Intron
chr2	73423278	73423818	Distal Intergenic
chr2	73749055	73749289	Intron
chr2	73749472	73749916	Intron
chr2	74688775	74689456	Exon
chr2	75447041	75449114	Distal Intergenic
chr2	75450672	75451212	Distal Intergenic
chr2	75451275	75451985	Distal Intergenic
chr2	75452149	75453069	Distal Intergenic
chr2	75469428	75469790	Distal Intergenic

chr2	75478546	75478828	Distal Intergenic
chr2	75479002	75482344	Distal Intergenic
chr2	75495863	75497218	Distal Intergenic
chr2	75497344	75497598	Distal Intergenic
chr2	75497698	75499630	Distal Intergenic
chr2	75499806	75500453	Distal Intergenic
chr2	75501716	75501930	Distal Intergenic
chr2	75507171	75507960	Distal Intergenic
chr2	75520762	75524360	Distal Intergenic
chr2	75527137	75527422	Distal Intergenic
chr2	75533230	75533432	Distal Intergenic
chr2	75533622	75534588	Distal Intergenic
chr2	75534751	75534972	Distal Intergenic
chr2	75539006	75542963	Distal Intergenic
chr2	75603242	75603834	Distal Intergenic
chr2	75605349	75607097	Distal Intergenic
chr2	75607184	75607668	Distal Intergenic
chr2	75610265	75611187	Distal Intergenic
chr2	75669761	75669975	Distal Intergenic
chr2	75670337	75671139	Distal Intergenic
chr2	75707424	75707768	Distal Intergenic
chr2	75895851	75896637	Intron
chr2	75898137	75898537	Intron
chr2	76895174	76895373	Distal Intergenic
chr2	77106314	77107356	Intron
chr2	77783543	77784569	Distal Intergenic
chr2	78962818	78963228	Distal Intergenic
chr2	78998933	79000194	Distal Intergenic
chr2	79004433	79006049	Distal Intergenic
chr2	79007465	79009041	Distal Intergenic
chr2	79238704	79239055	Distal Intergenic
chr2	79372664	79373310	Distal Intergenic
chr2	79396002	79396201	Distal Intergenic
chr2	79406392	79406611	Distal Intergenic
chr2	79436955	79438069	Intron
chr2	79443055	79444729	Intron
chr2	79445081	79445328	Intron
chr2	79450723	79451278	Intron
chr2	79458309	79460245	Intron
chr2	79462152	79462448	Intron
chr2	79462495	79463191	Intron
chr2	79475907	79476644	Intron
chr2	79496888	79497282	Intron
chr2	79538228	79538493	Intron

chr2	79538622	79538904	Intron
chr2	80019730	80020332	Intron
chr2	80419817	80421107	Intron
chr2	80478656	80478918	Intron
chr2	80478988	80479345	Intron
chr2	80528067	80528766	Intron
chr2	81733113	81733331	Distal Intergenic
chr2	82018444	82018784	Distal Intergenic
chr2	82018949	82019378	Distal Intergenic
chr2	83485029	83485371	Distal Intergenic
chr2	83511975	83512309	Distal Intergenic
chr2	83515034	83515280	Distal Intergenic
chr2	83563902	83564524	Distal Intergenic
chr2	83564805	83566236	Distal Intergenic
chr2	83568016	83569505	Distal Intergenic
chr2	83584436	83585076	Distal Intergenic
chr2	84333617	84334260	Distal Intergenic
chr2	84351870	84352108	Distal Intergenic
chr2	84369099	84369358	Distal Intergenic
chr2	84379222	84380214	Distal Intergenic
chr2	84380318	84380533	Distal Intergenic
chr2	84380663	84381583	Distal Intergenic
chr2	84384107	84386359	Distal Intergenic
chr2	84388454	84392502	Distal Intergenic
chr2	84458197	84460201	Distal Intergenic
chr2	84465898	84469354	Distal Intergenic
chr2	84470434	84470723	Distal Intergenic
chr2	84472349	84477298	Distal Intergenic
chr2	84478361	84478587	Distal Intergenic
chr2	84479917	84480495	Distal Intergenic
chr2	84487948	84488203	Distal Intergenic
chr2	84488325	84491650	Distal Intergenic
chr2	84510524	84510751	Distal Intergenic
chr2	84510916	84511472	Distal Intergenic
chr2	84544034	84546506	Distal Intergenic
chr2	84546741	84547114	Distal Intergenic
chr2	84553957	84555389	Distal Intergenic
chr2	84555875	84556713	Distal Intergenic
chr2	84583592	84583945	Distal Intergenic
chr2	84584422	84584646	Distal Intergenic
chr2	84667174	84667376	Intron
chr2	84877111	84877437	Intron
chr2	84877677	84877967	Intron
chr2	84887677	84888128	Intron

chr2	84888195	84888468	Intron
chr2	84888697	84888956	Intron
chr2	84889938	84891547	Intron
chr2	84900158	84901904	Intron
chr2	84906437	84909163	Exon
chr2	85349984	85350354	Distal Intergenic
chr2	85350398	85351381	Distal Intergenic
chr2	85351525	85352089	Distal Intergenic
chr2	85394590	85395642	Intron
chr2	90336188	90336994	Intron
chr2	90340754	90341442	Intron
chr2	90382631	90382974	Intron
chr2	90403275	90403487	Intron
chr2	90405624	90406516	Intron
chr2	90451245	90453059	Intron
chr2	90453137	90453498	Intron
chr2	90585153	90585467	Distal Intergenic
chr2	90586053	90586530	Distal Intergenic
chr2	90744305	90744826	Distal Intergenic
chr2	90744932	90745362	Distal Intergenic
chr2	90757062	90757814	Distal Intergenic
chr2	90757852	90758707	Distal Intergenic
chr2	90894193	90894580	Distal Intergenic
chr2	90901722	90902261	Distal Intergenic
chr2	90909258	90910420	Distal Intergenic
chr2	90910769	90911281	Distal Intergenic
chr2	91077324	91077742	Distal Intergenic
chr2	91077787	91078040	Distal Intergenic
chr2	91082714	91084044	Distal Intergenic
chr2	91097180	91097386	Distal Intergenic
chr2	91097422	91098251	Distal Intergenic
chr2	91104970	91105297	Distal Intergenic
chr2	91105372	91105950	Distal Intergenic
chr2	91139668	91139982	Distal Intergenic
chr2	91355599	91355933	Distal Intergenic
chr2	91366948	91367166	Distal Intergenic
chr2	91377402	91377708	Distal Intergenic
chr2	91489333	91490447	Distal Intergenic
chr2	91536170	91536521	Distal Intergenic
chr2	91536599	91536844	Distal Intergenic
chr2	91550580	91550979	Distal Intergenic
chr2	91551040	91551816	Distal Intergenic
chr2	91570657	91571057	Distal Intergenic
chr2	91787660	91789134	Distal Intergenic

chr2	91804304	91806422	Exon
chr2	91852313	91853186	Distal Intergenic
chr2	92018853	92019956	Distal Intergenic
chr2	92020205	92020749	Distal Intergenic
chr2	92024257	92024713	Distal Intergenic
chr2	92044496	92044911	Distal Intergenic
chr2	92097704	92098773	Distal Intergenic
chr2	92211748	92212017	Distal Intergenic
chr2	92212238	92214257	Distal Intergenic
chr2	92214890	92215860	Distal Intergenic
chr2	92267607	92268606	Distal Intergenic
chr2	92518679	92518887	Distal Intergenic
chr2	92776891	92778759	Distal Intergenic
chr2	92779097	92779751	Distal Intergenic
chr2	92785340	92785807	Distal Intergenic
chr2	92788773	92789100	Distal Intergenic
chr2	92835512	92835798	Distal Intergenic
chr2	92973230	92974533	Distal Intergenic
chr2	93023261	93028090	Distal Intergenic
chr2	93616649	93617230	Distal Intergenic
chr2	93742954	93743190	Distal Intergenic
chr2	93747580	93747854	Distal Intergenic
chr2	93997137	93998064	Distal Intergenic
chr2	94246565	94246768	Distal Intergenic
chr2	94246886	94247611	Distal Intergenic
chr2	95230549	95231023	Distal Intergenic
chr2	97278163	97278488	Exon
chr2	97474239	97475490	3' UTR
chr2	97550471	97551385	Intron
chr2	99973321	99973573	Intron
chr2	101518599	101518802	Intron
chr2	101632283	101633436	Intron
chr2	101826900	101827099	Distal Intergenic
chr2	101945292	101946038	Distal Intergenic
chr2	102143096	102143582	Distal Intergenic
chr2	102143644	102144358	Distal Intergenic
chr2	102273970	102274245	Distal Intergenic
chr2	102287949	102289249	Distal Intergenic
chr2	102289332	102290035	Distal Intergenic
chr2	102290228	102290584	Distal Intergenic
chr2	102347541	102348795	Intron
chr2	102713009	102713246	Intron
chr2	102713402	102714963	Intron
chr2	102721436	102723597	Intron

chr2	102723812	102726135	Intron
chr2	102728199	102728398	Intron
chr2	102729198	102729421	Intron
chr2	102729683	102730086	Intron
chr2	102730128	102730663	Intron
chr2	102733646	102738352	Intron
chr2	102738484	102740198	Intron
chr2	102740284	102741932	Intron
chr2	102784236	102785131	Exon
chr2	102785238	102785709	Intron
chr2	102787885	102789677	Exon
chr2	102796461	102797701	Distal Intergenic
chr2	102797756	102799845	Distal Intergenic
chr2	102820806	102822383	Intron
chr2	102913891	102914104	Distal Intergenic
chr2	102957324	102957666	Intron
chr2	103078708	103079298	Distal Intergenic
chr2	103111454	103111920	Intron
chr2	103350581	103350953	Intron
chr2	103351013	103351255	Intron
chr2	103471069	103471370	Distal Intergenic
chr2	103472525	103473947	Distal Intergenic
chr2	103495852	103496280	Distal Intergenic
chr2	103497807	103498038	Distal Intergenic
chr2	103510238	103510464	Distal Intergenic
chr2	103527041	103528060	Distal Intergenic
chr2	103530091	103531818	Distal Intergenic
chr2	103536323	103536777	Distal Intergenic
chr2	103601497	103601787	Distal Intergenic
chr2	103635796	103637106	Distal Intergenic
chr2	103936147	103936454	Distal Intergenic
chr2	103936536	103936946	Distal Intergenic
chr2	103938776	103939654	Distal Intergenic
chr2	103942632	103943381	Distal Intergenic
chr2	104306566	104307684	Distal Intergenic
chr2	104333322	104334318	Distal Intergenic
chr2	104430576	104431259	Distal Intergenic
chr2	104431310	104431618	Distal Intergenic
chr2	104470099	104470298	Distal Intergenic
chr2	104470347	104470617	Distal Intergenic
chr2	104539776	104540332	Distal Intergenic
chr2	104544557	104547112	Distal Intergenic
chr2	104572063	104573459	Distal Intergenic
chr2	104581778	104582101	Distal Intergenic

chr2	104585363	104585688	Distal Intergenic
chr2	104603142	104603405	Distal Intergenic
chr2	104603463	104603722	Distal Intergenic
chr2	104655669	104656359	Distal Intergenic
chr2	104657179	104657392	Distal Intergenic
chr2	104657444	104657682	Distal Intergenic
chr2	104688550	104689765	Distal Intergenic
chr2	104695833	104696371	Distal Intergenic
chr2	104856654	104857102	Distal Intergenic
chr2	104857233	104857750	Distal Intergenic
chr2	105212744	105214706	Distal Intergenic
chr2	105214815	105215698	Distal Intergenic
chr2	105215830	105216570	Distal Intergenic
chr2	105238672	105239318	Distal Intergenic
chr2	105239859	105240210	Distal Intergenic
chr2	105256643	105258312	Distal Intergenic
chr2	105636577	105636901	Distal Intergenic
chr2	105637376	105637575	Distal Intergenic
chr2	105637793	105637992	Distal Intergenic
chr2	105744779	105745012	Distal Intergenic
chr2	105756333	105757064	Distal Intergenic
chr2	106814319	106814522	Distal Intergenic
chr2	106830866	106832525	Distal Intergenic
chr2	106845015	106845240	Distal Intergenic
chr2	106845353	106847337	Distal Intergenic
chr2	106916521	106916753	Distal Intergenic
chr2	106916952	106917419	Distal Intergenic
chr2	107066909	107068886	Exon
chr2	107068988	107069380	Exon
chr2	107072376	107073810	Exon
chr2	107181392	107182082	Distal Intergenic
chr2	107232911	107233172	Distal Intergenic
chr2	108238940	108240831	Distal Intergenic
chr2	108244572	108244931	Distal Intergenic
chr2	108462621	108464165	Intron
chr2	108501471	108501736	Intron
chr2	108501901	108502253	Intron
chr2	109121019	109121397	Intron
chr2	109121467	109121687	Intron
chr2	109683201	109683436	Distal Intergenic
chr2	109700675	109701621	Distal Intergenic
chr2	109730718	109730968	Distal Intergenic
chr2	109760759	109762419	Intron
chr2	109763069	109763705	Intron

chr2	109799244	109799514	Intron
chr2	109809614	109809844	Intron
chr2	109810018	109810351	Intron
chr2	109824845	109825891	Intron
chr2	109892570	109894022	Intron
chr2	109995954	109996175	Intron
chr2	109996253	109997202	Intron
chr2	110012488	110014121	Intron
chr2	110016183	110017876	Intron
chr2	110025734	110027718	Intron
chr2	110032032	110033400	Intron
chr2	110360212	110361793	Intron
chr2	110549928	110550859	Promoter
chr2	110558571	110559502	Exon
chr2	112043044	112044027	Intron
chr2	112077958	112078703	Promoter
chr2	112124932	112125144	Exon
chr2	112147773	112148253	Intron
chr2	112148306	112149817	Intron
chr2	112213192	112214176	Intron
chr2	112217939	112218431	Intron
chr2	112219717	112220562	Intron
chr2	112238401	112239744	Intron
chr2	112294808	112295115	Distal Intergenic
chr2	112295600	112295829	Distal Intergenic
chr2	113214646	113214996	Distal Intergenic
chr2	113216242	113216778	Distal Intergenic
chr2	113216816	113217139	Distal Intergenic
chr2	113236538	113237037	Distal Intergenic
chr2	113259753	113261922	Exon
chr2	113280364	113281434	Intron
chr2	113657998	113658898	Distal Intergenic
chr2	113661185	113661797	Distal Intergenic
chr2	113687948	113689336	Distal Intergenic
chr2	114071796	114072279	Distal Intergenic
chr2	114480274	114480853	Exon
chr2	114622871	114623975	Distal Intergenic
chr2	115406823	115407841	Intron
chr2	115434306	115436493	Intron
chr2	115605815	115606999	Intron
chr2	115796171	115796417	Intron
chr2	115891868	115892178	Intron
chr2	116552952	116553687	Intron
chr2	116791808	116792111	Distal Intergenic

chr2	116797812	116798343	Distal Intergenic
chr2	117075490	117075933	Distal Intergenic
chr2	117186195	117187267	Distal Intergenic
chr2	117468769	117469189	Distal Intergenic
chr2	117469364	117470316	Distal Intergenic
chr2	117493578	117494056	Distal Intergenic
chr2	117556347	117557607	Distal Intergenic
chr2	117612856	117613243	Distal Intergenic
chr2	117613410	117615198	Distal Intergenic
chr2	117628161	117628630	Distal Intergenic
chr2	117628853	117629662	Distal Intergenic
chr2	117629773	117630185	Distal Intergenic
chr2	117630608	117630912	Distal Intergenic
chr2	117708180	117708444	Distal Intergenic
chr2	117728796	117729176	Distal Intergenic
chr2	117814240	117814552	Distal Intergenic
chr2	117825336	117825573	Distal Intergenic
chr2	117919865	117921333	Distal Intergenic
chr2	117935194	117936853	Distal Intergenic
chr2	118445582	118445929	Distal Intergenic
chr2	118446088	118446426	Distal Intergenic
chr2	118484258	118486280	Distal Intergenic
chr2	118489222	118491178	Distal Intergenic
chr2	118494447	118495532	Distal Intergenic
chr2	118499428	118499882	Distal Intergenic
chr2	118502294	118502667	Distal Intergenic
chr2	118502957	118503750	Distal Intergenic
chr2	118586673	118589442	3' UTR
chr2	118589602	118589830	3' UTR
chr2	118590000	118590326	Distal Intergenic
chr2	118590441	118592232	Distal Intergenic
chr2	118631142	118631342	Distal Intergenic
chr2	118631399	118632564	Distal Intergenic
chr2	118640083	118640335	Distal Intergenic
chr2	118787607	118787855	Distal Intergenic
chr2	118938870	118939296	Distal Intergenic
chr2	118998886	118999329	Distal Intergenic
chr2	118999542	119000818	Distal Intergenic
chr2	119034081	119034301	Distal Intergenic
chr2	119089070	119089277	Distal Intergenic
chr2	119170032	119170934	Distal Intergenic
chr2	119240889	119241536	Distal Intergenic
chr2	119241842	119242041	Distal Intergenic
chr2	119302481	119303827	Distal Intergenic

chr2	119372927	119373139	Distal Intergenic
chr2	119373796	119373998	Distal Intergenic
chr2	119420401	119420680	Distal Intergenic
chr2	119500868	119501067	Distal Intergenic
chr2	119612893	119613226	Distal Intergenic
chr2	119798736	119798935	Distal Intergenic
chr2	119799234	119799545	Distal Intergenic
chr2	119820926	119821290	Distal Intergenic
chr2	119832945	119834339	Distal Intergenic
chr2	119930476	119931740	Distal Intergenic
chr2	119954111	119955064	Distal Intergenic
chr2	119957225	119957710	Distal Intergenic
chr2	119957843	119958210	Distal Intergenic
chr2	119958441	119958646	Distal Intergenic
chr2	119959366	119961348	Distal Intergenic
chr2	119962184	119962622	Distal Intergenic
chr2	119965625	119967881	Distal Intergenic
chr2	119972617	119973143	Distal Intergenic
chr2	120212943	120213429	Intron
chr2	120229455	120229871	Intron
chr2	120339480	120339796	Intron
chr2	120434740	120435322	Distal Intergenic
chr2	120557287	120557548	Intron
chr2	121048709	121049438	Intron
chr2	121212314	121212713	Distal Intergenic
chr2	121238764	121239524	Distal Intergenic
chr2	121239983	121240227	Distal Intergenic
chr2	121282425	121283355	Distal Intergenic
chr2	121285724	121286364	Distal Intergenic
chr2	121446823	121447022	Distal Intergenic
chr2	121454324	121454570	Distal Intergenic
chr2	121782258	121782951	Distal Intergenic
chr2	121990327	121990622	Intron
chr2	122055395	122058139	Distal Intergenic
chr2	122059844	122060071	Distal Intergenic
chr2	122060284	122060542	Distal Intergenic
chr2	122130873	122131091	Intron
chr2	122131180	122132242	Intron
chr2	122132283	122132685	Intron
chr2	122151795	122152022	Intron
chr2	122152068	122153349	Intron
chr2	122153477	122153971	Intron
chr2	122170143	122172825	Intron
chr2	122174891	122175117	Intron

chr2	122477818	122478625	Intron
chr2	122523440	122523715	3' UTR
chr2	124684040	124684471	Distal Intergenic
chr2	124684523	124684756	Distal Intergenic
chr2	125564235	125564442	Intron
chr2	125564661	125565702	Intron
chr2	125617301	125617571	Intron
chr2	125618095	125618366	Intron
chr2	125618486	125618722	Intron
chr2	125683966	125685220	Distal Intergenic
chr2	126017820	126018266	Distal Intergenic
chr2	126128448	126129476	Distal Intergenic
chr2	126135085	126137483	Distal Intergenic
chr2	126189077	126189345	Distal Intergenic
chr2	126189894	126190331	Distal Intergenic
chr2	126191372	126191661	Distal Intergenic
chr2	126499663	126502520	Distal Intergenic
chr2	126502787	126503017	Distal Intergenic
chr2	126700536	126701441	Distal Intergenic
chr2	126701534	126701870	Distal Intergenic
chr2	126758092	126758458	Distal Intergenic
chr2	126758621	126759597	Distal Intergenic
chr2	126763654	126765201	Distal Intergenic
chr2	126771504	126771789	Distal Intergenic
chr2	126774002	126774235	Distal Intergenic
chr2	126774280	126774747	Distal Intergenic
chr2	126960037	126960301	Distal Intergenic
chr2	126962865	126963244	Distal Intergenic
chr2	126963387	126963630	Distal Intergenic
chr2	126963793	126964062	Distal Intergenic
chr2	126964229	126964892	Distal Intergenic
chr2	126965086	126965303	Distal Intergenic
chr2	127095620	127095876	Distal Intergenic
chr2	127412938	127413196	Promoter
chr2	127475109	127475786	Distal Intergenic
chr2	127475897	127476276	Distal Intergenic
chr2	127543901	127544322	Distal Intergenic
chr2	127544428	127544979	Distal Intergenic
chr2	127547704	127548607	Distal Intergenic
chr2	127959775	127960389	Intron
chr2	128253720	128255610	Exon
chr2	128644263	128644529	Promoter
chr2	128793064	128793537	Distal Intergenic
chr2	128955503	128955909	Distal Intergenic

chr2	128991877	128993921	Distal Intergenic
chr2	129001322	129002042	Distal Intergenic
chr2	129002186	129002436	Distal Intergenic
chr2	129002480	129003139	Distal Intergenic
chr2	129032414	129032766	Intron
chr2	129068546	129068971	Intron
chr2	129071618	129071904	Intron
chr2	129072040	129072610	Intron
chr2	129083895	129084114	Distal Intergenic
chr2	129088785	129090379	Distal Intergenic
chr2	129122635	129122910	Distal Intergenic
chr2	129123050	129123810	Distal Intergenic
chr2	129133671	129133939	Distal Intergenic
chr2	129134513	129134958	Distal Intergenic
chr2	129135529	129135817	Distal Intergenic
chr2	129149748	129150744	Distal Intergenic
chr2	129156214	129157061	Distal Intergenic
chr2	129160241	129160734	Distal Intergenic
chr2	129163764	129164449	Distal Intergenic
chr2	129232311	129234831	Distal Intergenic
chr2	129237322	129238256	Distal Intergenic
chr2	129246527	129247142	Distal Intergenic
chr2	129418180	129418693	Distal Intergenic
chr2	129419081	129419342	Distal Intergenic
chr2	129419395	129420064	Distal Intergenic
chr2	129425201	129425630	Distal Intergenic
chr2	129457915	129460705	Distal Intergenic
chr2	129626392	129627874	Distal Intergenic
chr2	129643033	129643245	Distal Intergenic
chr2	129645363	129646354	Distal Intergenic
chr2	129751430	129751701	Distal Intergenic
chr2	129754229	129755734	Distal Intergenic
chr2	129779934	129781722	Distal Intergenic
chr2	129788910	129789190	Distal Intergenic
chr2	129789294	129789796	Distal Intergenic
chr2	129825035	129825351	Distal Intergenic
chr2	129825409	129825993	Distal Intergenic
chr2	129859893	129860541	Distal Intergenic
chr2	130004413	130004703	Intron
chr2	130099736	130100054	Distal Intergenic
chr2	130250000	130250215	Distal Intergenic
chr2	130455628	130455997	Distal Intergenic
chr2	131025210	131026395	Distal Intergenic
chr2	131028834	131031362	Exon

chr2	131033882	131034711	Distal Intergenic
chr2	131034901	131035198	Distal Intergenic
chr2	131035399	131036349	Exon
chr2	131088475	131089284	Distal Intergenic
chr2	131218475	131218749	Downstream
chr2	131229322	131229691	Intron
chr2	131229846	131230504	Intron
chr2	131335591	131336444	Exon
chr2	131372862	131373074	Intron
chr2	131586810	131587093	Distal Intergenic
chr2	131587177	131587881	Distal Intergenic
chr2	131727629	131727835	Intron
chr2	131727899	131728372	Intron
chr2	131728577	131729285	Intron
chr2	131757518	131759220	Intron
chr2	131770494	131771378	Intron
chr2	131774738	131776823	Intron
chr2	131776991	131777258	Intron
chr2	131829941	131832974	Intron
chr2	131908057	131911336	Intron
chr2	131923102	131923356	Intron
chr2	131923407	131923857	Intron
chr2	131924059	131924261	Intron
chr2	131924462	131925364	Intron
chr2	131936431	131937208	Intron
chr2	131978427	131979211	Intron
chr2	131994565	131995485	Intron
chr2	132073242	132073511	Intron
chr2	132073919	132074208	Intron
chr2	132165728	132166225	Exon
chr2	132370475	132370710	Distal Intergenic
chr2	132404170	132404511	Distal Intergenic
chr2	132433627	132433954	Distal Intergenic
chr2	132438961	132439768	Distal Intergenic
chr2	132769377	132769654	Distal Intergenic
chr2	132769751	132770314	Distal Intergenic
chr2	132770363	132771256	Distal Intergenic
chr2	132771349	132772579	Distal Intergenic
chr2	132777441	132778103	Distal Intergenic
chr2	133542165	133542369	Exon
chr2	133916878	133917368	Intron
chr2	135350984	135351634	Intron
chr2	135441448	135442297	Intron
chr2	135563207	135563471	Distal Intergenic

chr2	135827346	135828065	Intron
chr2	136717821	136718312	Intron
chr2	136743721	136746844	Promoter
chr2	136890745	136890980	Distal Intergenic
chr2	136893290	136893601	Distal Intergenic
chr2	136933386	136935666	Distal Intergenic
chr2	136935703	136937947	Distal Intergenic
chr2	136938092	136938508	Distal Intergenic
chr2	136938552	136938802	Distal Intergenic
chr2	136939407	136940346	Distal Intergenic
chr2	136940628	136940926	Distal Intergenic
chr2	136941011	136942523	Distal Intergenic
chr2	136942604	136943193	Distal Intergenic
chr2	136974338	136975495	Distal Intergenic
chr2	136975605	136975874	Distal Intergenic
chr2	136976015	136977275	Distal Intergenic
chr2	137050096	137051809	Distal Intergenic
chr2	137088066	137089206	Distal Intergenic
chr2	137199272	137199495	Distal Intergenic
chr2	137234730	137236248	Distal Intergenic
chr2	137238534	137238855	Distal Intergenic
chr2	137238976	137239831	Distal Intergenic
chr2	137255896	137257181	Distal Intergenic
chr2	137280721	137283111	Distal Intergenic
chr2	137284008	137284208	Distal Intergenic
chr2	137284316	137284709	Distal Intergenic
chr2	137319877	137321221	Distal Intergenic
chr2	137391877	137394561	Distal Intergenic
chr2	137398852	137401237	Distal Intergenic
chr2	137419720	137424749	Distal Intergenic
chr2	137430524	137432979	Distal Intergenic
chr2	137479024	137481321	Distal Intergenic
chr2	137536414	137536680	Intron
chr2	137541586	137542175	Intron
chr2	137545840	137546698	Intron
chr2	137546909	137547141	Intron
chr2	137547346	137548391	Intron
chr2	137548537	137549126	Intron
chr2	137550594	137550856	Intron
chr2	137551022	137551275	Intron
chr2	137551457	137551890	Intron
chr2	137633242	137633603	Intron
chr2	137702463	137703479	Intron
chr2	138049312	138049757	Intron

chr2	138145873	138147921	Intron
chr2	138257547	138259159	Intron
chr2	138303109	138303394	Intron
chr2	138303548	138304053	Intron
chr2	138329851	138330050	Exon
chr2	139307965	139308716	Exon
chr2	139311186	139313033	Intron
chr2	140494745	140494971	Distal Intergenic
chr2	140495023	140495289	Distal Intergenic
chr2	140496091	140497086	Distal Intergenic
chr2	140616005	140616960	Distal Intergenic
chr2	140662448	140664362	Distal Intergenic
chr2	140664534	140664785	Distal Intergenic
chr2	140673155	140674145	Distal Intergenic
chr2	140730479	140730685	Distal Intergenic
chr2	140730840	140731070	Distal Intergenic
chr2	140731336	140731537	Distal Intergenic
chr2	141091128	141093466	Exon
chr2	141096713	141098385	Intron
chr2	141099557	141102736	Intron
chr2	141103441	141103664	Intron
chr2	141103969	141104255	Intron
chr2	141104327	141106362	Intron
chr2	141273823	141275473	Exon
chr2	141289471	141290733	Intron
chr2	142464814	142465077	Intron
chr2	142521539	142522222	Intron
chr2	142889132	142889333	Promoter
chr2	143678461	143678818	Intron
chr2	143678965	143679322	Intron
chr2	143729107	143731244	Intron
chr2	143734697	143735439	Intron
chr2	143739900	143740887	Intron
chr2	143741069	143741410	Intron
chr2	143741582	143741870	Intron
chr2	143755293	143755761	Intron
chr2	144094357	144094595	Intron
chr2	144095774	144096262	Intron
chr2	144381675	144381875	Exon
chr2	144565704	144566014	Distal Intergenic
chr2	144566058	144567221	Distal Intergenic
chr2	144772052	144773165	Intron
chr2	145044091	145045644	Intron
chr2	145099095	145101286	Distal Intergenic

chr2	145109625	145109875	Distal Intergenic
chr2	145147246	145147449	Exon
chr2	145163587	145165142	Intron
chr2	145187374	145188952	Exon
chr2	145190387	145190626	Intron
chr2	145195932	145196831	Intron
chr2	145656035	145656352	Intron
chr2	145668984	145669782	Intron
chr2	145671928	145673022	Intron
chr2	145674320	145675491	Intron
chr2	145686125	145686324	Intron
chr2	145686420	145687175	Intron
chr2	145687221	145687460	Intron
chr2	145687968	145688508	Intron
chr2	145691274	145692899	Intron
chr2	145729414	145729622	Intron
chr2	146114279	146114629	Distal Intergenic
chr2	146312843	146313941	Distal Intergenic
chr2	146359033	146359311	Distal Intergenic
chr2	146379252	146379576	Distal Intergenic
chr2	146379769	146380038	Distal Intergenic
chr2	146396876	146397937	Distal Intergenic
chr2	146400132	146401002	Distal Intergenic
chr2	146802503	146803166	Distal Intergenic
chr2	146811708	146811907	Distal Intergenic
chr2	146838278	146840051	Distal Intergenic
chr2	148006269	148006518	Distal Intergenic
chr2	148010032	148011515	Distal Intergenic
chr2	148265356	148266119	Distal Intergenic
chr2	148281400	148281788	Distal Intergenic
chr2	148294324	148295287	Distal Intergenic
chr2	148295383	148296112	Distal Intergenic
chr2	148332688	148333613	Distal Intergenic
chr2	148347077	148347344	Distal Intergenic
chr2	148347680	148348540	Distal Intergenic
chr2	148350525	148351709	Distal Intergenic
chr2	148351826	148352216	Distal Intergenic
chr2	148456711	148457418	Distal Intergenic
chr2	148504826	148505161	Distal Intergenic
chr2	148505453	148505665	Distal Intergenic
chr2	148505978	148506644	Distal Intergenic
chr2	148569644	148569888	Distal Intergenic
chr2	148570123	148570372	Distal Intergenic
chr2	148698658	148698938	Intron

chr2	149363857	149364159	Distal Intergenic
chr2	149399379	149399969	Distal Intergenic
chr2	149689652	149689865	Intron
chr2	149712467	149712735	Intron
chr2	149823984	149824248	Intron
chr2	149824402	149824608	Intron
chr2	150665341	150665714	Distal Intergenic
chr2	150665754	150666162	Distal Intergenic
chr2	150666208	150666671	Distal Intergenic
chr2	150667065	150668024	Distal Intergenic
chr2	150730660	150730861	Distal Intergenic
chr2	151798691	151799408	Distal Intergenic
chr2	151799848	151800051	Distal Intergenic
chr2	151803307	151804497	Distal Intergenic
chr2	151804881	151805097	Distal Intergenic
chr2	151805176	151805558	Distal Intergenic
chr2	151931242	151931495	Distal Intergenic
chr2	152052288	152052512	Distal Intergenic
chr2	152059691	152060053	Distal Intergenic
chr2	152065934	152066250	Distal Intergenic
chr2	152085029	152085577	Distal Intergenic
chr2	152085690	152085889	Distal Intergenic
chr2	152109250	152109468	Exon
chr2	152157552	152157800	Distal Intergenic
chr2	152522413	152522819	Exon
chr2	152525435	152526038	Exon
chr2	152526235	152526544	Intron
chr2	152526692	152526953	Intron
chr2	152552690	152553065	Intron
chr2	152560166	152560683	Intron
chr2	152561055	152562585	Promoter
chr2	152562637	152565091	Promoter
chr2	152565167	152566120	Intron
chr2	152566842	152567936	Exon
chr2	152570136	152570581	Intron
chr2	152612692	152614546	Distal Intergenic
chr2	152618153	152618424	Distal Intergenic
chr2	152618598	152619103	Distal Intergenic
chr2	152635685	152636099	Distal Intergenic
chr2	152636142	152637205	Distal Intergenic
chr2	152646402	152648009	Intron
chr2	152655851	152656050	Intron
chr2	152656289	152657011	Intron
chr2	152673895	152674147	Intron

chr2	152714062	152714534	Intron
chr2	152714694	152714976	Intron
chr2	152715012	152716740	Intron
chr2	152987046	152987563	Intron
chr2	153168730	153168929	Distal Intergenic
chr2	153172521	153172760	Distal Intergenic
chr2	153567482	153567706	Intron
chr2	153575978	153576279	Promoter
chr2	154007729	154007954	Distal Intergenic
chr2	154008596	154008947	Distal Intergenic
chr2	154163879	154164239	Distal Intergenic
chr2	154164475	154164896	Distal Intergenic
chr2	154262364	154262630	Distal Intergenic
chr2	154289364	154289738	Distal Intergenic
chr2	154482791	154483773	Distal Intergenic
chr2	154483957	154484162	Distal Intergenic
chr2	154494816	154495274	Distal Intergenic
chr2	154604800	154605051	Distal Intergenic
chr2	154616856	154617055	Distal Intergenic
chr2	154717361	154717668	Distal Intergenic
chr2	154717817	154718635	Distal Intergenic
chr2	154899650	154899926	Intron
chr2	154899993	154900193	Intron
chr2	154900393	154900660	Intron
chr2	155062362	155062669	Intron
chr2	155417824	155418126	Distal Intergenic
chr2	155576441	155576703	Intron
chr2	155629694	155630038	Intron
chr2	155630306	155633179	Intron
chr2	155634891	155635742	Intron
chr2	155643933	155644356	Intron
chr2	155644451	155644928	Intron
chr2	155644976	155647597	Intron
chr2	155650771	155652506	Intron
chr2	155699801	155700227	Intron
chr2	155704415	155705119	Intron
chr2	155755864	155756183	Distal Intergenic
chr2	155804608	155805506	Distal Intergenic
chr2	155936710	155937322	Distal Intergenic
chr2	155937441	155937643	Distal Intergenic
chr2	155970017	155970673	Distal Intergenic
chr2	156004932	156005357	Distal Intergenic
chr2	156005507	156005960	Distal Intergenic
chr2	156058858	156059522	Distal Intergenic

chr2	156138088	156138561	Distal Intergenic
chr2	156217823	156218053	Distal Intergenic
chr2	156218090	156218656	Distal Intergenic
chr2	156272808	156273102	Distal Intergenic
chr2	156282314	156282531	Distal Intergenic
chr2	156282707	156283127	Distal Intergenic
chr2	156364743	156365054	Distal Intergenic
chr2	156365207	156365457	Distal Intergenic
chr2	156547164	156547615	Distal Intergenic
chr2	156547838	156548152	Distal Intergenic
chr2	156667838	156668197	Distal Intergenic
chr2	156670329	156670570	Distal Intergenic
chr2	156670620	156670851	Distal Intergenic
chr2	156688150	156688399	Distal Intergenic
chr2	156689263	156689732	Distal Intergenic
chr2	156871582	156871944	Distal Intergenic
chr2	156897424	156898974	Intron
chr2	156899141	156899362	Intron
chr2	156899718	156900790	Intron
chr2	157028009	157030545	Intron
chr2	157193679	157194071	Exon
chr2	157201894	157202375	Distal Intergenic
chr2	157218038	157218274	Distal Intergenic
chr2	157218347	157218809	Distal Intergenic
chr2	157236355	157239112	Distal Intergenic
chr2	157248612	157250222	Distal Intergenic
chr2	157250300	157252843	Distal Intergenic
chr2	157253244	157253581	Distal Intergenic
chr2	157253662	157253873	Distal Intergenic
chr2	157254044	157254539	Distal Intergenic
chr2	157255131	157256526	Distal Intergenic
chr2	157259002	157259211	Distal Intergenic
chr2	157259361	157260363	Distal Intergenic
chr2	157260415	157260789	Distal Intergenic
chr2	157271399	157273233	Distal Intergenic
chr2	157273409	157274570	Distal Intergenic
chr2	157275650	157275892	Distal Intergenic
chr2	157276518	157278178	Distal Intergenic
chr2	157289764	157290008	Distal Intergenic
chr2	157297185	157297569	Intron
chr2	157298599	157299243	Intron
chr2	157299282	157299544	Intron
chr2	157391735	157393738	Intron
chr2	157633348	157634409	Distal Intergenic

chr2	157636225	157636648	Distal Intergenic
chr2	157639987	157640720	Distal Intergenic
chr2	157671266	157672003	Distal Intergenic
chr2	157853809	157854133	Distal Intergenic
chr2	157854175	157854394	Distal Intergenic
chr2	157854778	157855204	Distal Intergenic
chr2	157958541	157959075	Distal Intergenic
chr2	157980498	157980842	Distal Intergenic
chr2	157985569	157985987	Distal Intergenic
chr2	157993356	157993766	Distal Intergenic
chr2	157993825	157994676	Distal Intergenic
chr2	157994720	157995460	Distal Intergenic
chr2	158186992	158187252	Distal Intergenic
chr2	158187380	158187602	Distal Intergenic
chr2	158241753	158242073	Distal Intergenic
chr2	158336237	158336901	Intron
chr2	158658979	158660532	Intron
chr2	158665619	158666196	Intron
chr2	158666356	158666839	Intron
chr2	158919151	158919679	Intron
chr2	159973514	159975266	Intron
chr2	159975462	159976062	Intron
chr2	160120907	160121106	Intron
chr2	160139019	160139373	Exon
chr2	160171580	160172253	Distal Intergenic
chr2	160445694	160446761	Intron
chr2	160446990	160447513	Intron
chr2	160456416	160456631	Intron
chr2	160456719	160457575	Intron
chr2	160472119	160472416	Promoter
chr2	160472468	160473081	Promoter
chr2	160571169	160571397	Intron
chr2	160571444	160573612	5' UTR
chr2	160713486	160713722	Intron
chr2	160788876	160789119	Distal Intergenic
chr2	160937479	160937728	Distal Intergenic
chr2	162098164	162099262	Distal Intergenic
chr2	162099310	162099710	Distal Intergenic
chr2	162108243	162108707	Distal Intergenic
chr2	162108800	162109037	Distal Intergenic
chr2	162766600	162766961	Intron
chr2	162900749	162901555	Intron
chr2	163239090	163239594	Intron
chr2	163561887	163562269	Intron

chr2	163821288	163821627	Distal Intergenic
chr2	163842583	163843812	Distal Intergenic
chr2	164182139	164182386	Distal Intergenic
chr2	164247027	164248015	Distal Intergenic
chr2	164256907	164259439	Distal Intergenic
chr2	164260529	164261270	Distal Intergenic
chr2	164264359	164264591	Distal Intergenic
chr2	164264903	164265190	Distal Intergenic
chr2	164344308	164344645	Distal Intergenic
chr2	164344849	164345352	Distal Intergenic
chr2	164347166	164348167	Distal Intergenic
chr2	164384388	164385157	Distal Intergenic
chr2	164499379	164500305	Intron
chr2	164500412	164500636	Intron
chr2	164561243	164561766	Intron
chr2	164562350	164564136	Intron
chr2	164610769	164611087	Distal Intergenic
chr2	164682261	164682761	Distal Intergenic
chr2	164683217	164683620	Distal Intergenic
chr2	164736679	164737813	Distal Intergenic
chr2	165147290	165151625	Distal Intergenic
chr2	165645745	165646328	Intron
chr2	165646477	165646738	Intron
chr2	165656270	165658697	Exon
chr2	165681104	165683916	Intron
chr2	165684033	165684873	Intron
chr2	165685053	165685252	Intron
chr2	165709145	165710209	Distal Intergenic
chr2	165710285	165710589	Distal Intergenic
chr2	165719960	165720270	Distal Intergenic
chr2	165979947	165980161	Intron
chr2	165995379	165995848	Intron
chr2	166255032	166255293	Distal Intergenic
chr2	166261059	166261753	Distal Intergenic
chr2	166731954	166732387	Intron
chr2	166889107	166889590	Intron
chr2	166890014	166890674	Intron
chr2	166891374	166891681	Intron
chr2	166892046	166892297	Intron
chr2	167246468	167247106	Distal Intergenic
chr2	167250331	167251339	Distal Intergenic
chr2	167311221	167312129	Intron
chr2	167395571	167396283	Distal Intergenic
chr2	167600049	167601147	Distal Intergenic

chr2	167602381	167604013	Distal Intergenic
chr2	167614475	167615980	Distal Intergenic
chr2	167623718	167623925	Distal Intergenic
chr2	167624581	167624789	Distal Intergenic
chr2	167625038	167625415	Distal Intergenic
chr2	167626118	167626729	Distal Intergenic
chr2	167642815	167643194	Distal Intergenic
chr2	167656555	167656867	Distal Intergenic
chr2	167659220	167659670	Distal Intergenic
chr2	167659983	167660557	Distal Intergenic
chr2	167665002	167665279	Distal Intergenic
chr2	167744260	167745046	Promoter
chr2	167745177	167746153	Promoter
chr2	167774492	167775010	Intron
chr2	167775064	167777175	Intron
chr2	167786623	167786922	Intron
chr2	168017054	168018773	Intron
chr2	168031197	168031910	Intron
chr2	168032374	168032981	Intron
chr2	168055877	168056115	Intron
chr2	168283199	168283468	Distal Intergenic
chr2	168283722	168284274	Distal Intergenic
chr2	168371296	168372070	Distal Intergenic
chr2	168373563	168373971	Distal Intergenic
chr2	168374335	168375012	Distal Intergenic
chr2	168745245	168745485	Distal Intergenic
chr2	168745701	168745983	Distal Intergenic
chr2	168780366	168782130	Distal Intergenic
chr2	168783770	168785258	Distal Intergenic
chr2	169414822	169416283	Intron
chr2	169979433	169979654	Distal Intergenic
chr2	169979713	169980223	Distal Intergenic
chr2	169980361	169981364	Downstream
chr2	170071258	170071877	Intron
chr2	170071930	170072324	Intron
chr2	170073232	170073816	Intron
chr2	170133196	170135217	Exon
chr2	170135509	170136362	Exon
chr2	172469298	172470121	Distal Intergenic
chr2	172686801	172687570	Intron
chr2	172688918	172689527	Intron
chr2	172762833	172763093	Distal Intergenic
chr2	172763137	172763371	Distal Intergenic
chr2	172763595	172763807	Distal Intergenic

chr2	173057302	173059459	Distal Intergenic
chr2	173071222	173071638	Distal Intergenic
chr2	173076320	173076665	Distal Intergenic
chr2	173082668	173084890	Distal Intergenic
chr2	173086193	173094579	Distal Intergenic
chr2	173099082	173099303	Distal Intergenic
chr2	173099815	173101214	Distal Intergenic
chr2	173106380	173107156	Distal Intergenic
chr2	173485817	173486161	Exon
chr2	173487798	173488549	Intron
chr2	173505717	173506098	Distal Intergenic
chr2	174155921	174156340	Distal Intergenic
chr2	179905062	179905743	Intron
chr2	179970097	179972457	3' UTR
chr2	180192662	180192911	Distal Intergenic
chr2	180315890	180316211	Intron
chr2	180316980	180317189	Intron
chr2	180436143	180437534	Intron
chr2	180444298	180444714	Intron
chr2	180922842	180923463	Distal Intergenic
chr2	180923627	180923884	Distal Intergenic
chr2	181021849	181022390	Distal Intergenic
chr2	181232239	181232448	Distal Intergenic
chr2	181232577	181232793	Distal Intergenic
chr2	181254476	181255121	Distal Intergenic
chr2	181255391	181255922	Distal Intergenic
chr2	181257351	181257949	Distal Intergenic
chr2	181259436	181260869	Distal Intergenic
chr2	181316010	181316209	Distal Intergenic
chr3	5575337	5575908	Distal Intergenic
chr3	7503080	7503338	5' UTR
chr3	7503460	7503814	Intron
chr3	7512556	7514272	Intron
chr3	8053246	8054390	Intron
chr3	8084132	8084447	Distal Intergenic
chr3	8084517	8085108	Distal Intergenic
chr3	8085145	8085372	Distal Intergenic
chr3	8307021	8307529	Intron
chr3	8440870	8441583	Intron
chr3	8919227	8920311	3' UTR
chr3	8988147	8989338	Exon
chr3	8989647	8989961	Intron
chr3	8993444	8994055	Intron
chr3	9001157	9002288	Intron

chr3	9003713	9004021	Intron
chr3	9014766	9014999	Distal Intergenic
chr3	9015122	9015422	Distal Intergenic
chr3	10258177	10259909	Exon
chr3	10263042	10265910	Exon
chr3	10273006	10275160	Intron
chr3	10276390	10280434	Exon
chr3	10292223	10293637	Intron
chr3	10294007	10294879	Intron
chr3	14610841	14611261	Distal Intergenic
chr3	14641887	14642442	Distal Intergenic
chr3	14647764	14649892	Distal Intergenic
chr3	14659313	14660616	Distal Intergenic
chr3	14947635	14948830	Intron
chr3	15007595	15008431	Intron
chr3	15452378	15452715	3' UTR
chr3	16083224	16084841	Intron
chr3	18113621	18114138	Intron
chr3	18115014	18115245	Intron
chr3	18115449	18116347	Intron
chr3	18239546	18239746	Intron
chr3	18275804	18277625	Intron
chr3	19081494	19083031	Distal Intergenic
chr3	19087390	19088171	Distal Intergenic
chr3	19088223	19088594	Distal Intergenic
chr3	19116060	19116396	Distal Intergenic
chr3	19145409	19145669	Distal Intergenic
chr3	19145802	19146166	Distal Intergenic
chr3	19146451	19146657	Distal Intergenic
chr3	19146708	19147955	Distal Intergenic
chr3	19161990	19165649	Distal Intergenic
chr3	19165746	19166095	Distal Intergenic
chr3	19166305	19166663	Distal Intergenic
chr3	19176550	19177576	Distal Intergenic
chr3	19208324	19208549	Intron
chr3	19208684	19211279	Intron
chr3	19269275	19269774	Intron
chr3	19305689	19308994	Intron
chr3	19310924	19311532	Intron
chr3	19324792	19326457	Intron
chr3	19326502	19327054	Intron
chr3	19327984	19330758	Intron
chr3	19351336	19351648	Intron
chr3	19356051	19357333	Promoter

chr3	19409073	19409339	Intron
chr3	19418999	19420747	Intron
chr3	19957305	19957539	Intron
chr3	21299644	21301054	Distal Intergenic
chr3	21302159	21302994	Distal Intergenic
chr3	21303055	21303959	Distal Intergenic
chr3	21304135	21304515	Distal Intergenic
chr3	21536758	21536957	Intron
chr3	21566483	21567339	Intron
chr3	21570031	21571285	Intron
chr3	21571330	21572185	Intron
chr3	21578281	21578554	Intron
chr3	21598207	21598555	Intron
chr3	21628143	21628711	Intron
chr3	21659793	21662026	Intron
chr3	21739787	21740216	Intron
chr3	21804554	21805127	Intron
chr3	21836883	21837777	Intron
chr3	21976235	21976449	Intron
chr3	21976781	21977001	Intron
chr3	23769248	23769699	Distal Intergenic
chr3	24503596	24503844	Intron
chr3	24593494	24593801	Distal Intergenic
chr3	27001986	27002185	Distal Intergenic
chr3	27002419	27003105	Distal Intergenic
chr3	27051805	27052219	Distal Intergenic
chr3	27052530	27052788	Distal Intergenic
chr3	27052903	27054450	Distal Intergenic
chr3	27108179	27109944	Distal Intergenic
chr3	27487182	27487896	Intron
chr3	27489124	27490001	Intron
chr3	27490054	27490378	Exon
chr3	27515346	27515941	Intron
chr3	27550374	27553181	Distal Intergenic
chr3	27555585	27556040	Distal Intergenic
chr3	27556304	27556572	Distal Intergenic
chr3	27608380	27608883	Distal Intergenic
chr3	27609446	27609846	Distal Intergenic
chr3	27762198	27762397	Intron
chr3	27881280	27882256	Distal Intergenic
chr3	27882541	27883180	Distal Intergenic
chr3	27887464	27888084	Distal Intergenic
chr3	27907008	27908347	Distal Intergenic
chr3	27920755	27921225	Distal Intergenic

chr3	28088487	28088743	Distal Intergenic
chr3	28366562	28367025	Intron
chr3	28633413	28634244	Intron
chr3	28704379	28704779	Intron
chr3	28705151	28705564	Intron
chr3	28705661	28706073	Intron
chr3	29778379	29778812	Intron
chr3	29778891	29779404	Intron
chr3	29788558	29790752	Intron
chr3	29802189	29803513	Intron
chr3	29852384	29852631	Intron
chr3	30501529	30501956	Distal Intergenic
chr3	30691794	30692012	5' UTR
chr3	30692061	30692293	Intron
chr3	30692550	30692887	Intron
chr3	30828820	30829304	Intron
chr3	30867372	30867728	Intron
chr3	30895088	30896132	Intron
chr3	30896455	30896683	Intron
chr3	30896863	30899547	Exon
chr3	30917062	30918742	Intron
chr3	30920859	30922114	Intron
chr3	30932535	30933338	Intron
chr3	30937077	30938094	Promoter
chr3	30960128	30960900	Distal Intergenic
chr3	30992688	30993122	Distal Intergenic
chr3	30994254	30995429	Distal Intergenic
chr3	30995492	30995890	Distal Intergenic
chr3	31032677	31034782	Distal Intergenic
chr3	31095899	31096594	Distal Intergenic
chr3	31096638	31096902	Distal Intergenic
chr3	31159057	31160047	Distal Intergenic
chr3	31225680	31226906	Distal Intergenic
chr3	31336640	31338173	Distal Intergenic
chr3	31390479	31390945	Distal Intergenic
chr3	31394462	31394826	Distal Intergenic
chr3	32239386	32239639	Distal Intergenic
chr3	32241188	32241518	Distal Intergenic
chr3	32241596	32242140	Distal Intergenic
chr3	32262904	32263870	Distal Intergenic
chr3	32263937	32264233	Distal Intergenic
chr3	32295243	32295442	Intron
chr3	32314617	32315020	Intron
chr3	32316606	32316806	Intron

chr3	32409924	32410168	Intron
chr3	33464266	33464488	Intron
chr3	33698409	33698953	Intron
chr3	34310214	34311015	Distal Intergenic
chr3	34549610	34549839	Distal Intergenic
chr3	34550427	34550902	Distal Intergenic
chr3	34551303	34551568	Distal Intergenic
chr3	34551640	34551875	Distal Intergenic
chr3	34552460	34553000	Distal Intergenic
chr3	34723549	34724669	Distal Intergenic
chr3	34784339	34787456	Distal Intergenic
chr3	34791951	34793082	Distal Intergenic
chr3	35129621	35130394	Distal Intergenic
chr3	35130679	35132474	Distal Intergenic
chr3	35133952	35134206	Distal Intergenic
chr3	35144453	35146028	Distal Intergenic
chr3	35619339	35620433	Distal Intergenic
chr3	35620828	35621168	Distal Intergenic
chr3	35797023	35797418	Intron
chr3	35830945	35831442	Intron
chr3	35831528	35831727	Intron
chr3	35832127	35832618	Intron
chr3	36285964	36286744	Distal Intergenic
chr3	36471208	36471515	Intron
chr3	37211613	37212077	Intron
chr3	37212113	37212926	Intron
chr3	37319760	37320159	Intron
chr3	37372028	37372258	Intron
chr3	37394959	37395907	Intron
chr3	37400091	37400360	Intron
chr3	37400930	37403792	3' UTR
chr3	37805803	37806150	Intron
chr3	38175482	38175681	Exon
chr3	38175809	38176829	Intron
chr3	38181807	38183919	3' UTR
chr3	38271947	38273351	3' UTR
chr3	38280654	38282687	Intron
chr3	38341413	38342812	Intron
chr3	38380997	38381296	Distal Intergenic
chr3	38382483	38383437	Distal Intergenic
chr3	38412905	38413134	Intron
chr3	38460286	38460903	Distal Intergenic
chr3	38515484	38516734	Intron
chr3	39008138	39009042	Distal Intergenic

chr3	39168537	39168937	Intron
chr3	40485807	40487121	Exon
chr3	40491841	40492118	Intron
chr3	40498009	40498716	Promoter
chr3	40549770	40550172	Intron
chr3	40550208	40550458	Intron
chr3	40564841	40565211	Distal Intergenic
chr3	40603255	40603881	Distal Intergenic
chr3	40887417	40888029	Distal Intergenic
chr3	40997983	40999518	Distal Intergenic
chr3	41004052	41004594	Distal Intergenic
chr3	41024281	41025092	Distal Intergenic
chr3	41088413	41090104	Distal Intergenic
chr3	41092013	41093060	Distal Intergenic
chr3	41116168	41116786	Distal Intergenic
chr3	41121655	41123691	Distal Intergenic
chr3	41178573	41180083	Distal Intergenic
chr3	41180771	41181007	Distal Intergenic
chr3	41240491	41240759	Promoter
chr3	41313133	41313796	Intron
chr3	41325449	41327118	Intron
chr3	41351072	41351295	Intron
chr3	41351388	41351662	Intron
chr3	41367909	41368196	Intron
chr3	41409719	41410100	Intron
chr3	47466143	47467074	Exon
chr3	49635992	49636191	Intron
chr3	49805512	49806521	Intron
chr3	50241728	50242711	Promoter
chr3	50260137	50261690	Distal Intergenic
chr3	50352664	50353056	Downstream
chr3	51027520	51027731	Intron
chr3	51027845	51028051	Intron
chr3	51080981	51082045	Intron
chr3	51131485	51131684	Intron
chr3	51142317	51142654	Intron
chr3	51147091	51147370	Intron
chr3	51212503	51212702	Intron
chr3	51215852	51218106	Intron
chr3	51218657	51221017	Intron
chr3	51287337	51287857	Intron
chr3	51364084	51364810	Intron
chr3	51377902	51378479	Intron
chr3	51464702	51465587	Exon

chr3	51465788	51466264	Intron
chr3	51469246	51469923	Intron
chr3	51525206	51525676	Intron
chr3	51646040	51646979	Intron
chr3	51709189	51710459	Intron
chr3	51710976	51712070	Intron
chr3	51712111	51712947	Intron
chr3	51738634	51739498	Distal Intergenic
chr3	51747184	51747406	Exon
chr3	51747595	51748062	Exon
chr3	51821673	51822031	Distal Intergenic
chr3	51849859	51851641	Promoter
chr3	51851841	51852532	Promoter
chr3	51873531	51874995	Intron
chr3	51892348	51892733	Intron
chr3	51892805	51893013	Intron
chr3	51906731	51906930	Intron
chr3	51944355	51944554	Distal Intergenic
chr3	51954745	51955645	Distal Intergenic
chr3	51961994	51963212	Distal Intergenic
chr3	52000521	52000888	Promoter
chr3	52001530	52002057	Promoter
chr3	52002220	52003317	Promoter
chr3	52101683	52102048	Distal Intergenic
chr3	52106861	52107122	Downstream
chr3	52146747	52147561	Intron
chr3	52407307	52407529	Intron
chr3	52411912	52412174	Intron
chr3	52434096	52434602	3' UTR
chr3	52434643	52434993	Downstream
chr3	52867101	52867494	Promoter
chr3	52867600	52870117	Promoter
chr3	52912339	52912603	Intron
chr3	52912827	52913070	Intron
chr3	53004868	53006777	Intron
chr3	53007049	53007405	Intron
chr3	53007742	53009111	Intron
chr3	53009171	53009374	Intron
chr3	53266886	53267085	Intron
chr3	53267131	53267450	Exon
chr3	53667484	53667943	Intron
chr3	54131740	54131948	Distal Intergenic
chr3	54235711	54235937	Intron
chr3	54270492	54271458	Intron

chr3	54283761	54285398	Intron
chr3	54497270	54497520	Intron
chr3	54539828	54540070	Intron
chr3	54916106	54917238	Intron
chr3	54917761	54918072	Intron
chr3	54918184	54918405	Intron
chr3	54926169	54927559	Intron
chr3	55204389	55205652	Distal Intergenic
chr3	55392031	55392246	Distal Intergenic
chr3	55394247	55394648	Distal Intergenic
chr3	55396704	55397882	Distal Intergenic
chr3	55399669	55401007	Distal Intergenic
chr3	55403718	55404362	Distal Intergenic
chr3	55420640	55420875	Distal Intergenic
chr3	55421071	55421322	Distal Intergenic
chr3	55691686	55691936	Exon
chr3	55989567	55990091	Intron
chr3	55990476	55990841	Intron
chr3	55991062	55991826	Intron
chr3	56765937	56766635	3' UTR
chr3	56766782	56768041	Intron
chr3	56897119	56898024	Intron
chr3	57098604	57098910	Intron
chr3	57098949	57099184	Intron
chr3	57244794	57245390	Distal Intergenic
chr3	57304727	57305992	3' UTR
chr3	57358069	57359185	Intron
chr3	57359771	57360080	Intron
chr3	57539247	57539516	Distal Intergenic
chr3	57540084	57540350	Distal Intergenic
chr3	58007950	58008976	Intron
chr3	58014530	58015822	Intron
chr3	58174488	58177580	Downstream
chr3	58218058	58218522	Distal Intergenic
chr3	58219659	58220525	Distal Intergenic
chr3	58220593	58220860	Distal Intergenic
chr3	58220922	58221444	Distal Intergenic
chr3	58221518	58223929	Promoter
chr3	58223974	58224791	Promoter
chr3	58224961	58225224	Intron
chr3	58225314	58226210	Intron
chr3	58227071	58227485	Intron
chr3	58227661	58227860	Intron
chr3	58272827	58275234	Intron

chr3	58380899	58381288	Intron
chr3	58917771	58919000	Intron
chr3	58924676	58925991	Intron
chr3	58972480	58973469	Intron
chr3	58973540	58974677	Intron
chr3	58991282	58991899	Intron
chr3	58991975	58992292	Intron
chr3	58992374	58992690	Intron
chr3	58992982	58993456	Intron
chr3	58994154	58994477	Intron
chr3	58994628	58999457	Intron
chr3	58999625	58999829	Intron
chr3	59000311	59002696	Intron
chr3	59007557	59008558	Intron
chr3	59082440	59083174	Distal Intergenic
chr3	59092126	59095079	Distal Intergenic
chr3	59099444	59101068	Distal Intergenic
chr3	59116455	59116726	Distal Intergenic
chr3	59519599	59521118	Distal Intergenic
chr3	59856903	59858888	Intron
chr3	60150003	60151479	Intron
chr3	60163689	60163891	Intron
chr3	60163935	60164167	Intron
chr3	60167236	60168726	Intron
chr3	60192104	60193640	Intron
chr3	60212307	60213137	Intron
chr3	60227719	60228725	Intron
chr3	60290331	60290602	Intron
chr3	60302740	60304863	Intron
chr3	60305455	60309129	Intron
chr3	60321917	60322854	Intron
chr3	60374135	60374495	Intron
chr3	60406981	60407333	Intron
chr3	60419671	60420070	Intron
chr3	60420584	60421675	Intron
chr3	60421720	60422320	Intron
chr3	60436851	60437050	Intron
chr3	60602151	60603557	Intron
chr3	60690926	60691791	Intron
chr3	60839459	60840619	Intron
chr3	61020527	61020763	Intron
chr3	61143966	61144754	Intron
chr3	61147620	61147965	Intron
chr3	61148272	61148887	Intron

chr3	61166354	61167321	Intron
chr3	61167365	61167646	Intron
chr3	61167733	61168243	Intron
chr3	61168524	61168810	Intron
chr3	61168951	61169482	Intron
chr3	61169683	61170128	Intron
chr3	61179532	61180126	Intron
chr3	61270190	61270457	Distal Intergenic
chr3	61270550	61271009	Distal Intergenic
chr3	63767891	63768408	Distal Intergenic
chr3	63780085	63780516	Distal Intergenic
chr3	64142646	64142854	Exon
chr3	64142896	64143295	Exon
chr3	65195323	65195569	Distal Intergenic
chr3	65195740	65195953	Distal Intergenic
chr3	65569696	65571110	Intron
chr3	65645073	65645480	Intron
chr3	65646883	65650770	Intron
chr3	65689239	65689695	Intron
chr3	65726417	65727741	Intron
chr3	65728771	65729942	Intron
chr3	65730298	65730582	Intron
chr3	65730689	65732403	Intron
chr3	65732847	65733495	Intron
chr3	65760683	65761562	Intron
chr3	65778090	65778688	Intron
chr3	67267005	67267542	Distal Intergenic
chr3	68296511	68297714	Intron
chr3	68298151	68298861	Intron
chr3	68299057	68299766	Intron
chr3	68308630	68308829	Intron
chr3	68311452	68313084	Intron
chr3	68315420	68316172	Intron
chr3	68338203	68338402	Intron
chr3	68338720	68339000	Intron
chr3	68808874	68809912	Intron
chr3	68846712	68847039	Intron
chr3	68847177	68847431	Intron
chr3	68847492	68852104	Intron
chr3	68929729	68930381	Exon
chr3	68985313	68985538	Distal Intergenic
chr3	68985797	68986225	Distal Intergenic
chr3	69515659	69515902	Intron
chr3	69516285	69516484	Intron

chr3	69525959	69526182	Intron
chr3	69526480	69526755	Intron
chr3	70607138	70608205	Distal Intergenic
chr3	70608351	70608589	Distal Intergenic
chr3	75303502	75303701	Distal Intergenic
chr3	75733794	75735259	Distal Intergenic
chr3	75735979	75736311	Distal Intergenic
chr3	75739893	75740690	Distal Intergenic
chr3	75740804	75744683	Distal Intergenic
chr3	75745752	75747513	Distal Intergenic
chr3	75752361	75752656	Distal Intergenic
chr3	75756703	75757099	Downstream
chr3	75758226	75758449	Downstream
chr3	75759199	75759785	Exon
chr3	75759844	75760252	Exon
chr3	75760391	75760711	Exon
chr3	75789582	75791055	Exon
chr3	78933372	78933579	Intron
chr3	78956874	78957708	Intron
chr3	78984994	78986601	Intron
chr3	79051715	79053097	Intron
chr3	79198436	79198835	Intron
chr3	79198943	79199173	Intron
chr3	79294119	79294463	Intron
chr3	79362944	79363162	Intron
chr3	79363378	79363832	Intron
chr3	79395456	79395657	Intron
chr3	79395808	79396112	Intron
chr3	80687273	80688392	Distal Intergenic
chr3	80861775	80863156	Distal Intergenic
chr3	80910457	80910711	Distal Intergenic
chr3	80910779	80911090	Distal Intergenic
chr3	80947661	80947911	Distal Intergenic
chr3	80967765	80968192	Distal Intergenic
chr3	80988407	80989334	Distal Intergenic
chr3	81004412	81005354	Distal Intergenic
chr3	81009083	81009808	Distal Intergenic
chr3	81721623	81721945	Intron
chr3	81722006	81722295	Intron
chr3	83579026	83579236	Distal Intergenic
chr3	83666800	83667425	Distal Intergenic
chr3	83669271	83669615	Distal Intergenic
chr3	83747463	83748042	Distal Intergenic
chr3	83791430	83792310	Distal Intergenic

chr3	83797177	83797497	Distal Intergenic
chr3	83797812	83798097	Distal Intergenic
chr3	83825316	83827852	Distal Intergenic
chr3	83911922	83913211	Distal Intergenic
chr3	84097347	84097549	Distal Intergenic
chr3	84119060	84119325	Distal Intergenic
chr3	84122314	84123787	Distal Intergenic
chr3	84127398	84129297	Distal Intergenic
chr3	84284477	84284911	Distal Intergenic
chr3	84285075	84285310	Distal Intergenic
chr3	84307265	84307490	Distal Intergenic
chr3	84405950	84407802	Distal Intergenic
chr3	84449888	84450233	Distal Intergenic
chr3	84499190	84500519	Distal Intergenic
chr3	84500577	84501000	Distal Intergenic
chr3	84644242	84644441	Distal Intergenic
chr3	84644562	84644875	Distal Intergenic
chr3	84755640	84756483	Intron
chr3	84774692	84776633	Intron
chr3	84776731	84778780	Intron
chr3	84826838	84827187	Intron
chr3	84828414	84828805	Intron
chr3	85087525	85089021	Intron
chr3	85172037	85173947	Intron
chr3	85282482	85282850	Intron
chr3	85282991	85283265	Intron
chr3	85377983	85378190	Intron
chr3	85595958	85597497	Intron
chr3	85684561	85685299	Intron
chr3	85735433	85736149	Intron
chr3	85769426	85769630	Intron
chr3	85797960	85798457	Intron
chr3	85844273	85845175	Intron
chr3	86057178	86057511	Intron
chr3	86654595	86655334	Distal Intergenic
chr3	86876083	86878174	Distal Intergenic
chr3	86883380	86884299	Distal Intergenic
chr3	86903905	86904595	Distal Intergenic
chr3	86904634	86905584	Distal Intergenic
chr3	86916748	86917028	Distal Intergenic
chr3	86944670	86946175	Distal Intergenic
chr3	86953798	86955930	Distal Intergenic
chr3	86961859	86962909	Distal Intergenic
chr3	86977249	86977587	Distal Intergenic

chr3	86977633	86977879	Distal Intergenic
chr3	87322859	87323337	Intron
chr3	87369500	87370326	Distal Intergenic
chr3	87480179	87480480	Distal Intergenic
chr3	87689134	87689768	Distal Intergenic
chr3	87710775	87714544	Distal Intergenic
chr3	87714664	87715090	Distal Intergenic
chr3	87723156	87723359	Distal Intergenic
chr3	87723472	87723922	Distal Intergenic
chr3	87746470	87746747	Distal Intergenic
chr3	87748779	87749109	Distal Intergenic
chr3	87839352	87840176	Distal Intergenic
chr3	87942164	87942363	Distal Intergenic
chr3	87942583	87945475	Distal Intergenic
chr3	87945546	87945901	Distal Intergenic
chr3	87945968	87946218	Distal Intergenic
chr3	87947176	87947565	Distal Intergenic
chr3	87981091	87981404	Distal Intergenic
chr3	87981771	87981994	Distal Intergenic
chr3	88100108	88100699	Downstream
chr3	88101144	88101371	3' UTR
chr3	88137568	88139491	Intron
chr3	88139675	88140108	Intron
chr3	88140675	88141135	Intron
chr3	88141272	88141585	Intron
chr3	88220238	88221056	Distal Intergenic
chr3	88225919	88226447	Distal Intergenic
chr3	88292363	88293451	Distal Intergenic
chr3	88294643	88297057	Distal Intergenic
chr3	88297114	88300959	Distal Intergenic
chr3	88303923	88305165	Distal Intergenic
chr3	88305303	88306908	Distal Intergenic
chr3	88307336	88307596	Distal Intergenic
chr3	88313642	88314008	Distal Intergenic
chr3	88314084	88314905	Distal Intergenic
chr3	88315538	88316230	Distal Intergenic
chr3	88316283	88316629	Distal Intergenic
chr3	88316686	88317990	Distal Intergenic
chr3	88357112	88357314	Distal Intergenic
chr3	88395893	88398746	Distal Intergenic
chr3	88425503	88426815	Distal Intergenic
chr3	88427755	88428450	Distal Intergenic
chr3	88428487	88429331	Distal Intergenic
chr3	88457845	88458615	Distal Intergenic

chr3	88489306	88489622	Distal Intergenic
chr3	88489704	88490503	Distal Intergenic
chr3	88717935	88718134	Distal Intergenic
chr3	88754846	88755088	Distal Intergenic
chr3	88906040	88906411	Distal Intergenic
chr3	88906447	88906779	Distal Intergenic
chr3	89011790	89012545	Distal Intergenic
chr3	89017603	89017802	Distal Intergenic
chr3	89062942	89063404	Distal Intergenic
chr3	89081301	89083487	Distal Intergenic
chr3	89086038	89086295	Distal Intergenic
chr3	89132197	89133212	Distal Intergenic
chr3	89182993	89183262	Intron
chr3	89195447	89196497	Intron
chr3	89196554	89197168	Intron
chr3	89197397	89197628	Intron
chr3	89197726	89198331	Intron
chr3	89198387	89198632	Intron
chr3	89199124	89199354	Intron
chr3	89215554	89215801	Intron
chr3	89222110	89222376	Intron
chr3	89222428	89224950	Intron
chr3	89225173	89225395	Intron
chr3	89262433	89262982	Intron
chr3	89263457	89263682	Intron
chr3	89489300	89490303	Intron
chr3	89599598	89599921	Distal Intergenic
chr3	89611261	89612863	Distal Intergenic
chr3	89616837	89617236	Distal Intergenic
chr3	89665096	89665812	Distal Intergenic
chr3	89684016	89684728	Distal Intergenic
chr3	89699332	89703050	Distal Intergenic
chr3	89705218	89706251	Distal Intergenic
chr3	89716260	89717679	Distal Intergenic
chr3	89766244	89766908	Distal Intergenic
chr3	89767263	89768311	Distal Intergenic
chr3	89802321	89802581	Distal Intergenic
chr3	89802643	89802894	Distal Intergenic
chr3	89855435	89856017	Distal Intergenic
chr3	89883588	89884681	Distal Intergenic
chr3	89885214	89886141	Distal Intergenic
chr3	89990862	89991114	Distal Intergenic
chr3	89991167	89992539	Distal Intergenic
chr3	90012240	90013698	Distal Intergenic

chr3	90013789	90014319	Distal Intergenic
chr3	90014670	90015238	Distal Intergenic
chr3	90015727	90016608	Distal Intergenic
chr3	90017051	90017622	Distal Intergenic
chr3	90033057	90033368	Distal Intergenic
chr3	90049526	90050355	Distal Intergenic
chr3	90051607	90051985	Distal Intergenic
chr3	90052413	90052714	Distal Intergenic
chr3	90069342	90069687	Distal Intergenic
chr3	90096377	90096885	Distal Intergenic
chr3	90237147	90237480	Distal Intergenic
chr3	90312676	90312897	Distal Intergenic
chr3	90313196	90313423	Distal Intergenic
chr3	90313578	90313782	Distal Intergenic
chr3	90329832	90330224	Distal Intergenic
chr3	90330264	90331687	Distal Intergenic
chr3	90341082	90342146	Distal Intergenic
chr3	90342182	90342381	Distal Intergenic
chr3	90378814	90379407	Distal Intergenic
chr3	90379538	90379783	Distal Intergenic
chr3	90389324	90389616	Distal Intergenic
chr3	90389797	90394292	Distal Intergenic
chr3	90401945	90402167	Distal Intergenic
chr3	90404135	90405629	Distal Intergenic
chr3	90408074	90409160	Distal Intergenic
chr3	90416372	90416925	Distal Intergenic
chr3	90417018	90419420	Distal Intergenic
chr3	91816656	91817297	Distal Intergenic
chr3	91817358	91818027	Distal Intergenic
chr3	91838056	91838409	Distal Intergenic
chr3	91973403	91973950	Distal Intergenic
chr3	91974457	91974683	Distal Intergenic
chr3	92014785	92017021	Distal Intergenic
chr3	92017311	92018032	Distal Intergenic
chr3	92018138	92018526	Distal Intergenic
chr3	92018709	92019892	Distal Intergenic
chr3	92048302	92048715	Distal Intergenic
chr3	92048763	92051842	Distal Intergenic
chr3	92052114	92052859	Distal Intergenic
chr3	92052983	92053500	Distal Intergenic
chr3	92053553	92054918	Distal Intergenic
chr3	92082794	92086536	Distal Intergenic
chr3	92086816	92087500	Distal Intergenic
chr3	92088172	92089543	Distal Intergenic

chr3	92213919	92215309	Distal Intergenic
chr3	92219062	92219429	Distal Intergenic
chr3	92221318	92221667	Distal Intergenic
chr3	92244437	92245316	Distal Intergenic
chr3	92259095	92259564	Distal Intergenic
chr3	92317404	92317793	Distal Intergenic
chr3	92358179	92358456	Distal Intergenic
chr3	92358636	92358843	Distal Intergenic
chr3	92377916	92378115	Distal Intergenic
chr3	92383508	92383775	Distal Intergenic
chr3	92389904	92391022	Distal Intergenic
chr3	92391089	92391696	Distal Intergenic
chr3	92391880	92393454	Distal Intergenic
chr3	92413093	92413331	Distal Intergenic
chr3	92413387	92413924	Distal Intergenic
chr3	92413961	92414267	Distal Intergenic
chr3	92457341	92457740	Distal Intergenic
chr3	92626974	92627716	Distal Intergenic
chr3	92697980	92699581	Distal Intergenic
chr3	92827532	92827889	Distal Intergenic
chr3	92828033	92828280	Distal Intergenic
chr3	92829179	92829601	Distal Intergenic
chr3	92830083	92830282	Distal Intergenic
chr3	92948274	92949147	Distal Intergenic
chr3	92980509	92980949	Distal Intergenic
chr3	93233187	93234081	Distal Intergenic
chr3	93235818	93236152	Distal Intergenic
chr3	93310879	93311110	Distal Intergenic
chr3	93311146	93311741	Distal Intergenic
chr3	93323778	93326219	Distal Intergenic
chr3	93326469	93326874	Distal Intergenic
chr3	93340759	93341153	Distal Intergenic
chr3	93341377	93343230	Distal Intergenic
chr3	93359079	93360034	Distal Intergenic
chr3	93360451	93360657	Distal Intergenic
chr3	93375847	93376075	Distal Intergenic
chr3	93383719	93383924	Distal Intergenic
chr3	93384220	93385441	Distal Intergenic
chr3	94286465	94286853	Distal Intergenic
chr3	94368561	94369750	Distal Intergenic
chr3	94415019	94415825	Distal Intergenic
chr3	94421808	94422017	Distal Intergenic
chr3	94422089	94422589	Distal Intergenic
chr3	94425535	94426136	Distal Intergenic

chr3	94428259	94428613	Distal Intergenic
chr3	94432749	94433322	Distal Intergenic
chr3	94433519	94434590	Distal Intergenic
chr3	94462325	94462767	Distal Intergenic
chr3	94462877	94463735	Distal Intergenic
chr3	94561502	94561874	Distal Intergenic
chr3	94562226	94562528	Distal Intergenic
chr3	94584325	94585869	Distal Intergenic
chr3	94586104	94586341	Distal Intergenic
chr3	94586452	94588151	Distal Intergenic
chr3	94757206	94757789	Intron
chr3	94819554	94819844	Intron
chr3	94819887	94820119	Intron
chr3	94846084	94846426	Intron
chr3	94857877	94858093	Intron
chr3	94858242	94859830	Intron
chr3	94869860	94870635	Intron
chr3	94870711	94871131	Intron
chr3	94871211	94871565	Intron
chr3	94909067	94910828	Distal Intergenic
chr3	94912575	94913941	Distal Intergenic
chr3	94914813	94915350	Distal Intergenic
chr3	94937558	94937958	Distal Intergenic
chr3	95021372	95022695	Distal Intergenic
chr3	95031170	95031473	Distal Intergenic
chr3	95031597	95033810	Distal Intergenic
chr3	95033975	95034621	Distal Intergenic
chr3	95034718	95035697	Distal Intergenic
chr3	95036127	95036852	Distal Intergenic
chr3	95036935	95037228	Distal Intergenic
chr3	95065611	95066796	Distal Intergenic
chr3	95086385	95086818	Distal Intergenic
chr3	95238030	95238251	Distal Intergenic
chr3	95454497	95455407	Distal Intergenic
chr3	95463000	95463375	Distal Intergenic
chr3	95463431	95463972	Distal Intergenic
chr3	95465058	95465323	Distal Intergenic
chr3	95467061	95467338	Distal Intergenic
chr3	95467633	95469725	Distal Intergenic
chr3	95469970	95472103	Distal Intergenic
chr3	95477866	95478173	Distal Intergenic
chr3	95478210	95479885	Distal Intergenic
chr3	95479932	95480222	Distal Intergenic
chr3	95500238	95501135	Distal Intergenic

chr3	95505760	95506720	Distal Intergenic
chr3	95529201	95531060	Distal Intergenic
chr3	95537563	95538515	Distal Intergenic
chr3	95542903	95543511	Distal Intergenic
chr3	95562091	95563526	Distal Intergenic
chr3	95564857	95565350	Distal Intergenic
chr3	95565387	95566904	Distal Intergenic
chr3	95621885	95622883	Distal Intergenic
chr3	95623813	95624127	Distal Intergenic
chr3	95659541	95659740	Distal Intergenic
chr3	95891178	95892042	Distal Intergenic
chr3	95897881	95898105	Distal Intergenic
chr3	95963534	95965451	Distal Intergenic
chr3	95965504	95965736	Distal Intergenic
chr3	95975931	95976705	Distal Intergenic
chr3	96001228	96001706	Distal Intergenic
chr3	96023985	96024824	Distal Intergenic
chr3	96030602	96031277	Distal Intergenic
chr3	96031371	96031571	Distal Intergenic
chr3	96041303	96042852	Distal Intergenic
chr3	96043176	96043816	Distal Intergenic
chr3	96048462	96049074	Distal Intergenic
chr3	96049678	96050412	Distal Intergenic
chr3	96051377	96051622	Distal Intergenic
chr3	96051701	96052025	Distal Intergenic
chr3	96065680	96068550	Distal Intergenic
chr3	96071908	96073254	Distal Intergenic
chr3	96077782	96078094	Distal Intergenic
chr3	96218317	96218733	Distal Intergenic
chr3	96433879	96434078	Distal Intergenic
chr3	96477261	96480213	Distal Intergenic
chr3	96482701	96483446	Distal Intergenic
chr3	96483870	96484089	Distal Intergenic
chr3	96617642	96618024	Intron
chr3	97438526	97438836	Intron
chr3	97631812	97632354	Intron
chr3	97736727	97739079	Intron
chr3	97739258	97739483	Intron
chr3	97741105	97742590	Intron
chr3	97818245	97818485	Distal Intergenic
chr3	97824447	97824651	Distal Intergenic
chr3	97824821	97825061	Distal Intergenic
chr3	97831730	97831949	Distal Intergenic
chr3	97837389	97837722	Distal Intergenic

chr3	97860594	97861462	Distal Intergenic
chr3	97883816	97884981	Distal Intergenic
chr3	98080390	98080775	Distal Intergenic
chr3	98178024	98179886	Distal Intergenic
chr3	100488518	100488776	Intron
chr3	100507569	100507813	Intron
chr3	100600801	100602193	Intron
chr3	100652369	100652568	Intron
chr3	100743825	100745942	Distal Intergenic
chr3	100814575	100814801	Distal Intergenic
chr3	100826406	100827595	Distal Intergenic
chr3	100835564	100836276	Distal Intergenic
chr3	100837502	100838088	Distal Intergenic
chr3	100851145	100851950	Distal Intergenic
chr3	100852092	100852998	Distal Intergenic
chr3	100853090	100853534	Distal Intergenic
chr3	100855459	100855719	Distal Intergenic
chr3	100874488	100874761	Distal Intergenic
chr3	100877272	100878729	Distal Intergenic
chr3	100900259	100900563	Distal Intergenic
chr3	100919745	100920063	Distal Intergenic
chr3	100920241	100920551	Distal Intergenic
chr3	101116700	101117421	Intron
chr3	101117573	101118080	Exon
chr3	101122254	101122591	Intron
chr3	101122867	101123418	Intron
chr3	101125706	101126508	Intron
chr3	101150180	101151507	Intron
chr3	101246517	101246752	Distal Intergenic
chr3	101247127	101247623	Distal Intergenic
chr3	101251717	101251916	Distal Intergenic
chr3	101252100	101252940	Distal Intergenic
chr3	101254424	101254845	Distal Intergenic
chr3	101254930	101255180	Distal Intergenic
chr3	101265228	101266672	Distal Intergenic
chr3	101374947	101375499	Exon
chr3	101375645	101376406	Intron
chr3	101376459	101376803	Intron
chr3	101376904	101377107	Intron
chr3	101382426	101382689	Intron
chr3	101404365	101404673	Promoter
chr3	101405652	101405864	Promoter
chr3	101405980	101406270	Promoter
chr3	101407253	101407490	Distal Intergenic

chr3	101407801	101408075	Distal Intergenic
chr3	101439783	101441391	Distal Intergenic
chr3	101494885	101496630	Distal Intergenic
chr3	101503782	101504140	Intron
chr3	101504300	101505415	5' UTR
chr3	101505516	101505809	Intron
chr3	101632237	101635313	Distal Intergenic
chr3	101635549	101636303	Distal Intergenic
chr3	101639170	101639594	Distal Intergenic
chr3	101639732	101640518	Distal Intergenic
chr3	101842121	101844188	Intron
chr3	101907549	101908903	Intron
chr3	101912205	101912409	Intron
chr3	101915506	101917489	Intron
chr3	101939282	101939937	Intron
chr3	101940000	101940482	Intron
chr3	101945399	101947329	5' UTR
chr3	101955729	101956999	Intron
chr3	101963002	101964771	Intron
chr3	101973000	101973249	Intron
chr3	101973351	101974348	Intron
chr3	101974501	101975803	Intron
chr3	101976318	101976666	Intron
chr3	101976702	101977451	Intron
chr3	101977871	101978986	Intron
chr3	101981774	101983090	Intron
chr3	101991674	101992481	Intron
chr3	101992520	101992933	Intron
chr3	101996828	101997287	Intron
chr3	101997384	101998655	Intron
chr3	101999277	101999519	Intron
chr3	102007507	102007859	Intron
chr3	102136915	102137282	5' UTR
chr3	102140597	102141870	Intron
chr3	102142012	102142368	Intron
chr3	102168148	102168758	Intron
chr3	102176005	102176222	Intron
chr3	102176259	102177198	Exon
chr3	102178405	102178683	Intron
chr3	102178752	102179044	Intron
chr3	102278678	102278969	Distal Intergenic
chr3	102281353	102282203	Distal Intergenic
chr3	102471782	102472238	Distal Intergenic
chr3	102474691	102475105	Distal Intergenic

chr3	102492250	102492460	Distal Intergenic
chr3	102823935	102824220	Distal Intergenic
chr3	102824505	102824879	Distal Intergenic
chr3	102825007	102825488	Distal Intergenic
chr3	102859756	102859990	Distal Intergenic
chr3	102862243	102863098	Distal Intergenic
chr3	102904626	102905767	Distal Intergenic
chr3	102906022	102906507	Distal Intergenic
chr3	102906545	102907240	Distal Intergenic
chr3	102933982	102934770	Distal Intergenic
chr3	102943221	102944627	Distal Intergenic
chr3	102944845	102945961	Distal Intergenic
chr3	102949348	102951690	Distal Intergenic
chr3	103045744	103046052	Distal Intergenic
chr3	103059966	103060242	Distal Intergenic
chr3	103060389	103060642	Distal Intergenic
chr3	103066695	103067636	Distal Intergenic
chr3	103215880	103216850	Distal Intergenic
chr3	103634675	103634876	Distal Intergenic
chr3	103634931	103635243	Distal Intergenic
chr3	103711425	103711648	Intron
chr3	103718926	103719869	Intron
chr3	103720572	103720824	Intron
chr3	103771871	103772275	Distal Intergenic
chr3	104163880	104164103	Distal Intergenic
chr3	104544664	104545639	Distal Intergenic
chr3	104545840	104546471	Distal Intergenic
chr3	104546646	104546860	Distal Intergenic
chr3	104666991	104668197	Distal Intergenic
chr3	104856864	104857094	Distal Intergenic
chr3	104857674	104858089	Distal Intergenic
chr3	105187348	105187763	Intron
chr3	105198661	105198997	Intron
chr3	105199109	105199314	Intron
chr3	105199431	105200494	Intron
chr3	105507922	105508194	Intron
chr3	106803021	106803532	Intron
chr3	107136680	107137246	Distal Intergenic
chr3	107471565	107471943	Intron
chr3	107472024	107472702	Intron
chr3	107578047	107578926	Intron
chr3	107579074	107579958	Intron
chr3	107596105	107596527	Promoter
chr3	107761201	107761406	Downstream

chr3	107816303	107816584	Distal Intergenic
chr3	107888651	107889211	Intron
chr3	107889331	107889839	Intron
chr3	107929199	107929981	Intron
chr3	107931745	107933818	Exon
chr3	107934090	107935513	Intron
chr3	107948233	107948670	Distal Intergenic
chr3	107948800	107949378	Distal Intergenic
chr3	107949658	107949886	Distal Intergenic
chr3	108060120	108060365	Intron
chr3	108204949	108205196	Intron
chr3	108227094	108227576	Intron
chr3	108227648	108228018	Intron
chr3	108246837	108247135	Intron
chr3	108365473	108366048	Exon
chr3	108374712	108375026	3' UTR
chr3	108388598	108390397	Intron
chr3	108565462	108565765	Intron
chr3	108565958	108566696	Exon
chr3	108573821	108574351	Distal Intergenic
chr3	108574585	108575674	Distal Intergenic
chr3	108575844	108576183	Distal Intergenic
chr3	108576297	108576575	Distal Intergenic
chr3	108576938	108578228	Distal Intergenic
chr3	108581528	108582637	Distal Intergenic
chr3	108584074	108584794	Distal Intergenic
chr3	108623281	108623657	Downstream
chr3	108658575	108659469	Intron
chr3	108659670	108659944	Intron
chr3	108659988	108662004	Intron
chr3	108665674	108666434	Intron
chr3	108682931	108683372	Intron
chr3	108683516	108685424	Intron
chr3	108713659	108714334	Intron
chr3	108714451	108714834	Intron
chr3	108761121	108761465	Intron
chr3	108762040	108762422	Intron
chr3	108762597	108762991	Intron
chr3	108854783	108855727	Promoter
chr3	108926257	108926618	Distal Intergenic
chr3	108926806	108927148	Distal Intergenic
chr3	109111842	109113297	Distal Intergenic
chr3	109114346	109114548	Distal Intergenic
chr3	109171410	109171881	Intron

chr3	109271785	109272147	Distal Intergenic
chr3	109272625	109273166	Distal Intergenic
chr3	109424884	109425094	Distal Intergenic
chr3	109425166	109426681	Distal Intergenic
chr3	115590300	115590713	Intron
chr3	115590833	115591292	Intron
chr3	115817779	115818209	Intron
chr3	115821847	115822092	Intron
chr3	115892445	115892644	Intron
chr3	116365304	116365532	Distal Intergenic
chr3	116608798	116608999	Distal Intergenic
chr3	116747188	116747509	Distal Intergenic
chr3	116747616	116748676	Distal Intergenic
chr3	116770221	116770790	Distal Intergenic
chr3	116770915	116771478	Distal Intergenic
chr3	116772956	116773546	Distal Intergenic
chr3	116773617	116773930	Distal Intergenic
chr3	116773981	116774358	Distal Intergenic
chr3	116779370	116779726	Distal Intergenic
chr3	116779807	116780895	Distal Intergenic
chr3	116968419	116968703	Distal Intergenic
chr3	116968787	116968986	Distal Intergenic
chr3	116969093	116969414	Distal Intergenic
chr3	119598299	119598551	Intron
chr3	119658851	119659277	Intron
chr3	119718557	119719421	Intron
chr3	121081954	121082431	Intron
chr3	121129958	121130291	Intron
chr3	121304841	121306032	3' UTR
chr3	121319787	121321616	Intron
chr3	121328844	121329478	Intron
chr3	121352999	121353599	Exon
chr3	121354953	121355524	Exon
chr3	121364650	121365237	Intron
chr3	121377911	121379743	Promoter
chr3	121713356	121713792	Intron
chr3	121765866	121766094	Distal Intergenic
chr3	121785165	121785421	Intron
chr3	121790531	121792168	Intron
chr3	122024698	122025852	Distal Intergenic
chr3	122051849	122052048	Intron
chr3	122066563	122066773	Distal Intergenic
chr3	122093952	122095629	Intron
chr3	122096610	122097217	Intron

chr3	122111372	122111714	Intron
chr3	122111903	122112721	Intron
chr3	122117400	122117618	Intron
chr3	122122288	122122839	Intron
chr3	122123527	122123842	Intron
chr3	122124203	122124678	Intron
chr3	122129264	122129463	Downstream
chr3	122150697	122150928	Intron
chr3	122151227	122152098	Intron
chr3	122160664	122162677	Exon
chr3	122165168	122166413	Intron
chr3	122166519	122166718	Intron
chr3	122174088	122174798	Intron
chr3	122177821	122179439	Promoter
chr3	122184012	122184211	Intron
chr3	122184409	122185638	Intron
chr3	122186070	122186422	5' UTR
chr3	122189358	122189575	Intron
chr3	122190554	122191617	Intron
chr3	122192154	122192393	Intron
chr3	122200464	122200836	Intron
chr3	122200931	122201911	Intron
chr3	122211998	122213467	Intron
chr3	122214698	122214897	Promoter
chr3	122224046	122224608	Intron
chr3	122227466	122227831	Intron
chr3	122227870	122228212	Intron
chr3	122228402	122228972	Intron
chr3	122229184	122229430	Intron
chr3	122229656	122230407	Intron
chr3	122289416	122290368	3' UTR
chr3	122322170	122322372	Intron
chr3	122623799	122624780	Distal Intergenic
chr3	122756393	122756806	Distal Intergenic
chr3	122757014	122757309	Distal Intergenic
chr3	123041770	123041986	Intron
chr3	123134654	123135223	Promoter
chr3	123143976	123144340	Intron
chr3	123144628	123144836	Intron
chr3	123208464	123208797	Distal Intergenic
chr3	123208908	123210373	Downstream
chr3	123210433	123211304	Downstream
chr3	123211554	123211792	Downstream
chr3	123211843	123212209	Downstream

chr3	126087645	126088378	Distal Intergenic
chr3	126363508	126364127	Intron
chr3	126447524	126449034	Intron
chr3	126530736	126530956	Intron
chr3	126532499	126533981	Intron
chr3	126534021	126535083	Intron
chr3	126535230	126535429	Intron
chr3	126571882	126572487	Intron
chr3	126572575	126573536	Intron
chr3	127482750	127482979	Intron
chr3	127483069	127483472	Intron
chr3	127499965	127500789	Exon
chr3	127803346	127803953	Intron
chr3	127807254	127807653	Intron
chr3	127807708	127808318	Intron
chr3	127808423	127809135	Intron
chr3	127809559	127809950	Intron
chr3	127854320	127854538	Intron
chr3	127854639	127855016	Intron
chr3	127855203	127855653	Intron
chr3	129236246	129236511	Exon
chr3	129236550	129236767	Intron
chr3	129236841	129237670	Intron
chr3	129238003	129239336	3' UTR
chr3	129239449	129239761	Distal Intergenic
chr3	130953373	130953995	Intron
chr3	130955462	130956444	Intron
chr3	131530543	131530761	Intron
chr3	131890310	131890780	Intron
chr3	132550259	132550525	Intron
chr3	133263163	133264197	Distal Intergenic
chr3	133287729	133288749	Distal Intergenic
chr3	133289048	133289634	Distal Intergenic
chr3	133289804	133290559	Distal Intergenic
chr3	135102662	135103369	Distal Intergenic
chr3	135255719	135256999	Distal Intergenic
chr3	135257061	135257461	Distal Intergenic
chr3	135257682	135258217	Distal Intergenic
chr3	135304166	135304420	Distal Intergenic
chr3	135314207	135315023	Distal Intergenic
chr3	135333499	135334252	Distal Intergenic
chr3	135334429	135334659	Distal Intergenic
chr3	135335311	135338039	Distal Intergenic
chr3	135352367	135353143	Distal Intergenic

chr3	135353305	135353506	Distal Intergenic
chr3	135353636	135354083	Distal Intergenic
chr3	135375021	135375687	Distal Intergenic
chr3	136094616	136095282	Intron
chr3	136102972	136103271	Intron
chr3	136346715	136347523	Intron
chr3	136351597	136352162	Intron
chr3	136354602	136354853	Intron
chr3	137806924	137807179	Intron
chr3	137807255	137807771	Exon
chr3	141981347	141981621	Distal Intergenic
chr3	142035353	142035636	Intron
chr3	142035908	142036225	Intron
chr3	142036338	142036590	Intron
chr3	142051342	142051572	Exon
chr3	142054780	142055178	Intron
chr3	143790715	143791039	Distal Intergenic
chr3	143866790	143867168	Distal Intergenic
chr3	143973640	143973907	Distal Intergenic
chr3	144094746	144095975	Distal Intergenic
chr3	144325879	144326146	Distal Intergenic
chr3	144326346	144326762	Distal Intergenic
chr3	144656508	144656734	Distal Intergenic
chr3	145314588	145314787	Distal Intergenic
chr3	145579725	145579954	Distal Intergenic
chr3	145580003	145580202	Distal Intergenic
chr3	145587911	145588627	Distal Intergenic
chr3	146163567	146163842	Intron
chr3	151785383	151785723	Distal Intergenic
chr3	151785885	151786128	Distal Intergenic
chr3	157561455	157561974	Distal Intergenic
chr3	157562028	157562311	Distal Intergenic
chr3	159503429	159503839	Intron
chr3	159509836	159511205	Intron
chr3	159511388	159511705	Intron
chr4	3424199	3424951	Exon
chr4	3501762	3502087	Exon
chr4	3684564	3685398	Distal Intergenic
chr4	3760126	3760339	Distal Intergenic
chr4	3761711	3762496	Distal Intergenic
chr4	3793453	3793915	Distal Intergenic
chr4	4561963	4562229	Intron
chr4	4562286	4562698	Intron
chr4	4562764	4563137	Intron

chr4	4720087	4720505	Distal Intergenic
chr4	5451594	5452627	Intron
chr4	5649883	5650103	Intron
chr4	5652226	5652769	Intron
chr4	5695298	5695876	Intron
chr4	5695932	5696547	5' UTR
chr4	5699684	5700139	Intron
chr4	5719516	5720848	Intron
chr4	5763238	5763583	Intron
chr4	6293180	6293866	Exon
chr4	6363820	6364355	Intron
chr4	6364492	6364691	Intron
chr4	6379815	6380878	Exon
chr4	6380934	6381133	Intron
chr4	8426141	8426779	Intron
chr4	8437699	8437909	Intron
chr4	8443522	8444064	Promoter
chr4	8444163	8444625	Intron
chr4	8463434	8463806	Intron
chr4	8840234	8840483	Distal Intergenic
chr4	8931766	8931985	Distal Intergenic
chr4	8994949	8995544	Distal Intergenic
chr4	9031093	9031298	Distal Intergenic
chr4	10192740	10193041	Distal Intergenic
chr4	10193106	10193598	Distal Intergenic
chr4	10870046	10870842	Distal Intergenic
chr4	11021508	11022062	Distal Intergenic
chr4	11054200	11054477	Distal Intergenic
chr4	11054517	11054723	Distal Intergenic
chr4	11082255	11082474	Distal Intergenic
chr4	11082522	11083428	Distal Intergenic
chr4	11249428	11252068	Distal Intergenic
chr4	11312822	11313967	Distal Intergenic
chr4	11314053	11315342	Distal Intergenic
chr4	11346226	11346980	Distal Intergenic
chr4	11350800	11351017	Distal Intergenic
chr4	11354959	11355201	Distal Intergenic
chr4	11381980	11382267	Distal Intergenic
chr4	11463325	11463609	Distal Intergenic
chr4	11463726	11464156	Distal Intergenic
chr4	11618840	11619252	Distal Intergenic
chr4	11917200	11917980	Distal Intergenic
chr4	14783669	14784809	Intron
chr4	14790472	14791273	Intron

chr4	14791396	14792521	Intron
chr4	14825668	14826082	Intron
chr4	14937485	14938116	Intron
chr4	15126313	15127884	Distal Intergenic
chr4	15132120	15133471	Distal Intergenic
chr4	15998082	15998926	Intron
chr4	16095318	16096242	Distal Intergenic
chr4	16126809	16127240	Distal Intergenic
chr4	16311342	16311586	Distal Intergenic
chr4	17491835	17493069	Exon
chr4	17494085	17494338	Intron
chr4	17494436	17494641	Intron
chr4	17495735	17497339	Intron
chr4	19452132	19453032	Distal Intergenic
chr4	19491044	19491913	Distal Intergenic
chr4	20089393	20090523	Distal Intergenic
chr4	20121435	20123304	Distal Intergenic
chr4	20127899	20128122	Distal Intergenic
chr4	20326038	20326804	Intron
chr4	20329959	20330258	Intron
chr4	20567103	20567358	Intron
chr4	20603314	20604406	Intron
chr4	21122733	21122942	Intron
chr4	21420832	21421262	Intron
chr4	21421577	21421830	Intron
chr4	21654871	21655139	Intron
chr4	21774980	21775663	Intron
chr4	21806979	21807214	Intron
chr4	23793454	23793769	3' UTR
chr4	23881339	23881549	Intron
chr4	25742864	25743087	Distal Intergenic
chr4	25743469	25745564	Distal Intergenic
chr4	25810519	25811126	Intron
chr4	25811207	25811568	Intron
chr4	25811763	25811962	Intron
chr4	26142894	26144457	Distal Intergenic
chr4	26185987	26187198	Distal Intergenic
chr4	26187244	26187702	Distal Intergenic
chr4	29010177	29010526	Distal Intergenic
chr4	29508979	29509178	Distal Intergenic
chr4	29509406	29509651	Distal Intergenic
chr4	31855243	31855453	Distal Intergenic
chr4	32050371	32050899	Distal Intergenic
chr4	32051264	32051469	Distal Intergenic

chr4	32266994	32267293	Distal Intergenic
chr4	32337468	32338391	Distal Intergenic
chr4	32391106	32391568	Distal Intergenic
chr4	32392199	32392427	Distal Intergenic
chr4	32406788	32407041	Distal Intergenic
chr4	32492355	32493153	Distal Intergenic
chr4	32496205	32497200	Distal Intergenic
chr4	32744058	32744750	Distal Intergenic
chr4	32887040	32887760	Distal Intergenic
chr4	32918703	32919494	Distal Intergenic
chr4	33117617	33117855	Distal Intergenic
chr4	33335653	33336037	Distal Intergenic
chr4	33348008	33349777	Distal Intergenic
chr4	33360420	33360761	Distal Intergenic
chr4	33368613	33369802	Distal Intergenic
chr4	33424552	33425606	Distal Intergenic
chr4	33461597	33462594	Distal Intergenic
chr4	33506160	33507126	Distal Intergenic
chr4	33533057	33533313	Distal Intergenic
chr4	33533439	33533639	Distal Intergenic
chr4	33925400	33925684	Intron
chr4	34156233	34156526	Distal Intergenic
chr4	34182087	34182605	Distal Intergenic
chr4	34469705	34470234	Distal Intergenic
chr4	34470436	34470958	Distal Intergenic
chr4	34497740	34498068	Distal Intergenic
chr4	34498253	34499443	Distal Intergenic
chr4	34561761	34562154	Distal Intergenic
chr4	34562328	34562744	Distal Intergenic
chr4	34829619	34829872	Distal Intergenic
chr4	34937962	34938880	Distal Intergenic
chr4	34984885	34986480	Distal Intergenic
chr4	35006324	35006607	Distal Intergenic
chr4	35008141	35008736	Distal Intergenic
chr4	35037136	35037587	Distal Intergenic
chr4	35962621	35963058	Intron
chr4	35963147	35963752	Intron
chr4	35982087	35982381	Intron
chr4	36506512	36507146	Distal Intergenic
chr4	38690204	38690599	Exon
chr4	38695135	38695612	Intron
chr4	39035150	39035582	Distal Intergenic
chr4	40089707	40090269	Intron
chr4	40215667	40216154	Intron

chr4	40216202	40216516	Intron
chr4	40226594	40226884	Intron
chr4	40643221	40644688	Distal Intergenic
chr4	40647640	40647890	Distal Intergenic
chr4	40649183	40650869	Distal Intergenic
chr4	40668641	40669385	Distal Intergenic
chr4	40720276	40720652	Distal Intergenic
chr4	40723763	40725160	Distal Intergenic
chr4	40728792	40729666	Distal Intergenic
chr4	40745469	40746734	Distal Intergenic
chr4	40747140	40747360	Distal Intergenic
chr4	40747813	40750586	Distal Intergenic
chr4	40764703	40766009	Intron
chr4	40767565	40769227	Intron
chr4	40791823	40793114	Exon
chr4	40793172	40795087	Intron
chr4	40799006	40799532	Intron
chr4	40799569	40799889	Intron
chr4	40800091	40800577	Intron
chr4	40917667	40917866	Intron
chr4	40971759	40973881	Intron
chr4	40997912	40999373	Intron
chr4	41004189	41005345	Intron
chr4	41010188	41010423	Intron
chr4	41010653	41013689	Intron
chr4	41032951	41033266	Intron
chr4	41036921	41037320	Intron
chr4	41040532	41040743	Intron
chr4	41040838	41041996	Intron
chr4	41048903	41050791	Intron
chr4	41055407	41056018	Intron
chr4	41081815	41082385	Intron
chr4	41082810	41083973	Intron
chr4	41099797	41099996	Intron
chr4	41100152	41100460	Intron
chr4	41143778	41145559	5' UTR
chr4	41213116	41213805	Intron
chr4	41221407	41222080	Downstream
chr4	41231391	41231621	Intron
chr4	41240747	41241568	Intron
chr4	41242541	41242881	Intron
chr4	41260962	41262758	5' UTR
chr4	41296374	41296895	Distal Intergenic
chr4	41296940	41297241	Distal Intergenic

chr4	41299338	41300918	Distal Intergenic
chr4	41684764	41685617	Intron
chr4	41687328	41687585	Intron
chr4	41687810	41688153	Exon
chr4	41717171	41717613	Distal Intergenic
chr4	43012193	43013309	Intron
chr4	43013468	43013906	Intron
chr4	43044102	43044325	Distal Intergenic
chr4	43051716	43053164	Distal Intergenic
chr4	43053317	43053684	Distal Intergenic
chr4	43053808	43054266	Distal Intergenic
chr4	43261741	43262057	Distal Intergenic
chr4	43273067	43273305	Distal Intergenic
chr4	43410799	43412639	Distal Intergenic
chr4	43415077	43415823	Distal Intergenic
chr4	43432529	43433385	Distal Intergenic
chr4	43535115	43536225	Distal Intergenic
chr4	43573899	43575359	Distal Intergenic
chr4	43616940	43618500	Distal Intergenic
chr4	43741108	43741833	Distal Intergenic
chr4	44030774	44031019	Distal Intergenic
chr4	44107098	44107297	Distal Intergenic
chr4	44179882	44180518	Intron
chr4	44181302	44181505	Intron
chr4	44217829	44218784	Intron
chr4	44293989	44295719	Intron
chr4	44768812	44769011	Distal Intergenic
chr4	44769494	44770236	Distal Intergenic
chr4	44852199	44852441	Distal Intergenic
chr4	45023977	45024642	Distal Intergenic
chr4	45230610	45231159	Distal Intergenic
chr4	45354853	45355105	Distal Intergenic
chr4	45467714	45468588	Distal Intergenic
chr4	45469847	45471178	Distal Intergenic
chr4	45481837	45482411	Distal Intergenic
chr4	45485900	45486867	Distal Intergenic
chr4	45488052	45488753	Distal Intergenic
chr4	45496862	45497224	Distal Intergenic
chr4	45501518	45503818	Distal Intergenic
chr4	45504327	45504541	Distal Intergenic
chr4	45504756	45506992	Distal Intergenic
chr4	45507185	45507466	Distal Intergenic
chr4	45507716	45509155	Distal Intergenic
chr4	45516209	45518074	Distal Intergenic

chr4	45518127	45518820	Distal Intergenic
chr4	45519229	45519488	Distal Intergenic
chr4	45523240	45524544	Distal Intergenic
chr4	45526364	45527926	Distal Intergenic
chr4	45541705	45542459	Distal Intergenic
chr4	46082722	46083589	Intron
chr4	46150722	46151595	Distal Intergenic
chr4	46151782	46152196	Distal Intergenic
chr4	46366289	46366560	Intron
chr4	46384164	46384511	Intron
chr4	46402112	46402311	Distal Intergenic
chr4	46402392	46402740	Distal Intergenic
chr4	46402889	46403118	Distal Intergenic
chr4	46403209	46403485	Distal Intergenic
chr4	46463979	46465931	Distal Intergenic
chr4	46465994	46466219	Distal Intergenic
chr4	46498449	46499124	Distal Intergenic
chr4	46536504	46536851	Distal Intergenic
chr4	46646347	46647065	Distal Intergenic
chr4	46652774	46653888	Distal Intergenic
chr4	46655999	46656310	Distal Intergenic
chr4	46656511	46657038	Distal Intergenic
chr4	46661295	46661585	Distal Intergenic
chr4	46661683	46662874	Distal Intergenic
chr4	47263997	47264996	Intron
chr4	47323465	47324123	Intron
chr4	47341457	47342702	Intron
chr4	47343125	47343331	Intron
chr4	47419014	47419366	Intron
chr4	47419569	47420845	Intron
chr4	47487286	47487533	Promoter
chr4	48238661	48242552	Intron
chr4	48243182	48243415	Intron
chr4	48244398	48246440	Intron
chr4	48254648	48258438	Intron
chr4	48266773	48267292	Intron
chr4	48289783	48289995	Distal Intergenic
chr4	48290087	48290742	Distal Intergenic
chr4	48291272	48291790	Distal Intergenic
chr4	48292681	48292883	Distal Intergenic
chr4	48485758	48485957	Promoter
chr4	48552850	48553147	Intron
chr4	48553446	48554293	Exon
chr4	48554388	48555744	Exon

chr4	48556024	48556223	Intron
chr4	48598745	48599183	Intron
chr4	48917338	48919231	Distal Intergenic
chr4	49533521	49533942	Distal Intergenic
chr4	52451842	52452407	Distal Intergenic
chr4	52452473	52452735	Distal Intergenic
chr4	53319900	53320171	Distal Intergenic
chr4	53320233	53320601	Distal Intergenic
chr4	53422280	53422991	Distal Intergenic
chr4	53452604	53453027	Distal Intergenic
chr4	53458064	53459863	3' UTR
chr4	53464565	53465250	3' UTR
chr4	54034191	54034724	Intron
chr4	54034935	54035259	Intron
chr4	54353505	54353705	Intron
chr4	54353794	54354365	Intron
chr4	54368375	54368850	Intron
chr4	54368896	54369113	Intron
chr4	54369194	54369437	Intron
chr4	54369485	54369693	Intron
chr4	54960907	54961274	Intron
chr4	54961610	54961813	Intron
chr4	54962121	54962403	Intron
chr4	54977840	54979481	Intron
chr4	54980249	54981238	Intron
chr4	54984381	54985417	Intron
chr4	54985564	54986306	Intron
chr4	54992253	54992787	Intron
chr4	54992835	54993265	Intron
chr4	55013578	55013793	Intron
chr4	55013904	55015108	Intron
chr4	55218784	55219579	Distal Intergenic
chr4	55264393	55264945	Distal Intergenic
chr4	55266280	55267326	Distal Intergenic
chr4	55362257	55362583	Distal Intergenic
chr4	55362967	55364171	Distal Intergenic
chr4	55364334	55364536	Distal Intergenic
chr4	55365214	55365576	Distal Intergenic
chr4	55366495	55366717	Distal Intergenic
chr4	55366771	55366996	Distal Intergenic
chr4	55368555	55370815	Distal Intergenic
chr4	55544313	55544958	Intron
chr4	55545715	55546720	Intron
chr4	55546792	55547850	Intron

chr4	55547916	55549363	Intron
chr4	55734362	55735420	Distal Intergenic
chr4	55744922	55745268	Distal Intergenic
chr4	55849899	55850135	Distal Intergenic
chr4	56031344	56031649	Distal Intergenic
chr4	56227578	56228662	Intron
chr4	56318382	56319105	Intron
chr4	56386220	56388212	Intron
chr4	56399860	56400494	Intron
chr4	56492225	56492509	Intron
chr4	56493223	56498256	Promoter
chr4	56500483	56500979	Intron
chr4	56501629	56503310	Promoter
chr4	56504651	56505470	Distal Intergenic
chr4	56505534	56505805	Distal Intergenic
chr4	56545526	56547018	Distal Intergenic
chr4	56554664	56554912	Distal Intergenic
chr4	56594054	56597341	Distal Intergenic
chr4	56611496	56611804	Distal Intergenic
chr4	56618625	56619559	Distal Intergenic
chr4	56620812	56626011	Distal Intergenic
chr4	56629910	56631360	Distal Intergenic
chr4	56631579	56631824	Distal Intergenic
chr4	56632219	56635934	Distal Intergenic
chr4	56643846	56644263	Distal Intergenic
chr4	56650386	56650600	Distal Intergenic
chr4	56651201	56653834	Distal Intergenic
chr4	56656074	56657922	Distal Intergenic
chr4	56674039	56674274	Distal Intergenic
chr4	56674476	56675117	Distal Intergenic
chr4	56675155	56675411	Distal Intergenic
chr4	56680777	56682595	Distal Intergenic
chr4	56685543	56687440	Promoter
chr4	56693800	56694550	Intron
chr4	56876587	56877505	Intron
chr4	56949560	56951211	Distal Intergenic
chr4	56958865	56959298	Distal Intergenic
chr4	56959341	56959640	Distal Intergenic
chr4	57165879	57167292	Intron
chr4	57313692	57314174	Intron
chr4	57350368	57351655	Exon
chr4	57365662	57366933	Promoter
chr4	57572751	57573225	Distal Intergenic
chr4	57623855	57624323	Distal Intergenic

chr4	57810515	57812324	Intron
chr4	57814496	57814877	Intron
chr4	57814939	57815692	Intron
chr4	57817315	57818487	Intron
chr4	57967496	57968895	Intron
chr4	57969339	57970323	Intron
chr4	58024003	58024224	Intron
chr4	58024426	58024651	Intron
chr4	58044889	58045359	Intron
chr4	58045481	58046383	Intron
chr4	58052072	58053130	Intron
chr4	58414698	58415205	Distal Intergenic
chr4	58837045	58837334	Distal Intergenic
chr4	58924341	58924768	Distal Intergenic
chr4	58925250	58925978	Distal Intergenic
chr4	59251848	59253557	Distal Intergenic
chr4	59412085	59412483	Distal Intergenic
chr4	59446869	59448291	Distal Intergenic
chr4	59462838	59464481	Distal Intergenic
chr4	59466290	59468438	Distal Intergenic
chr4	59470219	59471408	Distal Intergenic
chr4	59537733	59538055	Distal Intergenic
chr4	59538123	59538421	Distal Intergenic
chr4	59539568	59540030	Distal Intergenic
chr4	59559952	59560409	Distal Intergenic
chr4	59560577	59560966	Distal Intergenic
chr4	59561525	59562108	Distal Intergenic
chr4	59585387	59586034	Distal Intergenic
chr4	59596650	59597172	Distal Intergenic
chr4	59664970	59665959	Distal Intergenic
chr4	59666007	59666276	Distal Intergenic
chr4	59768982	59770996	Distal Intergenic
chr4	59796376	59796641	Distal Intergenic
chr4	59828266	59828673	Distal Intergenic
chr4	59834825	59835146	Distal Intergenic
chr4	61861498	61861974	Distal Intergenic
chr4	61866665	61866883	Distal Intergenic
chr4	61868993	61869268	Distal Intergenic
chr4	61948003	61949996	Distal Intergenic
chr4	61950220	61950419	Distal Intergenic
chr4	62020833	62021111	Distal Intergenic
chr4	62021656	62021925	Distal Intergenic
chr4	62022060	62022504	Distal Intergenic
chr4	62040019	62040227	Distal Intergenic

chr4	62059761	62060705	Distal Intergenic
chr4	62068412	62070622	Intron
chr4	62133199	62134377	Intron
chr4	62181073	62181413	Intron
chr4	62186149	62186348	Intron
chr4	62324546	62325136	Intron
chr4	62391902	62394519	Intron
chr4	62399234	62399912	Intron
chr4	62400448	62400942	Intron
chr4	62984228	62985029	Distal Intergenic
chr4	63004140	63004376	Distal Intergenic
chr4	63004426	63005651	Distal Intergenic
chr4	63205875	63206384	Distal Intergenic
chr4	63413884	63414806	Distal Intergenic
chr4	63457386	63458461	Distal Intergenic
chr4	63619300	63620825	Distal Intergenic
chr4	63638104	63639667	Distal Intergenic
chr4	63670688	63671998	Distal Intergenic
chr4	63672114	63673358	Distal Intergenic
chr4	63673438	63673757	Distal Intergenic
chr4	63674032	63674233	Distal Intergenic
chr4	63674694	63676272	Distal Intergenic
chr4	63737305	63738069	Distal Intergenic
chr4	63738894	63740151	Distal Intergenic
chr4	63744117	63745584	Distal Intergenic
chr4	63748674	63748944	Distal Intergenic
chr4	63755601	63755931	Distal Intergenic
chr4	63758787	63760121	Distal Intergenic
chr4	63785707	63786000	Distal Intergenic
chr4	63793222	63793791	Distal Intergenic
chr4	63836259	63838050	Distal Intergenic
chr4	63915408	63916644	Distal Intergenic
chr4	64464960	64465635	Distal Intergenic
chr4	64558603	64559827	Distal Intergenic
chr4	64559863	64560118	Distal Intergenic
chr4	64560679	64563320	Distal Intergenic
chr4	65356868	65357424	Distal Intergenic
chr4	65360516	65361649	Distal Intergenic
chr4	65778868	65779614	Downstream
chr4	65779706	65780005	Exon
chr4	65787454	65789472	Intron
chr4	65849043	65849297	Intron
chr4	65849486	65850578	Intron
chr4	65998272	65998753	Distal Intergenic

chr4	66488429	66490193	Intron
chr4	70191064	70191478	Distal Intergenic
chr4	70191613	70192239	Distal Intergenic
chr4	70196311	70197990	Distal Intergenic
chr4	71801540	71801773	Intron
chr4	71836848	71838450	Intron
chr4	72317605	72317831	Intron
chr4	72325637	72326105	Intron
chr4	72331108	72331656	Intron
chr4	72331719	72331941	Intron
chr4	72331986	72333293	Exon
chr4	76070439	76070682	Distal Intergenic
chr4	77881283	77881639	Intron
chr4	80562650	80563760	Distal Intergenic
chr4	80563859	80565272	Distal Intergenic
chr4	80596131	80596414	Distal Intergenic
chr4	80617362	80618319	Distal Intergenic
chr4	80817058	80817677	Distal Intergenic
chr4	80817724	80818157	Distal Intergenic
chr4	80898716	80898917	3' UTR
chr4	80954920	80955152	Intron
chr4	80955213	80956203	Intron
chr4	81189828	81190027	Intron
chr4	81572059	81573313	Intron
chr4	81694572	81694884	Intron
chr4	81811809	81812098	Intron
chr4	81812297	81812954	Intron
chr4	82111826	82112146	Intron
chr4	82112280	82113363	Intron
chr4	82116156	82118086	Intron
chr4	82149200	82149399	Distal Intergenic
chr4	82149568	82149874	Distal Intergenic
chr4	82150089	82150301	Distal Intergenic
chr4	82937888	82938120	Distal Intergenic
chr4	82947181	82949461	Distal Intergenic
chr4	82949502	82949990	Distal Intergenic
chr4	82961073	82962096	Distal Intergenic
chr4	82962246	82962445	Distal Intergenic
chr4	82969054	82969363	Distal Intergenic
chr4	82969401	82969724	Distal Intergenic
chr4	82978137	82979760	Distal Intergenic
chr4	83019185	83019424	Distal Intergenic
chr4	83019555	83020919	Distal Intergenic
chr4	83021269	83021591	Distal Intergenic

chr4	83024992	83026907	Distal Intergenic
chr4	83062895	83063510	Distal Intergenic
chr4	83064100	83064631	Distal Intergenic
chr4	84030844	84031835	Promoter
chr4	86280545	86281059	Distal Intergenic
chr4	86314968	86315353	Distal Intergenic
chr4	86315458	86315916	Distal Intergenic
chr4	86316162	86316576	Distal Intergenic
chr4	86317650	86319520	Distal Intergenic
chr4	86319728	86321909	Distal Intergenic
chr4	86322018	86322428	Distal Intergenic
chr4	86322688	86323109	Distal Intergenic
chr4	86325241	86325723	Distal Intergenic
chr4	86356657	86357824	Distal Intergenic
chr4	86357893	86360062	Distal Intergenic
chr4	86502603	86503464	Intron
chr4	87466546	87467758	Intron
chr4	87484232	87484899	Intron
chr4	87485216	87485551	Intron
chr4	87495428	87497032	Intron
chr4	87630298	87630689	Intron
chr4	87630730	87631394	Intron
chr4	87725249	87725454	Intron
chr4	88161089	88161999	Distal Intergenic
chr4	88176208	88176475	Distal Intergenic
chr4	88176616	88177046	Distal Intergenic
chr4	88215664	88216002	Distal Intergenic
chr4	88287671	88288163	Intron
chr4	88368529	88368771	Intron
chr4	88552647	88552899	Distal Intergenic
chr4	88574196	88574862	Intron
chr4	88575421	88584117	5' UTR
chr4	88584668	88590973	3' UTR
chr4	88593670	88594084	Distal Intergenic
chr4	88594236	88594960	Distal Intergenic
chr4	88595150	88597827	Distal Intergenic
chr4	88597928	88598176	Distal Intergenic
chr4	88601453	88603436	Distal Intergenic
chr4	88604517	88604745	Distal Intergenic
chr4	88605156	88606411	Distal Intergenic
chr4	88606462	88606980	Distal Intergenic
chr4	88623632	88624713	Distal Intergenic
chr4	88644411	88645303	Distal Intergenic
chr4	88646518	88648318	Distal Intergenic

chr4	88684092	88686158	Distal Intergenic
chr4	88686200	88686411	Distal Intergenic
chr4	88686543	88686895	Distal Intergenic
chr4	88689939	88690406	Distal Intergenic
chr4	88694222	88694427	Distal Intergenic
chr4	88694821	88696470	Distal Intergenic
chr4	88696574	88705508	Distal Intergenic
chr4	88705620	88705819	Distal Intergenic
chr4	88706527	88708548	Distal Intergenic
chr4	88717702	88717990	Distal Intergenic
chr4	88718234	88718589	Distal Intergenic
chr4	88769361	88774868	Distal Intergenic
chr4	88775049	88775349	Distal Intergenic
chr4	88880435	88881363	Distal Intergenic
chr4	88903429	88903804	Exon
chr4	88941545	88941913	Intron
chr4	88953948	88954334	Intron
chr4	88955652	88955959	Intron
chr4	88956002	88956251	Intron
chr4	89094882	89095641	Intron
chr4	89136536	89137298	Intron
chr4	89137609	89137913	Intron
chr4	89138345	89138860	Intron
chr4	89145426	89146229	Intron
chr4	90119006	90119500	Distal Intergenic
chr4	93684256	93684488	Intron
chr4	93761350	93761616	Intron
chr4	93761765	93761994	Intron
chr4	93914831	93915389	Intron
chr4	94269129	94270130	Intron
chr4	94718453	94718778	Distal Intergenic
chr4	94719528	94720527	Distal Intergenic
chr4	94824980	94825987	Distal Intergenic
chr4	94827656	94828113	Distal Intergenic
chr4	94828158	94829128	Distal Intergenic
chr4	94854488	94855767	Distal Intergenic
chr4	94855809	94856198	Distal Intergenic
chr4	95050542	95050875	Distal Intergenic
chr4	95107215	95107414	Distal Intergenic
chr4	95107609	95107835	Distal Intergenic
chr4	95327843	95328208	Distal Intergenic
chr4	95330382	95330732	Distal Intergenic
chr4	95593052	95593565	Distal Intergenic
chr4	95593803	95594332	Distal Intergenic

chr4	95597855	95599084	Distal Intergenic
chr4	95689898	95690254	Intron
chr4	95690299	95690526	Intron
chr4	95697470	95698577	Intron
chr4	96233514	96234328	Intron
chr4	96234624	96234836	Intron
chr4	96234878	96235115	Intron
chr4	96457886	96458085	Intron
chr4	97497901	97498728	Distal Intergenic
chr4	97498767	97499024	Distal Intergenic
chr4	97499267	97500186	Distal Intergenic
chr4	98062887	98063337	Distal Intergenic
chr4	98074071	98074566	Distal Intergenic
chr4	98102533	98102924	Distal Intergenic
chr4	98121743	98122631	Distal Intergenic
chr4	99354760	99355328	Exon
chr4	99360790	99361490	Intron
chr4	99374209	99374446	Distal Intergenic
chr4	99717822	99718674	Distal Intergenic
chr4	99946019	99947326	Exon
chr4	99958069	99959265	Intron
chr4	99959332	99959531	Intron
chr4	100059434	100059676	Intron
chr4	100297397	100297612	Distal Intergenic
chr4	101204136	101205456	Distal Intergenic
chr4	101434995	101435353	Intron
chr4	102183707	102185097	Intron
chr4	102185144	102186008	Intron
chr4	102186054	102186263	Intron
chr4	102205762	102205979	Intron
chr4	102229433	102229632	Intron
chr4	102242699	102243923	Intron
chr4	102244367	102245114	Intron
chr4	102245300	102245811	Intron
chr4	102245852	102246295	Intron
chr4	102249752	102250662	Intron
chr4	102254518	102254907	Intron
chr4	102559340	102559641	Intron
chr4	102792345	102792759	Intron
chr4	103252559	103252784	Intron
chr4	104590691	104591274	Intron
chr4	104591702	104592367	Intron
chr4	104608742	104608941	Intron
chr4	105035752	105036057	Distal Intergenic

chr4	105076231	105076918	Distal Intergenic
chr4	105091822	105092103	Distal Intergenic
chr4	105092281	105092493	Distal Intergenic
chr4	105524267	105524807	Intron
chr4	105609881	105610239	Distal Intergenic
chr4	105720001	105720200	Distal Intergenic
chr4	105721502	105722280	Distal Intergenic
chr4	105722512	105722946	Distal Intergenic
chr4	105973210	105974899	Distal Intergenic
chr4	105988558	105988757	Distal Intergenic
chr4	105988877	105990709	Distal Intergenic
chr4	106223249	106223524	Distal Intergenic
chr4	106255020	106255726	Distal Intergenic
chr4	106262478	106263326	Distal Intergenic
chr4	106511825	106512188	Intron
chr4	106514181	106514498	Intron
chr4	106515468	106515716	Intron
chr4	106520541	106520989	Intron
chr4	106580988	106582383	Intron
chr4	106582909	106583283	Intron
chr4	106619337	106621493	5' UTR
chr4	106652881	106653946	Intron
chr4	106660268	106660769	Intron
chr4	106850571	106850997	Intron
chr4	107160929	107161725	Intron
chr4	107226256	107226455	Intron
chr4	107537734	107537950	Distal Intergenic
chr4	107552355	107552591	Distal Intergenic
chr4	108252090	108252456	Distal Intergenic
chr4	108452932	108453191	Distal Intergenic
chr4	108453865	108454064	Distal Intergenic
chr4	108454186	108454541	Distal Intergenic
chr4	108455295	108455897	Distal Intergenic
chr4	108505611	108506173	Distal Intergenic
chr4	108507011	108507619	Distal Intergenic
chr4	108520070	108520384	Distal Intergenic
chr4	108790156	108790560	Intron
chr4	108885791	108886146	Distal Intergenic
chr4	108886266	108887087	Distal Intergenic
chr4	108893317	108893616	Distal Intergenic
chr4	109132833	109133049	Distal Intergenic
chr4	109339434	109339994	Distal Intergenic
chr4	109348553	109348802	Distal Intergenic
chr4	109349405	109349775	Distal Intergenic

chr4	109492909	109494005	Intron
chr4	111097439	111098133	Intron
chr4	111106734	111107175	Intron
chr4	111249911	111250177	Distal Intergenic
chr4	111250251	111250518	Distal Intergenic
chr4	114032570	114032810	Intron
chr4	114033008	114033287	Intron
chr4	114033826	114034073	Intron
chr4	114034115	114034372	Intron
chr4	114218301	114218510	Intron
chr4	114250579	114251123	Intron
chr4	114251170	114251436	5' UTR
chr4	114262344	114262721	Promoter
chr4	114273312	114275114	Exon
chr4	114630865	114631280	Intron
chr4	114672846	114673362	Intron
chr4	115747735	115747953	Downstream
chr4	115748029	115748691	Downstream
chr4	115748790	115749632	3' UTR
chr4	116228828	116229436	Distal Intergenic
chr4	116229473	116229685	Distal Intergenic
chr4	116230839	116231039	Distal Intergenic
chr4	116267581	116268530	Distal Intergenic
chr4	116268755	116269087	Distal Intergenic
chr4	116269158	116269502	Distal Intergenic
chr4	116323356	116323968	Distal Intergenic
chr4	116324048	116324285	Distal Intergenic
chr4	116352325	116352536	Distal Intergenic
chr4	116358238	116358445	Distal Intergenic
chr4	116422569	116422802	Distal Intergenic
chr4	116790369	116791583	Distal Intergenic
chr4	116793448	116794162	Distal Intergenic
chr4	116795157	116795412	Distal Intergenic
chr4	116797247	116799068	Distal Intergenic
chr4	116799126	116799705	Distal Intergenic
chr4	116799758	116800026	Distal Intergenic
chr4	116862342	116862729	Distal Intergenic
chr4	117504718	117504917	Distal Intergenic
chr4	117619859	117620098	Distal Intergenic
chr4	117852732	117852931	Distal Intergenic
chr4	117901768	117902194	Distal Intergenic
chr4	117902276	117902594	Distal Intergenic
chr4	117902743	117902942	Distal Intergenic
chr4	117912914	117913299	Distal Intergenic

chr4	117918663	117919377	Distal Intergenic
chr4	117944322	117945560	Distal Intergenic
chr4	117946524	117946830	Distal Intergenic
chr4	117946873	117947444	Distal Intergenic
chr4	117947652	117947971	Distal Intergenic
chr4	117948672	117949343	Distal Intergenic
chr4	117949572	117951204	Distal Intergenic
chr4	118100214	118100605	Distal Intergenic
chr4	118100678	118101557	Distal Intergenic
chr4	118101788	118102006	Distal Intergenic
chr4	118109668	118110275	Distal Intergenic
chr4	118594869	118596210	Distal Intergenic
chr4	118685402	118686158	Distal Intergenic
chr4	118691180	118691824	Distal Intergenic
chr4	118692171	118692654	Distal Intergenic
chr4	118729134	118730943	Distal Intergenic
chr4	118732186	118732433	Distal Intergenic
chr4	118739369	118740289	Distal Intergenic
chr4	118774720	118775725	Distal Intergenic
chr4	118776837	118777113	Distal Intergenic
chr4	118777158	118777556	Distal Intergenic
chr4	118778100	118779513	Distal Intergenic
chr4	118780843	118781169	Distal Intergenic
chr4	118781471	118781719	Distal Intergenic
chr4	118782704	118782903	Distal Intergenic
chr4	118783627	118783826	Distal Intergenic
chr4	118791190	118792086	Distal Intergenic
chr4	118792151	118794679	Distal Intergenic
chr4	118797171	118799242	Distal Intergenic
chr4	118799409	118799713	Distal Intergenic
chr4	118813465	118814131	Distal Intergenic
chr4	118814171	118814401	Distal Intergenic
chr4	118830638	118831244	Distal Intergenic
chr4	119604883	119606448	Promoter
chr4	119608644	119608885	Intron
chr4	119627989	119628188	Intron
chr4	119695161	119696057	Intron
chr4	119820916	119821340	Intron
chr4	119842974	119843560	Intron
chr4	119902372	119902589	Intron
chr4	119928241	119929611	Intron
chr4	119942927	119945502	Promoter
chr4	120145148	120145722	Intron
chr4	120145850	120146082	Intron

chr4	120160113	120161294	Promoter
chr4	120188757	120191605	Promoter
chr4	120200534	120200787	Intron
chr4	120215917	120217925	3' UTR
chr4	120240885	120241465	Intron
chr4	120241665	120241876	Exon
chr4	120242156	120242592	Promoter
chr4	120243522	120245269	Promoter
chr4	120245768	120246906	Distal Intergenic
chr4	120488501	120488711	Intron
chr4	122545398	122546096	Distal Intergenic
chr4	122563452	122563721	Distal Intergenic
chr4	122632408	122633117	Distal Intergenic
chr4	122723103	122723997	Promoter
chr4	123093330	123093576	Intron
chr4	123099344	123099695	Intron
chr4	123099811	123101558	Intron
chr4	123206530	123206760	Intron
chr4	123206922	123207861	Exon
chr4	123207932	123208228	Exon
chr4	123212351	123212613	Intron
chr4	123229214	123229470	Exon
chr4	123232579	123232843	Intron
chr4	123233044	123233419	Intron
chr4	123233497	123233698	Intron
chr4	123241206	123244008	Intron
chr4	123248351	123249396	Exon
chr4	123249768	123251268	Intron
chr4	123261860	123262434	Intron
chr4	123264687	123266232	3' UTR
chr4	123266377	123267425	Intron
chr4	123288226	123288431	Distal Intergenic
chr4	123288553	123289619	Distal Intergenic
chr4	123298714	123299187	Promoter
chr4	123299340	123300281	Promoter
chr4	123300517	123302118	Promoter
chr4	123302308	123303220	Exon
chr4	123312331	123313660	Intron
chr4	123314029	123314998	Exon
chr4	123342267	123342887	Exon
chr4	123343957	123344388	Intron
chr4	123344574	123344785	Intron
chr4	123429127	123429374	Distal Intergenic
chr4	123979974	123980308	Intron

chr4	123980467	123980997	Intron
chr4	124078414	124078932	Intron
chr4	124106344	124106548	Intron
chr4	124248076	124248427	Distal Intergenic
chr4	124352056	124352255	Distal Intergenic
chr4	124353886	124354607	Distal Intergenic
chr4	124378720	124378927	Distal Intergenic
chr4	124379642	124379841	Distal Intergenic
chr4	124380673	124382398	Distal Intergenic
chr4	124385621	124386744	Distal Intergenic
chr4	124475233	124475501	Distal Intergenic
chr4	124792183	124792443	Intron
chr4	124793055	124795451	Intron
chr4	124799272	124799993	Intron
chr4	124800045	124803878	Intron
chr4	125779812	125780501	Distal Intergenic
chr4	125788013	125788492	Distal Intergenic
chr4	125878741	125879378	Distal Intergenic
chr4	125894876	125895144	Distal Intergenic
chr4	125917504	125917864	Distal Intergenic
chr4	125923482	125924597	Distal Intergenic
chr4	125927562	125928598	Distal Intergenic
chr4	125928640	125929125	Distal Intergenic
chr4	125932328	125932810	Distal Intergenic
chr4	125933525	125934049	Distal Intergenic
chr4	125936376	125938700	Distal Intergenic
chr4	126048817	126049432	Distal Intergenic
chr4	127005681	127006569	Distal Intergenic
chr4	127006708	127007687	Distal Intergenic
chr4	127026689	127026973	Distal Intergenic
chr4	127027048	127027553	Distal Intergenic
chr4	127030775	127032628	Distal Intergenic
chr4	128389719	128389918	Distal Intergenic
chr4	128405380	128406093	Distal Intergenic
chr4	128406166	128406600	Distal Intergenic
chr4	128406767	128407977	Distal Intergenic
chr4	128408586	128408861	Distal Intergenic
chr4	128410737	128411292	Distal Intergenic
chr4	128411578	128413143	Distal Intergenic
chr4	128413244	128416149	Distal Intergenic
chr4	128426258	128426457	Distal Intergenic
chr4	128515155	128515883	Distal Intergenic
chr4	128515944	128516151	Distal Intergenic
chr4	128516383	128517653	Distal Intergenic

chr4	128529531	128529991	Distal Intergenic
chr4	128531926	128532661	Distal Intergenic
chr4	128532853	128533810	Distal Intergenic
chr4	128543644	128543862	Distal Intergenic
chr4	128634727	128634949	Intron
chr4	128635022	128635221	Exon
chr4	128710655	128710929	Intron
chr4	128711055	128711410	Intron
chr4	128711885	128713717	Intron
chr4	128713753	128713962	Intron
chr4	128732094	128733285	Exon
chr4	128744881	128745653	Intron
chr4	128758676	128760113	Exon
chr4	128881843	128882042	Intron
chr4	128902967	128903348	Intron
chr4	128903469	128904194	5' UTR
chr4	128908317	128911108	Exon
chr4	128911559	128911959	Intron
chr4	128922307	128922593	Intron
chr4	128924856	128925241	Intron
chr4	128936024	128937191	Intron
chr4	128937507	128938029	Intron
chr4	128938604	128939127	Exon
chr4	128940262	128940471	Intron
chr4	128940845	128941295	Exon
chr4	129093300	129095526	Intron
chr4	129103304	129104211	Intron
chr4	129104258	129104479	Intron
chr4	129317418	129317617	Distal Intergenic
chr4	129419832	129421011	Intron
chr4	129488364	129489150	Distal Intergenic
chr4	129498298	129498505	Distal Intergenic
chr4	129498564	129498966	Distal Intergenic
chr4	129499439	129499728	Distal Intergenic
chr4	129500248	129500627	Distal Intergenic
chr4	129554733	129554932	Distal Intergenic
chr4	129727576	129727809	Distal Intergenic
chr4	129728028	129728557	Distal Intergenic
chr4	129784885	129785084	3' UTR
chr4	129850120	129851766	Intron
chr4	129851814	129852509	Intron
chr4	129852669	129853120	Intron
chr4	129854199	129855155	Intron
chr4	129946008	129946643	Intron

chr4	129950793	129951625	Intron
chr4	129951974	129952391	Intron
chr4	130186334	130186663	Distal Intergenic
chr4	130186700	130187116	Distal Intergenic
chr4	130722240	130722537	Intron
chr4	130751396	130752184	Intron
chr4	131380939	131382340	Distal Intergenic
chr4	131429883	131430609	Distal Intergenic
chr4	131767615	131767973	Distal Intergenic
chr4	131864681	131865279	Distal Intergenic
chr4	131865339	131865560	Distal Intergenic
chr4	131865691	131866343	Distal Intergenic
chr4	131897126	131897367	Distal Intergenic
chr4	131900460	131901136	Distal Intergenic
chr4	131909196	131909864	Distal Intergenic
chr4	131958989	131959622	Distal Intergenic
chr4	131975742	131975967	Distal Intergenic
chr4	132080160	132080983	Distal Intergenic
chr4	132611714	132612031	Distal Intergenic
chr4	132625715	132627067	Distal Intergenic
chr4	132668357	132668746	Distal Intergenic
chr4	132673834	132674126	Distal Intergenic
chr4	132686674	132688344	Distal Intergenic
chr4	132740916	132741220	Distal Intergenic
chr4	132818128	132818935	Distal Intergenic
chr4	132903610	132904647	Distal Intergenic
chr4	132909256	132909863	Distal Intergenic
chr4	132934056	132934387	Distal Intergenic
chr4	132934428	132935089	Distal Intergenic
chr4	132961815	132963950	Distal Intergenic
chr4	132964243	132964681	Distal Intergenic
chr4	133045453	133045814	Distal Intergenic
chr4	133047193	133047454	Distal Intergenic
chr4	133048635	133049821	Distal Intergenic
chr4	133049914	133050490	Distal Intergenic
chr4	133140009	133140298	Distal Intergenic
chr4	133150961	133152244	Distal Intergenic
chr4	133154261	133155575	Distal Intergenic
chr4	133155814	133156595	Distal Intergenic
chr4	133156693	133163819	Distal Intergenic
chr4	133165579	133166061	Distal Intergenic
chr4	133169295	133170468	Distal Intergenic
chr4	133188133	133188783	Distal Intergenic
chr4	133215531	133216244	Distal Intergenic

chr4	133227941	133228243	Distal Intergenic
chr4	133302235	133302973	Distal Intergenic
chr4	133307677	133308133	Distal Intergenic
chr4	133308369	133308578	Distal Intergenic
chr4	133308687	133309466	Distal Intergenic
chr4	133371218	133373173	Distal Intergenic
chr4	133396888	133397500	Distal Intergenic
chr4	133398807	133399006	Distal Intergenic
chr4	133399044	133399738	Distal Intergenic
chr4	133442218	133442417	Distal Intergenic
chr4	133465889	133466639	Distal Intergenic
chr4	133496210	133497659	Distal Intergenic
chr4	133793768	133794027	Distal Intergenic
chr4	134290407	134290861	Distal Intergenic
chr4	134305993	134306192	Distal Intergenic
chr4	134306553	134306959	Distal Intergenic
chr4	134313038	134313474	Distal Intergenic
chr4	134313517	134313728	Distal Intergenic
chr4	134314631	134315043	Distal Intergenic
chr4	134318060	134318751	Distal Intergenic
chr4	134318891	134319346	Distal Intergenic
chr4	134321857	134322971	Distal Intergenic
chr4	134323013	134323424	Distal Intergenic
chr4	134531452	134531719	Distal Intergenic
chr4	134635750	134636195	Distal Intergenic
chr4	134636561	134636780	Distal Intergenic
chr4	134827918	134828292	Distal Intergenic
chr4	134853056	134853306	Distal Intergenic
chr4	134853516	134855493	Distal Intergenic
chr4	134908511	134908958	Distal Intergenic
chr4	134909689	134910130	Distal Intergenic
chr4	135128877	135129097	Distal Intergenic
chr4	135131024	135131349	Distal Intergenic
chr4	135153791	135154376	Distal Intergenic
chr4	135336102	135336950	Distal Intergenic
chr4	135337775	135338085	Distal Intergenic
chr4	135428524	135429765	Distal Intergenic
chr4	135432404	135432725	Distal Intergenic
chr4	135612123	135612612	Distal Intergenic
chr4	135645964	135646275	Distal Intergenic
chr4	135678652	135679647	Distal Intergenic
chr4	135696465	135697314	Distal Intergenic
chr4	135698869	135699286	Distal Intergenic
chr4	135699375	135703219	Distal Intergenic

chr4	135835010	135835602	Distal Intergenic
chr4	135842558	135843463	Distal Intergenic
chr4	135843651	135843897	Distal Intergenic
chr4	135989130	135990440	Distal Intergenic
chr4	135997050	135998186	Distal Intergenic
chr4	136026513	136026723	Distal Intergenic
chr4	136061155	136062523	Distal Intergenic
chr4	136066971	136067858	Distal Intergenic
chr4	136073392	136073591	Distal Intergenic
chr4	136073683	136074102	Distal Intergenic
chr4	136074370	136074615	Distal Intergenic
chr4	136154031	136154230	Distal Intergenic
chr4	136154481	136154746	Distal Intergenic
chr4	136157647	136157894	Distal Intergenic
chr4	136357713	136358099	Distal Intergenic
chr4	136358240	136358462	Distal Intergenic
chr4	136757370	136757783	Distal Intergenic
chr4	136758225	136758452	Distal Intergenic
chr4	136763187	136763386	Distal Intergenic
chr4	136763618	136764091	Distal Intergenic
chr4	136782230	136782435	Distal Intergenic
chr4	136837261	136838184	Distal Intergenic
chr4	136838406	136838659	Distal Intergenic
chr4	136839262	136840191	Distal Intergenic
chr4	136842601	136843290	Distal Intergenic
chr4	136847487	136847757	Distal Intergenic
chr4	136847843	136848446	Distal Intergenic
chr4	136901193	136901764	Distal Intergenic
chr4	136923258	136923582	Distal Intergenic
chr4	136943951	136944568	Distal Intergenic
chr4	136944691	136944907	Distal Intergenic
chr4	137004507	137005265	Distal Intergenic
chr4	137025494	137027642	Distal Intergenic
chr4	137409440	137409816	Distal Intergenic
chr4	137419534	137419790	Distal Intergenic
chr4	137420077	137420302	Distal Intergenic
chr4	137420402	137421202	Distal Intergenic
chr4	137424951	137425763	Distal Intergenic
chr4	137452936	137453139	Distal Intergenic
chr4	137463929	137464665	Distal Intergenic
chr4	137484755	137485124	Distal Intergenic
chr4	137485164	137486372	Distal Intergenic
chr4	137486775	137487752	Distal Intergenic
chr4	137510694	137510936	Distal Intergenic

chr4	137511229	137511457	Distal Intergenic
chr4	137770103	137770305	Distal Intergenic
chr4	137773158	137773661	Distal Intergenic
chr4	137802571	137803013	Distal Intergenic
chr4	137803522	137803765	Distal Intergenic
chr4	137805332	137805607	Distal Intergenic
chr4	137912781	137913008	Distal Intergenic
chr4	138257507	138257712	Distal Intergenic
chr4	138257820	138258166	Distal Intergenic
chr4	138258454	138259211	Distal Intergenic
chr4	138348184	138349068	Distal Intergenic
chr4	138351786	138352011	Distal Intergenic
chr4	138352056	138352767	Distal Intergenic
chr4	138528526	138528788	Distal Intergenic
chr4	138772658	138772959	Distal Intergenic
chr4	138773021	138773258	Distal Intergenic
chr4	138942638	138943265	Distal Intergenic
chr4	139037062	139038252	Intron
chr4	139047977	139048286	Intron
chr4	139056978	139057331	Intron
chr4	139077582	139077917	Intron
chr4	139091510	139092720	3' UTR
chr4	139099898	139100748	Exon
chr4	139132684	139133077	Intron
chr4	139136168	139136402	Intron
chr4	139136595	139136794	Intron
chr4	139137857	139138135	Intron
chr4	139150582	139155422	Exon
chr4	139166055	139166707	Distal Intergenic
chr4	139352910	139353812	Distal Intergenic
chr4	140222150	140222444	Promoter
chr4	140255145	140255426	Exon
chr4	140255594	140257111	Intron
chr4	140364961	140365975	Distal Intergenic
chr4	140418190	140418395	Distal Intergenic
chr4	140694897	140695327	Intron
chr4	140695436	140695635	Intron
chr4	140798391	140798712	Intron
chr4	140798796	140799134	Intron
chr4	140800368	140800655	Intron
chr4	140800830	140801359	Intron
chr4	140815194	140815550	Intron
chr4	140815616	140815826	Intron
chr4	140816049	140816768	Intron

chr4	140829071	140829274	Intron
chr4	140829583	140830027	Intron
chr4	140830139	140832055	Intron
chr4	140832989	140834864	Intron
chr4	140834908	140835132	Intron
chr4	140843303	140843548	Intron
chr4	140858046	140858283	Intron
chr4	140859617	140860107	Intron
chr4	140865596	140866129	Intron
chr4	141010358	141011183	Intron
chr4	141013862	141014389	Intron
chr4	141089255	141089485	Distal Intergenic
chr4	141102080	141102337	Distal Intergenic
chr4	141114879	141115721	Distal Intergenic
chr4	141121134	141121649	Distal Intergenic
chr4	141148381	141148738	Distal Intergenic
chr4	141148800	141149414	Distal Intergenic
chr4	141160130	141160670	Distal Intergenic
chr4	141297167	141298744	Intron
chr4	141549209	141549563	Exon
chr4	141613524	141613812	Intron
chr4	141621495	141622256	Intron
chr4	141626497	141628377	Intron
chr4	141784973	141785426	Downstream
chr4	142710168	142712158	Distal Intergenic
chr4	142868271	142868737	Distal Intergenic
chr4	142878268	142878672	Distal Intergenic
chr4	142878721	142879279	Distal Intergenic
chr4	142884197	142885671	Distal Intergenic
chr4	142885722	142886106	Distal Intergenic
chr4	142886182	142886518	Distal Intergenic
chr4	142886555	142887043	Distal Intergenic
chr4	142895131	142895433	Distal Intergenic
chr4	143994775	143995295	Distal Intergenic
chr4	147279969	147281490	Intron
chr4	147861614	147862351	Intron
chr4	148000288	148000586	Distal Intergenic
chr4	148000669	148001124	Distal Intergenic
chr4	148172004	148172208	Distal Intergenic
chr4	148423545	148424043	Intron
chr4	148544042	148545839	Exon
chr4	148775377	148775593	Intron
chr4	148779850	148780284	Intron
chr4	148780394	148780647	Intron

chr4	148800495	148800694	Intron
chr4	148820294	148820500	Intron
chr4	148859767	148860112	Intron
chr4	148878678	148879088	Intron
chr4	148932500	148932900	Intron
chr4	148945338	148947418	Promoter
chr4	148966744	148967423	Intron
chr4	148967610	148967857	Intron
chr4	148967934	148968273	Exon
chr4	148981636	148982180	Intron
chr4	149270516	149270715	Intron
chr4	149272550	149272782	Intron
chr4	149272896	149273133	Intron
chr4	149434986	149435192	Distal Intergenic
chr4	149611388	149611745	Distal Intergenic
chr4	149612305	149612712	Distal Intergenic
chr4	149612951	149614108	Distal Intergenic
chr4	149656256	149656569	Distal Intergenic
chr4	149833056	149833256	Distal Intergenic
chr4	149918759	149919129	Distal Intergenic
chr4	150234622	150235043	Distal Intergenic
chr4	150244849	150245524	Distal Intergenic
chr4	150245660	150246067	Distal Intergenic
chr4	150246282	150246502	Distal Intergenic
chr4	150347065	150347565	Distal Intergenic
chr4	150347601	150347917	Distal Intergenic
chr4	151393123	151393359	Intron
chr4	151466508	151467388	Intron
chr4	151539422	151539644	Intron
chr4	151559440	151561249	Intron
chr4	151561342	151561584	Intron
chr4	151634268	151634501	Intron
chr4	153464942	153465260	Distal Intergenic
chr4	153592005	153592447	Intron
chr4	153610334	153610623	Distal Intergenic
chr4	154430523	154430736	Intron
chr4	154430821	154432500	Intron
chr4	154432607	154432905	Intron
chr4	154500996	154501381	Intron
chr4	154503573	154503970	Intron
chr4	154504075	154504301	Intron
chr4	154504364	154504954	Exon
chr4	154507344	154507886	Exon
chr4	154508401	154508642	Intron

chr4	154523307	154523708	Exon
chr4	154547400	154547704	Intron
chr4	154547817	154548448	Intron
chr4	154548664	154549233	Exon
chr4	154626517	154626906	3' UTR
chr4	154649443	154649825	Exon
chr4	154650018	154650395	Intron
chr4	154855958	154856427	Distal Intergenic
chr4	154856583	154857018	Distal Intergenic
chr4	154857069	154857290	Distal Intergenic
chr4	154857377	154857577	Distal Intergenic
chr4	154862389	154863029	Distal Intergenic
chr4	155071249	155071545	Distal Intergenic
chr4	155149114	155150294	Distal Intergenic
chr4	155150637	155151666	Distal Intergenic
chr4	155151718	155152028	Distal Intergenic
chr4	155153147	155153383	Downstream
chr4	155153720	155154552	Downstream
chr4	155457347	155457566	3' UTR
chr4	155566540	155567406	Intron
chr4	155567795	155568216	Intron
chr5	3123319	3124113	Distal Intergenic
chr5	3151969	3152695	Distal Intergenic
chr5	3300838	3302321	Distal Intergenic
chr5	3309292	3309727	Distal Intergenic
chr5	3321361	3322855	Distal Intergenic
chr5	3341549	3341748	Distal Intergenic
chr5	3341788	3344138	Distal Intergenic
chr5	3444368	3444699	Intron
chr5	3444879	3445706	Intron
chr5	3494234	3494810	Intron
chr5	3501273	3502080	Intron
chr5	3647037	3647269	Distal Intergenic
chr5	3802978	3804345	Distal Intergenic
chr5	4103151	4103935	Distal Intergenic
chr5	4104030	4105373	Distal Intergenic
chr5	4495865	4497263	Distal Intergenic
chr5	4530424	4531168	Distal Intergenic
chr5	5419550	5419753	Distal Intergenic
chr5	5510714	5511344	Distal Intergenic
chr5	5621295	5621539	Distal Intergenic
chr5	5621580	5623476	Distal Intergenic
chr5	5624336	5626284	Distal Intergenic
chr5	5648656	5649645	Distal Intergenic

chr5	5684273	5687687	Distal Intergenic
chr5	5687992	5688844	Distal Intergenic
chr5	5688903	5689301	Distal Intergenic
chr5	5707658	5708561	Distal Intergenic
chr5	8421706	8422722	Intron
chr5	8797932	8798960	Distal Intergenic
chr5	9068873	9069084	Intron
chr5	9069175	9069768	Intron
chr5	9141961	9142194	Intron
chr5	9160844	9161651	Intron
chr5	9161842	9162355	Intron
chr5	10645825	10646054	Intron
chr5	12107042	12107323	Distal Intergenic
chr5	12107401	12107642	Distal Intergenic
chr5	12242120	12242836	Distal Intergenic
chr5	12243119	12243688	Distal Intergenic
chr5	12404416	12404810	Distal Intergenic
chr5	12910758	12911222	Distal Intergenic
chr5	13415963	13416288	Distal Intergenic
chr5	13745441	13745859	Intron
chr5	13745903	13746209	Intron
chr5	13974328	13975192	Distal Intergenic
chr5	13983726	13984665	Distal Intergenic
chr5	13987036	13987590	Distal Intergenic
chr5	15838421	15838634	Intron
chr5	16864697	16866333	Intron
chr5	17010350	17012173	Distal Intergenic
chr5	17050190	17051156	Distal Intergenic
chr5	17061484	17063724	Distal Intergenic
chr5	17077281	17077954	Distal Intergenic
chr5	17079803	17080071	Distal Intergenic
chr5	17080699	17081083	Distal Intergenic
chr5	17143950	17145486	Intron
chr5	17179753	17179953	Intron
chr5	17194960	17195218	Intron
chr5	17195367	17195841	Intron
chr5	17505037	17506659	Distal Intergenic
chr5	17509010	17509540	Distal Intergenic
chr5	17509604	17510086	Distal Intergenic
chr5	17541080	17542000	Distal Intergenic
chr5	17542137	17542558	Distal Intergenic
chr5	17641747	17644048	Distal Intergenic
chr5	17663984	17664488	Distal Intergenic
chr5	17664535	17665110	Distal Intergenic

chr5	17665412	17665679	Distal Intergenic
chr5	17803399	17803706	Distal Intergenic
chr5	17880795	17881056	Intron
chr5	17881390	17881589	Intron
chr5	20215988	20217419	Intron
chr5	20254326	20256095	5' UTR
chr5	20256170	20256379	Intron
chr5	20405236	20405810	Intron
chr5	20559219	20561751	Intron
chr5	20562226	20562449	Intron
chr5	20642012	20642284	Intron
chr5	20692312	20692674	Intron
chr5	20694110	20695680	Intron
chr5	20723384	20723646	Intron
chr5	20723853	20724237	Intron
chr5	20946598	20947203	Distal Intergenic
chr5	20992938	20993302	Distal Intergenic
chr5	20993541	20993740	Distal Intergenic
chr5	20997849	20999919	Distal Intergenic
chr5	21004084	21004545	Distal Intergenic
chr5	21004591	21004865	Distal Intergenic
chr5	21044766	21045129	Distal Intergenic
chr5	21059428	21060096	Distal Intergenic
chr5	21065549	21066077	Distal Intergenic
chr5	21290449	21290673	Distal Intergenic
chr5	21291098	21291546	Distal Intergenic
chr5	21292010	21293194	Distal Intergenic
chr5	21617336	21617848	Intron
chr5	21618390	21621523	Intron
chr5	21624294	21624502	Intron
chr5	21624555	21624935	Intron
chr5	21624979	21625179	Intron
chr5	21625791	21627293	Intron
chr5	21647072	21648618	Intron
chr5	21660569	21663419	Intron
chr5	21663529	21664377	Intron
chr5	21664435	21664634	Intron
chr5	21664954	21665203	Intron
chr5	21665512	21665813	Intron
chr5	21729620	21731539	Intron
chr5	21833828	21834881	Intron
chr5	22048476	22048690	Intron
chr5	22055605	22056193	Intron
chr5	22892316	22892873	Distal Intergenic

chr5	22892948	22893260	Distal Intergenic
chr5	22923922	22924801	Distal Intergenic
chr5	22929100	22929302	Distal Intergenic
chr5	22937656	22938800	Distal Intergenic
chr5	22938849	22939164	Distal Intergenic
chr5	22939319	22941902	Distal Intergenic
chr5	23011257	23011992	Distal Intergenic
chr5	23087201	23087490	Distal Intergenic
chr5	23121673	23122643	Distal Intergenic
chr5	23157542	23157791	Distal Intergenic
chr5	23157882	23158952	Distal Intergenic
chr5	23217693	23218147	Distal Intergenic
chr5	23349990	23350217	Distal Intergenic
chr5	23357024	23357223	Distal Intergenic
chr5	23383209	23383868	Distal Intergenic
chr5	23383968	23385030	Distal Intergenic
chr5	23393110	23393491	Distal Intergenic
chr5	23399773	23400586	Distal Intergenic
chr5	23404326	23405141	Distal Intergenic
chr5	23408060	23409597	Distal Intergenic
chr5	23419024	23419855	Distal Intergenic
chr5	23419895	23420243	Distal Intergenic
chr5	23498427	23500834	Distal Intergenic
chr5	23535311	23535625	Distal Intergenic
chr5	23535695	23536452	Distal Intergenic
chr5	23542191	23543754	Distal Intergenic
chr5	23543986	23544185	Distal Intergenic
chr5	23544705	23545617	Distal Intergenic
chr5	23545707	23546054	Distal Intergenic
chr5	23552511	23554804	Distal Intergenic
chr5	23569032	23569439	Distal Intergenic
chr5	23569488	23571103	Distal Intergenic
chr5	23617586	23619299	Distal Intergenic
chr5	23729753	23730274	Distal Intergenic
chr5	23767328	23768861	Distal Intergenic
chr5	23772024	23772607	Distal Intergenic
chr5	23775305	23775817	Distal Intergenic
chr5	23895037	23895503	Distal Intergenic
chr5	23895804	23896037	Distal Intergenic
chr5	23896912	23899791	Distal Intergenic
chr5	23910515	23911501	Distal Intergenic
chr5	23929499	23930563	Distal Intergenic
chr5	23950663	23951106	Distal Intergenic
chr5	23971330	23971532	Intron

chr5	23977822	23978396	Exon
chr5	23978863	23982352	Exon
chr5	24190862	24191555	Distal Intergenic
chr5	24191608	24193023	Distal Intergenic
chr5	24195056	24196296	Distal Intergenic
chr5	24257008	24258233	Distal Intergenic
chr5	24282432	24283671	Distal Intergenic
chr5	24326361	24326595	Distal Intergenic
chr5	24326658	24328307	Distal Intergenic
chr5	24331605	24332018	Distal Intergenic
chr5	24343035	24344439	Distal Intergenic
chr5	24346042	24346377	Distal Intergenic
chr5	24346997	24348396	Distal Intergenic
chr5	25218889	25219160	Distal Intergenic
chr5	25253915	25254124	Distal Intergenic
chr5	25254243	25254488	Distal Intergenic
chr5	25254904	25255294	Distal Intergenic
chr5	25255350	25256428	Distal Intergenic
chr5	25256482	25258213	Distal Intergenic
chr5	26492318	26492627	Distal Intergenic
chr5	26495876	26498252	Distal Intergenic
chr5	28057211	28059404	Distal Intergenic
chr5	28413299	28413577	Distal Intergenic
chr5	28643224	28643662	Distal Intergenic
chr5	28643958	28644921	Distal Intergenic
chr5	28678294	28678763	Distal Intergenic
chr5	28716725	28717005	Distal Intergenic
chr5	28723847	28724121	Distal Intergenic
chr5	28955879	28958509	Distal Intergenic
chr5	28959416	28959706	Distal Intergenic
chr5	29705016	29705285	Distal Intergenic
chr5	29761281	29761580	Distal Intergenic
chr5	30024652	30027316	Distal Intergenic
chr5	30029097	30030639	Distal Intergenic
chr5	30032807	30033618	Distal Intergenic
chr5	30033698	30036791	Distal Intergenic
chr5	30036855	30038006	Distal Intergenic
chr5	30061810	30063894	Distal Intergenic
chr5	30070469	30070675	Distal Intergenic
chr5	30137926	30138130	Distal Intergenic
chr5	30169273	30169893	Distal Intergenic
chr5	30171364	30172372	Distal Intergenic
chr5	30399542	30399876	Distal Intergenic
chr5	30481001	30481788	Distal Intergenic

chr5	30559339	30559978	Distal Intergenic
chr5	30566683	30569635	Distal Intergenic
chr5	30572819	30573029	Distal Intergenic
chr5	30605233	30606092	Distal Intergenic
chr5	30615433	30615824	Distal Intergenic
chr5	30950828	30951285	Distal Intergenic
chr5	30951458	30952100	Distal Intergenic
chr5	30952147	30952346	Distal Intergenic
chr5	30953161	30954896	Distal Intergenic
chr5	30955055	30955928	Distal Intergenic
chr5	30957541	30958913	Distal Intergenic
chr5	30968966	30969511	Distal Intergenic
chr5	31152011	31153803	Distal Intergenic
chr5	31166116	31166708	Distal Intergenic
chr5	31191732	31191933	Distal Intergenic
chr5	31209592	31210117	Intron
chr5	31357229	31357440	Distal Intergenic
chr5	31456324	31456621	Intron
chr5	31456989	31457216	Intron
chr5	31481537	31483049	Intron
chr5	31495953	31496201	Intron
chr5	31498986	31499263	Intron
chr5	31503620	31505180	Exon
chr5	31521999	31522617	Intron
chr5	31522765	31523001	Intron
chr5	31523075	31523696	Intron
chr5	31554255	31554978	3' UTR
chr5	31555104	31555332	3' UTR
chr5	31556200	31557614	Distal Intergenic
chr5	31828928	31829371	Intron
chr5	31866162	31867976	Intron
chr5	31870437	31871010	Intron
chr5	31878298	31879066	Intron
chr5	31879222	31879787	Intron
chr5	31898674	31902002	Intron
chr5	31902245	31905438	Intron
chr5	31907335	31909219	Intron
chr5	31916029	31916941	Intron
chr5	31917193	31918687	Intron
chr5	31922330	31923262	Intron
chr5	31927614	31928113	Intron
chr5	31931134	31931555	Intron
chr5	31931723	31932225	Intron
chr5	31932328	31933966	Intron

chr5	31935810	31940087	Promoter
chr5	31995298	31995531	Intron
chr5	31995628	31995917	Exon
chr5	31995997	31996196	Intron
chr5	32000032	32000252	Exon
chr5	32000429	32001880	Intron
chr5	32002104	32002333	Intron
chr5	32002553	32002802	Intron
chr5	32052811	32053276	Exon
chr5	32053352	32054012	Exon
chr5	32054057	32054324	Intron
chr5	32057258	32058062	Exon
chr5	32060461	32061728	Exon
chr5	32184764	32185154	Distal Intergenic
chr5	32207619	32207888	Distal Intergenic
chr5	32238085	32238418	Intron
chr5	32243834	32245562	Intron
chr5	32285720	32287792	Intron
chr5	32337569	32338277	Distal Intergenic
chr5	32338345	32338605	Distal Intergenic
chr5	32423034	32424913	Intron
chr5	32436173	32437212	Intron
chr5	32438241	32438819	Intron
chr5	32439057	32441644	Intron
chr5	32441706	32441905	Intron
chr5	32448692	32448962	Distal Intergenic
chr5	32479492	32480055	Distal Intergenic
chr5	32494768	32495174	Distal Intergenic
chr5	32499858	32500550	Distal Intergenic
chr5	32501066	32502133	Distal Intergenic
chr5	32511727	32512043	Distal Intergenic
chr5	32616892	32617125	Distal Intergenic
chr5	32617285	32618008	Distal Intergenic
chr5	32637835	32641752	Distal Intergenic
chr5	32645025	32645478	Distal Intergenic
chr5	32716195	32716488	Intron
chr5	32716857	32717106	Intron
chr5	32727807	32728404	Intron
chr5	32728536	32728735	Intron
chr5	32760932	32761198	Intron
chr5	32761495	32763324	Intron
chr5	32765678	32765882	Intron
chr5	32774549	32775659	Exon
chr5	32802593	32803209	Distal Intergenic

chr5	32912870	32913074	Distal Intergenic
chr5	32913767	32913969	Distal Intergenic
chr5	32914024	32914241	Distal Intergenic
chr5	33196709	33197148	Distal Intergenic
chr5	33318954	33320039	Distal Intergenic
chr5	33345367	33346483	Distal Intergenic
chr5	33362489	33362707	Distal Intergenic
chr5	33558730	33558997	Intron
chr5	33562358	33563298	Intron
chr5	33616769	33617025	Intron
chr5	33678649	33680274	Intron
chr5	33684054	33684440	Exon
chr5	33684498	33686966	Intron
chr5	33693926	33694747	Intron
chr5	33694791	33696054	Intron
chr5	33721331	33721624	Intron
chr5	33971451	33971862	Intron
chr5	33999943	34000287	Intron
chr5	34000504	34001077	Intron
chr5	34125021	34125354	Distal Intergenic
chr5	34161892	34162393	Distal Intergenic
chr5	34261998	34262813	Distal Intergenic
chr5	34278684	34278916	Distal Intergenic
chr5	34279128	34279327	Distal Intergenic
chr5	34298259	34298975	Distal Intergenic
chr5	34352812	34353036	Distal Intergenic
chr5	34353073	34353576	Distal Intergenic
chr5	34419835	34420545	Distal Intergenic
chr5	34423930	34426854	Distal Intergenic
chr5	34430164	34431064	Distal Intergenic
chr5	34511829	34512100	Distal Intergenic
chr5	34678109	34680120	Intron
chr5	34702532	34702858	Intron
chr5	34702913	34703132	Intron
chr5	34703234	34703433	Intron
chr5	34772965	34773183	Intron
chr5	34924787	34925074	Exon
chr5	34951346	34954434	Exon
chr5	34965950	34967016	Distal Intergenic
chr5	34979057	34980148	Distal Intergenic
chr5	35005972	35006256	Intron
chr5	35331184	35333372	Distal Intergenic
chr5	35340062	35343645	Distal Intergenic
chr5	35343828	35344155	Distal Intergenic

chr5	35346961	35347173	Distal Intergenic
chr5	35363403	35363793	Distal Intergenic
chr5	35375090	35377717	Distal Intergenic
chr5	35377910	35381285	Distal Intergenic
chr5	35917469	35918304	Intron
chr5	35936473	35937131	Intron
chr5	35957993	35958542	Intron
chr5	36004555	36005329	Distal Intergenic
chr5	36045140	36046788	Intron
chr5	36059973	36060799	Intron
chr5	36082443	36082751	Distal Intergenic
chr5	36097966	36098275	Distal Intergenic
chr5	36236415	36237009	Intron
chr5	36250021	36252046	Exon
chr5	36266108	36266737	Intron
chr5	36266832	36267094	Intron
chr5	36282698	36287264	Intron
chr5	36289692	36291565	Intron
chr5	36297133	36297892	Intron
chr5	36315967	36316842	Distal Intergenic
chr5	36545181	36546728	Distal Intergenic
chr5	36709492	36709732	Distal Intergenic
chr5	36709939	36710277	Distal Intergenic
chr5	36722315	36723876	Distal Intergenic
chr5	36724664	36726584	Distal Intergenic
chr5	36732467	36733138	Distal Intergenic
chr5	36757230	36757976	Distal Intergenic
chr5	36764514	36766177	Distal Intergenic
chr5	36803004	36804706	Distal Intergenic
chr5	36961582	36961785	Promoter
chr5	37065608	37067391	3' UTR
chr5	37067824	37068135	Distal Intergenic
chr5	37103982	37104885	Downstream
chr5	37105646	37106737	3' UTR
chr5	37113677	37114130	Intron
chr5	37114252	37114529	Intron
chr5	37187211	37187464	Intron
chr5	37230166	37232711	Exon
chr5	37236581	37238099	Intron
chr5	37239487	37241943	Exon
chr5	37243186	37244635	Exon
chr5	37296290	37296750	Intron
chr5	37311200	37313368	Intron
chr5	38871295	38871704	Intron

chr5	38944934	38945900	Exon
chr5	38951752	38952106	Intron
chr5	38953114	38953313	Exon
chr5	39824353	39825245	Distal Intergenic
chr5	40153179	40153775	Distal Intergenic
chr5	42542073	42542680	Intron
chr5	42547116	42547339	Promoter
chr5	43371966	43373246	Distal Intergenic
chr5	43378641	43379701	Intron
chr5	43414297	43414519	Distal Intergenic
chr5	43414562	43415410	Distal Intergenic
chr5	43415461	43416007	Distal Intergenic
chr5	43436824	43437793	Distal Intergenic
chr5	43439695	43440245	Distal Intergenic
chr5	43463243	43464766	Intron
chr5	43472041	43473790	Intron
chr5	43623668	43624011	Intron
chr5	43624459	43624999	Intron
chr5	43746496	43746961	Distal Intergenic
chr5	43749477	43749705	Distal Intergenic
chr5	43766770	43767015	Distal Intergenic
chr5	44083338	44084995	Distal Intergenic
chr5	44157502	44157783	Distal Intergenic
chr5	44157903	44158505	Distal Intergenic
chr5	44171540	44172769	Distal Intergenic
chr5	44173032	44173301	Distal Intergenic
chr5	44178817	44179717	Distal Intergenic
chr5	44179773	44180071	Distal Intergenic
chr5	44188505	44189303	Distal Intergenic
chr5	44190558	44191572	Distal Intergenic
chr5	44191701	44192002	Distal Intergenic
chr5	44192602	44193634	Distal Intergenic
chr5	44206580	44208148	Distal Intergenic
chr5	44208422	44208731	Distal Intergenic
chr5	44211567	44211852	Distal Intergenic
chr5	44211972	44212504	Distal Intergenic
chr5	44336625	44336951	Intron
chr5	44338564	44341201	Intron
chr5	44365291	44366820	Intron
chr5	44376130	44377929	Intron
chr5	44549262	44549476	Distal Intergenic
chr5	44642863	44643137	Distal Intergenic
chr5	44643176	44643390	Distal Intergenic
chr5	45686643	45686978	Intron

chr5	45868863	45869619	Distal Intergenic
chr5	45911183	45911444	Distal Intergenic
chr5	46248509	46248708	Distal Intergenic
chr5	47787301	47789258	Distal Intergenic
chr5	47893966	47894409	Distal Intergenic
chr5	48080197	48080455	Distal Intergenic
chr5	48504968	48505218	Distal Intergenic
chr5	48505259	48505549	Distal Intergenic
chr5	48955113	48955455	Distal Intergenic
chr5	49728042	49728260	Intron
chr5	49728555	49728763	Intron
chr5	51432664	51433513	Distal Intergenic
chr5	51437224	51437476	Distal Intergenic
chr5	52529202	52530251	Distal Intergenic
chr5	52530310	52530637	Distal Intergenic
chr5	52580579	52581287	Distal Intergenic
chr5	52582327	52582767	Distal Intergenic
chr5	52623099	52623332	Distal Intergenic
chr5	52623373	52623572	Distal Intergenic
chr5	53052520	53053302	Distal Intergenic
chr5	53174399	53174605	Exon
chr5	53177729	53178001	Downstream
chr5	53178075	53178383	Downstream
chr5	53329559	53330071	Intron
chr5	53331495	53331760	Intron
chr5	53432959	53434561	Intron
chr5	53493213	53494354	Intron
chr5	53494706	53494924	Intron
chr5	53494977	53495945	Intron
chr5	53528610	53530625	Intron
chr5	53562687	53563613	Intron
chr5	53587792	53588127	Intron
chr5	53625970	53627071	Distal Intergenic
chr5	53627351	53627762	Distal Intergenic
chr5	53629779	53631867	Distal Intergenic
chr5	53658140	53658799	Distal Intergenic
chr5	53658915	53659181	Distal Intergenic
chr5	53659382	53659693	Distal Intergenic
chr5	54312253	54312663	Distal Intergenic
chr5	54340079	54340602	Distal Intergenic
chr5	54344294	54345825	Distal Intergenic
chr5	54353055	54353355	Distal Intergenic
chr5	54362178	54363170	Distal Intergenic
chr5	58107925	58108140	Intron

chr5	58109565	58109909	Intron
chr5	58113722	58114033	Intron
chr5	58146893	58147457	3' UTR
chr5	58206911	58208234	Distal Intergenic
chr5	58310170	58310399	Intron
chr5	58310486	58310753	Intron
chr5	58311064	58311263	Intron
chr5	58331730	58332139	Intron
chr5	58333808	58335747	Promoter
chr5	58365214	58365964	Intron
chr5	58366932	58368681	Intron
chr5	58430741	58432008	Intron
chr5	58497558	58497922	Intron
chr5	58498256	58498710	Intron
chr5	58991032	58991335	Intron
chr5	63156434	63156727	Distal Intergenic
chr5	63156807	63157060	Distal Intergenic
chr5	63157600	63158078	Distal Intergenic
chr5	63193666	63194582	Distal Intergenic
chr5	63203926	63204127	Distal Intergenic
chr5	64345133	64345354	Distal Intergenic
chr5	64349086	64349715	Distal Intergenic
chr5	64355753	64356625	Distal Intergenic
chr5	64358601	64359511	Distal Intergenic
chr5	64397832	64398613	Distal Intergenic
chr5	64436094	64436905	Distal Intergenic
chr5	64436985	64437243	Distal Intergenic
chr5	64439090	64439289	Distal Intergenic
chr5	64503245	64503660	Intron
chr5	64504013	64504215	Intron
chr5	64537610	64538409	Exon
chr5	64552093	64552416	Intron
chr5	64565393	64566309	Intron
chr5	64625779	64627157	Intron
chr5	64630419	64631530	Intron
chr5	64897157	64897377	Intron
chr5	64912743	64913077	Intron
chr5	64970673	64971666	Intron
chr5	65094956	65095202	Intron
chr5	65095549	65096147	Intron
chr5	65195480	65198563	Distal Intergenic
chr5	65200773	65200977	Distal Intergenic
chr5	65201660	65204502	Distal Intergenic
chr5	65384030	65384256	Distal Intergenic

chr5	65781054	65781260	Distal Intergenic
chr5	65781321	65781776	Distal Intergenic
chr5	65782112	65782672	Distal Intergenic
chr5	65819838	65820341	Distal Intergenic
chr5	65845568	65845767	Distal Intergenic
chr5	65845872	65846727	Distal Intergenic
chr5	65848119	65850082	Distal Intergenic
chr5	65863613	65863857	Distal Intergenic
chr5	65927646	65928182	Intron
chr5	65928559	65929818	Intron
chr5	66037836	66038193	Intron
chr5	66088645	66088965	Intron
chr5	66154271	66154673	Intron
chr5	66156010	66156236	Intron
chr5	66425590	66425843	Intron
chr5	66425981	66426340	Exon
chr5	66436988	66437920	Intron
chr5	66541090	66543488	Distal Intergenic
chr5	66556871	66557304	Distal Intergenic
chr5	66666258	66666457	Distal Intergenic
chr5	66888529	66888992	Distal Intergenic
chr5	66889193	66889423	Distal Intergenic
chr5	66909675	66909885	Distal Intergenic
chr5	66933567	66935871	Distal Intergenic
chr5	66941460	66942575	Distal Intergenic
chr5	66948905	66952012	Distal Intergenic
chr5	66970287	66970894	Distal Intergenic
chr5	66972754	66972953	Distal Intergenic
chr5	66973106	66973359	Distal Intergenic
chr5	66977039	66978032	Distal Intergenic
chr5	66979402	66981566	Distal Intergenic
chr5	66998229	67000317	Distal Intergenic
chr5	67652015	67652542	Distal Intergenic
chr5	67799195	67799575	Distal Intergenic
chr5	67799618	67800394	Distal Intergenic
chr5	67801733	67802171	Distal Intergenic
chr5	68222543	68222990	Distal Intergenic
chr5	68235337	68236034	Distal Intergenic
chr5	68238804	68239037	Distal Intergenic
chr5	68257479	68258080	Distal Intergenic
chr5	68258123	68258742	Distal Intergenic
chr5	72558788	72559160	Distal Intergenic
chr5	72591498	72594471	Distal Intergenic
chr5	72594554	72595256	Distal Intergenic

chr5	72595530	72595812	Distal Intergenic
chr5	72602467	72604029	Distal Intergenic
chr5	72640771	72642094	Distal Intergenic
chr5	72645928	72646342	Distal Intergenic
chr5	73246076	73247362	Distal Intergenic
chr5	73471271	73471484	Exon
chr5	73495030	73495233	Distal Intergenic
chr5	73624362	73625014	Distal Intergenic
chr5	73625204	73625469	Distal Intergenic
chr5	73733392	73736174	Distal Intergenic
chr5	73760129	73760331	Distal Intergenic
chr5	73764002	73764461	Distal Intergenic
chr5	73894022	73894332	Distal Intergenic
chr5	73955022	73955639	Intron
chr5	73956792	73958828	Intron
chr5	73969496	73970866	Intron
chr5	74488782	74488984	Intron
chr5	74489127	74490646	Exon
chr5	74490927	74491488	Exon
chr5	74493136	74493565	Intron
chr5	74493611	74495132	Intron
chr5	74497673	74498226	Intron
chr5	74501561	74501844	Intron
chr5	74568198	74568677	Distal Intergenic
chr5	74681113	74681478	Intron
chr5	74681601	74681800	Exon
chr5	74888496	74888991	Intron
chr5	74931583	74932298	Exon
chr5	75148797	75149790	Distal Intergenic
chr5	75292750	75294262	Distal Intergenic
chr5	75331219	75331423	Distal Intergenic
chr5	75331516	75332989	Distal Intergenic
chr5	75334254	75334515	Distal Intergenic
chr5	75429259	75429556	Intron
chr5	75439425	75439757	Intron
chr5	75812879	75813131	Intron
chr5	75817526	75817860	Intron
chr5	76613480	76614071	Intron
chr5	76733901	76734532	Exon
chr5	76774501	76775045	Intron
chr5	76775091	76776939	Intron
chr5	77334227	77334430	Intron
chr5	77335458	77335807	Intron
chr5	77391164	77391389	Intron

chr5	77391492	77391769	Intron
chr5	77403178	77403688	Intron
chr5	77403750	77404027	Intron
chr5	77404391	77404618	Intron
chr5	77501519	77501785	Intron
chr5	77583769	77583984	Intron
chr5	77584174	77584704	Intron
chr5	77629401	77629613	Distal Intergenic
chr5	77629758	77630342	Distal Intergenic
chr5	77677174	77677571	Intron
chr5	77681454	77681685	Intron
chr5	77695549	77696479	Intron
chr5	77793581	77793825	Intron
chr5	77794043	77794347	Intron
chr5	77794669	77797751	Intron
chr5	86493659	86493949	Intron
chr5	86494147	86494615	Intron
chr5	86669931	86670255	Exon
chr5	86674032	86674281	Exon
chr5	86676478	86676772	Intron
chr5	86677190	86677841	Intron
chr5	86969811	86970301	Distal Intergenic
chr5	87059712	87060110	Distal Intergenic
chr5	87105347	87105841	Distal Intergenic
chr5	87124114	87124647	Distal Intergenic
chr5	87151661	87152759	Distal Intergenic
chr5	87155742	87157921	Distal Intergenic
chr5	87158572	87160682	Distal Intergenic
chr5	87170191	87170631	Distal Intergenic
chr5	87171118	87171470	Distal Intergenic
chr5	87171698	87172488	Distal Intergenic
chr5	87233562	87234049	Distal Intergenic
chr5	87234212	87234464	Distal Intergenic
chr5	87285437	87286162	Distal Intergenic
chr5	88982821	88983179	Distal Intergenic
chr5	89116952	89118258	Distal Intergenic
chr5	89149644	89150062	Distal Intergenic
chr5	89412231	89412686	Distal Intergenic
chr5	89899084	89899986	Intron
chr5	89902088	89902296	Intron
chr5	89902338	89902576	Intron
chr5	90177453	90178054	Intron
chr5	90653118	90653530	Distal Intergenic
chr5	90653651	90653947	Distal Intergenic

chr5	90793710	90794751	Distal Intergenic
chr5	90794837	90796311	Distal Intergenic
chr5	91023954	91024468	Distal Intergenic
chr5	91040219	91042117	Distal Intergenic
chr5	91063733	91063932	Distal Intergenic
chr5	91088681	91090563	Distal Intergenic
chr5	91116709	91119561	Distal Intergenic
chr5	91120165	91120364	Distal Intergenic
chr5	91120428	91120769	Distal Intergenic
chr5	91120990	91121195	Distal Intergenic
chr5	91122680	91124225	Distal Intergenic
chr5	91127693	91129417	Distal Intergenic
chr5	91134992	91135357	Distal Intergenic
chr5	91135399	91135780	Distal Intergenic
chr5	91187828	91188122	Distal Intergenic
chr5	91188267	91188479	Distal Intergenic
chr5	91188601	91189327	Distal Intergenic
chr5	91189515	91191808	Distal Intergenic
chr5	91191967	91193844	Distal Intergenic
chr5	91206396	91206783	Distal Intergenic
chr5	91206837	91207829	Distal Intergenic
chr5	91208084	91208524	Distal Intergenic
chr5	91304442	91306227	Distal Intergenic
chr5	91308253	91310056	Distal Intergenic
chr5	91310687	91311163	Distal Intergenic
chr5	91320241	91320445	Distal Intergenic
chr5	91320540	91321126	Distal Intergenic
chr5	91328310	91330271	Distal Intergenic
chr5	91360425	91361201	Distal Intergenic
chr5	91400937	91401977	Distal Intergenic
chr5	91406610	91407478	Distal Intergenic
chr5	91416339	91417024	Distal Intergenic
chr5	91444002	91444260	Distal Intergenic
chr5	91448029	91448420	Distal Intergenic
chr5	91456312	91456631	Distal Intergenic
chr5	91457077	91457276	Distal Intergenic
chr5	91463929	91467395	Distal Intergenic
chr5	91502139	91502503	Intron
chr5	91502618	91504095	Intron
chr5	91505144	91505367	Intron
chr5	91510280	91510625	Intron
chr5	91530652	91531012	Intron
chr5	91531074	91531480	Intron
chr5	91533152	91533352	Intron

chr5	91533440	91533929	Intron
chr5	91534037	91534651	Intron
chr5	91534855	91535183	Intron
chr5	91538361	91540951	Intron
chr5	91543336	91543642	Intron
chr5	91670607	91671490	Intron
chr5	91726077	91726305	Intron
chr5	92038074	92038306	Distal Intergenic
chr5	92038379	92039698	Distal Intergenic
chr5	92041771	92042543	Distal Intergenic
chr5	92046262	92046919	Distal Intergenic
chr5	92100299	92100554	Distal Intergenic
chr5	92137270	92138004	Distal Intergenic
chr5	92269712	92270579	Distal Intergenic
chr5	92389801	92392081	Distal Intergenic
chr5	92415766	92417023	Distal Intergenic
chr5	92472245	92472444	Distal Intergenic
chr5	92647704	92650899	Distal Intergenic
chr5	92658438	92659971	Distal Intergenic
chr5	92711913	92712892	Distal Intergenic
chr5	92897709	92899662	Promoter
chr5	92899746	92899994	Promoter
chr5	92900102	92901541	Promoter
chr5	92918263	92919638	Promoter
chr5	92933839	92934158	Distal Intergenic
chr5	93253579	93253938	Intron
chr5	93278776	93279169	Intron
chr5	93390587	93391445	Intron
chr5	93395888	93397190	Intron
chr5	93398283	93398600	Intron
chr5	93398825	93399025	Intron
chr5	93399173	93399720	Intron
chr5	93522236	93522602	Intron
chr5	93532421	93534230	Intron
chr5	93534555	93534851	Intron
chr5	93555100	93555390	Intron
chr5	93556994	93557292	Intron
chr5	93558794	93559200	Intron
chr5	93564303	93564502	Intron
chr5	96241488	96242964	Intron
chr5	96589456	96590109	Distal Intergenic
chr5	96590733	96591015	Distal Intergenic
chr5	96591117	96591516	Distal Intergenic
chr5	96650296	96650620	Distal Intergenic

chr5	96650686	96651073	Distal Intergenic
chr5	96651128	96651355	Distal Intergenic
chr5	96700253	96700480	Distal Intergenic
chr5	97007056	97008246	Distal Intergenic
chr5	97048681	97050593	Distal Intergenic
chr5	97051362	97051885	Distal Intergenic
chr5	97109757	97110050	Distal Intergenic
chr5	97222287	97224491	Distal Intergenic
chr5	97229547	97230004	Distal Intergenic
chr5	97230141	97230757	Distal Intergenic
chr5	97230935	97231977	Distal Intergenic
chr5	97232056	97233130	Distal Intergenic
chr5	97233305	97234609	Distal Intergenic
chr5	97326651	97328868	Distal Intergenic
chr5	97328969	97329347	Distal Intergenic
chr5	97393484	97393741	Distal Intergenic
chr5	98712866	98713147	Distal Intergenic
chr5	98713322	98713902	Distal Intergenic
chr5	98719529	98720735	Distal Intergenic
chr5	98721731	98723071	Distal Intergenic
chr5	98855739	98855964	Distal Intergenic
chr5	98858446	98859830	Exon
chr5	99273413	99274090	Distal Intergenic
chr5	99311908	99313443	Distal Intergenic
chr5	99319253	99320014	Distal Intergenic
chr5	99326954	99327647	Distal Intergenic
chr5	99372640	99372915	Distal Intergenic
chr5	99372983	99373483	Distal Intergenic
chr5	99500988	99502549	Distal Intergenic
chr5	99502598	99502801	Distal Intergenic
chr5	99502871	99503154	Distal Intergenic
chr5	99533787	99534916	Distal Intergenic
chr5	100396481	100397985	Distal Intergenic
chr5	100398181	100398834	Distal Intergenic
chr5	100398875	100399323	Distal Intergenic
chr5	100404826	100405097	Distal Intergenic
chr5	100405963	100407610	Distal Intergenic
chr5	100488377	100488699	Distal Intergenic
chr5	100844469	100844830	Distal Intergenic
chr5	100845518	100845882	Distal Intergenic
chr5	100845977	100846183	Distal Intergenic
chr5	100857910	100858539	Distal Intergenic
chr5	100858785	100859091	Distal Intergenic
chr5	100928875	100929074	Distal Intergenic

chr5	100947427	100947784	Distal Intergenic
chr5	101051059	101052372	Distal Intergenic
chr5	101061858	101062125	Distal Intergenic
chr5	101529856	101531691	Distal Intergenic
chr5	101591704	101593151	Exon
chr5	101645879	101646370	Distal Intergenic
chr5	102171068	102171747	Distal Intergenic
chr5	102276611	102277258	Intron
chr5	102835666	102835932	Distal Intergenic
chr5	103793892	103795768	Distal Intergenic
chr5	103795890	103797089	Distal Intergenic
chr5	103805397	103806097	Distal Intergenic
chr5	103806156	103806401	Distal Intergenic
chr5	103824689	103824925	Distal Intergenic
chr5	103848658	103850218	Distal Intergenic
chr5	103855009	103855461	Distal Intergenic
chr5	103879292	103880565	Distal Intergenic
chr5	103887897	103888489	Distal Intergenic
chr5	103888559	103888762	Distal Intergenic
chr5	103947355	103948549	Distal Intergenic
chr5	104024662	104026298	Distal Intergenic
chr5	104120782	104121153	Distal Intergenic
chr5	104161238	104161975	Distal Intergenic
chr5	104165464	104167635	Distal Intergenic
chr5	104181632	104182484	Distal Intergenic
chr5	104182620	104182871	Distal Intergenic
chr5	104183149	104183995	Distal Intergenic
chr5	104187063	104187818	Distal Intergenic
chr5	104201824	104202067	Distal Intergenic
chr5	104202191	104202682	Distal Intergenic
chr5	104203261	104204930	Distal Intergenic
chr5	104207024	104208085	Distal Intergenic
chr5	104208626	104209184	Distal Intergenic
chr5	104248991	104249277	Distal Intergenic
chr5	104250711	104251060	Distal Intergenic
chr5	104251206	104253704	Distal Intergenic
chr5	104257942	104258143	Distal Intergenic
chr5	104258214	104258671	Distal Intergenic
chr5	104475339	104475691	Distal Intergenic
chr5	104836300	104837412	Distal Intergenic
chr5	105583515	105583947	Distal Intergenic
chr5	105949118	105949411	Distal Intergenic
chr5	105949519	105949813	Distal Intergenic
chr5	105950064	105950383	Distal Intergenic

chr5	105950894	105951103	Distal Intergenic
chr5	105951285	105953244	Distal Intergenic
chr5	105993569	105994469	Distal Intergenic
chr5	106065663	106066138	Distal Intergenic
chr5	106066174	106066586	Distal Intergenic
chr5	106206092	106206449	Distal Intergenic
chr5	106207306	106209120	Distal Intergenic
chr5	106221568	106222098	Distal Intergenic
chr5	106222249	106222469	Distal Intergenic
chr5	106227517	106228134	Distal Intergenic
chr5	106229501	106229775	Distal Intergenic
chr5	106235018	106235258	Distal Intergenic
chr5	106250907	106252612	Distal Intergenic
chr5	106304441	106304667	Distal Intergenic
chr5	106304821	106305183	Distal Intergenic
chr5	106306126	106306355	Distal Intergenic
chr5	106353209	106353432	Distal Intergenic
chr5	106353495	106353705	Distal Intergenic
chr5	107430829	107431169	Intron
chr5	107553758	107553997	Intron
chr5	107697383	107697837	Intron
chr5	107697886	107698243	Intron
chr5	107920705	107922031	Distal Intergenic
chr5	108222617	108222903	Intron
chr5	108231898	108232100	Intron
chr5	108274418	108274635	Intron
chr5	108276201	108277153	Intron
chr5	108330721	108330960	Intron
chr5	108366834	108367915	Intron
chr5	108400807	108401543	Intron
chr5	108401644	108401914	Intron
chr5	108416938	108417144	Intron
chr5	108424737	108425924	Intron
chr5	108494749	108495121	Intron
chr5	108528978	108529338	Exon
chr5	108560776	108561619	Distal Intergenic
chr5	108697501	108697863	Intron
chr5	108741231	108741531	Intron
chr5	108890678	108891006	Distal Intergenic
chr5	108891194	108891601	Distal Intergenic
chr5	109057797	109058614	Intron
chr5	109098462	109098692	Intron
chr5	109143079	109143278	Intron
chr5	110528811	110529132	Distal Intergenic

chr5	110755268	110755893	Intron
chr5	110856956	110858198	Intron
chr5	111088590	111088886	Intron
chr5	111089087	111089292	Intron
chr5	111133155	111133363	Intron
chr5	111229845	111232321	Intron
chr5	111232882	111233385	Intron
chr5	111233423	111234480	Intron
chr5	111237266	111237480	Intron
chr5	111237754	111237957	Intron
chr5	111238008	111238260	Intron
chr5	111739188	111739688	Intron
chr5	111759936	111760265	Distal Intergenic
chr5	111970689	111971005	Distal Intergenic
chr5	112023052	112023452	Distal Intergenic
chr5	112219284	112220145	Intron
chr5	112309411	112309913	Distal Intergenic
chr5	112310082	112310737	Distal Intergenic
chr5	112707446	112707780	Intron
chr5	112707939	112710992	Intron
chr5	112724098	112724542	Intron
chr5	112726381	112726825	Intron
chr5	112752744	112753082	Intron
chr5	112753193	112755191	Intron
chr5	113534123	113535145	Distal Intergenic
chr5	113535581	113536402	Distal Intergenic
chr5	113581818	113583613	Distal Intergenic
chr5	113583708	113584127	Distal Intergenic
chr5	113644874	113645929	Distal Intergenic
chr5	113808784	113808999	Exon
chr5	113990247	113991606	Intron
chr5	113994369	113994568	Intron
chr5	114135335	114136051	Distal Intergenic
chr5	114221707	114222199	Distal Intergenic
chr5	114291031	114291260	Distal Intergenic
chr5	114292082	114295339	Distal Intergenic
chr5	114323381	114323668	Distal Intergenic
chr5	114323896	114324162	Distal Intergenic
chr5	114324610	114324944	Distal Intergenic
chr5	114328587	114330116	Distal Intergenic
chr5	114357136	114357914	Distal Intergenic
chr5	114380003	114380287	Distal Intergenic
chr5	114425496	114425695	Distal Intergenic
chr5	114821510	114821950	Distal Intergenic

chr5	114822086	114823889	Distal Intergenic
chr5	114828107	114828344	Distal Intergenic
chr5	114829255	114829958	Distal Intergenic
chr5	114830616	114831215	Distal Intergenic
chr5	114843129	114843341	Distal Intergenic
chr5	114847793	114848614	Distal Intergenic
chr5	114856232	114858524	3' UTR
chr5	114858573	114859211	3' UTR
chr5	115097295	115098280	Distal Intergenic
chr5	115098322	115099663	Distal Intergenic
chr5	115100982	115101339	Distal Intergenic
chr5	115101678	115101945	Distal Intergenic
chr5	115183677	115184162	Intron
chr5	115201054	115201962	Intron
chr5	115222089	115222374	Intron
chr5	115297081	115297683	Promoter
chr5	115447505	115447907	Intron
chr5	115449866	115453143	Intron
chr5	115457772	115458010	Intron
chr5	115458127	115458656	Intron
chr5	115512402	115513547	Intron
chr5	115518699	115520023	Intron
chr5	115521121	115522683	Intron
chr5	115602374	115602604	Intron
chr5	115762554	115764414	Distal Intergenic
chr5	115941177	115941493	Distal Intergenic
chr5	115941549	115942506	Distal Intergenic
chr5	115953126	115953465	Distal Intergenic
chr5	115964652	115965302	Distal Intergenic
chr5	115971772	115972109	Distal Intergenic
chr5	115975369	115977055	Distal Intergenic
chr5	115983933	115985655	Distal Intergenic
chr5	115993479	115993943	Distal Intergenic
chr5	115994429	115995320	Distal Intergenic
chr5	115995459	115996044	Distal Intergenic
chr5	115997573	115997795	Distal Intergenic
chr5	116010051	116010627	Distal Intergenic
chr5	116013775	116014417	Distal Intergenic
chr5	116022494	116022761	Distal Intergenic
chr5	116022965	116023601	Distal Intergenic
chr5	116459105	116459322	Distal Intergenic
chr5	116459561	116460165	Distal Intergenic
chr5	116870520	116871218	Intron
chr5	116872811	116873538	Intron

chr5	116877421	116878798	Intron
chr5	116895996	116897088	Intron
chr5	117558097	117558296	Distal Intergenic
chr5	117558467	117559022	Distal Intergenic
chr5	117565025	117565626	Distal Intergenic
chr5	117573262	117573604	Distal Intergenic
chr5	117584273	117585019	Distal Intergenic
chr5	117608646	117609362	Distal Intergenic
chr5	117632067	117632935	Distal Intergenic
chr5	117729242	117729442	Distal Intergenic
chr5	117810492	117811488	Distal Intergenic
chr5	117812017	117812528	Distal Intergenic
chr5	117812566	117813470	Distal Intergenic
chr5	118339110	118340432	Exon
chr5	118534528	118534808	Intron
chr5	118534845	118535131	Intron
chr5	118898668	118899123	Distal Intergenic
chr5	118899330	118899828	Distal Intergenic
chr5	118899953	118900196	Distal Intergenic
chr5	118901877	118902104	Distal Intergenic
chr5	118902192	118902391	Distal Intergenic
chr5	118902482	118902762	Distal Intergenic
chr5	118909335	118909932	Distal Intergenic
chr5	119062828	119063843	Distal Intergenic
chr5	119077386	119078004	Distal Intergenic
chr5	119099129	119100226	Distal Intergenic
chr5	119579029	119579500	Distal Intergenic
chr5	119579992	119580282	Distal Intergenic
chr5	119590132	119590654	Distal Intergenic
chr5	119602490	119604124	Distal Intergenic
chr5	119606388	119607108	Distal Intergenic
chr5	119617562	119617942	Distal Intergenic
chr5	119663507	119663719	Distal Intergenic
chr5	119663892	119664112	Distal Intergenic
chr5	119749998	119750403	Distal Intergenic
chr5	120067873	120068541	Distal Intergenic
chr5	120120187	120120620	Distal Intergenic
chr5	120965995	120966962	Distal Intergenic
chr5	120967045	120968192	Distal Intergenic
chr5	121294954	121295316	Distal Intergenic
chr5	121654476	121654815	Intron
chr5	121737428	121737935	Intron
chr5	121744504	121746208	Intron
chr5	121843502	121844133	Distal Intergenic

chr5	121847210	121847745	Distal Intergenic
chr5	121888252	121889642	Distal Intergenic
chr5	121902736	121906483	Distal Intergenic
chr5	121987298	121988077	Intron
chr5	122255992	122257480	Intron
chr5	122269444	122269831	Intron
chr5	122594920	122595935	Distal Intergenic
chr5	122616245	122617524	Distal Intergenic
chr5	122749468	122750604	Intron
chr5	122754270	122754469	Intron
chr5	122968584	122970225	Distal Intergenic
chr5	122974257	122975709	Distal Intergenic
chr5	122975776	122977049	Distal Intergenic
chr5	122997348	122997596	Distal Intergenic
chr5	123094319	123094551	Distal Intergenic
chr5	123332605	123333238	Distal Intergenic
chr5	123585780	123592021	Distal Intergenic
chr5	123592222	123592433	Distal Intergenic
chr5	123775629	123776812	Distal Intergenic
chr5	123777092	123777911	Distal Intergenic
chr5	123814227	123815067	Distal Intergenic
chr5	124133128	124133845	Distal Intergenic
chr5	124146048	124146270	Distal Intergenic
chr5	124146350	124146602	Distal Intergenic
chr5	124198754	124199440	Distal Intergenic
chr5	124312694	124313679	Distal Intergenic
chr5	124313761	124314073	Distal Intergenic
chr5	124314284	124314531	Distal Intergenic
chr5	124314674	124314881	Distal Intergenic
chr5	124315217	124315621	Distal Intergenic
chr5	124318920	124320612	Distal Intergenic
chr5	124320649	124320911	Distal Intergenic
chr5	124428121	124428673	Distal Intergenic
chr5	124430384	124430876	Distal Intergenic
chr5	124480541	124480742	Distal Intergenic
chr5	124480930	124481907	Distal Intergenic
chr5	124485493	124485693	Distal Intergenic
chr5	124486015	124486240	Distal Intergenic
chr5	124496922	124498042	Distal Intergenic
chr5	124501886	124502593	Distal Intergenic
chr5	124616139	124616388	Distal Intergenic
chr5	124616429	124616735	Distal Intergenic
chr5	124618399	124619119	Distal Intergenic
chr5	124788745	124790515	Distal Intergenic

chr5	124801706	124802362	Distal Intergenic
chr5	124802524	124803789	Distal Intergenic
chr5	124874793	124875179	Distal Intergenic
chr5	124876450	124876752	Distal Intergenic
chr5	124889931	124890178	Distal Intergenic
chr5	124892157	124892373	Distal Intergenic
chr5	124892571	124893846	Distal Intergenic
chr5	124894196	124894449	Distal Intergenic
chr5	124894889	124895246	Distal Intergenic
chr5	124895916	124896617	Distal Intergenic
chr5	124896757	124897031	Distal Intergenic
chr5	124932540	124933897	Distal Intergenic
chr5	124963180	124963421	Distal Intergenic
chr5	125552735	125552934	Distal Intergenic
chr5	125561120	125562968	Distal Intergenic
chr5	125563045	125563273	Distal Intergenic
chr5	125606091	125606492	Distal Intergenic
chr5	125614667	125614920	Distal Intergenic
chr5	125615126	125615891	Distal Intergenic
chr5	125616125	125616478	Distal Intergenic
chr5	125620305	125622555	Distal Intergenic
chr5	125627118	125628318	Distal Intergenic
chr5	125634955	125636577	Distal Intergenic
chr5	125644598	125646699	Distal Intergenic
chr5	125647232	125647498	Distal Intergenic
chr5	125658773	125659039	Distal Intergenic
chr5	125669736	125670210	Distal Intergenic
chr5	125725588	125726326	Intron
chr5	125726411	125727031	Intron
chr5	125732536	125734186	Intron
chr5	125734520	125734920	Intron
chr5	125869590	125870088	Distal Intergenic
chr5	125870187	125875167	Downstream
chr5	125886398	125887181	Intron
chr5	128090980	128091337	Distal Intergenic
chr5	128091464	128091777	Distal Intergenic
chr5	128100422	128100955	Distal Intergenic
chr5	128101026	128101426	Distal Intergenic
chr5	129527083	129527328	Distal Intergenic
chr5	130024339	130024636	Distal Intergenic
chr5	130029468	130029829	Distal Intergenic
chr5	130029866	130031136	Distal Intergenic
chr5	130032598	130033149	Distal Intergenic
chr5	130033374	130033576	Distal Intergenic

chr5	130292428	130292733	Distal Intergenic
chr5	130292816	130293105	Distal Intergenic
chr5	130293276	130293504	Distal Intergenic
chr5	130505338	130505614	Distal Intergenic
chr5	130539569	130539909	3' UTR
chr5	130564992	130566397	Distal Intergenic
chr5	130584265	130586347	Distal Intergenic
chr5	130647933	130648712	Intron
chr5	130653600	130654023	Intron
chr5	130660539	130660743	Intron
chr5	130660824	130662027	Intron
chr5	130662206	130662684	Intron
chr5	130662861	130663939	Intron
chr5	130664008	130664230	Intron
chr5	130730754	130731207	Distal Intergenic
chr5	131318044	131318963	Intron
chr5	131991775	131992161	Exon
chr5	132013120	132013522	Intron
chr5	132033538	132034662	Intron
chr5	132042040	132042277	Exon
chr5	132088870	132090168	3' UTR
chr5	132373642	132374655	Distal Intergenic
chr5	134578629	134578873	Intron
chr5	134722035	134722483	Intron
chr5	134912626	134913414	Intron
chr5	135015994	135016370	Distal Intergenic
chr5	135027815	135028516	Distal Intergenic
chr5	135052515	135054112	Distal Intergenic
chr5	135057023	135057904	Distal Intergenic
chr5	135087937	135088725	Distal Intergenic
chr5	135114238	135114437	Distal Intergenic
chr5	135114610	135114837	Distal Intergenic
chr5	135114927	135115211	Distal Intergenic
chr5	135155904	135156687	Distal Intergenic
chr5	135312304	135312581	Distal Intergenic
chr5	135312639	135313125	Distal Intergenic
chr5	135411525	135411760	Distal Intergenic
chr5	135412061	135412384	Distal Intergenic
chr5	135426669	135427331	Distal Intergenic
chr5	135434923	135435455	Distal Intergenic
chr5	135508740	135509728	Intron
chr5	135663488	135663732	Intron
chr5	135869806	135870373	Distal Intergenic
chr5	136009470	136009741	Distal Intergenic

chr5	136096979	136098036	Distal Intergenic
chr5	136102177	136102796	Distal Intergenic
chr5	136115850	136116385	Distal Intergenic
chr5	136119696	136119899	Distal Intergenic
chr5	136146370	136146683	Distal Intergenic
chr5	136146735	136146942	Distal Intergenic
chr5	136178512	136179122	Distal Intergenic
chr5	136183654	136183936	Distal Intergenic
chr5	136353619	136354182	Intron
chr5	136354277	136354478	Intron
chr5	136355028	136356319	Intron
chr5	136357962	136359870	Intron
chr5	136359962	136361275	Intron
chr5	136361464	136361949	Intron
chr5	136363275	136364100	Intron
chr5	136364290	136364911	Intron
chr5	136368601	136369679	Intron
chr5	136405689	136405948	Intron
chr5	136408315	136408674	Intron
chr5	136409659	136410481	Intron
chr5	136410884	136411093	Intron
chr5	136421160	136422403	Intron
chr5	136478538	136478863	Intron
chr5	136482003	136482898	Intron
chr5	136483170	136483418	Intron
chr5	136490447	136490716	Intron
chr5	136490753	136493374	Intron
chr5	136527576	136528225	Intron
chr5	136569825	136570286	Intron
chr5	136612049	136612259	Intron
chr5	136645918	136646196	Intron
chr5	136646353	136648869	Intron
chr5	136896882	136898197	Distal Intergenic
chr5	136941640	136942569	Distal Intergenic
chr5	136942703	136942910	Distal Intergenic
chr5	136943326	136943727	Distal Intergenic
chr5	136954988	136956298	3' UTR
chr5	136988936	136990908	Intron
chr5	137007632	137009071	Promoter
chr5	137010028	137010286	Promoter
chr5	137028763	137029070	Promoter
chr5	137030002	137030321	Intron
chr5	137034783	137035383	Intron
chr5	137035488	137036324	Intron

chr5	137039848	137040497	Intron
chr5	137040632	137041873	Promoter
chr5	137041953	137043529	Promoter
chr5	137043829	137044037	Intron
chr5	137044208	137044502	Intron
chr5	137059882	137060114	Intron
chr5	137060279	137061400	Intron
chr5	137067486	137068490	Intron
chr5	137084736	137085051	Intron
chr5	137106001	137107566	Intron
chr5	137107720	137110260	Intron
chr5	137117428	137119088	Intron
chr5	137122975	137124184	Intron
chr5	137128381	137128605	Intron
chr5	137129194	137129558	Intron
chr5	137130300	137130728	Intron
chr5	137132831	137133954	Intron
chr5	137398861	137399402	Distal Intergenic
chr5	137429315	137429514	Distal Intergenic
chr5	137438001	137438238	Distal Intergenic
chr5	137438371	137438580	Distal Intergenic
chr5	137548308	137549198	Promoter
chr5	137555707	137556340	Distal Intergenic
chr5	137578169	137579494	Distal Intergenic
chr5	137735128	137735344	Intron
chr5	137735455	137735709	Exon
chr5	137815075	137815544	Distal Intergenic
chr5	137942598	137943079	Distal Intergenic
chr5	137943118	137943362	Distal Intergenic
chr5	137974236	137974460	Distal Intergenic
chr5	138187222	138187712	Intron
chr5	138388299	138389362	Intron
chr5	138558260	138559178	Distal Intergenic
chr5	138628070	138628270	Promoter
chr5	139183511	139183760	Intron
chr5	139189324	139189798	Exon
chr5	139350946	139351204	Intron
chr5	139374208	139374612	Intron
chr5	139374655	139375682	Intron
chr5	139464108	139465527	Distal Intergenic
chr5	139465720	139466175	Distal Intergenic
chr5	139469033	139470043	Distal Intergenic
chr5	139672695	139673292	Intron
chr5	139705628	139706145	Distal Intergenic

chr5	140208209	140209291	Promoter
chr5	140209429	140209691	Exon
chr5	140210158	140210357	Intron
chr5	140212442	140212953	Intron
chr5	140213495	140214364	Promoter
chr5	140268682	140268942	Intron
chr5	140269118	140269351	Intron
chr5	140273126	140273359	Intron
chr5	140556733	140557181	Promoter
chr5	140797671	140797993	Promoter
chr5	140808922	140809371	Promoter
chr5	140894361	140895224	3' UTR
chr5	140920232	140920503	Intron
chr5	140990386	140990732	Intron
chr5	140991244	140991674	Intron
chr5	141083857	141084117	Distal Intergenic
chr5	141084613	141085218	Distal Intergenic
chr5	141085270	141085776	Distal Intergenic
chr5	141086756	141086974	Distal Intergenic
chr5	141087014	141087247	Distal Intergenic
chr5	141156779	141158621	Distal Intergenic
chr5	142880389	142880824	Distal Intergenic
chr5	143530915	143531147	Distal Intergenic
chr5	143555845	143557594	Intron
chr5	143557943	143558586	Intron
chr5	143558625	143559711	Intron
chr5	143575720	143576128	Intron
chr5	143576312	143579320	Intron
chr5	143579887	143585074	Promoter
chr5	143585208	143585787	Promoter
chr5	143668723	143668922	Intron
chr5	143682486	143682930	Intron
chr5	143723012	143723679	Intron
chr5	143723745	143724373	Intron
chr5	143724680	143725043	Intron
chr5	143725544	143726102	Intron
chr5	143810282	143810490	Intron
chr5	143810564	143810815	Intron
chr5	143818030	143818882	Intron
chr5	143819466	143819694	Intron
chr5	144026627	144026826	Distal Intergenic
chr5	144033960	144034695	Distal Intergenic
chr5	144141228	144141514	Distal Intergenic
chr5	144175627	144176007	Distal Intergenic

chr5	144272994	144273194	Distal Intergenic
chr5	144278257	144279954	Distal Intergenic
chr5	144286092	144288381	Distal Intergenic
chr5	144289004	144289551	Distal Intergenic
chr5	144379162	144379694	Distal Intergenic
chr5	144396838	144399702	Distal Intergenic
chr5	144402673	144403707	Distal Intergenic
chr5	145049785	145050197	Distal Intergenic
chr5	145050495	145050817	Distal Intergenic
chr5	145079025	145079224	Distal Intergenic
chr5	145595685	145596927	Intron
chr5	145659120	145659319	Intron
chr5	145659580	145660263	Intron
chr5	145665456	145665856	3' UTR
chr5	145680098	145680399	Distal Intergenic
chr5	145691106	145692229	Distal Intergenic
chr5	145692406	145692659	Distal Intergenic
chr5	145710846	145711714	Distal Intergenic
chr5	145713798	145714072	Distal Intergenic
chr5	145715028	145716193	Distal Intergenic
chr5	145727972	145728373	Distal Intergenic
chr5	145728732	145729581	Distal Intergenic
chr5	145739427	145740226	Distal Intergenic
chr5	145863260	145863906	Intron
chr5	145875648	145875856	Intron
chr5	145876670	145877525	Intron
chr5	145952277	145952555	Distal Intergenic
chr5	145965480	145965935	Distal Intergenic
chr5	147084624	147084856	Intron
chr5	147633630	147633901	Distal Intergenic
chr5	147644493	147644729	Distal Intergenic
chr5	147644774	147645070	Distal Intergenic
chr5	147801963	147804400	Exon
chr5	147804513	147805455	Exon
chr5	147810915	147811204	Intron
chr5	147887746	147887945	Intron
chr5	147895094	147896615	Intron
chr5	147901598	147902074	Intron
chr5	147902254	147902931	Exon
chr5	147970420	147970901	Intron
chr5	148672608	148672935	Intron
chr5	149179385	149179653	Intron
chr5	149184873	149186732	Intron
chr5	149231062	149231489	3' UTR

chr5	149235876	149238301	3' UTR
chr5	149246766	149247632	Exon
chr5	149255335	149255583	Intron
chr5	149255774	149255983	Intron
chr5	149301146	149301378	Exon
chr5	149444553	149444843	Intron
chr5	149444885	149445268	Intron
chr5	149445342	149445775	Intron
chr5	149445813	149446125	Intron
chr5	149459289	149460271	Promoter
chr5	149588409	149588776	Intron
chr5	149589006	149589278	3' UTR
chr5	149614289	149614927	Intron
chr5	149777006	149777342	Intron
chr5	149777482	149777859	Intron
chr5	149806387	149806605	Distal Intergenic
chr5	149807106	149807333	Distal Intergenic
chr5	149995204	149995623	Intron
chr5	150191688	150193385	Distal Intergenic
chr5	151260466	151261520	Intron
chr5	151402706	151403411	Intron
chr5	151403489	151403745	Intron
chr5	151414431	151414693	Intron
chr5	151424984	151426511	Intron
chr5	151428079	151428755	Intron
chr5	151467269	151468170	Intron
chr5	151509188	151509486	Intron
chr5	151967103	151967776	Distal Intergenic
chr5	151969096	151969352	Distal Intergenic
chr5	151976031	151976865	Distal Intergenic
chr5	152005831	152006359	Intron
chr5	152054494	152054719	Intron
chr5	152209890	152210151	Intron
chr6	3238285	3238742	Intron
chr6	4140958	4141178	Intron
chr6	5079186	5079528	Intron
chr6	5079929	5080295	Intron
chr6	5088798	5090051	Intron
chr6	5247170	5248332	Intron
chr6	5250137	5250344	Intron
chr6	5250385	5250729	Intron
chr6	5250774	5251241	Intron
chr6	6527618	6528857	Intron
chr6	6536473	6536703	Intron

chr6	6536877	6537102	Intron
chr6	6538229	6539134	Intron
chr6	6541198	6543412	Intron
chr6	6607307	6607702	Intron
chr6	7165701	7166030	Intron
chr6	7732888	7734289	Intron
chr6	7795517	7795789	Intron
chr6	7795888	7796619	Intron
chr6	7797016	7798854	Intron
chr6	7806525	7807922	Intron
chr6	7807968	7808261	Intron
chr6	7891814	7892207	Exon
chr6	7893405	7893696	Intron
chr6	7893739	7894149	Intron
chr6	7969310	7969649	Intron
chr6	7998332	7998545	Intron
chr6	8302338	8302743	Distal Intergenic
chr6	11798396	11800243	Distal Intergenic
chr6	11800349	11800617	Distal Intergenic
chr6	11824514	11826755	Distal Intergenic
chr6	11857122	11857692	Distal Intergenic
chr6	11875420	11875740	Distal Intergenic
chr6	12762872	12763715	Intron
chr6	12763788	12764467	Intron
chr6	13332995	13333241	Distal Intergenic
chr6	13336558	13336803	Distal Intergenic
chr6	13350537	13352091	Distal Intergenic
chr6	13526391	13527200	Distal Intergenic
chr6	13531877	13532094	Distal Intergenic
chr6	13627444	13627656	Intron
chr6	13821028	13822693	Distal Intergenic
chr6	13832410	13833219	Distal Intergenic
chr6	17015214	17016806	Distal Intergenic
chr6	17022804	17023120	Distal Intergenic
chr6	17023204	17023551	Distal Intergenic
chr6	17058853	17060108	Distal Intergenic
chr6	17092569	17093722	Distal Intergenic
chr6	17120821	17121058	Exon
chr6	17121189	17121772	Intron
chr6	17121881	17122302	Intron
chr6	17143464	17144941	Distal Intergenic
chr6	17161477	17161684	Distal Intergenic
chr6	17196686	17198267	Distal Intergenic
chr6	17209053	17209360	Distal Intergenic

chr6	17214937	17215485	Distal Intergenic
chr6	17215766	17216540	Distal Intergenic
chr6	17231197	17232944	Distal Intergenic
chr6	17256019	17256750	Distal Intergenic
chr6	17256964	17257440	Distal Intergenic
chr6	17257525	17258081	Distal Intergenic
chr6	17258549	17258888	Distal Intergenic
chr6	17259068	17259483	Distal Intergenic
chr6	17264587	17267902	Distal Intergenic
chr6	17359021	17361257	Distal Intergenic
chr6	17413942	17417005	Intron
chr6	17427934	17429995	Intron
chr6	17443444	17446228	Intron
chr6	17446293	17450689	Intron
chr6	17450808	17451260	Intron
chr6	17451353	17452008	Intron
chr6	17453190	17457808	Intron
chr6	17522525	17524274	Intron
chr6	17562194	17563229	Distal Intergenic
chr6	17587546	17588327	Distal Intergenic
chr6	17668747	17670112	Exon
chr6	17760883	17761639	3' UTR
chr6	18192506	18192713	Intron
chr6	18220964	18221671	Intron
chr6	18798628	18798967	Distal Intergenic
chr6	18811243	18811537	Distal Intergenic
chr6	19611022	19611357	Distal Intergenic
chr6	19623041	19624444	Distal Intergenic
chr6	21580398	21580598	Distal Intergenic
chr6	22328717	22329062	Distal Intergenic
chr6	22367169	22367561	Distal Intergenic
chr6	22393548	22393758	Distal Intergenic
chr6	22393903	22394221	Distal Intergenic
chr6	22397113	22399438	Distal Intergenic
chr6	22741980	22742189	Distal Intergenic
chr6	22752630	22752835	Distal Intergenic
chr6	22817478	22817677	Distal Intergenic
chr6	22894762	22895018	Distal Intergenic
chr6	22895228	22895474	Distal Intergenic
chr6	23118777	23120523	Distal Intergenic
chr6	23132583	23133809	Distal Intergenic
chr6	24613733	24614580	Intron
chr6	24656174	24657724	Intron
chr6	28178333	28179423	Distal Intergenic

chr6	28179759	28180002	Distal Intergenic
chr6	28272411	28272787	Distal Intergenic
chr6	28272858	28273059	Distal Intergenic
chr6	28371860	28372150	Distal Intergenic
chr6	28487603	28487834	Distal Intergenic
chr6	28573909	28574127	Distal Intergenic
chr6	28766417	28766675	Distal Intergenic
chr6	28766779	28767185	Distal Intergenic
chr6	28771951	28774331	Distal Intergenic
chr6	28811220	28811536	Distal Intergenic
chr6	28811887	28812989	Distal Intergenic
chr6	28814151	28815675	Distal Intergenic
chr6	28816207	28818050	Distal Intergenic
chr6	28978256	28978698	Distal Intergenic
chr6	28980416	28980631	Distal Intergenic
chr6	28981014	28983395	Distal Intergenic
chr6	28986042	28987466	Distal Intergenic
chr6	29023462	29024670	Intron
chr6	29032358	29034566	Exon
chr6	29039280	29040150	Intron
chr6	29114259	29114488	Distal Intergenic
chr6	29222701	29223288	Distal Intergenic
chr6	29248851	29249176	Distal Intergenic
chr6	29258324	29258854	Distal Intergenic
chr6	29265181	29266414	Distal Intergenic
chr6	29266726	29267807	Distal Intergenic
chr6	29298476	29298737	Distal Intergenic
chr6	29322457	29322814	Downstream
chr6	29497126	29497884	Promoter
chr6	29497962	29499338	Promoter
chr6	29536208	29537009	Intron
chr6	29559010	29560171	Intron
chr6	29679604	29680706	Distal Intergenic
chr6	29685787	29686926	Distal Intergenic
chr6	29717727	29718087	Promoter
chr6	29718506	29718803	Promoter
chr6	29718902	29719787	Promoter
chr6	29719831	29721173	Promoter
chr6	29722087	29723008	Distal Intergenic
chr6	29997311	29997675	Intron
chr6	29998381	29999518	Intron
chr6	30006979	30007745	Intron
chr6	30123245	30123473	Intron
chr6	30123573	30124758	Exon

chr6	30202182	30203468	Exon
chr6	30250731	30251575	Intron
chr6	30251628	30251911	Intron
chr6	30251991	30252539	Intron
chr6	30252771	30253006	Intron
chr6	30253260	30254007	Intron
chr6	30257285	30258775	Exon
chr6	30296558	30297364	Promoter
chr6	30302624	30303316	Intron
chr6	30340738	30341001	Distal Intergenic
chr6	30458958	30460381	3' UTR
chr6	30511966	30512586	3' UTR
chr6	30512860	30513094	3' UTR
chr6	30999217	30999719	Intron
chr6	31010829	31012560	Distal Intergenic
chr6	31015137	31016929	Distal Intergenic
chr6	31024874	31025218	Intron
chr6	31025401	31025987	Intron
chr6	31030496	31031260	Distal Intergenic
chr6	31031455	31032697	Distal Intergenic
chr6	31037714	31039448	Distal Intergenic
chr6	31050579	31050778	Distal Intergenic
chr6	31057015	31057241	Distal Intergenic
chr6	31062237	31062489	Distal Intergenic
chr6	31062570	31063104	Distal Intergenic
chr6	31063181	31065199	Distal Intergenic
chr6	31065236	31065544	Distal Intergenic
chr6	31065877	31066086	Distal Intergenic
chr6	31074958	31077050	Downstream
chr6	31170371	31170717	3' UTR
chr6	32812516	32813658	Promoter
chr6	32967952	32968944	Distal Intergenic
chr6	33198730	33199550	Distal Intergenic
chr6	33240033	33240769	Promoter
chr6	33670917	33671502	Intron
chr6	34297661	34298257	Intron
chr6	34304123	34304329	Intron
chr6	34304495	34304797	Intron
chr6	34334032	34334375	Intron
chr6	34448272	34448504	Intron
chr6	34547600	34549343	Distal Intergenic
chr6	34549474	34549938	Distal Intergenic
chr6	34550305	34550504	Distal Intergenic
chr6	34551872	34552089	Downstream

chr6	34558333	34558999	3' UTR
chr6	34559156	34560134	Intron
chr6	34560187	34561145	Intron
chr6	34569431	34569727	Intron
chr6	34569860	34570539	Intron
chr6	34572317	34575204	Exon
chr6	34657004	34658957	Intron
chr6	34659012	34660505	Intron
chr6	34660547	34661343	Intron
chr6	34663411	34663753	Promoter
chr6	34666779	34667814	Distal Intergenic
chr6	34668057	34668390	Distal Intergenic
chr6	34671432	34672959	Distal Intergenic
chr6	34682255	34682564	Distal Intergenic
chr6	34686719	34687303	Distal Intergenic
chr6	34697086	34697538	Distal Intergenic
chr6	34697783	34698220	Distal Intergenic
chr6	34699064	34700729	Distal Intergenic
chr6	34700784	34701123	Distal Intergenic
chr6	34730522	34730862	Intron
chr6	34731244	34731469	Intron
chr6	34746183	34748309	Distal Intergenic
chr6	34812754	34813035	Intron
chr6	34813168	34813546	Intron
chr6	34815696	34818038	Intron
chr6	34818317	34820062	Intron
chr6	34902350	34903282	Intron
chr6	34916260	34916489	Intron
chr6	34921161	34922076	Intron
chr6	34970551	34970967	Intron
chr6	34977376	34978234	Intron
chr6	35083452	35083695	Intron
chr6	35374732	35375465	Intron
chr6	35376086	35376321	Intron
chr6	35469243	35469498	Intron
chr6	35488956	35489854	Distal Intergenic
chr6	35492778	35493230	Distal Intergenic
chr6	37153162	37154197	Distal Intergenic
chr6	37306276	37307006	Distal Intergenic
chr6	37320684	37321147	Promoter
chr6	37321207	37321508	Promoter
chr6	37327263	37328176	Intron
chr6	37335534	37335811	Intron
chr6	37338070	37339704	Exon

chr6	37349258	37349624	Intron
chr6	37378131	37378370	Distal Intergenic
chr6	37381219	37382260	Distal Intergenic
chr6	37405720	37406786	Intron
chr6	37407389	37408893	Intron
chr6	37415173	37416871	Intron
chr6	37471502	37471701	Distal Intergenic
chr6	37567758	37567976	Distal Intergenic
chr6	37685622	37686624	Distal Intergenic
chr6	38188443	38188647	Intron
chr6	38188977	38189230	Intron
chr6	38204782	38205332	Intron
chr6	38303764	38304909	Intron
chr6	38324389	38324708	Intron
chr6	38369503	38370111	Intron
chr6	38383449	38383760	Intron
chr6	38431096	38432985	Intron
chr6	38483001	38483682	Intron
chr6	38500535	38500897	Intron
chr6	38501003	38501202	Intron
chr6	38501949	38502536	Intron
chr6	38575493	38576264	Intron
chr6	38582673	38584021	Intron
chr6	38592394	38593646	Intron
chr6	38594017	38595373	Intron
chr6	38779628	38779980	Intron
chr6	38824852	38825184	Intron
chr6	39193160	39194447	Intron
chr6	39284066	39284376	Exon
chr6	39313821	39314439	Exon
chr6	39460579	39460964	Intron
chr6	39464748	39466308	Intron
chr6	39542156	39542583	Intron
chr6	39542637	39542855	Intron
chr6	39731250	39732808	Distal Intergenic
chr6	40053872	40054652	Distal Intergenic
chr6	40421462	40421737	Intron
chr6	40689782	40690140	Distal Intergenic
chr6	41124021	41124269	Downstream
chr6	41490032	41490460	Distal Intergenic
chr6	41498230	41499122	Distal Intergenic
chr6	41506969	41507372	Distal Intergenic
chr6	41507422	41509893	Distal Intergenic
chr6	41510843	41511256	Distal Intergenic

chr6	42056537	42057132	Distal Intergenic
chr6	42297924	42298276	Intron
chr6	42298327	42299591	Intron
chr6	42300603	42300805	Intron
chr6	42302401	42302668	Intron
chr6	42328699	42328898	Intron
chr6	42329019	42329888	Intron
chr6	42331526	42332426	Intron
chr6	42340776	42341164	Intron
chr6	42345726	42347538	Intron
chr6	42369291	42370112	Intron
chr6	42439558	42440522	Distal Intergenic
chr6	42440701	42441061	Distal Intergenic
chr6	42491990	42492432	Distal Intergenic
chr6	42642342	42642649	Intron
chr6	42998096	42999287	Distal Intergenic
chr6	43005007	43005791	3' UTR
chr6	43009951	43010599	Exon
chr6	43215619	43216873	Intron
chr6	43217162	43217624	Intron
chr6	43241848	43242606	Intron
chr6	43242672	43243081	Intron
chr6	43332085	43332670	Intron
chr6	45791734	45792468	Distal Intergenic
chr6	47189210	47189692	Distal Intergenic
chr6	47189760	47190300	Distal Intergenic
chr6	47219973	47220319	Intron
chr6	47278319	47278580	Promoter
chr6	47404413	47405498	Distal Intergenic
chr6	47405632	47405941	Distal Intergenic
chr6	47477606	47478037	Intron
chr6	47510046	47510409	Intron
chr6	47544448	47544650	Intron
chr6	47545534	47545756	Intron
chr6	47738419	47738627	Distal Intergenic
chr6	47762194	47762727	Intron
chr6	47763761	47764155	Intron
chr6	47827870	47830053	Distal Intergenic
chr6	47871016	47871217	Intron
chr6	48035957	48036174	Promoter
chr6	48374627	48374910	Distal Intergenic
chr6	48382666	48382886	Distal Intergenic
chr6	48383073	48383493	Distal Intergenic
chr6	48779048	48780637	Distal Intergenic

chr6	49023370	49023754	Distal Intergenic
chr6	49209934	49210485	Distal Intergenic
chr6	49212825	49214057	Distal Intergenic
chr6	49214095	49214341	Distal Intergenic
chr6	50313541	50313784	Distal Intergenic
chr6	50327839	50330532	Distal Intergenic
chr6	50331222	50333412	Distal Intergenic
chr6	50333547	50333805	Distal Intergenic
chr6	50333904	50334488	Distal Intergenic
chr6	50337466	50337679	Distal Intergenic
chr6	50337715	50338519	Distal Intergenic
chr6	50339055	50341593	Distal Intergenic
chr6	50341655	50341927	Distal Intergenic
chr6	50344864	50345109	Distal Intergenic
chr6	50345705	50346090	Distal Intergenic
chr6	50353937	50355554	Distal Intergenic
chr6	50357539	50359551	Distal Intergenic
chr6	50359614	50359813	Distal Intergenic
chr6	50391603	50392206	Distal Intergenic
chr6	50392340	50392539	Distal Intergenic
chr6	50404672	50405088	Distal Intergenic
chr6	50405531	50405730	Distal Intergenic
chr6	50405867	50406155	Distal Intergenic
chr6	50406235	50406780	Distal Intergenic
chr6	50406981	50408481	Distal Intergenic
chr6	50409764	50409987	Distal Intergenic
chr6	50410212	50410812	Distal Intergenic
chr6	50637627	50637891	Distal Intergenic
chr6	50974513	50975391	Distal Intergenic
chr6	51009937	51011737	Distal Intergenic
chr6	51026468	51027476	Distal Intergenic
chr6	51068836	51069355	Distal Intergenic
chr6	51069655	51070251	Distal Intergenic
chr6	51418060	51419852	Distal Intergenic
chr6	51494257	51495124	Intron
chr6	51495253	51495730	Intron
chr6	51503835	51504299	Intron
chr6	52108747	52110269	Promoter
chr6	52663490	52663689	Intron
chr6	52663752	52664614	5' UTR
chr6	53384784	53385488	Intron
chr6	53411897	53413615	Distal Intergenic
chr6	53447627	53447953	Intron
chr6	53448109	53449044	Intron

chr6	53552520	53553070	Distal Intergenic
chr6	53573910	53574230	Distal Intergenic
chr6	53592400	53593106	Distal Intergenic
chr6	53593251	53593870	Distal Intergenic
chr6	54040086	54040618	Intron
chr6	54040854	54041088	Intron
chr6	54042537	54042932	Intron
chr6	54046578	54046892	Intron
chr6	54152901	54154264	Distal Intergenic
chr6	54654072	54654856	Distal Intergenic
chr6	54682066	54682415	Distal Intergenic
chr6	54687601	54687958	Distal Intergenic
chr6	54767329	54767886	Intron
chr6	54816722	54816921	Distal Intergenic
chr6	54817001	54817478	Distal Intergenic
chr6	54826887	54827104	Distal Intergenic
chr6	56746600	56747265	Intron
chr6	56747777	56748236	Intron
chr6	56750164	56750700	Intron
chr6	56786922	56788802	Intron
chr6	56806142	56807418	Intron
chr6	56810046	56810565	Intron
chr6	56851673	56852058	Intron
chr6	57653392	57653601	Distal Intergenic
chr6	57761457	57764096	Distal Intergenic
chr6	57774270	57774898	Distal Intergenic
chr6	58790761	58791817	Distal Intergenic
chr6	58791882	58792546	Distal Intergenic
chr6	58797495	58797912	Distal Intergenic
chr6	59252685	59252991	Distal Intergenic
chr6	59253090	59253942	Distal Intergenic
chr6	61156914	61157559	Distal Intergenic
chr6	61175625	61175856	Distal Intergenic
chr6	61182964	61184844	Distal Intergenic
chr6	61192514	61193216	Distal Intergenic
chr6	61196660	61197013	Distal Intergenic
chr6	61689573	61689810	Distal Intergenic
chr6	63848036	63848581	Distal Intergenic
chr6	64066508	64066707	Distal Intergenic
chr6	64992651	64993120	Intron
chr6	65540329	65540552	Intron
chr6	65556996	65557240	Intron
chr6	66485365	66485670	Distal Intergenic
chr6	66859670	66859881	Distal Intergenic

chr6	66859930	66861779	Distal Intergenic
chr6	66946693	66947591	Distal Intergenic
chr6	66963769	66965446	Distal Intergenic
chr6	66986031	66986360	Distal Intergenic
chr6	66987600	66987854	Distal Intergenic
chr6	66990729	66992326	Distal Intergenic
chr6	67008103	67008589	Distal Intergenic
chr6	67008783	67009004	Distal Intergenic
chr6	67020785	67022159	Distal Intergenic
chr6	67080696	67080926	Distal Intergenic
chr6	67081077	67081443	Distal Intergenic
chr6	67083578	67085526	Distal Intergenic
chr6	67111183	67111382	Distal Intergenic
chr6	67216904	67217598	Distal Intergenic
chr6	67217828	67218458	Distal Intergenic
chr6	67218809	67219575	Distal Intergenic
chr6	67250899	67251280	Distal Intergenic
chr6	67251922	67252182	Distal Intergenic
chr6	67253043	67253896	Distal Intergenic
chr6	67263570	67263974	Distal Intergenic
chr6	67384024	67386159	Distal Intergenic
chr6	67386223	67386432	Distal Intergenic
chr6	67391191	67392203	Distal Intergenic
chr6	67404051	67404387	Distal Intergenic
chr6	67430870	67431587	Distal Intergenic
chr6	67431636	67432016	Distal Intergenic
chr6	67480767	67485691	Distal Intergenic
chr6	70493830	70494463	Intron
chr6	70494512	70494864	Intron
chr6	70583058	70583877	Intron
chr6	70589265	70589484	5' UTR
chr6	70719980	70720181	Intron
chr6	70723736	70724359	Intron
chr6	70763718	70763921	Intron
chr6	70774606	70775168	Intron
chr6	70829775	70831023	Intron
chr6	71038566	71039941	Distal Intergenic
chr6	71221767	71222305	Intron
chr6	71493900	71494686	Intron
chr6	71540927	71541176	Intron
chr6	71541252	71541837	Intron
chr6	71858916	71859193	Distal Intergenic
chr6	72006323	72006730	Exon
chr6	72167295	72167826	Distal Intergenic

chr6	72167886	72168187	Distal Intergenic
chr6	72266003	72266271	Distal Intergenic
chr6	72294799	72295072	Distal Intergenic
chr6	72297517	72297953	Distal Intergenic
chr6	72319564	72322360	Distal Intergenic
chr6	72339610	72340057	Distal Intergenic
chr6	72340113	72340418	Distal Intergenic
chr6	72423386	72424009	Distal Intergenic
chr6	72497773	72502819	Distal Intergenic
chr6	72566605	72566832	Distal Intergenic
chr6	72626944	72627577	Intron
chr6	72627638	72628019	Intron
chr6	72748400	72748624	Intron
chr6	72748717	72749022	Intron
chr6	72806859	72807811	Exon
chr6	72822335	72822876	Intron
chr6	72831219	72832245	Intron
chr6	72838969	72839169	Intron
chr6	72848398	72849600	Intron
chr6	72850178	72850976	Intron
chr6	72876593	72877698	Intron
chr6	72907731	72908221	Intron
chr6	73198390	73198629	Distal Intergenic
chr6	76915037	76915575	Distal Intergenic
chr6	76916845	76917207	Distal Intergenic
chr6	81896022	81896423	Distal Intergenic
chr6	81970731	81971208	Distal Intergenic
chr6	81982092	81982837	Distal Intergenic
chr6	81985338	81986190	Distal Intergenic
chr6	81990886	81991089	Distal Intergenic
chr6	81991259	81991540	Distal Intergenic
chr6	81991708	81992474	Distal Intergenic
chr6	82002250	82002914	Distal Intergenic
chr6	82047448	82048136	Distal Intergenic
chr6	82048229	82048976	Distal Intergenic
chr6	82049083	82049471	Distal Intergenic
chr6	82812703	82813908	Distal Intergenic
chr6	83085808	83087873	Distal Intergenic
chr6	83091902	83093781	Distal Intergenic
chr6	83094848	83095375	Distal Intergenic
chr6	83095417	83095720	Distal Intergenic
chr6	83383251	83383451	Distal Intergenic
chr6	83385268	83385570	Distal Intergenic
chr6	83391763	83392735	Distal Intergenic

chr6	83392779	83393485	Distal Intergenic
chr6	83393548	83393799	Distal Intergenic
chr6	83406903	83407596	Distal Intergenic
chr6	83439027	83439290	Distal Intergenic
chr6	83445942	83446146	Distal Intergenic
chr6	83446186	83446611	Distal Intergenic
chr6	83446782	83447127	Distal Intergenic
chr6	83451486	83453311	Distal Intergenic
chr6	83724573	83725013	Intron
chr6	84128554	84129240	Intron
chr6	84129306	84129573	Intron
chr6	84454425	84455272	Distal Intergenic
chr6	84493712	84493966	Distal Intergenic
chr6	84508732	84512150	Distal Intergenic
chr6	84515038	84516844	Distal Intergenic
chr6	84521052	84521298	Distal Intergenic
chr6	84551082	84551794	Distal Intergenic
chr6	84725846	84726560	Distal Intergenic
chr6	84842938	84843428	Intron
chr6	84870330	84870667	Exon
chr6	84870712	84871686	Exon
chr6	84963420	84964328	Distal Intergenic
chr6	85024720	85025393	Distal Intergenic
chr6	85036644	85037207	Distal Intergenic
chr6	85037259	85038147	Distal Intergenic
chr6	85049121	85049448	Distal Intergenic
chr6	85298340	85301155	Distal Intergenic
chr6	85316695	85317063	Distal Intergenic
chr6	85323566	85323787	Distal Intergenic
chr6	85332340	85332748	Distal Intergenic
chr6	85401991	85402441	Intron
chr6	85887383	85887612	Distal Intergenic
chr6	85910999	85911884	Distal Intergenic
chr6	86099293	86099528	Intron
chr6	86099825	86100315	Exon
chr6	86107511	86107920	Distal Intergenic
chr6	86130443	86130745	Distal Intergenic
chr6	86168777	86170126	Intron
chr6	86268371	86269442	Intron
chr6	86274008	86276076	5' UTR
chr6	86316037	86316448	Downstream
chr6	86321527	86321894	3' UTR
chr6	86344842	86345973	Intron
chr6	86354326	86355633	Distal Intergenic

chr6	86387990	86388260	Promoter
chr6	86388901	86389105	Promoter
chr6	86389195	86389425	Promoter
chr6	86474454	86474679	Distal Intergenic
chr6	86474902	86475386	Distal Intergenic
chr6	86475503	86475917	Distal Intergenic
chr6	86476691	86477697	Distal Intergenic
chr6	86550243	86550472	Distal Intergenic
chr6	86555525	86555904	Distal Intergenic
chr6	86556008	86556606	Distal Intergenic
chr6	86589845	86590662	Distal Intergenic
chr6	86799024	86799321	Distal Intergenic
chr6	86922455	86923207	Distal Intergenic
chr6	87018738	87020091	Distal Intergenic
chr6	87136218	87137938	Distal Intergenic
chr6	87172515	87172804	Distal Intergenic
chr6	87172930	87177728	Distal Intergenic
chr6	87186857	87188241	Distal Intergenic
chr6	87208257	87210061	Distal Intergenic
chr6	87215118	87215543	Distal Intergenic
chr6	87221419	87222779	Distal Intergenic
chr6	87222865	87223252	Distal Intergenic
chr6	87274949	87275950	Distal Intergenic
chr6	87336572	87336771	Distal Intergenic
chr6	87460094	87460502	Distal Intergenic
chr6	87788907	87789517	Distal Intergenic
chr6	87789556	87789816	Distal Intergenic
chr6	87799964	87800205	Intron
chr6	87800365	87800726	Intron
chr6	87818637	87819011	Distal Intergenic
chr6	87994349	87994867	5' UTR
chr6	88003529	88003734	Intron
chr6	88003773	88004133	Intron
chr6	88415393	88415618	Distal Intergenic
chr6	88468415	88468750	Distal Intergenic
chr6	88524153	88525028	Intron
chr6	88713382	88715128	Distal Intergenic
chr6	88723509	88725374	Distal Intergenic
chr6	88874438	88874863	Promoter
chr6	89363327	89364643	Intron
chr6	89372835	89373087	Intron
chr6	89411517	89411719	Intron
chr6	89502248	89502782	Intron
chr6	89502824	89503069	Intron

chr6	89826098	89827548	Promoter
chr6	90298620	90298872	Intron
chr6	90309231	90310011	Intron
chr6	90310108	90311079	Intron
chr6	90456993	90457199	Exon
chr6	91151792	91153585	Distal Intergenic
chr6	91157645	91160266	Distal Intergenic
chr6	91160333	91161821	Distal Intergenic
chr6	91162479	91162949	Distal Intergenic
chr6	91164396	91165271	Distal Intergenic
chr6	91182882	91183085	Distal Intergenic
chr6	91282860	91283788	Intron
chr6	91322701	91322914	Distal Intergenic
chr6	91339262	91339952	Distal Intergenic
chr6	91401905	91402566	Distal Intergenic
chr6	91404743	91404978	Distal Intergenic
chr6	91417395	91417610	Distal Intergenic
chr6	91422884	91423207	Distal Intergenic
chr6	91423643	91424166	Distal Intergenic
chr6	91424268	91426813	Distal Intergenic
chr6	91433230	91433471	Distal Intergenic
chr6	91439067	91439307	Distal Intergenic
chr6	91526702	91527011	Distal Intergenic
chr6	91529110	91531562	Distal Intergenic
chr6	91596660	91597077	Distal Intergenic
chr6	91597114	91597496	Distal Intergenic
chr6	91597533	91597970	Distal Intergenic
chr6	91602146	91602571	Distal Intergenic
chr6	91602671	91602932	Distal Intergenic
chr6	91606489	91606688	Distal Intergenic
chr6	91638982	91639824	Distal Intergenic
chr6	91676345	91677492	Distal Intergenic
chr6	92046825	92047277	Distal Intergenic
chr6	92189546	92191535	Distal Intergenic
chr6	92374285	92376346	Intron
chr6	92377376	92377778	Intron
chr6	92377828	92378067	Intron
chr6	92400009	92400251	Exon
chr6	93207251	93207865	Distal Intergenic
chr6	93874143	93874499	Distal Intergenic
chr6	94050376	94050793	Intron
chr6	94051244	94051488	Intron
chr6	94105698	94106562	Intron
chr6	94160681	94160965	Distal Intergenic

chr6	94161026	94161321	Distal Intergenic
chr6	94175150	94175752	Distal Intergenic
chr6	94175898	94176585	Distal Intergenic
chr6	94213541	94215796	Distal Intergenic
chr6	94224862	94225543	Distal Intergenic
chr6	94225673	94225877	Distal Intergenic
chr6	94577104	94577525	Distal Intergenic
chr6	94577843	94578083	Distal Intergenic
chr6	94578239	94578539	Distal Intergenic
chr6	94584824	94586451	Distal Intergenic
chr6	94589169	94589483	Distal Intergenic
chr6	94589542	94590932	Distal Intergenic
chr6	94595963	94596716	Distal Intergenic
chr6	94596769	94597046	Distal Intergenic
chr6	94597232	94597471	Distal Intergenic
chr6	94694866	94695215	Distal Intergenic
chr6	94695425	94695624	Distal Intergenic
chr6	94695702	94696528	Distal Intergenic
chr6	96002934	96003256	Distal Intergenic
chr6	96003817	96004254	Distal Intergenic
chr6	96021029	96021276	Distal Intergenic
chr6	97097475	97098688	Distal Intergenic
chr6	97128165	97129593	Distal Intergenic
chr6	97129771	97130036	Distal Intergenic
chr6	97155211	97155544	Distal Intergenic
chr6	97243045	97244919	3' UTR
chr6	97253782	97256338	Intron
chr6	97260962	97263149	Intron
chr6	97265200	97266733	Intron
chr6	97266802	97267030	Intron
chr6	97305975	97306395	Distal Intergenic
chr6	97396087	97397961	Intron
chr6	97456986	97457350	Promoter
chr6	97460036	97462419	Intron
chr6	97508804	97511233	Exon
chr6	97513446	97513787	Intron
chr6	97513842	97514181	Intron
chr6	97514325	97514551	Intron
chr6	97514598	97515018	Intron
chr6	97515065	97515350	Intron
chr6	97524590	97526143	Intron
chr6	97540903	97542634	Intron
chr6	97545573	97545892	Intron
chr6	97546036	97546792	Intron

chr6	97563084	97564147	Intron
chr6	97565015	97567842	Intron
chr6	98899193	98900625	Distal Intergenic
chr6	98904276	98905976	Distal Intergenic
chr6	98976958	98977493	Distal Intergenic
chr6	99034805	99035377	Distal Intergenic
chr6	99043059	99043529	Distal Intergenic
chr6	99045635	99046532	Distal Intergenic
chr6	99149033	99149904	Distal Intergenic
chr6	99167393	99168384	Distal Intergenic
chr6	99199770	99200116	Distal Intergenic
chr6	99293721	99294012	Distal Intergenic
chr6	99294158	99294645	Distal Intergenic
chr6	99300166	99301154	Distal Intergenic
chr6	99320078	99320422	Downstream
chr6	99325495	99325827	Intron
chr6	99342720	99343232	Intron
chr6	99393739	99393950	Intron
chr6	99396277	99398495	Promoter
chr6	99415808	99417712	Distal Intergenic
chr6	99417751	99417956	Distal Intergenic
chr6	99580274	99580676	Distal Intergenic
chr6	99612223	99612599	Distal Intergenic
chr6	99612928	99613529	Distal Intergenic
chr6	100748679	100751247	Distal Intergenic
chr6	100784728	100784981	Distal Intergenic
chr6	100785086	100785343	Distal Intergenic
chr6	101278753	101278976	Intron
chr6	101376981	101377451	Distal Intergenic
chr6	101414653	101415734	Distal Intergenic
chr6	103294720	103296268	Distal Intergenic
chr6	103296305	103296626	Distal Intergenic
chr6	103296782	103297249	Distal Intergenic
chr6	103309715	103309914	Distal Intergenic
chr6	103330710	103331188	Distal Intergenic
chr6	103339301	103339500	Distal Intergenic
chr6	103350883	103351719	Distal Intergenic
chr6	103460639	103461036	Distal Intergenic
chr6	103475443	103475759	Distal Intergenic
chr6	103475823	103476083	Distal Intergenic
chr6	105498123	105498573	Intron
chr6	108018934	108019297	Distal Intergenic
chr6	108392895	108393144	Intron
chr6	108397999	108398461	Distal Intergenic

chr6	108398707	108399026	Distal Intergenic
chr6	108401265	108402108	Distal Intergenic
chr6	108402208	108402635	Distal Intergenic
chr6	108480901	108482573	Distal Intergenic
chr6	108484826	108485025	Distal Intergenic
chr6	108609870	108610219	Distal Intergenic
chr6	108610705	108610904	Distal Intergenic
chr6	108615397	108615687	Promoter
chr6	108654444	108656254	Intron
chr6	108733091	108733368	Intron
chr6	108733530	108734470	Intron
chr6	108744706	108746955	Intron
chr6	109130604	109130848	Distal Intergenic
chr6	112981203	112981407	Distal Intergenic
chr6	112984376	112985039	Distal Intergenic
chr6	112995656	112995998	Distal Intergenic
chr6	112996575	112996865	Distal Intergenic
chr6	112996959	112997264	Distal Intergenic
chr6	113022701	113022925	Distal Intergenic
chr6	113023103	113023324	Distal Intergenic
chr6	113024824	113026088	Distal Intergenic
chr6	113027509	113029478	Distal Intergenic
chr6	113029553	113029805	Distal Intergenic
chr6	113033305	113034746	Distal Intergenic
chr6	113049081	113050220	Distal Intergenic
chr6	113116279	113117449	Distal Intergenic
chr6	113117582	113117819	Distal Intergenic
chr6	113257534	113258380	Distal Intergenic
chr6	113327511	113328094	Distal Intergenic
chr6	113421348	113422169	Distal Intergenic
chr6	113430164	113431867	Distal Intergenic
chr6	113436530	113437680	Distal Intergenic
chr6	113472982	113473304	Distal Intergenic
chr6	113640480	113640996	Distal Intergenic
chr6	113645836	113646725	Distal Intergenic
chr6	114371013	114372103	Intron
chr6	114373222	114374675	Intron
chr6	114398552	114400317	Intron
chr6	114408380	114408824	Intron
chr6	114705359	114706931	Distal Intergenic
chr6	114755750	114756608	Distal Intergenic
chr6	114811424	114811665	Distal Intergenic
chr6	114816068	114816663	Distal Intergenic
chr6	114824534	114826316	Distal Intergenic

chr6	114832922	114834013	Distal Intergenic
chr6	114838677	114839326	Distal Intergenic
chr6	114870249	114872236	Distal Intergenic
chr6	114880172	114880427	Distal Intergenic
chr6	114880790	114882583	Distal Intergenic
chr6	114882803	114887652	Distal Intergenic
chr6	114889315	114889772	Distal Intergenic
chr6	114894598	114895571	Distal Intergenic
chr6	114913844	114914243	Distal Intergenic
chr6	114914413	114914848	Distal Intergenic
chr6	114917503	114919033	Distal Intergenic
chr6	114937409	114940141	Distal Intergenic
chr6	114955038	114956561	Distal Intergenic
chr6	114956910	114957109	Distal Intergenic
chr6	115625467	115625764	Distal Intergenic
chr6	115626189	115626567	Distal Intergenic
chr6	115708743	115708990	Distal Intergenic
chr6	115709066	115710158	Distal Intergenic
chr6	115758069	115759114	Distal Intergenic
chr6	115852053	115852427	Distal Intergenic
chr6	116216111	116216813	Distal Intergenic
chr6	116356562	116358306	Intron
chr6	116369689	116369966	Intron
chr6	117666694	117666914	Intron
chr6	117791456	117791827	Intron
chr6	117792192	117792406	Intron
chr6	117792666	117792907	Intron
chr6	117792949	117793177	Intron
chr6	117828968	117829199	Intron
chr6	117829283	117829486	Intron
chr6	117829594	117829868	Intron
chr6	117829978	117830534	Intron
chr6	117856191	117856560	Intron
chr6	117857245	117858000	Intron
chr6	117858203	117858896	Exon
chr6	117863036	117863552	Intron
chr6	117865773	117867222	3' UTR
chr6	117867331	117868396	Intron
chr6	118404043	118405145	Intron
chr6	118405248	118405520	Intron
chr6	119439953	119440183	Intron
chr6	119613230	119613467	Intron
chr6	119732350	119733690	Distal Intergenic
chr6	119811680	119811995	Promoter

chr6	119814248	119814974	Distal Intergenic
chr6	119874618	119875033	Distal Intergenic
chr6	119960571	119960839	Distal Intergenic
chr6	119973790	119974002	Distal Intergenic
chr6	119986765	119987351	Distal Intergenic
chr6	119989569	119989786	Distal Intergenic
chr6	119989845	119990281	Distal Intergenic
chr6	120048409	120048811	Distal Intergenic
chr6	120053818	120054053	Distal Intergenic
chr6	120064709	120065275	Distal Intergenic
chr6	120066729	120067595	Distal Intergenic
chr6	120095141	120095549	Distal Intergenic
chr6	120122043	120122959	Distal Intergenic
chr6	120123170	120123571	Distal Intergenic
chr6	120139666	120141395	Distal Intergenic
chr6	120141503	120142691	Distal Intergenic
chr6	120142889	120143355	Distal Intergenic
chr6	120187206	120188490	Distal Intergenic
chr6	120212235	120214961	Distal Intergenic
chr6	120307040	120307411	Distal Intergenic
chr6	120312830	120314324	Distal Intergenic
chr6	120592021	120592420	Distal Intergenic
chr6	120747815	120750556	Distal Intergenic
chr6	120781519	120783032	Distal Intergenic
chr6	120785983	120787536	Distal Intergenic
chr6	120815879	120816273	Distal Intergenic
chr6	120822931	120824761	Distal Intergenic
chr6	120866938	120867288	Distal Intergenic
chr6	120877148	120877472	Distal Intergenic
chr6	120877517	120878278	Distal Intergenic
chr6	120937186	120937963	Distal Intergenic
chr6	120945109	120948188	Distal Intergenic
chr6	120949363	120949562	Distal Intergenic
chr6	120967156	120967509	Distal Intergenic
chr6	120967670	120968267	Distal Intergenic
chr6	120985224	120986085	Distal Intergenic
chr6	120989359	120989755	Distal Intergenic
chr6	120993319	120995982	Distal Intergenic
chr6	121013968	121015043	Distal Intergenic
chr6	121015209	121017952	Distal Intergenic
chr6	121018055	121018261	Distal Intergenic
chr6	121018344	121018877	Distal Intergenic
chr6	121035092	121038825	Distal Intergenic
chr6	121143901	121144179	Distal Intergenic

chr6	121144225	121144998	Distal Intergenic
chr6	122259064	122260152	Distal Intergenic
chr6	122551965	122552634	Distal Intergenic
chr6	122552706	122553196	Distal Intergenic
chr6	122760638	122761002	Distal Intergenic
chr6	122761455	122762117	Downstream
chr6	122769524	122769764	Intron
chr6	122776523	122776758	Intron
chr6	122818966	122819166	Intron
chr6	122819218	122820463	Intron
chr6	122954409	122955581	5' UTR
chr6	124365225	124365540	Intron
chr6	124435040	124435290	Intron
chr6	124633434	124634488	Intron
chr6	124647093	124648147	Intron
chr6	124650129	124650945	Intron
chr6	124662327	124662538	Intron
chr6	124667094	124669494	Intron
chr6	124688438	124689391	Intron
chr6	124689870	124690285	Intron
chr6	124690361	124691853	Intron
chr6	124707106	124707471	Intron
chr6	124755135	124756519	Intron
chr6	124763229	124763514	Intron
chr6	124763629	124764168	Intron
chr6	124905080	124905965	Intron
chr6	124915494	124915701	Intron
chr6	124915868	124916349	Intron
chr6	124989843	124990276	Intron
chr6	125017739	125018125	Intron
chr6	125081365	125081810	Intron
chr6	125110830	125111248	Intron
chr6	125114505	125114992	Intron
chr6	125116243	125116756	Intron
chr6	125116820	125117071	Intron
chr6	125141921	125142890	Intron
chr6	125205665	125206898	Distal Intergenic
chr6	125263658	125263911	Intron
chr6	125299486	125300171	Intron
chr6	125300208	125300821	Intron
chr6	125302220	125302466	Intron
chr6	125302619	125303006	Intron
chr6	125304061	125304915	Promoter
chr6	125327177	125328823	Intron

chr6	125363653	125363915	Intron
chr6	125371602	125372543	Intron
chr6	125372760	125376447	Intron
chr6	125383603	125384155	Intron
chr6	125417836	125418862	Distal Intergenic
chr6	125428186	125429002	Distal Intergenic
chr6	125430712	125434883	Distal Intergenic
chr6	125442432	125444209	Distal Intergenic
chr6	125453179	125453445	Distal Intergenic
chr6	125453609	125453997	Distal Intergenic
chr6	125898085	125898408	Distal Intergenic
chr6	125948332	125948721	Distal Intergenic
chr6	125948911	125949238	Distal Intergenic
chr6	125995309	125995910	Promoter
chr6	126059420	126059623	Intron
chr6	126121841	126122974	Intron
chr6	126188403	126189640	Intron
chr6	126241498	126242149	Exon
chr6	126297220	126298074	Intron
chr6	126315697	126317148	Exon
chr6	126317193	126317401	Intron
chr6	126324554	126325904	Intron
chr6	126325964	126326163	Intron
chr6	127099036	127099497	Intron
chr6	127099545	127100393	Intron
chr6	127275935	127276440	Intron
chr6	127279195	127280364	Intron
chr6	127280623	127281040	Intron
chr6	127325338	127326173	Intron
chr6	127326238	127326470	Intron
chr6	127941110	127942468	Distal Intergenic
chr6	127982277	127982508	Distal Intergenic
chr6	127993194	127993589	Distal Intergenic
chr6	127993783	127994176	Distal Intergenic
chr6	127996320	127998663	Distal Intergenic
chr6	128011928	128013199	Distal Intergenic
chr6	128052467	128053003	Intron
chr6	128054449	128055556	Intron
chr6	128084956	128085179	Intron
chr6	128085221	128086152	Intron
chr6	128092627	128093102	Intron
chr6	128096714	128096994	Intron
chr6	128097304	128097555	Intron
chr6	128232675	128232915	Intron

chr6	128246313	128246778	Distal Intergenic
chr6	128247594	128247869	Distal Intergenic
chr6	128248289	128249463	Distal Intergenic
chr6	128289019	128290388	3' UTR
chr6	128291810	128292362	Intron
chr6	128292522	128292738	Intron
chr6	128311976	128312542	Exon
chr6	128313566	128313765	Intron
chr6	128371223	128371438	Intron
chr6	128371590	128371789	Intron
chr6	128374997	128375196	Intron
chr6	128794693	128794901	Intron
chr6	128912952	128913211	Distal Intergenic
chr6	128925028	128925431	Distal Intergenic
chr6	128942796	128943026	Distal Intergenic
chr6	128943225	128943424	Distal Intergenic
chr6	128958103	128961958	Distal Intergenic
chr6	128962483	128964524	Distal Intergenic
chr6	129048084	129048283	Distal Intergenic
chr6	129113686	129114341	Distal Intergenic
chr6	129130496	129131450	Distal Intergenic
chr6	129131714	129131999	Distal Intergenic
chr6	129179634	129181052	Distal Intergenic
chr6	129181256	129181490	Distal Intergenic
chr6	129181713	129183988	Distal Intergenic
chr6	129184733	129188052	Distal Intergenic
chr6	129188120	129188371	Distal Intergenic
chr6	130013521	130013758	Intron
chr6	130013809	130014043	Intron
chr6	131331917	131334281	Intron
chr6	131337016	131337564	Intron
chr6	133055379	133055706	Promoter
chr6	133291401	133292412	Distal Intergenic
chr6	133503353	133504186	Distal Intergenic
chr6	133504387	133504647	Distal Intergenic
chr6	133985061	133985577	Intron
chr6	133986141	133986433	Intron
chr6	133986489	133986786	Intron
chr6	134032540	134032981	Intron
chr6	134079564	134080359	Intron
chr6	134517019	134517478	Intron
chr6	134525381	134525606	Intron
chr6	134525723	134526056	Intron
chr6	134701104	134702275	Distal Intergenic

chr6	134702389	134702607	Distal Intergenic
chr6	134870523	134870780	Intron
chr6	134871113	134871678	Intron
chr6	134871878	134872674	Intron
chr6	134872766	134875508	Intron
chr6	134875759	134878592	Intron
chr6	134879136	134879570	Intron
chr6	134940340	134941494	Distal Intergenic
chr6	134943245	134943456	Distal Intergenic
chr6	134943804	134944125	Exon
chr6	134944332	134944823	Distal Intergenic
chr6	134948465	134950103	Distal Intergenic
chr6	134950143	134951123	Distal Intergenic
chr6	134951169	134951434	Distal Intergenic
chr6	134961778	134962187	Distal Intergenic
chr6	134974755	134975149	Distal Intergenic
chr6	135010890	135011441	Distal Intergenic
chr6	135019847	135020706	Distal Intergenic
chr6	135200484	135200836	Distal Intergenic
chr6	135201033	135202852	Distal Intergenic
chr6	135215842	135216082	Distal Intergenic
chr6	135216283	135216512	Distal Intergenic
chr6	135245264	135245603	Intron
chr6	135245672	135246018	Intron
chr6	135293752	135294794	Intron
chr6	135312523	135312903	Intron
chr6	135312946	135314054	Intron
chr6	135314138	135314469	Intron
chr6	135337900	135339065	Intron
chr6	135454046	135455195	Distal Intergenic
chr6	136414567	136415048	Intron
chr6	136415128	136415373	Intron
chr6	136439176	136439443	Intron
chr6	136440654	136440992	Intron
chr6	136489650	136490667	Intron
chr6	136752164	136753869	Intron
chr6	136756879	136757207	Intron
chr6	136757258	136758012	Intron
chr6	136775631	136776775	Intron
chr6	136776879	136777267	Intron
chr6	136878648	136879933	3' UTR
chr6	136883234	136883474	Intron
chr6	136883677	136883876	Exon
chr6	136885490	136886235	Intron

chr6	136886275	136887703	Intron
chr6	136888260	136888577	Intron
chr6	136979169	136979391	Intron
chr6	136979594	136979972	Intron
chr6	140496273	140496583	Distal Intergenic
chr6	140496889	140497193	Distal Intergenic
chr6	140563687	140563967	Distal Intergenic
chr6	140568775	140571950	Distal Intergenic
chr6	140572568	140572861	Distal Intergenic
chr6	140572916	140573301	Distal Intergenic
chr6	140573540	140573869	Distal Intergenic
chr6	140574249	140574470	Distal Intergenic
chr6	142287238	142288908	Intron
chr6	142289065	142289343	Intron
chr6	142314424	142315503	Intron
chr6	142316061	142316401	Intron
chr6	143116883	143117145	Intron
chr6	143117282	143117552	Intron
chr6	143117790	143118511	Intron
chr6	144850687	144851256	Intron
chr6	145159921	145160255	Intron
chr6	145225088	145226032	Distal Intergenic
chr6	145240321	145240550	Distal Intergenic
chr6	145921602	145921944	Distal Intergenic
chr6	145926536	145926822	Distal Intergenic
chr6	145947258	145947493	3' UTR
chr6	146058978	146059833	Intron
chr6	146069180	146069419	Intron
chr6	146380595	146381398	Intron
chr6	146564560	146564821	Intron
chr6	146564963	146565498	Intron
chr6	146591213	146591496	Intron
chr6	146591884	146592123	Intron
chr6	146741929	146742238	Intron
chr6	146742294	146742774	Intron
chr6	146745962	146746741	Intron
chr6	146838358	146838672	Distal Intergenic
chr6	146847853	146848328	Distal Intergenic
chr6	146857041	146859814	Distal Intergenic
chr6	146883609	146883808	Distal Intergenic
chr6	146887033	146887561	Distal Intergenic
chr6	146887680	146887973	Distal Intergenic
chr6	146904045	146904298	Distal Intergenic
chr6	146908270	146908719	Distal Intergenic

chr6	146933414	146933626	Intron
chr6	146958706	146959175	Intron
chr6	147011495	147011805	Intron
chr6	147021323	147022195	5' UTR
chr6	147065997	147066377	Intron
chr6	147066488	147066758	Intron
chr6	147147582	147149019	Distal Intergenic
chr6	147209281	147212595	Intron
chr6	147212738	147213182	Intron
chr6	147213355	147216324	Intron
chr6	147216555	147216801	Intron
chr6	147217049	147217300	Intron
chr6	147224640	147224909	Intron
chr6	147225208	147226973	Intron
chr6	147241170	147242341	Intron
chr6	147249116	147250305	Intron
chr6	147270709	147271622	Intron
chr6	147272706	147273537	Intron
chr6	147338210	147341269	Intron
chr6	147346165	147347982	Intron
chr6	147361023	147363191	Intron
chr6	147366919	147367282	Intron
chr6	147367333	147367789	Intron
chr6	147374779	147375284	Intron
chr6	147375404	147376139	Intron
chr6	147482135	147482437	Intron
chr6	147482643	147483236	Intron
chr6	147689923	147690785	Intron
chr6	147899907	147900106	Distal Intergenic
chr6	147913302	147913649	Distal Intergenic
chr6	147913694	147913903	Distal Intergenic
chr6	148893370	148893741	Distal Intergenic
chr6	149136471	149137177	Intron
chr6	149141572	149143053	Intron
chr6	149208662	149210521	Intron
chr6	149258034	149258719	Intron
chr6	149303661	149304901	Intron
chr6	149316339	149316749	Intron
chr7	3021789	3022179	Intron
chr7	3028724	3029341	Intron
chr7	3067392	3068166	Intron
chr7	3135027	3135938	Distal Intergenic
chr7	3149863	3151664	Distal Intergenic
chr7	3167790	3168985	Distal Intergenic

chr7	3169045	3169272	Distal Intergenic
chr7	3275542	3275829	Distal Intergenic
chr7	3276025	3276238	Distal Intergenic
chr7	3289266	3293312	Distal Intergenic
chr7	3451336	3452653	Intron
chr7	3498966	3499341	Intron
chr7	3542187	3542634	Intron
chr7	3581138	3581497	Intron
chr7	3616675	3617120	Intron
chr7	3628360	3629191	Intron
chr7	3644934	3645757	Intron
chr7	3645818	3646019	Intron
chr7	3646234	3646561	Intron
chr7	3654900	3655104	Intron
chr7	3655311	3655518	Intron
chr7	3655596	3656046	Intron
chr7	3656177	3656610	Intron
chr7	3658490	3658910	Exon
chr7	4050257	4051232	Promoter
chr7	4085661	4086417	Intron
chr7	4087835	4088918	Intron
chr7	4101154	4101516	Intron
chr7	4453412	4453765	Distal Intergenic
chr7	4740229	4740428	Intron
chr7	4743978	4744889	Intron
chr7	4745065	4745280	Intron
chr7	4764145	4765300	Intron
chr7	4966464	4966908	Intron
chr7	4971051	4972253	Intron
chr7	4989808	4990055	Intron
chr7	4990186	4990649	Intron
chr7	5012413	5012680	Promoter
chr7	5012880	5013453	Promoter
chr7	5014151	5015287	Promoter
chr7	5032096	5033118	Intron
chr7	6106981	6107197	Distal Intergenic
chr7	13490343	13490860	Distal Intergenic
chr7	13506626	13507594	Distal Intergenic
chr7	13507676	13508142	Distal Intergenic
chr7	13508984	13509183	Distal Intergenic
chr7	13563520	13563954	Distal Intergenic
chr7	13595060	13595512	Distal Intergenic
chr7	13618812	13620105	Distal Intergenic
chr7	13622519	13623523	Distal Intergenic

chr7	13623943	13624459	Distal Intergenic
chr7	13724185	13725215	Distal Intergenic
chr7	13863627	13864549	Distal Intergenic
chr7	13876434	13877114	Distal Intergenic
chr7	13915581	13916598	Distal Intergenic
chr7	16528530	16531944	Intron
chr7	16533248	16534937	Intron
chr7	16540671	16541813	Intron
chr7	16541856	16542196	Intron
chr7	16548236	16548616	Intron
chr7	16561508	16562993	Intron
chr7	16569213	16569676	Promoter
chr7	16613603	16614599	Intron
chr7	16614667	16614866	Intron
chr7	16622149	16622839	Distal Intergenic
chr7	16634101	16634674	Distal Intergenic
chr7	16637432	16637687	Downstream
chr7	16637807	16638183	Downstream
chr7	16642105	16642971	Exon
chr7	16684151	16684384	Intron
chr7	16893495	16894777	Distal Intergenic
chr7	16951905	16952104	Distal Intergenic
chr7	16952166	16952376	Distal Intergenic
chr7	16952620	16955419	Distal Intergenic
chr7	16955584	16955784	Distal Intergenic
chr7	16984711	16984913	Distal Intergenic
chr7	16984986	16987521	Distal Intergenic
chr7	16987564	16989155	Distal Intergenic
chr7	16989698	16992030	Distal Intergenic
chr7	17109164	17111322	Distal Intergenic
chr7	17163242	17164128	Distal Intergenic
chr7	17167579	17167815	Distal Intergenic
chr7	17182296	17182907	Distal Intergenic
chr7	17186703	17187785	Distal Intergenic
chr7	17199544	17199892	Distal Intergenic
chr7	17439572	17439777	Distal Intergenic
chr7	17439877	17440188	Distal Intergenic
chr7	17590858	17591157	Distal Intergenic
chr7	17602443	17603033	Distal Intergenic
chr7	17610298	17611965	Distal Intergenic
chr7	17612435	17612842	Distal Intergenic
chr7	17612963	17613263	Distal Intergenic
chr7	18772246	18772929	Intron
chr7	18799726	18799975	Intron

chr7	18800050	18800922	Intron
chr7	18821585	18823215	Intron
chr7	18823642	18824508	Intron
chr7	19443729	19443954	Distal Intergenic
chr7	19588448	19588897	Distal Intergenic
chr7	19741405	19741736	Intron
chr7	19812639	19813335	Promoter
chr7	19814368	19814609	Distal Intergenic
chr7	19855279	19858331	Distal Intergenic
chr7	19929040	19929239	Distal Intergenic
chr7	19929579	19930752	Distal Intergenic
chr7	19934467	19934675	Distal Intergenic
chr7	19944084	19944311	Distal Intergenic
chr7	19944840	19945055	Distal Intergenic
chr7	19945721	19946510	Distal Intergenic
chr7	19946618	19947250	Distal Intergenic
chr7	19947303	19951694	Distal Intergenic
chr7	20043418	20045159	Distal Intergenic
chr7	20062552	20063901	Distal Intergenic
chr7	20064095	20064414	Distal Intergenic
chr7	20093754	20093969	Distal Intergenic
chr7	20094068	20094334	Distal Intergenic
chr7	20094419	20094817	Distal Intergenic
chr7	20146592	20147166	Distal Intergenic
chr7	20147581	20147782	Distal Intergenic
chr7	20157885	20158686	Distal Intergenic
chr7	20158753	20159379	Distal Intergenic
chr7	20163502	20163777	Distal Intergenic
chr7	20163832	20164536	Distal Intergenic
chr7	20164626	20164825	Distal Intergenic
chr7	20165676	20167051	Distal Intergenic
chr7	20194769	20195367	Intron
chr7	20250548	20251227	Intron
chr7	20289472	20289959	Distal Intergenic
chr7	20354342	20355157	Distal Intergenic
chr7	20355209	20355610	Distal Intergenic
chr7	20382256	20382647	Intron
chr7	20402082	20403883	5' UTR
chr7	20408919	20409175	Intron
chr7	20409264	20409658	Intron
chr7	20409805	20410223	Intron
chr7	20416099	20417913	Exon
chr7	20462001	20462206	Distal Intergenic
chr7	24867229	24868070	Intron

chr7	24868129	24868540	Intron
chr7	24897459	24898028	Intron
chr7	25150436	25150852	Distal Intergenic
chr7	25171209	25172759	Downstream
chr7	25179464	25180577	Intron
chr7	25234259	25234516	Distal Intergenic
chr7	25244673	25245462	Distal Intergenic
chr7	25246250	25247291	Distal Intergenic
chr7	25247505	25247963	Distal Intergenic
chr7	25255504	25256133	Distal Intergenic
chr7	25256275	25256968	Distal Intergenic
chr7	25257030	25258138	Distal Intergenic
chr7	25265225	25266506	Exon
chr7	25291800	25292241	Distal Intergenic
chr7	25315639	25315838	Distal Intergenic
chr7	25316019	25316607	Distal Intergenic
chr7	25384306	25385226	Distal Intergenic
chr7	25386731	25387177	Distal Intergenic
chr7	25418474	25418686	Distal Intergenic
chr7	25421285	25422329	Distal Intergenic
chr7	25422552	25423006	Distal Intergenic
chr7	25670041	25670347	Intron
chr7	25670535	25671144	Intron
chr7	25689308	25689878	Intron
chr7	25691929	25693159	Intron
chr7	25988861	25992066	Promoter
chr7	25993474	25995357	Distal Intergenic
chr7	26005025	26006134	Distal Intergenic
chr7	26006480	26006796	Distal Intergenic
chr7	26033438	26033678	Distal Intergenic
chr7	26033980	26034493	Distal Intergenic
chr7	26034637	26035369	Distal Intergenic
chr7	26053900	26055498	Distal Intergenic
chr7	26058945	26063798	Distal Intergenic
chr7	26064070	26064435	Distal Intergenic
chr7	26064593	26066467	Distal Intergenic
chr7	26066585	26066919	Distal Intergenic
chr7	26082715	26083045	Distal Intergenic
chr7	26178473	26179817	Distal Intergenic
chr7	26179865	26180201	Distal Intergenic
chr7	26429425	26430524	Distal Intergenic
chr7	26472253	26474561	Intron
chr7	26490569	26491516	Intron
chr7	26492625	26494452	Intron

chr7	26495506	26497071	Intron
chr7	26497107	26497483	Intron
chr7	26497686	26498023	Intron
chr7	26503552	26504338	Intron
chr7	26538916	26539129	Distal Intergenic
chr7	26539392	26539821	Distal Intergenic
chr7	26539865	26540253	Distal Intergenic
chr7	26565492	26567314	Distal Intergenic
chr7	26568009	26568304	Distal Intergenic
chr7	26568357	26568568	Distal Intergenic
chr7	26569031	26569503	Distal Intergenic
chr7	26573357	26574066	Exon
chr7	26616194	26617736	Distal Intergenic
chr7	26621986	26622478	Distal Intergenic
chr7	26628497	26630288	Distal Intergenic
chr7	26687178	26687719	Distal Intergenic
chr7	26981121	26982380	Intron
chr7	27776484	27777013	Distal Intergenic
chr7	27979018	27979267	Intron
chr7	27980201	27980999	Intron
chr7	28006961	28007297	Intron
chr7	28010159	28010994	Intron
chr7	28012935	28013246	Intron
chr7	28013336	28013616	Intron
chr7	28016402	28018233	Intron
chr7	28076349	28076850	Intron
chr7	28116043	28117545	Intron
chr7	28117584	28118120	Intron
chr7	28140557	28140793	Intron
chr7	28272026	28272920	Intron
chr7	28273007	28273235	Intron
chr7	28273289	28273552	Intron
chr7	28275491	28276650	Intron
chr7	28292816	28293113	Distal Intergenic
chr7	28338302	28338857	Promoter
chr7	28354106	28355553	Intron
chr7	28359068	28359674	Intron
chr7	28376160	28376359	Intron
chr7	28376820	28377475	Intron
chr7	28392858	28393813	Intron
chr7	28516450	28516777	Intron
chr7	28589586	28589811	Intron
chr7	28763869	28764077	3' UTR
chr7	28946655	28947797	Distal Intergenic

chr7	28953107	28953430	Distal Intergenic
chr7	28954103	28954495	Distal Intergenic
chr7	29002388	29002602	Distal Intergenic
chr7	29119479	29120350	Intron
chr7	29123889	29124121	Intron
chr7	29135860	29136059	Intron
chr7	29136256	29136693	Intron
chr7	29136850	29137049	Intron
chr7	29156066	29156510	Intron
chr7	29157086	29157649	Intron
chr7	29160142	29162533	5' UTR
chr7	29162583	29164962	Intron
chr7	29165292	29167195	Intron
chr7	29167532	29168157	Intron
chr7	29203071	29205381	Intron
chr7	29338087	29338286	Intron
chr7	29355108	29355413	Intron
chr7	29355548	29355857	Intron
chr7	29361587	29363307	Intron
chr7	29404310	29404661	Intron
chr7	29586381	29588298	Intron
chr7	29593061	29593291	Intron
chr7	29593511	29595218	Intron
chr7	29634446	29635758	Distal Intergenic
chr7	29650889	29651579	Distal Intergenic
chr7	29651772	29652242	Distal Intergenic
chr7	29652382	29652841	Distal Intergenic
chr7	29704225	29706309	Intron
chr7	29706510	29706851	Intron
chr7	29708799	29709520	Intron
chr7	29709704	29711578	Intron
chr7	29713902	29717308	Intron
chr7	29717442	29718058	Intron
chr7	29723638	29724083	Promoter
chr7	29724472	29725499	Promoter
chr7	29725924	29726338	Promoter
chr7	29737844	29739269	Intron
chr7	29742938	29743204	Intron
chr7	29743272	29743842	Intron
chr7	29744009	29745010	Intron
chr7	29745545	29745766	Intron
chr7	29745916	29746534	Intron
chr7	29746702	29747138	Intron
chr7	29747387	29748089	Intron

chr7	29754808	29757613	Distal Intergenic
chr7	29759634	29760205	Intron
chr7	29767062	29767294	Intron
chr7	29809941	29812340	Distal Intergenic
chr7	29964912	29965345	Intron
chr7	30022797	30023301	Intron
chr7	30032765	30034501	Distal Intergenic
chr7	30036168	30036617	Distal Intergenic
chr7	30036722	30037725	Distal Intergenic
chr7	30041738	30043124	Distal Intergenic
chr7	30060802	30061207	Intron
chr7	30061378	30062279	3' UTR
chr7	30126489	30128666	Intron
chr7	30134350	30134578	Intron
chr7	30134671	30135684	Intron
chr7	30135724	30138429	Intron
chr7	30227195	30228512	Distal Intergenic
chr7	30229112	30230644	Distal Intergenic
chr7	30235052	30235305	Distal Intergenic
chr7	30237942	30238343	Distal Intergenic
chr7	30239578	30239777	Distal Intergenic
chr7	30239832	30240127	Distal Intergenic
chr7	30243061	30243648	Distal Intergenic
chr7	30247203	30247513	Distal Intergenic
chr7	30247749	30247948	Distal Intergenic
chr7	30256088	30256483	Distal Intergenic
chr7	30256678	30257141	Distal Intergenic
chr7	30261909	30262271	Distal Intergenic
chr7	30262319	30262722	Distal Intergenic
chr7	30265899	30267039	Distal Intergenic
chr7	30269119	30270009	Distal Intergenic
chr7	30270121	30270520	Distal Intergenic
chr7	30290202	30291262	Distal Intergenic
chr7	30298114	30298332	Distal Intergenic
chr7	30552970	30553200	Downstream
chr7	30553346	30553664	Downstream
chr7	30738523	30738808	Promoter
chr7	30738906	30739177	Promoter
chr7	30767219	30767717	Intron
chr7	30768020	30768780	Intron
chr7	30879932	30880334	Intron
chr7	30954369	30955093	Intron
chr7	30979152	30980165	Distal Intergenic
chr7	30980299	30981183	Distal Intergenic

chr7	30981380	30981923	Distal Intergenic
chr7	31336757	31337337	Distal Intergenic
chr7	31337893	31338383	Distal Intergenic
chr7	31344830	31345272	Distal Intergenic
chr7	31347450	31348029	Distal Intergenic
chr7	31348196	31348623	Distal Intergenic
chr7	31372891	31373241	Distal Intergenic
chr7	31514822	31515062	Distal Intergenic
chr7	31533018	31533236	Distal Intergenic
chr7	31536475	31536678	Distal Intergenic
chr7	31543997	31544517	Distal Intergenic
chr7	31544598	31545190	Distal Intergenic
chr7	31547916	31549142	Distal Intergenic
chr7	31560842	31562104	Intron
chr7	31579399	31580283	Intron
chr7	31580392	31580837	Intron
chr7	31580905	31582375	Intron
chr7	31589413	31589818	Intron
chr7	31601033	31602687	Intron
chr7	31859086	31859461	Intron
chr7	31860618	31861784	Intron
chr7	34915678	34917339	Intron
chr7	34951767	34952015	Distal Intergenic
chr7	34952950	34953519	Distal Intergenic
chr7	34953625	34955159	Distal Intergenic
chr7	34998015	34999096	Intron
chr7	35018498	35019079	Intron
chr7	35051655	35052177	Intron
chr7	35052463	35054673	Exon
chr7	35127137	35128094	Intron
chr7	35163490	35164020	Exon
chr7	35172902	35174586	Intron
chr7	35174901	35175375	Intron
chr7	35414165	35415689	Exon
chr7	35438829	35439028	Distal Intergenic
chr7	35439819	35440131	Distal Intergenic
chr7	35590491	35591803	Distal Intergenic
chr7	35840818	35841201	Promoter
chr7	35861307	35861773	Intron
chr7	35861842	35862377	Intron
chr7	35869966	35870581	Promoter
chr7	35912174	35912561	Exon
chr7	35912635	35912916	Intron
chr7	36134057	36134256	Distal Intergenic

chr7	36180925	36181630	Distal Intergenic
chr7	38470767	38471046	Intron
chr7	38480324	38480736	Intron
chr7	38862367	38862577	Intron
chr7	38877213	38877877	Intron
chr7	38969266	38969473	Distal Intergenic
chr7	38969921	38970120	Distal Intergenic
chr7	39055969	39056175	Intron
chr7	46772895	46773188	Distal Intergenic
chr7	46808101	46808365	Distal Intergenic
chr7	47121625	47123366	Distal Intergenic
chr7	47123580	47124144	Distal Intergenic
chr7	47127903	47128748	Distal Intergenic
chr7	48341203	48341426	Intron
chr7	48648959	48649177	Intron
chr7	50654212	50654940	Downstream
chr7	50927040	50927264	Distal Intergenic
chr7	50927374	50927747	Distal Intergenic
chr7	51501283	51501515	Distal Intergenic
chr7	51501566	51502331	Distal Intergenic
chr7	51546363	51546860	Distal Intergenic
chr7	51660459	51660737	Distal Intergenic
chr7	51792152	51792372	Distal Intergenic
chr7	51869967	51870180	Distal Intergenic
chr7	51917334	51918838	Distal Intergenic
chr7	52003524	52003767	Distal Intergenic
chr7	52032235	52032811	Distal Intergenic
chr7	52071776	52071994	Distal Intergenic
chr7	52072058	52072668	Distal Intergenic
chr7	52081949	52082226	Distal Intergenic
chr7	52104660	52105251	Distal Intergenic
chr7	52105334	52105613	Distal Intergenic
chr7	52239526	52240164	Distal Intergenic
chr7	52242216	52243009	Distal Intergenic
chr7	52253065	52254034	Distal Intergenic
chr7	52270876	52271132	Distal Intergenic
chr7	52271178	52271638	Distal Intergenic
chr7	52272142	52274756	Distal Intergenic
chr7	52280707	52281460	Distal Intergenic
chr7	52306534	52306746	Distal Intergenic
chr7	52307095	52307430	Distal Intergenic
chr7	52316884	52317772	Distal Intergenic
chr7	52379330	52379552	Distal Intergenic
chr7	52379623	52379827	Distal Intergenic

chr7	52380126	52380615	Distal Intergenic
chr7	52380708	52380927	Distal Intergenic
chr7	52381620	52382666	Distal Intergenic
chr7	52382747	52384187	Distal Intergenic
chr7	52395122	52395524	Distal Intergenic
chr7	52395724	52396607	Distal Intergenic
chr7	52408820	52409667	Distal Intergenic
chr7	52409737	52409936	Distal Intergenic
chr7	52650336	52651030	Distal Intergenic
chr7	52668926	52669145	Distal Intergenic
chr7	52669210	52669681	Distal Intergenic
chr7	52715154	52716787	Distal Intergenic
chr7	52717510	52717723	Distal Intergenic
chr7	52747593	52748199	Distal Intergenic
chr7	52780779	52781281	Distal Intergenic
chr7	52781692	52782238	Distal Intergenic
chr7	52860985	52861719	Distal Intergenic
chr7	52961424	52961888	Distal Intergenic
chr7	52962594	52962845	Distal Intergenic
chr7	52963105	52963387	Distal Intergenic
chr7	52969526	52970480	Distal Intergenic
chr7	52970803	52971025	Distal Intergenic
chr7	52972470	52972729	Distal Intergenic
chr7	52972781	52973945	Distal Intergenic
chr7	52974103	52974465	Distal Intergenic
chr7	53026150	53026717	Distal Intergenic
chr7	53055599	53055963	Distal Intergenic
chr7	53057507	53058921	Distal Intergenic
chr7	53060003	53060614	Distal Intergenic
chr7	53069451	53069674	Distal Intergenic
chr7	53128191	53128445	Distal Intergenic
chr7	53128643	53128983	Distal Intergenic
chr7	53129184	53129573	Distal Intergenic
chr7	53176167	53176815	Distal Intergenic
chr7	54016451	54016899	Distal Intergenic
chr7	54051612	54051904	Distal Intergenic
chr7	54069703	54070217	Distal Intergenic
chr7	54094004	54095303	Distal Intergenic
chr7	54100261	54100627	Distal Intergenic
chr7	54100708	54101133	Distal Intergenic
chr7	54101175	54101388	Distal Intergenic
chr7	54101569	54102414	Distal Intergenic
chr7	54173965	54175316	Distal Intergenic
chr7	54213279	54213802	Distal Intergenic

chr7	54263411	54263782	Distal Intergenic
chr7	54278007	54278211	Distal Intergenic
chr7	54286317	54286839	Distal Intergenic
chr7	54286922	54287253	Distal Intergenic
chr7	54353715	54353933	Distal Intergenic
chr7	54366033	54366383	Distal Intergenic
chr7	55704439	55705043	Distal Intergenic
chr7	55913064	55913452	Intron
chr7	56017897	56018269	Distal Intergenic
chr7	56088316	56088852	Exon
chr7	56115812	56116826	Intron
chr7	56143422	56144523	Intron
chr7	56172669	56173386	Promoter
chr7	56174572	56174772	Promoter
chr7	56349265	56349860	Distal Intergenic
chr7	56675752	56676323	Distal Intergenic
chr7	56775663	56777197	Distal Intergenic
chr7	56777258	56779474	Distal Intergenic
chr7	56808282	56810491	Distal Intergenic
chr7	56810798	56811598	Distal Intergenic
chr7	56812207	56814502	Distal Intergenic
chr7	56814568	56816509	Distal Intergenic
chr7	56817224	56817464	Distal Intergenic
chr7	56817623	56818193	Distal Intergenic
chr7	56841285	56841495	Distal Intergenic
chr7	56841702	56842291	Distal Intergenic
chr7	56855763	56858962	Distal Intergenic
chr7	57014725	57015103	Distal Intergenic
chr7	57015283	57015511	Distal Intergenic
chr7	57016102	57018950	Distal Intergenic
chr7	57063957	57065116	Distal Intergenic
chr7	57137561	57137933	Distal Intergenic
chr7	57143078	57143766	Distal Intergenic
chr7	59001217	59001557	Distal Intergenic
chr7	59169130	59169647	Distal Intergenic
chr7	59222921	59223446	Distal Intergenic
chr7	59593027	59593655	Distal Intergenic
chr7	60483324	60484047	Distal Intergenic
chr7	61106225	61106481	Distal Intergenic
chr7	61106578	61107610	Distal Intergenic
chr7	62103424	62103789	Distal Intergenic
chr7	62791827	62792261	Distal Intergenic
chr7	62794910	62796126	Distal Intergenic
chr7	63097097	63097496	Distal Intergenic

chr7	63097666	63098779	Distal Intergenic
chr7	63114195	63114482	Distal Intergenic
chr7	63195045	63195261	Distal Intergenic
chr7	63217930	63218252	Distal Intergenic
chr7	63305792	63306009	Distal Intergenic
chr7	65061237	65062989	Distal Intergenic
chr7	66005230	66005708	Intron
chr7	66055628	66055835	Intron
chr7	66055883	66057673	Promoter
chr7	66077390	66078386	Distal Intergenic
chr7	66483382	66483610	Intron
chr7	66483793	66483997	Intron
chr7	66484160	66484422	Intron
chr7	66484730	66485392	Intron
chr7	68659275	68660414	Distal Intergenic
chr7	69021687	69022095	Distal Intergenic
chr7	69022215	69022634	Distal Intergenic
chr7	70627356	70628646	Intron
chr7	70641976	70642672	Intron
chr7	71001515	71001714	Intron
chr7	71084897	71085096	Intron
chr7	71195494	71196695	Distal Intergenic
chr7	71196815	71197970	Distal Intergenic
chr7	71209468	71210880	Distal Intergenic
chr7	71384504	71384703	Intron
chr7	71394558	71394922	Intron
chr7	71399692	71400447	Intron
chr7	71410406	71411394	Intron
chr7	71421528	71422658	Intron
chr7	71505441	71505672	Intron
chr7	71536904	71537180	Intron
chr7	72051307	72052122	Intron
chr7	72612883	72613766	Exon
chr7	72614844	72615423	Intron
chr7	73223958	73224327	Distal Intergenic
chr7	73322656	73323755	Distal Intergenic
chr7	73324058	73324349	Distal Intergenic
chr7	73345261	73346857	Distal Intergenic
chr7	73362953	73363313	Distal Intergenic
chr7	73369349	73370469	Distal Intergenic
chr7	73510230	73511540	Promoter
chr7	73513921	73514762	Intron
chr7	73515113	73515480	Intron
chr7	73520275	73522443	Exon

chr7	73742753	73743224	Intron
chr7	73887831	73889250	Intron
chr7	73891566	73891929	Intron
chr7	73900317	73900632	Intron
chr7	73900977	73901473	Intron
chr7	73901603	73902083	Intron
chr7	73902200	73902515	Intron
chr7	73971283	73971762	Intron
chr7	73971925	73973033	Exon
chr7	74428412	74429166	Intron
chr7	74431937	74432173	Exon
chr7	74432293	74433203	Exon
chr7	74586959	74588055	Promoter
chr7	74637135	74638665	Intron
chr7	74641118	74641511	Intron
chr7	74649902	74650107	Intron
chr7	74693309	74694218	Intron
chr7	74790299	74790952	Intron
chr7	74791851	74792058	Intron
chr7	74792301	74793192	Intron
chr7	74808507	74809869	Intron
chr7	74981138	74982135	Promoter
chr7	74982222	74982640	Promoter
chr7	74988775	74989340	Promoter
chr7	74998864	74999159	Intron
chr7	75097433	75097734	Intron
chr7	75097865	75098651	Intron
chr7	75099265	75099929	Intron
chr7	75100298	75100526	Intron
chr7	75104154	75104353	5' UTR
chr7	75104400	75105818	5' UTR
chr7	75112476	75114913	Promoter
chr7	75150971	75151836	Intron
chr7	75165136	75167715	3' UTR
chr7	75278767	75279018	Intron
chr7	75279103	75279488	Intron
chr7	75279621	75280702	Intron
chr7	77160606	77162202	Distal Intergenic
chr7	77181073	77183062	Intron
chr7	77288955	77291056	Intron
chr7	77411274	77411525	Distal Intergenic
chr7	77411667	77411994	Distal Intergenic
chr7	77412243	77412756	Distal Intergenic
chr7	77448729	77450253	Intron

chr7	77503747	77504235	Intron
chr7	77504300	77504644	Intron
chr7	77504822	77505027	Intron
chr7	77505296	77505782	Intron
chr7	77718750	77719688	Intron
chr7	79262452	79262667	Distal Intergenic
chr7	79262735	79262963	Distal Intergenic
chr7	79443173	79444948	Distal Intergenic
chr7	79470470	79471957	Distal Intergenic
chr7	79589143	79589541	Distal Intergenic
chr7	79589609	79589845	Distal Intergenic
chr7	79589904	79590136	Distal Intergenic
chr7	79591180	79593085	Distal Intergenic
chr7	79593234	79593433	Distal Intergenic
chr7	79637484	79639309	Distal Intergenic
chr7	79640657	79643299	Distal Intergenic
chr7	79643438	79644529	Distal Intergenic
chr7	79644606	79645034	Distal Intergenic
chr7	79654654	79655394	Distal Intergenic
chr7	79664499	79664733	Distal Intergenic
chr7	79735391	79735771	Distal Intergenic
chr7	79835708	79838363	Intron
chr7	79840551	79840793	Intron
chr7	79840846	79841354	Intron
chr7	80165003	80165577	Intron
chr7	80173611	80174937	Intron
chr7	80191954	80192610	Intron
chr7	80193023	80193249	Intron
chr7	80378243	80378532	Exon
chr7	80678122	80679579	Distal Intergenic
chr7	80680095	80680563	Distal Intergenic
chr7	80681470	80682828	Distal Intergenic
chr7	80683001	80683716	Distal Intergenic
chr7	80683768	80684411	Distal Intergenic
chr7	80684473	80687280	Distal Intergenic
chr7	80696496	80698500	Distal Intergenic
chr7	80698536	80698806	Distal Intergenic
chr7	80698895	80699426	Distal Intergenic
chr7	80699578	80703171	Distal Intergenic
chr7	80703672	80704386	Distal Intergenic
chr7	80731417	80731745	Distal Intergenic
chr7	80732191	80732392	Distal Intergenic
chr7	80732641	80735085	Distal Intergenic
chr7	80751608	80753455	Distal Intergenic

chr7	80760610	80763205	Distal Intergenic
chr7	80763522	80764445	Distal Intergenic
chr7	80768861	80769221	Distal Intergenic
chr7	81317667	81317877	Intron
chr7	81521496	81521718	Distal Intergenic
chr7	81609554	81610498	Intron
chr7	81678402	81678601	Intron
chr7	82069013	82069798	Intron
chr7	82538143	82538877	Exon
chr7	82539619	82540342	Intron
chr7	82542455	82543295	Intron
chr7	82543893	82544220	Exon
chr7	82556069	82556887	Intron
chr7	82571609	82572613	Intron
chr7	82786252	82789904	Intron
chr7	82793194	82795562	Promoter
chr7	82799954	82801212	Distal Intergenic
chr7	82811093	82812585	Distal Intergenic
chr7	82823911	82828949	Distal Intergenic
chr7	82847829	82848231	Distal Intergenic
chr7	85927989	85928223	Distal Intergenic
chr7	85969688	85970562	Distal Intergenic
chr7	86040787	86042080	Distal Intergenic
chr7	86058257	86058480	Distal Intergenic
chr7	86058838	86059800	Distal Intergenic
chr7	86285335	86285709	Intron
chr7	86293279	86293571	Intron
chr7	86294281	86294656	Intron
chr7	86355022	86357217	Intron
chr7	86414442	86415390	Intron
chr7	86415663	86416183	Exon
chr7	86417627	86417826	Intron
chr7	86430058	86430446	Intron
chr7	86433246	86433445	Intron
chr7	86481248	86481508	Intron
chr7	87004902	87006137	Exon
chr7	87025894	87026646	Intron
chr7	87026806	87027005	Intron
chr7	87174423	87175122	Intron
chr7	87204266	87204609	Intron
chr7	87305378	87305739	Intron
chr7	87308780	87312599	Intron
chr7	87320765	87321010	Intron
chr7	87331136	87331751	Intron

chr7	87331901	87332424	Intron
chr7	87332577	87332842	Intron
chr7	87332926	87337320	Intron
chr7	87342399	87342863	Promoter
chr7	87343504	87343995	Promoter
chr7	87348993	87349384	Intron
chr7	87349479	87349779	Intron
chr7	87350881	87354294	Intron
chr7	87354355	87354577	Intron
chr7	87406107	87406684	Intron
chr7	87414553	87415026	Intron
chr7	87439265	87440036	Intron
chr7	87481441	87482723	Intron
chr7	87484287	87484507	Intron
chr7	87485245	87485455	Intron
chr7	87514466	87515389	5' UTR
chr7	87515431	87515730	Intron
chr7	87520050	87520345	Intron
chr7	87545259	87547134	Distal Intergenic
chr7	87548289	87548591	Distal Intergenic
chr7	87584319	87584854	Intron
chr7	87587009	87588059	Intron
chr7	87618512	87618867	Intron
chr7	87860552	87861021	Distal Intergenic
chr7	87907316	87908161	3' UTR
chr7	87918845	87919243	Intron
chr7	87919374	87919774	Intron
chr7	87924007	87925133	Intron
chr7	87931188	87932524	Intron
chr7	87947792	87948231	Distal Intergenic
chr7	87968207	87969743	Distal Intergenic
chr7	87969972	87971308	Distal Intergenic
chr7	87972667	87973813	Distal Intergenic
chr7	87980691	87981109	Distal Intergenic
chr7	87982438	87983725	Distal Intergenic
chr7	87984279	87985023	Distal Intergenic
chr7	87985187	87985890	Distal Intergenic
chr7	88045771	88045992	Distal Intergenic
chr7	88092257	88092697	Distal Intergenic
chr7	88137656	88138317	Distal Intergenic
chr7	88327605	88328299	Distal Intergenic
chr7	88364303	88364530	Distal Intergenic
chr7	88364860	88365059	Distal Intergenic
chr7	88455705	88456779	Intron

chr7	88464272	88465566	Intron
chr7	88933642	88934444	Intron
chr7	89020176	89020552	Distal Intergenic
chr7	89063232	89063610	Distal Intergenic
chr7	89134405	89134839	Distal Intergenic
chr7	89136215	89136693	Distal Intergenic
chr7	89136827	89137242	Distal Intergenic
chr7	89137447	89138001	Distal Intergenic
chr7	89140385	89141006	Distal Intergenic
chr7	89141617	89141830	Distal Intergenic
chr7	89143759	89144975	Distal Intergenic
chr7	89152237	89152485	Distal Intergenic
chr7	89152599	89153111	Distal Intergenic
chr7	89161609	89163735	Distal Intergenic
chr7	89172996	89175509	Distal Intergenic
chr7	89175820	89176074	Distal Intergenic
chr7	89201426	89202029	Distal Intergenic
chr7	89212724	89216228	Distal Intergenic
chr7	89216436	89217087	Distal Intergenic
chr7	89612782	89613052	Distal Intergenic
chr7	89613123	89613375	Distal Intergenic
chr7	89628069	89628330	Distal Intergenic
chr7	89797158	89797393	Promoter
chr7	89813397	89813600	Intron
chr7	89813648	89813968	Intron
chr7	89814130	89814333	Intron
chr7	89846186	89846936	Intron
chr7	90043157	90043757	3' UTR
chr7	90043824	90044145	3' UTR
chr7	90204786	90205516	Intron
chr7	90205565	90205771	Intron
chr7	90455012	90455249	Intron
chr7	91031719	91032568	Distal Intergenic
chr7	91032901	91033290	Distal Intergenic
chr7	91111892	91112091	Distal Intergenic
chr7	91177996	91178201	Distal Intergenic
chr7	91236637	91237284	Distal Intergenic
chr7	91266427	91266684	Distal Intergenic
chr7	91284072	91285287	Distal Intergenic
chr7	91290420	91290648	Distal Intergenic
chr7	91332860	91334071	Distal Intergenic
chr7	91394577	91394822	Distal Intergenic
chr7	91394907	91395901	Distal Intergenic
chr7	91613560	91615824	Intron

chr7	91669404	91670575	3' UTR
chr7	91670937	91671305	3' UTR
chr7	91695452	91695689	Intron
chr7	91827765	91827965	Downstream
chr7	93635484	93638098	Distal Intergenic
chr7	95348008	95348838	Distal Intergenic
chr7	95419830	95420475	Intron
chr7	95573887	95574198	Intron
chr7	95574248	95574909	Intron
chr7	95578429	95578717	Intron
chr7	95578873	95579550	Intron
chr7	95580021	95580305	Intron
chr7	95614394	95614844	Intron
chr7	95616236	95618043	Exon
chr7	95623974	95624308	Intron
chr7	96084033	96084255	Distal Intergenic
chr7	96326713	96327316	Intron
chr7	96327356	96327621	Intron
chr7	96590128	96590632	Distal Intergenic
chr7	96612386	96612663	Intron
chr7	96612775	96613134	Intron
chr7	96665371	96665953	Distal Intergenic
chr7	96666291	96667001	Distal Intergenic
chr7	96667322	96668270	Distal Intergenic
chr7	96671864	96672766	Distal Intergenic
chr7	96689014	96690448	Distal Intergenic
chr7	96707605	96710320	Distal Intergenic
chr7	96712728	96712945	Distal Intergenic
chr7	96728627	96730356	Distal Intergenic
chr7	96754626	96754865	Intron
chr7	96759272	96760132	Intron
chr7	96824861	96825090	Distal Intergenic
chr7	96887246	96887981	Distal Intergenic
chr7	96936283	96936488	Distal Intergenic
chr7	96957805	96958533	Distal Intergenic
chr7	97089481	97089727	Distal Intergenic
chr7	97128091	97128676	Distal Intergenic
chr7	97129051	97129253	Distal Intergenic
chr7	97154963	97156397	Distal Intergenic
chr7	97187902	97188190	Distal Intergenic
chr7	97190114	97190528	Distal Intergenic
chr7	97190816	97191102	Distal Intergenic
chr7	97208049	97208637	Distal Intergenic
chr7	97244013	97244680	Distal Intergenic

chr7	97244798	97245068	Distal Intergenic
chr7	97245171	97245984	Distal Intergenic
chr7	97278043	97278406	Distal Intergenic
chr7	97279194	97280798	Distal Intergenic
chr7	99345985	99347295	Distal Intergenic
chr7	99351106	99351518	Distal Intergenic
chr7	99351569	99351772	Downstream
chr7	99578856	99580016	Promoter
chr7	99627858	99628122	Exon
chr7	99790473	99790738	Intron
chr7	99815325	99815804	Promoter
chr7	99816914	99818440	Promoter
chr7	99847073	99847488	Intron
chr7	99851615	99852202	Intron
chr7	99885612	99887968	Distal Intergenic
chr7	99888015	99890001	Distal Intergenic
chr7	99923552	99925042	Intron
chr7	99945182	99946338	Intron
chr7	99971614	99972230	Promoter
chr7	99974303	99975267	Intron
chr7	99985451	99986561	Intron
chr7	99986662	99986867	Intron
chr7	100021859	100023620	5' UTR
chr7	100023706	100023921	Intron
chr7	100100090	100100372	Distal Intergenic
chr7	100101154	100101526	Distal Intergenic
chr7	103658440	103659315	Distal Intergenic
chr7	103670333	103670578	Distal Intergenic
chr7	103670994	103671201	Distal Intergenic
chr7	103710760	103711700	Distal Intergenic
chr7	103723771	103724026	Distal Intergenic
chr7	103856204	103856447	Distal Intergenic
chr7	104520463	104520671	Intron
chr7	104659741	104660201	Intron
chr7	104728729	104729603	Intron
chr7	104793445	104794215	Intron
chr7	104849202	104849483	Intron
chr7	104849652	104850239	Intron
chr7	104857635	104858004	Intron
chr7	104919376	104919575	Intron
chr7	104937054	104937259	Intron
chr7	104937764	104938078	5' UTR
chr7	104938495	104938772	Promoter
chr7	104940009	104941684	Promoter

chr7	104978479	104979936	Intron
chr7	105264513	105264838	Promoter
chr7	105265234	105265960	Promoter
chr7	105319746	105319945	Promoter
chr7	105326993	105327471	Intron
chr7	105452385	105452871	Intron
chr7	105457200	105457435	Intron
chr7	105457756	105457975	Intron
chr7	105537241	105537537	Intron
chr7	105537713	105537912	Intron
chr7	105539213	105539552	Intron
chr7	105539634	105541055	Intron
chr7	105564043	105564634	Intron
chr7	105640463	105640798	Intron
chr7	105706920	105707944	Distal Intergenic
chr7	105708121	105708348	Distal Intergenic
chr7	105708526	105711176	Distal Intergenic
chr7	105736370	105736656	Intron
chr7	105804890	105805171	Distal Intergenic
chr7	105841239	105842090	Distal Intergenic
chr7	105842128	105842327	Distal Intergenic
chr7	105850467	105851303	Distal Intergenic
chr7	106116151	106117927	Distal Intergenic
chr7	106146263	106147270	Intron
chr7	106172095	106172936	Intron
chr7	106175059	106176642	Intron
chr7	106233598	106233841	Intron
chr7	106417731	106418733	Distal Intergenic
chr7	106424640	106426128	Distal Intergenic
chr7	106451098	106451329	Distal Intergenic
chr7	106451955	106452183	Distal Intergenic
chr7	106452522	106453945	Distal Intergenic
chr7	106467242	106468234	Distal Intergenic
chr7	106469422	106469703	Distal Intergenic
chr7	106470261	106471768	Distal Intergenic
chr7	106542428	106542664	Intron
chr7	106556301	106556500	Distal Intergenic
chr7	106607660	106608534	Distal Intergenic
chr7	106608675	106608882	Distal Intergenic
chr7	106614633	106614995	Distal Intergenic
chr7	106622595	106623178	Distal Intergenic
chr7	106631774	106632032	Distal Intergenic
chr7	106974450	106975161	Intron
chr7	106975369	106975636	Intron

chr7	106975803	106976812	Intron
chr7	107007007	107007206	Intron
chr7	107095390	107095606	Intron
chr7	107123109	107124208	Intron
chr7	107127421	107128461	Intron
chr7	107578386	107578711	Intron
chr7	107578859	107580062	Intron
chr7	107613134	107614772	Exon
chr7	107739286	107740013	Intron
chr7	107826484	107827150	Intron
chr7	107827278	107828702	Intron
chr7	107988620	107988835	Intron
chr7	108080792	108081059	Intron
chr7	108189708	108189969	Distal Intergenic
chr7	108192460	108192930	Distal Intergenic
chr7	108193004	108193203	Distal Intergenic
chr7	108264758	108265324	Distal Intergenic
chr7	108834132	108835248	Distal Intergenic
chr7	108985263	108985596	Distal Intergenic
chr7	108985675	108985877	Distal Intergenic
chr7	109053672	109054628	Distal Intergenic
chr7	109081294	109082290	Distal Intergenic
chr7	109113589	109114174	Distal Intergenic
chr7	109117438	109117645	Distal Intergenic
chr7	109117968	109118761	Distal Intergenic
chr7	109119200	109120763	Distal Intergenic
chr7	109214027	109214293	Distal Intergenic
chr7	109371504	109371816	Distal Intergenic
chr7	109371990	109373245	Distal Intergenic
chr7	109386989	109388167	Distal Intergenic
chr7	109398626	109399111	Distal Intergenic
chr7	109399461	109399989	Distal Intergenic
chr7	109400820	109401189	Distal Intergenic
chr7	109537601	109538024	Distal Intergenic
chr7	109544031	109545100	Distal Intergenic
chr7	109546878	109548958	Distal Intergenic
chr7	109590279	109591082	Distal Intergenic
chr7	109627197	109628917	Distal Intergenic
chr7	111366323	111368181	3' UTR
chr7	111368316	111368603	3' UTR
chr7	112884410	112884609	Distal Intergenic
chr7	112885201	112885558	Distal Intergenic
chr7	112885861	112886084	Distal Intergenic
chr7	112886825	112887822	Distal Intergenic

chr7	112892348	112892555	Distal Intergenic
chr7	112892645	112893096	Distal Intergenic
chr7	113677960	113678205	Distal Intergenic
chr7	113694732	113694944	Distal Intergenic
chr7	113700063	113701195	Distal Intergenic
chr7	114825103	114826519	Distal Intergenic
chr7	114828715	114829335	Distal Intergenic
chr7	114829372	114829643	Distal Intergenic
chr7	114829880	114830122	Distal Intergenic
chr7	114831147	114831700	Distal Intergenic
chr7	114869586	114870383	Distal Intergenic
chr7	116215497	116215874	Distal Intergenic
chr7	116279072	116279771	Distal Intergenic
chr7	116280591	116281752	Distal Intergenic
chr7	116284351	116284669	Distal Intergenic
chr7	116284805	116285064	Distal Intergenic
chr7	116285117	116286833	Distal Intergenic
chr7	116286972	116287634	Distal Intergenic
chr7	116287897	116288122	Distal Intergenic
chr7	116288656	116290287	Distal Intergenic
chr7	116330966	116331323	Intron
chr7	116575160	116575463	Distal Intergenic
chr7	116575500	116575763	Distal Intergenic
chr7	116614503	116614799	Intron
chr7	116661939	116662285	Intron
chr7	116662658	116663192	Intron
chr7	116663369	116663935	Intron
chr7	116664256	116664529	Intron
chr7	116664834	116665448	Intron
chr7	116665684	116666078	Intron
chr7	116666827	116667035	Intron
chr7	116690022	116690236	Intron
chr7	116690834	116691033	Intron
chr7	116705037	116705279	Intron
chr7	116705679	116705994	Intron
chr7	116711655	116713162	Exon
chr7	116722045	116723599	Intron
chr7	116752837	116753943	Intron
chr7	116764919	116765233	Intron
chr7	116773102	116775888	Exon
chr7	116779017	116779232	Intron
chr7	116779290	116781875	Intron
chr7	117130006	117130415	Intron
chr7	117152007	117152226	Intron

chr7	117152324	117152546	Intron
chr7	117161934	117162988	Intron
chr7	117203670	117204964	Exon
chr7	117205181	117206344	Intron
chr7	117252430	117253266	Intron
chr7	117266859	117267223	Intron
chr7	117267270	117267469	Intron
chr7	117307897	117308291	3' UTR
chr7	117380221	117380802	Intron
chr7	117380868	117382015	Intron
chr7	117431258	117433900	Exon
chr7	117469053	117470522	Intron
chr7	117478077	117479150	Intron
chr7	117480717	117481215	Intron
chr7	117481290	117481511	Intron
chr7	117574557	117575799	Distal Intergenic
chr7	117672300	117673293	Distal Intergenic
chr7	117682366	117683398	Distal Intergenic
chr7	117683679	117683945	Distal Intergenic
chr7	117698581	117698838	Distal Intergenic
chr7	117757393	117757762	Distal Intergenic
chr7	117812367	117812570	Distal Intergenic
chr7	117814892	117815259	Distal Intergenic
chr7	117912830	117915134	Distal Intergenic
chr7	117916540	117918047	Distal Intergenic
chr7	117923340	117924842	Distal Intergenic
chr7	117924882	117925385	Distal Intergenic
chr7	117928169	117929000	Distal Intergenic
chr7	117929512	117929747	Distal Intergenic
chr7	117930314	117930872	Distal Intergenic
chr7	117930983	117931297	Distal Intergenic
chr7	117939223	117939956	Distal Intergenic
chr7	117940423	117941883	Distal Intergenic
chr7	117950785	117951069	Distal Intergenic
chr7	117974624	117975458	Distal Intergenic
chr7	117994964	117995328	Distal Intergenic
chr7	118084941	118085228	Distal Intergenic
chr7	118225517	118226231	Distal Intergenic
chr7	118226859	118227391	Distal Intergenic
chr7	118265810	118266925	Distal Intergenic
chr7	118299019	118300732	Distal Intergenic
chr7	118300798	118301848	Distal Intergenic
chr7	118388704	118389488	Distal Intergenic
chr7	118845333	118846192	Distal Intergenic

chr7	118859927	118861646	Distal Intergenic
chr7	118862323	118862879	Distal Intergenic
chr7	118866974	118868421	Distal Intergenic
chr7	118868633	118868873	Distal Intergenic
chr7	118869007	118869672	Distal Intergenic
chr7	118914899	118915532	Distal Intergenic
chr7	119120022	119120299	Distal Intergenic
chr7	119120351	119120608	Distal Intergenic
chr7	119120719	119120968	Distal Intergenic
chr7	119167035	119167390	Distal Intergenic
chr7	119167460	119168419	Distal Intergenic
chr7	119174050	119174269	Distal Intergenic
chr7	119284184	119284675	Distal Intergenic
chr7	119284747	119284984	Distal Intergenic
chr7	119286903	119289003	Distal Intergenic
chr7	119300847	119301240	Distal Intergenic
chr7	119331355	119331987	Distal Intergenic
chr7	119345455	119345840	Distal Intergenic
chr7	119345903	119347109	Distal Intergenic
chr7	119348237	119348653	Distal Intergenic
chr7	119370179	119370881	Distal Intergenic
chr7	119371044	119371342	Distal Intergenic
chr7	119389787	119390187	Distal Intergenic
chr7	119420286	119420910	Distal Intergenic
chr7	119441943	119444099	Distal Intergenic
chr7	119447970	119448310	Distal Intergenic
chr7	119479628	119479841	Distal Intergenic
chr7	119519292	119519496	Distal Intergenic
chr7	119519696	119519907	Distal Intergenic
chr7	119551994	119552298	Distal Intergenic
chr7	119560497	119560752	Distal Intergenic
chr7	119596984	119597439	Distal Intergenic
chr7	119597610	119598012	Distal Intergenic
chr7	119598063	119598989	Distal Intergenic
chr7	119599039	119600031	Distal Intergenic
chr7	119610763	119612267	Distal Intergenic
chr7	119642798	119643006	Distal Intergenic
chr7	119643263	119643561	Distal Intergenic
chr7	119806323	119806927	Distal Intergenic
chr7	119840537	119840999	Distal Intergenic
chr7	120050779	120051262	Intron
chr7	120147703	120147968	Intron
chr7	120148899	120149871	Intron
chr7	120152280	120153057	Intron

chr7	120190896	120192544	Intron
chr7	120207189	120207593	Intron
chr7	120249849	120250481	Intron
chr7	120250690	120251146	Intron
chr7	120277295	120277566	Intron
chr7	120289956	120290276	Intron
chr7	120290347	120293740	Intron
chr7	120294476	120295509	Intron
chr7	120296614	120296986	Intron
chr7	120311983	120312675	Intron
chr7	120329492	120330191	Intron
chr7	120377328	120377975	Intron
chr7	120378819	120379018	Intron
chr7	120379156	120380197	Intron
chr7	120385847	120386188	Exon
chr7	120392849	120393103	Distal Intergenic
chr7	120403425	120404155	Distal Intergenic
chr7	120644324	120644654	Intron
chr7	120645384	120646418	Intron
chr7	120646523	120646814	Intron
chr7	120686862	120687064	Exon
chr7	120687158	120687733	Intron
chr7	121207288	121207564	Distal Intergenic
chr7	121314578	121315886	Distal Intergenic
chr7	121319739	121320393	Distal Intergenic
chr7	121324528	121325159	Distal Intergenic
chr7	121419188	121419387	Distal Intergenic
chr7	122347545	122348177	Intron
chr7	122452559	122453158	Intron
chr7	122453247	122453446	Intron
chr7	122453668	122455616	Intron
chr7	122465867	122467302	Intron
chr7	122718266	122719508	Distal Intergenic
chr7	122723477	122724250	Distal Intergenic
chr7	122724395	122724823	Distal Intergenic
chr7	122726502	122726773	Distal Intergenic
chr7	123236848	123237249	Distal Intergenic
chr7	123243592	123243901	Intron
chr7	123332974	123334123	Intron
chr7	123336189	123337498	Exon
chr7	123340857	123341458	Intron
chr7	123341623	123342090	Intron
chr7	123374374	123375235	Intron
chr7	123477271	123477673	Distal Intergenic

chr7	123477757	123477956	Distal Intergenic
chr7	123587354	123587837	5' UTR
chr7	123587913	123588477	Intron
chr7	124400273	124400610	Intron
chr7	124400647	124400877	Intron
chr7	125248602	125248946	Distal Intergenic
chr7	125259032	125259283	Distal Intergenic
chr7	125259507	125260175	Distal Intergenic
chr7	125272776	125273088	Distal Intergenic
chr7	125273289	125273932	Distal Intergenic
chr7	125274087	125274432	Distal Intergenic
chr7	125385996	125386795	Distal Intergenic
chr7	125386843	125387089	Distal Intergenic
chr7	125395308	125395672	Distal Intergenic
chr7	125395909	125396141	Distal Intergenic
chr7	125420842	125421399	Distal Intergenic
chr7	125606630	125606829	Distal Intergenic
chr7	125623559	125625481	Distal Intergenic
chr7	125625618	125626051	Distal Intergenic
chr7	125627320	125627532	Distal Intergenic
chr7	125630032	125630284	Distal Intergenic
chr7	125630323	125630522	Distal Intergenic
chr7	125630562	125630949	Distal Intergenic
chr7	125631040	125632585	Distal Intergenic
chr7	125634938	125635473	Distal Intergenic
chr7	125642135	125643372	Distal Intergenic
chr7	125651256	125652221	Distal Intergenic
chr7	125712315	125712651	Distal Intergenic
chr7	126038797	126039669	Distal Intergenic
chr7	126072925	126073225	Distal Intergenic
chr7	126073493	126073915	Distal Intergenic
chr7	126078764	126079045	3' UTR
chr7	126079205	126079560	3' UTR
chr7	126743030	126743396	Intron
chr7	127003797	127005872	Distal Intergenic
chr7	127102846	127103766	Distal Intergenic
chr7	127797811	127798569	Distal Intergenic
chr7	127981624	127982102	Intron
chr7	127982327	127982736	Intron
chr7	127982785	127982984	Promoter
chr7	128008182	128010842	Distal Intergenic
chr7	128012517	128013432	Distal Intergenic
chr7	128013596	128013819	Distal Intergenic
chr7	128845879	128846101	Promoter

chr7	128853925	128855499	Distal Intergenic
chr7	129158733	129159262	Distal Intergenic
chr7	129209565	129210549	Distal Intergenic
chr7	129210681	129210923	Distal Intergenic
chr7	129302235	129302670	Intron
chr7	130113970	130114307	Distal Intergenic
chr7	130251822	130252021	Intron
chr7	130252126	130252727	Intron
chr7	130253459	130254511	Intron
chr7	130264308	130264684	Intron
chr7	130267424	130267623	Intron
chr7	130554772	130555084	Downstream
chr7	132526304	132527337	Intron
chr7	132642271	132642517	Intron
chr7	132642700	132643727	Intron
chr7	132678021	132680294	Intron
chr7	132682470	132682705	Intron
chr7	132682794	132683302	Intron
chr7	132849671	132850852	Distal Intergenic
chr7	132859790	132860733	Distal Intergenic
chr7	133935099	133935320	Intron
chr7	133935402	133936020	Intron
chr7	133936118	133936518	Intron
chr7	134119659	134119858	Distal Intergenic
chr7	134119920	134120233	Distal Intergenic
chr7	134321263	134321818	Distal Intergenic
chr7	134339099	134339300	Intron
chr7	134339348	134339576	Intron
chr7	134375718	134376530	Distal Intergenic
chr7	134613767	134615357	Promoter
chr7	134622041	134622490	Intron
chr7	134655537	134655799	Distal Intergenic
chr7	134656203	134656410	Distal Intergenic
chr7	134704849	134706978	Intron
chr7	134707106	134707417	Intron
chr7	134889066	134889622	5' UTR
chr7	135018977	135019586	Distal Intergenic
chr7	135056170	135056721	Intron
chr7	135168228	135169027	Intron
chr7	135169547	135169746	Intron
chr7	135232617	135232844	Distal Intergenic
chr7	135232933	135233234	Distal Intergenic
chr7	135381174	135381584	Intron
chr7	135461838	135464913	Distal Intergenic

chr7	135506364	135508802	Distal Intergenic
chr7	135540476	135541964	Distal Intergenic
chr7	135545871	135547308	Distal Intergenic
chr7	135582912	135583535	Distal Intergenic
chr7	135603360	135603622	Distal Intergenic
chr7	135603965	135604170	Distal Intergenic
chr7	135667077	135668244	Distal Intergenic
chr7	135668373	135668744	Distal Intergenic
chr7	135669939	135670468	Distal Intergenic
chr7	135672306	135672664	Distal Intergenic
chr7	135672889	135673191	Distal Intergenic
chr7	135673294	135673493	Distal Intergenic
chr7	135675425	135675671	Distal Intergenic
chr7	135676222	135676559	Distal Intergenic
chr7	135696866	135697313	Distal Intergenic
chr7	135697358	135698242	Distal Intergenic
chr7	135765406	135767067	Distal Intergenic
chr7	135815232	135815459	Distal Intergenic
chr7	135815646	135815989	Distal Intergenic
chr7	135816154	135816369	Distal Intergenic
chr7	136794923	136795914	Intron
chr7	136796458	136796657	Intron
chr7	136807355	136807600	Intron
chr7	136807977	136808183	Intron
chr7	136809253	136810052	Intron
chr7	136906505	136908212	Distal Intergenic
chr7	136908295	136908755	Distal Intergenic
chr7	136990434	136990808	Intron
chr7	136990859	136991361	Intron
chr7	137117759	137118151	Intron
chr7	137118292	137119041	Intron
chr7	137175965	137176180	Intron
chr7	137331893	137332305	Intron
chr7	137356099	137358183	Intron
chr7	137358498	137360377	Intron
chr7	137406633	137407673	Intron
chr7	137408209	137408634	Intron
chr7	137432345	137432820	Intron
chr7	137450955	137451406	Intron
chr7	137451552	137451891	Intron
chr7	137452502	137452971	Intron
chr7	137483276	137485451	Intron
chr7	137718165	137718769	Intron
chr7	137767692	137768012	Intron

chr7	137788492	137789593	Intron
chr7	137789659	137790214	Exon
chr7	137790372	137791153	Intron
chr7	137804620	137804915	Distal Intergenic
chr7	137813804	137814010	Distal Intergenic
chr7	137814113	137815443	Distal Intergenic
chr7	137816249	137817579	Distal Intergenic
chr7	137840194	137840508	Distal Intergenic
chr7	138085639	138087223	Distal Intergenic
chr7	138089220	138090921	Distal Intergenic
chr7	138091529	138091981	Distal Intergenic
chr7	138092026	138093234	Distal Intergenic
chr7	138093688	138095285	Distal Intergenic
chr7	138108197	138108508	Distal Intergenic
chr7	138108585	138109305	Distal Intergenic
chr7	138120482	138120806	Distal Intergenic
chr7	138120875	138121423	Distal Intergenic
chr7	138121544	138124357	Distal Intergenic
chr7	138125653	138125963	Distal Intergenic
chr7	138126173	138126525	Distal Intergenic
chr7	138379025	138379795	Distal Intergenic
chr7	138505112	138505682	Distal Intergenic
chr7	138704296	138704544	Distal Intergenic
chr7	138723526	138723795	Distal Intergenic
chr7	138724014	138724343	Distal Intergenic
chr7	138725390	138726331	Downstream
chr7	139423257	139423672	Intron
chr7	139425846	139426070	Intron
chr7	139426145	139426556	Intron
chr7	139481976	139483026	5' UTR
chr7	139508202	139508401	Intron
chr7	139564634	139565050	Intron
chr7	139926791	139929331	Distal Intergenic
chr7	139982139	139982894	Distal Intergenic
chr7	139982974	139983173	Distal Intergenic
chr7	139993965	139995735	Distal Intergenic
chr7	140003506	140004106	Distal Intergenic
chr7	140004203	140004420	Distal Intergenic
chr7	140044150	140044349	Intron
chr7	140123526	140123946	Intron
chr7	140197428	140198821	Distal Intergenic
chr7	140198939	140199228	Distal Intergenic
chr7	140289979	140291215	Intron
chr7	140304191	140305127	Intron

chr7	140313245	140313685	Intron
chr7	140313732	140314343	Intron
chr7	140315664	140315891	Intron
chr7	140710440	140710813	5' UTR
chr7	140954355	140954582	Intron
chr7	140974634	140974843	Intron
chr7	140985434	140985844	Intron
chr7	141061578	141062022	Intron
chr7	141306160	141306866	Intron
chr7	141422459	141422681	Intron
chr7	141422727	141423927	3' UTR
chr7	141428523	141429315	Intron
chr7	141452576	141453105	Distal Intergenic
chr7	141454876	141455191	Distal Intergenic
chr7	141455387	141455586	Distal Intergenic
chr7	142436877	142437560	Intron
chr7	142437596	142437796	Intron
chr7	142796648	142797497	Distal Intergenic
chr7	142801612	142801954	Distal Intergenic
chr7	142843625	142844825	Distal Intergenic
chr7	142845063	142845775	Distal Intergenic
chr7	142845907	142846271	Distal Intergenic
chr7	142850938	142851137	Distal Intergenic
chr7	142851181	142851421	Distal Intergenic
chr7	144629377	144630855	Distal Intergenic
chr7	146098673	146099035	Intron
chr7	146431489	146434237	Intron
chr7	146438335	146438561	Intron
chr7	146438659	146438920	Intron
chr7	146439136	146439539	Intron
chr7	146443442	146444200	Intron
chr7	146466109	146466340	Intron
chr7	146466455	146467113	Intron
chr7	146469842	146470325	Intron
chr7	146470521	146470901	Intron
chr7	146471220	146471575	Exon
chr7	146568855	146569415	Intron
chr7	146600015	146601908	Intron
chr7	146702097	146702324	Intron
chr7	146702367	146702607	Intron
chr7	146717524	146717735	Intron
chr7	147155945	147156752	Intron
chr7	147156817	147159025	Intron
chr7	147179440	147179939	Intron

chr7	147180182	147180548	Intron
chr7	148007681	148007883	Intron
chr7	148017626	148017843	Intron
chr7	148020365	148020951	Intron
chr7	148021152	148021594	Intron
chr7	148141729	148142206	Distal Intergenic
chr7	148159115	148160405	Distal Intergenic
chr7	148167812	148168357	Distal Intergenic
chr7	148172880	148173149	Distal Intergenic
chr7	148173246	148173995	Distal Intergenic
chr7	148195692	148195894	Distal Intergenic
chr7	148196397	148196891	Distal Intergenic
chr7	148264322	148266562	Distal Intergenic
chr7	148353366	148353667	Distal Intergenic
chr7	148353733	148354631	Distal Intergenic
chr7	148422760	148424430	Intron
chr7	148513722	148514147	3' UTR
chr7	148517947	148518178	Intron
chr7	148524895	148525250	Intron
chr7	148525450	148526073	Exon
chr7	148526192	148526417	Intron
chr7	148558245	148558527	Intron
chr7	148653768	148654777	Distal Intergenic
chr7	148715754	148715953	Intron
chr7	148771382	148771628	5' UTR
chr7	149245052	149245254	Exon
chr7	149245963	149246373	Exon
chr7	149269889	149270178	Intron
chr7	149270379	149272102	Intron
chr7	149526281	149526488	Intron
chr7	149548662	149549229	Intron
chr7	149549292	149549543	Intron
chr7	149561892	149562109	3' UTR
chr7	149573540	149573759	Intron
chr7	149579085	149579563	Exon
chr7	149619298	149621934	Distal Intergenic
chr7	149621996	149623162	Distal Intergenic
chr7	150210918	150211869	Promoter
chr7	150248142	150248873	Distal Intergenic
chr7	150535872	150536356	Intron
chr7	150536442	150536759	Intron
chr7	150542686	150543156	Intron
chr7	150810567	150811325	Promoter
chr7	150815663	150816021	Exon

chr7	150817401	150817854	Intron
chr7	150876710	150877788	Intron
chr7	150923917	150924800	Promoter
chr7	150925153	150925399	Promoter
chr7	150971157	150971437	Intron
chr7	151017499	151017775	Distal Intergenic
chr7	151017854	151018358	Distal Intergenic
chr7	151223889	151224296	Distal Intergenic
chr7	151655311	151655599	Intron
chr7	151749433	151749790	Intron
chr7	151749971	151750488	Intron
chr7	151752659	151753019	Intron
chr7	151753090	151753580	Intron
chr7	151753619	151753823	Intron
chr7	151798484	151798846	Intron
chr7	151799045	151799272	Intron
chr7	151869963	151870739	Intron
chr7	151870970	151871603	Exon
chr7	151933337	151933967	Intron
chr7	152126050	152126538	Intron
chr7	152126817	152127551	Intron
chr7	152149497	152151264	Distal Intergenic
chr7	152156312	152157382	Distal Intergenic
chr7	152157820	152158764	Distal Intergenic
chr7	152158887	152159261	Distal Intergenic
chr7	152292155	152292544	Distal Intergenic
chr7	152297274	152297546	Distal Intergenic
chr7	152470144	152470440	Intron
chr7	152470638	152471085	Intron
chr7	152516307	152516875	Intron
chr8	3077171	3078161	Intron
chr8	3393110	3393371	Intron
chr8	3407490	3409835	Intron
chr8	3411222	3413415	Intron
chr8	3417828	3418482	Intron
chr8	3424385	3424784	Intron
chr8	3424869	3426824	Intron
chr8	3428598	3429087	Intron
chr8	3429126	3430559	Intron
chr8	3515169	3516495	Intron
chr8	3631200	3631528	Intron
chr8	3647213	3648233	Intron
chr8	3676484	3677368	Intron
chr8	3991344	3991836	Intron

chr8	4013112	4015295	Intron
chr8	4165349	4165904	Intron
chr8	4252035	4252686	Intron
chr8	4259440	4259803	Intron
chr8	4275334	4275963	Intron
chr8	4276066	4276324	Intron
chr8	4323872	4324248	Intron
chr8	4324493	4324866	Intron
chr8	4612013	4612266	Intron
chr8	4612658	4613568	Intron
chr8	4677953	4678413	Intron
chr8	4678467	4679202	Intron
chr8	5283162	5284384	Distal Intergenic
chr8	7086316	7086989	Distal Intergenic
chr8	8109968	8110423	Distal Intergenic
chr8	8186553	8188354	Intron
chr8	8273426	8275417	Distal Intergenic
chr8	8275563	8275783	Distal Intergenic
chr8	8487512	8488132	Distal Intergenic
chr8	8602941	8603429	Distal Intergenic
chr8	8650071	8650289	Intron
chr8	9515117	9516988	Intron
chr8	9810738	9811330	Distal Intergenic
chr8	10547517	10547745	Intron
chr8	10574856	10576952	Distal Intergenic
chr8	10589073	10589669	Intron
chr8	10589751	10590553	Intron
chr8	10629702	10630247	Intron
chr8	10646751	10646950	Intron
chr8	10652942	10654204	Intron
chr8	10664934	10665282	Intron
chr8	10855791	10856062	Intron
chr8	10930236	10930593	Intron
chr8	10930668	10930990	Intron
chr8	10935108	10935848	Intron
chr8	10977401	10978516	Intron
chr8	10982803	10983110	Intron
chr8	10990687	10991580	Intron
chr8	10993255	10993956	Intron
chr8	10994363	10994859	Intron
chr8	10997253	10997527	Intron
chr8	11259659	11259918	Intron
chr8	11263763	11264402	Intron
chr8	11322558	11323496	Promoter

chr8	11346043	11346903	Distal Intergenic
chr8	11457548	11457756	Distal Intergenic
chr8	11476576	11478061	Distal Intergenic
chr8	11497501	11497922	Distal Intergenic
chr8	11550368	11550592	Intron
chr8	11632475	11634797	Intron
chr8	11635918	11636279	Intron
chr8	12512567	12513367	Intron
chr8	12516144	12517476	5' UTR
chr8	12537464	12538250	Distal Intergenic
chr8	12544845	12545455	Distal Intergenic
chr8	12710503	12710797	Distal Intergenic
chr8	12730503	12731691	Distal Intergenic
chr8	12756020	12756226	Distal Intergenic
chr8	12756376	12756936	Distal Intergenic
chr8	12778858	12780282	Distal Intergenic
chr8	12996495	12997087	Intron
chr8	13105757	13105976	Intron
chr8	13106152	13106601	Intron
chr8	13139305	13140519	Intron
chr8	13144788	13145002	Intron
chr8	13159447	13159902	Intron
chr8	13160098	13160315	Intron
chr8	13339171	13339730	Intron
chr8	13372709	13373115	Promoter
chr8	13491860	13492928	Distal Intergenic
chr8	13597464	13597965	Distal Intergenic
chr8	13624179	13625636	Distal Intergenic
chr8	13664669	13665295	Distal Intergenic
chr8	13667886	13669143	Distal Intergenic
chr8	13694881	13696397	Distal Intergenic
chr8	13758188	13758567	Distal Intergenic
chr8	13869449	13869650	Distal Intergenic
chr8	14321455	14321998	Intron
chr8	14880571	14882068	Intron
chr8	14888528	14888770	Intron
chr8	14913884	14914083	Intron
chr8	14917111	14917868	Intron
chr8	17593906	17595431	Intron
chr8	17599638	17601346	Exon
chr8	18595502	18595887	Intron
chr8	18608972	18609432	Intron
chr8	18619098	18619554	Intron
chr8	18696034	18696677	Intron

chr8	18698698	18699951	Intron
chr8	18768172	18768527	Intron
chr8	18846641	18846875	Intron
chr8	19163239	19165075	Distal Intergenic
chr8	19193979	19194238	Intron
chr8	19194279	19194953	Intron
chr8	19784662	19785158	Distal Intergenic
chr8	19785358	19785769	Distal Intergenic
chr8	19892108	19892943	Distal Intergenic
chr8	20019260	20020367	Intron
chr8	22044235	22044511	Intron
chr8	22044564	22044800	Intron
chr8	22057110	22057320	Intron
chr8	22057493	22058030	Intron
chr8	22869327	22870583	Intron
chr8	23303539	23304401	Intron
chr8	23325033	23325589	Distal Intergenic
chr8	23325778	23326102	Distal Intergenic
chr8	23374689	23375896	Distal Intergenic
chr8	23587460	23588627	Distal Intergenic
chr8	23590756	23591104	Distal Intergenic
chr8	23591339	23591927	Distal Intergenic
chr8	23601703	23602587	Distal Intergenic
chr8	23803939	23804200	Distal Intergenic
chr8	23816456	23816733	Distal Intergenic
chr8	23864702	23866122	Distal Intergenic
chr8	23867429	23868021	Distal Intergenic
chr8	23917569	23918125	Distal Intergenic
chr8	23918166	23918406	Distal Intergenic
chr8	23983766	23983983	Distal Intergenic
chr8	23991229	23994151	Distal Intergenic
chr8	24118644	24121475	Distal Intergenic
chr8	24126362	24126859	Distal Intergenic
chr8	24140811	24141723	Distal Intergenic
chr8	24254655	24255030	Exon
chr8	24318568	24318848	Intron
chr8	24366938	24367518	3' UTR
chr8	24499111	24499507	Distal Intergenic
chr8	24577152	24577363	Distal Intergenic
chr8	24595850	24597189	Distal Intergenic
chr8	24626722	24627600	Distal Intergenic
chr8	24664205	24665165	Distal Intergenic
chr8	24835373	24835663	Distal Intergenic
chr8	25469152	25469551	Intron

chr8	25469837	25470148	Intron
chr8	25475033	25476155	Intron
chr8	25510086	25510417	Intron
chr8	25515802	25516045	Intron
chr8	25529472	25532120	Intron
chr8	25540597	25542676	Intron
chr8	25548024	25549441	Intron
chr8	25576701	25578030	Intron
chr8	25629053	25629890	Intron
chr8	26085442	26086853	Intron
chr8	26101314	26101586	Intron
chr8	26120285	26121351	Intron
chr8	26125878	26126175	Intron
chr8	26126376	26126896	Intron
chr8	26177828	26178302	Intron
chr8	26178621	26179714	Intron
chr8	26180304	26180592	Intron
chr8	26211523	26212574	Exon
chr8	26532215	26532641	Distal Intergenic
chr8	26548740	26549025	Distal Intergenic
chr8	26576277	26576605	Distal Intergenic
chr8	26579217	26579858	Distal Intergenic
chr8	26707122	26708023	Intron
chr8	26708100	26708300	Intron
chr8	26711773	26711992	Intron
chr8	26712122	26712580	Intron
chr8	26712699	26713380	Intron
chr8	26722493	26723626	Promoter
chr8	26745715	26748037	Distal Intergenic
chr8	26762011	26764600	Distal Intergenic
chr8	26909165	26909858	Distal Intergenic
chr8	26910143	26911190	Distal Intergenic
chr8	26911813	26912259	Distal Intergenic
chr8	26914991	26915633	Distal Intergenic
chr8	26916334	26916534	Distal Intergenic
chr8	26916587	26917385	Distal Intergenic
chr8	26929349	26930757	Distal Intergenic
chr8	26951109	26951316	Distal Intergenic
chr8	27229895	27230165	Intron
chr8	27923268	27923723	Intron
chr8	27934174	27935743	Intron
chr8	27939742	27940243	Intron
chr8	27941681	27943292	Promoter
chr8	27946153	27948146	Promoter

chr8	27996935	27998839	Intron
chr8	28073347	28074230	Distal Intergenic
chr8	28084701	28085410	Distal Intergenic
chr8	28085569	28085890	Distal Intergenic
chr8	28086232	28087450	Distal Intergenic
chr8	28087948	28089051	Distal Intergenic
chr8	28153365	28153599	Distal Intergenic
chr8	28153639	28153903	Distal Intergenic
chr8	28259995	28260407	Intron
chr8	28260459	28260856	Intron
chr8	28371507	28372220	Intron
chr8	29720167	29720399	Distal Intergenic
chr8	32222679	32224918	Intron
chr8	32259565	32260378	Intron
chr8	32260476	32261002	Intron
chr8	32277843	32278234	Intron
chr8	32278478	32279231	Intron
chr8	32483652	32484190	Intron
chr8	32631386	32632487	Distal Intergenic
chr8	32831129	32831339	Intron
chr8	32831377	32831635	Intron
chr8	32866003	32866730	Intron
chr8	33103765	33105203	Distal Intergenic
chr8	33117913	33118198	Distal Intergenic
chr8	33118369	33119082	Distal Intergenic
chr8	33119327	33119535	Distal Intergenic
chr8	33147497	33147745	Distal Intergenic
chr8	33148664	33150153	Distal Intergenic
chr8	33313200	33313924	Promoter
chr8	33320273	33320656	Intron
chr8	33328548	33329520	Intron
chr8	33329579	33331318	Promoter
chr8	33331405	33332625	Promoter
chr8	33374531	33377307	Distal Intergenic
chr8	33396649	33398438	Distal Intergenic
chr8	34751830	34752823	Distal Intergenic
chr8	34764755	34765030	Distal Intergenic
chr8	34770373	34771870	Distal Intergenic
chr8	34783519	34785240	Distal Intergenic
chr8	34798025	34799376	Distal Intergenic
chr8	34840931	34841605	Distal Intergenic
chr8	34843334	34843673	Distal Intergenic
chr8	34912602	34913317	Distal Intergenic
chr8	35078503	35079171	Distal Intergenic

chr8	35170955	35171327	Intron
chr8	35171542	35171741	Intron
chr8	35235801	35237072	Intron
chr8	35350382	35351508	Intron
chr8	35432740	35433194	Intron
chr8	35646733	35646934	Intron
chr8	35653808	35654963	Distal Intergenic
chr8	35675991	35676190	Distal Intergenic
chr8	35676257	35676508	Distal Intergenic
chr8	35676544	35676744	Distal Intergenic
chr8	35676839	35677151	Distal Intergenic
chr8	35677192	35677977	Distal Intergenic
chr8	35685636	35685872	Distal Intergenic
chr8	35685911	35686344	Distal Intergenic
chr8	36016152	36018149	Distal Intergenic
chr8	36027660	36027954	Distal Intergenic
chr8	36028963	36029268	Distal Intergenic
chr8	36384593	36384792	Distal Intergenic
chr8	36626842	36628412	Distal Intergenic
chr8	36629974	36630999	Distal Intergenic
chr8	36649505	36649746	Intron
chr8	36650399	36650772	Intron
chr8	36676682	36677007	Intron
chr8	36677320	36677792	Intron
chr8	36927937	36929655	Distal Intergenic
chr8	36929698	36930110	Distal Intergenic
chr8	36937116	36937744	Distal Intergenic
chr8	36944482	36945822	Distal Intergenic
chr8	36960947	36963142	Distal Intergenic
chr8	36963278	36965885	Distal Intergenic
chr8	36975201	36976967	Distal Intergenic
chr8	36981224	36982408	Distal Intergenic
chr8	36982610	36984171	Distal Intergenic
chr8	37002877	37004310	Distal Intergenic
chr8	37005895	37006193	Distal Intergenic
chr8	37033874	37034765	Distal Intergenic
chr8	37068274	37068847	Distal Intergenic
chr8	37091230	37092294	Distal Intergenic
chr8	37157121	37157811	Distal Intergenic
chr8	37157878	37158865	Distal Intergenic
chr8	37158913	37159112	Distal Intergenic
chr8	37159221	37159675	Distal Intergenic
chr8	37179636	37181087	Distal Intergenic
chr8	37218451	37218746	Distal Intergenic

chr8	37226793	37228878	Distal Intergenic
chr8	37234663	37236498	Distal Intergenic
chr8	37237274	37239529	Distal Intergenic
chr8	37247079	37247320	Distal Intergenic
chr8	37247429	37247959	Distal Intergenic
chr8	37249740	37250931	Distal Intergenic
chr8	37251727	37252722	Distal Intergenic
chr8	37252841	37254172	Distal Intergenic
chr8	37287925	37289989	Distal Intergenic
chr8	37310785	37311037	Distal Intergenic
chr8	37702724	37705709	Exon
chr8	37754441	37754769	Intron
chr8	37790020	37791478	Downstream
chr8	38025339	38025665	Intron
chr8	41594567	41595036	Intron
chr8	41595565	41595924	Intron
chr8	41595962	41597823	Intron
chr8	42325064	42325755	Intron
chr8	42433293	42434680	Distal Intergenic
chr8	42459563	42459852	Distal Intergenic
chr8	42460134	42460457	Distal Intergenic
chr8	42502137	42502463	Distal Intergenic
chr8	43154515	43155140	Intron
chr8	45346298	45346639	Distal Intergenic
chr8	45422255	45423724	Distal Intergenic
chr8	45540580	45540968	Distal Intergenic
chr8	45541011	45541495	Distal Intergenic
chr8	45606918	45608081	Distal Intergenic
chr8	45755966	45756783	Distal Intergenic
chr8	45843589	45845010	Distal Intergenic
chr8	45852589	45853108	Distal Intergenic
chr8	45884704	45885981	Distal Intergenic
chr8	45886847	45887057	Distal Intergenic
chr8	45887507	45888314	Distal Intergenic
chr8	45890642	45891219	Distal Intergenic
chr8	45949834	45950677	Distal Intergenic
chr8	45962242	45964170	Distal Intergenic
chr8	45967043	45969038	Distal Intergenic
chr8	45979498	45980157	Distal Intergenic
chr8	46010178	46010495	Distal Intergenic
chr8	46018675	46018905	Distal Intergenic
chr8	46019156	46020541	Distal Intergenic
chr8	46020620	46022053	Distal Intergenic
chr8	46022106	46022355	Distal Intergenic

chr8	46022511	46023195	Distal Intergenic
chr8	46023514	46024044	Distal Intergenic
chr8	46024109	46024536	Distal Intergenic
chr8	46024912	46025442	Distal Intergenic
chr8	46044507	46044948	Distal Intergenic
chr8	46045105	46045443	Distal Intergenic
chr8	46046034	46046755	Distal Intergenic
chr8	46046882	46047136	Distal Intergenic
chr8	46062779	46062978	Distal Intergenic
chr8	46063386	46064772	Distal Intergenic
chr8	46086033	46086246	Distal Intergenic
chr8	46086288	46086757	Distal Intergenic
chr8	46495728	46495946	Distal Intergenic
chr8	46614207	46614458	Distal Intergenic
chr8	46665242	46665719	Distal Intergenic
chr8	46734364	46736935	Distal Intergenic
chr8	46804930	46805314	Distal Intergenic
chr8	47531561	47532315	Distal Intergenic
chr8	47716031	47716231	Distal Intergenic
chr8	47799286	47799534	Distal Intergenic
chr8	47825137	47827135	Distal Intergenic
chr8	47866089	47866491	Distal Intergenic
chr8	47866614	47866832	Distal Intergenic
chr8	47896588	47896820	Distal Intergenic
chr8	47896879	47897078	Distal Intergenic
chr8	47951730	47952732	Distal Intergenic
chr8	47953413	47955411	Distal Intergenic
chr8	47955469	47955677	Distal Intergenic
chr8	48303805	48304060	Intron
chr8	48437861	48438270	Intron
chr8	48460562	48461010	Intron
chr8	48464787	48465067	Intron
chr8	48506302	48506820	Intron
chr8	48758547	48759031	Intron
chr8	48760270	48760517	Intron
chr8	48760778	48761370	Intron
chr8	49017601	49017904	Distal Intergenic
chr8	49018002	49018402	Distal Intergenic
chr8	49059113	49060557	Distal Intergenic
chr8	49067800	49069340	Distal Intergenic
chr8	49202025	49202677	Distal Intergenic
chr8	49633613	49633823	Intron
chr8	49633915	49635742	Intron
chr8	49636059	49636258	3' UTR

chr8	49639143	49639417	Intron
chr8	49639634	49639937	Intron
chr8	50367977	50368195	Distal Intergenic
chr8	55301111	55301377	Distal Intergenic
chr8	55301416	55301688	Distal Intergenic
chr8	55301825	55302027	Distal Intergenic
chr8	55510372	55510856	Distal Intergenic
chr8	55535002	55536165	Intron
chr8	55536274	55536738	Intron
chr8	58871791	58872193	Distal Intergenic
chr8	59029500	59029870	Intron
chr8	59977445	59977824	Intron
chr8	59990473	59991004	Intron
chr8	60068225	60068592	Distal Intergenic
chr8	60130601	60130899	Distal Intergenic
chr8	60278942	60279175	Distal Intergenic
chr8	63308859	63309984	Intron
chr8	63320227	63321861	Intron
chr8	63326869	63327343	Intron
chr8	63338286	63338756	Intron
chr8	63343738	63343948	Intron
chr8	63432472	63432891	Intron
chr8	63432930	63434074	Intron
chr8	63747330	63748108	Intron
chr8	63748348	63750481	Intron
chr8	63817158	63817408	Intron
chr8	63818556	63819290	Intron
chr8	63819415	63821410	Intron
chr8	64057215	64059591	Distal Intergenic
chr8	64059666	64060710	Distal Intergenic
chr8	64068677	64069244	Distal Intergenic
chr8	64069419	64070039	Distal Intergenic
chr8	64084992	64085464	5' UTR
chr8	64085918	64086144	Intron
chr8	64102175	64103397	Intron
chr8	64121071	64121427	Intron
chr8	64143659	64144837	Distal Intergenic
chr8	64176616	64176818	Distal Intergenic
chr8	64176966	64177408	Distal Intergenic
chr8	64261315	64261514	Distal Intergenic
chr8	64261628	64262221	Distal Intergenic
chr8	64265792	64266197	Distal Intergenic
chr8	64267871	64270577	Distal Intergenic
chr8	64274663	64276011	Distal Intergenic

chr8	64281323	64283388	Distal Intergenic
chr8	67001341	67002408	Intron
chr8	67021133	67021915	Distal Intergenic
chr8	67060981	67061274	Intron
chr8	67113083	67113418	Distal Intergenic
chr8	67204337	67206358	Distal Intergenic
chr8	67206488	67206698	Distal Intergenic
chr8	67206822	67207051	Distal Intergenic
chr8	67207262	67207872	Distal Intergenic
chr8	67211222	67212262	Distal Intergenic
chr8	67469282	67469909	Distal Intergenic
chr8	67728053	67728554	Intron
chr8	69205334	69205939	Distal Intergenic
chr8	69275565	69275773	Intron
chr8	69281768	69282820	Intron
chr8	69428887	69430895	Intron
chr8	70245731	70247368	Distal Intergenic
chr8	70248727	70249735	Distal Intergenic
chr8	70249926	70250323	Distal Intergenic
chr8	70434662	70434918	Intron
chr8	70454787	70455369	Intron
chr8	70460399	70461583	Intron
chr8	70534743	70534950	Intron
chr8	70535412	70535988	Intron
chr8	70606727	70608120	Intron
chr8	70680958	70681261	Intron
chr8	70681358	70681668	Intron
chr8	70695569	70696005	Intron
chr8	70698899	70699102	Intron
chr8	70701343	70701703	Intron
chr8	70721465	70721903	Intron
chr8	70722189	70722537	Intron
chr8	70767940	70768256	Distal Intergenic
chr8	70777100	70777553	Distal Intergenic
chr8	70850595	70851124	Distal Intergenic
chr8	71551855	71552268	Intron
chr8	71561381	71562780	Intron
chr8	71732950	71733343	Distal Intergenic
chr8	71919063	71919298	Distal Intergenic
chr8	72371959	72374564	Intron
chr8	72374624	72375380	Intron
chr8	72460065	72461005	Distal Intergenic
chr8	72461213	72461516	Distal Intergenic
chr8	72463580	72463861	Distal Intergenic

chr8	72519383	72519775	Distal Intergenic
chr8	72520749	72521222	Distal Intergenic
chr8	72524805	72525661	Distal Intergenic
chr8	72536008	72536910	Distal Intergenic
chr8	72745692	72746112	Distal Intergenic
chr8	72758849	72759070	Intron
chr8	72759979	72760433	Intron
chr8	72760626	72762626	Intron
chr8	72795349	72797099	Intron
chr8	72797136	72797335	Intron
chr8	72876483	72876682	Intron
chr8	72978185	72978429	Intron
chr8	72978470	72978819	Intron
chr8	73000147	73000641	Distal Intergenic
chr8	73001177	73001437	Distal Intergenic
chr8	73033535	73033769	Distal Intergenic
chr8	73034388	73034803	Distal Intergenic
chr8	73046787	73047007	Distal Intergenic
chr8	73053051	73054386	Distal Intergenic
chr8	73063949	73064202	Distal Intergenic
chr8	73085610	73086280	Distal Intergenic
chr8	73172955	73173744	Distal Intergenic
chr8	73197678	73197881	Distal Intergenic
chr8	73201168	73201373	Distal Intergenic
chr8	73202772	73202990	Distal Intergenic
chr8	73203478	73204123	Distal Intergenic
chr8	73206014	73206238	Distal Intergenic
chr8	73222471	73222670	Distal Intergenic
chr8	73290275	73290558	Distal Intergenic
chr8	73290643	73290940	Distal Intergenic
chr8	73296928	73297481	Distal Intergenic
chr8	73297553	73298279	Distal Intergenic
chr8	73300924	73301151	Distal Intergenic
chr8	73403776	73403982	Distal Intergenic
chr8	73404153	73404770	Distal Intergenic
chr8	73420533	73421768	Distal Intergenic
chr8	73796955	73797683	Intron
chr8	73800361	73800687	Intron
chr8	73836562	73836888	Intron
chr8	73891136	73891365	Distal Intergenic
chr8	73891539	73892124	Distal Intergenic
chr8	73894787	73895026	Distal Intergenic
chr8	74096582	74097877	Distal Intergenic
chr8	74097967	74098524	Distal Intergenic

chr8	74102155	74102397	Distal Intergenic
chr8	74102903	74103367	Distal Intergenic
chr8	74104634	74105782	Distal Intergenic
chr8	74195725	74195925	Distal Intergenic
chr8	74200032	74200237	Downstream
chr8	74225842	74227363	Intron
chr8	74446765	74447510	Intron
chr8	74659707	74660711	Promoter
chr8	74668137	74668850	Distal Intergenic
chr8	74669243	74671633	Distal Intergenic
chr8	74685259	74685639	Distal Intergenic
chr8	74931503	74932285	Intron
chr8	74932342	74932545	Intron
chr8	74937607	74937946	Intron
chr8	74999356	74999567	Distal Intergenic
chr8	74999629	74999852	Distal Intergenic
chr8	75016820	75017232	Distal Intergenic
chr8	75034383	75035666	Distal Intergenic
chr8	75071353	75071634	Distal Intergenic
chr8	75095106	75095735	Distal Intergenic
chr8	75178981	75179395	Intron
chr8	75179456	75179984	Intron
chr8	75187770	75189587	Intron
chr8	75647551	75647804	Intron
chr8	75647977	75648872	Intron
chr8	75799678	75800253	Distal Intergenic
chr8	75805619	75806189	Distal Intergenic
chr8	75808576	75808797	Distal Intergenic
chr8	75808842	75809077	Distal Intergenic
chr8	75813371	75814350	Distal Intergenic
chr8	75814509	75814739	Distal Intergenic
chr8	77617044	77617256	Promoter
chr8	77617552	77617751	Exon
chr8	77632715	77633225	Intron
chr8	77693355	77694013	Intron
chr8	78012945	78013144	Distal Intergenic
chr8	79851107	79851323	Distal Intergenic
chr8	79945498	79945939	Distal Intergenic
chr8	79966186	79967572	Distal Intergenic
chr8	79967873	79969013	Distal Intergenic
chr8	79982484	79983169	Distal Intergenic
chr8	80005433	80006584	Distal Intergenic
chr8	80040475	80040975	Distal Intergenic
chr8	80301617	80301849	Distal Intergenic

chr8	80845746	80846071	Intron
chr8	80846152	80846807	Intron
chr8	80846857	80847076	Intron
chr8	81031957	81033049	Intron
chr8	81033205	81033690	Intron
chr8	81033968	81035429	Intron
chr8	81104784	81106047	Distal Intergenic
chr8	81108524	81109052	Distal Intergenic
chr8	81172180	81172388	Distal Intergenic
chr8	81552414	81552648	3' UTR
chr8	81731720	81732289	Intron
chr8	81732475	81733538	Intron
chr8	81818245	81818540	Distal Intergenic
chr8	81873645	81875485	Distal Intergenic
chr8	82066121	82066546	Distal Intergenic
chr8	82074376	82074611	Distal Intergenic
chr8	82085525	82085848	Distal Intergenic
chr8	82085895	82086442	Distal Intergenic
chr8	82106821	82107083	Distal Intergenic
chr8	82235134	82235472	Distal Intergenic
chr8	83235016	83235511	Distal Intergenic
chr8	83263915	83264134	Distal Intergenic
chr8	83375890	83376510	Distal Intergenic
chr8	83376677	83378581	Distal Intergenic
chr8	83380261	83381240	Distal Intergenic
chr8	83381489	83382559	Distal Intergenic
chr8	83382697	83382977	Distal Intergenic
chr8	83385472	83386322	Distal Intergenic
chr8	83390850	83392302	Distal Intergenic
chr8	83392473	83392855	Distal Intergenic
chr8	84280532	84282111	Distal Intergenic
chr8	84373547	84373791	Distal Intergenic
chr8	84374316	84374515	Distal Intergenic
chr8	84378189	84378738	Distal Intergenic
chr8	84389556	84390328	Distal Intergenic
chr8	84390888	84391310	Distal Intergenic
chr8	84849612	84849914	Distal Intergenic
chr8	85297450	85297690	Intron
chr8	86117544	86117743	Intron
chr8	86125796	86126909	3' UTR
chr8	86131076	86131301	Intron
chr8	86131406	86131791	Promoter
chr8	86132435	86133475	Promoter
chr8	86142866	86144284	Intron

chr8	86239147	86241093	3' UTR
chr8	86714791	86715069	Distal Intergenic
chr8	86715108	86716645	Distal Intergenic
chr8	86721064	86722453	Distal Intergenic
chr8	86722494	86724731	Distal Intergenic
chr8	86725074	86728752	5' UTR
chr8	86860278	86860529	Distal Intergenic
chr8	87180323	87180747	Distal Intergenic
chr8	87180805	87182303	Distal Intergenic
chr8	87183895	87184292	Distal Intergenic
chr8	87184340	87186014	Distal Intergenic
chr8	87356096	87356390	Intron
chr8	87433677	87433927	Intron
chr8	87492506	87492965	Exon
chr8	87493186	87493636	Intron
chr8	87498354	87500181	Exon
chr8	87500597	87501351	Exon
chr8	87501501	87501753	Intron
chr8	87501799	87502069	Intron
chr8	87502290	87502574	Intron
chr8	87503007	87506872	Intron
chr8	87506971	87509748	Intron
chr8	87513581	87513788	Intron
chr8	87540597	87541134	5' UTR
chr8	87549178	87549425	Intron
chr8	87550816	87551659	Intron
chr8	87556632	87556867	Intron
chr8	87556919	87557453	Exon
chr8	87559483	87560606	Promoter
chr8	87561106	87562206	Intron
chr8	87562470	87562699	Intron
chr8	87564839	87565054	Intron
chr8	87565181	87565424	Intron
chr8	87584596	87584932	Downstream
chr8	87595309	87595572	Intron
chr8	87825848	87826116	Distal Intergenic
chr8	87861241	87862387	Distal Intergenic
chr8	87932476	87932826	Intron
chr8	87953242	87953516	Intron
chr8	87953567	87953791	Intron
chr8	87954723	87955074	Intron
chr8	87955324	87955568	Intron
chr8	88036828	88037959	Intron
chr8	88038196	88038448	Intron

chr8	88042845	88043231	Intron
chr8	88061494	88062016	Intron
chr8	88078084	88078802	Intron
chr8	88967220	88967746	Distal Intergenic
chr8	88969581	88969840	Distal Intergenic
chr8	88978417	88979210	Distal Intergenic
chr8	89254272	89255307	Intron
chr8	89269090	89269563	Intron
chr8	89269630	89269904	Intron
chr8	89289662	89291262	Intron
chr8	89291760	89292862	Intron
chr8	89293012	89294966	Intron
chr8	89296459	89297698	Intron
chr8	89408101	89408406	Distal Intergenic
chr8	90684281	90684560	Distal Intergenic
chr8	90804790	90805614	Distal Intergenic
chr8	90806894	90807130	Distal Intergenic
chr8	90807339	90807541	Distal Intergenic
chr8	90807647	90808990	Distal Intergenic
chr8	91160968	91162836	Distal Intergenic
chr8	91170777	91171692	Distal Intergenic
chr8	91171903	91172641	Distal Intergenic
chr8	91172792	91175192	Distal Intergenic
chr8	91222631	91222973	Distal Intergenic
chr8	93348737	93349930	Distal Intergenic
chr8	93352225	93352650	Distal Intergenic
chr8	93352748	93353214	Distal Intergenic
chr8	93363786	93364487	Distal Intergenic
chr8	93364669	93364871	Distal Intergenic
chr8	93385141	93385725	Distal Intergenic
chr8	93387953	93388749	Distal Intergenic
chr8	93399903	93401030	Distal Intergenic
chr8	93401086	93401485	Distal Intergenic
chr8	93410987	93411278	Distal Intergenic
chr8	93697225	93697567	Distal Intergenic
chr8	93828200	93828603	Distal Intergenic
chr8	93828760	93829072	Distal Intergenic
chr8	93919012	93920165	Intron
chr8	93920337	93920569	Intron
chr8	93933924	93934123	Intron
chr8	93934176	93934418	Intron
chr8	93935440	93935969	Intron
chr8	94012958	94013217	Intron
chr8	94013270	94014276	Intron

chr8	94016888	94017410	Intron
chr8	94018825	94019129	Intron
chr8	94824701	94825110	Intron
chr8	95012897	95013168	Distal Intergenic
chr8	95370416	95373581	Distal Intergenic
chr8	95418673	95419539	Intron
chr8	95426514	95426962	Intron
chr8	95554521	95555166	Intron
chr8	95893155	95893763	3' UTR
chr8	95898978	95899292	Intron
chr8	96464966	96465303	Intron
chr8	96505187	96506454	Intron
chr8	96707639	96708442	Intron
chr8	96708667	96709066	Intron
chr8	96710182	96710554	Intron
chr8	97501193	97501392	Distal Intergenic
chr8	97506584	97508366	Promoter
chr8	97516496	97517335	Intron
chr8	97571223	97571682	Intron
chr8	97615123	97615699	Intron
chr8	97615862	97616087	Intron
chr8	97699217	97700116	Intron
chr8	97872476	97872677	Intron
chr8	98331656	98331915	Distal Intergenic
chr8	98372753	98373527	Distal Intergenic
chr8	106960181	106960439	Distal Intergenic
chr8	107410211	107410542	Intron
chr8	107410620	107410852	Intron
chr8	107426421	107427782	Intron
chr8	107449589	107450649	Intron
chr8	107458772	107459848	Promoter
chr8	107485033	107486287	Intron
chr8	107683024	107683827	Intron
chr8	107683868	107684119	Intron
chr8	107710265	107710657	Intron
chr8	107751844	107752078	Intron
chr8	107776884	107777308	Intron
chr8	107785462	107785791	Distal Intergenic
chr8	107788177	107788460	Distal Intergenic
chr8	107821411	107821766	Distal Intergenic
chr8	107827859	107828259	Distal Intergenic
chr8	107828407	107829125	Distal Intergenic
chr8	107829306	107829916	Distal Intergenic
chr8	108117667	108118082	Distal Intergenic

chr8	108160644	108161349	Distal Intergenic
chr8	108161589	108161951	Distal Intergenic
chr8	108313493	108314071	Intron
chr8	108322843	108323664	Intron
chr8	108374761	108375248	Intron
chr8	108375305	108375736	Intron
chr8	108384511	108384851	Intron
chr8	108385593	108385889	Intron
chr8	108424390	108425107	Intron
chr8	108582478	108583322	Distal Intergenic
chr8	108593224	108594341	Distal Intergenic
chr8	108658174	108658436	Distal Intergenic
chr8	108658474	108658693	Distal Intergenic
chr8	108659274	108660030	Distal Intergenic
chr8	108660138	108660533	Distal Intergenic
chr8	108660773	108662504	Distal Intergenic
chr8	108745824	108746177	Distal Intergenic
chr8	109042337	109045144	Intron
chr8	109050493	109054597	Intron
chr8	109085232	109085810	Intron
chr8	109087333	109088623	Intron
chr8	109134560	109135070	Distal Intergenic
chr8	109135824	109136023	Distal Intergenic
chr8	109139361	109140669	Distal Intergenic
chr8	109143019	109143279	Distal Intergenic
chr8	109143417	109143616	Distal Intergenic
chr8	109147729	109147973	Distal Intergenic
chr8	109260828	109261062	Promoter
chr8	109274979	109275485	Distal Intergenic
chr8	109292665	109293923	Distal Intergenic
chr8	109294080	109294480	Distal Intergenic
chr8	109313033	109314365	Distal Intergenic
chr8	109333672	109334567	Distal Intergenic
chr8	109341854	109344262	Distal Intergenic
chr8	109416249	109416927	Distal Intergenic
chr8	109477497	109478113	Intron
chr8	109674167	109674446	Intron
chr8	109674702	109674932	Intron
chr8	109730386	109730849	Intron
chr8	109730890	109731240	Intron
chr8	109778332	109779414	Intron
chr8	109779991	109780227	Intron
chr8	109815263	109815867	Distal Intergenic
chr8	109927062	109927399	Distal Intergenic

chr8	109976954	109977267	Distal Intergenic
chr8	109981675	109981890	Distal Intergenic
chr8	110024660	110028078	Distal Intergenic
chr8	110068988	110069325	Distal Intergenic
chr8	110112131	110112332	Intron
chr8	110904129	110904328	Distal Intergenic
chr8	111246376	111247460	Distal Intergenic
chr8	111250100	111250367	Distal Intergenic
chr8	111250473	111250881	Distal Intergenic
chr8	111288073	111290281	Distal Intergenic
chr8	112089234	112089714	Distal Intergenic
chr8	112174537	112177021	Distal Intergenic
chr8	112199881	112200758	Distal Intergenic
chr8	112216627	112216943	Distal Intergenic
chr8	112273541	112273959	Distal Intergenic
chr8	112274092	112274725	Distal Intergenic
chr8	112274770	112275191	Distal Intergenic
chr8	112302835	112303809	Distal Intergenic
chr8	112334011	112334214	Distal Intergenic
chr8	112334302	112335326	Distal Intergenic
chr8	112443608	112443916	Distal Intergenic
chr8	112444073	112445183	Distal Intergenic
chr8	112481915	112482235	Distal Intergenic
chr8	113278557	113278960	Intron
chr8	113318230	113318599	Exon
chr8	113348961	113349185	Promoter
chr8	113369978	113370203	Intron
chr8	113370432	113371107	Intron
chr8	113464651	113466073	Intron
chr8	113557784	113558355	Intron
chr8	113581143	113581342	Intron
chr8	113805892	113806677	Intron
chr8	113861351	113861581	Intron
chr8	113861902	113862429	Intron
chr8	113959161	113959413	Intron
chr8	114061373	114061751	Intron
chr8	114062229	114062428	Intron
chr8	114123397	114123689	Intron
chr8	114123872	114124348	Intron
chr8	114128815	114129036	Intron
chr8	114129087	114129425	Intron
chr8	114250049	114251322	Intron
chr8	114255437	114255943	Intron
chr8	114256134	114256747	Intron

chr8	114257004	114257528	Intron
chr8	114269343	114271360	Intron
chr8	114271548	114272365	Intron
chr8	114303291	114303607	Intron
chr8	114303767	114306390	Intron
chr8	114429618	114429848	Intron
chr8	114534645	114534873	Distal Intergenic
chr8	118288822	118289241	Distal Intergenic
chr8	118294667	118295253	Distal Intergenic
chr8	118295372	118295728	Distal Intergenic
chr8	118307167	118307415	Distal Intergenic
chr8	118378608	118379229	Distal Intergenic
chr8	118606379	118607596	Distal Intergenic
chr8	118620358	118620590	Distal Intergenic
chr8	118620889	118621372	Distal Intergenic
chr8	118623655	118623919	Distal Intergenic
chr8	118922217	118922438	Intron
chr8	119645167	119646017	Intron
chr8	119646284	119646483	Intron
chr8	119646560	119648866	Intron
chr8	119694127	119694565	Intron
chr8	119781190	119781862	Distal Intergenic
chr8	119782074	119783070	Distal Intergenic
chr8	119850502	119853152	Distal Intergenic
chr8	119857917	119858140	Distal Intergenic
chr8	119858362	119860225	Distal Intergenic
chr8	119895666	119895934	Distal Intergenic
chr8	119896103	119896835	Distal Intergenic
chr8	119910114	119910326	Distal Intergenic
chr8	119910453	119910820	Distal Intergenic
chr8	119910967	119911502	Distal Intergenic
chr8	120085142	120085767	Intron
chr8	120243043	120243383	Intron
chr8	120310606	120311391	Distal Intergenic
chr8	120810362	120811361	Intron
chr8	121945305	121947213	Distal Intergenic
chr8	121952171	121952431	Distal Intergenic
chr8	121954774	121954973	Distal Intergenic
chr8	122210669	122211133	Distal Intergenic
chr8	122211298	122212376	Distal Intergenic
chr8	122232492	122233191	Distal Intergenic
chr8	122234255	122234481	Distal Intergenic
chr8	122335182	122335619	Distal Intergenic
chr8	122335768	122337475	Distal Intergenic

chr8	122338308	122338520	Distal Intergenic
chr8	122339743	122339959	Distal Intergenic
chr8	122340116	122342300	Distal Intergenic
chr8	122352798	122354047	Distal Intergenic
chr8	122471867	122472535	Distal Intergenic
chr8	122472737	122473065	Distal Intergenic
chr8	122473104	122473303	Distal Intergenic
chr8	122645032	122645477	Intron
chr8	122645854	122646216	Intron
chr8	123062308	123062547	Intron
chr8	123087155	123088053	Intron
chr8	123806884	123807404	Intron
chr8	124192057	124192418	Promoter
chr8	124192563	124192922	Exon
chr8	124193125	124193501	Promoter
chr8	124385747	124386039	Intron
chr8	124386099	124386303	Intron
chr8	124422159	124422409	Intron
chr8	124494775	124495011	Distal Intergenic
chr8	124528051	124528390	Intron
chr8	124542806	124543535	Promoter
chr8	124543585	124544881	Promoter
chr8	124867064	124867283	Intron
chr8	124867452	124867890	Intron
chr8	124985356	124985706	Exon
chr8	125047005	125047232	Intron
chr8	125047283	125048411	Exon
chr8	125050134	125050743	Intron
chr8	125060691	125061614	Intron
chr8	125072617	125072899	Exon
chr8	125090935	125091222	Intron
chr8	125091277	125091677	Intron
chr8	125092274	125092497	Intron
chr8	125099193	125100747	Intron
chr8	125109116	125109818	Exon
chr8	125429117	125430876	Distal Intergenic
chr8	125456084	125456320	Distal Intergenic
chr8	125457845	125458082	Distal Intergenic
chr8	125522083	125522295	Intron
chr8	125544813	125547486	Intron
chr8	125625241	125625454	Intron
chr8	125627614	125627813	Intron
chr8	125937168	125937768	Exon
chr8	125970978	125971430	Distal Intergenic

chr8	126407851	126408271	Distal Intergenic
chr8	126612299	126612543	Distal Intergenic
chr8	126789224	126789470	Distal Intergenic
chr8	126789560	126789846	Distal Intergenic
chr8	126794633	126795088	Distal Intergenic
chr8	126801489	126803163	Distal Intergenic
chr8	126854884	126855112	Distal Intergenic
chr8	126871764	126872122	Distal Intergenic
chr8	127167920	127168125	Distal Intergenic
chr8	127168161	127168388	Distal Intergenic
chr8	127777368	127777771	Distal Intergenic
chr8	128498326	128498563	Distal Intergenic
chr8	128519990	128520742	Distal Intergenic
chr8	129046705	129046914	Intron
chr8	129046960	129047854	Intron
chr8	129055141	129055552	Intron
chr8	129055860	129056372	Intron
chr8	129063141	129063541	Intron
chr8	129063757	129064481	Intron
chr8	129094981	129095305	Intron
chr8	129113718	129114085	Distal Intergenic
chr8	129115837	129116142	Distal Intergenic
chr8	129119041	129119262	Distal Intergenic
chr8	129119448	129120068	Distal Intergenic
chr8	129120141	129120685	Distal Intergenic
chr8	129127271	129127522	Distal Intergenic
chr8	129137841	129139004	Distal Intergenic
chr8	129139112	129140155	Distal Intergenic
chr8	129196622	129196950	Distal Intergenic
chr8	129208891	129209258	Distal Intergenic
chr8	129209909	129210827	Distal Intergenic
chr8	129211070	129211884	Distal Intergenic
chr8	129363126	129363386	Distal Intergenic
chr8	129376065	129376927	Distal Intergenic
chr8	129495102	129497034	Distal Intergenic
chr8	129552544	129553090	Distal Intergenic
chr8	129626946	129627425	Distal Intergenic
chr8	129633250	129633669	Distal Intergenic
chr8	129633738	129634186	Distal Intergenic
chr8	129637129	129637328	Distal Intergenic
chr8	129685890	129686616	Distal Intergenic
chr8	129750470	129751816	Distal Intergenic
chr8	129751854	129752956	Distal Intergenic
chr8	130636952	130637405	Distal Intergenic

chr8	130689279	130689750	Distal Intergenic
chr8	130743280	130743675	Distal Intergenic
chr8	131143226	131143507	Intron
chr8	131178566	131179014	Intron
chr8	131190547	131190848	Intron
chr8	131191110	131191372	Intron
chr8	131209041	131209374	Intron
chr8	131209600	131210665	Intron
chr8	131210792	131211139	Intron
chr8	131214341	131214540	Intron
chr8	131221820	131222075	Intron
chr8	131226407	131226733	Intron
chr8	131226772	131228060	Exon
chr8	131228265	131229384	Intron
chr9	3403415	3404401	Intron
chr9	4309054	4309433	Distal Intergenic
chr9	5309100	5309427	Distal Intergenic
chr9	6374019	6374247	Distal Intergenic
chr9	7183829	7184489	Distal Intergenic
chr9	7387623	7388323	Distal Intergenic
chr9	7510003	7510478	Distal Intergenic
chr9	7835902	7837020	Distal Intergenic
chr9	8003101	8003504	Distal Intergenic
chr9	8003613	8003974	Distal Intergenic
chr9	8004177	8004549	Distal Intergenic
chr9	8004907	8005121	Distal Intergenic
chr9	8103677	8103907	Distal Intergenic
chr9	8104067	8104393	Distal Intergenic
chr9	8104431	8104715	Distal Intergenic
chr9	8113777	8114884	Distal Intergenic
chr9	9120380	9120579	Intron
chr9	9120655	9120871	Intron
chr9	9171323	9171785	Intron
chr9	9207408	9208074	Intron
chr9	9241131	9241360	Intron
chr9	9241428	9242423	Intron
chr9	9245764	9246406	Intron
chr9	11380235	11381220	Distal Intergenic
chr9	11570268	11571375	Distal Intergenic
chr9	12016690	12017090	Distal Intergenic
chr9	12017208	12017434	Distal Intergenic
chr9	13228486	13228721	Intron
chr9	13245194	13246020	Intron
chr9	13258490	13260273	Intron

chr9	13298791	13299024	Distal Intergenic
chr9	13299887	13301645	Distal Intergenic
chr9	13326965	13327867	Distal Intergenic
chr9	13329784	13330533	Distal Intergenic
chr9	13365021	13365459	Distal Intergenic
chr9	13385264	13386109	Distal Intergenic
chr9	13464843	13465885	Distal Intergenic
chr9	13468541	13469456	Distal Intergenic
chr9	13469532	13470086	Distal Intergenic
chr9	13473235	13473499	Distal Intergenic
chr9	13553885	13554256	Distal Intergenic
chr9	13632195	13632439	Distal Intergenic
chr9	13632712	13633038	Distal Intergenic
chr9	13776081	13776722	Distal Intergenic
chr9	13777576	13778259	Distal Intergenic
chr9	13778415	13778641	Distal Intergenic
chr9	13858040	13858390	Distal Intergenic
chr9	13961802	13962018	Distal Intergenic
chr9	14019980	14020585	Distal Intergenic
chr9	14169750	14169964	Intron
chr9	14170091	14171631	Intron
chr9	14171697	14172995	Intron
chr9	14183919	14184167	Intron
chr9	14184498	14184783	Intron
chr9	14325466	14326944	Intron
chr9	14339683	14339998	Intron
chr9	14362220	14362653	Intron
chr9	14363695	14364117	Intron
chr9	14364170	14364638	Intron
chr9	14372785	14373323	Intron
chr9	14373536	14373779	Intron
chr9	14393126	14393489	Intron
chr9	14408912	14409264	Distal Intergenic
chr9	14417345	14420123	Distal Intergenic
chr9	14422410	14422713	Distal Intergenic
chr9	14465940	14468233	Distal Intergenic
chr9	14480322	14480817	Distal Intergenic
chr9	14588684	14589022	Intron
chr9	14589127	14589328	Intron
chr9	14834226	14834627	Intron
chr9	14849040	14849352	Intron
chr9	14873160	14874270	Intron
chr9	14923636	14924133	Distal Intergenic
chr9	14924381	14924698	Distal Intergenic

chr9	14926287	14927510	Distal Intergenic
chr9	14942921	14943431	Distal Intergenic
chr9	14949895	14951047	Distal Intergenic
chr9	15042377	15042792	Intron
chr9	15110796	15111015	Intron
chr9	15111069	15111320	Intron
chr9	15111368	15112744	Intron
chr9	15113726	15114074	Intron
chr9	15161591	15162248	Distal Intergenic
chr9	15531954	15532193	Distal Intergenic
chr9	15539156	15539519	Distal Intergenic
chr9	15551102	15551920	Promoter
chr9	17362046	17362901	Intron
chr9	18096347	18097781	Distal Intergenic
chr9	19260096	19260916	Intron
chr9	19261025	19261257	Intron
chr9	19426532	19426959	Intron
chr9	20263993	20264820	Distal Intergenic
chr9	20296791	20297243	Distal Intergenic
chr9	20297312	20297844	Distal Intergenic
chr9	20325072	20325271	Distal Intergenic
chr9	20325664	20325873	Distal Intergenic
chr9	20411872	20412071	Promoter
chr9	20447016	20447495	Intron
chr9	20447675	20448228	Exon
chr9	20449024	20449237	Intron
chr9	20449358	20449621	Intron
chr9	20466169	20466399	Intron
chr9	20466465	20466915	Intron
chr9	20532463	20533707	Intron
chr9	20692659	20693089	Intron
chr9	20702242	20702447	Intron
chr9	20702561	20702760	Intron
chr9	20748893	20749625	Intron
chr9	20756257	20756746	Intron
chr9	20877859	20878375	Intron
chr9	21029542	21030033	Intron
chr9	21042790	21043098	Distal Intergenic
chr9	21055396	21055925	Distal Intergenic
chr9	21062001	21062217	Distal Intergenic
chr9	21068712	21069636	Distal Intergenic
chr9	21092066	21092366	Distal Intergenic
chr9	21136959	21138537	Downstream
chr9	21141877	21142076	Promoter

chr9	21172758	21173181	Distal Intergenic
chr9	21229307	21229779	Intron
chr9	21298779	21299504	Distal Intergenic
chr9	21351824	21352108	Promoter
chr9	21420056	21420352	Distal Intergenic
chr9	21420702	21421024	Distal Intergenic
chr9	21421108	21421338	Distal Intergenic
chr9	21421503	21422207	Distal Intergenic
chr9	21474518	21474730	Intron
chr9	21484396	21485398	Intron
chr9	21491042	21491279	Intron
chr9	21491336	21491842	Intron
chr9	21532831	21533920	Intron
chr9	21596294	21596493	Distal Intergenic
chr9	21728115	21728429	Distal Intergenic
chr9	21728619	21729065	Distal Intergenic
chr9	22029772	22030026	Intron
chr9	22030448	22032004	Intron
chr9	22190357	22190578	Distal Intergenic
chr9	22190785	22191227	Distal Intergenic
chr9	22192523	22193281	Distal Intergenic
chr9	22193352	22195534	Distal Intergenic
chr9	22210106	22210328	Distal Intergenic
chr9	23498237	23500191	Distal Intergenic
chr9	23503381	23504133	Intron
chr9	24747311	24747634	Distal Intergenic
chr9	24748095	24748477	Distal Intergenic
chr9	24748596	24749043	Distal Intergenic
chr9	24847023	24847384	Distal Intergenic
chr9	24955880	24956736	Distal Intergenic
chr9	24957271	24957774	Distal Intergenic
chr9	25051494	25052006	Distal Intergenic
chr9	25058957	25059932	Distal Intergenic
chr9	25060295	25061032	Distal Intergenic
chr9	25061123	25061323	Distal Intergenic
chr9	25074371	25075085	Distal Intergenic
chr9	25399189	25400132	Exon
chr9	25400283	25400667	Distal Intergenic
chr9	26806881	26807166	Distal Intergenic
chr9	26807284	26807729	Distal Intergenic
chr9	26834561	26834803	Distal Intergenic
chr9	28716075	28716303	Intron
chr9	28797652	28797925	Intron
chr9	28800252	28801408	Intron

chr9	28804692	28809082	Intron
chr9	28811313	28813132	Intron
chr9	28816154	28816448	Intron
chr9	28867024	28869821	Intron
chr9	28875134	28875551	Intron
chr9	29046668	29047243	Intron
chr9	29170980	29171605	Intron
chr9	29301427	29302066	Distal Intergenic
chr9	30585099	30586277	Distal Intergenic
chr9	30586521	30586804	Distal Intergenic
chr9	30587826	30591650	Distal Intergenic
chr9	30600786	30603062	Distal Intergenic
chr9	30838227	30839054	Distal Intergenic
chr9	31010765	31012231	Distal Intergenic
chr9	31012346	31012852	Distal Intergenic
chr9	31025652	31025851	Distal Intergenic
chr9	31050028	31051748	Distal Intergenic
chr9	31072153	31073174	Distal Intergenic
chr9	31073245	31074157	Distal Intergenic
chr9	31087329	31088715	Distal Intergenic
chr9	31088772	31089430	Distal Intergenic
chr9	31126400	31127402	Distal Intergenic
chr9	31127979	31130096	Distal Intergenic
chr9	31306612	31307364	Distal Intergenic
chr9	31315070	31315286	Distal Intergenic
chr9	31315328	31316330	Distal Intergenic
chr9	31421613	31422072	Distal Intergenic
chr9	31423255	31423539	Distal Intergenic
chr9	31458118	31458902	Distal Intergenic
chr9	31462066	31462502	Distal Intergenic
chr9	31663703	31664576	Distal Intergenic
chr9	31805092	31806649	Distal Intergenic
chr9	31806711	31807000	Distal Intergenic
chr9	31818489	31819421	Distal Intergenic
chr9	31863333	31864304	Distal Intergenic
chr9	31864830	31865965	Distal Intergenic
chr9	31871092	31871299	Distal Intergenic
chr9	31923320	31924511	Distal Intergenic
chr9	31924582	31927166	Distal Intergenic
chr9	31927203	31929180	Distal Intergenic
chr9	31966876	31967814	Distal Intergenic
chr9	31967899	31969733	Distal Intergenic
chr9	32031224	32032264	Distal Intergenic
chr9	32032353	32032665	Distal Intergenic

chr9	32436742	32436979	Intron
chr9	32503073	32503480	Promoter
chr9	32504324	32504672	Intron
chr9	32512990	32513859	Intron
chr9	32515797	32516224	Intron
chr9	32516320	32519063	Intron
chr9	32521763	32522668	Intron
chr9	32557279	32557967	Intron
chr9	32788678	32789110	3' UTR
chr9	33666452	33666783	Distal Intergenic
chr9	34087080	34087634	3' UTR
chr9	34087680	34088081	3' UTR
chr9	34869089	34870000	Distal Intergenic
chr9	34924614	34926584	Distal Intergenic
chr9	34946930	34947187	Distal Intergenic
chr9	35000878	35001386	Distal Intergenic
chr9	35006790	35007099	Distal Intergenic
chr9	35018106	35018620	Distal Intergenic
chr9	35019111	35019481	Distal Intergenic
chr9	35055362	35056081	3' UTR
chr9	35367093	35367320	Intron
chr9	36533908	36534177	Distal Intergenic
chr9	36534269	36534599	Distal Intergenic
chr9	36535142	36535518	Distal Intergenic
chr9	36542702	36543680	Distal Intergenic
chr9	36544274	36544554	Distal Intergenic
chr9	36603961	36604629	5' UTR
chr9	37043217	37043546	Distal Intergenic
chr9	37155544	37155974	Intron
chr9	37226183	37227019	Intron
chr9	37295971	37296829	Intron
chr9	37463781	37465251	Promoter
chr9	37541267	37542508	5' UTR
chr9	37544060	37544377	Intron
chr9	37641136	37641335	Distal Intergenic
chr9	38525849	38526089	Distal Intergenic
chr9	38526142	38526940	Distal Intergenic
chr9	40554760	40555475	Intron
chr9	40555542	40556305	Intron
chr9	40573186	40576550	Intron
chr9	40587871	40591057	Intron
chr9	40602446	40604537	Intron
chr9	40606416	40606638	Intron
chr9	40607033	40608909	Intron

chr9	40609335	40609544	Intron
chr9	40609725	40609993	Intron
chr9	40610132	40610373	5' UTR
chr9	40614518	40615564	Intron
chr9	40615612	40615826	Intron
chr9	40829481	40829715	Intron
chr9	41288597	41288892	Distal Intergenic
chr9	41296137	41296538	Distal Intergenic
chr9	41316935	41317210	Distal Intergenic
chr9	41385648	41386060	Distal Intergenic
chr9	41387761	41387985	Distal Intergenic
chr9	41388460	41388733	Distal Intergenic
chr9	41388807	41390031	Distal Intergenic
chr9	41390136	41392806	Distal Intergenic
chr9	41392860	41394228	Distal Intergenic
chr9	41395387	41395774	Distal Intergenic
chr9	41395886	41397206	Distal Intergenic
chr9	41543092	41544105	Distal Intergenic
chr9	41640516	41640743	Distal Intergenic
chr9	41666515	41667089	Distal Intergenic
chr9	41816526	41817231	Distal Intergenic
chr9	41901876	41903082	Distal Intergenic
chr9	41916070	41918737	Distal Intergenic
chr9	41988382	41988581	Intron
chr9	42060142	42060726	Distal Intergenic
chr9	42060873	42061137	Distal Intergenic
chr9	42071324	42072387	Distal Intergenic
chr9	42072722	42073122	Distal Intergenic
chr9	42073174	42073373	Distal Intergenic
chr9	42080923	42081893	Distal Intergenic
chr9	42081961	42082182	Distal Intergenic
chr9	42269494	42269975	Distal Intergenic
chr9	42279744	42279969	Distal Intergenic
chr9	42811097	42811646	Distal Intergenic
chr9	42811736	42812138	Distal Intergenic
chr9	42818944	42819796	Distal Intergenic
chr9	42819869	42820068	Distal Intergenic
chr9	42822807	42824717	Distal Intergenic
chr9	42825050	42825371	Distal Intergenic
chr9	42842422	42842679	Downstream
chr9	42842830	42844143	Downstream
chr9	42869624	42870612	Intron
chr9	42882509	42883597	Exon
chr9	42906021	42907310	Distal Intergenic

chr9	42910089	42910916	Distal Intergenic
chr9	42913144	42913726	Distal Intergenic
chr9	43093289	43093832	Exon
chr9	43108377	43109111	Intron
chr9	43116678	43116984	Intron
chr9	43117114	43117389	Intron
chr9	43117474	43117983	Intron
chr9	43118299	43118541	Intron
chr9	43119827	43120056	Intron
chr9	43120157	43121692	Exon
chr9	43122146	43122544	Intron
chr9	43122693	43122945	Intron
chr9	43123069	43123994	Intron
chr9	43124087	43125138	Exon
chr9	43125354	43125589	Exon
chr9	43126032	43127788	Intron
chr9	43133509	43135234	Promoter
chr9	43165406	43167782	Distal Intergenic
chr9	43349871	43350804	Distal Intergenic
chr9	43476313	43477495	Distal Intergenic
chr9	43481355	43482604	Distal Intergenic
chr9	43482643	43483210	Distal Intergenic
chr9	43487469	43490660	Distal Intergenic
chr9	43527559	43529549	Distal Intergenic
chr9	43552913	43554520	Distal Intergenic
chr9	43554778	43555147	Distal Intergenic
chr9	43557410	43558335	Distal Intergenic
chr9	43558497	43559171	Distal Intergenic
chr9	43561288	43561504	Distal Intergenic
chr9	43563869	43564213	Distal Intergenic
chr9	43564907	43565198	Distal Intergenic
chr9	43565246	43566628	Distal Intergenic
chr9	43579611	43582984	Distal Intergenic
chr9	43583040	43583796	Distal Intergenic
chr9	43583837	43584909	Distal Intergenic
chr9	43585008	43586823	Distal Intergenic
chr9	43590103	43590400	Distal Intergenic
chr9	43591142	43591547	Distal Intergenic
chr9	43591596	43592366	Distal Intergenic
chr9	43592486	43593788	Distal Intergenic
chr9	43595027	43597333	Distal Intergenic
chr9	43603763	43604631	Distal Intergenic
chr9	43604794	43605750	Distal Intergenic
chr9	43607874	43608582	Distal Intergenic

chr9	43609058	43610341	Distal Intergenic
chr9	43658932	43659367	Distal Intergenic
chr9	43665666	43667124	Distal Intergenic
chr9	43681928	43682954	Distal Intergenic
chr9	43848514	43848963	Intron
chr9	43920997	43921231	3' UTR
chr9	43921313	43921585	3' UTR
chr9	43943362	43944606	Distal Intergenic
chr9	43948585	43948927	Distal Intergenic
chr9	44006035	44006354	Distal Intergenic
chr9	44006491	44006818	Distal Intergenic
chr9	44007064	44007302	Distal Intergenic
chr9	44007358	44008068	Distal Intergenic
chr9	44008256	44008459	Distal Intergenic
chr9	44012819	44013187	Distal Intergenic
chr9	44015546	44016256	Distal Intergenic
chr9	44040793	44041612	Distal Intergenic
chr9	44075907	44076214	Distal Intergenic
chr9	44076415	44077162	Distal Intergenic
chr9	44077942	44078181	Distal Intergenic
chr9	44134311	44134854	Distal Intergenic
chr9	44141926	44142553	Distal Intergenic
chr9	44142873	44144415	Distal Intergenic
chr9	44151631	44151833	Distal Intergenic
chr9	44214805	44215034	Distal Intergenic
chr9	44215086	44215388	Distal Intergenic
chr9	44215435	44216495	Distal Intergenic
chr9	44286129	44287742	Distal Intergenic
chr9	44288378	44288849	Distal Intergenic
chr9	44290545	44291027	Distal Intergenic
chr9	44291505	44292496	Distal Intergenic
chr9	44292833	44293415	Distal Intergenic
chr9	44293563	44294611	Distal Intergenic
chr9	44294699	44297646	Distal Intergenic
chr9	44302178	44304184	Distal Intergenic
chr9	44305142	44305997	Distal Intergenic
chr9	44306059	44306887	Distal Intergenic
chr9	44307264	44307861	Distal Intergenic
chr9	44308264	44308576	Distal Intergenic
chr9	44309803	44310057	Distal Intergenic
chr9	44407306	44407518	Distal Intergenic
chr9	44411730	44412272	Distal Intergenic
chr9	44412425	44412637	Distal Intergenic
chr9	44413126	44414036	Distal Intergenic

chr9	44524625	44525579	Distal Intergenic
chr9	44527075	44528049	Distal Intergenic
chr9	44528232	44528496	Distal Intergenic
chr9	44728671	44729597	Distal Intergenic
chr9	44773311	44773705	Distal Intergenic
chr9	44773826	44774241	Distal Intergenic
chr9	44835470	44836470	Distal Intergenic
chr9	44850216	44851442	Exon
chr9	44854495	44854948	Distal Intergenic
chr9	44863172	44863432	Distal Intergenic
chr9	44863615	44864129	Distal Intergenic
chr9	44867700	44869358	Distal Intergenic
chr9	45008276	45008798	Distal Intergenic
chr9	45008856	45009067	Distal Intergenic
chr9	45012149	45012705	Distal Intergenic
chr9	45012906	45013195	Distal Intergenic
chr9	45013252	45013536	Distal Intergenic
chr9	45017842	45018278	Distal Intergenic
chr9	45145328	45145854	Distal Intergenic
chr9	45636740	45636970	Distal Intergenic
chr9	45653945	45654843	Distal Intergenic
chr9	45654953	45655308	Distal Intergenic
chr9	45762252	45762451	Distal Intergenic
chr9	45762490	45763288	Distal Intergenic
chr9	45791832	45792533	Distal Intergenic
chr9	45792834	45793328	Distal Intergenic
chr9	45854869	45855819	Distal Intergenic
chr9	45856896	45857110	Distal Intergenic
chr9	45876792	45877009	Distal Intergenic
chr9	45877102	45877848	Distal Intergenic
chr9	45891402	45891895	Distal Intergenic
chr9	45928313	45929572	Distal Intergenic
chr9	46090824	46091023	Distal Intergenic
chr9	46478212	46478797	Distal Intergenic
chr9	46667637	46668744	Distal Intergenic
chr9	46769198	46769493	Distal Intergenic
chr9	46769700	46769962	Distal Intergenic
chr9	46830015	46831206	Promoter
chr9	46831311	46831622	Intron
chr9	46870073	46870881	Distal Intergenic
chr9	47170423	47171330	Distal Intergenic
chr9	47360833	47361170	Distal Intergenic
chr9	47361227	47361711	Distal Intergenic
chr9	47397111	47398951	Distal Intergenic

chr9	47427058	47428079	Distal Intergenic
chr9	47433840	47434132	Distal Intergenic
chr9	47510737	47511201	Distal Intergenic
chr9	47511705	47512267	Distal Intergenic
chr9	48215836	48216543	Distal Intergenic
chr9	48302892	48303376	Distal Intergenic
chr9	49234181	49235810	Distal Intergenic
chr9	49622106	49622428	Distal Intergenic
chr9	49692603	49693002	Distal Intergenic
chr9	49707317	49707534	Distal Intergenic
chr9	49731512	49732419	Distal Intergenic
chr9	49732562	49732807	Distal Intergenic
chr9	49780572	49781553	Distal Intergenic
chr9	49783964	49784948	Distal Intergenic
chr9	49788740	49789757	Distal Intergenic
chr9	49808302	49810042	Distal Intergenic
chr9	49813806	49814806	Distal Intergenic
chr9	49900270	49900937	Distal Intergenic
chr9	50278266	50280155	Distal Intergenic
chr9	50280937	50281936	Distal Intergenic
chr9	50284983	50285430	Distal Intergenic
chr9	50302828	50303192	Distal Intergenic
chr9	50304322	50304912	Distal Intergenic
chr9	50328772	50329080	Distal Intergenic
chr9	50329416	50329631	Distal Intergenic
chr9	50399172	50399414	Distal Intergenic
chr9	50412212	50412657	Distal Intergenic
chr9	50424829	50425039	Distal Intergenic
chr9	50425184	50425591	Distal Intergenic
chr9	50489720	50490053	Distal Intergenic
chr9	50494678	50496045	Distal Intergenic
chr9	50549398	50550355	Distal Intergenic
chr9	50554026	50554977	Distal Intergenic
chr9	50655052	50655501	Distal Intergenic
chr9	50660385	50661919	Distal Intergenic
chr9	50662095	50662901	Distal Intergenic
chr9	50708304	50709179	Distal Intergenic
chr9	50864863	50865144	Distal Intergenic
chr9	50866198	50866704	Distal Intergenic
chr9	50866762	50867573	Distal Intergenic
chr9	50913584	50914547	Distal Intergenic
chr9	50977096	50978791	Distal Intergenic
chr9	50983608	50984441	Distal Intergenic
chr9	51076920	51078756	Distal Intergenic

chr9	51772025	51772232	Distal Intergenic
chr9	51856250	51856712	Distal Intergenic
chr9	51857199	51857412	Distal Intergenic
chr9	51975350	51975651	Distal Intergenic
chr9	51975828	51976367	Distal Intergenic
chr9	51980845	51981337	Distal Intergenic
chr9	51982601	51983736	Distal Intergenic
chr9	51988545	51990413	Distal Intergenic
chr9	53071925	53072927	Distal Intergenic
chr9	53086981	53087543	Distal Intergenic
chr9	53095093	53095995	Distal Intergenic
chr9	53098054	53098745	Distal Intergenic
chr9	53192048	53192376	Distal Intergenic
chr9	53192530	53192992	Distal Intergenic
chr9	53216922	53217382	Distal Intergenic
chr9	54414515	54415563	Distal Intergenic
chr9	54460981	54461418	Distal Intergenic
chr9	54490089	54491445	Distal Intergenic
chr9	54493076	54494080	Distal Intergenic
chr9	54494857	54495476	Distal Intergenic
chr9	54496871	54497378	Distal Intergenic
chr9	54520554	54521479	Distal Intergenic
chr9	54555293	54556024	Distal Intergenic
chr9	54570306	54570770	Distal Intergenic
chr9	54580643	54580844	Distal Intergenic
chr9	54580920	54582319	Distal Intergenic
chr9	54642763	54643204	Distal Intergenic
chr9	54650165	54651776	Distal Intergenic
chr9	54756647	54757411	Distal Intergenic
chr9	54757487	54757911	Distal Intergenic
chr9	54798834	54799353	Distal Intergenic
chr9	54962531	54962786	Distal Intergenic
chr9	54962932	54963665	Distal Intergenic
chr9	54984400	54985234	Distal Intergenic
chr9	55258270	55258471	Distal Intergenic
chr9	55258687	55259047	Distal Intergenic
chr9	55324215	55324414	Distal Intergenic
chr9	55787976	55788971	Distal Intergenic
chr9	55789010	55789213	Distal Intergenic
chr9	55907153	55907354	Distal Intergenic
chr9	55937013	55937961	Distal Intergenic
chr9	55938018	55939445	Distal Intergenic
chr9	55947848	55949192	Distal Intergenic
chr9	55949312	55949577	Distal Intergenic

chr9	55951638	55951837	Distal Intergenic
chr9	55953897	55956306	Distal Intergenic
chr9	56007986	56008542	Distal Intergenic
chr9	56015125	56016671	Distal Intergenic
chr9	56025069	56026268	Distal Intergenic
chr9	56027106	56028885	Distal Intergenic
chr9	56044348	56045199	Distal Intergenic
chr9	56112659	56113006	Distal Intergenic
chr9	56113071	56113639	Distal Intergenic
chr9	56182211	56182462	Distal Intergenic
chr9	56218436	56219133	Distal Intergenic
chr9	56240923	56241373	Distal Intergenic
chr9	56353358	56353865	Distal Intergenic
chr9	56354019	56354720	Distal Intergenic
chr9	56356684	56358124	Distal Intergenic
chr9	56362894	56363377	Distal Intergenic
chr9	56392934	56393242	Distal Intergenic
chr9	56400426	56400660	Distal Intergenic
chr9	56706927	56707325	Distal Intergenic
chr9	56708617	56709075	Distal Intergenic
chr9	56748295	56749384	Distal Intergenic
chr9	56749488	56749875	Distal Intergenic
chr9	56754675	56755675	Distal Intergenic
chr9	56755734	56756305	Distal Intergenic
chr9	56758358	56759647	Distal Intergenic
chr9	56760021	56760220	Distal Intergenic
chr9	56760827	56762765	Distal Intergenic
chr9	56766352	56767127	Distal Intergenic
chr9	56767234	56767522	Distal Intergenic
chr9	56771835	56772970	Distal Intergenic
chr9	56774893	56775923	Distal Intergenic
chr9	56798297	56798640	Distal Intergenic
chr9	56799104	56799359	Distal Intergenic
chr9	56816931	56818360	Distal Intergenic
chr9	56842888	56843676	Distal Intergenic
chr9	56843730	56844244	Distal Intergenic
chr9	56922640	56922857	Distal Intergenic
chr9	56923258	56923556	Distal Intergenic
chr9	56978675	56979004	Distal Intergenic
chr9	57051542	57051803	Distal Intergenic
chr9	57369621	57369950	Distal Intergenic
chr9	57385103	57385345	Distal Intergenic
chr9	57462478	57463116	Distal Intergenic
chr9	57466533	57467328	Distal Intergenic

chr9	57473391	57474043	Distal Intergenic
chr9	57488417	57488910	Distal Intergenic
chr9	57612083	57612962	Distal Intergenic
chr9	57758457	57758706	Distal Intergenic
chr9	57903653	57904560	Distal Intergenic
chr9	57979121	57979328	Distal Intergenic
chr9	58095105	58097953	Distal Intergenic
chr9	58101045	58101339	Distal Intergenic
chr9	58103333	58104586	Distal Intergenic
chr9	58283882	58285452	Distal Intergenic
chr9	58290421	58290765	Distal Intergenic
chr9	58295807	58296183	Distal Intergenic
chr9	58301571	58302267	Distal Intergenic
chr9	58395224	58395458	Distal Intergenic
chr9	58396259	58398013	Distal Intergenic
chr9	58401487	58403654	Distal Intergenic
chr9	58428861	58429264	Distal Intergenic
chr9	58429347	58429933	Distal Intergenic
chr9	58511073	58511283	Distal Intergenic
chr9	58513032	58514018	Distal Intergenic
chr9	58554085	58554400	Distal Intergenic
chr9	59317315	59317645	Distal Intergenic
chr9	59333128	59333944	Distal Intergenic
chr9	59334723	59334970	Distal Intergenic
chr9	59469763	59470058	Distal Intergenic
chr9	59470096	59471181	Distal Intergenic
chr9	59488827	59489491	Distal Intergenic
chr9	59500560	59502017	Distal Intergenic
chr9	59502112	59502603	Distal Intergenic
chr9	59503216	59504330	Distal Intergenic
chr9	59504456	59504841	Distal Intergenic
chr9	59505062	59507695	Distal Intergenic
chr9	59507871	59508070	Distal Intergenic
chr9	59528574	59529675	Distal Intergenic
chr9	59529755	59529971	Distal Intergenic
chr9	59534565	59536088	Distal Intergenic
chr9	59538539	59541542	Distal Intergenic
chr9	59554252	59555817	Distal Intergenic
chr9	59580985	59581749	Distal Intergenic
chr9	59581955	59582173	Distal Intergenic
chr9	60535579	60535809	Distal Intergenic
chr9	60685445	60685992	Distal Intergenic
chr9	61128917	61129332	Distal Intergenic
chr9	61129511	61131584	Distal Intergenic

chr9	61134590	61134793	Distal Intergenic
chr9	61169505	61170282	Distal Intergenic
chr9	61170359	61170765	Distal Intergenic
chr9	61406441	61406644	Distal Intergenic
chr9	61406845	61407189	Distal Intergenic
chr9	61517330	61517615	Distal Intergenic
chr9	61759340	61759591	Distal Intergenic
chr9	61760781	61760999	Distal Intergenic
chr9	61794762	61795845	Distal Intergenic
chr9	61800142	61800402	Distal Intergenic
chr9	61810735	61813837	Distal Intergenic
chr9	61813939	61814374	Distal Intergenic
chr9	61846018	61846222	Distal Intergenic
chr9	61847242	61847472	Distal Intergenic
chr9	61847603	61848594	Distal Intergenic
chr9	62658432	62658839	Distal Intergenic
chr9	62659045	62659541	Distal Intergenic
chr9	62664909	62666558	Distal Intergenic
chr9	62675788	62676128	Distal Intergenic
chr9	62885174	62885402	Distal Intergenic
chr9	63427874	63428337	Distal Intergenic
chr9	63455708	63456226	Distal Intergenic
chr9	63458805	63459013	Distal Intergenic
chr9	63494656	63495047	Distal Intergenic
chr9	63495489	63495785	Distal Intergenic
chr9	63522442	63523355	Distal Intergenic
chr9	63525404	63525784	Distal Intergenic
chr9	63569096	63569674	Distal Intergenic
chr9	63573372	63573774	Distal Intergenic
chr9	63593798	63594071	Distal Intergenic
chr9	63662617	63663492	Distal Intergenic
chr9	63684628	63684828	Distal Intergenic
chr9	63840239	63841159	Distal Intergenic
chr9	63865700	63866915	Distal Intergenic
chr9	63869599	63869798	Distal Intergenic
chr9	63897365	63898817	Distal Intergenic
chr9	64020498	64021540	Distal Intergenic
chr9	64031922	64032455	Distal Intergenic
chr9	64050494	64050908	Distal Intergenic
chr9	64051230	64051530	Distal Intergenic
chr9	64064371	64064771	Distal Intergenic
chr9	64065754	64066108	Distal Intergenic
chr9	64100418	64100681	Distal Intergenic
chr9	64129453	64129836	Distal Intergenic

chr9	64188138	64189328	Distal Intergenic
chr9	64319051	64319257	Distal Intergenic
chr9	64548807	64552069	Distal Intergenic
chr9	64582091	64582617	Distal Intergenic
chr9	64583346	64584563	Distal Intergenic
chr9	64692829	64694224	Distal Intergenic
chr9	64808625	64808995	Distal Intergenic
chr9	64836055	64836310	Distal Intergenic
chr9	64841906	64842725	Distal Intergenic
chr9	64866135	64866604	Distal Intergenic
chr9	64867661	64867869	Distal Intergenic
chr9	65049083	65049833	Distal Intergenic
chr9	65139311	65140332	Distal Intergenic
chr9	65141108	65141829	Distal Intergenic
chr9	65193556	65194069	Distal Intergenic
chr9	65340224	65340821	Distal Intergenic
chr9	65389147	65389920	Distal Intergenic
chr9	65397274	65399607	Distal Intergenic
chr9	65422266	65424009	Distal Intergenic
chr9	65484953	65485325	Downstream
chr9	65567747	65568250	Distal Intergenic
chr9	65624461	65625604	Distal Intergenic
chr9	65674341	65675091	Distal Intergenic
chr9	65676015	65676262	Distal Intergenic
chr9	65676834	65677173	Distal Intergenic
chr9	65756386	65756632	Distal Intergenic
chr9	65757038	65757679	Distal Intergenic
chr9	65757726	65758176	Distal Intergenic
chr9	65972331	65972564	Distal Intergenic
chr9	66054586	66055033	Distal Intergenic
chr9	66065420	66066177	Distal Intergenic
chr9	66073482	66074708	Distal Intergenic
chr9	66075390	66075682	Distal Intergenic
chr9	66075901	66077286	Distal Intergenic
chr9	66088405	66088736	Distal Intergenic
chr9	66364664	66364876	Distal Intergenic
chr9	66439558	66440346	Distal Intergenic
chr9	66440487	66440774	Distal Intergenic
chr9	66468236	66469449	Exon
chr9	66469559	66470101	Distal Intergenic
chr9	66470456	66470732	Distal Intergenic
chr9	66568367	66568597	Distal Intergenic
chr9	66578829	66579040	Distal Intergenic
chr9	66763748	66764354	Distal Intergenic

chr9	66793398	66794436	Distal Intergenic
chr9	66794488	66794875	Distal Intergenic
chr9	66823241	66823786	Distal Intergenic
chr9	66836139	66838991	Distal Intergenic
chr9	66872487	66872694	Distal Intergenic
chr9	66872970	66873169	Distal Intergenic
chr9	66873210	66873670	Distal Intergenic
chr9	66884438	66885457	Distal Intergenic
chr9	66885656	66886086	Distal Intergenic
chr9	66888734	66888987	Distal Intergenic
chr9	66890925	66891124	Distal Intergenic
chr9	66893012	66893764	Distal Intergenic
chr9	66896177	66896663	Distal Intergenic
chr9	66929405	66930024	Intron
chr9	66952422	66954224	Distal Intergenic
chr9	66954334	66954558	Distal Intergenic
chr9	66955746	66956040	Distal Intergenic
chr9	67007216	67007415	Distal Intergenic
chr9	67008030	67008300	Distal Intergenic
chr9	67013149	67013781	Distal Intergenic
chr9	67055720	67055976	Distal Intergenic
chr9	67056337	67057465	Distal Intergenic
chr9	67071242	67072183	Distal Intergenic
chr9	67178890	67179229	Distal Intergenic
chr9	67724795	67725263	Distal Intergenic
chr9	67725310	67725548	Distal Intergenic
chr9	67802578	67804129	Distal Intergenic
chr9	68635226	68636179	Distal Intergenic
chr9	68636240	68636713	Distal Intergenic
chr9	68636911	68637155	Distal Intergenic
chr9	69245832	69246031	Exon
chr9	69278995	69280312	Distal Intergenic
chr9	69290060	69291382	Distal Intergenic
chr9	69301486	69308316	Distal Intergenic
chr9	69310098	69310370	Distal Intergenic
chr9	69311170	69311370	Distal Intergenic
chr9	69311422	69311621	Distal Intergenic
chr9	69313405	69313658	Distal Intergenic
chr9	69313696	69317339	Distal Intergenic
chr9	69319204	69319692	Distal Intergenic
chr9	69319743	69321189	Distal Intergenic
chr9	69321226	69321516	Distal Intergenic
chr9	69895476	69896917	Distal Intergenic
chr9	70055271	70055548	Distal Intergenic

chr9	70055956	70056269	Distal Intergenic
chr9	70056669	70058118	Distal Intergenic
chr9	70058271	70058658	Distal Intergenic
chr9	70064981	70065314	Distal Intergenic
chr9	70065635	70066023	Distal Intergenic
chr9	70075265	70076131	Distal Intergenic
chr9	70077187	70078157	Distal Intergenic
chr9	70096881	70097132	Distal Intergenic
chr9	70100581	70100780	Distal Intergenic
chr9	70100835	70101742	Distal Intergenic
chr9	70104602	70105903	Distal Intergenic
chr9	70108400	70109753	Distal Intergenic
chr9	70138350	70138920	Distal Intergenic
chr9	70148264	70150315	Distal Intergenic
chr9	70268937	70269384	Distal Intergenic
chr9	70357613	70358839	Distal Intergenic
chr9	70359669	70360011	Distal Intergenic
chr9	70423517	70423771	Downstream
chr9	70477678	70477877	Promoter
chr9	70478900	70479397	Intron
chr9	70484421	70485203	Exon
chr9	70528005	70528205	Distal Intergenic
chr9	70561101	70561322	Distal Intergenic
chr9	70580520	70581537	Distal Intergenic
chr9	70599024	70600032	Distal Intergenic
chr9	70600098	70600453	Distal Intergenic
chr9	70609148	70609416	Distal Intergenic
chr9	70682108	70683539	Distal Intergenic
chr9	70685517	70687367	Distal Intergenic
chr9	70689771	70689977	Distal Intergenic
chr9	70690035	70690320	Distal Intergenic
chr9	70735826	70737588	Distal Intergenic
chr9	70742074	70742482	Distal Intergenic
chr9	70742714	70743895	Distal Intergenic
chr9	70772454	70776074	Distal Intergenic
chr9	71003364	71003583	Intron
chr9	71332746	71333747	Intron
chr9	71418744	71419483	Intron
chr9	71421810	71422035	Intron
chr9	71868892	71870063	3' UTR
chr9	71923305	71925404	Distal Intergenic
chr9	71948566	71949877	Intron
chr9	71958991	71959210	Intron
chr9	72123088	72123630	Intron

chr9	72378622	72379867	Distal Intergenic
chr9	72380130	72380453	Distal Intergenic
chr9	72510725	72511345	Intron
chr9	72549439	72550116	Distal Intergenic
chr9	72887702	72888060	Intron
chr9	72915588	72916761	Intron
chr9	72960793	72961200	Promoter
chr9	72961442	72961731	Promoter
chr9	73654168	73654544	Intron
chr9	73753533	73753791	Intron
chr9	74866724	74868312	3' UTR
chr9	74870747	74871197	Distal Intergenic
chr9	74871279	74871985	Distal Intergenic
chr9	74885424	74886437	Distal Intergenic
chr9	74886557	74887822	Distal Intergenic
chr9	74932817	74933666	Intron
chr9	74974751	74974996	Promoter
chr9	74975132	74975453	Promoter
chr9	75042105	75042304	Distal Intergenic
chr9	75043042	75044951	Distal Intergenic
chr9	75200134	75201822	Intron
chr9	75214008	75214371	Intron
chr9	75226433	75226639	Intron
chr9	75226838	75228175	Intron
chr9	75232574	75234988	Intron
chr9	75255410	75255688	Intron
chr9	75255855	75257425	Intron
chr9	75257566	75257815	Intron
chr9	75258123	75258353	Intron
chr9	75269536	75271329	Intron
chr9	75271431	75271917	Intron
chr9	75273757	75273956	Intron
chr9	75275192	75275546	Intron
chr9	75275592	75275942	Intron
chr9	75312675	75312905	Intron
chr9	75313253	75313912	Intron
chr9	75313988	75314219	Intron
chr9	75377417	75377723	Intron
chr9	75389226	75389536	Intron
chr9	75389673	75389919	Intron
chr9	75406794	75407104	Exon
chr9	75858025	75858363	Distal Intergenic
chr9	75862391	75863598	Distal Intergenic
chr9	75871214	75872631	Distal Intergenic

chr9	76359377	76359623	Distal Intergenic
chr9	76362737	76363899	Distal Intergenic
chr9	76370488	76371833	Distal Intergenic
chr9	76386738	76386971	Distal Intergenic
chr9	76387734	76390490	Distal Intergenic
chr9	76401380	76402295	Distal Intergenic
chr9	76413357	76414916	Distal Intergenic
chr9	76415148	76415419	Distal Intergenic
chr9	76500970	76501273	Distal Intergenic
chr9	76651718	76652173	Distal Intergenic
chr9	76652568	76653323	Distal Intergenic
chr9	77312338	77312744	Distal Intergenic
chr9	77393635	77394044	Intron
chr9	77434327	77435906	Exon
chr9	77549298	77549497	Distal Intergenic
chr9	77549541	77551231	Distal Intergenic
chr9	77551316	77552539	Distal Intergenic
chr9	77558678	77559905	Downstream
chr9	77592308	77592893	Intron
chr9	77602818	77603135	Intron
chr9	77758629	77759674	Intron
chr9	77833935	77834355	Distal Intergenic
chr9	78023788	78024843	Distal Intergenic
chr9	78026576	78027058	Distal Intergenic
chr9	78031696	78032496	Distal Intergenic
chr9	78038374	78039951	Distal Intergenic
chr9	78040011	78040257	Distal Intergenic
chr9	78040447	78040744	Distal Intergenic
chr9	78041038	78041239	Distal Intergenic
chr9	78059650	78061041	Distal Intergenic
chr9	78077614	78078516	Distal Intergenic
chr9	78103266	78104699	Distal Intergenic
chr9	78118714	78119590	Intron
chr9	78129900	78130113	Intron
chr9	78324455	78325319	Intron
chr9	78325443	78325678	Intron
chr9	78327400	78327668	Intron
chr9	78328087	78328320	Intron
chr9	78328522	78328782	Intron
chr9	78329805	78330258	Intron
chr9	78350669	78352619	Distal Intergenic
chr9	78375406	78375792	Distal Intergenic
chr9	78470042	78470269	Distal Intergenic
chr9	78470432	78471874	Distal Intergenic

chr9	78569141	78569468	Intron
chr9	78919542	78919780	Intron
chr9	79013268	79014072	Promoter
chr9	79014117	79014363	Promoter
chr9	79063575	79063802	Intron
chr9	79064167	79064407	Intron
chr9	79377836	79379191	Promoter
chr9	79437147	79437402	Intron
chr9	79496595	79497179	Intron
chr9	79497221	79497524	Intron
chr9	79528977	79529398	Distal Intergenic
chr9	79556448	79557425	Distal Intergenic
chr9	79561255	79563041	Distal Intergenic
chr9	79607213	79607849	Distal Intergenic
chr9	79914701	79916267	Intron
chr9	80007488	80007689	Intron
chr9	80581838	80582109	Intron
chr9	80582363	80582621	Intron
chr9	80707899	80709051	Distal Intergenic
chr9	80997348	80997610	Distal Intergenic
chr9	81019881	81020321	Distal Intergenic
chr9	82199838	82200041	Intron
chr9	82200088	82200707	Intron
chr9	82860265	82861642	Distal Intergenic
chr9	82862173	82862413	Distal Intergenic
chr9	82865083	82865282	Distal Intergenic
chr9	82866818	82867661	Distal Intergenic
chr9	83126609	83126869	Distal Intergenic
chr9	83126920	83127127	Distal Intergenic
chr9	83236280	83236822	Distal Intergenic
chr9	83241354	83241761	Distal Intergenic
chr9	83242728	83243763	Distal Intergenic
chr9	83442147	83442904	Distal Intergenic
chr9	83456431	83460117	Distal Intergenic
chr9	83460203	83460500	Distal Intergenic
chr9	83461510	83463294	Distal Intergenic
chr9	83463528	83463807	Distal Intergenic
chr9	83464568	83465956	Distal Intergenic
chr9	83509716	83510680	Distal Intergenic
chr9	83649990	83650225	Distal Intergenic
chr9	83715757	83717814	Distal Intergenic
chr9	83724552	83724840	Distal Intergenic
chr9	83776095	83776464	Distal Intergenic
chr9	83776553	83777041	Distal Intergenic

chr9	85391054	85391503	Distal Intergenic
chr9	85510296	85510903	Distal Intergenic
chr9	85642593	85642794	Intron
chr9	85643030	85643598	Intron
chr9	85714615	85714935	Distal Intergenic
chr9	85736020	85737538	Distal Intergenic
chr9	86340895	86341113	Distal Intergenic
chr9	86361147	86361376	Intron
chr9	86517989	86519369	Exon
chr9	86521488	86521792	Intron
chr9	86521887	86522086	Intron
chr9	86698257	86698823	Intron
chr9	86894738	86895187	Exon
chr9	86897958	86898250	Intron
chr9	86898356	86898870	Intron
chr9	87358180	87359871	Intron
chr9	88220584	88221358	Intron
chr9	88267881	88268345	Intron
chr9	88334050	88334417	Intron
chr9	88334513	88334748	Intron
chr9	88375893	88376172	Distal Intergenic
chr9	88392437	88393240	Distal Intergenic
chr9	88414631	88414912	Distal Intergenic
chr9	88416723	88416934	Distal Intergenic
chr9	88417323	88417691	Distal Intergenic
chr9	88434596	88434877	Intron
chr9	88614648	88614907	Intron
chr9	88614990	88615202	Intron
chr9	89930209	89932600	Distal Intergenic
chr9	89963414	89964469	Distal Intergenic
chr9	89964517	89965039	Distal Intergenic
chr9	90008260	90008586	Distal Intergenic
chr9	90008768	90008967	Distal Intergenic
chr9	90108648	90109046	Distal Intergenic
chr9	90110453	90110771	Distal Intergenic
chr9	90110829	90111073	Distal Intergenic
chr9	90111112	90111999	Promoter
chr9	90130296	90130897	Intron
chr9	90135182	90135780	Intron
chr9	90135933	90136354	Intron
chr9	90143773	90145625	Intron
chr9	90156755	90156963	Intron
chr9	90157092	90158542	Intron
chr9	90246384	90246658	Intron

chr9	92436621	92436820	Distal Intergenic
chr9	92437423	92437694	Distal Intergenic
chr9	93026525	93026903	Distal Intergenic
chr9	94329242	94329627	Intron
chr9	95093156	95094254	Intron
chr9	95411747	95412057	Exon
chr9	95412449	95412718	Intron
chr9	95414502	95414939	Exon
chr9	95543542	95543744	Distal Intergenic
chr9	95572745	95572952	Promoter
chr9	95758386	95758690	Intron
chr9	95904636	95905304	Distal Intergenic
chr9	96102983	96103182	Intron
chr9	96263981	96264225	Intron
chr9	96281494	96282734	Intron
chr9	96284057	96284638	Intron
chr9	96284836	96285036	Intron
chr9	96313059	96313998	Intron
chr9	96315682	96316027	Intron
chr9	96316459	96317333	Intron
chr9	96317370	96317590	Intron
chr9	96606564	96606832	Distal Intergenic
chr9	96614584	96614917	Distal Intergenic
chr9	96614976	96615377	Distal Intergenic
chr9	96621031	96622031	Distal Intergenic
chr9	96651889	96652359	Distal Intergenic
chr9	96652451	96653885	Distal Intergenic
chr9	96655718	96656487	Distal Intergenic
chr9	96656719	96656980	Distal Intergenic
chr9	96669987	96670558	Distal Intergenic
chr9	96670600	96670877	Distal Intergenic
chr9	96672923	96673500	Distal Intergenic
chr9	96673681	96675631	Distal Intergenic
chr9	96681343	96681542	Distal Intergenic
chr9	96681592	96682988	Distal Intergenic
chr9	96683030	96684830	Distal Intergenic
chr9	96689465	96691365	Distal Intergenic
chr9	96691414	96691656	Distal Intergenic
chr9	96700430	96701281	Distal Intergenic
chr9	96761029	96761681	Distal Intergenic
chr9	96761802	96762031	Distal Intergenic
chr9	96763319	96764036	Distal Intergenic
chr9	97003863	97004568	Distal Intergenic
chr9	97176290	97176591	Promoter

chr9	97535399	97535621	Exon
chr9	97535724	97535952	Intron
chr9	98197804	98198174	Distal Intergenic
chr9	98214707	98215063	Intron
chr9	98238273	98239317	Exon
chr9	98333149	98335010	Distal Intergenic
chr9	99030994	99031363	Intron
chr9	99031902	99032474	Intron
chr9	99032597	99033032	Intron
chr9	99037598	99037896	Intron
chr9	99066024	99067930	Distal Intergenic
chr9	99529972	99530171	Intron
chr9	99530545	99533552	Intron
chr9	99620458	99621047	Intron
chr9	100505575	100506891	Distal Intergenic
chr9	100542229	100542578	Distal Intergenic
chr9	100542724	100543293	Distal Intergenic
chr9	100544995	100545433	Distal Intergenic
chr9	100979696	100981048	Intron
chr9	101077623	101077843	Intron
chr9	101131400	101131697	Intron
chr9	101131906	101132135	Intron
chr9	101145527	101146980	Intron
chr9	101147137	101147351	Intron
chr9	101167837	101168872	Exon
chr9	101170666	101173280	Intron
chr9	101191115	101193036	Intron
chr9	102535979	102536216	Intron
chr9	102536393	102536630	Intron
chr9	102536712	102537154	Intron
chr9	102540408	102540654	Intron
chr9	102575429	102575840	Intron
chr9	102576098	102576904	Intron
chr9	102639710	102639941	Distal Intergenic
chr9	102639987	102640194	Distal Intergenic
chr9	102640388	102640732	Distal Intergenic
chr9	102663467	102666545	Intron
chr9	102679329	102682071	Intron
chr9	102682128	102683180	Intron
chr9	102683441	102683886	Intron
chr9	102683928	102684358	Intron
chr9	102685118	102685894	Intron
chr9	102707615	102708397	Intron
chr9	102708912	102709210	Intron

chr9	102744735	102745376	3' UTR
chr9	102745816	102746400	Intron
chr9	102874770	102875079	Intron
chr9	102963397	102964424	Intron
chr9	102975010	102975313	Intron
chr9	102984146	102984345	Intron
chr9	103207555	103207832	Intron
chr9	103207869	103208095	Intron
chr9	103274677	103275172	Intron
chr9	103278603	103278876	Intron
chr9	103278928	103279667	Exon
chr9	103987669	103987887	Intron
chr9	103988001	103990081	Intron
chr9	104057427	104057691	Intron
chr9	104154009	104154493	Intron
chr9	104154621	104155006	Intron
chr9	104156756	104157221	Intron
chr9	104239755	104240710	Intron
chr9	104240794	104241048	Intron
chr9	104263762	104264349	Distal Intergenic
chr9	105360912	105361486	Distal Intergenic
chr9	105362815	105363104	Distal Intergenic
chr9	105363748	105364395	Distal Intergenic
chr9	105397738	105397943	Distal Intergenic
chr9	105595193	105595498	Distal Intergenic
chr9	105599395	105599677	Distal Intergenic
chr9	105599770	105600210	Distal Intergenic
chr9	105604564	105604984	Distal Intergenic
chr9	105905144	105905409	Exon
chr9	106074450	106074672	Intron
chr9	106177252	106177536	Distal Intergenic
chr9	106261871	106262101	Distal Intergenic
chr9	106263292	106263544	Distal Intergenic
chr9	106267871	106268261	Distal Intergenic
chr9	106268907	106269896	Distal Intergenic
chr9	106271550	106274740	Distal Intergenic
chr9	106277192	106277414	Distal Intergenic
chr9	106279152	106281904	Distal Intergenic
chr9	106293460	106293879	Distal Intergenic
chr9	106294032	106294868	Distal Intergenic
chr9	106295218	106295534	Distal Intergenic
chr9	106296681	106296964	Distal Intergenic
chr9	106314244	106314597	Distal Intergenic
chr9	106314772	106315974	Distal Intergenic

chr9	106316062	106316311	Distal Intergenic
chr9	106378768	106378968	Distal Intergenic
chr9	106724440	106724647	Distal Intergenic
chr9	106794586	106794949	Distal Intergenic
chr9	107181697	107181949	Distal Intergenic
chr9	107191160	107191886	Distal Intergenic
chr9	107191958	107192184	Distal Intergenic
chr9	107200933	107201132	Distal Intergenic
chr9	107207702	107207924	Distal Intergenic
chr9	107458117	107458745	Distal Intergenic
chr9	107458787	107459144	Distal Intergenic
chr9	107459852	107460093	Distal Intergenic
chr9	107471021	107472549	Distal Intergenic
chr9	107477571	107479252	Distal Intergenic
chr9	107479482	107479715	Distal Intergenic
chr9	107481661	107482127	Distal Intergenic
chr9	107482440	107482771	Distal Intergenic
chr9	107535209	107535667	Promoter
chr9	107536174	107536412	Promoter
chr9	107536570	107536987	Promoter
chr9	107537328	107537579	Promoter
chr9	107603846	107604144	Intron
chr9	107605845	107606125	Exon
chr9	107606209	107606571	Exon
chr9	107609251	107609967	Intron
chr9	107610105	107610920	Intron
chr9	107611174	107611611	Intron
chr9	107672965	107673314	Intron
chr9	107673485	107673736	Intron
chr9	107809674	107813548	Distal Intergenic
chr9	107982741	107983342	Distal Intergenic
chr9	108039484	108039689	Intron
chr9	108166480	108167172	Intron
chr9	108167235	108167487	Intron
chr9	108169654	108170508	Intron
chr9	108174228	108175001	Intron
chr9	108207903	108208347	Distal Intergenic
chr9	108240624	108241453	Intron
chr9	108241652	108241964	Exon
chr9	108243945	108244268	Intron
chr9	108244574	108244868	Intron
chr9	108264864	108265305	Intron
chr9	108361472	108363392	5' UTR
chr9	108393191	108393945	Intron

chr9	108394172	108394473	Intron
chr9	108424028	108424241	Promoter
chr9	108847078	108847736	Distal Intergenic
chr9	108850248	108852908	Distal Intergenic
chr9	108854101	108855149	Distal Intergenic
chr9	108956776	108957018	Intron
chr9	108961409	108962512	Intron
chr9	108996326	108997132	Intron
chr9	108997218	108997620	Intron
chr9	108998609	108998994	Intron
chr9	108999102	108999313	Intron
chr9	109021497	109021778	Intron
chr9	109022128	109022730	Intron
chr9	109798303	109798564	Intron
chr9	109799273	109800365	Intron
chr9	110247267	110247941	3' UTR
chr9	110251548	110251942	Promoter
chr9	110251981	110253666	Promoter
chr9	110309904	110310376	Distal Intergenic
chr9	110378825	110379162	Distal Intergenic
chr9	110435418	110435723	Distal Intergenic
chr9	110435805	110436105	Distal Intergenic
chr9	110486548	110486747	Distal Intergenic
chr9	110486976	110487353	Distal Intergenic
chr9	110487525	110488053	Distal Intergenic
chr9	110583475	110583992	Distal Intergenic
chr9	110585821	110586270	Distal Intergenic
chr9	110586471	110587801	Distal Intergenic
chr9	110599324	110599930	Distal Intergenic
chr9	110600077	110600321	Distal Intergenic
chr9	110612210	110612505	Distal Intergenic
chr9	110782963	110784584	Distal Intergenic
chr9	111006324	111006625	Distal Intergenic
chr9	111006814	111007013	Distal Intergenic
chr9	111021253	111021724	Distal Intergenic
chr9	111034273	111035102	Distal Intergenic
chr9	111051454	111051838	Distal Intergenic
chr9	111058497	111058897	Distal Intergenic
chr9	111058935	111059236	Distal Intergenic
chr9	111066092	111066980	Distal Intergenic
chr9	111145859	111146261	Distal Intergenic
chr9	111174404	111174685	Distal Intergenic
chr9	111174873	111175383	Distal Intergenic
chr9	111489509	111489868	Distal Intergenic

chr9	111634116	111636471	Intron
chr9	111657527	111658182	Intron
chr9	112852910	112853228	Promoter
chr9	113287125	113288012	Intron
chr9	113578962	113580528	Distal Intergenic
chr9	113615075	113616644	Distal Intergenic
chr9	113616944	113617178	Distal Intergenic
chr9	113638237	113641280	Intron
chr9	114310028	114310235	Intron
chr9	115216820	115217164	Intron
chr9	116060158	116060398	Promoter
chr9	116061313	116063828	Promoter
chr9	116083287	116084181	Exon
chr9	116084435	116085078	Intron
chr9	116127261	116129019	Intron
chr9	116130894	116131522	Intron
chr9	116131998	116132957	3' UTR
chr9	116133063	116133486	3' UTR
chr9	116193143	116193389	Distal Intergenic
chr9	116220314	116220513	Intron
chr9	116220782	116221276	Intron
chr9	116222352	116223890	5' UTR
chr9	116228178	116228616	Intron
chr9	116229066	116229505	Intron
chr9	116238349	116238857	Intron
chr9	116244641	116245270	5' UTR
chr9	116251435	116251816	Intron
chr9	116251962	116252496	Intron
chr9	116277412	116278801	Intron
chr9	116278854	116279264	Intron
chr9	116281052	116281547	Intron
chr9	116281738	116281946	Intron
chr9	116292821	116293869	Intron
chr9	116499746	116500007	Distal Intergenic
chr9	116500145	116500517	Distal Intergenic
chr9	116500900	116501598	Distal Intergenic
chr9	116501678	116502158	Distal Intergenic
chr9	116525993	116526789	Distal Intergenic
chr9	116811411	116813616	3' UTR
chr9	116818150	116818499	3' UTR
chr9	116818539	116819608	3' UTR
chr9	116925963	116926647	Intron
chr9	116930162	116930750	Promoter
chr9	117060880	117061823	Intron

chr9	117106665	117107097	Intron
chr9	118441668	118442262	Distal Intergenic
chr9	118918885	118919977	Intron
chr9	119001687	119002275	Intron
chr9	119002643	119002965	Intron
chr9	119003063	119004234	Intron
chr9	119890198	119890690	Intron
chr9	119890759	119891065	Intron
chr9	119923027	119923235	Intron
chr9	120436125	120436717	Distal Intergenic
chr9	120479994	120480294	Distal Intergenic
chr9	120502325	120502924	Distal Intergenic
chr9	120648459	120649007	Distal Intergenic
chr9	120827948	120828255	Distal Intergenic
chr9	120870843	120871487	Distal Intergenic
chr9	121276076	121276449	Distal Intergenic
chr9	121276497	121276747	Distal Intergenic
chr9	121740631	121740972	Distal Intergenic
chr9	122006644	122007159	Intron
chr9	122012786	122013228	Intron
chr9	122013278	122014475	Intron
chr9	122016641	122017477	Intron
chr9	122017670	122017904	Intron
chr9	122019181	122019608	Intron
chr9	123032625	123034204	Distal Intergenic
chr9	123045871	123046983	Distal Intergenic
chr9	123078621	123078820	Distal Intergenic
chr9	123086617	123087138	Distal Intergenic
chr9	123092696	123092916	Distal Intergenic
chr9	123093067	123093554	Distal Intergenic
chr9	123110592	123111125	Distal Intergenic
chr9	123111842	123112602	Distal Intergenic
chr9	123112875	123113113	Distal Intergenic
chr9	123115830	123116846	Distal Intergenic
chr9	123122304	123123370	Distal Intergenic
chr9	123123645	123124922	Distal Intergenic
chr9	123144771	123145075	Distal Intergenic
chr9	123145111	123145447	Distal Intergenic
chr9	123145751	123145950	Distal Intergenic
chr9	123145992	123146304	Distal Intergenic
chr9	123146422	123146936	Distal Intergenic
chr9	123147471	123147858	Distal Intergenic
chr9	123148752	123149019	Downstream
chr9	123840102	123840468	Intron

chr9	124060930	124061129	Promoter
chrX	5564385	5565107	Distal Intergenic
chrX	5992980	5993184	Intron
chrX	6320789	6321075	Distal Intergenic
chrX	6764800	6765104	Distal Intergenic
chrX	6765309	6765908	Distal Intergenic
chrX	7234717	7235127	Intron
chrX	7235200	7236228	Intron
chrX	7248026	7248240	Intron
chrX	7250346	7252291	Exon
chrX	7299292	7299662	Distal Intergenic
chrX	7316981	7318294	Distal Intergenic
chrX	7340025	7341380	Distal Intergenic
chrX	7366650	7367101	Distal Intergenic
chrX	7418992	7419236	Distal Intergenic
chrX	7461086	7461352	Distal Intergenic
chrX	7722453	7722871	Distal Intergenic
chrX	7723082	7723346	Distal Intergenic
chrX	7778891	7779351	Distal Intergenic
chrX	7799409	7800649	Distal Intergenic
chrX	7800790	7801023	Distal Intergenic
chrX	7806295	7806538	Distal Intergenic
chrX	7806710	7807347	Distal Intergenic
chrX	8779534	8779744	Distal Intergenic
chrX	8798979	8799218	Distal Intergenic
chrX	8799508	8800480	Distal Intergenic
chrX	8800733	8800980	Distal Intergenic
chrX	9229725	9231403	Distal Intergenic
chrX	9236503	9240051	Distal Intergenic
chrX	9386642	9386841	Distal Intergenic
chrX	9682461	9682763	Intron
chrX	9682950	9683159	Exon
chrX	9685085	9686853	3' UTR
chrX	10294177	10294468	Distal Intergenic
chrX	10294760	10295059	Distal Intergenic
chrX	10295109	10295398	Distal Intergenic
chrX	10295830	10296251	Distal Intergenic
chrX	10567730	10568636	Intron
chrX	10572509	10572708	Intron
chrX	10572774	10573004	Intron
chrX	10578270	10578502	Intron
chrX	10768087	10768342	Intron
chrX	11105440	11105676	Intron
chrX	11106015	11106214	Intron

chrX	11142682	11142975	Distal Intergenic
chrX	11372013	11372289	Intron
chrX	11644906	11645327	Intron
chrX	11923950	11924800	Distal Intergenic
chrX	11942980	11943268	Distal Intergenic
chrX	12107649	12108214	Distal Intergenic
chrX	12178867	12179308	Intron
chrX	12250468	12250751	Intron
chrX	12250841	12251219	Intron
chrX	12337787	12338032	Intron
chrX	12338070	12338860	Intron
chrX	12338933	12339721	Intron
chrX	12339872	12340087	Intron
chrX	12399404	12400524	Intron
chrX	12447629	12447917	Intron
chrX	12503686	12504348	Intron
chrX	12536534	12537113	Intron
chrX	12555028	12555386	Intron
chrX	12619242	12619470	Intron
chrX	12648886	12649264	Intron
chrX	12649492	12649741	Intron
chrX	12649873	12650295	Intron
chrX	12807316	12807515	Distal Intergenic
chrX	12807560	12807911	Distal Intergenic
chrX	12857172	12858702	Distal Intergenic
chrX	12859201	12859499	Distal Intergenic
chrX	12859697	12859949	Distal Intergenic
chrX	12975540	12975749	Distal Intergenic
chrX	13029835	13030037	Distal Intergenic
chrX	13030238	13030946	Distal Intergenic
chrX	13038979	13039463	Distal Intergenic
chrX	13039737	13040383	Distal Intergenic
chrX	13084090	13084538	Distal Intergenic
chrX	13100856	13103070	Distal Intergenic
chrX	16175033	16176441	Distal Intergenic
chrX	16723067	16723266	Intron
chrX	16724183	16724424	Intron
chrX	16767848	16768886	Intron
chrX	17148463	17149034	Intron
chrX	17149085	17149353	Intron
chrX	17740156	17741332	Intron
chrX	18417706	18418070	Distal Intergenic
chrX	18739716	18741489	Intron
chrX	18742702	18745024	Intron

chrX	18745407	18745935	Intron
chrX	19264313	19265464	Distal Intergenic
chrX	19314123	19314373	Distal Intergenic
chrX	19314516	19314715	Distal Intergenic
chrX	19314775	19314989	Distal Intergenic
chrX	19481192	19481801	Intron
chrX	19481926	19482251	Intron
chrX	19941732	19941942	Intron
chrX	19995452	19996159	Distal Intergenic
chrX	20003467	20003700	Downstream
chrX	20194428	20194643	Exon
chrX	20194684	20195277	Exon
chrX	20240020	20240840	Intron
chrX	20264089	20264466	Intron
chrX	20264503	20264892	Intron
chrX	20393701	20394291	Distal Intergenic
chrX	20394376	20394651	Distal Intergenic
chrX	20394828	20395077	Distal Intergenic
chrX	20444761	20445898	Distal Intergenic
chrX	20446054	20447062	Distal Intergenic
chrX	20492189	20492402	Distal Intergenic
chrX	20538804	20539104	Distal Intergenic
chrX	21142675	21143203	Distal Intergenic
chrX	21144234	21144827	Distal Intergenic
chrX	21155647	21156379	Distal Intergenic
chrX	21156591	21157334	Distal Intergenic
chrX	33468297	33469096	Distal Intergenic
chrX	33470236	33471252	Distal Intergenic
chrX	33618632	33618939	Distal Intergenic
chrX	33619121	33619417	Distal Intergenic
chrX	33654081	33654442	Distal Intergenic
chrX	33654957	33656264	Distal Intergenic
chrX	33669000	33669877	Distal Intergenic
chrX	33925621	33926270	Distal Intergenic
chrX	34137716	34138031	Distal Intergenic
chrX	34335888	34336121	Distal Intergenic
chrX	34347525	34347821	Distal Intergenic
chrX	34414488	34414742	Distal Intergenic
chrX	34622767	34622966	Distal Intergenic
chrX	34624057	34624262	Distal Intergenic
chrX	34624689	34625157	Distal Intergenic
chrX	34649969	34650286	Intron
chrX	34666995	34667201	Intron
chrX	35395446	35395749	Distal Intergenic

chrX	35395829	35396250	Distal Intergenic
chrX	35396702	35397538	Distal Intergenic
chrX	35514429	35514645	Distal Intergenic
chrX	35538305	35539509	Distal Intergenic
chrX	35542689	35543525	Distal Intergenic
chrX	35781987	35782191	Distal Intergenic
chrX	35795565	35797588	Distal Intergenic
chrX	35798668	35799479	Distal Intergenic
chrX	35826560	35826759	Distal Intergenic
chrX	35917633	35918048	Distal Intergenic
chrX	35953519	35954369	Intron
chrX	39265115	39265392	Distal Intergenic
chrX	39277419	39278300	Distal Intergenic
chrX	39317486	39318159	Distal Intergenic
chrX	39322855	39324334	Distal Intergenic
chrX	39421411	39421707	Distal Intergenic
chrX	39421974	39422929	Distal Intergenic
chrX	39492545	39493762	Distal Intergenic
chrX	39502665	39503108	Distal Intergenic
chrX	39503907	39505290	Distal Intergenic
chrX	42460138	42460351	Distal Intergenic
chrX	45283123	45284337	Distal Intergenic
chrX	45537995	45538232	Distal Intergenic
chrX	45538424	45538817	Distal Intergenic
chrX	45606588	45606896	Promoter
chrX	45610050	45610299	Distal Intergenic
chrX	45806616	45806842	Distal Intergenic
chrX	45813611	45813812	Distal Intergenic
chrX	45819240	45821053	Distal Intergenic
chrX	47988351	47988558	Intron
chrX	48176957	48177723	Distal Intergenic
chrX	48272116	48272552	Promoter
chrX	48282742	48284252	Distal Intergenic
chrX	48343913	48345511	3' UTR
chrX	48370469	48371049	5' UTR
chrX	48372056	48372667	Exon
chrX	48521061	48521493	Distal Intergenic
chrX	48521815	48522181	Distal Intergenic
chrX	48538213	48539289	Distal Intergenic
chrX	48585635	48586428	Distal Intergenic
chrX	48727041	48727440	Distal Intergenic
chrX	48859361	48860250	Promoter
chrX	48860572	48860790	Distal Intergenic
chrX	49056123	49056592	Promoter

chrX	49517481	49517693	Distal Intergenic
chrX	49573751	49574372	Distal Intergenic
chrX	50266050	50266354	Distal Intergenic
chrX	50340953	50341182	Intron
chrX	50341341	50342683	Exon
chrX	50468397	50468617	Intron
chrX	53260351	53261438	Downstream
chrX	53930920	53933003	Distal Intergenic
chrX	60399410	60399896	Distal Intergenic
chrX	60715706	60717329	Distal Intergenic
chrX	65665530	65665790	Distal Intergenic
chrX	65931896	65932095	Distal Intergenic
chrX	65932173	65932373	Distal Intergenic
chrX	66585241	66585555	Distal Intergenic
chrX	67639084	67639484	Intron
chrX	67639595	67639951	Intron
chrX	69289693	69290288	Distal Intergenic
chrX	69543338	69543537	Intron
chrX	70184712	70185829	Distal Intergenic
chrX	70919751	70920524	Intron
chrX	70920573	70920840	Exon
chrX	70923608	70924386	Intron
chrX	70924428	70924776	Intron
chrX	70927089	70927726	Intron
chrX	70961078	70961277	Distal Intergenic
chrX	70961654	70961976	Distal Intergenic
chrX	70963793	70964889	Distal Intergenic
chrX	71081321	71081722	Distal Intergenic
chrX	71160149	71160358	Intron
chrX	71167396	71167707	Intron
chrX	71211068	71213256	Intron
chrX	71489132	71490147	Downstream
chrX	71490307	71490526	Downstream
chrX	71492242	71492441	Downstream
chrX	71492977	71493191	Exon
chrX	71494073	71495226	3' UTR
chrX	71495275	71495816	Exon
chrX	71500396	71500940	Distal Intergenic
chrX	71514905	71516893	Distal Intergenic
chrX	71527055	71527444	Promoter
chrX	71528111	71528602	Distal Intergenic
chrX	71614091	71614306	Intron
chrX	71638137	71638533	Intron
chrX	71638646	71639290	Intron

chrX	71669350	71672220	Intron
chrX	71672467	71672681	Intron
chrX	71674075	71675257	Intron
chrX	72271388	72271626	Distal Intergenic
chrX	72273170	72273451	Distal Intergenic
chrX	72273518	72273786	Distal Intergenic
chrX	72273850	72274230	Distal Intergenic
chrX	72277237	72277455	Distal Intergenic
chrX	72277516	72277717	Distal Intergenic
chrX	72375861	72376303	Distal Intergenic
chrX	72381901	72383045	Distal Intergenic
chrX	72383316	72383515	Distal Intergenic
chrX	72661506	72661792	Distal Intergenic
chrX	72782203	72782402	Promoter
chrX	72783323	72783581	Promoter
chrX	73009450	73009764	Distal Intergenic
chrX	73077950	73080483	Distal Intergenic
chrX	73080526	73080810	Distal Intergenic
chrX	73087565	73090159	Distal Intergenic
chrX	73091013	73091271	Distal Intergenic
chrX	73091475	73091709	Distal Intergenic
chrX	73119324	73119629	Distal Intergenic
chrX	73120027	73120431	Distal Intergenic
chrX	74756994	74757425	Distal Intergenic
chrX	74861772	74862379	Distal Intergenic
chrX	74862808	74863267	Distal Intergenic
chrX	74863358	74863804	Distal Intergenic
chrX	75041051	75041256	Distal Intergenic
chrX	76896368	76896612	Intron
chrX	83016706	83017061	Distal Intergenic
chrX	90779815	90780394	Distal Intergenic
chrX	90780451	90780687	Distal Intergenic
chrX	90877129	90877494	Distal Intergenic
chrX	90879719	90880126	Distal Intergenic
chrX	90919832	90920204	Distal Intergenic
chrX	90920525	90921209	Distal Intergenic
chrX	91305953	91306179	Intron
chrX	91363365	91364248	Intron
chrX	91366433	91368283	Intron
chrX	91384592	91384827	Intron
chrX	91397618	91398021	Intron
chrX	91414946	91415562	Intron
chrX	91423064	91423263	Intron
chrX	92361216	92361415	Distal Intergenic

chrX	92361616	92361840	Distal Intergenic
chrX	93143775	93144120	Distal Intergenic
chrX	93291414	93293844	Distal Intergenic
chrX	93302143	93303132	Distal Intergenic
chrX	93303292	93303988	Distal Intergenic
chrX	93316119	93318425	Distal Intergenic
chrX	93320862	93321082	Distal Intergenic
chrX	93324932	93325483	Distal Intergenic
chrX	93325527	93326290	Distal Intergenic
chrX	93326359	93326656	Distal Intergenic
chrX	93326696	93326997	Distal Intergenic
chrX	93328030	93328633	Distal Intergenic
chrX	93328693	93330861	Distal Intergenic
chrX	94561095	94562068	Distal Intergenic
chrX	94568019	94568293	Distal Intergenic
chrX	94568341	94568599	Distal Intergenic
chrX	94568828	94569071	Distal Intergenic
chrX	94569119	94569569	Distal Intergenic
chrX	94569618	94570311	Distal Intergenic
chrX	94570907	94572542	Distal Intergenic
chrX	94572611	94572869	Distal Intergenic
chrX	96331187	96331820	Intron
chrX	96331967	96332422	Intron
chrX	96332579	96332902	Intron
chrX	96333018	96334391	Intron
chrX	96334793	96335028	Intron
chrX	96335515	96336872	Intron
chrX	96336920	96337699	Intron
chrX	96338696	96339041	Intron
chrX	96339097	96340038	Intron
chrX	96618040	96618480	Intron
chrX	96618684	96619189	Intron
chrX	96648619	96648823	Intron
chrX	96717720	96718447	Intron
chrX	96799482	96805051	Intron
chrX	96805137	96805903	Intron
chrX	96805961	96806258	Intron
chrX	97821100	97821360	Distal Intergenic
chrX	97882896	97883449	Distal Intergenic
chrX	97911220	97911651	Distal Intergenic
chrX	97911711	97912144	Distal Intergenic
chrX	97912188	97912459	Distal Intergenic
chrX	97933655	97933913	Distal Intergenic
chrX	98624342	98624757	Distal Intergenic

chrX	98625243	98625669	Distal Intergenic
chrX	98835334	98835815	Intron
chrX	98962473	98962672	Intron
chrX	99383860	99384084	Distal Intergenic
chrX	99384439	99384638	Distal Intergenic
chrX	99579114	99579326	Intron
chrX	99862020	99862784	Distal Intergenic
chrX	100689266	100689572	Distal Intergenic
chrX	100704502	100705059	Distal Intergenic
chrX	100709942	100710531	Distal Intergenic
chrX	100710572	100711340	Distal Intergenic
chrX	100807380	100807668	Intron
chrX	100811546	100812036	Distal Intergenic
chrX	100812411	100812610	Distal Intergenic
chrX	100843620	100844572	Distal Intergenic
chrX	100845839	100846136	Distal Intergenic
chrX	100846736	100847150	Distal Intergenic
chrX	100847519	100847756	Distal Intergenic
chrX	100847820	100848786	Distal Intergenic
chrX	100985397	100986736	Distal Intergenic
chrX	101175354	101176580	Intron
chrX	101678498	101678893	Intron
chrX	101682806	101683085	Intron
chrX	101683127	101683612	Intron
chrX	101683704	101683985	Intron
chrX	103124328	103124728	Distal Intergenic
chrX	103381398	103381825	Intron
chrX	103381969	103382274	Intron
chrX	103382483	103382690	Intron
chrX	103382736	103383764	Intron
chrX	103383808	103384064	Intron
chrX	106028222	106028463	Exon
chrX	109812340	109812654	Distal Intergenic
chrX	115931578	115931813	Distal Intergenic
chrX	126091455	126092009	Distal Intergenic
chrX	126092082	126092334	Distal Intergenic
chrX	131075502	131075748	Distal Intergenic
chrX	131120283	131120585	Distal Intergenic
chrX	132779679	132779902	Intron
chrX	132802400	132802861	Intron
chrX	133275282	133275625	Distal Intergenic
chrX	133555758	133556629	Intron
chrX	136532500	136533027	Distal Intergenic
chrX	137077547	137077761	Distal Intergenic

chrX	137107362	137107962	Distal Intergenic
chrX	137837741	137837959	Intron
chrX	138771149	138771451	Intron
chrX	138825197	138825488	Intron
chrX	139332077	139332349	Distal Intergenic
chrX	139332388	139333512	Distal Intergenic
chrX	139348070	139348457	Distal Intergenic
chrX	147022897	147023208	Intron
chrX	147511088	147511581	Distal Intergenic
chrX	148236856	148237103	Distal Intergenic
chrX	148238221	148238557	Distal Intergenic
chrX	149138008	149138543	Intron
chrX	149138735	149139113	Intron
chrX	149139284	149139575	Intron
chrX	149151654	149152097	Intron
chrX	149152164	149152648	Intron
chrX	149196782	149197401	Intron
chrX	149197439	149197638	Intron
chrX	151651187	151651583	Distal Intergenic
chrX	151697081	151697463	Distal Intergenic
chrX	156246649	156247651	Distal Intergenic
chrX	156247729	156248551	Distal Intergenic
chrX	156270508	156271199	Distal Intergenic
chrX	156278147	156278789	Distal Intergenic
chrX	156279034	156280312	Distal Intergenic
chrX	156286751	156287097	Distal Intergenic
chrX	156288380	156288773	Distal Intergenic
chrX	160450798	160451173	Distal Intergenic
chrX	160451267	160452068	Distal Intergenic
chrX	160573485	160573809	Distal Intergenic
chrX	160720827	160722663	Distal Intergenic
chrX	160817521	160817980	Distal Intergenic
chrX	160818113	160818417	Distal Intergenic
chrX	160831624	160832185	Distal Intergenic
chrX	160832250	160832482	Distal Intergenic
chrX	163099039	163099362	Distal Intergenic
chrX	163203275	163204348	Distal Intergenic
chrX	163243592	163243948	Distal Intergenic
chrX	163409328	163409898	Distal Intergenic
chrX	163410170	163410461	Distal Intergenic
chrX	163445325	163446044	Distal Intergenic
chrX	163529872	163530408	Distal Intergenic
chrX	163589691	163589956	Distal Intergenic

Supplementary Table 4. Summary of Primers

Primers used for real-time quantitative PCR (5' to 3')		
	Forward primer sequence	Reverse primer sequence
Mouse *Sox2*	GCGGAGTGGAAACT TTTGTCC	GGGAAGCGTGTACTTA TCCTTCT
Human *SMAD4*	CTCATGTGATCTAT GCCCGTC	AGGTGATACAACTCGT TCGTAGT
Human *SOX2*	CACAACTCGGAGAT CAGCAA	CTCCGGGAAGCGTGTA CTTA
GAPDH	AATCCCATCACCAT CTTCCA	TGGACTCCACGACGTA CTCA
Primers (5' to 3') used for PCR to verify the knockout of *Sox2/SOX2*-associated loops targeted regions		
Mouse *Sox2*-associated loop targeted enhancer region knockout	TCAGCTTCAACGAA ACTCTG	GGCCAATAAATGGCAA GAAG
Human *SOX2*-associated loop targeted enhancer region knockout	GTTTTGGTGTCACCC ATTGGAG	ATGCAAATTGTTAATG CCCAAAGA
Primers (5' to 3') used for 3C-PCR and 3C-qPCR		
Mouse *Sox2*-associated loop	GGTCCTCCCAAACC TAATGT	GGTGGTGTGCCATTGT TTCT
Human *SOX2*-associated loop	CCGGGTTCCCAAGA ACTAAA	ATGCAAATTGTTAATG CCCA

Primers (5' to 3') used for ChIP-PCR and ChIP-qPCR		
Mouse *Sox2* promoter region	GGCTTGGGTCTAACTTCTCG	GTGGTGTGCCATTGTTTCTG
Mouse *Sox2*-associated loop targeted enhancer region	GGGACGAGTGTGCTTTATGT	AGCAGAGCATCATGGGAATG

Supplementary Table 5. Summary of Guide RNA (gRNA) Sequences

Summary of guide RNA (gRNA) sequences (5' to 3')			
	vector to ligation	gRNA oligo1	gRNA oligo2
Mouse *Smad4* gRNA	LentiCRISPRV2	CACCGGCCAAGTAATCGCGCATCAA	AAACTTGATGCGCGATTACTTGGCC
Human *SMAD4* gRNA		CACCGAACTCTGTACAAAGACCGCG	AAACCGCGGTCTTTGTACAGAGTTC
Human *PTEN* gRNA		CACCGACCGCCAAATTTAATTGCAG	AAACCTGCAATTAAATTTGGCGGTC
Mouse *Sox2*-associated loop targeted enhancer region dual-gRNA	Plenti-H1-hU6-hygro-dual-gRNA	ATTTCTAGCTCTAAAACACATTACGGCGAAAAGAATCCGGGAAAGAGTGGTCTC	ATTTCTAGCTCTAAAACCTCGAGCTGCTAAAAGTTCGCGGTGTTTCGTCCTTTCC
Mouse *p300* dual-gRNA		ATTTCTAGCTCTAAAACTCTCGGCGTCCGCCAGCGATCGGGAAAGAGTGGTCTC	ATTTCTAGCTCTAAAACGCCGGTAAAGTGCCTCCAATCGGTGTTTCGTCCTTTCC

Human *SOX2*-associated loop targeted enhancer region dual-gRNA	ATTTCTAGCTCTAAAACTCCACTTACATGCTTCGGTGCGGGAAAGAGTGGTCTC	ATTTCTAGCTCTAAAACCGTTACACCCAATATCTTCCCGGTGTTTCGTCCTTTCC

Supplementary Table 6. Summary of siRNA Sequences

Summary of guide siRNA sequences (5' to 3')		
	sence	anti-sence
si-NC (Mouse)	UUCUCCGACAGUGUCACGU(dT)(dT)	ACGUGACACUGUCGGAGAA(dT)(dT)
si-*Smad4* (Mouse)	CAGUAUGCGUUUGACUUAA(dT)(dT)	UUAAGUCAAACGCAUACUG(dT)(dT)
si-NC (Human)	UUCUCCGAACGUGUCACGU(dT)(dT)	ACGUGACACGUUCGGAGAA(dT)(dT)
si-*SMAD4* (Human)	CAGAGUUACUACUUAGACA(dT)(dT)	UGUCUAAGUAGUAACUCUG(dT)(dT)

Supplementary Table 7. Summary of Antisense Oligonucleotides (ASO) Sequences

Summary of guide siRNA sequences (5' to 3')	
	sence
NC ASO	GCGUATTATAGCCGAUUAAC
Sox2-associated enhancer region targeted by ASO	AATGCACATAAAGCACACTC
